# Asymmetric radical carboesterification of dienes

**DOI:** 10.1038/s41467-021-26843-2

**Published:** 2021-11-18

**Authors:** Xiaotao Zhu, Wujun Jian, Meirong Huang, Daliang Li, Yajun Li, Xinhao Zhang, Hongli Bao

**Affiliations:** 1 State Key Laboratory of Structural Chemistry, Key Laboratory of Coal to Ethylene Glycol and Its Related Technology, Center for Excellence in Molecular Synthesis, Fujian Institute of Research on the Structure of Matter, Chinese Academy of Sciences, 155 Yangqiao Road West, Fuzhou, Fujian, 350002 P. R. of China; 2 Shenzhen Bay Laboratory, State Key Laboratory of Chemical Oncogenomics, Peking University Shenzhen Graduate School, Shenzhen, 518055 P. R. of China; 3 Fujian Key Laboratory of Innate Immune Biology, Biomedical Research Center of South China, Key Laboratory of OptoElectronic Science and Technology for Medicine of Ministry of Education, College of Life Sciences, Fujian Normal University, Fujian, China; 4 University of Chinese Academy of Sciences, Beijing, 100049 P. R. of China

**Keywords:** Asymmetric catalysis, Synthetic chemistry methodology

## Abstract

The straightforward strategy of building a chiral C-O bond directly on a general carbon radical center is challenging and stereocontrol of the reactions of open-chain hydrocarbon radicals remains a largely unsolved problem. Advance in this elementary step will spur the development of asymmetric radical C-O bond construction. Herein, we report a copper-catalyzed regioselective and enantioselective carboesterification of substituted dienes using alkyl diacyl peroxides as the source of both the carbon and oxygen substituents. The participation of external acids in this reaction substantially extends its applicability and leads to structurally diverse allylic ester products. This work represents the advance in the key elementary reaction of intermolecular enantioselective construction of C-O bond on open-chain hydrocarbon radicals and may lead to the discovery of other asymmetric radical reactions.

## Abstract (web summary)

Stereocontrol of C–O bond formation from a carbon-based radical is very difficult due to the rapid inversion of the carbon radical. Here the authors present a method to form chiral esters from conjugated dienes with copper and chiral PyBox ligands, likely proceeding via an allylic radical.

## Introduction

**Fig. 1:**
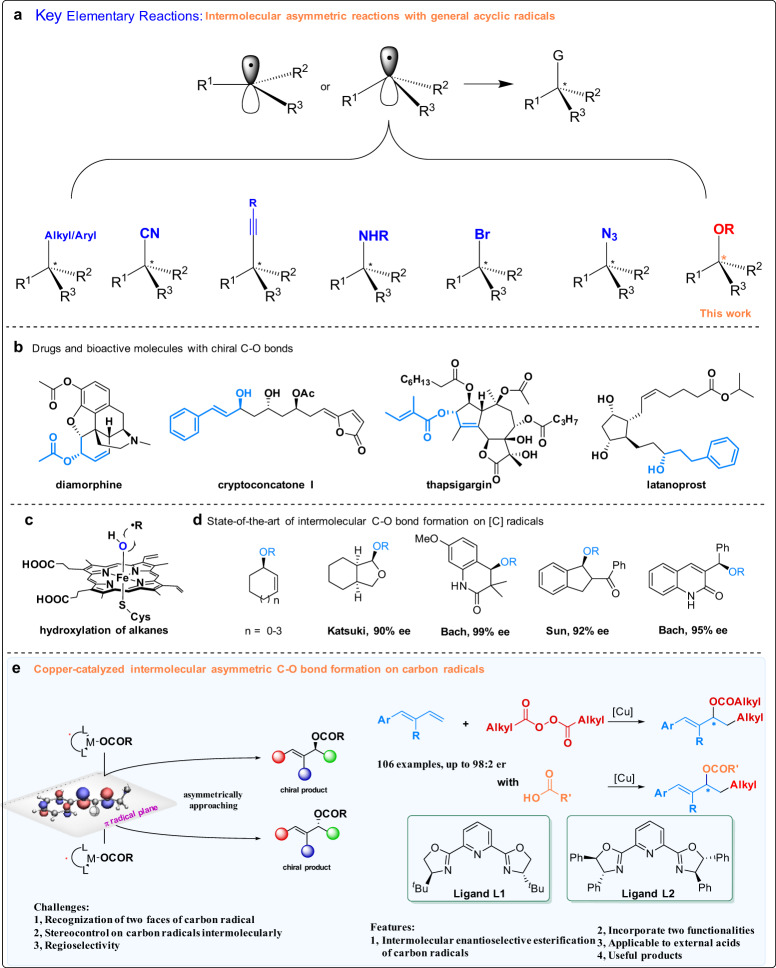
Elementary radical reactions and asymmetric radical C–O bond formation. **a** Key elementary enantioselective radical reactions. **b** Bioactive molecules with chiral C–O bonds. **c** Enzymatic hydroxyl group transfer. **d** State-of-the-art of intermolecular C–O bond formation on carbon radicals. **e** This work.

Direct enantioselective creation of a C–X bond on a carbon radical center is a conceptually simple, but important elementary reaction. The free nature of a radical, its short lifetime, high reactivity, and low activation energy make stereocontrol on the radical center extremely difficult. The two faces of a planar radical or the two invertomers of pyramidal radicals must be recognized and the radical must be rapidly converted into the desired products before undesirable side reactions can take place. Recently, the creation of stereoselective C–C, C–N, C–Br bonds on a radical center has been incrementally realized by Fu^1–5^, Liu^6–9^, Ready^10^, Reisman^11,12^, and others^13–20^ (Fig. 1a), and these breakthroughs have inspired the field, leading to significant progress and discovery of many relevant and useful reactions.

The construction of a chiral C–O bond directly on a carbon radical center is a powerful elementary reaction. This elementary reaction is highly useful because it could deliver compounds containing chiral C–O bonds which are ubiquitous in natural products and bioactive molecules (Fig. 1b) but stereocontrol remains an important unsolved problem.

Some of the early developments in asymmetric oxygenation of carbon radicals stem from a seminal report by Kharasch and Sosnovsky in the late 1950s^21,22^ on the allylic oxidation reactions of alkenes^23,24^. It was found that only 5–8 membered cyclic alkenes can afford the desired products with moderate to high enantioselectivity, and stereocontrol of open-chain alkenes was not achieved^25,26^. Inspired by ubiquitous metabolic C–H hydroxylation reactions of hydrocarbons in cytochrome P450 (Fig. 1c)^5–7^, Groves et al.^27^ pioneered the bioinspired asymmetric hydroxylation of hydrocarbon radicals, and the reactions that were discovered provided the corresponding products with moderate enantioselectivity. Subsequently, several reactions achieving the asymmetric hydroxylation of carbon radical centers with high enantioselectivity were reported by Katsuki^28,29^, Bach^30,31^, Sun^32^, and others^33^ (Fig. 1d).

Asymmetric carbooxygenative difunctionalization of C–C double bonds is an efficient reaction that simultaneously incorporates two functionalities and can quickly build complex molecules from bulk chemicals^34–46^. In the category of radical asymmetric transformations, Buchwald et al. developed a two-component asymmetric oxyfunctionalization of alkenes with intramolecular carboxylic acid groups^36,37,40^, and Liu et al. reported enantioselective oxyfunctionalization of alkenes with linked alcohols and oximes^39,41,44,45^. These reports provide a solution to the long-standing unresolved problems concerning the stereocontrol of acyclic radicals, but with intramolecular oxygen functionalities.

In spite of these breakthroughs in the last decades, chiral C–O bond construction on acyclic carbon radicals with an intermolecular oxygen source remains an important and unsolved problem in the field of asymmetric catalysis. A reason underlying this challenge is that classic interactants, such as covalent bonds, ionic bonds, dative bonds, and hydrogen bonds, which help catalysts to recognize the substrate and control the enantioselectivity, are less available between a carbon radical and another reaction partner in this elementary reaction mode. In the reaction of an acyclic carbon radical, especially an intermolecular reaction, this challenge is even more severe.

Herein, we report our work on asymmetric carboesterification of dienes (Fig. 1e). This type of reaction represents an advance on the elementary reaction, and also on the stereocontrol of acyclic radicals with intermolecular oxygen functionalities. This work provides an important strategy for stereocontrol at free radical centers and supports the direct formation of valuable chiral allylic esters that are often biologically important (Fig. 1b). The reaction products contain one additional double bond which can be subsequently used to produce versatile functionalities. Notably, during the reviewing process of this paper, the Chen and Xiao group reported elegant photoinduced copper-catalyzed asymmetric carboesterifications of dienes with redox-active oxime esters^47–49^.

## Results

### Reaction development

**Fig. 2:**
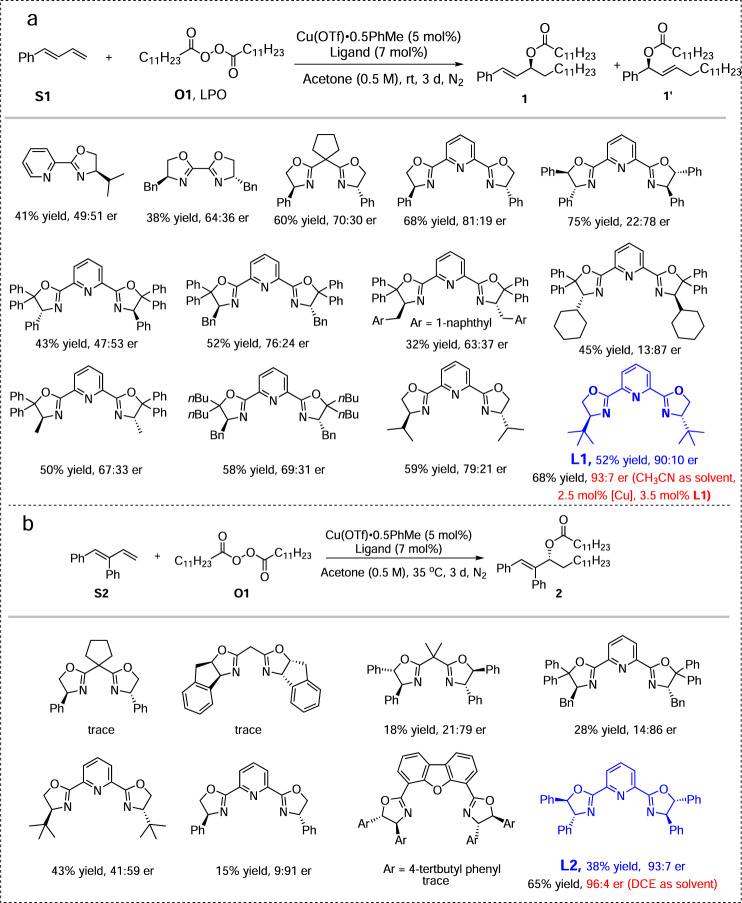
Reaction condition optimizations. **a** Reaction condition optimizations with diene S1. **b** Reaction condition optimizations with diene S2.

We began the studies with (*E*)-buta-1,3-dien-1-ylbenzene (**S1**) and lauroyl peroxide (LPO, **O1**) as substrates. The optimal conditions were identified after extensive studies with PyBox (**L1**) and CuOTf• 1/2PhMe shown in Fig. 2a (for details of optimization of the conditions, see Supplementary Tables 1–5). A trace amount of the 1,4-addition product (**1’**) was observed in all the cases and interestingly, for disubstituted dienes, the tridentate ligand (**L2**) was found to provide better enantioselective and regioselective control than ligand **L1** when dichloroethane (DCE) was used as the solvent and the reaction delivers the desired product (**2**) without the formation of its regioisomer (see Fig. 2b). In some reactions with low yields, the substrate diene, alkyl Heck-type product, and some unidentified compounds can be observed by gas chromatography mass spectroscopy analysis (Please see Supplementary Figure 2 for details).

**Fig. 3:**
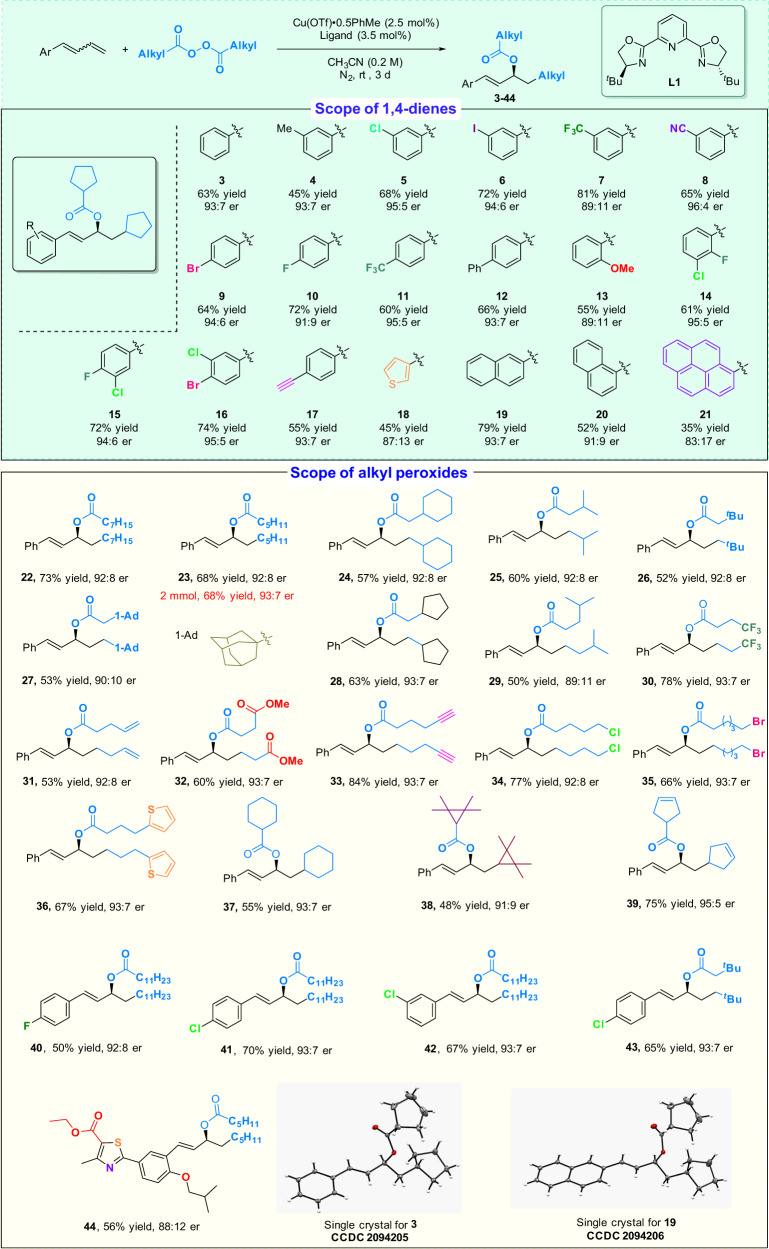
Substrate scope for carboesterification of monoaryl substituted dienes. Reaction conditions: diene (0.20 mmol, 1 equiv), peroxide (0.4 mmol, 2 equiv), Cu(OTf)·0.5PhMe (2.5 mol%), **L1** (3.5 mol %), and CH_3_CN (1 mL, 0.2 M) at rt for 3 d under nitrogen atmosphere. For dienes **41** and **42**, the reactions were performed with Cu(OTf)·0.5PhMe (5 mol%) and **L1** (7 mol %) for 5d.

With the optimal reaction conditions in hand, a series of dienes and alkyl diacyl peroxides were selected to examine the tolerance of substitution patterns and functional groups in the asymmetric radical carboesterification process (Fig. 3). A variety of enantioenriched allylic esters (**3**–**44**) were obtained under the optimal reaction conditions and even substrates as *E/Z* isomers were found to only provide the corresponding (*E)*-selective products. Functional groups such as alkyl (**4**), cyano (**8**), alkyloxy (**13**), and trifluoromethyl (**7** and **11**) are tolerated, as are halides (**5**, **6**, **9**, and **10**) that can be used subsequently in well-developed cross-coupling reactions. The diene substrates with a terminal C–C triple bond (**17**), a disubstituted phenyl group (**14**, **15**, and **16**), a thienyl group (**18**), a naphthyl group (**19** and **20**), and a pyrenyl group (**21**) can provide the corresponding products with a er value as high as 95:5. A screening of the alkyl diacyl peroxides revealed that not only primary alkyl radicals but secondary alkyl radicals can participate in the reaction to afford the corresponding chiral allylic esters (**37–39**) with a high er. Other structural fragments, such as long alkyl chains (**22** and **23**), cycloalkyl groups (**24**, **27**, **28**, **37**, **38**, and **39**), an ester group (**32**), a heteroaryl group (**36** and **44**), or a terminal C–C triple bond (**33**) are tolerated. However, 1-phenylethyl buta-1,3-diene, as an example of alkyl-substituted diene, provided the mixture of 1,2- and 1,4-carboesterrification products in 58% yield and low enantioselectivity (The ratio of the products is 1:0.51:0.13, and the ee values are 44%, 44%, and 27%, respectively). The wide group tolerance makes this reaction a valuable process for the direct construction of useful chiral compounds. The absolute stereochemistry of products **3** and **19** was confirmed as *S* by single-crystal x-ray crystallography.

**Fig. 4:**
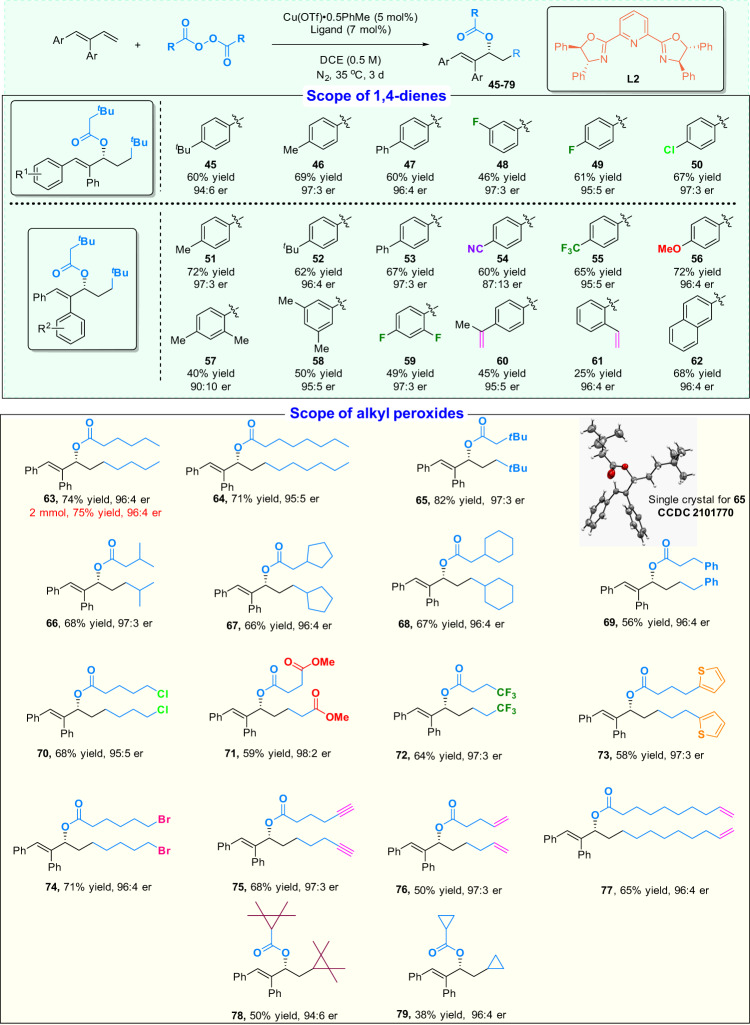
Substrate scope for carboesterification of diaryl substituted dienes. Reaction conditions: diene (0.2 mmol, 1 equiv), peroxide (0.24 mmol, 1.2 equiv), Cu(OTf)·0.5PhMe (5 mol%), **L2** (7 mol %), and DCE (0.4 mL, 0.5 M) at 35 °C for 3 d under nitrogen atmosphere. For **68**, **70**, **74**, **75**, and **76**, peroxide (0.6 mmol, 3 equiv) was used instead.

Disubstituted dienes are challenging reaction partners for this asymmetric radical carboesterification. A bulkier PyBox ligand (**L2**) can successfully address this issue. As shown in Fig. 4, an extensive examination of the substrate scope of dienes and alkyl diacyl peroxides was conducted. The corresponding enantioenriched allylic esters (**45**–**79**) with various functional groups can be obtained with good to high enantioselectivity. The compatibility of a terminal C=C double bond (**60** and **61**) showed a remarkable chemoselectivity when comparing the reactivity of the conjugate dienes and the isolated C=C double bond in this reaction. Notably, the reaction is completely regioselective for 1,2-addition of the diaryl substituted dienes and high enantioselectivities were observed in the majority of cases.

### Mechanistic considerations

**Fig. 5:**
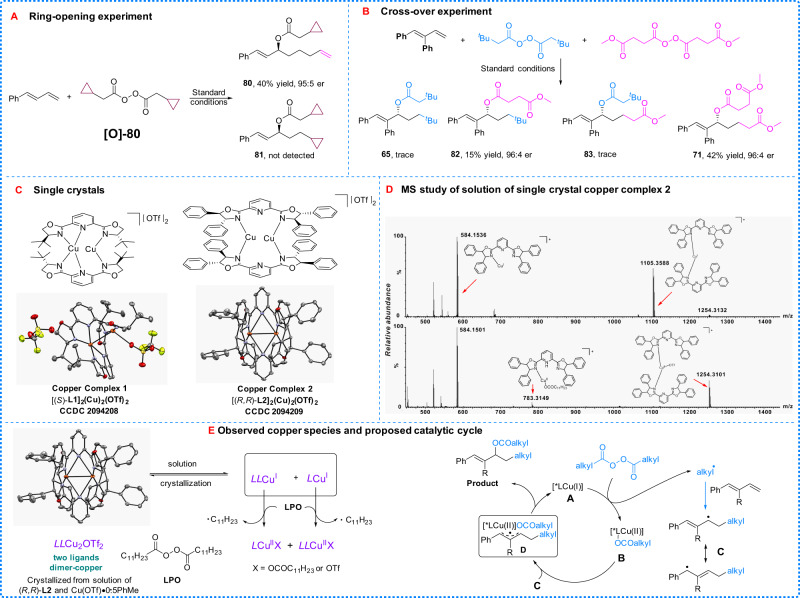
Mechanistic studies. **a** Ring-opening reaction with 2-cyclopropylacetic peroxyanhydride. **b** Crossover reaction of two different peroxides. **c** Single crystals of dimer copper-dimer ligand complex (copper is in orange). **d** Mass spectrometric studies of copper complex 2. **e** Mass spectrometric studies and a plausible catalytic mechanism.

Studies were conducted to probe the radical nature and the mechanism of the process. First, a radical clock reaction with 2-cyclopropylacetic peroxyanhydride (**[O]-80**) was conducted and the reaction afforded the ring-opened product (**80**) in 40% yield, which suggests that the corresponding cyclopropylcarbinyl radical is an intermediate that undergoes fast ring-opening to give the 3-butenyl radical (Fig. 5a).

**Fig. 6:**
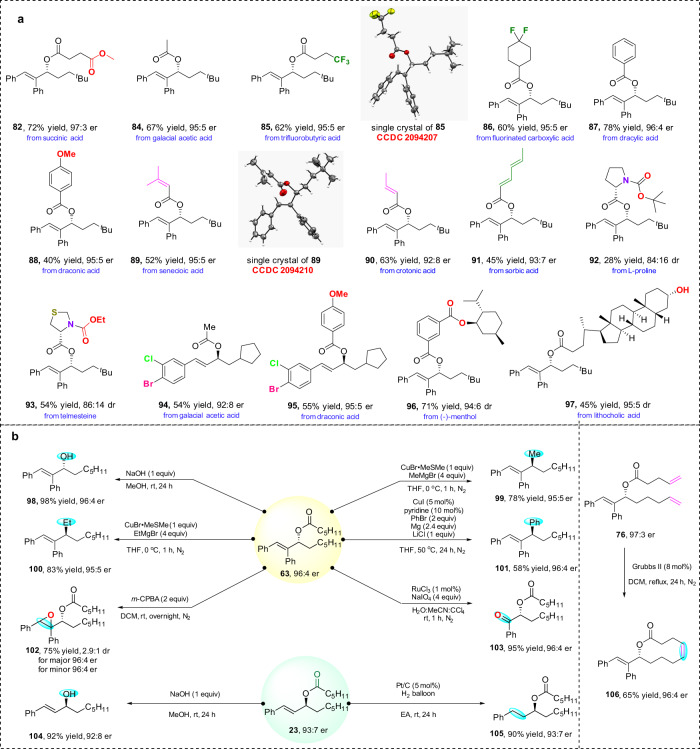
Synthetic applications. **a** Application to external acids. Reaction conditions: diene (0.2 mmol, 1 equiv), peroxide (0.24 mmol, 1.2 equiv), external acid (0.22 mmol 1.1 equiv), Cu(OTf)·0.5PhMe (5 mol%), **L2** (7 mol%), and DCE (0.4 mL, 0.5 M) at 50 °C for 3 d under nitrogen atmosphere. For **92** and **97**: acid (0.2 mmol 1 equiv), diene (0.4 mmol, 2 equiv), peroxide (0.4 mmol, 2 equiv), Cu(OTf)·0.5PhMe (5 mol%), **L2** (7 mol%), and DCE (1 mL, 0.2 M) at 50 °C for 3 d under nitrogen atmosphere. For **94** and **95**: diene (0.20 mmol, 1 equiv), peroxide (0.4 mmol, 2 equiv), external acid (0.3 mmol 1.5 equiv), Cu(OTf)·0.5PhMe (2.5 mol%), **L1** (3.5 mol%), and CH_3_CN (1 mL, 0.2 M) at rt for 3 d under nitrogen atmosphere. **b** Further transformation of chiral allylic esters.

Radical trap experiments were investigated and the model reaction was found to be inhibited by radical inhibitors such as 2,6-di-*tert*-butyl-4-methylphenol (butylated hydroxy toluene, BHT) and 2,2,6,6-tetramethylpiperidine-1-oxyl (TEMPO) (for details, see mechanistic studies section of Supplementary Information). In addition, a crossover experiment with two different peroxides was studied (Fig. 5b). The isolation of products **71** and **82** from this crossover reaction suggested that a stepwise process might be involved in the reaction. This observation inspired us to further study the behavior of externally added carboxylic acids, obtaining the results summarized in Fig. 6a.

Two copper complexes were obtained as single crystals, shown in Fig. 5c. Reactions catalyzed by the two complexes afforded the desired products with identical yields and equivalent enantioselectivity. These results seem to support the involvement of an active species involving two copper atoms, but the coordination environment of the copper dimer is apparently too crowded to realize the catalytic process. Mass spectral (MS) experiments were undertaken in an attempt to identify the real active copper species in the reaction solution. MS studies of the solution of crystal copper complex **2 [(*R,R*)-L2]**_**2**_**Cu**_**2**_**(OTf)**_**2**_ led to the observation of the monomer copper species **[*LL*Cu**^**I**^**]**^+^ and [***L*Cu**^I^]^+^ (Fig. 5d, top, (*R,R*)-**L2** was simplified as ***L***). These results suggest that the crystal dimer copper species [***LL*Cu**_**2**_**OTf**_**2**_] in solution, tend to dissociate into a monomeric copper species with one or two ligands. Upon addition of LPO, the **[*LL*Cu**^**I**^**]**^+^ disappeared and the **[*L*Cu**^**II**^**OCOC**_**11**_**H**_**23**_**]**^**+**^ appeared along with an increase of **[*LL*Cu**^**II**^**OTf]**^**+**^ (Fig. 5d, bottom). Kinetic experiments on the reaction showed first-order dependence of the rate on the copper catalyst. Further kinetic studies disclosed that the reaction with a lower concentration has a higher initial rate (same amount of catalyst and substrates loading in different volumes of solvent, see details in kinetic studies section of Supplementary Information). These kinetic experiments and MS studies suggest that the active copper species are more likely to be monomeric^50–52^.

Based on these preliminary results obtained, a possible reaction pathway is proposed (Fig. 5e). Copper (I) complex (**A**) catalyzes the decomposition of an alkyl diacyl peroxide forming an alkyl radical and a copper (II) species (**B**). The addition of the alkyl radical to a diene affords an allylic radical (**C**) which can react with copper (II) species (**B**) to deliver the chiral product and regenerate the copper catalyst (I) (**A**). Due to the complexity of copper chemistry, it is unclear whether the reaction involves a copper (III) species or proceeds through a ligand transfer pathway^3,5,6,45,53–57^.

### Synthetic applications

Enantioenriched allylic esters are important intermediates in organic synthesis. Inspired by the crossover experiments, externally added carboxylic acids rather than in-situ generated carboxylic acid groups, also show high priority towards esterification in the reported cases (Fig. 6a). This exceptional feature can greatly broaden the application of this reaction in the synthesis of enantioenriched allylic esters. As exemplified in Fig. 6b, the allylic esters (**96** and **97**) can be stemmed from (-)-menthol and lithocholic acid, respectively. Chiral allylic alcohols are useful building blocks for the construction of valuable organic compounds. The allylic esters that are obtained can be easily converted into chiral allylic alcohols (**98** and **104**) in high yields. The “magic methyl” effect is frequently invoked to explain the dramatic increase of biological activity of molecules by the introduction of one or more methyl groups. The allylic ester (**63**) can smoothly react with a methyl Grignard reagent to afford a chiral methylated allylic compound (**99**) under mild reaction conditions. Similarly, when treated with ethyl Grignard reagent and phenyl Grignard reagent, the corresponding ethylated (**100**) and phenylated (**101**) products can be generated. In the epoxidation reaction of the allylic ester (**63**), an epoxide (**102**) was obtained in 75% yield with 96:4 er for both of the diastereoisomers. In addition, the product (**63**) can be transformed into a chiral asymmetric α-alkylcarbonyloxy ketone (**103**) in almost quantitative yield under oxidative conditions with RuCl_3_/NaIO_4_. The C–C double bond was selectively reduced with the chiral C–O bond unchanged (**105**). A compound with a ten-membered ring (**106**) was successfully synthesized using a Grubbs II catalyst under the typical olefin metathesis conditions. Notably, enantioselectivity is retained in all these reactions.

We have developed a useful copper-catalyzed radical regioselective asymmetric carboesterification of dienes which takes place under mild reaction conditions. The carbon and oxygen functionalities in the product both originate from the alkyl diacyl peroxide reagents that are readily prepared from commercially available aliphatic carboxylic acids. The reaction can be further extended to use external carboxylic acids as the oxygen functionalities. A broad range of chiral structurally diversified allylic esters have been synthesized, and the products can be further transformed into various useful chiral synthons. This work describes a key advance in the elementary reaction that enantioselective construct C–O bond on the open-chain hydrocarbon radicals in intermolecular fashion and this work may inspire the discovery of other asymmetric radical transformations.

## Methods

### General method for the synthesis of compounds 3–44

In a flame-dried Schlenk tube, Cu(OTf)·0.5PhMe (0.005 mmol, 2.5 mol%) and ligand **L1** (0.007 mmol, 3.5 mol%) were dissolved in CH_3_CN (1.0 mL, 0.2 M) under a nitrogen atmosphere, and the mixture was stirred at room temperature for 30 mins. Then, diene (0.2 mmol, 1.0 equiv) and peroxide (0.4 mmol, 2.0 equiv) were sequentially added. The reaction mixture was stirred at room temperature for 3 days. After reaction completion, the solvent was evaporated under reduced pressure. The residue was purified by flash column chromatography on silica gel to afford the product.

### General method for the synthesis of compounds 45–79

In a flame-dried Schlenk tube, Cu(OTf)·0.5PhMe (0.01 mmol, 5 mol%) and ligand **L2** (0.014 mmol, 7 mol%) were dissolved in DCE (0.4 mL, 0.5 M) under a nitrogen atmosphere, and the mixture was stirred at room temperature for 30 mins. Then, diene (0.2 mmol, 1.0 equiv) and peroxide (0.24 mmol, 1.2 equiv) were sequentially added. The reaction mixture was stirred at 35°C for 3 days. After reaction completion, the solvent was evaporated under reduced pressure. The residue was purified by flash column chromatography on silica gel to afford the product.

### General method for the synthesis of compounds 82–93, 96, and 97

In a flame-dried Schlenk tube, Cu(OTf)·0.5PhMe (0.01 mmol, 5 mol%) and ligand **L2** (0.014 mmol, 5 mol%) were dissolved in DCE (0.4 mL, 0.5 M) under a nitrogen atmosphere, and the mixture was stirred at room temperature for 30 mins. Then, diene (0.2 mmol, 1.0 equiv), peroxide (0.24 mmol, 1.2 equiv) and acid (0.22 mmol, 1.1 equiv) were sequentially added. The reaction mixture was stirred at 50 °C for 3 days. After reaction completion, the solvent was evaporated under reduced pressure. The residue was purified by flash column chromatography on silica gel to afford the product.

## Supplementary information

Supporting Information

## Data Availability

Data relating to the characterization data of materials and products, general methods, optimization studies, experimental procedures, mechanistic studies, mass spectra, and HPLC and NMR spectra are available in Supplementary Information. Crystallographic data for compounds **3**, **9**, **65**, Copper Complex 1 [(*S*)-**L1**]_2_(Cu)_2_(OTf)_2_, Copper Complex 2 [(*R,R)-***L2**]_2_(Cu)_2_(OTf)_2_, **85**, **89** are available free of charge from the Cambridge Crystallographic Data Centre under reference numbers 2094205, 2094206, 2101770, 2094208, 2094209, 2094207, and 2094210, respectively.
