# Supplementary material for: Asymmetric radical carboesterification of dienes
Source: Nat Commun. 2021 Nov 18;12:6670. doi: 10.1038/s41467-021-26843-2 (PMC8602303; doi:10.1038/s41467-021-26843-2)
Supplement: Supplementary file 1 — Supporting Information [file 41467_2021_26843_MOESM1_ESM.pdf]

Supporting Information

Asymmetric Radical Carboesterification of Dienes

Xiaotao Zhu,[1,4] Wujun Jian,[1] Meirong Huang,[2] Daliang Li,[3] Yajun Li,[1] Xinhao Zhang[2] and Hongli Bao[1,4*]

Affiliations

[1] State Key Laboratory of Structural Chemistry, Key Laboratory of Coal to Ethylene Glycol and Its Related Technology, Center for Excellence in Molecular Synthesis, Fujian Institute of Research on the Structure of Matter, Chinese Academy of Sciences, 155 Yangqiao Road West, Fuzhou, Fujian 350002, P. R. of China.

[2] Shenzhen Bay Laboratory, State Key Laboratory of Chemical Oncogenomics, Peking University Shenzhen Graduate School, Shenzhen 518055, P. R. of China.

[3] Biomedical Research Center of South China & College of Chemistry and Materials Science, Fujian Normal University, Fuzhou 350007, P.R. of China

[4] University of Chinese Academy of Sciences, Beijing 100049, P. R. of China

*Correspondence to: hlbao@fjirsm.ac.cn

Table of contents

I. Supplementary Methods 3
A. Materials and methods 3
B. Synthesis of diacyl peroxides 4
C. Synthesis of conjugated diene materials 4
D. Characterization data for dienes 5
E. Optimization of the reaction conditions 14
1. Reaction condition optimization for diene S1. 14
2. Reaction condition optimization for diene S2 17
3. Probe of side product. 18
F. General procedure for asymmetric radical carboesterification of dienes 19
G. Gram-Scale Reaction 19
F. Characterization data for allyl esters 20
H. Mechanistic studies 57
1. Radical clock experiment 57
2. Radical trapping experiments 57
3. Cross-over experiment 59
4. Reactions catalyzed by single crystals of dimer copper complexes 60
5. MS studies 60
6. Kinetic Studies 63
I. Synthetic applications 69
J. Single crystal data 84
I. NMR spectra 92
L. HPLC traces 238
II. Supplementary References 342

I. Supplementary Methods

A. Materials and methods

All reactions were carried out under an atmosphere of nitrogen in glassware with magnetic stirring unless otherwise indicated. Commercially obtained reagents were used as received. Solvents were dried by Inert PureSolv MD5. Liquids and solutions were transferred via syringe. All reactions were monitored by thin-layer chromatography. ^{1}H and ^{13}C NMR spectra were recorded on Bruker-BioSpin AVANCE III HD and JEOL ECZ600S. Data for ^{1}H NMR spectra are reported relative to $CDCl_3$ as an internal standard (7.26 ppm) and are reported as follows: chemical shift (ppm), multiplicity, coupling constant (Hz), and integration. Data for ^{13}C NMR spectra are reported relative to $CDCl_3$ as an internal standard (77.0 ppm) and are reported in terms of chemical shift (ppm). GC-MS data were recorded on Thermo ISQ QD. HRMS data were recorded on Bruker Impact II UHR-TOF, Waters Micromass GCT Premier, or Thermo Fisher Scientific LTQ FT Ultra. IR data were obtained from Bruker VERTEX 70. GC-MS data were recorded on Thermo ISQ QD. High performance liquid chromatography (HPLC) analysis was performed on chiral columns. Optical rotations were measured using a 1 mL cell with a 5 dm path length on an INESA SGW-1 polarimeter. Melting points were measured on a Melt-Temp apparatus and were uncorrected.

B. Synthesis of diacyl peroxides

Diacyl peroxides were synthesized according to the literature.[1]

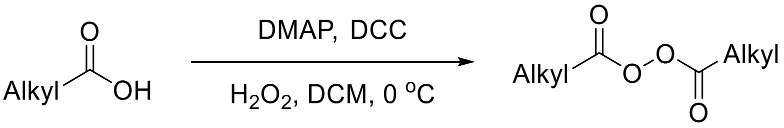

A solution of DMAP (0.6 mmol), 30% hydrogen peroxide (8 mmol), and acid (6 mmol) in DCM (8 mL) was cooled to -15 °C for about 10 min, then DCC (6.72 mmol) was added. After stirring for 1.5 h at -15 ~ -10 °C, DCM (15 mL) was added into the reaction solution and the solution was filtered through a short pad of silica gel. Then washed the pad of silica gel by additional 40 mL of DCM. The combined solution was concentrated on a rotary evaporator under vacuum at 10 ~ 15 °C and then purified by flash column chromatography on silica gel to give the diacyl peroxide.

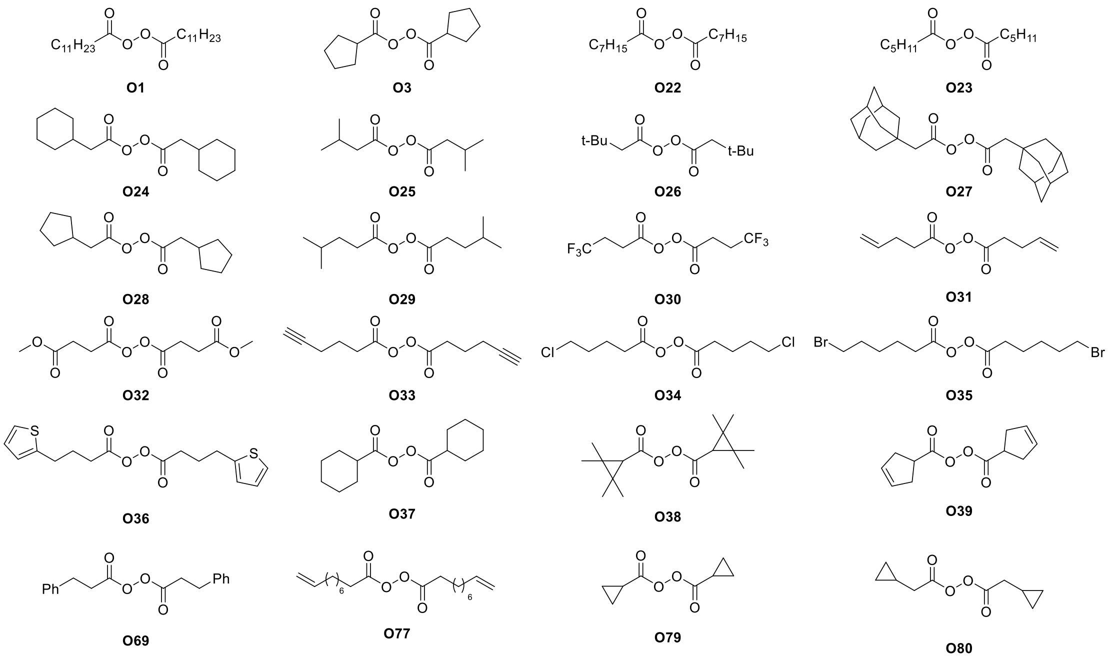

Supplementary Figure 1. Number of diacyl peroxide

C. Synthesis of conjugated diene materials

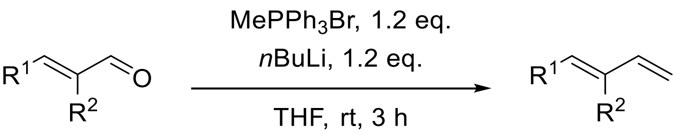

*n*BuLi (2.4 mL, 6 mmol, 2.5 M in hexane) was added dropwise to a stirred suspension of methyltriphenylphosphonium bromide (2.14 g, 6 mmol) in dry THF (25 mL) at 0 °C under a nitrogen atmosphere. After stirring for 30 mins at 0 °C, a THF (5 mL)

solution of cinnamaldehyde (5 mmol) was added dropwise. The reaction was kept stirring for 3 h with TLC detection. Then, the mixture was poured into saturated aq. NH_4Cl and extracted with EA for 3 times. The combined organic extracts were dried over anhydrous Na_2SO_4, concentrated, and purified by flash column chromatography on silica gel to afford conjugated dienes.

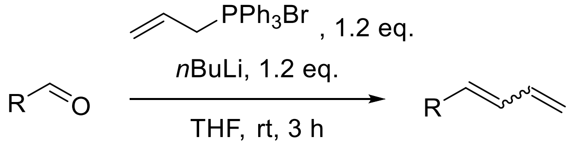

*n*BuLi (2.4 mL, 6 mmol, 2.5 M in hexane) was added dropwise to a stirred suspension of allyltriphenylphosphonium bromide (2.15 g, 6 mmol) in dry THF (25 mL) at 0 °C under a nitrogen atmosphere. After stirring for 30 mins at 0 °C, a THF (5 mL) solution of cinnamaldehyde (5 mmol) was added dropwise. The reaction was kept stirring for 3 h with TLC detection. Then, the mixture was poured into saturated aq. NH_4Cl and extracted with EA for 3 times. The combined organic extracts were dried over anhydrous Na_2SO_4, concentrated, and purified by flash column chromatography on silica gel to afford conjugated dienes.

D. Characterization data for dienes

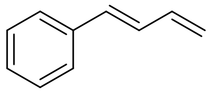

S1

^{1}H NMR (400 MHz, Chloroform-*d*) δ 7.38 (d, *J* = 7.5 Hz, 2H), 7.29 (t, *J* = 7.6 Hz, 2H), 7.21 (t, *J* = 7.6 Hz, 1H), 6.77 (dd, *J* = 15.6, 10.5 Hz, 1H), 6.59 – 6.43 (m, 2H), 5.32 (d, *J* = 16.9 Hz, 1H), 5.16 (d, *J* = 10.0 Hz, 1H). ^{13}C NMR (101 MHz, Chloroform-*d*) δ 137.24, 137.17, 132.92, 129.67, 128.66, 127.68, 126.50, 117.67. [2]

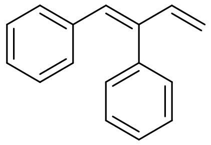

S2

^{1}H NMR (600 MHz, Chloroform-*d*) δ 7.42 – 7.37 (m, 2H), 7.36 – 7.31 (m, 1H), 7.20 – 7.14 (m, 2H), 7.12 – 7.04 (m, 3H), 6.92 – 6.86 (m, 2H), 6.74 (dd, *J* = 17.2, 10.5 Hz, 1H), 6.60 (s, 1H), 5.16 (dt, *J* = 10.4, 0.9 Hz, 1H), 4.84 (dd, *J* = 17.4, 0.8 Hz, 1H). ^{13}C NMR (151 MHz, Chloroform-*d*) δ 141.83, 137.93, 136.73, 131.57, 129.66, 129.50, 128.85, 128.03, 127.38, 126.99, 116.53, 77.32.[3]

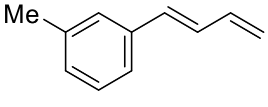

S4

^{1}H NMR (400 MHz, Chloroform-*d*) δ 7.21 – 7.13 (m, 3H), 7.06 – 6.96 (m, 1H), 6.75 (dd, *J* = 15.5, 10.6 Hz, 1H), 6.56 – 6.41 (m, 2H), 5.29 (d, *J* = 16.3 Hz, 1H), 5.13 (d, *J* = 9.3 Hz, 1H), 2.31 (s, 3H). ^{13}C NMR (101 MHz, Chloroform-*d*) δ 138.19, 137.39, 137.17, 133.12, 129.53, 128.61, 128.58, 127.28, 123.75, 117.49, 21.50.[2]

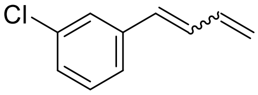

S5 (50:50)

^{1}H NMR (600 MHz, Chloroform-*d*) δ 7.44 – 7.36 (m, 0.5H), 7.32 – 7.29 (m, 0.5H), 7.28 – 7.14 (m, 3H), 6.88 – 6.74 (m, 1H), 6.55 – 6.44 (m, 1H), 6.38 (d, *J* = 11.5 Hz, 0.5H), 6.29 (t, *J* = 11.4 Hz, 0.5H), 5.43 – 5.39 (m, 0.5H), 5.39 – 5.35 (m, 0.5H), 5.29 – 5.26 (m, 0.5H), 5.24 – 5.21 (m, 0.5H). ^{13}C NMR (151 MHz, Chloroform-*d*) δ 139.19, 139.10, 136.84, 134.65, 134.22, 132.73, 132.00, 131.39, 131.04, 129.90, 129.57, 128.96, 128.91, 127.60, 127.24, 127.17, 126.32, 124.74, 120.81, 118.89.[4]

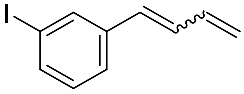

S6 (53:47)

^{1}H NMR (600 MHz, Chloroform-*d*) δ 7.88 – 7.67 (m, 1H), 7.67 – 7.52 (m, 1H), 7.39 – 7.24 (m, 1H), 7.12 – 7.01 (m, 1H), 6.91 – 6.74 (m, 1H), 6.57 – 6.28 (m, 2H), 5.45 (d, *J* = 16.8 Hz, 0.53H), 5.41 (d, *J* = 16.2 Hz, 0.47H), 5.32 (d, *J* = 10.2 Hz, 0.53H), 5.27 (d, *J* = 10.0 Hz, 0.47H). ^{13}C NMR (151 MHz, Chloroform-*d*) δ 139.63, 139.51, 137.87, 136.98, 136.54, 136.12, 135.39, 132.78, 132.08, 131.31, 131.02, 130.44, 130.10, 128.84, 128.37, 125.86, 121.03, 119.01, 95.16, 94.68.[5]

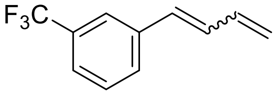

S7 (54:46)

^{1}H NMR (600 MHz, Chloroform-*d*) δ 7.68 – 7.62 (m, 1H), 7.59 – 7.40 (m, 3H), 6.95 – 6.77 (m, 1H), 6.61 – 6.34 (m, 2H), 5.50 – 5.46 (m, 0.46H), 5.46 – 5.42 (m, 0.54H), 5.36 – 5.32 (m, 0.46H), 5.31 – 5.28 (m, 0.54H). ^{13}C NMR (151 MHz, Chloroform-*d*) δ 138.17, 138.04, 136.78, 132.48, 132.38, 132.25, 131.44, 131.26, 131.10 (q, *J* = 51.7 Hz), 130.89 (q, *J* = 51.7 Hz), 129.55, 129.12, 128.78, 128.74, 125.70 (q, *J* = 3.9 Hz), 124.32 (q, *J* = 272.6 Hz), 124.12 (q, *J* = 3.1 Hz), 123.74 (q, *J* = 3.2 Hz), 123.08 (q, *J* = 3.1 Hz), 121.12, 119.11.[6]

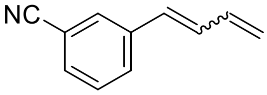

S8 (50:50)

^{1}H NMR (600 MHz, Chloroform-*d*) δ 7.64 – 7.27 (m, 4H), 6.83 – 6.68 (m, 1H), 6.52 – 6.43 (m, 1H), 6.41 – 6.30 (m, 1H), 5.46 – 5.37 (m, 1H), 5.32 – 5.23 (m, 1H). ^{13}C NMR (151 MHz, Chloroform-*d*) δ 138.54, 138.44, 136.48, 133.88, 133.75, 133.32, 132.95, 132.38, 132.12, 130.79, 130.52, 130.30, 129.84, 129.51, 129.21, 127.81, 121.86, 119.94, 118.85, 112.91, 112.55.[7]

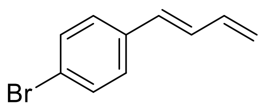

S9

^{1}H NMR (600 MHz, Chloroform-*d*) δ 7.43 (d, *J* = 8.5 Hz, 2H), 7.25 (d, *J* = 8.4 Hz, 2H), 6.76 (dd, *J* = 15.7, 10.5 Hz, 1H), 6.54 – 6.40 (m, 2H), 5.35 (d, *J* = 17.5 Hz, 1H), 5.21 (d, *J* = 10.8 Hz, 1H). ^{13}C NMR (151 MHz, Chloroform-*d*) δ 136.97, 136.13, 131.81, 131.62, 130.37, 127.99, 121.43, 118.50.[2]

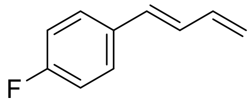

S10

^{1}H NMR (600 MHz, Chloroform-*d*) δ 7.38 – 7.34 (m, 2H), 7.03 – 6.99 (m, 2H), 6.70 (dd, *J* = 15.6, 10.5 Hz, 1H), 6.54 – 6.45 (m, 2H), 5.36 – 5.30 (m, 1H), 5.20 – 5.16 (m, 1H). ^{13}C NMR (151 MHz, Chloroform-*d*) δ 162.4 (d, *J* = 247.1 Hz), 137.1, 133.4 (d, *J* = 2.9 Hz), 131.7 (d, *J* = 1.4 Hz), 129.5 (d, *J* = 2.9 Hz), 128.0 (d, *J* = 7.9 Hz), 117.8 (d, *J* = 2.2 Hz), 115.7 (d, *J* = 21.7 Hz).[2]

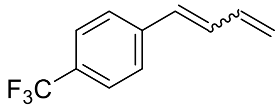

S11 (50:50)

^{1}H NMR (600 MHz, Chloroform-*d*) δ 7.59 (d, *J* = 8.2 Hz, 1H), 7.56 (d, *J* = 8.2 Hz, 1H), 7.48 (d, *J* = 8.1 Hz, 1H), 7.41 (d, *J* = 8.6 Hz, 1H), 6.90 – 6.77 (m, 1H), 6.61 – 6.43 (m, 1.5H), 6.35 (t, *J* = 11.4 Hz, 0.5H), 5.50 – 5.37 (m, 1H), 5.31 – 5.22 (m, 1H). ^{13}C NMR (151 MHz, Chloroform-*d*) δ 140.85, 140.54, 136.63, 132.56, 132.46, 131.93, 131.18, 129.13, 128.75, 126.46, 125.52 (q, *J* = 3.5 Hz), 125.14 (q, *J* = 3.5 Hz), 124.17 (q, *J* = 270.5 Hz), 121.14, 119.41.[7]

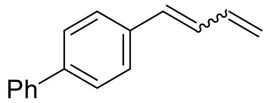

S12 (61:39)

^{1}H NMR (600 MHz, Chloroform-*d*) δ 7.72 – 7.57 (m, 4H), 7.54 – 7.42 (m, 4H), 7.41 – 7.34 (m, 1H), 7.05 – 6.96 (m, 0.61H), 6.91 – 6.84 (m, 0.39H), 6.71 – 6.48 (m, 1.39H), 6.37 – 6.30 (m, 0.61H), 5.45 (d, *J* = 16.8 Hz, 0.61H), 5.40 (d, *J* = 16.9 Hz, 0.39H), 5.30 (d, *J* = 10.1 Hz, 0.61H), 5.24 (d, *J* = 10.0 Hz, 0.39H). ^{13}C NMR (151 MHz, Chloroform-*d*) δ 140.82, 140.77, 140.45, 139.94, 137.36, 136.54, 136.29, 133.38, 132.55, 131.07, 130.08, 129.83, 129.63, 128.97, 127.49, 127.44, 127.14, 127.11, 127.08, 127.05, 127.03, 120.03, 117.90.[4]

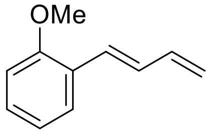

S13

^{1}H NMR (600 MHz, Chloroform-*d*) δ 7.51 (dd, *J* = 7.5, 1.6 Hz, 1H), 7.26 – 7.22 (m, 1H), 7.00 – 6.93 (m, 2H), 6.91 – 6.82 (m, 2H), 6.58 (dt, *J* = 17.4, 10.5 Hz, 1H), 5.39 – 5.30 (m, 1H), 5.22 – 5.13 (m, 1H),

3.87 (s, 3H). ^{13}C NMR (151 MHz, Chloroform-*d*) δ 156.91, 138.08, 130.33, 128.84, 127.81, 126.58, 126.19, 120.72, 117.10, 111.04, 55.56.[2]

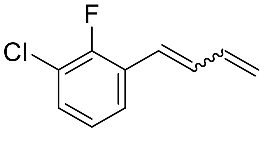

S14 (50:50)

^{1}H NMR (600 MHz, Chloroform-*d*) δ 7.40 – 7.34 (m, 0.5H), 7.33 – 7.27 (m, 0.5H), 7.26 – 7.21 (m, 1H), 7.07 – 6.99 (m, 1H), 6.89 – 6.81 (m, 0.5H), 6.73 – 6.63 (m, 1H), 6.57 – 6.48 (m, 0.5H), 6.46 – 6.34 (m, 1H), 5.46 – 5.36 (m, 1H), 5.31 – 5.23 (m, 1H). ^{13}C NMR (151 MHz, Chloroform-*d*) δ 155.66 (d, *J* = 252.3 Hz), 155.57 (d, *J* = 249.9 Hz), 137.08, 133.46, 133.16 (d, *J* = 5.3 Hz), 132.69, 129.44, 129.29, 129.23, 126.72 (d, *J* = 5.8 Hz), 126.63 (d, *J* = 8.7 Hz), 125.34 (d, *J* = 2.6 Hz), 124.47 (d, *J* = 4.6 Hz), 124.22 (d, *J* = 3.0 Hz), 124.10 (d, *J* = 4.7 Hz), 122.04 (d, *J* = 2.6 Hz), 121.65 (d, *J* = 17.8 Hz), 121.40 (d, *J* = 18.4 Hz), 119.55. HRMS (DART) *m/z* calcd for $[C_{10}H_9ClF]^+$ (M+H$^+$): 183.0371, found: 183.0371.

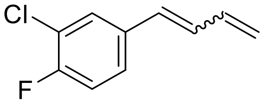

S15 (56:44)

^{1}H NMR (600 MHz, Chloroform-*d*) δ 7.42 – 7.40 (m, 0.44H), 7.33 (dd, *J* = 7.1, 2.1 Hz, 0.56H), 7.25 – 7.17 (m, 0.44H), 7.16 (ddd, *J* = 8.3, 4.6, 2.1 Hz, 0.56H), 7.08 (dt, *J* = 17.4, 8.7 Hz, 1H), 6.78 (dt, *J* = 16.8, 10.4 Hz, 0.56H), 6.74 – 6.63 (m, 0.44H), 6.52 – 6.23 (m, 2H), 5.42 (dd, *J* = 16.8, 1.7 Hz, 0.56H), 5.36 (d, *J* = 16.8 Hz, 0.44H), 5.29 (d, *J* = 10.1 Hz, 0.56H), 5.22 (d, *J* = 10.0 Hz, 0.44H). ^{13}C NMR (151 MHz, Chloroform-*d*) δ 157.49 (d, *J* = 249.8 Hz), 157.12 (d, *J* = 249.3 Hz), 136.70, 134.59 (d, *J* = 6.8 Hz), 132.41, 131.86, 130.96, 130.73, 130.32, 128.81 (d, *J* = 6.5 Hz), 128.23, 127.88, 126.16, 126.11, 121.33 (d, *J* = 18.4 Hz), 120.98, 120.86 (d, *J* = 17.7 Hz), 118.80, 116.76 (d, *J* = 21.5 Hz), 116.42 (d, *J* = 21.2 Hz). HRMS (DART) *m/z* calcd for $[C_{10}H_9ClF]^+$ (M+H$^+$): 183.0371, found: 183.0371.

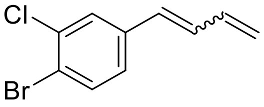

S16 (54:46)

^{1}H NMR (600 MHz, Chloroform-*d*) δ 7.55 (d, *J* = 8.3 Hz, 0.54H), 7.52 (d, *J* = 8.3 Hz, 0.46H), 7.45 (d, *J* = 2.1 Hz, 0.46H), 7.38 (d, *J* = 2.0 Hz, 0.54H), 7.11 (dd, *J* = 8.4, 2.1 Hz, 0.46H), 7.07 – 7.01 (m, 0.54H), 6.82 – 6.72 (m, 1H), 6.53 – 6.37 (m, 1H), 6.32 – 6.27 (m, 1H), 5.42 (dd, *J* = 16.8, 1.8 Hz, 0.54H), 5.38 (d, *J* = 16.9 Hz, 0.46H), 5.30 (dd, *J* = 10.0, 1.2 Hz, 0.54H), 5.24 (d, *J* = 10.0 Hz, 0.46H). ^{13}C NMR (151 MHz, Chloroform-*d*) δ 138.08, 138.03, 136.62, 134.77, 134.39, 133.83, 133.53, 132.47, 132.37, 130.61, 130.33, 128.52, 127.98, 127.81, 125.77, 121.43, 121.03, 120.83, 119.45. HRMS (EI) *m/z* calcd for $[C_{10}H_8BrCl]^+$ (M$^+$): 241.9492, found: 241.9495.

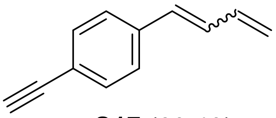

S17 (60:40)

^{1}H NMR (600 MHz, Chloroform-*d*) δ 7.49 – 7.42 (m, 2H), 7.35 (d, *J* = 8.2 Hz, 1H), 7.28 (d, *J* = 8.0 Hz, 1H), 6.89 – 6.76 (m, 1H), 6.56 – 6.46 (m, 0.8H), 6.42 (d, *J* = 11.5 Hz, 0.6H), 6.29 (t, *J* = 11.4 Hz, 0.6H), 5.45 – 5.33 (m, 1H), 5.24 (dd, *J* = 29.8, 10.2 Hz, 1H), 3.13 (s, 0.4H), 3.11 (s, 0.6H). ^{13}C NMR (151 MHz, Chloroform-*d*) δ 138.00, 137.69, 137.03, 132.96, 132.49, 132.10, 132.06, 131.74, 130.87, 129.59, 129.02, 126.38, 121.10, 121.69, 120.65, 118.72, 83.83, 83.75, 78.06, 77.81. HRMS (EI) *m/z* calcd for $[C_{12}H_{10}]^+$ (M^+): 154.0777, found: 154.0781.

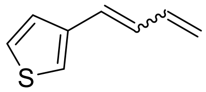

S18 (55:45)

^{1}H NMR (600 MHz, Chloroform-*d*) δ 7.35 – 7.27 (m, 0.55H), 7.29 (d, *J* = 6.9 Hz, 0.45H), 7.25 (d, *J* = 12.7 Hz, 1H), 7.19 (dd, *J* = 2.9, 1.2 Hz, 0.45H), 7.16 (dd, *J* = 5.0, 1.1 Hz, 0.55H), 7.04 – 6.93 (m, 0.55H), 6.72 – 6.55 (m, 1H), 6.49 (d, *J* = 36.8 Hz, 0.45H), 6.40 (d, *J* = 11.5 Hz, 0.55H), 6.22 (t, *J* = 11.4 Hz, 0.55H), 5.45 – 5.35 (m, 0.55H), 5.33 (d, *J* = 16.7 Hz, 0.45H), 5.26 (d, *J* = 9.9 Hz, 0.55H), 5.17 (d, *J* = 10.0 Hz, 0.45H). ^{13}C NMR (151 MHz, Chloroform-*d*) δ 140.01, 138.60, 137.27, 133.49, 130.06, 129.78, 128.67, 127.08, 126.25, 125.49, 125.02, 124.38, 123.87, 122.51, 119.57, 117.34.[8]

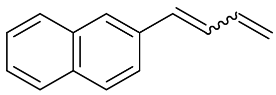

S19 (55:45)

^{1}H NMR (600 MHz, Chloroform-*d*) δ 8.01 – 7.80 (m, 4H), 7.70 (d, *J* = 10.1 Hz, 0.45H), 7.62 – 7.46 (m, 2.55H), 7.17 – 7.05 (m, 0.55H), 7.05 – 6.95 (m, 0.45H), 6.80 (d, *J* = 15.6 Hz, 0.45H), 6.73 – 6.61 (m, 1H), 6.45 (t, *J* = 11.4 Hz, 0.55H), 5.51 (dd, *J* = 30.0, 17.3 Hz, 1H), 5.34 (dd, *J* = 35.6, 10.1 Hz, 1H). ^{13}C NMR (151 MHz, Chloroform-*d*) δ 137.49, 135.11, 134.82, 133.86, 133.51, 133.25, 133.23, 132.63, 131.35, 130.65, 130.18, 128.46, 128.22, 128.15, 127.98, 127.91, 127.85, 127.39, 126.82, 126.52, 126.42, 126.18, 126.12, 123.65, 120.19, 118.02.[2]

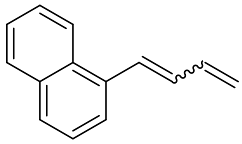

S20 (60:40)

^{1}H NMR (600 MHz, Chloroform-*d*) δ 8.21 (d, *J* = 8.3 Hz, 0.4H), 8.06 (d, *J* = 9.6 Hz, 0.6H), 7.95 – 7.88 (m, 1H), 7.87 – 7.79 (m, 1H), 7.73 (d, *J* = 7.2 Hz, 0.4H), 7.62 – 7.47 (m, 3.6H), 7.42 (d, *J* = 15.4 Hz, 0.4H), 7.03 (d, *J* = 11.4 Hz, 0.6H), 6.93 (dd, *J* = 15.4, 10.6 Hz, 0.4H), 6.78 – 6.69 (m, 1H), 6.59 (t, *J* = 11.2 Hz, 0.6H), 5.47 (d, *J* = 16.3 Hz, 1H), 5.31 (d, *J* = 10.0 Hz, 0.4H), 5.24 (d, *J* = 10.1 Hz, 0.6H). ^{13}C NMR (151 MHz, Chloroform-*d*) δ 137.62, 134.66, 134.49, 133.89, 133.82, 133.72, 132.65, 132.63, 131.98, 131.34, 129.80, 128.91, 128.79, 128.57, 128.23, 127.90, 127.42, 126.25, 126.20, 126.05, 125.97, 125.79, 125.41, 125.16, 123.79, 123.58, 119.50, 118.17.[2]

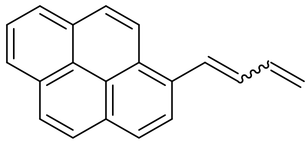

S21 (67:33)

^{1}H NMR (600 MHz, Chloroform-*d*) δ 8.36 (d, *J* = 9.2 Hz, 0.33H), 8.25 – 8.11 (m, 4H), 8.09 – 7.96 (m, 4.67H), 7.65 (d, *J* = 15.4 Hz, 0.33H), 7.30 – 7.24 (m, 0.67H), 7.09 (dd, *J* = 15.4, 10.5 Hz, 0.33H), 6.85 – 6.73 (m, 1H), 6.67 (t, *J* = 11.2 Hz, 0.67H), 5.53 (d, *J* = 16.8 Hz, 1H), 5.37 (d, *J* = 10.0 Hz, 0.33H), 5.28 (d, *J* = 10.0 Hz, 0.67H). ^{13}C NMR (151 MHz, Chloroform-*d*) δ 137.85, 133.90, 132.75, 132.58, 132.08, 131.62, 131.53, 131.48, 131.09, 131.02, 130.97, 130.77, 129.86, 129.30, 129.04, 128.32, 127.77, 127.66, 127.59, 127.56, 127.52, 127.46, 127.40, 126.09, 125.41, 125.34, 125.26, 125.18, 125.15, 124.92, 124.89, 124.54, 124.49, 123.50, 122.95, 122.95, 119.96, 118.27. HRMS (DART) *m/z* calcd for $[C_{20}H_{15}]^+$ $(M+H^+)$: 255.1168, found: 255.1167.

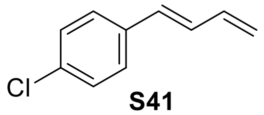

S41

^{1}H NMR (600 MHz, Chloroform-*d*) δ 7.34 – 7.31 (m, 2H), 7.29 – 7.26 (m, 2H), 6.75 (dd, *J* = 15.6, 10.6 Hz, 1H), 6.55 – 6.41 (m, 2H), 5.35 (d, *J* = 16.8 Hz, 1H), 5.20 (d, *J* = 10.0 Hz, 1H). ^{13}C NMR (151 MHz, Chloroform-*d*) δ 136.98, 135.69, 133.25, 131.55, 130.27, 128.77, 127.67, 118.35.[4]

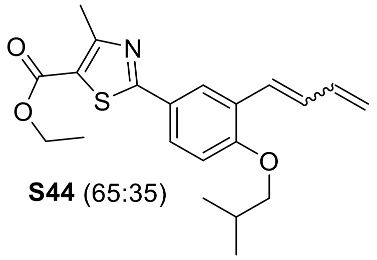

S44 (65:35)

^{1}H NMR (600 MHz, Chloroform-*d*) δ 8.07 – 8.02 (m, 0.35H), 7.87 – 7.82 (m, 1.3H), 7.80 – 7.72 (m, 0.35H), 6.97 – 6.76 (m, 2.35H), 6.60 – 6.49 (m, 1H), 6.33 (t, *J* = 11.4 Hz, 0.65H), 5.41 – 5.33 (m, 1H), 5.24 (d, *J* = 10.1 Hz, 0.65H), 5.18 (d, *J* = 10.1 Hz, 0.35H), 4.32 (q, *J* = 7.2 Hz, 2H), 3.79 (d, *J* = 6.4 Hz, 0.7H), 3.76 (d, *J* = 6.4 Hz, 1.3H), 2.76 (s, 1H), 2.75 (s, 2H), 2.20 – 2.06 (m, 1H), 1.39 – 1.34 (m, 3H), 1.06 (d, *J* = 6.7 Hz, 2.1H), 1.02 (d, *J* = 6.8 Hz, 3.9H). ^{13}C NMR (151 MHz, Chloroform-*d*) δ 169.98, 162.50, 162.47, 161.14, 161.08, 159.12, 158.66, 137.76, 133.25, 131.46, 131.43, 129.15, 127.35, 127.33, 127.02, 126.88, 126.69, 125.59, 125.27, 125.11, 125.07, 125.06, 120.86, 119.95, 117.96, 111.98, 111.60, 80.78, 74.94, 74.82, 61.20, 28.37, 19.42, 19.35, 17.65, 14.45. HRMS (DART) *m/z* calcd for $[C_{22}H_{26}O_3NS]^+$ $(M+H^+)$: 372.1628, found: 372.1623.

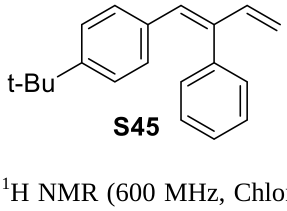

S45

^{1}H NMR (600 MHz, Chloroform-*d*) δ 7.44 – 7.38 (m, 2H), 7.37 – 7.34 (m, 1H), 7.20 – 7.17 (m, 2H), 7.11 (d, J = 8.6 Hz, 2H), 6.82 (d, J = 8.3 Hz, 2H), 6.74 (dd, J = 17.5, 10.8 Hz, 1H), 6.57 (s, 1H), 5.14 – 5.09 (m, 1H), 4.80 – 4.73 (m, 1H), 1.23 (s, 9H). ^{13}C NMR (151 MHz, Chloroform-*d*) δ 150.12, 142.18,

141.03, 138.27, 133.83, 131.49, 129.69, 129.27, 128.93, 125.15, 125.03, 115.93, 34.57, 31.24. HRMS (DART) m/z calcd for $[C_{20}H_{23}]^+$ $(M+H^+)$: 263.1794, found: 263.1792.

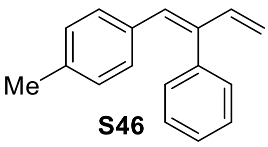

S46

^{1}H NMR (400 MHz, Chloroform-*d*) δ 7.44 – 7.29 (m, 3H), 7.21 – 7.12 (m, 2H), 6.90 (d, *J* = 8.3 Hz, 2H), 6.81 – 6.68 (m, 3H), 6.57 (s, 1H), 5.12 (d, *J* = 10.4 Hz, 1H), 4.80 (d, *J* = 17.1 Hz, 1H), 2.23 (s, 3H). ^{13}C NMR (101 MHz, Chloroform-*d*) δ 141.86, 140.93, 138.09, 136.81, 133.81, 131.48, 129.60, 129.34, 128.77, 128.73, 127.21, 115.90, 21.15.[2]

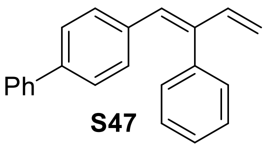

S47

^{1}H NMR (600 MHz, Chloroform-*d*) δ 7.54 – 7.49 (m, 2H), 7.45 – 7.32 (m, 8H), 7.23 – 7.19 (m, 2H), 6.98 – 6.94 (m, 2H), 6.80 – 6.72 (m, 1H), 6.64 (s, 1H), 5.20 – 5.14 (m, 1H), 4.88 – 4.81 (m, 1H). ^{13}C NMR (101 MHz, Chloroform-*d*) δ 141.92, 141.83, 140.54, 139.50, 137.98, 135.75, 131.13, 129.89, 129.62, 128.91, 128.75, 127.42, 127.29, 126.86, 126.64, 116.58. HRMS (DART) *m/z* calcd for $[C_{22}H_{19}]^+$ $(M+H^+)$: 283.1481, found: 283.1480.

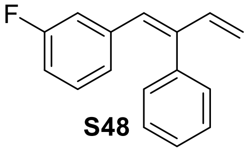

S48

^{1}H NMR (600 MHz, Chloroform-*d*) δ 7.46 – 7.37 (m, 3H), 7.22 – 7.16 (m, 2H), 7.08 (q, *J* = 6.9 Hz, 1H), 6.83 – 6.73 (m, 3H), 6.59 (s, 1H), 6.56 (dt, *J* = 10.9, 2.0 Hz, 1H), 5.24 (d, *J* = 10.5 Hz, 1H), 4.91 (d, *J* = 10.5 Hz, 1H). ^{13}C NMR (151 MHz, Chloroform-*d*) δ 162.52 (d, *J* = 244.2 Hz), 143.11, 141.52, 139.04 (d, *J* = 7.9 Hz), 137.47, 130.31 (d, *J* = 2.9 Hz), 129.52, 129.41 (d, *J* = 8.7 Hz), 129.05, 127.75, 125.47 (d, *J* = 2.9 Hz), 117.59, 115.83 (d, *J* = 22.4 Hz), 113.90 (d, *J* = 21.7 Hz). HRMS (DART) *m/z* calcd for $[C_{16}H_{14}F]^+$ $(M+H^+)$: 225.1074, found: 225.1073.

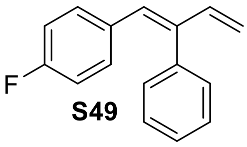

S49

^{1}H NMR (600 MHz, Chloroform-*d*) δ 7.53 – 7.43 (m, 3H), 7.32 – 7.27 (m, 2H), 7.02 – 6.95 (m, 2H), 6.92 – 6.81 (m, 3H), 6.68 (s, 1H), 5.29 (d, *J* = 10.5 Hz, 1H), 5.00 (d, *J* = 17.2 Hz, 1H). ^{13}C NMR (151 MHz, Chloroform-*d*) δ 161.7 (d, *J* = 248.0 Hz), 141.6, 141.6, 137.7, 132.9, 131.1 (d, *J* = 7.5 Hz), 130.3, 129.7, 129.0, 127.5, 116.6, 115.0 (d, *J* = 21.2 Hz). HRMS (DART) *m/z* calcd for $[C_{16}H_{14}F]^+$ $(M+H^+)$: 225.1074, found: 225.1073.

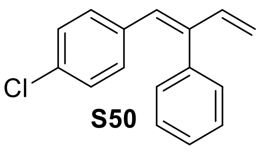

S50

^{1}H NMR (600 MHz, Chloroform-*d*) δ 7.43 – 7.33 (m, 3H), 7.18 – 7.14 (m, 2H), 7.06 (d, *J* = 7.6 Hz, 2H), 6.87 – 6.78 (m, 2H), 6.79 – 6.67 (m, 1H), 6.55 (s, 1H), 5.20 (d, *J* = 10.3 Hz, 1H), 4.88 (d, *J* = 17.2

Hz, 1H). ^{13}C NMR (151 MHz, Chloroform-*d*) δ 142.46, 141.51, 137.57, 135.25, 132.64, 130.66, 130.21, 129.58, 129.01, 128.25, 127.62, 117.17. HRMS (EI) *m/z* calcd for $[C_{16}H_{13}Cl]^+$ (M^+): 240.0700, found: 240.0705.

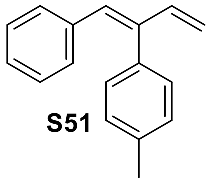

S51

^{1}H NMR (400 MHz, Chloroform-*d*) δ 7.19 (d, *J* = 7.8 Hz, 2H), 7.12 – 7.03 (m, 5H), 6.92 (dd, *J* = 7.3, 1.8 Hz, 2H), 6.72 (dd, *J* = 17.1, 10.4 Hz, 1H), 6.58 (s, 1H), 5.14 (d, *J* = 10.5 Hz, 1H), 4.86 (d, *J* = 17.1 Hz, 1H), 2.39 (s, 3H). ^{13}C NMR (101 MHz, Chloroform-*d*) δ 141.91, 141.75, 136.86, 136.81, 134.69, 131.36, 129.48, 129.46, 129.41, 127.93, 126.81, 116.29, 21.35. HRMS (DART) *m/z* calcd for $[C_{17}H_{17}]^+$ ($M+H^+$): 221.1325, found: 221.1324.

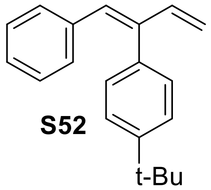

S52

^{1}H NMR (400 MHz, Chloroform-*d*) δ 7.44 – 7.34 (m, 2H), 7.12 – 6.99 (m, 5H), 6.95 – 6.85 (m, 2H), 6.73 (dd, *J* = 17.1, 10.4 Hz, 1H), 6.58 (s, 1H), 5.15 (d, *J* = 10.4 Hz, 1H), 4.89 (d, *J* = 17.1 Hz, 1H), 1.36 (s, 9H). ^{13}C NMR (101 MHz, Chloroform-*d*) δ 150.24, 141.87, 141.82, 136.86, 134.68, 131.39, 129.44, 129.14, 127.91, 126.79, 125.62, 116.41, 34.62, 31.47. HRMS (EI) *m/z* calcd for $[C_{20}H_{22}]^+$ (M^+): 262.1716, found: 262.1723.

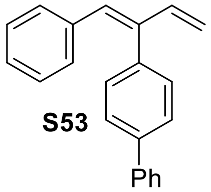

S53

^{1}H NMR (400 MHz, Chloroform-*d*) δ 7.72 – 7.51 (m, 4H), 7.46 (t, *J* = 7.0 Hz, 2H), 7.41 – 7.31 (m, 1H), 7.29 – 7.21 (m, 2H), 7.15 – 7.05 (m, 3H), 7.00 – 6.91 (m, 2H), 6.76 (dd, *J* = 17.1, 8.7 Hz, 1H), 6.63 (s, 1H), 5.19 (d, *J* = 10.4 Hz, 1H), 4.92 (d, *J* = 17.2 Hz, 1H). ^{13}C NMR (101 MHz, Chloroform-*d*) δ 141.70, 141.35, 140.74, 139.93, 136.90, 136.66, 131.65, 130.09, 129.47, 128.84, 128.03, 127.40, 127.36, 127.01, 116.51. HRMS (DART) *m/z* calcd for $[C_{22}H_{19}]^+$ ($M+H^+$): 283.1481, found: 283.1480.

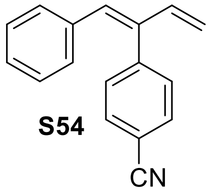

S54

^{1}H NMR (400 MHz, Chloroform-*d*) δ 7.61 (d, *J* = 8.2 Hz, 2H), 7.23 (d, *J* = 8.3 Hz, 2H), 7.09 – 6.99 (m, 3H), 6.83 – 6.74 (m, 2H), 6.71 – 6.58 (m, 2H), 5.12 (d, *J* = 10.5 Hz, 1H), 4.68 (d, *J* = 17.1 Hz, 1H). ^{13}C NMR (101 MHz, Chloroform-*d*) δ 142.25, 139.60, 138.75, 134.73, 131.54, 131.50, 129.71, 128.31, 127.16, 126.45, 117.84, 115.72, 110.24. MS (EI) *m/z* calcd for $[C_{17}H_{13}N]^+$ ($M+H^+$): 231.1, found: 231.2.

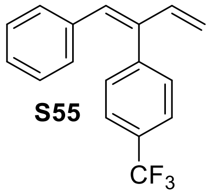

S55

^{1}H NMR (400 MHz, Chloroform-*d*) δ 7.64 (d, *J* = 7.6 Hz, 2H), 7.30 (d, *J* = 7.6 Hz, 2H), 7.16 – 7.03 (m, 3H), 6.91 – 6.84 (m, 2H), 6.73 (dd, *J* = 17.2, 10.5 Hz, 1H), 6.66 (s, 1H), 5.18 (d, *J* = 10.5 Hz, 1H), 4.77 (d, *J* = 17.2 Hz, 1H). ^{13}C NMR (101 MHz, Chloroform-*d*) δ 141.92, 141.11, 140.26, 136.06, 132.28, 130.19, 129.38, 128.15, 127.31, 126.32 (q, *J* = 270.4 Hz), 125.74 (q, *J* = 3.9 Hz), 116.67. HRMS (EI) *m/z* calcd for $[C_{17}H_{13}F_3]^+$ (M^+): 274.0964, found: 274.0961.

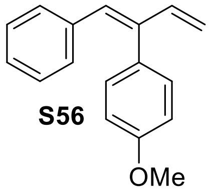

S56

^{1}H NMR (400 MHz, Chloroform-*d*) δ 7.18 – 7.00 (m, 5H), 6.97 – 6.86 (m, 4H), 6.72 (dd, *J* = 17.1, 10.4 Hz, 1H), 6.58 (s, 1H), 5.15 (d, *J* = 10.4 Hz, 1H), 4.88 (d, *J* = 17.1 Hz, 1H), 3.84 (s, 3H). ^{13}C NMR (101 MHz, Chloroform-*d*) δ 158.83, 142.01, 141.36, 136.86, 131.51, 130.73, 129.89, 129.42, 127.97, 126.82, 116.27, 114.22, 55.22. HRMS (DART) *m/z* calcd for $[C_{17}H_{17}O]^+$ ($M+H^+$): 237.1274, found: 237.1273.

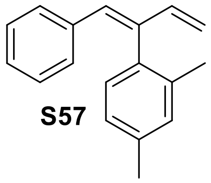
S57

^{1}H NMR (400 MHz, Chloroform-*d*) δ 7.20 – 7.06 (m, 5H), 7.02 – 6.92 (m, 3H), 6.77 (dd, *J* = 17.1, 10.3 Hz, 1H), 6.67 (s, 1H), 5.15 (d, *J* = 10.3 Hz, 1H), 4.75 (d, *J* = 17.1 Hz, 8H), 2.43 (s, 3H), 2.12 (s, 3H). ^{13}C NMR (101 MHz, Chloroform-*d*) δ 141.38, 141.20, 137.04, 137.00, 135.96, 134.29, 131.68, 131.18, 129.35, 128.97, 128.18, 127.12, 127.02, 115.89, 21.33, 19.18. HRMS (DART) *m/z* calcd for $[C_{18}H_{19}]^+$ ($M+H^+$): 235.1481, found: 235.1480.

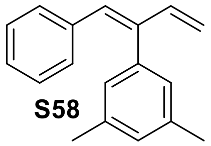
S58

^{1}H NMR (400 MHz, Chloroform-*d*) δ 7.22 – 7.12 (m, 3H), 7.09 – 6.99 (m, 3H), 6.92 – 6.87 (m, 2H), 6.81 (dd, *J* = 17.2, 10.5 Hz, 1H), 6.64 (s, 1H), 5.23 (d, *J* = 10.4 Hz, 1H), 4.95 (dd, *J* = 17.1, 1.3 Hz, 1H), 2.38 (s, 6H). ^{13}C NMR (101 MHz, Chloroform-*d*) δ 142.09, 138.26, 137.81, 136.86, 131.21, 129.52, 128.96, 128.02, 127.14, 126.91, 116.37, 21.42. HRMS (DART) *m/z* calcd for $[C_{18}H_{19}]^+$ ($M+H^+$): 235.1481, found: 235.1480.

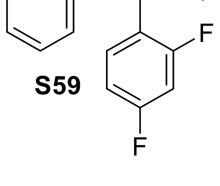

S59

^{1}H NMR (400 MHz, Chloroform-*d*) δ 7.18 – 7.04 (m, 4H), 6.98 – 6.83 (m, 4H), 6.79 – 6.67 (m, 2H), 5.16 (d, *J* = 10.6 Hz, 1H), 4.78 (d, *J* = 17.5 Hz, 1H). ^{13}C NMR (151 MHz, Chloroform-*d*) δ 162.73 (dd, *J* = 248.6, 11.4 Hz), 161.07 – 158.96 (m), 140.36, 136.28, 134.52, 133.87, 132.56 (dd, *J* = 8.9, 5.7 Hz), 128.97, 128.29, 127.53, 121.19 (d, *J* = 20.8 Hz), 115.93, 111.92 (d, *J* = 21.5 Hz), 104.58 (t, *J* = 25.5 Hz). HRMS (EI) *m/z* calcd for $[C_{16}H_{12}F_2]^+$ (M^+): 242.0902, found: 242.0905.

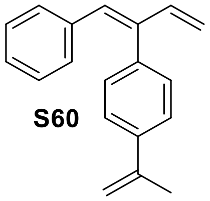

S60

^{1}H NMR (400 MHz, Chloroform-*d*) δ 7.50 (d, *J* = 8.2 Hz, 2H), 7.16 – 7.03 (m, 5H), 6.99 – 6.90 (m, 2H), 6.73 (dd, *J* = 17.2, 10.5 Hz, 1H), 6.60 (s, 1H), 5.46 (s, 1H), 5.20 – 5.07 (m, 2H), 4.87 (d, *J* = 17.1 Hz, 1H), 2.19 (s, 3H). ^{13}C NMR (101 MHz, Chloroform-*d*) δ 142.82, 141.70, 141.41, 139.82, 137.00, 136.67, 131.54, 129.50, 129.44, 128.01, 126.93, 125.81, 116.43, 112.31, 21.79. HRMS (EI) *m/z* calcd for $[C_{19}H_{18}]^+$ (M^+): 246.1404, found: 246.1410.

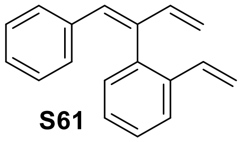

S61

^{1}H NMR (400 MHz, Chloroform-*d*) δ 7.44 (d, *J* = 6.9 Hz, 2H), 7.18 – 7.06 (m, 5H), 6.99 – 6.89 (m, 2H), 6.81 – 6.67 (m, 2H), 6.60 (s, 1H), 5.79 (d, *J* = 17.6 Hz, 1H), 5.28 (d, *J* = 10.9 Hz, 1H), 5.15 (d, *J* = 10.4 Hz, 1H), 4.86 (d, *J* = 17.2 Hz, 1H). ^{13}C NMR (101 MHz, Chloroform-*d*) δ 141.64, 141.37, 137.47, 136.67, 136.62, 136.50, 131.56, 129.85, 129.44, 128.02, 126.97, 126.67, 116.44, 113.78. HRMS (DART) *m/z* calcd for $[C_{18}H_{17}]^+$ ($M+H^+$): 233.1325, found: 233.1324.

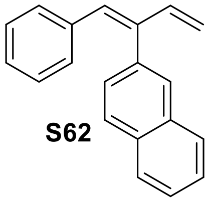

S62

^{1}H NMR (600 MHz, Chloroform-*d*) δ 7.91 – 7.86 (m, 2H), 7.80 (d, *J* = 8.4 Hz, 1H), 7.68 (s, 1H), 7.55 – 7.46 (m, 2H), 7.29 (d, *J* = 7.6 Hz, 1H), 7.07 – 7.00 (m, 3H), 6.95 – 6.90 (m, 2H), 6.85 – 6.76 (m, 1H), 6.70 (s, 1H), 5.18 (d, *J* = 10.3 Hz, 1H), 4.84 (d, *J* = 17.0 Hz, 1H). ^{13}C NMR (151 MHz, Chloroform-*d*) δ 141.82, 141.42, 136.51, 135.38, 133.66, 132.62, 131.83, 129.42, 128.44, 128.32, 128.01, 127.97, 127.76, 126.96, 125.94, 125.90, 116.59. HRMS (DART) *m/z* calcd for $[C_{20}H_{17}]^+$ ($M+H^+$): 257.1325, found: 257.1324.

E. Optimization of the reaction conditions

1. Reaction condition optimization for diene S1.

Supplementary Table 1. Evaluation of the chiral ligand

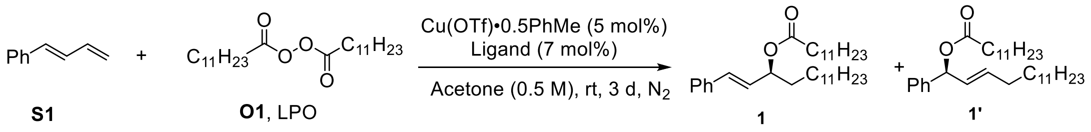

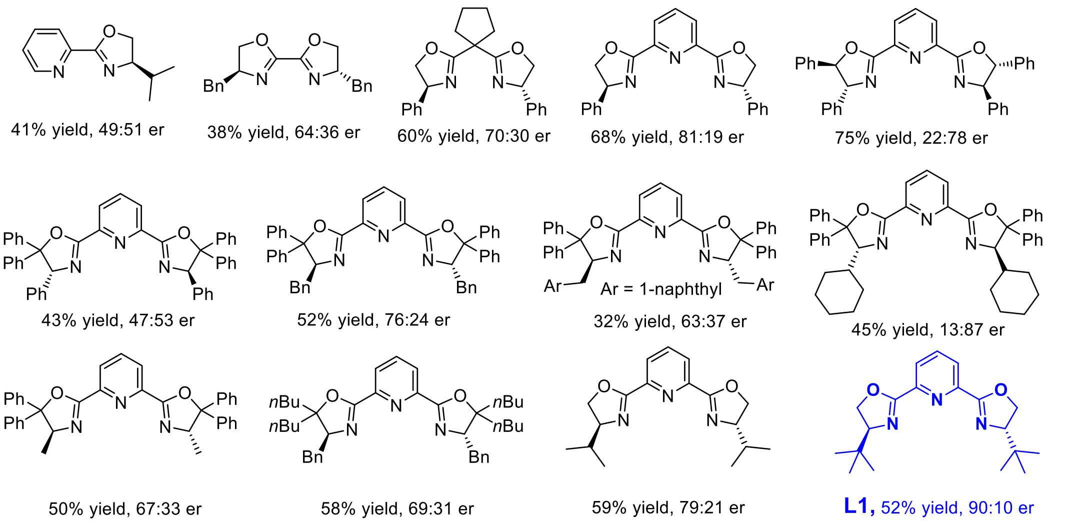

Reaction conditions: **S1** (0.20 mmol, 1 equiv), **O1** (0.3 mmol, 1.5 equiv), Cu(OTf)•0.5PhMe (5 mol%), chiral ligand (7 mol%) and acetone (0.4 mL, 0.5 M) at rt for 3 d.

Supplementary Table 2. Evaluation of the solvent

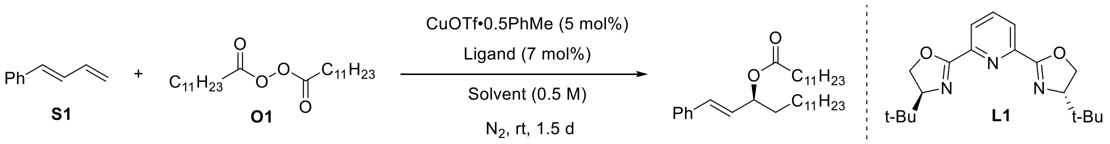

Entry	Solvent	Yield	Er
1	Acetone	63%	90:10
2	DCM	58%	80:20
3	DCE	55%	78:22
4	$CHCl_3$	61%	82:18
5	EA	41%	82:18
6	CH_3CN	49%	92:8
7	1,4-Dioxane	trace	-
8	Toluene	trace	-
9	DMF	26%	90:10
10	CH_3NO_2	15%	89:11

Reaction conditions: **S1** (0.20 mmol, 1 equiv), **O1** (0.4 mmol, 2 equiv), CuOTf•0.5PhMe (5 mol%), **L1** (7 mol%), and solvent (0.4 mL, 0.5 M) at rt for 1.5 d.

Supplementary Table 3. Evaluation of the catalyst

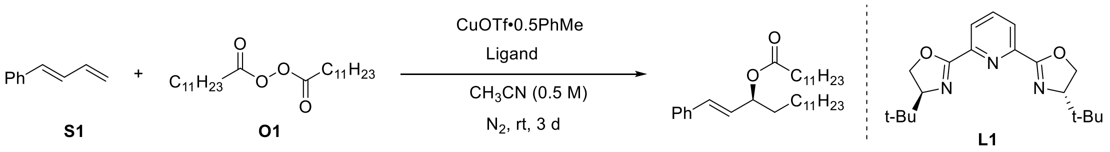

Entry	CuOTf•0.5PhMe	Ligand	Yield	Er
1	1.25 mol%	1.75 mol%	40%	93:7
2	2.5 mol%	3.5 mol%	72%	92:8
3	5 mol%	7 mol%	71%	92:8
4	10 mol%	14 mol%	68%	91:9

Reaction conditions: **S1** (0.20 mmol, 1 equiv), **O1** (0.4 mmol, 2 equiv), CuOTf•0.5PhMe (x mol%), **L1** (y mol%), and CH_3CN (0.4 mL, 0.5 M) at rt for 3 d.

Supplementary Table 4. Further reaction optimizations

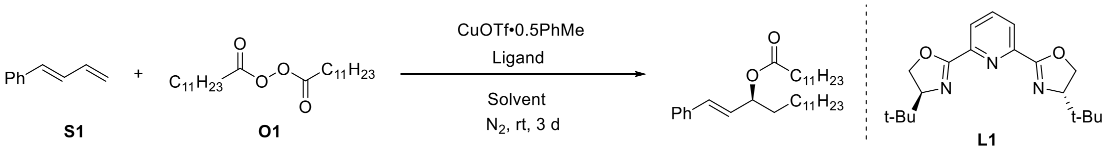

Entry	CuOTf•0.5PhMe	Ligand	Solvent	Yield	Er
1	5 mol%	7 mol%	0.2 mL CH_3CN	70%	90:10
2	5 mol%	7 mol%	0.4 mL CH_3CN	75%	91:9
3	5 mol%	7 mol%	1 mL CH_3CN	74%	91:9
4	5 mol%	7 mol%	2 mL CH_3CN	68%	92:8
5	5 mol%	7 mol%	0.2 mL $(CH_3)_2CHCN$	61%	92:8
6	5 mol%	7 mol%	0.2 mL DMA	59%	89:11
7	5 mol%	7 mol%	0.2 mL NMP	46%	87:13
8	2.5 mol%	3.5 mol%	1 mL CH_3CN	68%	93:7

Reaction conditions: **S1** (0.20 mmol, 1 equiv), **O1** (0.4 mmol, 2 equiv), CuOTf•0.5PhMe, **L1**, and solvent at rt for 3 d.

Supplementary Table 5. Other Cu catalyst optimizations

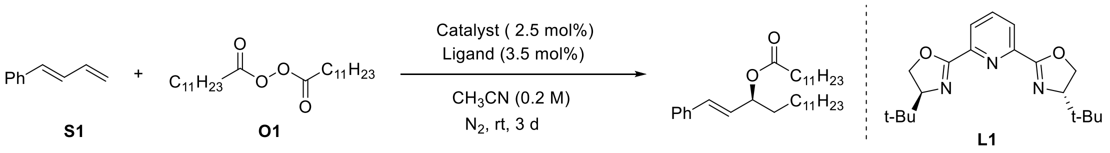

Entry	Cu Catalyst	Yield	Er
1	CuOTf	53%	92:8
2	$Cu(CH_3CN)_4PF_6$	65%	92:8
3	CuC_2O_4•0.5 H_2O	<10%	-
4	CuTc	51%	91:9
5	CuI	<10%	-
6	CuOAc	27%	91:9
7	CuBr	<10%	-
8	CuF_2	<10%	-
9	$Cu(acac)_2$	trace	-
10	$Cu(TFA)_2$	63%	92:8
11	$Cu(OAc)_2$	30%	88:12
12	CuSCN	66%	89:11
13	Cu_2O	<10%	-
14	CuBr•SMe_2	41%	84:16

Reaction conditions: **S1** (0.20 mmol, 1 equiv), **O1** (0.4 mmol, 2 equiv), Cu Catalyst (2.5 mol%), **L1** (7 mol%), and CH_3CN (1 mL, 0.5 M) at rt for 3 d.

2. Reaction condition optimization for diene S2

Supplementary Table 6. Evaluation of the chiral ligand for diene **S2**

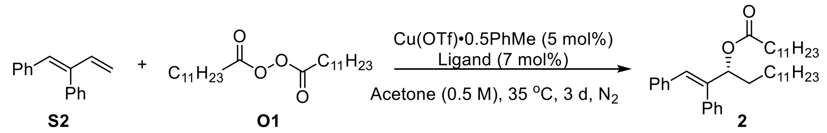

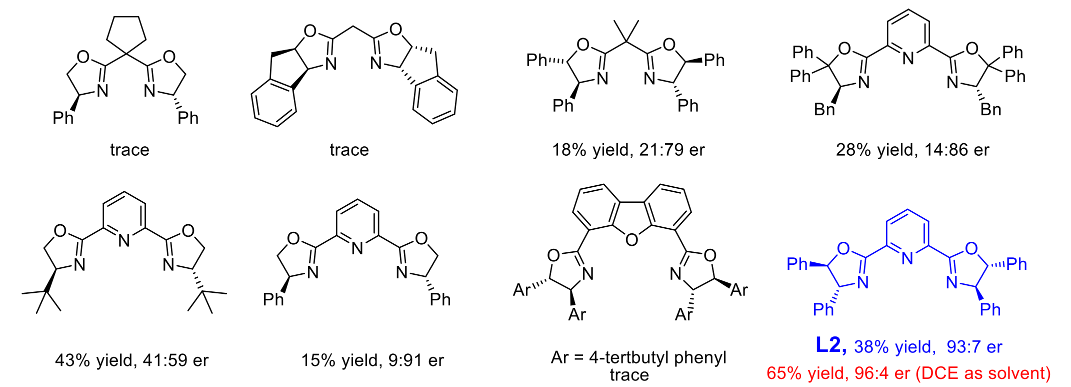

Reaction conditions: **S2** (0.20 mmol, 1 equiv), **O1** (0.24 mmol, 1.2 equiv), CuOTf•0.5PhMe (5 mol%), **L1** (7 mol%), and acetone (0.4 mL, 0.5 M) at 35 °C for 3 d.

3. Probe of side product.

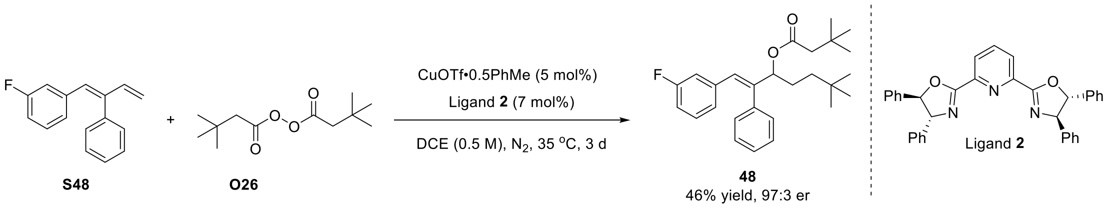

In a flame-dried Schlenk tube, Cu(OTf) 0.5PhMe (0.01 mmol, 5 mol%) and ligand **L2** (0.014 mmol, 7 mol%) were dissolved in DCE (0.4 mL, 0.5 M) under a nitrogen atmosphere, and the mixture was stirred at room temperature for 30 mins. Then, diene **S48** (44.8 mg, 0.2 mmol, 1.0 equiv) and peroxide **O26** (55.2 mg, 0.24 mmol, 1.2 equiv) were sequentially added. The reaction mixture was stirred at 35 °C for 3 days. After the reaction completion, the solvent was evaporated under reduced pressure. GC-MS analysis showed that the side products included 3,3-dimethylbutanoic acid, the remaining **S48**, Heck-type product and some unknown products.

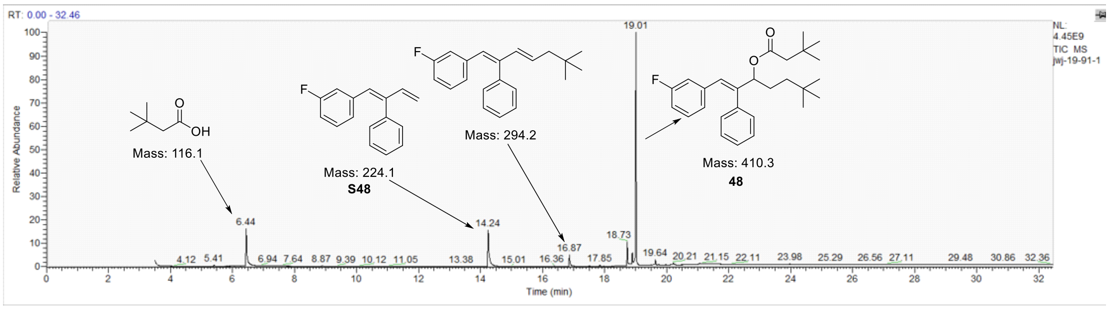

Supplementary Figure 2. Mass analysis of side product

F. General procedure for asymmetric radical carboesterification of dienes

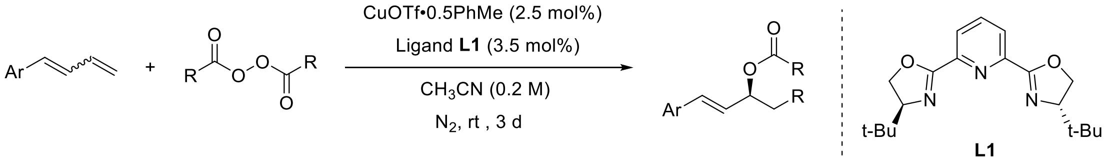

General procedure A: In a flame-dried Schlenk tube, Cu(OTf) 0.5PhMe (0.005 mmol, 2.5 mol%) and ligand **L1** (0.007 mmol, 3.5 mol%) were dissolved in CH_3CN (1.0 mL, 0.2 M) under a nitrogen atmosphere, and the mixture was stirred at room temperature for 30 mins. Then, diene (0.2 mmol, 1.0 equiv) and peroxide (0.4 mmol, 2.0 equiv) were sequentially added. The reaction mixture was stirred at room temperature for 3 days. After reaction completion, the solvent was evaporated under reduced pressure. The residue was purified by flash column chromatography on silica gel to afford the product.

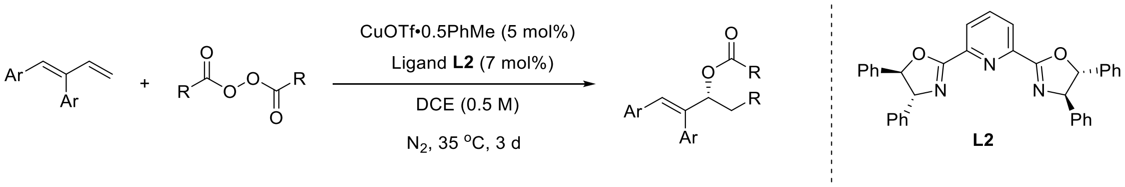

General procedure B: In a flame-dried Schlenk tube, Cu(OTf) 0.5PhMe (0.01 mmol, 5 mol%) and ligand **L2** (0.014 mmol, 7 mol%) were dissolved in DCE (0.4 mL, 0.5 M) under a nitrogen atmosphere, and the mixture was stirred at room temperature for 30 mins. Then, diene (0.2 mmol, 1.0 equiv) and peroxide (0.24 mmol, 1.2 equiv) were sequentially added. The reaction mixture was stirred at 35 ^{o}C for 3 days. After reaction completion, the solvent was evaporated under reduced pressure. The residue was purified by flash column chromatography on silica gel to afford the product.

G. Gram-Scale Reaction

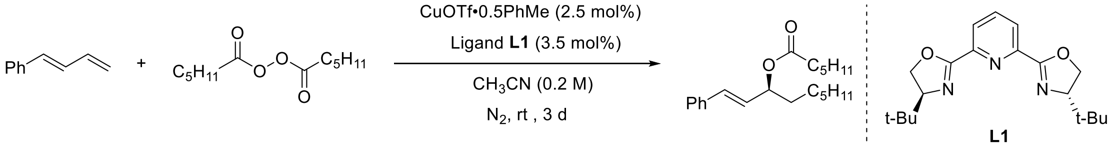

In a flame-dried Schlenk tube, Cu(OTf) 0.5PhMe (13.0 mg, 0.05 mmol, 2.5 mol%) and ligand **L2** (23.0 mg, 0.07 mmol, 3.5 mol%) were dissolved in CH_3CN (10.0 mL, 0.2 M) under a nitrogen atmosphere, and the mixture was stirred at room temperature for 30 mins. Then, **S1** (26.0 mg, 2.0 mmol, 1.0 equiv), peroxide **O23** (920.0 mg, 4.0 mmol, 2.0 equiv) were sequentially added. The reaction mixture was stirred at room temperature for 3 days. After reaction completion, the solvent was evaporated under

reduced pressure. The residue was purified by flash column chromatography on silica gel to afford the product **23** as a colorless oil (430.0 mg, 68% yield, 93:7 er).

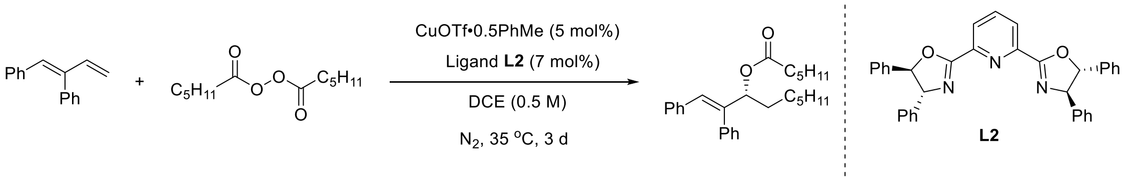

In a flame-dried Schlenk tube, Cu(OTf) 0.5PhMe (26.0 mg, 0.1 mmol, 5 mol%) and ligand **L2** (72.0 mg, 0.14 mmol, 7.0 mol%) were dissolved in DCE (4.0 mL, 0.5 M) under a nitrogen atmosphere, and the mixture was stirred at room temperature for 30 mins. Then, **S2** (412.0 mg, 2.0 mmol, 1.0 equiv) and peroxide **O23** (552.0 mg, 2.4 mmol, 1.2 equiv) were sequentially added. The reaction mixture was stirred at 35 °C for 3 days. After the reaction completion, the solvent was evaporated under reduced pressure. The residue was purified by flash column chromatography on silica gel to afford the product **63** as a colorless oil (588.0 mg, 75% yield, 96:4 er).

F. Characterization data for allyl esters

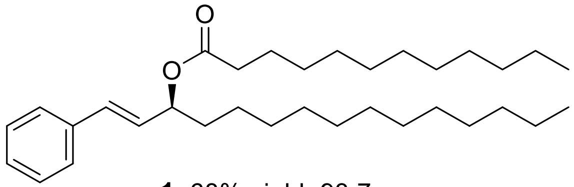

1, 68% yield, 93:7 er

According to General Procedure A, the reaction between diene **S1** (26.0 mg, 0.2 mmol, 1.0 equiv.) and peroxide **O1** (159.2 mg, 0.4 mmol, 2.0 equiv.) afforded product **1** as a white solid (65.8 mg, 68% yield, 93:7 er). $[\alpha]^{28.3}_{D}$ -90.88 (*c* 0.4, $CHCl_3$). Mp 45.4-46.3 °C. ^{1}H NMR (600 MHz, Chloroform-*d*) δ 7.38 – 7.35 (m, 2H), 7.33 – 7.28 (m, 2H), 7.23 (t, *J* = 7.3 Hz, 1H), 6.58 (d, *J* = 15.9 Hz, 1H), 6.11 (dd, *J* = 15.9, 7.3 Hz, 1H), 5.40 (q, *J* = 6.5 Hz, 1H), 2.41 (t, *J* = 7.5 Hz, 2H), 2.31 (t, *J* = 7.7 Hz, 2H), 1.70 (p, *J* = 7.6 Hz, 2H), 1.66 – 1.59 (m, 2H), 1.36 – 1.23 (m, 34H), 0.87 (t, *J* = 6.9 Hz, 6H). ^{13}C NMR (151 MHz, Chloroform-*d*) δ 173.35, 169.41, 136.53, 132.31, 128.63, 127.91, 126.63, 74.60, 34.81, 34.71, 32.02, 30.11, 29.78, 29.75, 29.72, 29.68, 29.66, 29.64, 29.60, 29.50, 29.45, 29.43, 29.41, 29.26, 29.20, 29.02, 25.31, 25.18, 24.92, 22.80, 14.24. HRMS (ESI) *m/z* calcd for $[C_{33}H_{56}NaO_2]^+$ ($[M+Na]^+$): 507.4173, found: 507.4171. IR (v/cm^{-1}) 3006, 2989, 2921, 2851, 1733, 1637, 1508, 1458, 1260, 750, 703. HPLC (OD-H, 0.46*25 cm, 2 µm, hexane/isopropanol = 99.5/0.5, flow 1.0 mL/min, detection at 254 nm) retention time = 5.386 min (major) and 7.699 min (minor).

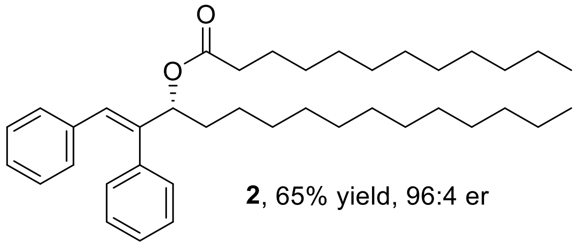

2, 65% yield, 96:4 er

According to General Procedure B, the reaction between diene **S2** (41.2 mg, 0.2 mmol, 1.0 equiv.) and peroxide **O1** (95.5 mg, 0.24 mmol, 1.2 equiv.) afforded product **2** was obtained as a white solid (72.8 mg, 65% yield, 96:4 er). $[\alpha]^{28.3}_{D}$ 37.88 (*c* 0.4, $CHCl_3$). Mp 65.3–67.2 ℃. ^1H NMR (600 MHz, Chloroform-*d*) δ 7.34 – 7.28 (m, 3H), 7.24 – 7.21 (m, 2H), 7.09 – 7.05 (m, 3H), 6.91 – 6.88 (m, 2H), 6.59 (s, 1H), 5.51 (t, *J* = 6.3 Hz, 1H), 2.35 (td, *J* = 7.4, 1.6 Hz, 2H), 1.66 – 1.57 (m, 4H), 1.30 – 1.20 (m, 36H), 0.87 (t, *J* = 7.1 Hz, 6H). ^{13}C NMR (151 MHz, Chloroform-*d*) δ 173.36, 141.68, 138.42, 136.41, 129.42, 129.37, 128.70, 127.94, 127.88, 127.53, 126.89, 78.43, 34.82, 33.39, 32.02, 29.77, 29.75, 29.72, 29.65, 29.59, 29.47, 29.44, 29.39, 29.29, 25.67, 25.17, 22.80, 14.24. HRMS (ESI) *m/z* calcd for $[C_{39}H_{60}NaO_2]^+$ ($[M+Na]^+$): 583.4486, found: 583.4487. IR (*v*/cm^{-1}) 3007, 2990, 2924, 2853, 1734, 1717, 1637, 1507, 1458, 1260, 750, 697. HPLC (OD-H, 0.46*25 cm, 2 μm, hexane/isopropanol = 99.5/0.5, flow 1.0 mL/min, detection at 254 nm) retention time = 5.015 min (major) and 5.985 min (minor).

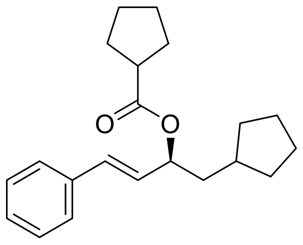

3, 63% yield, 93:7 er

According to General Procedure A, the reaction between diene **S1** (26.0 mg, 0.2 mmol, 1.0 equiv.) and peroxide **O3** (90.4 mg, 0.4 mmol, 2.0 equiv.) afforded product **3** was obtained as a white solid (39.3 mg, 63% yield, 93:7 er). $[\alpha]^{28.3}_{D}$ -66.038 (*c* 0.4, $CHCl_3$). Mp 63.8-65.0 °C. ^1H NMR (400 MHz, Chloroform-*d*) δ 7.38 (d, *J* = 7.0 Hz, 2H), 7.31 (t, *J* = 8.1 Hz, 2H), 7.26 – 7.21 (m, 1H), 6.60 (d, *J* = 16.0 Hz, 1H), 6.13 (dd, *J* = 16.0, 7.3 Hz, 1H), 5.43 (q, *J* = 6.6 Hz, 1H), 2.74 (p, *J* = 8.0 Hz, 1H), 1.93 – 1.78 (m, 7H), 1.73 – 1.48 (m, 10H), 1.20 – 1.06 (m, 2H). ^{13}C NMR (101 MHz, Chloroform-*d*) δ 176.05, 136.53, 132.05, 128.55, 128.31, 127.80, 126.56, 74.05, 44.15, 40.93, 36.42, 32.90, 32.72, 30.05, 29.94, 25.85, 25.81, 25.13, 25.05. HRMS (ESI) *m/z* calcd for $[C_{21}H_{28}NaO_2]^+$ ($[M+Na]^+$): 335.1982, found: 335.1982. IR (*v*/cm^{-1}) 3006, 2989, 2921, 2851, 1733, 1637, 1508, 1458, 1260, 750, 703. HPLC (OD-H, 0.46*25 cm, 2 μm, hexane/isopropanol = 100/0, flow 1.0 mL/min, detection at 254 nm) retention time = 17.587 min (major) and 18.945 min (minor).

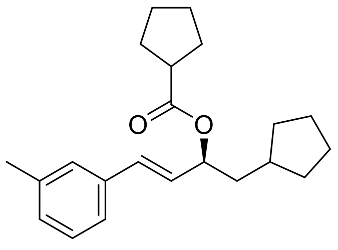

4, 45% yield, 93:7 er

According to General Procedure A, the reaction between diene **S4** (28.8 mg, 0.2 mmol, 1.0 equiv.) and peroxide **O3** (90.4 mg, 0.4 mmol, 2.0 equiv.) afforded product **4** was obtained as a colorless oil (29.3 mg, 45% yield, 93:7 er). $[\alpha]^{28.3}_{D}$ -61.035 (c 0.4, $CHCl_3$). ^1H NMR (400 MHz, Chloroform-*d*) δ 7.24 – 7.13 (m, 3H), 7.10 – 7.02 (m, 1H), 6.56 (d, *J* = 15.9 Hz, 1H), 6.12 (dd, *J* = 15.9, 7.3 Hz, 1H), 5.42 (q, *J* = 6.8 Hz, 1H), 2.74 (p, *J* = 8.0 Hz, 1H), 2.34 (s, 3H), 1.95 – 1.74 (m, 7H), 1.73 – 1.43 (m, 10H), 1.22 – 1.03 (m, 2H). ^{13}C NMR (101 MHz, Chloroform-*d*) δ 176.03, 138.11, 136.47, 132.17, 128.58, 128.44, 128.08, 127.25, 123.73, 74.11, 44.16, 40.93, 36.41, 32.91, 32.69, 30.04, 29.93, 25.84, 25.81, 25.13,

25.04, 21.38. HRMS (ESI) *m/z* calcd for $[C_{22}H_{30}NaO_2]^+$ ($[M+Na]^+$): 349.2138, found: 349.2140. IR (*v*/cm^{-1}) 3006, 2989, 2956, 2852, 1732, 1637, 1508, 1458, 1260, 750, 702. HPLC (IC, 0.46*25 cm, 2 µm, hexane/isopropanol = 99/1, flow 1.0 mL/min, detection at 254 nm) retention time = 4.740 min (minor) and 5.034 min (major).

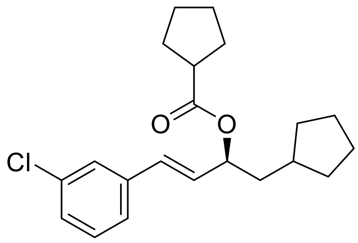

5, 68% yield, 95:5 er

According to General Procedure A, the reaction between diene **S5** (32.8 mg, 0.2 mmol, 1.0 equiv.) and peroxide **O3** (90.4 mg, 0.4 mmol, 2.0 equiv.) afforded product **5** was obtained as a colorless oil (47.1 mg, 68% yield, 95:5 er). $[\alpha]^{28.3}_{D}$ -89.42 (*c* 0.4, $CHCl_3$). ^1H NMR (400 MHz, Chloroform-*d*) δ 7.36 (s, 1H), 7.25 – 7.17 (m, 3H), 6.53 (dd, *J* = 15.9, 1.0 Hz, 1H), 6.15 (dd, *J* = 15.9, 7.1 Hz, 1H), 5.42 (q, *J* = 6.5 Hz, 1H), 2.80 – 2.67 (m, 1H), 1.96 – 1.76 (m, 7H), 1.73 – 1.46 (m, 10H), 1.21 – 1.06 (m, 2H). ^{13}C NMR (101 MHz, Chloroform-*d*) δ 176.02, 138.42, 134.51, 130.53, 129.91, 129.77, 127.71, 126.37, 124.87, 73.71, 44.12, 40.84, 36.39, 32.88, 32.74, 30.05, 29.95, 25.84, 25.81, 25.12, 25.04. HRMS (ESI) *m/z* calcd for $[C_{21}H_{27}ClNaO_2]^+$ ($[M+Na]^+$): 369.1592, found: 369.1592. IR (*v*/cm^{-1}) 3006, 2956, 2921, 2852, 1732, 1637, 1508, 1458, 1260, 750, 702. HPLC (OD-H, 0.46*25 cm, 2 µm, hexane/isopropanol = 99.5/0.5, flow 1.0 mL/min, detection at 254 nm) retention time = 6.171 min (major) and 6.513 min (minor).

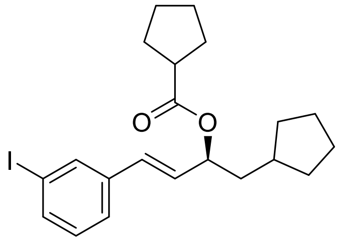

6, 72% yield, 94:6 er

According to General Procedure A, the reaction between diene **S6** (51.2 mg, 0.2 mmol, 1.0 equiv.) and peroxide **O3** (90.4 mg, 0.4 mmol, 2.0 equiv.) afforded product **6** was obtained as a colorless oil (63.1 mg, 72% yield, 94:6 er). $[\alpha]^{28.3}_{D}$ -75.43 (*c* 0.4, $CHCl_3$). ^1H NMR (400 MHz, Chloroform-*d*) δ 7.66 (s, 1H), 7.49 (d, *J* = 7.9 Hz, 1H), 7.24 (d, *J* = 7.9 Hz, 1H), 6.96 (t, *J* = 7.8 Hz, 1H), 6.41 (d, *J* = 15.9 Hz, 1H), 6.05 (dd, *J* = 16.0, 7.2 Hz, 1H), 5.34 (q, *J* = 6.7 Hz, 1H), 2.67 (p, *J* = 7.9 Hz, 1H), 1.88 – 1.68 (m, 7H), 1.66 – 1.34 (m, 10H), 1.15 – 0.98 (m, 2H). ^{13}C NMR (101 MHz, Chloroform-*d*) δ 176.00, 138.79, 136.58, 135.30, 130.33, 130.20, 129.85, 125.90, 94.62, 73.70, 44.12, 40.84, 36.39, 32.89, 32.73, 30.05, 29.94, 25.84, 25.81, 25.13, 25.04. HRMS (ESI) *m/z* calcd for $[C_{21}H_{27}INaO_2]^+$ ($[M+Na]^+$): 461.0948, found: 461.0949. IR (*v*/cm^{-1}) 3005, 2957, 2870, 1732, 1637, 1542, 1450, 1332, 1260, 750, 698. HPLC (OD-H, 0.46*25 cm, 2 µm, hexane/isopropanol = 99.8/0.2, flow 0.8 mL/min, detection at 254 nm) retention time = 13.409 min (major) and 14.387 min (minor).

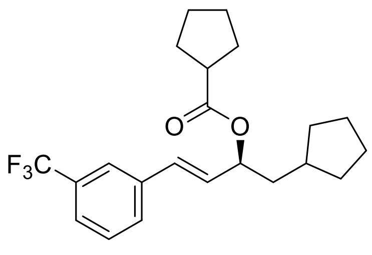

7, 81% yield, 89:11 er

According to General Procedure A, the reaction between diene **S7** (39.6 mg, 0.2 mmol, 1.0 equiv.) and peroxide **O3** (90.4 mg, 0.4 mmol, 2.0 equiv.) afforded product **7** was obtained as a colorless oil (61.2 mg, 81% yield, 89:11 er). $[\alpha]^{28.3}_{D}$ -90.13 (*c* 0.4, $CHCl_3$). ^1H NMR (400 MHz, Chloroform-*d*) δ 7.54 (s, 1H), 7.46 (d, *J* = 7.7 Hz, 1H), 7.41 (d, *J* = 7.9 Hz, 1H), 7.35 (t, *J* = 7.7 Hz, 1H), 6.54 (d, *J* = 16.0 Hz, 1H), 6.14 (dd, *J* = 16.0, 7.1 Hz, 1H), 5.37 (q, *J* = 6.8 Hz, 1H), 2.68 (p, *J* = 7.9 Hz, 1H), 1.88 – 1.71 (m, 7H), 1.66 – 1.36 (m, 10H), 1.15 – 0.99 (m, 2H). ^{13}C NMR (101 MHz, Chloroform-*d*) δ 176.03, 137.31, 130.98 (q, *J* = 32.3 Hz), 130.46, 130.38, 129.75 (d, *J* = 1.5 Hz), 129.00, 124.29 (q, *J* = 3.7 Hz), 124.08 (q, *J* = 270.0 Hz), 123.12 (q, *J* = 3.7 Hz), 73.66, 44.12, 40.84, 36.39, 32.86, 32.74, 30.07, 29.93, 25.83, 25.80, 25.12, 25.03. HRMS (ESI) *m/z* calcd for $[C_{22}H_{27}F_3NaO_2]^+$ ($[M+Na]^+$): 403.1855, found: 403.1856. IR (ν/cm^{-1}) 3007, 2956, 2852, 1719, 1637, 1508, 1458, 1260, 750, 702. HPLC (IC, 0.46*25 cm, 2 μm, hexane/isopropanol = 99/1, flow 1.0 mL/min, detection at 254 nm) retention time = 4.066 min (minor) and 4.249 min (major).

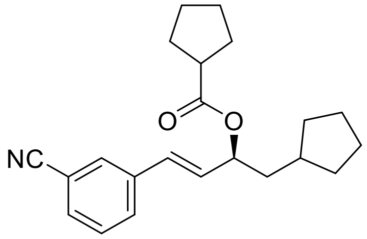

8, 65% yield, 94:6 er

According to General Procedure A, the reaction between diene **S8** (31.0 mg, 0.2 mmol, 1.0 equiv.) and peroxide **O3** (90.4 mg, 0.4 mmol, 2.0 equiv.) afforded product **8** was obtained as a colorless oil (43.8 mg, 65% yield, 94:6 er). $[\alpha]^{28.3}_{D}$ -79.45 (*c* 0.4, $CHCl_3$). ^1H NMR (600 MHz, Chloroform-*d*) δ 7.63 (s, 1H), 7.56 (d, *J* = 7.9 Hz, 1H), 7.50 (d, *J* = 7.7 Hz, 1H), 7.40 (t, *J* = 7.8 Hz, 1H), 6.58 – 6.51 (m, 1H), 6.19 (dd, *J* = 16.0, 7.0 Hz, 1H), 5.41 (q, *J* = 7.2 Hz, 1H), 2.79 – 2.70 (m, 1H), 1.93 – 1.85 (m, 2H), 1.82 – 1.77 (m, 5H), 1.70 – 1.55 (m, 8H), 1.53 – 1.47 (m, 2H), 1.18 – 1.08 (m, 2H). ^{13}C NMR (151 MHz, Chloroform-*d*) δ 176.14, 137.86, 131.36, 131.09, 130.87, 130.01, 129.59, 129.47, 118.80, 112.86, 73.55, 44.15, 40.85, 36.45, 32.92, 32.84, 30.14, 30.03, 25.91, 25.88, 25.18, 25.10. HRMS (ESI) *m/z* calcd for $[C_{22}H_{27}NNaO_2]^+$ ($[M+Na]^+$): 360.1934, found: 360.1931. IR (ν/cm^{-1}) 3005, 2952, 2868, 2231, 1731, 1637, 1508, 1458, 1260, 750, 689. HPLC (IC, 0.46*25 cm, 2 μm, hexane/isopropanol = 99/1, flow 1.0 mL/min, detection at 254 nm) retention time = 28.137 min (minor) and 33.066 min (major).

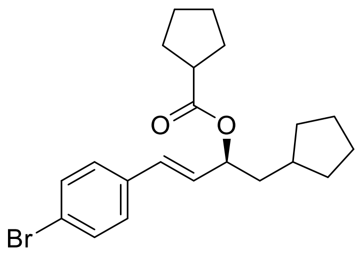

9, 64% yield, 94:6 er

According to General Procedure A, the reaction between diene **S9** (41.6 mg, 0.2 mmol, 1.0 equiv.) and peroxide **O3** (90.4 mg, 0.4 mmol, 2.0 equiv.) afforded product **9** was obtained as a colorless oil (49.9 mg, 64% yield, 94:6 er). [α] $^{28.3}_{D}$ -56.94 (*c* 0.4, $CHCl_3$). ^{1}H NMR (400 MHz, Chloroform-*d*) δ 7.43 (d, *J* = 8.4 Hz, 2H), 7.23 (d, *J* = 8.4 Hz, 2H), 6.52 (d, *J* = 16.0 Hz, 1H), 6.12 (dd, *J* = 15.9, 7.2 Hz, 1H), 5.41 (q, *J* = 6.8 Hz, 1H), 2.74 (p, *J* = 7.9 Hz, 1H), 1.95 – 1.76 (m, 7H), 1.73 – 1.42 (m, 10H), 1.21 – 1.07 (m, 2H). ^{13}C NMR (101 MHz, Chloroform-*d*) δ 176.04, 135.48, 131.64, 130.75, 129.17, 128.08, 121.57, 73.82, 44.13, 40.85, 36.40, 32.86, 32.75, 30.06, 29.93, 25.83, 25.80, 25.12, 25.04. HRMS (ESI) *m/z* calcd for $[C_{21}H_{27}BrNaO_2]^+$ ($[M+Na]^+$): 413.1087, found: 413.1085. IR (*v*/cm^{-1}) 2952, 2868, 1730, 1637, 1508, 1488, 1260, 750, 695. HPLC (OD-H, 0.46*25 cm, 2 µm, hexane/isopropanol = 99.5/0.5, flow 1.0 mL/min, detection at 254 nm) retention time = 5.672 min (minor) and 5.981 min (major).

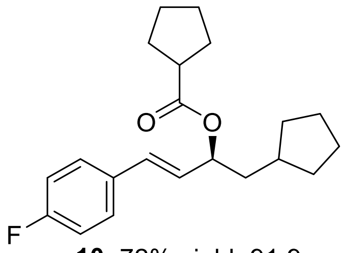

10, 72% yield, 91:9 er

According to General Procedure A, the reaction between diene **S10** (29.6 mg, 0.2 mmol, 1.0 equiv.) and peroxide **O3** (90.4 mg, 0.4 mmol, 2.0 equiv.) afforded product **10** was obtained as a white solid (47.5 mg, 72% yield, 91:9 er). [α] $^{28.3}_{D}$ -72.53 (*c* 0.4, $CHCl_3$). Mp 41.4-42.9 ^{o}C. ^{1}H NMR (400 MHz, Chloroform-*d*) δ 7.44 – 7.29 (m, 2H), 7.03 – 6.94 (m, 2H), 6.55 (d, *J* = 15.9 Hz, 1H), 6.04 (dd, *J* = 15.9, 7.3 Hz, 1H), 5.41 (q, *J* = 6.8, 6.2 Hz, 1H), 2.81 – 2.66 (m, 1H), 1.94 – 1.77 (m, 7H), 1.73 – 1.50 (m, 10H), 1.20 – 1.08 (m, 2H). ^{13}C NMR (101 MHz, Chloroform-*d*) δ 176.07, 162.43 (d, *J* = 247.2 Hz), 132.68 (d, *J* = 3.3 Hz), 130.90, 128.09 (d, *J* = 8.1 Hz), 128.08, 115.45 (d, *J* = 21.6 Hz), 73.98, 44.14, 40.92, 36.41, 32.88, 32.73, 30.06, 29.92, 25.84, 25.80, 25.12, 25.04. HRMS (ESI) *m/z* calcd for $[C_{21}H_{27}FNaO_2]^+$ ($[M+Na]^+$): 353.1887, found: 353.1886. IR (v/cm^{-1}) 3006, 2954, 2921, 2852, 1733, 1637, 1508, 1458, 1260, 750, 701. HPLC (OD-H, 0.46*25 cm, 2 µm, hexane/isopropanol = 99.5/0.5, flow 1.0 mL/min, detection at 254 nm) retention time = 5.129 min (major) and 5.797 min (minor).

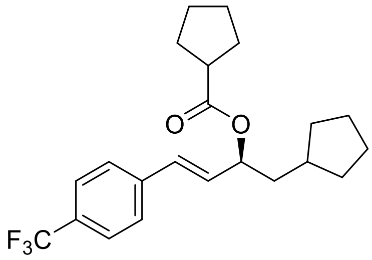

11, 60% yield, 95:5 er

According to General Procedure A, the reaction between diene **S11** (39.6 mg, 0.2 mmol, 1.0 equiv.) and peroxide **O3** (90.4 mg, 0.4 mmol, 2.0 equiv.) afforded product **11** was obtained as a colorless oil (45.6 mg, 60% yield, 95:5 er). $[\alpha]^{28.3}_{D}$ -82.39 (*c* 0.4, $CHCl_3$). ^{1}H NMR (600 MHz, Chloroform-*d*) δ 7.56 (d, *J* = 8.2 Hz, 2H), 7.47 (d, *J* = 8.2 Hz, 2H), 6.62 (d, *J* = 15.9 Hz, 1H), 6.23 (dd, *J* = 15.9, 7.1 Hz, 1H), 5.45 (q, *J* = 6.4 Hz, 1H), 2.76 (p, *J* = 8.0 Hz, 1H), 1.96 – 1.78 (m, 7H), 1.73 – 1.50 (m, 10H), 1.22 – 1.08 (m, 2H). ^{13}C NMR (151 MHz, Chloroform-*d*) δ 176.15, 140.09, 131.16, 130.50, 129.63 (d, *J* = 32.5 Hz), 126.78, 125.59 (q, *J* = 4.1 Hz), 124.22 (q, *J* = 271.6 Hz), 73.70, 44.17, 40.87, 36.47, 32.93, 32.84, 30.14, 30.02, 25.91, 25.88, 25.19, 25.11. HRMS (ESI) *m/z* calcd for $[C_{22}H_{27}F_3NaO_2]^+$ ($[M+Na]^+$): 403.1855, found: 403.1852. IR (*v*/cm^{-1}) 2954, 2870, 1731, 1637, 1508, 1458, 1260, 750, 700. HPLC (IC, 0.46*25 cm, 2 μm, hexane/isopropanol = 99/1, flow 1.0 mL/min, detection at 254 nm) retention time = 4.186 min (minor) and 4.457 min (major).

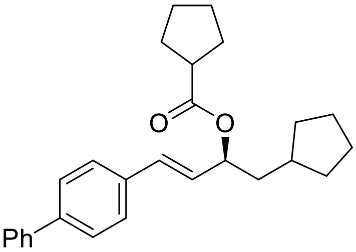

12, 66% yield, 93:7 er

According to General Procedure A, the reaction between diene **S12** (41.2 mg, 0.2 mmol, 1.0 equiv.) and peroxide **O3** (90.4 mg, 0.4 mmol, 2.0 equiv.) afforded product **12** was obtained as a white solid (51.2 mg, 66% yield, 93:7 er). $[\alpha]^{28.3}_{D}$ -65.94 (*c* 0.4, $CHCl_3$). Mp 49.5-51.5 °C. ^{1}H NMR (400 MHz, Chloroform-*d*) δ 7.63 – 7.52 (m, 4H), 7.49 – 7.40 (m, 4H), 7.37 – 7.30 (m, 1H), 6.64 (d, *J* = 15.9 Hz, 1H), 6.18 (dd, *J* = 15.9, 7.3 Hz, 1H), 5.46 (q, *J* = 6.8 Hz, 1H), 2.75 (p, *J* = 7.9 Hz, 1H), 1.94 – 1.78 (m, 7H), 1.73 – 1.46 (m, 10H), 1.22 – 1.06 (m, 2H). ^{13}C NMR (101 MHz, Chloroform-*d*) δ 176.07, 140.66, 140.59, 135.57, 131.63, 128.80, 128.41, 127.35, 127.25, 127.00, 126.95, 74.09, 44.17, 40.95, 36.44, 32.93, 32.74, 30.07, 29.96, 25.86, 25.83, 25.15, 25.06. HRMS (ESI) *m/z* calcd for $[C_{27}H_{32}NaO_2]^+$ ($[M+Na]^+$): 411.2295, found: 411.2295. IR (*v*/cm^{-1}) 3006, 2953, 2852, 1728, 1637, 1508, 1458, 1260, 750, 698. HPLC (IC, 0.46*25 cm, 2 μm, hexane/isopropanol = 99/1, flow 1.0 mL/min, detection at 254 nm) retention time = 7.106 min (minor) and 8.019 min (major).

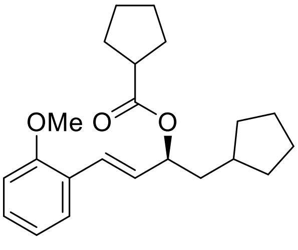

13, 55% yield, 89:11 er

According to General Procedure A, the reaction between diene **S13** (32.0 mg, 0.2 mmol, 1.0 equiv.) and peroxide **O3** (90.4 mg, 0.4 mmol, 2.0 equiv.) afforded product **13** was obtained as a colorless oil (37.6 mg, 55% yield, 89:11 er). $[\alpha]^{28.3}_{D}$ -90.04 (*c* 0.4, $CHCl_3$). ^{1}H NMR (400 MHz, Chloroform-*d*) δ 7.42 (dd, *J* = 7.6, 1.8 Hz, 1H), 7.25 – 7.19 (m, 1H), 6.99 – 6.81 (m, 3H), 6.17 (dd, *J* = 16.1, 7.3 Hz, 1H), 5.51 – 5.41 (m, 1H), 3.84 (s, 3H), 2.77 – 2.74 (m, 1H), 1.94 – 1.79 (m, 7H), 1.73 – 1.47 (m, 10H), 1.19 – 1.05 (m, 2H). ^{13}C NMR (101 MHz, Chloroform-*d*) δ 176.04, 156.85, 128.82, 128.75, 126.83, 126.65, 125.52, 120.54, 110.90, 74.51, 55.46, 44.19, 41.03, 36.46, 32.89, 32.74, 30.04, 29.93, 25.85, 25.82,

25.15, 25.06. HRMS (ESI) *m/z* calcd for $[C_{22}H_{30}NaO_3]^+$ ($[M+Na]^+$): 365.2087, found: 365.2086. IR (ν/cm^{-1}) 3006, 2954, 2851, 1719, 1637, 1508, 1458, 1260, 750, 703. HPLC (IC, 0.46*25 cm, 2 μm, hexane/isopropanol = 99/1, flow 1.0 mL/min, detection at 254 nm) retention time = 7.010 min (minor) and 7.539 min (major).

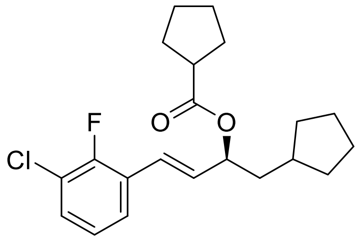

14, 61% yield, 95:5 er

According to General Procedure A, the reaction between diene **S14** (36.4 mg, 0.2 mmol, 1.0 equiv.) and peroxide **O3** (90.4 mg, 0.4 mmol, 2.0 equiv.) afforded product **14** was obtained as a colorless oil (44.4 mg, 61% yield, 95:5 er). $[\alpha]^{28.3}_{D}$ -68.24 (*c* 0.4, $CHCl_3$). ^{1}H NMR (400 MHz, Chloroform-*d*) δ 7.36 – 7.30 (m, 1H), 7.29 – 7.24 (m, 1H), 7.02 (td, *J* = 7.9, 1.3 Hz, 1H), 6.69 (d, *J* = 16.0 Hz, 1H), 6.27 (dd, *J* = 16.1, 6.7 Hz, 1H), 5.44 (q, *J* = 6.7, 6.0 Hz, 1H), 2.81 – 2.68 (m, 1H), 1.95 – 1.77 (m, 7H), 1.72 – 1.51 (m, 10H), 1.20 – 1.07 (m, 2H). ^{13}C NMR (101 MHz, Chloroform-*d*) δ 176.00, 155.64 (d, *J* = 252.0 Hz), 132.51 (d, *J* = 5.1 Hz), 129.40, 125.99 (d, *J* = 12.1 Hz), 125.82 (d, *J* = 3.3 Hz), 124.34 (d, *J* = 4.8 Hz), 123.21 (d, *J* = 3.3 Hz), 121.56 (d, *J* = 18.3 Hz), 73.69, 44.11, 40.82, 36.41, 32.86, 32.78, 30.01, 29.99, 25.83, 25.13, 25.04. HRMS (ESI) *m/z* calcd for $[C_{21}H_{26}ClFNaO_2]^+$ ($[M+Na]^+$): 387.1498, found: 387.1498. IR (ν/cm^{-1}) 3006, 2954, 2868, 1732, 1637, 1508, 1458, 1260, 750, 669. HPLC (IC, 0.46*25 cm, 2 μm, hexane/isopropanol = 99.5/0.5, flow 1.0 mL/min, detection at 254 nm) retention time = 4.918 min (minor) and 5.482 min (major).

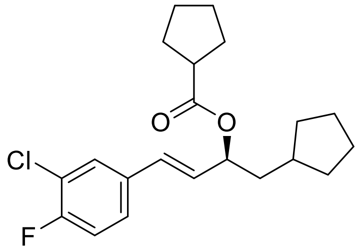

15, 72% yield, 94:6 er

According to General Procedure A, the reaction between diene **S15** (36.4 mg, 0.2 mmol, 1.0 equiv.) and peroxide **O3** (90.4 mg, 0.4 mmol, 2.0 equiv.) afforded product **15** was obtained as a colorless oil (52.4 mg, 72% yield, 94:6 er). $[\alpha]^{28.3}_{D}$ -77.38 (*c* 0.4, $CHCl_3$). ^{1}H NMR (400 MHz, Chloroform-*d*) δ 7.41 (dd, *J* = 7.0, 2.3 Hz, 1H), 7.25 – 7.17 (m, 1H), 7.07 (t, *J* = 8.7 Hz, 1H), 6.49 (d, *J* = 15.9 Hz, 1H), 6.07 (dd, *J* = 15.9, 7.1 Hz, 1H), 5.40 (q, *J* = 6.8, 6.4 Hz, 1H), 2.80 – 2.67 (m, 1H), 1.95 – 1.75 (m, 7H), 1.73 – 1.44 (m, 10H), 1.21 – 1.06 (m, 2H). ^{13}C NMR (101 MHz, Chloroform-*d*) δ 176.03, 157.56 (d, *J* = 249.8 Hz), 133.88 (d, *J* = 4.0 Hz), 129.61 (d, *J* = 2.9 Hz), 129.59 (d, *J* = 3.7 Hz), 128.34, 126.30 (d, *J* = 7.0 Hz), 121.17 (d, *J* = 18.3 Hz), 116.60 (d, *J* = 21.3 Hz), 73.66, 44.11, 40.84, 36.38, 32.86, 32.74, 30.05, 29.93, 25.83, 25.80, 25.11, 25.03. HRMS (ESI) *m/z* calcd for $[C_{21}H_{26}ClFNaO_2]^+$ ($[M+Na]^+$): 387.1498, found: 387.1497. IR (ν/cm^{-1}) 3006, 2989, 1698, 1652, 1507, 1457, 1260, 750, 669. HPLC (IC, 0.46*25 cm, 2 μm, hexane/isopropanol = 99/1, flow 1.0 mL/min, detection at 254 nm) retention time = 4.773 min (minor) and 5.150 min (major).

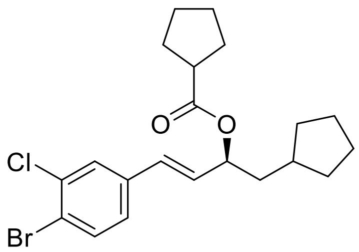

16, 74% yield, 95:5 er

According to General Procedure A, the reaction between diene **S16** (48.4 mg, 0.2 mmol, 1.0 equiv.) and peroxide **O3** (90.4 mg, 0.4 mmol, 2.0 equiv.) afforded product **16** was obtained as a colorless oil (62.8 mg, 74% yield, 95:5 er). $[\alpha]^{28.3}_{D}$ -99.48 (*c* 0.4, $CHCl_3$). ^1H NMR (400 MHz, Chloroform-*d*) δ 7.53 (d, *J* = 8.3 Hz, 1H), 7.45 (d, *J* = 2.2 Hz, 1H), 7.10 (dd, *J* = 8.3, 2.2 Hz, 1H), 6.48 (d, *J* = 15.9 Hz, 1H), 6.15 (dd, *J* = 15.9, 7.1 Hz, 1H), 5.40 (q, *J* = 6.8 Hz, 1H), 2.75 (p, *J* = 7.9 Hz, 1H), 1.96 – 1.77 (m, 7H), 1.73 – 1.43 (m, 10H), 1.22 – 1.06 (m, 2H). ^{13}C NMR (101 MHz, Chloroform-*d*) δ 176.01, 137.36, 134.65, 133.70, 130.59, 129.52, 128.08, 125.94, 121.24, 73.55, 44.10, 40.78, 36.38, 32.85, 32.76, 30.06, 29.94, 25.83, 25.80, 25.11, 25.03. HRMS (ESI) *m/z* calcd for $[C_{21}H_{26}BrClNaO_2]^+$ ($[M+Na]^+$): 447.0697, found: 447.0696. IR (*v*/cm^{-1}) 3007, 2990, 2868, 1718, 1637, 1508, 1458, 1260, 750, 704. HPLC (IC, 0.46*25 cm, 2 μm, hexane/isopropanol = 99/1, flow 1.0 mL/min, detection at 254 nm) retention time = 5.098 min (minor) and 5.671 min (major).

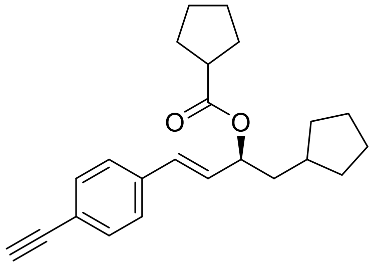

17, 55% yield, 93:7 er

According to General Procedure A, the reaction between diene **S17** (30.8 mg, 0.2 mmol, 1.0 equiv.) and peroxide **O3** (90.4 mg, 0.4 mmol, 2.0 equiv.) afforded product **17** was obtained as a colorless oil (37.0 mg, 55% yield, 93:7 er). $[\alpha]^{28.3}_{D}$ -83.04 (*c* 0.4, $CHCl_3$). ^1H NMR (600 MHz, Chloroform-*d*) δ 7.42 (d, *J* = 8.3 Hz, 2H), 7.31 (d, *J* = 8.2 Hz, 2H), 6.55 (d, *J* = 15.9 Hz, 1H), 6.14 (dd, *J* = 15.9, 7.2 Hz, 1H), 5.41 (q, *J* = 6.8, 6.3 Hz, 1H), 3.10 (s, 1H), 2.73 (p, *J* = 8.0 Hz, 1H), 1.92 – 1.75 (m, 7H), 1.70 – 1.49 (m, 10H), 1.18 – 1.07 (m, 2H). ^{13}C NMR (151 MHz, Chloroform-*d*) δ 176.16, 137.08, 132.40, 131.20, 129.72, 126.52, 121.34, 83.69, 77.92, 73.92, 44.19, 40.94, 36.47, 32.94, 32.81, 30.14, 30.01, 25.92, 25.89, 25.20, 25.12. HRMS (ESI) *m/z* calcd for $[C_{23}H_{28}NaO_2]^+$ ($[M+Na]^+$): 359.1982, found: 359.1983. IR (*v*/cm^{-1}) 2960, 2869, 2106, 1719, 1637, 1508, 1457, 1260, 750, 704. HPLC (IC, 0.46*25 cm, 2 μm, hexane/isopropanol = 99.5/0.5, flow 1.0 mL/min, detection at 254 nm) retention time = 7.570 min (minor) and 8.657 min (major).

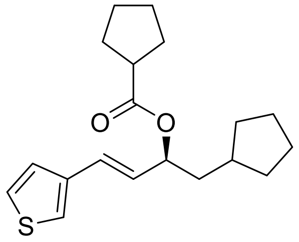

18, 45% yield, 87:13 er

According to General Procedure A, the reaction between diene **S18** (27.2 mg, 0.2 mmol, 1.0 equiv.) and peroxide **O3** (90.4 mg, 0.4 mmol, 2.0 equiv.) afforded product **18** was obtained as a colorless oil (28.6 mg, 45% yield, 87:13 er). $[\alpha]^{28.3}_{D}$ -54.74 (*c* 0.4, $CHCl_3$). ^1H NMR (400 MHz, Chloroform-*d*) δ 7.30 – 7.24 (m, 1H), 7.21 – 7.18 (m, 1H), 7.18 – 7.14 (m, 1H), 6.61 (d, *J* = 15.9 Hz, 1H), 5.98 (dd, *J* = 15.9, 7.4 Hz, 1H), 5.39 (q, *J* = 7.0 Hz, 1H), 2.73 (p, *J* = 7.9 Hz, 1H), 1.95 – 1.73 (m, 7H), 1.72 – 1.43 (m, 10H), 1.22 – 1.05 (m, 2H). ^{13}C NMR (101 MHz, Chloroform-*d*) δ 175.02, 138.16, 127.12, 125.37, 125.01, 123.94, 121.65, 73.04, 43.12, 39.87, 35.37, 31.85, 31.65, 29.02, 28.86, 24.81, 24.77, 24.09, 24.00. HRMS (ESI) *m/z* calcd for $[C_{19}H_{26}NaO_2S]^+$ ($[M+Na]^+$): 341.1546, found: 341.1545. IR (*v*/cm^{-1}) 1732, 1637, 1508, 1458, 1260, 750, 703. HPLC (IC, 0.46*25 cm, 2 μm, hexane/isopropanol = 99/1, flow 1.0 mL/min, detection at 254 nm) retention time = 5.452 min (minor) and 5.840 min (major).

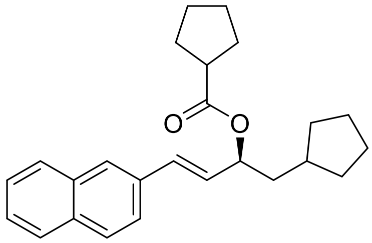

19, 79% yield, 93:7 er

According to General Procedure A, the reaction between diene **S19** (36.0 mg, 0.2 mmol, 1.0 equiv.) and peroxide **O3** (90.4 mg, 0.4 mmol, 2.0 equiv.) afforded product **19** was obtained as a white solid (57.2 mg, 79% yield, 93:7 er). $[\alpha]^{28.3}_{D}$ -103.54 (*c* 0.4, $CHCl_3$). Mp 54.0-55.2 ^oC. ^1H NMR (600 MHz, Chloroform-*d*) δ 7.81 – 7.75 (m, 3H), 7.73 (s, 1H), 7.58 (dd, *J* = 8.6, 1.7 Hz, 1H), 7.45 – 7.43 (m, 2H), 6.76 (d, *J* = 15.8 Hz, 1H), 6.26 (dd, *J* = 15.9, 7.3 Hz, 1H), 5.49 (q, *J* = 6.5 Hz, 1H), 2.76 (p, *J* = 8.0 Hz, 1H), 1.93 – 1.78 (m, 7H), 1.73 – 1.48 (m, 10H), 1.21 – 1.10 (m, 2H). ^{13}C NMR (151 MHz, Chloroform-*d*) δ 176.21, 134.06, 133.61, 133.16, 132.23, 128.77, 128.27, 128.09, 127.74, 126.79, 126.38, 126.05, 123.62, 74.22, 44.25, 41.05, 36.53, 33.00, 32.83, 30.17, 30.03, 25.95, 25.91, 25.23, 25.15. HRMS (ESI) *m/z* calcd for $[C_{25}H_{30}NaO_2]^+$ ($[M+Na]^+$): 385.2138, found: 385.2139. IR (*v*/cm^{-1}) 2991, 2953, 2867, 1720, 1637, 1508, 1458, 1260, 750, 704. HPLC (OD-H, 0.46*25 cm, 2 μm, hexane/isopropanol = 99.5/0.5, flow 1.0 mL/min, detection at 254 nm) retention time = 10.550 min (minor) and 13.386 min (major).

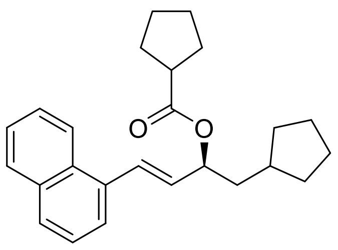

20, 52% yield, 91:9 er

According to General Procedure A, the reaction between diene **S20** (36.0 mg, 0.2 mmol, 1.0 equiv.) and peroxide **O3** (90.4 mg, 0.4 mmol, 2.0 equiv.) afforded product **20** was obtained as a white solid (37.6 mg, 52% yield, 91:9 er). $[\alpha]^{28.3}_{D}$ -77.39 (*c* 0.4, $CHCl_3$). Mp 54.0-55.2 ^{o}C. ^{1}H NMR (600 MHz, Chloroform-*d*) δ 8.07 (d, *J* = 8.5 Hz, 1H), 7.83 (dd, *J* = 7.6, 1.1 Hz, 1H), 7.77 (d, *J* = 8.2 Hz, 1H), 7.56 (d, *J* = 7.1 Hz, 1H), 7.53 – 7.45 (m, 2H), 7.45 – 7.40 (m, 1H), 7.34 (d, *J* = 15.6 Hz, 1H), 6.15 (dd, *J* = 15.6, 7.2 Hz, 1H), 5.55 (q, *J* = 6.8, 6.2 Hz, 1H), 2.77 (p, *J* = 8.1 Hz, 1H), 1.95 – 1.80 (m, 7H), 1.77 – 1.57 (m, 8H), 1.54 – 1.47 (m, 2H), 1.23 – 1.13 (m, 2H). ^{13}C NMR (151 MHz, Chloroform-*d*) δ 176.22, 134.48, 133.63, 131.65, 131.26, 129.40, 128.58, 128.21, 126.23, 125.91, 125.64, 123.97, 123.89, 74.24, 44.27, 41.02, 36.60, 33.03, 32.86, 30.12, 30.12, 25.95, 25.92, 25.25, 25.15. HRMS (ESI) *m/z* calcd for $[C_{25}H_{30}NaO_2]^+$ ($[M+Na]^+$): 385.2138, found: 385.2139. IR (*v*/cm^{-1}) 2952, 2968, 1726, 1646, 1508, 1451, 1261, 750, 704. HPLC (OD-H, 0.46*25 cm, 2 μm, hexane/isopropanol = 99.5/0.5, flow 1.0 mL/min, detection at 254 nm) retention time = 13.696 min (major) and 23.634 min (minor).

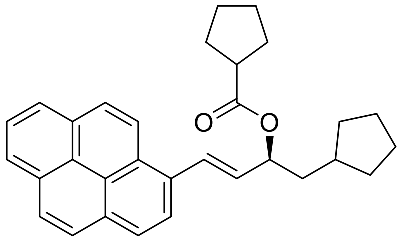

21, 35% yield, 83:17 er

According to General Procedure A, the reaction between diene **S21** (51.2 mg, 0.2 mmol, 1.0 equiv.) and peroxide **O3** (90.4 mg, 0.4 mmol, 2.0 equiv.) afforded product **21** was obtained as a colorless oil (30.5 mg, 35% yield, 83:17 er). $[\alpha]^{28.3}_{D}$ -43.42 (*c* 0.4, $CHCl_3$). ^{1}H NMR (600 MHz, Chloroform-*d*) δ 8.35 (d, *J* = 9.2 Hz, 1H), 8.17 (d, *J* = 7.9 Hz, 2H), 8.14 – 8.09 (m, 3H), 8.03 (d, *J* = 2.3 Hz, 2H), 7.99 (t, *J* = 7.6 Hz, 1H), 7.66 (d, *J* = 15.6 Hz, 1H), 6.35 (dd, *J* = 15.7, 7.2 Hz, 3H), 5.65 (q, *J* = 6.5 Hz, 1H), 2.82 (p, *J* = 8.0 Hz, 1H), 1.97 – 1.81 (m, 9H), 1.76 – 1.69 (m, 2H), 1.66 – 1.52 (m, 6H), 1.23 – 1.16 (m, 2H). ^{13}C NMR (151 MHz, Chloroform-*d*) δ 176.30, 131.79, 131.53, 131.29, 131.03, 130.97, 129.40, 128.36, 127.75, 127.50, 127.42, 126.08, 125.37, 125.17, 125.05, 124.97, 124.94, 124.07, 123.14, 74.48, 44.31, 41.14, 36.64, 33.05, 32.91, 30.18, 30.13, 25.97, 25.94, 25.27, 25.18. HRMS (ESI) *m/z* calcd for $[C_{31}H_{32}NaO_2]^+$ ($[M+Na]^+$): 459.2295, found: 459.2297. IR (*v*/cm^{-1}) 2968, 2939, 2879, 1717, 1638, 1458, 1261, 750. HPLC (AD-H, 0.46*25 cm, 2 μm, hexane/isopropanol = 99.5/0.5, flow 1.0 mL/min, detection at 254 nm) retention time = 15.071 min (minor) and 18.566 min (major).

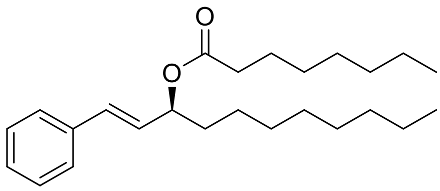

22, 73% yield, 92:8 er

According to General Procedure A, the reaction between diene **S1** (26.0 mg, 0.2 mmol, 1.0 equiv.) and peroxide **O22** (114.4 mg, 0.4 mmol, 2.0 equiv.) afforded product **22** was obtained as a colorless oil (54.3 mg, 73% yield, 92:8 er). $[\alpha]^{28.3}_{D}$ -67.55 (*c* 0.4, $CHCl_3$). ^1H NMR (400 MHz, Chloroform-*d*) δ 7.30 (d, *J* = 7.1 Hz, 2H), 7.24 (t, *J* = 7.5 Hz, 2H), 7.16 (d, *J* = 7.2 Hz, 1H), 6.52 (d, *J* = 15.9 Hz, 1H), 6.05 (dd, *J* = 16.0, 7.3 Hz, 1H), 5.34 (q, *J* = 6.7 Hz, 1H), 2.25 (t, *J* = 7.5 Hz, 2H), 1.70 – 1.48 (m, 4H), 1.31 – 1.14 (m, 20H), 0.93 – 0.65 (m, 6H). ^{13}C NMR (101 MHz, Chloroform-*d*) δ 173.24, 136.47, 132.24, 128.54, 128.03, 127.82, 126.55, 74.51, 34.73, 34.64, 31.87, 31.70, 29.50, 29.42, 29.25, 29.13, 28.98, 25.22, 25.10, 22.68, 22.62, 14.12, 14.08. HRMS (ESI) *m/z* calcd for $[C_{25}H_{40}NaO_2]^+$ ($[M+Na]^+$): 395.2921, found: 396.2922. IR (ν/cm^{-1}) 2956, 2928, 2856, 1734, 1637, 1508, 1458, 1260, 750, 693. HPLC (IC, 0.46*25 cm, 2 μm, hexane/isopropanol = 99/1, flow 1.0 mL/min, detection at 254 nm) retention time = 4.367 min (minor) and 4.679 min (major).

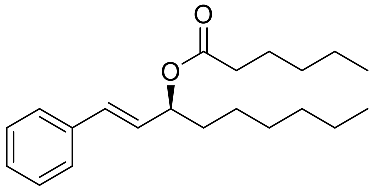

23, 68% yield, 92:8 er

According to General Procedure A, the reaction between diene **S1** (26.0 mg, 0.2 mmol, 1.0 equiv.) and peroxide **O23** (92.0 mg, 0.4 mmol, 2.0 equiv.) afforded product **23** was obtained as a colorless oil (43.0 mg, 68% yield, 92:8 er). $[\alpha]^{28.3}_{D}$ -81.46 (*c* 0.4, $CHCl_3$). ^1H NMR (400 MHz, Chloroform-*d*) δ 7.28 – 7.23 (m, 2H), 7.19 (t, *J* = 7.5 Hz, 2H), 7.14 – 7.08 (m, 1H), 6.50 (d, *J* = 15.9 Hz, 1H), 6.03 (dd, *J* = 15.9, 7.3 Hz, 1H), 5.33 (q, *J* = 6.9 Hz, 1H), 2.21 (t, *J* = 7.5 Hz, 2H), 1.70 – 1.48 (m, 4H), 1.31 – 1.10 (m, 12H), 0.79 (t, *J* = 6.0 Hz, 6H). ^{13}C NMR (101 MHz, Chloroform-*d*) δ 173.10, 136.51, 132.31, 128.55, 128.04, 127.83, 126.57, 74.49, 34.69, 34.66, 31.79, 31.38, 29.12, 25.23, 24.80, 22.63, 22.40, 14.10, 13.96. HRMS (ESI) *m/z* calcd for $[C_{21}H_{32}NaO_2]^+$ ($[M+Na]^+$): 339.2295, found: 339.2296. IR (ν/cm^{-1}) 2956, 2927, 2856, 1734, 1637, 1508, 1458, 1260, 750, 693. HPLC (IC, 0.46*25 cm, 2 μm, hexane/isopropanol = 99/1, flow 1.0 mL/min, detection at 254 nm) retention time = 4.564 min (minor) and 4.914 min (major).

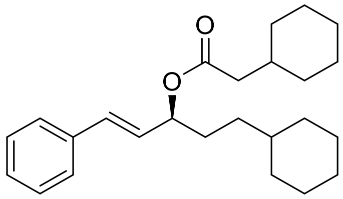

24, 57% yield, 92:8 er

According to General Procedure A, the reaction between diene **S1** (26.0 mg, 0.2 mmol, 1.0 equiv.) and peroxide **O24** (112.8 mg, 0.4 mmol, 2.0 equiv.) afforded product **24** was obtained as a colorless oil (42.0 mg, 57% yield, 92:8 er). $[\alpha]^{28.3}_{D}$ -73.09 (*c* 0.4, $CHCl_3$). ^1H NMR (400 MHz, Chloroform-*d*) δ

7.30 (d, *J* = 7.3 Hz, 2H), 7.23 (t, *J* = 7.6 Hz, 2H), 7.15 (d, *J* = 7.1 Hz, 1H), 6.51 (d, *J* = 15.9 Hz, 1H), 6.05 (dd, *J* = 15.9, 7.2 Hz, 1H), 5.31 (q, *J* = 6.7 Hz, 1H), 2.13 (d, *J* = 7.0 Hz, 2H), 1.79 – 1.57 (m, 12H), 1.28 – 1.04 (m, 10H), 0.96 – 0.86 (m, 2H), 0.85 – 0.75 (m, 2H). ^{13}C NMR (101 MHz, Chloroform-*d*) δ 171.44, 135.48, 131.12, 127.50, 127.05, 126.76, 125.51, 73.64, 41.52, 36.45, 33.98, 32.28, 32.26, 32.01, 31.72, 30.99, 25.61, 25.30, 25.14, 25.01. HRMS (ESI) *m/z* calcd for $[C_{25}H_{36}NaO_2]^+$ ($[M+Na]^+$): 391.2608, found: 391.2608. IR (*v*/cm^{-1}) 3006, 2967, 2926, 2852, 1718, 1637, 1508, 1458, 1260, 750, 703. HPLC (IC, 0.46*25 cm, 2 μm, hexane/isopropanol = 99/1, flow 1 mL/min, detection at 254 nm) retention time = 4.813 min (minor) and 5.219 min (major).

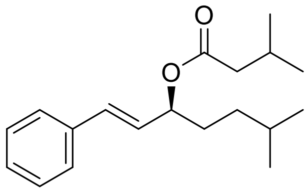

25, 60% yield, 92:8 er

According to General Procedure A, the reaction between diene **S1** (26.0 mg, 0.2 mmol, 1.0 equiv.) and peroxide **O25** (80.8 mg, 0.4 mmol, 2.0 equiv.) afforded product **25** was obtained as a colorless oil (34.6 mg, 60% yield, 92:8 er). $[\alpha]^{28.3}_{D}$ -81.34 (*c* 0.4, $CHCl_3$). ^{1}H NMR (400 MHz, Chloroform-*d*) δ 7.38 (dd, *J* = 7.0, 1.7 Hz, 2H), 7.31 (t, *J* = 7.4 Hz, 2H), 7.23 (d, *J* = 7.2 Hz, 1H), 6.60 (d, *J* = 15.9 Hz, 1H), 6.12 (dd, *J* = 15.9, 7.3 Hz, 1H), 5.40 (q, *J* = 7.0 Hz, 1H), 2.21 (d, *J* = 6.6 Hz, 2H), 2.17 – 2.07 (m, 1H), 1.80 – 1.61 (m, 2H), 1.55 (dd, *J* = 13.3, 6.6 Hz, 2H), 1.27 – 1.17 (m, 1H), 0.96 (d, *J* = 5.9 Hz, 6H), 0.89 (d, *J* = 6.6 Hz, 6H). ^{13}C NMR (101 MHz, Chloroform-*d*) δ 172.51, 136.47, 132.31, 128.55, 128.02, 127.83, 126.55, 74.69, 43.83, 34.21, 32.53, 27.89, 25.81, 22.53, 22.44. HRMS (ESI) *m/z* calcd for $[C_{19}H_{28}NaO_2]^+$ ($[M+Na]^+$): 311.1982, found: 311.1981. IR (*v*/cm^{-1}) 3007, 2959, 2931, 2871, 1733, 1637, 1508, 1458, 1260, 750, 694. HPLC (IC, 0.46*25 cm, 2 μm, hexane/isopropanol = 99/1, flow 1.0 mL/min, detection at 254 nm) retention time = 4.386 min (minor) and 4.682 min (major).

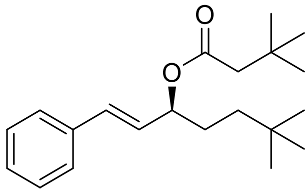

26, 52% yield, 92:8 er

According to General Procedure A, the reaction between diene **S1** (26.0 mg, 0.2 mmol, 1.0 equiv.) and peroxide **O26** (92.0 mg, 0.4 mmol, 2.0 equiv.) afforded product **26** was obtained as a colorless oil (32.9 mg, 52% yield, 92:8 er). $[\alpha]^{28.3}_{D}$ -59.55 (*c* 0.4, $CHCl_3$). ^{1}H NMR (400 MHz, Chloroform-*d*) δ 7.34 – 7.29 (m, 2H), 7.28 – 7.21 (m, 2H), 7.19 – 7.14 (m, 1H), 6.54 (d, *J* = 15.9 Hz, 1H), 6.05 (dd, *J* = 15.9, 7.5 Hz, 1H), 5.30 (q, *J* = 6.8 Hz, 1H), 2.15 (s, 2H), 1.71 – 1.53 (m, 2H), 1.25 – 1.17 (m, 1H), 1.17 – 1.09 (m, 1H), 0.97 (s, 9H), 0.81 (s, 9H). ^{13}C NMR (101 MHz, Chloroform-*d*) δ 170.68, 135.47, 131.44, 127.52, 127.04, 126.79, 125.52, 74.05, 47.26, 38.12, 29.80, 29.04, 28.87, 28.68, 28.27. HRMS (ESI) *m/z* calcd for $[C_{21}H_{32}NaO_2]^+$ ($[M+Na]^+$): 339.2295, found: 339.2296. IR (*v*/cm^{-1}) 3007, 2989, 2959, 2869, 1717, 1637, 1508, 1451, 1260, 750, 704. HPLC (OH-D, 0.46*25 cm, 2 μm, hexane/isopropanol = 99.5/0.5, flow 1.0 mL/min, detection at 254 nm) retention time = 11.630 min (major) and 13.241 min (minor).

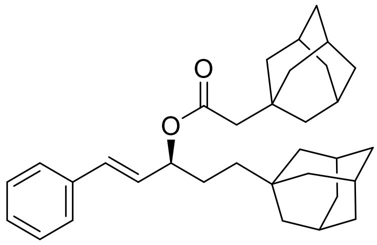

27, 53% yield, 90:10 er

According to General Procedure A, the reaction between diene **S1** (26.0 mg, 0.2 mmol, 1.0 equiv.) and peroxide **O27** (154.4 mg, 0.4 mmol, 2.0 equiv.) afforded product **27** was obtained as a white solid (50.0 mg, 53% yield, 90:10 er). $[\alpha]^{28.3}_{D}$ -73.21 (*c* 0.4, $CHCl_3$). Mp 91.5-93.0 °C. ^{1}H NMR (400 MHz, Chloroform-*d*) δ 7.37 (d, *J* = 7.3 Hz, 2H), 7.31 (t, *J* = 7.5 Hz, 2H), 7.26 – 7.20 (m, 1H), 6.60 (d, *J* = 16.0 Hz, 1H), 6.12 (dd, *J* = 15.9, 7.3 Hz, 1H), 5.36 (q, *J* = 6.7 Hz, 1H), 2.09 (s, 2H), 2.04 – 1.84 (m, 7H), 1.82 – 1.53 (m, 20H), 1.48 – 1.42 (m, 5H), 1.20 – 1.03 (m, 2H). ^{13}C NMR (101 MHz, Chloroform-*d*) δ 171.22, 136.57, 132.28, 128.54, 128.26, 127.77, 126.54, 75.12, 49.30, 42.47, 42.38, 39.61, 37.20, 36.78, 32.89, 31.98, 29.72, 28.70, 28.64, 27.82. HRMS (ESI) *m/z* calcd for $[C_{33}H_{44}NaO_2]^+$ ($[M+Na]^+$): 495.3234, found: 495.3228. IR (*v*/cm^{-1}) 2902, 2847, 1729, 1637, 1508, 1451, 1260, 750, 693. HPLC (IC, 0.46*25 cm, 2 μm, hexane/isopropanol = 99/1, flow 1.0 mL/min, detection at 254 nm) retention time = 5.522 min (minor) and 6.276 min (major).

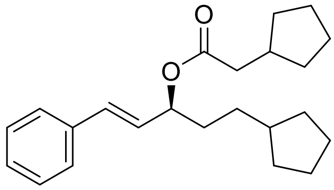

28, 63% yield, 93:7 er

According to General Procedure A, the reaction between diene **S1** (26.0 mg, 0.2 mmol, 1.0 equiv.) and peroxide **O28** (101.6 mg, 0.4 mmol, 2.0 equiv.) afforded product **28** was obtained as a colorless oil (42.8 mg, 63% yield, 93:7 er). $[\alpha]^{28.3}_{D}$ -83.72 (c 0.4, $CHCl_3$). ^{1}H NMR (400 MHz, Chloroform-*d*) δ 7.30 (d, *J* = 7.0 Hz, 2H), 7.24 (t, *J* = 7.4 Hz, 2H), 7.18 – 7.13 (m, 1H), 6.52 (d, *J* = 15.9 Hz, 1H), 6.05 (dd, *J* = 16.0, 7.3 Hz, 1H), 5.33 (q, *J* = 6.8 Hz, 1H), 2.33 – 2.23 (m, 2H), 2.23 – 2.12 (m, 1H), 1.79 – 1.40 (m, 15H), 1.35 – 1.23 (m, 2H), 1.16 – 1.06 (m, 2H), 1.05 – 0.94 (m, 2H). ^{13}C NMR (101 MHz, Chloroform-*d*) δ 172.75, 136.50, 132.21, 128.55, 128.06, 127.81, 126.55, 74.61, 40.86, 39.92, 36.61, 33.87, 32.69, 32.64, 32.48, 32.47, 31.53, 25.17, 25.03. HRMS (ESI) *m/z* calcd for $[C_{23}H_{32}NaO_2]^+$ ($[M+Na]^+$): 363.2295, found: 363.2296. IR (*v*/cm^{-1}) 3007, 2950, 2865, 1732, 1637, 1508, 1451, 1260, 750, 694. HPLC (IC, 0.46*25 cm, 2 μm, hexane/isopropanol = 99/1, flow 1.0 mL/min, detection at 254 nm) retention time = 4.948 min (minor) and 5.511 min (major).

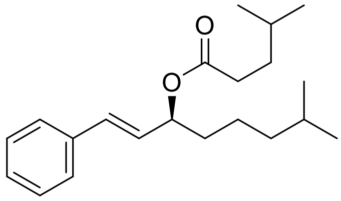

29, 50% yield, 89:11 er

According to General Procedure A, the reaction between diene **S1** (26.0 mg, 0.2 mmol, 1.0 equiv.) and peroxide **O29** (92.0 mg, 0.4 mmol, 2.0 equiv.) afforded product **29** was obtained as a colorless oil (31.6 mg, 50% yield, 89:11 er). $[\alpha]^{28.3}_{D}$ -70.53 (*c* 0.4, $CHCl_3$). ^1H NMR (600 MHz, Chloroform-*d*) δ 7.37 (d, *J* = 7.5 Hz, 2H), 7.30 (t, *J* = 7.6 Hz, 2H), 7.25 – 7.21 (m, 1H), 6.58 (d, *J* = 15.9 Hz, 1H), 6.11 (dd, *J* = 15.9, 7.3 Hz, 1H), 5.40 (q, *J* = 6.9 Hz, 1H), 2.34 – 2.30 (m, 2H), 1.74 – 1.67 (m, 1H), 1.64 – 1.52 (m, 5H), 1.39 – 1.28 (m, 2H), 1.23 – 1.14 (m, 2H), 0.89 (d, *J* = 6.3, 6H), 0.85 (d, *J* = 6.6 Hz, 6H). ^{13}C NMR (151 MHz, Chloroform-*d*) δ 173.52, 136.53, 132.37, 128.64, 128.06, 127.93, 126.64, 74.62, 38.72, 34.90, 33.93, 32.86, 27.95, 27.78, 23.08, 22.66, 22.65, 22.37, 22.33. HRMS (ESI) *m/z* calcd for $[C_{21}H_{32}NaO_2]^+$ ($[M+Na]^+$): 339.2295, found: 339.2295. IR (*v*/cm^{-1}) 3007, 2956, 2869, 1733, 1637, 1508, 1451, 1260, 750, 693. HPLC (IC, 0.46*25 cm, 2 μm, hexane/isopropanol = 99/1, flow 1.0 mL/min, detection at 254 nm) retention time = 4.367 min (minor) and 4.659 min (major).

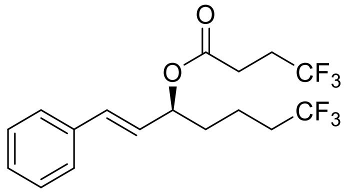

30, 78% yield, 93:7 er

According to General Procedure A, the reaction between diene **S1** (26.0 mg, 0.2 mmol, 1.0 equiv.) and peroxide **O30** (112.8 mg, 0.4 mmol, 2.0 equiv.) afforded product **30** was obtained as a colorless oil (57.4 mg, 78% yield, 93:7 er). $[\alpha]^{28.3}_{D}$ -58.37 (*c* 0.4, $CHCl_3$). ^1H NMR (600 MHz, Chloroform-*d*) δ 7.39 – 7.35 (m, 2H), 7.34 – 7.30 (m, 2H), 7.28 – 7.26 (m, 1H), 6.62 (d, *J* = 15.8 Hz, 1H), 6.20 – 5.99 (m, 1H), 5.43 (q, *J* = 7.1 Hz, 1H), 2.64 – 2.57 (m, 2H), 2.52 – 2.39 (m, 2H), 2.17 – 2.05 (m, 2H), 1.87 – 1.79 (m, 2H), 1.80 – 1.71 (m, 2H), 1.69 – 1.60 (m, 2H). ^{13}C NMR (151 MHz, Chloroform-*d*) δ 169.24, 134.78, 132.73, 127.63, 127.27, 125.87 (q, *J* = 274.4 Hz), 125.62, 125.42 (q, *J* = 274.2 Hz), 125.14, 73.98, 32.41, 32.36 (q, *J* = 28.8 Hz), 28.28 (q, *J* = 30.1 Hz), 26.34, 16.77. HRMS (ESI) *m/z* calcd for $[C_{17}H_{18}F_6NaO_2]^+$ ($[M+Na]^+$): 391.1103, found: 391.1102. IR (*v*/cm^{-1}) 3007, 2961, 2851, 1735, 1637, 1508, 1451, 1260, 750, 694. HPLC (OD-H, 0.46*25 cm, 2 μm, hexane/isopropanol = 99.5/0.5, flow 1.0 mL/min, detection at 254 nm) retention time = 14.342 min (major) and 17.769 min (minor).

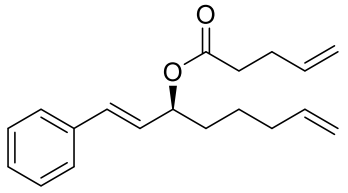

31, 53% yield, 92:8 er

According to General Procedure A, the reaction between diene **S1** (26.0 mg, 0.2 mmol, 1.0 equiv.) and peroxide **O31** (79.2 mg, 0.4 mmol, 2.0 equiv.) afforded product **31** was obtained as a colorless oil (30.1 mg, 53% yield, 92:8 er). $[\alpha]^{28.3}_{D}$ -69.85 (*c* 0.4, $CHCl_3$). ^1H NMR (600 MHz, Chloroform-*d*) δ 7.38 – 7.35 (m, 2H), 7.32 – 7.29 (m, 2H), 7.24 – 7.22 (m, 1H), 6.59 (d, *J* = 15.9 Hz, 1H), 6.11 (dd, *J* = 15.9, 7.3 Hz, 1H), 5.87 – 5.74 (m, 2H), 5.46 – 5.39 (m, 1H), 5.09 – 4.93 (m, 4H), 2.47 – 2.35 (m, 4H), 2.11 – 2.05 (m, 2H), 1.79 – 1.71 (m, 1H), 1.71 – 1.64 (m, 1H), 1.52 – 1.39 (m, 2H). ^{13}C NMR (151 MHz, Chloroform-*d*) δ 172.50, 138.39, 136.78, 136.42, 132.59, 128.66, 128.00, 127.76, 126.65, 115.63, 115.01, 74.71, 34.11, 33.93, 33.52, 29.03, 24.55. HRMS (ESI) *m/z* calcd for $[C_{19}H_{24}NaO_2]^+$ ($[M+Na]^+$): 307.1669, found: 307.1668. IR (*v*/cm^{-1}) 3007, 2990, 2852, 1733, 1637, 1508, 1451, 1260, 750, 693.

HPLC (IC, 0.46*25 cm, 2 μm, hexane/isopropanol = 99/1, flow 1.0 mL/min, detection at 254 nm) retention time = 5.007 min (minor) and 5.538 min (major).

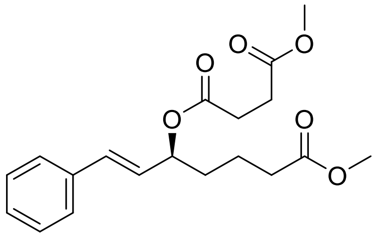

32, 60% yield, 93:7 er

According to General Procedure A, the reaction between diene **S1** (26.0 mg, 0.2 mmol, 1.0 equiv.) and peroxide **O32** (104.8 mg, 0.4 mmol, 2.0 equiv.) afforded product **32** was obtained as a colorless oil (41.8 mg, 60% yield, 93:7 er). [α] $^{28.3}{}_{D}$ -112.44 (*c* 0.4, $CHCl_3$). ^1H NMR (600 MHz, Chloroform-*d*) δ 7.36 (dd, *J* = 8.3, 1.2 Hz, 2H), 7.30 (t, *J* = 7.6 Hz, 2H), 7.25 – 7.21 (m, 1H), 6.59 (d, *J* = 15.9 Hz, 1H), 6.09 (dd, *J* = 15.9, 7.3 Hz, 1H), 5.41 (q, *J* = 7.0 Hz, 1H), 3.66 (s, 3H), 3.65 (s, 3H), 2.69 – 2.59 (m, 4H), 2.34 (t, *J* = 7.2 Hz, 2H), 1.81 – 1.66 (m, 4H). ^{13}C NMR (101 MHz, Chloroform-*d*) δ 172.63, 171.67, 170.53, 135.13, 131.86, 127.54, 126.97, 125.93, 125.58, 73.65, 50.83, 50.56, 32.84, 32.59, 28.39, 27.93, 19.54. HRMS (ESI) *m/z* calcd for $[C_{19}H_{24}NaO_6]^+$ ($[M+Na]^+$): 371.1465, found: 371.1467. IR (v/cm^{-1}) 3007, 2990, 2852, 1733, 1637, 1508, 1451, 1260, 750, 695. HPLC (OD-H, 0.46*25 cm, 2 μm, hexane/isopropanol = 95/5, flow 1.0 mL/min, detection at 254 nm) retention time = 15.896 min (major) and 17.235 min (minor).

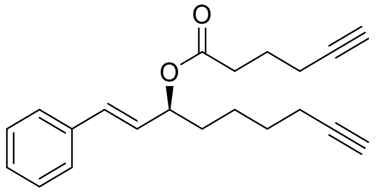

33, 84% yield, 93:7 er

According to General Procedure A, the reaction between diene **S1** (26.0 mg, 0.2 mmol, 1.0 equiv.) and peroxide **O33** (88.8 mg, 0.4 mmol, 2.0 equiv.) afforded product **33** was obtained as a colorless oil (51.7 mg, 84% yield, 93:7 er). [α] $^{28.3}{}_{D}$ -88.37 (*c* 0.4, $CHCl_3$). ^1H NMR (400 MHz, Chloroform-*d*) δ 7.30 (d, *J* = 7.2 Hz, 2H), 7.24 (t, *J* = 7.4 Hz, 2H), 7.20 – 7.14 (m, 1H), 6.53 (d, *J* = 15.9 Hz, 1H), 6.05 (dd, *J* = 15.9, 7.3 Hz, 1H), 5.36 (q, *J* = 6.9 Hz, 1H), 2.41 (t, *J* = 7.4 Hz, 2H), 2.20 (td, *J* = 6.9, 2.7 Hz, 2H), 2.13 (td, *J* = 6.9, 2.7 Hz, 2H), 1.90 (t, *J* = 2.7 Hz, 1H), 1.87 (t, *J* = 2.7 Hz, 1H), 1.81 – 1.75 (m, 2H), 1.73 – 1.59 (m, 2H), 1.53 – 1.34 (m, 4H). ^{13}C NMR (101 MHz, Chloroform-*d*) δ 171.34, 135.26, 131.61, 127.55, 126.93, 126.49, 125.56, 83.16, 82.26, 73.53, 68.15, 67.49, 33.02, 32.20, 27.10, 23.25, 22.63, 17.26, 16.84. HRMS (ESI) *m/z* calcd for $[C_{21}H_{24}NaO_2]^+$ ($[M+Na]^+$): 331.1669, found: 331.1670. IR (v/cm^{-1}) 2990, 2865, 2065, 1717, 1637, 1508, 1451, 1260, 750, 695. HPLC (IC, 0.46*25 cm, 2 μm, hexane/isopropanol = 99/1, flow 1.0 mL/min, detection at 254 nm) retention time = 8.239 min (minor) and 8.962 min (major).

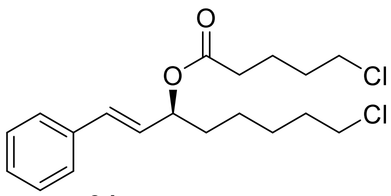

34, 77% yield, 92:8 er

According to General Procedure A, the reaction between diene **S1** (26.0 mg, 0.2 mmol, 1.0 equiv.) and peroxide **O34** (108.0 mg, 0.4 mmol, 2.0 equiv.) afforded product **34** was obtained as a colorless oil (54.8 mg, 77% yield, 92:8 er). $[\alpha]^{28.3}_{D}$ -99.26 (*c* 0.4, $CHCl_3$). ^1H NMR (400 MHz, Chloroform-*d*) δ 7.31 (d, *J* = 7.4 Hz, 2H), 7.24 (t, *J* = 7.5 Hz, 2H), 7.19 (t, *J* = 3.5 Hz, 1H), 6.53 (d, *J* = 15.9 Hz, 1H), 6.04 (dd, *J* = 15.9, 7.4 Hz, 1H), 5.35 (q, *J* = 6.9 Hz, 1H), 3.48 – 3.44 (m, 4H), 2.30 (t, *J* = 6.7 Hz, 2H), 1.80 – 1.55 (m, 8H), 1.45 – 1.36 (m, 2H), 1.36 – 1.23 (m, 2H). ^{13}C NMR (101 MHz, Chloroform-*d*) δ 171.45, 135.20, 131.71, 127.56, 126.96, 126.45, 125.54, 73.63, 43.90, 43.44, 33.39, 32.71, 31.39, 30.82, 25.59, 23.50, 21.28. HRMS (ESI) *m/z* calcd for $[C_{19}H_{26}Cl_2NaO_2]^+$ ($[M+Na]^+$): 379.1202, found: 379.1201. IR (*v*/cm^{-1}) 2958, 2867, 1718, 1637, 1508, 1451, 1260, 750, 694. HPLC (IC, 0.46*25 cm, 2 μm, hexane/isopropanol = 99/1, flow 1.0 mL/min, detection at 254 nm) retention time = 11.404 min (minor) and 13.446 min (major).

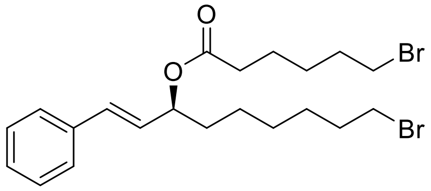

35, 66% yield, 93:7 er

According to General Procedure A, the reaction between diene **S1** (26.0 mg, 0.2 mmol, 1.0 equiv.) and peroxide **O35** (154.4 mg, 0.4 mmol, 2.0 equiv.) afforded product **35** was obtained as a colorless oil (31.15 mg, 66% yield, 93:7 er). $[\alpha]^{28.3}_{D}$ -59.38 (*c* 0.4, $CHCl_3$). ^1H NMR (400 MHz, Chloroform-*d*) δ 7.30 (d, *J* = 7.4 Hz, 2H), 7.24 (t, *J* = 7.5 Hz, 2H), 7.21 – 7.14 (m, 1H), 6.52 (d, *J* = 15.9 Hz, 1H), 6.04 (dd, *J* = 15.9, 7.4 Hz, 1H), 5.34 (q, *J* = 6.9 Hz, 1H), 3.40 – 3.24 (m, 4H), 2.28 (t, *J* = 7.4 Hz, 2H), 1.86 – 1.72 (m, 4H), 1.71 – 1.51 (m, 4H), 1.47 – 1.33 (m, 4H), 1.32 – 1.23 (m, 4H). ^{13}C NMR (101 MHz, Chloroform-*d*) δ 171.75, 135.27, 131.55, 127.54, 126.91, 126.64, 125.53, 73.58, 33.44, 33.36, 32.85, 32.50, 31.63, 31.37, 27.47, 26.99, 26.62, 24.00, 23.13. HRMS (ESI) *m/z* calcd for $[C_{21}H_{30}Br_2NaO_2]^+$ ($[M+Na]^+$): 495. 0505, found: 495.0505. IR (*v*/cm^{-1}) 2990, 2960, 2855, 1718, 1637, 1508, 1451, 1260, 750, 693. HPLC (IC, 0.46*25 cm, 2 μm, hexane/isopropanol = 99/1, flow 1.0 mL/min, detection at 254 nm) retention time = 12.563 min (minor) and 13.761 min (major).

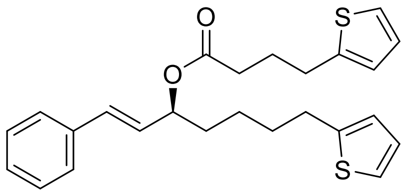

36, 67% yield, 93:7 er

According to General Procedure A, the reaction between diene **S1** (26.0 mg, 0.2 mmol, 1.0 equiv.) and peroxide **O36** (135.2 mg, 0.4 mmol, 2.0 equiv.) afforded product **36** was obtained as a colorless oil (56.8 mg, 67% yield, 93:7 er). $[\alpha]^{28.3}_{D}$ -82.94 (*c* 0.4, $CHCl_3$). ^1H NMR (600 MHz, Chloroform-*d*) δ 7.39 – 7.36 (m, 2H), 7.34 – 7.30 (m, 2H), 7.27 – 7.23 (m, 1H), 7.12 (dd, *J* = 5.1, 1.2 Hz, 1H), 7.09 (dd, *J* = 5.1, 1.2 Hz, 1H), 6.95 – 6.86 (m, 2H), 6.83 – 6.75 (m, 1H), 6.80 – 6.73 (m, 1H), 6.60 (d, *J* = 15.8 Hz, 1H), 6.11 (dd, *J* = 16.0, 6.9 Hz, 1H), 5.43 (q, *J* = 7.2 Hz, 1H), 2.88 (t, *J* = 7.7 Hz, 2H), 2.83 (t, *J* = 7.7 Hz, 2H), 2.38 (t, *J* = 7.5 Hz, 2H), 2.02 (q, *J* = 7.3 Hz, 2H), 1.82 – 1.66 (m, 4H), 1.49 – 1.39 (m, 2H). ^{13}C NMR (151 MHz, Chloroform-*d*) δ 172.68, 145.30, 144.20, 136.38, 132.72, 128.68, 128.05, 127.70, 126.90, 126.79, 126.69, 124.66, 124.20, 123.35, 123.01, 74.69, 34.39, 33.76, 31.59, 29.82, 29.21, 26.99,

24.75. HRMS (ESI) *m/z* calcd for $[C_{25}H_{28}NaO_2S_2]^+$ ($[M+Na]^+$): 447.1423, found: 447.1421. IR (ν/cm^{-1}) 3006, 2989, 2923, 2851, 1719, 1637, 1508, 1451, 1260, 750, 703. HPLC (IC, 0.46*25 cm, 2 μm, hexane/isopropanol = 99/1, flow 1.0 mL/min, detection at 254 nm) retention time = 12.810 min (minor) and 15.078 min (major).

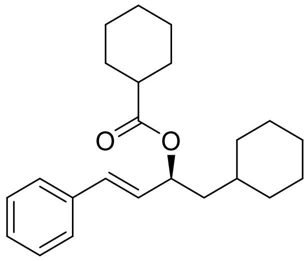

37, 55% yield, 93:7 er

According to General Procedure A, the reaction between diene **S1** (26.0 mg, 0.2 mmol, 1.0 equiv.) and peroxide **O37** (101.6 mg, 0.4 mmol, 2.0 equiv.) afforded product **37** was obtained as a colorless oil (37.4 mg, 55% yield, 93:7 er). $[\alpha]^{28.3}_{D}$ -78.02 (*c* 0.4, $CHCl_3$). ^{1}H NMR (600 MHz, Chloroform-*d*) δ 7.38 – 7.35 (m, 2H), 7.33 – 7.28 (m, 2H), 7.24 – 7.21 (m, 1H), 6.57 (d, *J* = 15.9 Hz, 1H), 6.10 (dd, *J* = 15.9, 7.2 Hz, 1H), 5.54 – 5.47 (m, 1H), 2.33 – 2.26 (m, 1H), 1.95 – 1.85 (m, 2H), 1.79 – 1.59 (m, 8H), 1.52 – 1.40 (m, 4H), 1.39 – 1.30 (m, 1H), 1.30 – 1.10 (m, 6H), 0.99 – 0.84 (m, 2H). ^{13}C NMR (151 MHz, Chloroform-*d*) δ 175.54, 136.62, 132.01, 128.62, 128.52, 127.87, 126.64, 72.14, 43.56, 42.33, 34.04, 33.61, 33.09, 29.22, 29.03, 26.57, 26.32, 26.26, 25.88, 25.60, 25.51. HRMS (ESI) *m/z* calcd for $[C_{23}H_{32}NaO_2]^+$ ($[M+Na]^+$): 363. 2295, found: 363.2296. IR (ν/cm^{-1}) 3006, 2989, 2923, 2851, 1719, 1637, 1508, 1451, 1260, 750, 704. HPLC (OD-H, 0.46*25 cm, 2 μm, hexane/isopropanol = 100/0, flow 1.0 mL/min, detection at 254 nm) retention time = 16.538 min (major) and 19.207 min (minor).

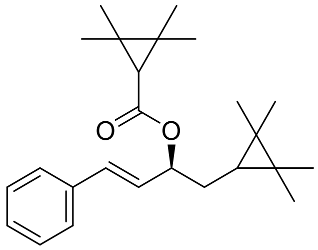

38, 48% yield, 91:9 er

According to General Procedure A, the reaction between diene **S1** (26.0 mg, 0.2 mmol, 1.0 equiv.) and peroxide **O38** (112.8 mg, 0.4 mmol, 2.0 equiv.) afforded product **38** was obtained as a colorless oil (35.3 mg, 48% yield, 91:9 er). $[\alpha]^{28.3}_{D}$ -53.68 (*c* 0.4, $CHCl_3$). ^{1}H NMR (400 MHz, Chloroform-*d*) δ 7.39 (d, *J* = 7.2 Hz, 2H), 7.31 (t, *J* = 7.6 Hz, 2H), 7.28 – 7.19 (m, 1H), 6.57 (d, *J* = 15.9 Hz, 1H), 6.17 (dd, *J* = 15.9, 7.2 Hz, 1H), 5.43 (q, *J* = 6.8 Hz, 1H), 1.70 (q, *J* = 7.0 Hz, 1H), 1.67 – 1.60 (m, 1H), 1.26 (s, 3H), 1.24 (s, 3H), 1.23 (s, 1H), 1.20 (s, 3H), 1.19 (s, 3H), 1.05 (s, 3H), 1.02 (s, 3H), 0.94 (s, 3H), 0.91 (s, 3H), 0.19 (t, *J* = 6.9 Hz, 1H). ^{13}C NMR (101 MHz, Chloroform-*d*) δ 171.36, 136.70, 131.44, 128.66, 128.54, 127.65, 126.49, 74.43, 36.08, 30.79, 29.93, 29.90, 29.72, 23.64, 23.60, 23.59, 20.82, 17.19, 17.11, 16.68, 16.66. HRMS (ESI) *m/z* calcd for $[C_{25}H_{36}NaO_2]^+$ ($[M+Na]^+$): 391.2608, found: 391.2608. IR (ν/cm^{-1}) 2988, 2924, 2867, 1719, 1637, 1508, 1451, 1260, 750, 693. HPLC (IC, 0.46*25 cm, 2 μm, hexane/isopropanol = 99.7/0.3, flow 1.0 mL/min, detection at 254 nm) retention time = 4.447 min (minor) and 4.698 min (major).

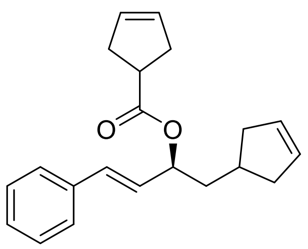

39, 75% yield, 95:5 er

According to General Procedure A, the reaction between diene **S1** (26.0 mg, 0.2 mmol, 1.0 equiv.) and peroxide **O39** (88.8 mg, 0.4 mmol, 2.0 equiv.) afforded product **39** was obtained as a colorless oil (46.2 mg, 75% yield, 95:5 er). $[\alpha]^{28.3}_{D}$ -67.44 (*c* 0.4, $CHCl_3$). ^1H NMR (400 MHz, Chloroform-*d*) δ 7.30 (d, *J* = 7.5 Hz, 2H), 7.24 (t, *J* = 7.6 Hz, 2H), 7.18 – 7.13 (m, 1H), 6.54 (d, *J* = 16.0 Hz, 1H), 6.07 (dd, *J* = 16.0, 7.4 Hz, 1H), 5.62 – 5.56 (m, 4H), 5.41 (q, *J* = 7.4 Hz, 1H), 3.07 (p, *J* = 8.4 Hz, 1H), 2.66 – 2.54 (m, 4H), 2.49 – 2.37 (m, 2H), 2.30 – 2.17 (m, 1H), 2.06 – 1.92 (m, 2H), 1.89 – 1.80 (m, 1H), 1.76 – 1.66 (m, 1H). ^{13}C NMR (101 MHz, $CDCl_3$) δ 175.45, 136.41, 132.41, 129.86, 129.80, 129.05, 128.94, 128.57, 127.94, 127.90, 126.58, 74.10, 41.84, 41.17, 39.01, 39.00, 36.37, 36.28, 34.13. HRMS (ESI) *m/z* calcd for $[C_{21}H_{24}NaO_2]^+$ ($[M+Na]^+$): 331.1669, found: 331.1667. IR (ν/cm^{-1}) 2925, 2854, 1719, 1637, 1508, 1451, 1260, 750, 694. HPLC (OD-H, 0.46*25 cm, 2 μm, hexane/isopropanol = 99.5/0.5, flow 1.0 mL/min, detection at 254 nm) retention time = 7.198 min (major) and 9.494 min (minor).

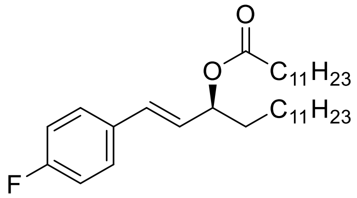

40, 50% yield, 92:8 er

According to General Procedure A, the reaction between diene **S10** (29.6 mg, 0.2 mmol, 1.0 equiv.) and peroxide **O1** (159.2 mg, 0.4 mmol, 2.0 equiv.) afforded product **40** was obtained as a white solid (50.2 mg, 50% yield, 92:8 er). $[\alpha]^{28.3}_{D}$ -44.68 (*c* 0.4, $CHCl_3$). Mp 43.1-44.2 °C. ^1H NMR (400 MHz, Chloroform-*d*) δ 7.34 (dd, *J* = 8.6, 5.5 Hz, 2H), 7.00 (t, *J* = 8.6 Hz, 2H), 6.55 (d, *J* = 15.9 Hz, 1H), 6.03 (dd, *J* = 15.9, 7.3 Hz, 1H), 5.39 (q, *J* = 6.8 Hz, 1H), 2.32 (t, *J* = 7.5 Hz, 2H), 1.84 – 1.69 (m, 1H), 1.68 – 1.53 (m, 4H), 1.35 – 1.18 (m, 35H), 0.88 (t, *J* = 6.8 Hz, 6H). ^{13}C NMR (101 MHz, Chloroform-*d*) δ 173.25, 162.45 (d, *J* = 246.9 Hz), 132.62 (d, *J* = 3.3 Hz), 131.09, 128.08 (d, *J* = 8.1 Hz), 127.80 (d, *J* = 2.2 Hz), 115.45 (d, *J* = 21.6 Hz), 74.43, 34.71, 34.62, 31.93, 29.69, 29.66, 29.63, 29.59, 29.53, 29.51, 29.40, 29.38, 29.35, 29.31, 29.18, 25.22, 25.09, 22.71, 14.14. HRMS (ESI) *m/z* calcd for $[C_{33}H_{55}FNaO_2]^+$ ($[M+Na]^+$): 525.4078, found: 525.4076. IR (ν/cm^{-1}) 3006, 2918, 2851, 1734, 1654, 1508, 1451, 1260, 750. HPLC (OD-H, 0.46*25 cm, 1 μm, hexane/isopropanol = 99.5/0.5, flow 1.0 mL/min, detection at 254 nm) retention time = 4.327 min (major) and 5.215 min (minor).

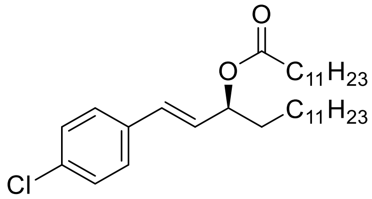

41, 70% yield, 93:7 er

According to General Procedure A, the reaction between diene **S41** (32.8 mg, 0.2 mmol, 1.0 equiv.) and peroxide **O1** (159.2 mg, 0.4 mmol, 2.0 equiv.) with 5 mol% of CuOTf 0.5PhMe and 7 mol% of

ligand **L1** for 5 days afforded product **41** was obtained as a white solid (72.5 mg, 70% yield, 93:7 er). $[\alpha]^{28.3}_{D}$ -51.36 (*c* 0.4, $CHCl_3$). Mp 40.4-42.5 °C. ^{1}H NMR (600 MHz, Chloroform-*d*) δ 7.30 – 7.25 (m, 4H), 6.60 – 6.42 (m, 1H), 6.08 (dd, *J* = 15.9, 7.2 Hz, 1H), 5.38 (q, *J* = 6.4 Hz, 1H), 2.31 (t, *J* = 7.7 Hz, 2H), 1.74 – 1.67 (m, 1H), 1.66 – 1.55 (m, 4H), 1.31 – 1.19 (m, 35H), 0.86 (t, *J* = 7.0 Hz, 6H). ^{13}C NMR (151 MHz, Chloroform-*d*) δ 173.35, 135.03, 133.53, 131.02, 128.80, 128.78, 127.83, 74.39, 34.77, 34.64, 32.02, 29.77, 29.75, 29.72, 29.67, 29.61, 29.59, 29.46, 29.45, 29.40, 29.25, 25.29, 25.16, 22.80, 14.24. HRMS (ESI) *m/z* calcd for $[C_{33}H_{55}ClNaO_2]^+$ ($[M+Na]^+$): 541.3783, found: 541.3778. IR (*v*/cm^{-1}) 3006, 2918, 2851, 1774, 1733, 1508, 1451, 1260, 750. HPLC (OD-H, 0.46*25 cm, 1 μm, hexane/isopropanol = 100/0, flow 1.0 mL/min, detection at 254 nm) retention time = 19.282 min (minor) and 22.726 min (major).

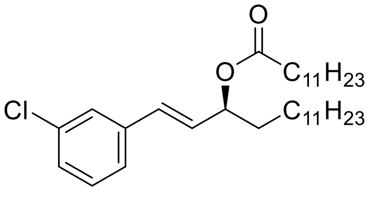

42, 67% yield, 93:7 er

According to General Procedure A, the reaction between diene **S5** (32.8 mg, 0.2 mmol, 1.0 equiv.) and peroxide **O1** (159.2 mg, 0.4 mmol, 2.0 equiv.) with 5 mol% of CuOTf 0.5PhMe and 7 mol% of ligand **L1** for 5 days afforded product **42** was obtained as white solid (69.4 mg, 67% yield, 93:7 er). $[\alpha]^{28.3}_{D}$ -40.67 (*c* 0.4, $CHCl_3$). Mp 36.5-38.2 °C. ^{1}H NMR (400 MHz, Chloroform-*d*) δ 7.29 (d, *J* = 2.2 Hz, 1H), 7.26 – 7.11 (m, 3H), 6.45 (d, *J* = 15.9 Hz, 1H), 6.06 (dd, *J* = 15.9, 7.1 Hz, 1H), 5.32 (q, *J* = 6.7 Hz, 1H), 2.25 (t, *J* = 7.5 Hz, 2H), 1.69 – 1.46 (m, 5H), 1.40 – 1.07 (m, 35H), 0.81 (t, *J* = 6.9 Hz, 6H). ^{13}C NMR (101 MHz, Chloroform-*d*) δ 172.17, 137.32, 133.48, 129.69, 128.72, 128.59, 126.70, 125.34, 123.81, 73.11, 33.64, 33.50, 30.89, 28.65, 28.62, 28.59, 28.55, 28.47, 28.36, 28.34, 28.31, 28.28, 28.14, 24.14, 24.04, 21.67, 13.10. HRMS (ESI) *m/z* calcd for $[C_{33}H_{55}ClNaO_2]^+$ ($[M+Na]^+$): 541.3783, found: 541.3781. IR (*v*/cm^{-1}) 2920, 2852, 1717, 1653, 1508, 1451, 1260, 750. HPLC (IC, 0.46*25 cm, 2 μm, hexane/isopropanol = 99/1, flow 1.0 mL/min, detection at 254 nm) retention time = 4.027 min (minor) and 4.236 min (major).

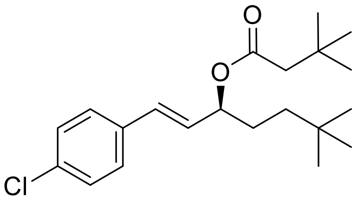

43, 65% yield, 93:7 er

According to General Procedure A, the reaction between diene **S41** (29.6 mg, 0.2 mmol, 1.0 equiv.) and peroxide **O26** (92.0 mg, 0.4 mmol, 2.0 equiv.) afforded product **43** was obtained as a colorless oil (45.5 mg, 65% yield, 93:7 er). $[\alpha]^{28.3}_{D}$ -53.37 (*c* 0.4, $CHCl_3$). ^{1}H NMR (400 MHz, Chloroform-*d*) δ 7.34 – 7.26 (m, 4H), 6.56 (d, *J* = 15.9 Hz, 1H), 6.09 (dd, *J* = 15.9, 7.3 Hz, 1H), 5.36 (q, *J* = 6.8 Hz, 1H), 2.22 (s, 2H), 1.82 – 1.53 (m, 2H), 1.38 – 1.27 (m, 1H), 1.24 – 1.13 (m, 1H), 1.04 (s, 9H), 0.88 (s, 9H). ^{13}C NMR (101 MHz, Chloroform-*d*) δ 171.72, 134.98, 133.47, 131.20, 128.77, 128.72, 127.75, 74.88, 48.26, 39.16, 30.85, 30.08, 29.85, 29.71, 29.29. HRMS (ESI) *m/z* calcd for $[C_{21}H_{31}ClNaO_2]^+$ ($[M+Na]^+$): 373.1905, found: 373.1906. IR (*v*/cm^{-1}) 2958 2869, 1775, 1732, 1492, 1260, 750. HPLC (OD-H, 0.46*25 cm, 2 μm, hexane/isopropanol = 100/1, flow 0.5 mL/min, detection at 254 nm) retention time = 17.368 min (minor) and 22.558 min (major).

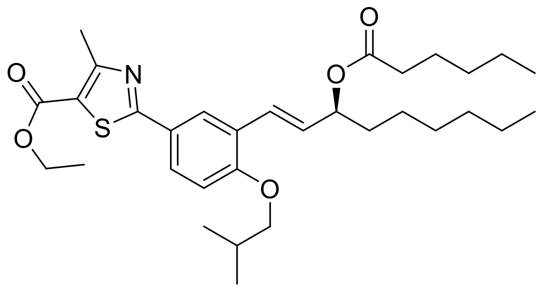

44, 56% yield, 88:12 er

According to General Procedure A, the reaction between diene **S44** (74.2 mg, 0.2 mmol, 1.0 equiv.) and peroxide **O23** (92.0 mg, 0.4 mmol, 2.0 equiv.) afforded product **44** was obtained as a colorless oil (62.4 mg, 56% yield, 88:12 er). $[\alpha]^{28.3}_{D}$ -52.96 (*c* 0.4, $CHCl_3$). ^1H NMR (400 MHz, Chloroform-*d*) δ 8.01 (d, *J* = 2.3 Hz, 1H), 7.80 (dd, *J* = 8.6, 2.3 Hz, 1H), 7.01 – 6.80 (m, 2H), 6.31 (dd, *J* = 16.1, 7.0 Hz, 1H), 5.45 (q, *J* = 6.4 Hz, 1H), 4.35 (q, *J* = 7.2 Hz, 2H), 3.81 (d, *J* = 6.5 Hz, 2H), 2.77 (s, 3H), 2.34 (t, *J* = 7.5 Hz, 2H), 2.16 (p, *J* = 6.7 Hz, 1H), 1.77 – 1.59 (m, 3H), 1.43 – 1.22 (m, 16H), 1.07 (d, *J* = 6.7 Hz, 6H), 0.95 – 0.82 (m, 6H). ^{13}C NMR (101 MHz, Chloroform-*d*) δ 173.19, 169.89, 162.39, 160.97, 158.70, 129.99, 127.49, 126.25, 125.99, 125.53, 125.45, 120.85, 111.91, 74.86, 74.74, 61.14, 34.69, 34.65, 31.75, 31.37, 29.09, 28.36, 25.16, 24.75, 22.59, 22.35, 19.33, 19.31, 17.55, 14.37, 14.08, 13.94. HRMS (ESI) *m/z* calcd for $[C_{32}H_{47}NNaO_5S]^+$ ($[M+Na]^+$): 580.3067, found: 580.3066. IR (*v*/cm^{-1}) 2958, 2929, 2859, 1733, 1715, 1508, 1451, 1260, 750. HPLC (AD-H, 0.46*25 cm, 2 μm, hexane/isopropanol = 99/1, flow 1.0 mL/min, detection at 254 nm) retention time = 18.863 min (minor) and 26.936 min (major).

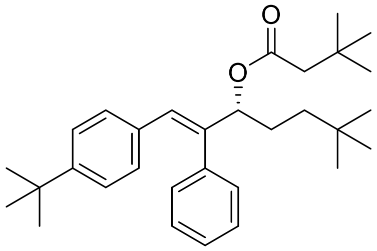

45, 60% yield, 94:6 er

According to General Procedure B, the reaction between diene **S45** (52.4 mg, 0.2 mmol, 1.0 equiv.) and peroxide **O26** (55.2 mg, 0.24 mmol, 1.2 equiv.) afforded product **45** was obtained as a colorless oil (53.8 mg, 60% yield, 94:6 er). $[\alpha]^{28.3}_{D}$ 34.02 (*c* 0.4, $CHCl_3$). ^1H NMR (400 MHz, Chloroform-*d*) δ 7.34 – 7.24 (m, 3H), 7.18 (dd, *J* = 8.9, 2.4 Hz, 2H), 7.03 (d, *J* = 8.4 Hz, 2H), 6.77 (d, *J* = 8.4 Hz, 2H), 6.51 (s, 1H), 5.40 (t, *J* = 6.4 Hz, 1H), 2.23 – 2.11 (m, 2H), 1.57 – 1.44 (m, 2H), 1.28 – 1.20 (m, 2H), 1.15 (s, 9H), 0.97 (s, 9H), 0.74 (s, 9H). ^{13}C NMR (101 MHz, Chloroform-*d*) δ 170.59, 148.92, 139.07, 137.53, 132.27, 128.32, 127.98, 127.58, 127.42, 126.34, 123.83, 78.17, 47.21, 38.21, 33.40, 30.15, 29.79, 29.02, 28.70, 28.27, 27.11. HRMS (ESI) *m/z* calcd for $[C_{31}H_{44}NaO_2]^+$ ($[M+Na]^+$): 471.3234, found: 471.3233. IR (*v*/cm^{-1}) 2958, 2868, 1734, 1653, 1467, 1260, 750, 704. HPLC (IC, 0.46*25 cm, 2 μm, hexane/isopropanol = 99.8/0.2, flow 0.8 mL/min, detection at 254 nm) retention time = 6.779 min (major) and 8.229 min (minor).

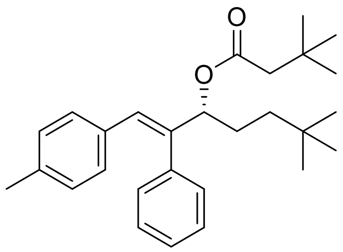

46, 69% yield, 97:3 er

According to General Procedure B, the reaction between diene **S46** (44.0 mg, 0.2 mmol, 1.0 equiv.) and peroxide **O26** (55.2 mg, 0.24 mmol, 1.2 equiv.) afforded product **46** was obtained as a colorless oil (56.0 mg, 69% yield, 97:3 er). $[\alpha]^{28.3}_{D}$ 37.02 (*c* 0.4, $CHCl_3$). ^1H NMR (400 MHz, Chloroform-*d*) δ 7.30 – 7.21 (m, 3H), 7.18 – 7.13 (m, 2H), 6.82 (d, *J* = 8.3 Hz, 2H), 6.73 (d, *J* = 8.2 Hz, 2H), 6.52 (s, 1H), 5.41 (t, *J* = 6.5 Hz, 1H), 2.21 – 2.17 (m, 2H), 2.15 (s, 3H), 1.55 – 1.51 (m, 1H), 1.50 – 1.45 (m, 1H), 1.27 – 1.19 (m, 1H), 1.16 – 1.07 (m, 1H), 0.98 (s, 9H), 0.74 (s, 9H). ^{13}C NMR (151 MHz, Chloroform-*d*) δ 171.82, 140.31, 138.56, 136.76, 133.49, 129.48, 129.32, 128.71, 128.67, 128.59, 127.45, 79.17, 48.34, 39.37, 30.95, 30.15, 29.82, 29.38, 28.29, 21.21. HRMS (ESI) *m/z* calcd for $[C_{28}H_{38}NaO_2]^+$ ($[M+Na]^+$): 429.2764, found: 429.2764. IR (ν/cm^{-1}) 2958, 2867, 1733, 1636, 1473, 1260, 750, 702. HPLC (IC, 0.46*25 cm, 2 μm, hexane/isopropanol = 99.8/0.2, flow 0.8 mL/min, detection at 254 nm) retention time = 7.136 min (major) and 10.148 min (minor).

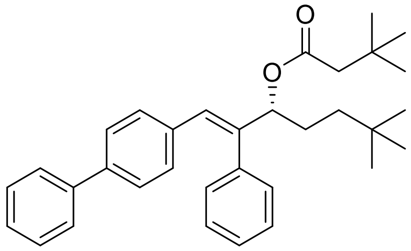

47, 60% yield, 96:4 er

According to General Procedure B, the reaction between diene **S47** (56.4 mg, 0.2 mmol, 1.0 equiv.) and peroxide **O26** (55.2 mg, 0.24 mmol, 1.2 equiv.) afforded product **47** was obtained as a colorless oil (56.2 mg, 60% yield, 96:4 er). $[\alpha]^{28.3}_{D}$ 37.49 (*c* 0.4, $CHCl_3$). ^1H NMR (400 MHz, Chloroform-*d*) δ 7.43 (d, *J* = 7.4 Hz, 2H), 7.33 – 7.18 (m, 10H), 6.91 (d, *J* = 8.2 Hz, 2H), 6.58 (s, 1H), 5.44 (t, *J* = 6.3 Hz, 1H), 2.30 – 2.13 (m, 2H), 1.60 – 1.46 (m, 2H), 1.31 – 1.20 (m, 1H), 1.19 – 1.09 (m, 1H), 0.98 (s, 9H), 0.75 (s, 9H). ^{13}C NMR (101 MHz, Chloroform-*d*) δ 170.66, 140.37, 139.52, 138.41, 137.32, 134.32, 128.69, 128.32, 127.64, 126.98, 126.50, 126.16, 125.78, 125.47, 77.95, 47.22, 38.26, 29.82, 29.02, 28.71, 28.26, 27.22. HRMS (ESI) *m/z* calcd for $[C_{33}H_{40}NaO_2]^+$ ($[M+Na]^+$): 491.2921, found: 491.2919. IR (ν/cm^{-1}) 2957, 2867, 1771, 1647, 1488, 1260, 750, 701. HPLC (IC, 0.46*25 cm, 2 μm, hexane/isopropanol = 99/1, flow 1 mL/min, detection at 254 nm) retention time = 4.070 min (major) and 4.600 min (minor).

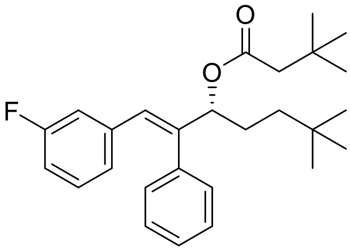

48, 46% yield, 97:3 er

According to General Procedure B, the reaction between diene **S48** (44.8 mg, 0.2 mmol, 1.0 equiv.) and peroxide **O26** (55.2 mg, 0.24 mmol, 1.2 equiv.) afforded product **48** was obtained as a white solid (37.7 mg, 46% yield, 97:3 er). $[\alpha]^{28.3}_{D}$ 38.92 (*c* 0.4, $CHCl_3$). Mp 47.8–49.5 ℃. ^1H NMR (400 MHz, Chloroform-*d*) δ 7.37 – 7.30 (m, 3H), 7.25 – 7.19 (m, 2H), 7.10 – 7.01 (m, 1H), 6.81 – 6.74 (m, 1H), 6.71 (d, *J* = 7.8 Hz, 1H), 6.56 (d, *J* = 13.7 Hz, 2H), 5.49 (t, *J* = 6.3 Hz, 1H), 2.30 – 2.23 (m, 2H), 1.63 – 1.53 (m, 2H), 1.34 – 1.27 (m, 1H) 1.23 – 1.14 (m, 1H), 1.06 (s, 9H), 0.82 (s, 9H). ^{13}C NMR (101 MHz, Chloroform-*d*) δ 170.64, 161.32 (d, *J* = 244.5 Hz), 141.81, 137.58 (d, *J* = 8.0 Hz), 136.75, 128.20, 128.12, 127.69, 126.74, 125.97 (d, *J* = 2.4 Hz), 124.12 (d, *J* = 2.7 Hz), 114.69 (d, *J* = 22.2 Hz), 112.64 (d, *J* = 21.3 Hz), 77.59, 47.16, 38.22, 29.83, 29.01, 28.69, 28.24, 27.17. HRMS (ESI) *m/z* calcd for $[C_{27}H_{35}FNaO_2]^+$ ($[M+Na]^+$): 433.2513, found: 433.2513. IR (*v*/cm^{-1}) 2991, 2963, 1731, 1636, 1458, 1260, 750, 702. HPLC (IC, 0.46*25 cm, 2 μm, hexane/isopropanol = 99.5/0.5, flow 1 mL/min, detection at 254 nm) retention time = 3.965 min (major) and 4.446 min (minor).

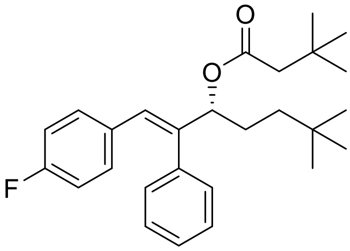

49, 61% yield, 95:5 er

According to General Procedure B, the reaction between diene **S49** (44.8 mg, 0.2 mmol, 1.0 equiv.) and peroxide **O26** (55.2 mg, 0.24 mmol, 1.2 equiv.) afforded product **49** was obtained as a white solid (50.0 mg, 61% yield, 95:5 er). $[\alpha]^{28.3}_{D}$ 33.43 (*c* 0.4, $CHCl_3$). Mp 57.1–58.2 ℃. ^1H NMR (600 MHz, Chloroform-*d*) δ 7.35 – 7.27 (m, 3H), 7.23 – 7.17 (m, 2H), 6.88 – 6.83 (m, 2H), 6.79 – 6.73 (m, 2H), 6.57 (s, 1H), 5.46 (t, *J* = 6.2 Hz, 1H), 2.30 – 2.22 (m, 2H), 1.60 – 1.51 (m, 2H), 1.32 – 1.24 (m, 1H), 1.21 – 1.13 (m, 1H), 1.04 (s, 9H), 0.80 (s, 9H). ^{13}C NMR (151 MHz, Chloroform-*d*) δ 171.83, 161.62 (d, *J* = 246.9 Hz), 141.16 (d, *J* = 2.2 Hz), 138.08, 132.47 (d, *J* = 3.6 Hz), 130.95 (d, *J* = 8.0 Hz), 129.41, 128.78, 127.66, 127.34, 114.88 (d, *J* = 21.3 Hz), 78.95, 48.31, 39.37, 30.96, 30.14, 29.81, 29.36, 28.29. HRMS (ESI) *m/z* calcd for $[C_{27}H_{35}FNaO_2]^+$ ($[M+Na]^+$): 433.2513, found: 433.2513. IR (*v*/cm^{-1}) 2959, 2867, 1732, 1636, 1508, 1260, 750, 702. HPLC (IC, 0.46*25 cm, 2 μm, hexane/isopropanol = 99.8/0.2, flow 1 mL/min, detection at 254 nm) retention time = 6.004 min (major) and 6.892 min (minor).

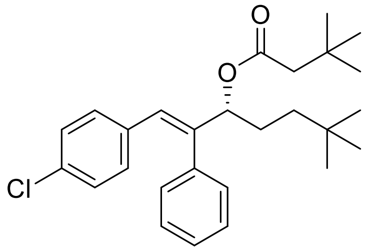

50, 67% yield, 97:3 er

According to General Procedure B, the reaction between diene **S50** (48.0 mg, 0.2 mmol, 1.0 equiv.) and peroxide **O26** (55.2 mg, 0.24 mmol, 1.2 equiv.) afforded product **50** was obtained as a colorless oil (57.1 mg, 67% yield, 97:3 er). $[\alpha]^{28.3}_{D}$ 39.43 (*c* 0.4, $CHCl_3$). ^1H NMR (600 MHz, Chloroform-*d*) δ 7.33 – 7.28 (m, 3H), 7.23 – 7.19 (m, 2H), 7.05 – 7.01 (m, 2H), 6.84 – 6.79 (m, 2H), 6.55 (s, 1H), 5.46 (t, *J* = 6.4 Hz, 1H), 2.30 – 2.23 (m, 2H), 1.60 – 1.56 (m, 1H), 1.55 – 1.51 (m, 1H), 1.34 – 1.27 (m, 1H), 1.20 – 1.16 (m, 1H), 1.04 (s, 9H), 0.80 (s, 9H). ^{13}C NMR (151 MHz, Chloroform-*d*) δ 171.81, 142.26, 137.97, 134.91, 132.54, 130.59, 129.31, 128.81, 128.13, 127.76, 127.09, 78.80, 48.29, 39.36, 30.96, 30.13, 29.80, 29.35, 28.30. HRMS (ESI) *m/z* calcd for $[C_{27}H_{35}ClNaO_2]^+$ ($[M+Na]^+$): 449.2218, found: 449.2216. IR (v/cm^{-1}) 2958, 2868, 1733, 1647, 1490, 1473, 1260, 750, 703. HPLC (IC, 0.46*25 cm, 2 μm, hexane/isopropanol = 99.5/0.5, flow 1 mL/min, detection at 254 nm) retention time = 3.906 min (major) and 4.141 min (minor).

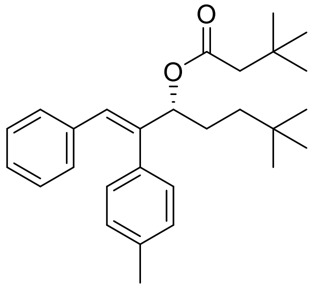

51, 72% yield, 97:3 er

According to General Procedure B, the reaction between diene **S51** (44.0 mg, 0.2 mmol, 1.0 equiv.) and peroxide **O26** (55.2 mg, 0.24 mmol, 1.2 equiv.) afforded product **51** was obtained as a colorless oil (58.5 mg, 72% yield, 97:3 er). $[\alpha]^{28.3}_{D}$ 32.16 (*c* 0.4, $CHCl_3$). ^1H NMR (400 MHz, Chloroform-*d*) δ 7.06 – 7.04 (m, 4H), 7.03 – 6.97 (m, 3H), 6.91 – 6.80 (m, 2H), 6.52 (s, 1H), 5.40 (t, *J* = 6.3 Hz, 1H), 2.28 (s, 3H), 2.23 – 2.14 (m, 2H), 1.57 – 1.43 (m, 2H), 1.28 – 1.19 (m, 1H), 1.16 – 1.06 (m, 1H), 0.98 (s, 9H), 0.74 (s, 9H). ^{13}C NMR (101 MHz, Chloroform-*d*) δ 170.67, 140.25, 136.02, 135.48, 134.16, 128.27, 128.24, 128.13, 127.03, 126.80, 125.68, 77.95, 47.22, 38.24, 29.81, 29.01, 28.70, 28.27, 27.22, 20.26. HRMS (ESI) *m/z* calcd for $[C_{28}H_{38}NaO_2]^+$ ($[M+Na]^+$): 429.2764, found: 429.2760. IR (v/cm^{-1}) 2958, 2867, 1733, 1636, 1473, 1260, 750, 702. HPLC (OD-H, 0.46*25 cm, 2 μm, hexane/isopropanol = 99.5/0.5, flow 1.0 mL/min, detection at 254 nm) retention time = 6.075 min (major) and 7.669 min (minor).

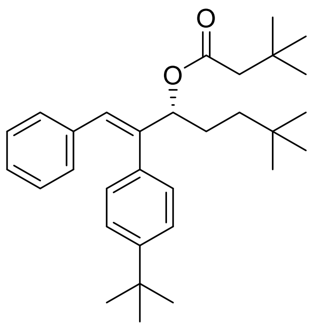

52, 62% yield, 96:4 er

According to General Procedure B, the reaction between diene **S52** (52.4 mg, 0.2 mmol, 1.0 equiv.) and peroxide **O26** (55.2 mg, 0.24 mmol, 1.2 equiv.) afforded product **52** was obtained as a colorless oil (55.6 mg, 62% yield, 96:4 er). $[\alpha]^{28.3}_{D}$ 46.43 (*c* 0.4, $CHCl_3$). ^1H NMR (400 MHz, Chloroform-*d*) δ 7.33 (d, *J* = 8.2 Hz, 2H), 7.16 (d, *J* = 8.2 Hz, 2H), 7.10 – 7.07 (m, 3H), 6.94 – 6.92 (m, 2H), 6.61 (s, 1H), 5.50 (t, *J* = 6.4 Hz, 1H), 2.27 (d, *J* = 3.5 Hz, 2H), 1.67 – 1.50 (m, 2H), 1.34 (s, 9H), 1.28 – 1.15 (m, 2H), 1.06 (s, 9H), 0.83 (s, 9H). ^{13}C NMR (101 MHz, Chloroform-*d*) δ 170.65, 149.32, 140.25, 135.51, 134.07, 128.26, 127.85, 127.19, 126.76, 125.64, 124.37, 77.93, 47.22, 38.22, 33.52, 30.33, 29.80, 29.02, 28.26, 27.25. HRMS (ESI) *m/z* calcd for $[C_{31}H_{44}NaO_2]^+$ ($[M+Na]^+$): 471.3234, found: 471.3230. IR (ν/cm^{-1}) 2958, 2867, 1733, 1636, 1473, 1260, 750, 694. HPLC (OD-H, 0.46*25 cm, 2 μm, hexane/isopropanol = 99.5/0.5, flow 1.0 mL/min, detection at 254 nm) retention time = 4.413 min (major) and 6.430 min (minor).

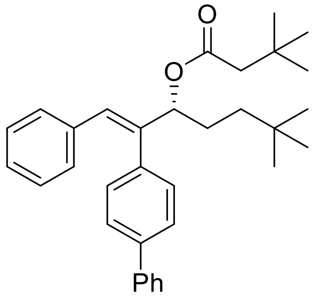

53, 67% yield, 97:3 er

According to General Procedure B, the reaction between diene **S53** (56.4 mg, 0.2 mmol, 1.0 equiv.) and peroxide **O26** (55.2 mg, 0.24 mmol, 1.2 equiv.) afforded product **53** was obtained as a colorless oil (62.7 mg, 67% yield, 97:3 er). $[\alpha]^{28.3}_{D}$ 42.38 (*c* 0.4, $CHCl_3$). ^1H NMR (400 MHz, Chloroform-*d*) δ 7.56 (d, *J* = 7.4 Hz, 2H), 7.50 (d, *J* = 8.1 Hz, 2H), 7.37 – 7.35 (m, 2H), 7.27 – 7.25 (m, 3H), 7.03 – 7.01 (m, 3H), 6.95 – 6.86 (m, 2H), 6.58 (s, 1H), 5.46 (t, *J* = 6.4 Hz, 1H), 2.26 – 2.15 (m, 2H), 1.61 – 1.52 (m, 2H), 1.30 – 1.20 (m, 1H), 1.17 – 1.10 (m, 1H), 0.98 (s, 9H), 0.75 (s, 9H). ^{13}C NMR (101 MHz, Chloroform-*d*) δ 170.68, 139.90, 139.56, 138.97, 136.28, 135.30, 128.77, 128.30, 127.74, 127.55, 126.89, 126.30, 126.13, 125.90, 125.84, 77.87, 47.23, 38.29, 29.83, 29.04, 28.70, 28.27, 27.35. HRMS (ESI) *m/z* calcd for $[C_{33}H_{40}NaO_2]^+$ ($[M+Na]^+$): 491.2921, found: 491.2921. IR (ν/cm^{-1}) 2958, 2867, 1734, 1654, 1474, 1260, 750, 696. HPLC (OD-H, 0.46*25 cm, 2 μm, hexane/isopropanol = 99.5/0.5, flow 1.0 mL/min, detection at 254 nm) retention time = 5.843 min (major) and 8.225 min (minor).

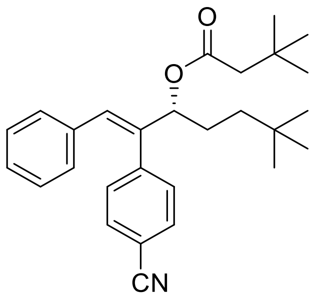

54, 60% yield, 87:13 er

According to General Procedure B, the reaction between diene **S54** (46.2 mg, 0.2 mmol, 1.0 equiv.) and peroxide **O26** (55.2 mg, 0.24 mmol, 1.2 equiv.) afforded product **54** was obtained as a colorless oil (50.0 mg, 60% yield, 87:13 er). $[\alpha]^{28.3}_{D}$ 28.35 (*c* 0.4, $CHCl_3$). ^1H NMR (600 MHz, Chloroform-*d*) δ 7.64 – 7.58 (m, 2H), 7.41 – 7.36 (m, 2H), 7.16 – 7.08 (m, 3H), 6.91 – 6.85 (m, 2H), 6.73 (s, 1H), 5.46 (t, *J* = 6.6 Hz, 1H), 2.29 – 2.22 (m, 2H), 1.57 – 1.50 (m, 2H), 1.31 – 1.28 (m, 1H), 1.18 – 1.12 (m, 1H), 1.04 (s, 9H), 0.81 (s, 9H). ^{13}C NMR (151 MHz, Chloroform-*d*) δ 171.76, 143.70, 139.83, 135.49, 132.47, 130.48, 130.40, 129.39, 128.24, 127.56, 118.94, 111.36, 78.44, 48.24, 39.46, 31.02, 30.14, 29.78, 29.32, 28.62. HRMS (ESI) *m/z* calcd for $[C_{28}H_{35}NNaO_2]^+$ ($[M+Na]^+$): 440.2560, found: 440.2560. IR (v/cm^{-1}) 2958, 2867, 2229, 1734, 1637, 1473, 1260, 750, 697. HPLC (IC, 0.46*25 cm, 2 μm, hexane/isopropanol = 99/1, flow 1 mL/min, detection at 254 nm) retention time = 9.311 min (major) and 10.990 min (minor).

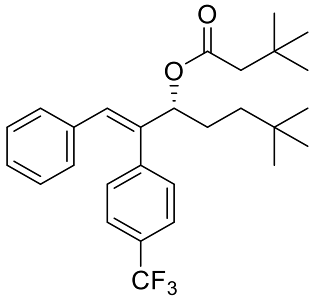

55, 65% yield, 95:5 er

According to General Procedure B, the reaction between diene **S55** (54.8 mg, 0.2 mmol, 1.0 equiv.) and peroxide **O26** (55.2 mg, 0.24 mmol, 1.2 equiv.) afforded product **55** was obtained as a colorless oil (59.8 mg, 65% yield, 95:5 er). $[\alpha]^{28.3}_{D}$ 37.42 (*c* 0.4, $CHCl_3$). ^1H NMR (600 MHz, Chloroform-*d*) δ 7.57 (d, *J* = 8.0 Hz, 2H), 7.37 (d, *J* = 7.9 Hz, 2H), 7.12 – 7.09 (m, 3H), 6.92 – 6.86 (m, 2H), 6.70 (s, 1H), 5.50 – 5.44 (m, 1H), 2.29 – 2.22 (m, 2H), 1.57 – 1.52 (m, 2H), 1.32 – 1.26 (m, 1H), 1.20 – 1.15 (m, 1H), 1.04 (s, 9H), 0.81 (s, 9H). ^{13}C NMR (151 MHz, Chloroform-*d*) δ 171.79, 142.32, 140.06, 135.76, 129.96, 129.84, 129.38, 128.17, 127.35, 125.61 (q, *J* = 4.1 Hz), 124.25 (d, *J* = 272.0 Hz), 78.69, 48.27, 39.43, 30.99, 30.14, 29.79, 29.34, 28.48. HRMS (ESI) *m/z* calcd for $[C_{28}H_{35}F_3NaO_2]^+$ ($[M+Na]^+$): 483.2481, found: 483.2481. IR (v/cm^{-1}) 3005, 2982, 2961, 1734, 1637,1541, 1458, 1260, 750, 697. HPLC (IC, 0.46*25 cm, 2 μm, hexane/isopropanol = 99.8/0.2, flow 0.8 mL/min, detection at 254 nm) retention time = 5.173 min (major) and 5.678 min (minor).

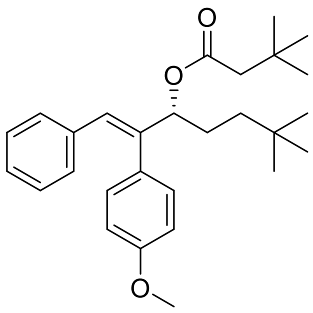

56, 72% yield, 96:4 er

According to General Procedure B, the reaction between diene **S56** (47.2 mg, 0.2 mmol, 1.0 equiv.) and peroxide **O26** (55.2 mg, 0.24 mmol, 1.2 equiv.) afforded product **56** was obtained as a colorless oil (60.8 mg, 72% yield, 96:4 er). $[\alpha]^{28.3}_{D}$ 35.83 (*c* 0.4, $CHCl_3$). ^{1}H NMR (400 MHz, Chloroform-*d*) δ 7.09 (d, *J* = 8.6 Hz, 2H), 7.06 – 6.97 (m, 3H), 6.90 – 6.84 (m, 2H), 6.79 (d, *J* = 8.6 Hz, 2H), 6.51 (s, 1H), 5.40 (t, *J* = 6.4 Hz, 1H), 3.75 (s, 3H), 2.25 – 2.13 (m, 2H), 1.53 – 1.45 (m, 2H), 1.26 – 1.19 (m, 1H), 1.17 – 1.05 (m, 1H), 0.98 (s, 9H), 0.74 (s, 9H). ^{13}C NMR (101 MHz, Chloroform-*d*) δ 170.69, 157.89, 139.90, 135.54, 129.46, 129.39, 128.24, 127.10, 126.84, 125.66, 112.98, 78.01, 54.14, 47.24, 38.27, 29.82, 29.01, 28.70, 28.27, 27.26. HRMS (ESI) *m/z* calcd for $[C_{28}H_{38}NaO_3]^+$ ($[M+Na]^+$): 445.2713, found: 445.2714. IR (*v*/cm^{-1}) 3006, 2956, 2925, 2854, 1734, 1637, 1508,1458, 1260, 750, 697. HPLC (OD-H, 0.46*25 cm, 2 μm, hexane/isopropanol = 99.5/0.5, flow 1.0 mL/min, detection at 254 nm) retention time = 5.609 min (major) and 14.433 min (minor).

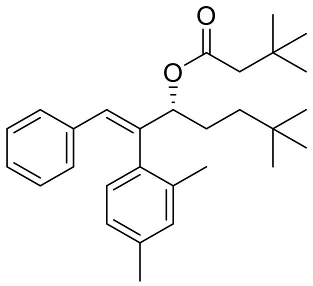

57, 40% yield, 90:10 er

According to General Procedure B, the reaction between diene **S57** (46.8 mg, 0.2 mmol, 1.0 equiv.) and peroxide **O26** (55.2 mg, 0.24 mmol, 1.2 equiv.) afforded product **57** was obtained as a colorless oil (atropisomers, 33.6 mg, 40% yield, 90:10 er). $[\alpha]^{28.3}_{D}$ 32.44 (*c* 0.4, $CHCl_3$). ^{1}H NMR (400 MHz, Chloroform-*d*) δ 7.24 – 7.14 (m, 1H), 7.04 – 6.85 (m, 5H), 6.82 – 6.76 (m, 2H), 6.63 – 6.48 (m, 1H), 5.48 – 5.31 (m, 1H), 2.27 (s, 3H), 2.25 – 2.16 (m, 2H), 2.12 – 1.82 (m, 3H), 1.68 – 1.54 (m, 1H), 1.52 – 1.43 (m, 1H), 1.32 – 1.21 (m, 1H), 1.19 – 1.07 (m, 1H), 1.05 – 0.95 (m, 9H), 0.83 – 0.71 (m, 9H). ^{13}C NMR (101 MHz, Chloroform-*d*) δ 170.78, 170.47, 139.30, 138.97, 136.13, 136.03, 135.83, 135.59, 135.11, 134.66, 133.63, 130.38, 130.14, 128.25, 128.15, 127.62, 127.39, 126.98, 126.55, 126.27, 125.92, 125.81, 125.73, 78.42, 47.27, 47.12, 38.73, 37.92, 29.84, 29.74, 29.07, 29.02, 28.74, 28.68, 28.31, 28.29, 27.29, 26.88, 20.18, 20.16, 18.41. HRMS (ESI) *m/z* calcd for $[C_{29}H_{40}NaO_2]^+$ ($[M+Na]^+$): 443.2921, found: 443.2920. IR (*v*/cm^{-1}) 3006, 2957, 2867, 1734, 1637, 1508, 1364, 1260, 750, 695. HPLC (OD-H, 0.46*25 cm, 2 μm, hexane/isopropanol = 99.5/0.5, flow 0.8 mL/min, detection at 254 nm) retention time = 4.032 min (major) and 5.744 min (minor).

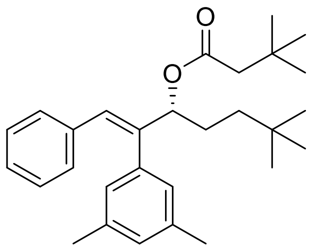

58, 50% yield, 95:5 er

According to General Procedure B, the reaction between diene **S58** (46.8 mg, 0.2 mmol, 1.0 equiv.) and peroxide **O26** (55.2 mg, 0.24 mmol, 1.2 equiv.) afforded product **58** was obtained as a colorless oil (42.0 mg, 50% yield, 95:5 er). $[\alpha]^{28.3}_{D}$ 39.53 (*c* 0.4, $CHCl_3$). ^{1}H NMR (400 MHz, Chloroform-*d*) δ 7.31 – 7.15 (m, 1H), 7.04 – 6.97 (m, 3H), 6.90 – 6.84 (m, 3H), 6.83 – 6.72 (m, 2H), 6.48 (s, 1H), 5.39 (t, *J* = 6.3 Hz, 1H), 2.22 – 2.14 (m, 8H), 1.58 – 1.44 (m, 2H), 1.26 – 1.11 (m, 2H), 0.99 (s, 9H), 0.75 (s, 9H). ^{13}C NMR (101 MHz, Chloroform-*d*) δ 170.66, 140.28, 137.08, 136.95, 135.40, 128.22, 128.04, 126.80, 126.63, 125.81, 125.70, 77.94, 47.26, 38.08, 29.79, 29.03, 28.72, 28.28, 27.03, 20.28. HRMS (ESI) *m/z* calcd for $[C_{29}H_{40}NaO_2]^+$ ($[M+Na]^+$): 443.2921, found: 443.2922. IR (*v*/cm^{-1}) 3006, 2959, 2867, 1734, 1637, 1508, 1458, 1260, 750, 705. HPLC (IC, 0.46*25 cm, 2 μm, hexane/isopropanol = 99.8/0.2, flow 0.8 mL/min, detection at 254 nm) retention time = 4.425 min (major) and 5.188 min (minor).

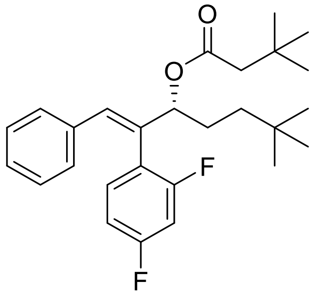

59, 49% yield, 97:3 er

According to General Procedure B, the reaction between diene **S59** (48.4 mg, 0.2 mmol, 1.0 equiv.) and peroxide **O26** (55.2 mg, 0.24 mmol, 1.2 equiv.) afforded product **59** was obtained as a colorless oil (41.9 mg, 49% yield, 97:3 er). $[\alpha]^{28.3}_{D}$ 42.47 (*c* 0.4, $CHCl_3$). ^{1}H NMR (600 MHz, Chloroform-*d*) δ 7.29 – 7.20 (m, 1H), 7.15 – 7.10 (m, 3H), 6.95 – 6.91 (m, 2H), 6.90 – 6.86 (m, 1H), 6.82 – 6.76 (m, 2H), 5.47 (t, *J* = 6.5 Hz, 1H), 2.27 – 2.22 (m, 2H), 1.64 – 1.52 (m, 2H), 1.32 – 1.25 (m, 1H), 1.23 – 1.18 (m, 1H), 1.03 (s, 9H), 0.82 (s, 9H). ^{13}C NMR (151 MHz, Chloroform-*d*) δ 171.75, 162.67 (dd, *J* = 249.1, 11.8 Hz), 160.01 (dd, *J* = 249.3, 12.0 Hz), 136.06, 133.57, 132.34 (dd, *J* = 9.7, 5.3 Hz), 128.67, 128.22, 127.51, 115.36, 111.76 (dd, *J* = 21.2, 4.0 Hz), 104.43 (dd, *J* = 26.8, 25.0 Hz), 100.00, 78.58, 48.21, 39.33, 30.90, 30.13, 29.77, 29.35, 28.33. HRMS (ESI) *m/z* calcd for $[C_{27}H_{34}F_2NaO_2]^+$ ($[M+Na]^+$): 451.2419, found: 451.2419. IR (*v*/cm^{-1}) 3006, 2989, 1734, 1637, 1507, 1458, 1260, 750, 697. HPLC (IC, 0.46*25 cm, 2 μm, hexane/isopropanol = 99.8/0.2, flow 0.8 mL/min, detection at 254 nm) retention time = 6.122 min (major) and 7.858 min (minor).

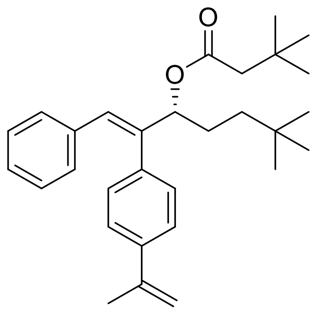

60, 45% yield, 95:5 er

According to General Procedure B, the reaction between diene **S60** (49.2 mg, 0.2 mmol, 1.0 equiv.) and peroxide **O26** (55.2 mg, 0.24 mmol, 1.2 equiv.) afforded product **60** was obtained as a colorless oil (38.9 mg, 45% yield, 95:5 er). $[\alpha]^{28.3}_{D}$ 49.14 (*c* 0.4, $CHCl_3$). ^1H NMR (600 MHz, Chloroform-*d*) δ 7.45 – 7.41 (m, 2H), 7.22 – 7.18 (m, 2H), 7.11 – 7.06 (m, 3H), 6.96 – 6.92 (m, 2H), 6.61 (s, 1H), 5.48 (t, *J* = 6.2 Hz, 1H), 5.44 – 5.41 (m, 1H), 5.11 – 5.07 (m, 1H), 2.30 – 2.22 (m, 2H), 2.17 – 2.14 (m, 3H), 1.62 – 1.57 (m, 1H), 1.57 – 1.52 (m, 1H), 1.32 – 1.26 (m, 1H), 1.21 – 1.14 (m, 1H), 1.04 (s, 9H), 0.80 (s, 9H). ^{13}C NMR (151 MHz, Chloroform-*d*) δ 171.83, 142.79, 141.06, 139.95, 137.49, 136.43, 129.40, 129.30, 128.51, 128.01, 127.99, 126.93, 125.69, 112.41, 78.98, 48.33, 39.38, 30.96, 30.15, 29.82, 29.39, 28.40, 21.81. HRMS (ESI) *m/z* calcd for $[C_{30}H_{40}NaO_2]^+$ ($[M+Na]^+$): 455.2921, found: 455.2925. IR (v/cm^{-1}) 3006, 2989, 2961, 1734, 1717, 1637, 1507, 1458, 1260, 750, 697. HPLC (IC, 0.46*25 cm, 2 μm, hexane/isopropanol = 99.8/0.2, flow 0.8 mL/min, detection at 254 nm) retention time = 6.426 min (major) and 8.306 min (minor).

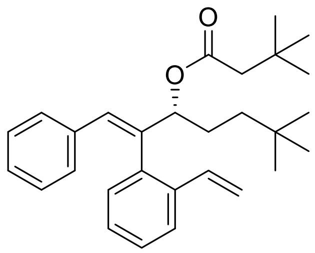

61, 25% yield, 96:4 er

According to General Procedure B, the reaction between diene **S61** (46.4 mg, 0.2 mmol, 1.0 equiv.) and peroxide **O26** (55.2 mg, 0.24 mmol, 1.2 equiv.) afforded product **61** was obtained as a colorless oil (20.9 mg, 25% yield, 96:4 er). $[\alpha]^{28.3}_{D}$ 53.42 (*c* 0.4, $CHCl_3$). ^1H NMR (400 MHz, Chloroform-*d*) δ 7.38 (d, *J* = 8.1 Hz, 2H), 7.22 (d, *J* = 8.1 Hz, 2H), 7.11 – 7.06 (m, 3H), 6.98 – 6.90 (m, 2H), 6.73 (dd, *J* = 17.6, 10.9 Hz, 1H), 6.63 (s, 1H), 5.77 (d, *J* = 17.6 Hz, 1H), 5.49 (t, *J* = 6.4 Hz, 1H), 5.26 (d, *J* = 10.9 Hz, 1H), 2.38 – 2.16 (m, 2H), 1.66 – 1.51 (m, 2H), 1.35 – 1.27 (m, 1H), 1.24 – 1.14 (m, 1H), 1.06 (s, 9H), 0.82 (s, 9H). ^{13}C NMR (101 MHz, Chloroform-*d*) δ 170.67, 139.94, 136.84, 135.54, 135.28, 128.53, 128.28, 127.45, 126.88, 125.83, 125.43, 112.78, 77.82, 47.22, 38.28, 29.83, 29.02, 28.70, 28.26, 27.31. HRMS (ESI) *m/z* calcd for $[C_{29}H_{38}NaO_2]^+$ ($[M+Na]^+$): 441.2764, found: 441.2760. IR (v/cm^{-1}) 3006, 2989, 2961, 1734, 1718, 1637, 1507, 1458, 1260, 750, 697. HPLC (OD-H, 0.46*25 cm, 2 μm, hexane/isopropanol = 99.9/0.1, flow 1.0 mL/min, detection at 254 nm) retention time = 7.355 min (major) and 15.245 min (minor).

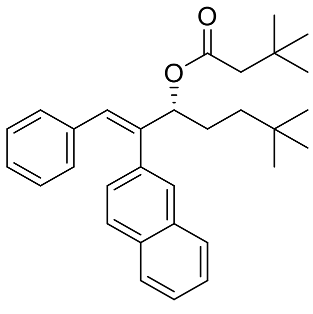

62, 68% yield, 96:4 er

According to General Procedure B, the reaction between diene **S62** (51.2 mg, 0.2 mmol, 1.0 equiv.) and peroxide **O26** (55.2 mg, 0.24 mmol, 1.2 equiv.) afforded product **62** was obtained as a colorless oil (60.1 mg, 68% yield, 96:4 er). $[\alpha]^{28.3}_{D}$ 42.04 (*c* 0.4, $CHCl_3$). ^1H NMR (400 MHz, Chloroform-*d*) δ 7.88 – 7.77 (m, 4H), 7.54 – 7.45 (m, 2H), 7.36 – 7.30 (m, 1H), 7.12 – 7.03 (m, 3H), 6.96 (dd, *J* = 6.6, 2.9 Hz, 2H), 6.73 (s, 1H), 5.62 (t, *J* = 6.3 Hz, 1H), 2.39 – 2.24 (m, 2H), 1.63 (dd, *J* = 13.1, 7.1 Hz, 2H), 1.41 – 1.31 (m, 1H), 1.27 – 1.19 (m, 2H), 1.09 (s, 9H), 0.82 (s, 9H). ^{13}C NMR (101 MHz, Chloroform-*d*) δ 170.73, 140.07, 135.21, 134.90, 132.53, 131.65, 128.31, 127.49, 127.12, 127.08, 126.95, 126.89, 126.82, 126.66, 125.86, 124.94, 78.03, 47.26, 38.28, 29.85, 29.02, 28.72, 28.26, 27.36. HRMS (ESI) *m/z* calcd for $[C_{31}H_{38}NaO_2]^+$ ($[M+Na]^+$): 465.2764, found: 465.2763. IR (ν/cm^{-1}) 3006, 2989, 2957, 1733, 1637, 1507, 1458, 1260, 750, 697. HPLC (OD-H, 0.46*25 cm, 2 μm, hexane/isopropanol = 99.5/0.5, flow 1.0 mL/min, detection at 254 nm) retention time = 5.116 min (major) and 7.053 min (minor).

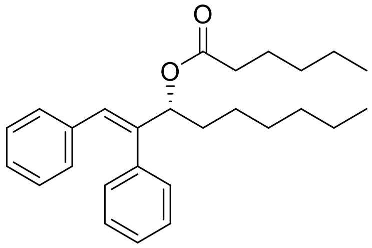

63, 74% yield, 96:4 er

According to General Procedure B, the reaction between diene **S2** (41.2 mg, 0.2 mmol, 1.0 equiv.) and peroxide **O23** (55.2 mg, 0.24 mmol, 1.2 equiv.) afforded product **63** was obtained as a colorless oil (58.0 mg, 74% yield, 96:4 er). $[\alpha]^{28.3}_{D}$ 37.88 (*c* 0.4, $CHCl_3$). ^1H NMR (600 MHz, Chloroform-*d*) δ 7.34 – 7.28 (m, 3H), 7.23 – 7.21 (m, 2H), 7.08 – 7.04 (m, 3H), 6.91 – 6.88 (m, 2H), 6.59 (s, 1H), 5.51 (t, *J* = 6.3 Hz, 1H), 2.35 (td, *J* = 7.4, 1.9 Hz, 2H), 1.66 – 1.55 (m, 4H), 1.31 – 1.17 (m, 12H), 0.89 – 0.81 (m, 6H). ^{13}C NMR (151 MHz, Chloroform-*d*) δ 173.36, 141.66, 138.41, 136.40, 129.42, 129.37, 128.70, 127.95, 127.90, 127.54, 126.89, 78.44, 34.76, 33.39, 31.79, 31.42, 29.05, 25.62, 24.83, 22.65, 22.47, 14.15, 14.02. HRMS (ESI) *m/z* calcd for $[C_{27}H_{36}NaO_2]^+$ ($[M+Na]^+$): 415.2608, found: 415.2608. IR (ν/cm^{-1}) 3007, 2990, 2924, 2853, 1734, 1637, 1507, 1458, 1260, 750, 697. HPLC (OD-H, 0.46*25 cm, 2 μm, hexane/isopropanol = 99.5/0.5, flow 1.0 mL/min, detection at 254 nm) retention time = 5.595 min (major) and 6.690 min (minor).

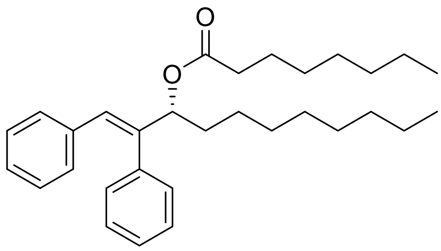

64, 71% yield, 95:5 er

According to General Procedure B, the reaction between diene **S2** (41.2 mg, 0.2 mmol, 1.0 equiv.) and peroxide **O22** (68.6 mg, 0.24 mmol, 1.2 equiv.) afforded product **64** was obtained as a colorless oil (63.6 mg, 71% yield, 95:5 er). $[\alpha]^{28.3}_{D}$ 35.18 (*c* 0.4, $CHCl_3$). ^1H NMR (600 MHz, Chloroform-*d*) δ 7.33 – 7.28 (m, 3H), 7.23 – 7.21 (m, 2H), 7.08 – 7.05 (m, 2H), 6.91 – 6.88 (m, 2H), 6.59 (s, 1H), 5.55 – 5.47 (m, 1H), 2.35 (td, *J* = 7.4, 1.5 Hz, 2H), 1.66 – 1.57 (m, 4H), 1.32 – 1.19 (m, 20H), 0.87 – 0.82 (m, 6H). ^{13}C NMR (151 MHz, Chloroform-*d*) δ 173.36, 141.68, 138.42, 136.41, 129.42, 129.37, 128.70, 127.95, 127.88, 127.54, 126.89, 78.43, 34.81, 33.39, 31.92, 31.77, 29.55, 29.38, 29.30, 29.23, 29.10, 25.67, 25.16, 22.75, 22.71, 14.22, 14.18. HRMS (ESI) *m/z* calcd for $[C_{31}H_{44}NaO_2]^+$ ($[M+Na]^+$): 471.3234, found: 471.3234. IR (*v*/cm^{-1}) 2958, 2927, 2856, 1735, 1637, 1507, 1458, 1260, 750, 702. HPLC (OD-H, 0.46*25 cm, 2 μm, hexane/isopropanol = 100/0, flow 1.0 mL/min, detection at 254 nm) retention time = 10.318 min (major) and 22.103 min (minor).

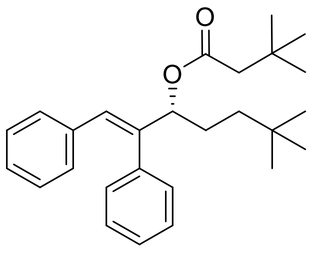

65, 82% yield, 97:3 er

According to General Procedure B, the reaction between diene **S2** (41.2 mg, 0.2 mmol, 1.0 equiv.) and peroxide **O26** (55.2 mg, 0.24 mmol, 1.2 equiv.) afforded product **65** was obtained as a white solid (62.3 mg, 82% yield, 97:3 er). $[\alpha]^{28.3}_{D}$ 44.14 (*c* 0.4, $CHCl_3$). Mp 48.4–49.9 ℃. ^1H NMR (400 MHz, Chloroform-d) δ 7.29 – 7.22 (m, 3H), 7.20 – 7.15 (m, 2H), 7.03 – 6.99 (m, 3H), 6.87 – 6.81 (m, 2H), 6.55 (s, 1H), 5.43 (t, *J* = 6.5 Hz, 1H), 2.24 – 2.15 (m, 2H), 1.58 – 1.43 (m, 2H), 1.29 – 1.20 (m, 1H), 1.16 – 1.08 (m, 1H), 0.98 (s, 9H), 0.74 (s, 9H). ^{13}C NMR (101 MHz, Chloroform-*d*) δ 170.67, 140.22, 137.25, 135.29, 128.32, 128.27, 127.54, 127.37, 126.82, 126.41, 125.79, 77.87, 47.21, 38.23, 29.82, 29.01, 28.70, 28.25, 27.20. HRMS (ESI) *m/z* calcd for $[C_{27}H_{36}NaO_2]^+$ ($[M+Na]^+$): 415.2608, found: 415.2608. IR (*v*/cm^{-1}) 3006, 2959, 2867, 1734, 1637, 1508, 1458, 1260, 750, 705. HPLC (OD-H, 0.46*25 cm, 2 μm, hexane/isopropanol = 100/0, flow 1.0 mL/min, detection at 254 nm) retention time = 7.550 min (major) and 21.363 min (minor).

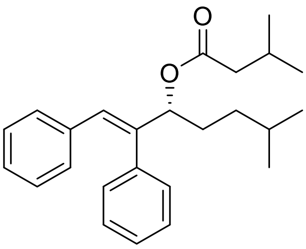

66, 68% yield, 97:3 er

According to General Procedure B, the reaction between diene **S2** (41.2 mg, 0.2 mmol, 1.0 equiv.) and peroxide **O25** (48.5 mg, 0.24 mmol, 1.2 equiv.) afforded product **66** was obtained as a colorless oil (49.5 mg, 68% yield, 97:3 er). $[\alpha]^{28.3}_{D}$ 53.72 (*c* 0.4, $CHCl_3$). ^1H NMR (600 MHz, Chloroform-*d*) δ 7.34 – 7.29 (m, 3H), 7.25 – 7.23 (m, 2H), 7.10 – 7.05 (m, 3H), 6.92 – 6.89 (m, 2H), 6.61 (s, 1H), 5.53 – 5.50 (m, 1H), 2.26 – 2.24 (m, 2H), 2.18 – 2.10 (m, 1H), 1.64 – 1.58 (m, 2H), 1.53 – 1.44 (m, 1H), 1.30 – 1.24 (m, 1H), 1.23 – 1.17 (m, 1H), 0.96 (d, *J* = 6.6 Hz, 6H), 0.82 (dd, *J* = 6.5, 5.3 Hz, 6H). ^{13}C NMR (151 MHz, Chloroform-*d*) δ 172.62, 141.56, 138.40, 136.40, 129.42, 129.38, 128.71, 128.11, 127.97, 127.55, 126.92, 78.58, 43.91, 34.60, 31.15, 27.88, 25.87, 22.72, 22.62, 22.53. HRMS (ESI) *m/z* calcd for $[C_{25}H_{32}NaO_2]^+$ ($[M+Na]^+$): 387.2295, found: 387.2293. IR (ν/cm^{-1}) 2958, 2870, 1734, 1637, 1507, 1458, 1260, 750, 702. HPLC (OD-H, 0.46*25 cm, 2 µm, hexane/isopropanol = 100/0, flow 1.0 mL/min, detection at 254 nm) retention time = 8.038 min (major) and 10.920 min (minor).

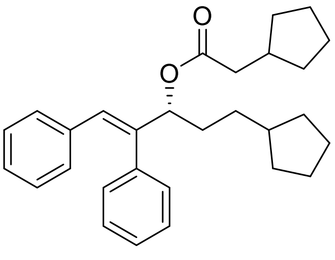

67, 66% yield, 96:4 er

According to General Procedure B, the reaction between diene **S2** (41.2 mg, 0.2 mmol, 1.0 equiv.) and peroxide **O28** (61.0 mg, 0.24 mmol, 1.2 equiv.) afforded product **67** was obtained as a colorless oil (54.9 mg, 66% yield, 96:4 er). $[\alpha]^{28.3}_{D}$ 38.70 (*c* 0.4, $CHCl_3$). ^1H NMR (400 MHz, Chloroform-*d*) δ 7.36 – 7.28 (m, 3H), 7.24 – 7.22 (m, 2H), 7.09 – 7.07 (m, 3H), 6.92 – 6.90 (m, 2H), 6.60 (s, 1H), 5.52 (t, *J* = 6.5 Hz, 1H), 2.37 (d, *J* = 7.8 Hz, 2H), 2.31 – 2.18 (m, 1H), 1.90 – 1.77 (m, 2H), 1.70 – 1.45 (m, 13H), 1.41 – 1.29 (m, 2H), 1.22 – 1.11 (m, 2H), 1.08 – 0.95 (m, 2H). ^{13}C NMR (101 MHz, Chloroform-*d*) δ 172.74, 141.48, 138.35, 136.35, 129.34, 129.29, 128.60, 127.93, 127.86, 127.44, 126.81, 78.45, 40.84, 39.82, 36.57, 32.69, 32.56, 32.55, 32.50, 32.39, 31.80, 25.12, 25.01. HRMS (ESI) m/z calcd for $[C_{29}H_{36}NaO_2]^+$ ($[M+Na]^+$): 439.2608, found: 439.2607. IR (ν/cm-1) 3007, 2990, 2866, 1734, 1637, 1507, 1458, 1260, 750, 703. HPLC (OD-H, 0.46*25 cm, 2 µm, hexane/isopropanol = 99.5/0.5, flow 1.0 mL/min, detection at 254 nm) retention time = 5.943 min (major) and 7.259 min (minor).

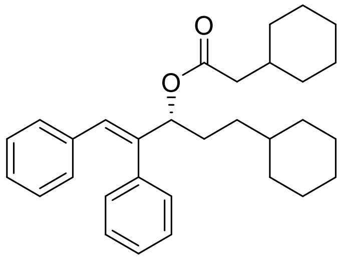

68, 67% yield, 96:4 er

According to General Procedure B, the reaction between diene **S2** (41.2 mg, 0.2 mmol, 1.0 equiv.) and peroxide **O24** (169.2 mg, 0.6 mmol, 3.0 equiv.) afforded product **68** was obtained as a colorless oil (59.5 mg, 67% yield, 96:4 er). $[\alpha]^{28.3}_{D}$ 33.47 (*c* 0.4, $CHCl_3$). ^1H NMR (600 MHz, Chloroform-*d*) δ 7.33 – 7.27 (m, 3H), 7.23 – 7.20 (m, 2H), 7.08 – 7.05 (m, 3H), 6.91 – 6.87 (m, 2H), 6.58 (s, 1H), 5.52 – 5.45 (m, 1H), 2.23 (d, *J* = 7.1 Hz, 2H), 1.74 – 1.57 (m, 12H), 1.29 – 1.04 (m, 10H), 1.00 – 0.90 (m, 2H), 0.86 – 0.76 (m, 2H). ^{13}C NMR (151 MHz, Chloroform-*d*) δ 172.61, 141.57, 138.41, 136.41, 129.42, 129.36, 128.69, 128.04, 127.94, 127.52, 126.89, 78.66, 42.65, 37.51, 35.06, 33.45, 33.30, 33.22, 33.17, 33.09, 30.69, 26.70, 26.41, 26.39, 26.24, 26.13, 26.11. HRMS (ESI) *m/z* calcd for $[C_{31}H_{40}NaO_2]^+$ ($[M+Na]^+$): 467.2921, found: 467.2921. IR (*v*/cm^{-1}) 3024, 2923, 2851, 1734, 1637, 1507, 1458, 1260, 750, 702. HPLC (IC, 0.46*25 cm, 2 μm, hexane/isopropanol = 99/1, flow 1 mL/min, detection at 254 nm) retention time = 4.092 min (major) and 4.779 min (minor).

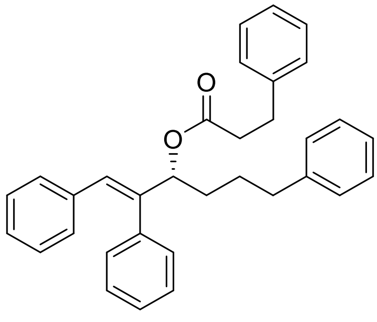

69, 56% yield, 96:4 er

According to General Procedure B, the reaction between diene **S2** (41.2 mg, 0.2 mmol, 1.0 equiv.) and peroxide **O69** (71.5 mg, 0.24 mmol, 1.2 equiv.) afforded product **69** was obtained as a colorless oil (51.5 mg, 56% yield, 96:4 er). $[\alpha]^{28.3}_{D}$ 39.68 (*c* 0.4, $CHCl_3$). ^1H NMR (600 MHz, Chloroform-*d*) δ 7.33 – 7.30 (m, 3H), 7.27 – 7.25 (m, 2H), 7.24 – 7.22 (m, 2H), 7.21 – 7.15 (m, 7H), 7.12 – 7.07 (m, 5H), 6.91 – 6.87 (m, 2H), 6.57 (s, 1H), 5.58 (t, *J* = 5.9 Hz, 1H), 2.98 (t, *J* = 7.8 Hz, 2H), 2.72 – 2.67 (m, 2H), 2.59 – 2.52 (m, 2H), 1.69 – 1.60 (m, 4H). ^{13}C NMR (151 MHz, Chloroform-*d*) δ 172.36, 142.13, 141.08, 140.55, 138.17, 136.25, 129.43, 129.41, 128.78, 128.61, 128.50, 128.42, 128.40, 127.98, 127.61, 127.03, 126.38, 125.90, 78.56, 36.28, 35.57, 32.80, 31.06, 27.44. HRMS (ESI) *m/z* calcd for $[C_{33}H_{32}NaO_2]^+$ ($[M+Na]^+$): 483.2295, found: 483.2294. IR (*v*/cm^{-1}) 3026, 2990, 2925, 2854, 1734, 1637, 1507, 1458, 1260, 750, 699. HPLC (OD-H, 0.46*25 cm, 2 μm, hexane/isopropanol = 98/2, flow 1.0 mL/min, detection at 254 nm) retention time = 14.278 min (minor) and 15.861 min (major).

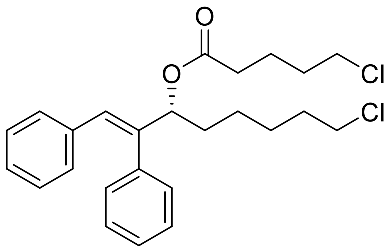

70, 68% yield, 95:5 er

According to General Procedure B, the reaction between diene **S2** (41.2 mg, 0.2 mmol, 1.0 equiv.) and peroxide **O34** (202.8 mg, 0.6 mmol, 3.0 equiv.) afforded product **70** was obtained as a colorless oil (58.8 mg, 68% yield, 95:5 er). $[\alpha]^{28.3}_{D}$ 35.42 (*c* 0.4, $CHCl_3$). ^1H NMR (400 MHz, Chloroform-*d*) δ 7.34 – 7.32 (m, 3H), 7.24 – 7.19 (m, 2H), 7.12 – 7.04 (m, 3H), 6.93 – 6.87 (m, 2H), 6.60 (s, 1H), 5.54 (t, *J* = 6.5 Hz, 1H), 3.57 – 3.46 (m, 4H), 2.47 – 2.33 (m, 2H), 1.83 – 1.77 (m, 4H), 1.76 – 1.69 (m, 2H), 1.68 – 1.61 (m, 2H), 1.47 – 1.31 (m, 4H). ^{13}C NMR (101 MHz, Chloroform-*d*) δ 171.45, 140.11, 137.06, 135.08, 128.25, 127.67, 127.14, 126.88, 126.56, 125.93, 77.39, 43.88, 43.44, 32.71, 32.07, 31.34, 30.79, 25.49, 23.85, 21.26. HRMS (ESI) *m/z* calcd for $[C_{25}H_{30}Cl_2NaO_2]^+$ ($[M+Na]^+$): 455.1515, found: 455.1515. IR (*v*/cm^{-1}) 2990, 2927, 2855, 1734, 1637, 1507, 1458, 1260, 750, 702. HPLC (IC, 0.46*25 cm, 2 μm, hexane/isopropanol = 99/1, flow 1 mL/min, detection at 254 nm) retention time = 7.508 min (major) and 10.219 min (minor).

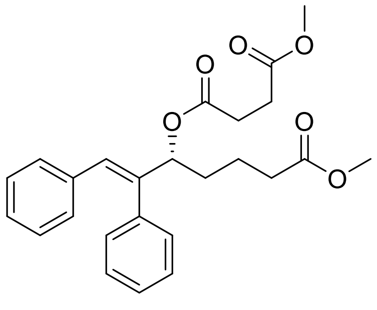

71, 59% yield, 98:2 er

According to General Procedure B, the reaction between diene **S2** (41.2 mg, 0.2 mmol, 1.0 equiv.) and peroxide **O32** (62.9 mg, 0.24 mmol, 1.2 equiv.) afforded product **71** was obtained as a colorless oil (50.0 mg, 59% yield, 98:2 er). $[\alpha]^{28.3}_{D}$ 49.02 (*c* 0.4, $CHCl_3$). ^1H NMR (600 MHz, Chloroform-*d*) δ 7.34 – 7.29 (m, 3H), 7.22 – 7.19 (m, 2H), 7.08 – 7.04 (m, 3H), 6.90 – 6.88 (m, 2H), 6.59 (s, 1H), 5.53 (t, *J* = 7.1 Hz, 1H), 3.65 (s, 3H), 3.62 (s, 3H), 2.75 – 2.67 (m, 2H), 2.65 (d, *J* = 8.1 Hz, 2H), 2.28 (t, *J* = 7.2 Hz, 2H), 1.68 – 1.60 (m, 4H). ^{13}C NMR (151 MHz, Chloroform-*d*) δ 173.77, 172.77, 171.67, 140.59, 137.99, 136.12, 129.41, 129.37, 128.83, 128.40, 127.98, 127.71, 127.07, 78.53, 51.99, 51.66, 33.66, 32.54, 29.52, 29.01, 20.97. HRMS (ESI) *m/z* calcd for $[C_{25}H_{28}NaO_6]^+$ ($[M+Na]^+$): 447.1778, found: 447.1778. IR (*v*/cm^{-1}) 2981, 2924, 2852, 1735, 1637, 1507, 1458, 1260, 750, 702. HPLC (OD-H, 0.46*25 cm, 2 μm, hexane/isopropanol = 97/3, flow 1.0 mL/min, detection at 254 nm) retention time = 12.832 min (major) and 14.543 min (minor).

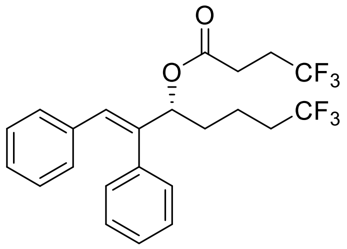

72, 64% yield, 97:3 er

According to General Procedure B, the reaction between diene **S2** (41.2 mg, 0.2 mmol, 1.0 equiv.) and peroxide **O30** (67.7 mg, 0.24 mmol, 1.2 equiv.) afforded product **72** was obtained as a colorless oil (56.8 mg, 64% yield, 97:3 er). $[\alpha]^{28.3}_{D}$ 28.68 (*c* 0.4, $CHCl_3$). ^{1}H NMR (600 MHz, Chloroform-*d*) δ 7.36 – 7.31 (m, 3H), 7.21 – 7.19 (m, 2H), 7.10 – 7.07 (m, 3H), 6.93 – 6.87 (m, 2H), 6.61 (s, 1H), 5.56 (t, *J* = 7.2 Hz, 1H), 2.72 – 2.59 (m, 2H), 2.52 – 2.42 (m, 2H), 2.09 – 1.99 (m, 2H), 1.74 – 1.58 (m, 4H). ^{13}C NMR (151 MHz, Chloroform-*d*) δ 170.36, 140.10, 137.67, 135.80, 129.42, 129.26, 128.98, 128.92, 128.08, 127.95, 127.33, 78.73, 33.41 (q, *J* = 28.7 Hz), 32.19, 29.37 (q, *J* = 30.2 Hz), 27.46, 18.19. HRMS (ESI) *m/z* calcd for $[C_{22}H_{23}F_6NaO_2]^+$ ($[M+Na]^+$): 467.1416, found: 467.1418. IR (*v*/cm^{-1}) 2962, 2923, 2851, 1736, 1638, 1507, 1458, 1260, 750, 701. HPLC (OD-H, 0.46*25 cm, 2 μm, hexane/isopropanol = 99.5/0.5, flow 1.0 mL/min, detection at 254 nm) retention time = 9.441 min (major) and 10.334 min (minor).

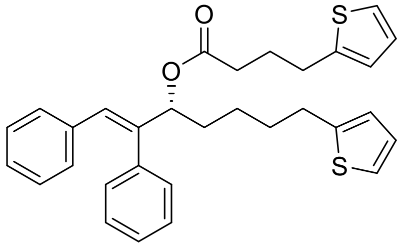

73, 58% yield, 97:3 er

According to General Procedure B, the reaction between diene **S2** (41.2 mg, 0.2 mmol, 1.0 equiv.) and peroxide **O36** (71.5 mg, 0.24 mmol, 1.2 equiv.) afforded product **73** was obtained as a colorless oil (58.0 mg, 58% yield, 97:3 er). $[\alpha]^{28.3}_{D}$ 35.01(c 0.4, $CHCl_3$). ^{1}H NMR (600 MHz, Chloroform-*d*) δ 7.34 – 7.30 (m, 3H), 7.23 – 7.21 (m, 2H), 7.12 (dd, *J* = 5.1, 1.2 Hz, 1H), 7.10 – 7.07 (m, 4H), 6.92 – 6.87 (m, 4H), 6.77 (s, 1H), 6.73 (s, 1H), 6.61 (s, 1H), 5.55 (t, *J* = 7.5 Hz, 1H), 2.88 (t, *J* = 7.7 Hz, 2H), 2.78 (t, *J* = 7.7 Hz, 2H), 2.45 – 2.39 (m, 2H), 2.02 (q, *J* = 7.4 Hz, 2H), 1.66 (p, *J* = 7.3 Hz, 4H), 1.51 – 1.36 (m, 2H). ^{13}C NMR (151 MHz, Chloroform-*d*) δ 172.70, 145.31, 144.19, 141.34, 138.24, 136.28, 129.42, 128.81, 128.18, 128.00, 127.67, 127.03, 126.90, 126.77, 124.69, 124.18, 123.37, 122.99, 78.49, 33.73, 33.06, 31.48, 29.80, 29.20, 26.92, 25.12. HRMS (ESI) *m/z* calcd for $[C_{31}H_{32}NaO_2S_2]^+$ ($[M+Na]^+$): 523.1736, found: 523.1731. IR (*v*/cm^{-1}) 3056, 3024, 2990, 2930, 2855, 1733, 1647, 1492, 1442, 1260, 751, 695. HPLC (IC, 0.46*25 cm, 2 μm, hexane/isopropanol = 99/1, flow 1.0 mL/min, detection at 254 nm) retention time = 7.863 min (major) and 11.381 min (minor).

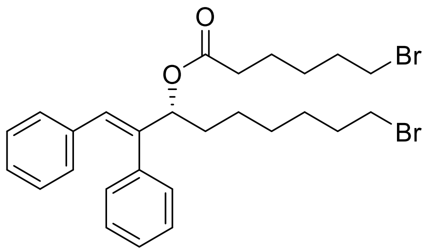

74, 71% yield, 96:4 er

According to General Procedure B, the reaction between diene **S2** (41.2 mg, 0.2 mmol, 1.0 equiv.) and peroxide **O35** (231.6 mg, 0.6 mmol, 3.0 equiv.) afforded product **74** was obtained as a colorless oil (77.8 mg, 71% yield, 96:4 er). $[\alpha]^{28.3}_{D}$ 31.18 (*c* 0.4, $CHCl_3$). ^1H NMR (400 MHz, Chloroform-*d*) δ 7.27 – 7.25 (m, 3H), 7.18 – 7.13 (m, 2H), 7.04 – 6.98 (m, 3H), 6.84 – 6.82 (m, 2H), 6.53 (s, 1H), 5.46 (t, *J* = 6.5 Hz, 1H), 3.34 – 3.25 (m, 4H), 2.32 (t, *J* = 7.4 Hz, 2H), 1.84 – 1.68 (m, 4H), 1.66 – 1.52 (m, 4H), 1.45 – 1.37 (m, 2H), 1.34 – 1.27 (m, 4H), 1.26 – 1.21 (m, 2H). ^{13}C NMR (101 MHz, Chloroform-*d*) δ 171.76, 140.31, 137.16, 135.15, 128.27, 128.25, 127.64, 126.99, 126.87, 126.51, 125.89, 77.34, 33.37, 32.83, 32.44, 32.14, 31.60, 31.42, 27.36, 26.96, 26.66, 24.35, 23.13. HRMS (ESI) *m/z* calcd for $[C_{27}H_{34}Br_2NaO_2]^+$ ($[M+Na]^+$): 571.0818, found: 571.0813. IR (ν/cm^{-1}) 3006, 2922, 2851, 1734, 1637, 1508, 1458, 1260, 750, 702. HPLC (IC, 0.46*25 cm, 2 μm, hexane/isopropanol = 99/1, flow 1 mL/min, detection at 254 nm) retention time = 8.405 min (major) and 11.137 min (minor).

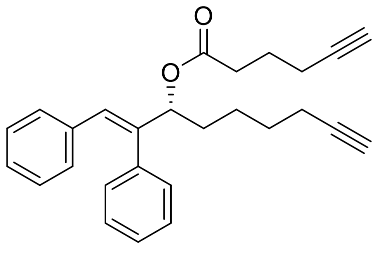

75, 68% yield, 97:3 er

According to General Procedure B, the reaction between diene **S2** (41.2 mg, 0.2 mmol, 1.0 equiv.) and peroxide **O33** (133.2 mg, 0.6 mmol, 3.0 equiv.) afforded product **75** was obtained as a colorless oil (52.2 mg, 68% yield, 97:3 er). $[\alpha]^{28.3}_{D}$ 39.01 (*c* 0.4, $CHCl_3$). ^1H NMR (400 MHz, Chloroform-*d*) δ 7.30 – 7.22 (m, 3H), 7.18 – 7.12 (m, 2H), 7.03 – 6.98 (m, 3H), 6.86 – 6.81 (m, 2H), 6.55 (s, 1H), 5.48 (t, *J* = 6.5 Hz, 1H), 2.48 – 2.41 (m, 2H), 2.26 – 2.16 (m, 2H), 2.12 – 2.04 (m, 2H), 1.91 (t, *J* = 2.6 Hz, 1H), 1.85 (t, *J* = 2.6 Hz, 1H), 1.83 – 1.77 (m, 2H), 1.61 – 1.53 (m, 2H), 1.47 – 1.33 (m, 4H). ^{13}C NMR (101 MHz, Chloroform-*d*) δ 171.34, 140.04, 137.07, 135.12, 128.28, 127.66, 127.16, 126.86, 126.52, 125.90, 83.21, 82.24, 77.34, 68.16, 67.41, 32.19, 31.64, 27.03, 23.59, 22.59, 17.24, 16.84. HRMS (ESI) *m/z* calcd for $[C_{27}H_{28}NaO_2]^+$ ($[M+Na]^+$): 407.1982, found: 407.1982. IR (ν/cm^{-1}) 2921, 2851, 2256, 1734, 1637, 1541, 1458, 1260, 750, 702. HPLC (IC, 0.46*25 cm, 2 μm, hexane/isopropanol = 99/1, flow 1 mL/min, detection at 254 nm) retention time = 6.360 min (major) and 7.887 min (minor).

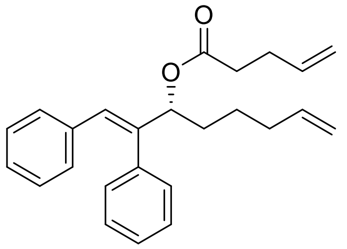

76, 50% yield, 97:3 er

According to General Procedure B, the reaction between diene **S2** (41.2 mg, 0.2 mmol, 1.0 equiv.) and peroxide **O31** (118.8 mg, 0.6 mmol, 3.0 equiv.) afforded product **76** was obtained as a colorless oil (36.0 mg, 50% yield, 97:3 er). $[\alpha]^{28.3}_{D}$ 36.52 (*c* 0.4, $CHCl_3$). ^1H NMR (600 MHz, Chloroform-*d*) δ 7.34 – 7.28 (m, 3H), 7.24 – 7.22 (m, 3H), 7.09 – 7.05 (m, 2H), 6.91 – 6.88 (m, 2H), 6.59 (s, 1H), 5.82 (ddt, *J* = 16.4, 10.2, 6.3 Hz, 1H), 5.73 (ddt, *J* = 16.9, 10.2, 6.7 Hz, 1H), 5.53 (t, *J* = 6.3 Hz, 1H), 5.06 (dq, *J* = 17.1, 1.6 Hz, 1H), 5.01 – 4.89 (m, 3H), 2.50 – 2.44 (m, 2H), 2.42 – 2.37 (m, 2H), 2.01 (dt, *J* = 13.7, 6.6 Hz, 2H), 1.67 – 1.59 (m, 2H), 1.52 – 1.45 (m, 1H), 1.44 – 1.37 (m, 1H). ^{13}C NMR (151 MHz, Chloroform-*d*) δ 172.51, 141.37, 138.40, 138.28, 136.77, 136.30, 129.40, 129.37, 128.76, 128.04, 127.98, 127.61, 126.97, 115.66, 114.91, 78.46, 33.92, 33.43, 32.79, 28.97, 24.92. HRMS (ESI) *m/z* calcd for $[C_{25}H_{28}NaO_2]^+$ ($[M+Na]^+$): 383.1982, found: 383.1982. IR (*v*/cm^{-1}) 3006, 2921, 2851, 1735, 1637, 1542, 1458, 1260, 750, 702. HPLC (IC, 0.46*25 cm, 2 μm, hexane/isopropanol = 99/1, flow 1 mL/min, detection at 254 nm) retention time = 4.353 min (major) and 5.001 min (minor).

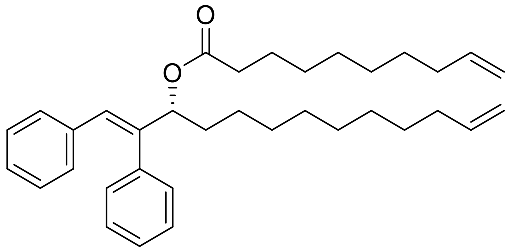

77, 65% yield, 96:4 er

According to General Procedure B, the reaction between diene **S2** (41.2 mg, 0.2 mmol, 1.0 equiv.) and peroxide **O77** (81.1 mg, 0.24 mmol, 1.2 equiv.) afforded product **77** was obtained as a colorless oil (65.0 mg, 65% yield, 96:4 er). $[\alpha]^{28.3}_{D}$ 44.06 (*c* 0.4, $CHCl_3$). ^1H NMR (400 MHz, Chloroform-*d*) δ 7.37 – 7.28 (m, 3H), 7.26 – 7.21 (m, 2H), 7.09 – 7.04 (m, 3H), 6.91 – 6.89 (m, 2H), 6.60 (s, 1H), 5.86 – 5.71 (m, 2H), 5.52 (t, *J* = 6.5 Hz, 1H), 4.98 (d, *J* = 17.2 Hz, 2H), 4.92 (d, *J* = 9.8 Hz, 2H), 2.36 (t, *J* = 7.5 Hz, 2H), 2.09 – 1.91 (m, 4H), 1.68 – 1.55 (m, 4H), 1.39 – 1.18 (m, 20H). ^{13}C NMR (101 MHz, Chloroform-*d*) δ 173.20, 141.60, 139.20, 139.12, 138.35, 136.33, 129.36, 129.29, 128.62, 127.87, 127.46, 126.82, 114.19, 114.14, 78.36, 34.71, 33.82, 33.78, 33.32, 29.44, 29.38, 29.28, 29.18, 29.14, 29.09, 28.95, 28.92, 28.87, 25.58, 25.05. HRMS (ESI) *m/z* calcd for $[C_{35}H_{48}NaO_2]^+$ ($[M+Na]^+$): 523.3547, found: 523.3543. IR (*v*/cm^{-1}) 3006, 2920, 2849 1734, 1637, 1508, 1458, 1260, 750, 702. HPLC (OD-H, 0.46*25 cm, 2 μm, hexane/isopropanol = 99.5/0.5, flow 1.0 mL/min, detection at 254 nm) retention time = 7.005 min (major) and 8.313 min (minor).

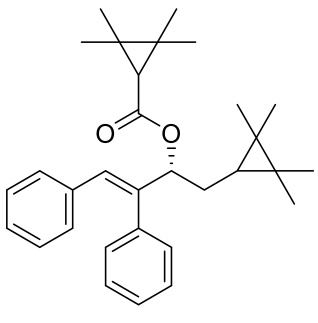

78, 50% yield, 94:6 er

According to General Procedure B, the reaction between diene **S2** (41.2 mg, 0.2 mmol, 1.0 equiv.) and peroxide **O38** (67.7 mg, 0.24 mmol, 1.2 equiv.) afforded product **78** was obtained as a white solid (44.4 mg, 50% yield, 94:6 er). $[\alpha]^{28.3}_{D}$ 39.01 (*c* 0.4, $CHCl_3$). Mp 84.9-86.5 °C. ^1H NMR (600 MHz, Chloroform-*d*) δ 7.34 – 7.28 (m, 3H), 7.25 – 7.23 (m, 2H), 7.11 – 7.06 (m, 3H), 6.93 (dd, *J* = 7.5, 1.8 Hz, 2H), 6.61 (s, 1H), 5.53 (dd, *J* = 7.8, 5.2 Hz, 1H), 1.67 – 1.60 (m, 1H), 1.58 – 1.52 (m, 1H), 1.27 (s, 3H), 1.25 (s, 1H), 1.21 (s, 3H), 1.20 (s, 3H), 1.19 (s, 3H), 1.01 (s, 3H), 1.01 (s, 3H), 0.91 (s, 3H), 0.83 (s, 3H), 0.20 (t, *J* = 7.1 Hz, 1H). ^{13}C NMR (151 MHz, Chloroform-*d*) δ 171.49, 142.24, 138.66, 136.70, 129.48, 129.34, 128.62, 127.95, 127.41, 127.21, 126.74, 78.00, 36.11, 30.19, 30.10, 30.04, 29.57, 23.77, 23.75, 23.71, 23.67, 20.98, 20.81, 17.31, 17.00, 16.79, 16.77. HRMS (ESI) *m/z* calcd for $[C_{31}H_{40}NaO_2]^+$ ($[M+Na]^+$): 467.2921, found: 467.2922. IR (ν/cm^{-1}) 3006, 2989, 2920, 2851, 1734, 1637, 1541, 1458, 1260, 750, 702. HPLC (IC, 0.46*25 cm, 2 μm, hexane/isopropanol = 99.7/0.3, flow 1.0 mL/min, detection at 254 nm) retention time = 3.738 min (major) and 4.917 min (minor).

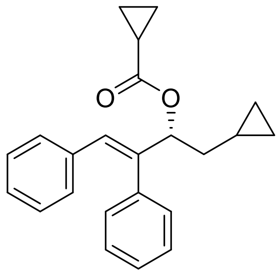

79, 38% yield, 96:4 er

According to General Procedure B, the reaction between diene **S2** (41.2 mg, 0.2 mmol, 1.0 equiv.) and peroxide **O79** (40.8 mg, 0.24 mmol, 1.2 equiv.) afforded product **79** was obtained as a colorless oil (25.2 mg, 38% yield, 96:4 er). $[\alpha]^{28.3}_{D}$ 42.43 (*c* 0.4, $CHCl_3$). ^1H NMR (400 MHz, Acetonitrile-d_3) δ 7.28 – 7.19 (m, 3H), 7.08 (dd, *J* = 7.4, 2.3 Hz, 2H), 7.00 – 6.94 (m, 3H), 6.80 (dd, *J* = 6.7, 3.0 Hz, 2H), 6.58 (s, 1H), 5.43 (t, *J* = 6.6 Hz, 1H), 1.57 – 1.48 (m, 1H), 1.42 (t, *J* = 6.8 Hz, 2H), 0.82 – 0.71 (m, 4H), 0.68 – 0.57 (m, 1H), 0.37 – 0.22 (m, 2H), -0.02 (h, *J* = 5.0 Hz, 1H), -0.08 – -0.17 (m, 1H). ^{13}C NMR (101 MHz, Chloroform-*d*) δ 173.13, 140.26, 137.23, 135.35, 128.36, 128.26, 127.55, 126.88, 126.83, 126.39, 125.77, 77.83, 37.44, 12.23, 7.32, 7.27, 6.57, 3.60, 3.06. HRMS (ESI) *m/z* calcd for $[C_{23}H_{24}NaO_2]^+$ ($[M+Na]^+$): 355.1669, found: 355.1667. IR (ν/cm^{-1}) 3006, 2922, 2851, 1725, 1637, 1542, 1458, 1260, 750, 701. HPLC (OD-H, 0.46*25 cm, 2 μm, hexane/isopropanol = 100/0, flow 1.0 mL/min, detection at 254 nm) retention time = 15.183 min (major) and 18.855 min (minor).

H. Mechanistic studies

1. Radical clock experiment

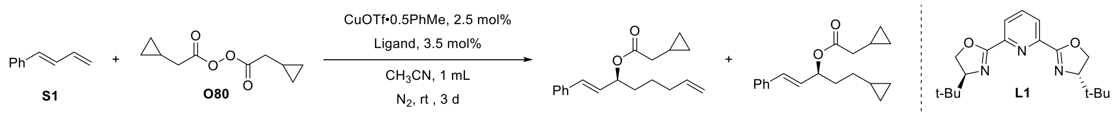

80, 40% yield, 95:5 er **81**, not detected

In a flame-dried Schlenk tube, Cu(OTf) 0.5PhMe (1.3 mg, 0.005 mmol, 2.5 mol%) and ligand **L1** (2.3 mg, 0.007 mmol, 3.5 mol%) were dissolved in CH_3CN (1.0 mL) under a nitrogen atmosphere, and the mixture was stirred at room temperature for 30 minutes. Then, diene **S1** (26.0 mg, 0.2 mmol, 1.0 equiv) and peroxide **O80** (79.2 mg, 0.4 mmol, 2.0 equiv) were sequentially added. The reaction mixture was stirred at room temperature for 3 days. After the reaction completion, the solvent was evaporated under reduced pressure. The residue was purified by flash column chromatography on silica gel to afford the product **80** as a colorless oil (22.7 mg, 40% yield, 95:5 er). $[\alpha]^{28.3}_{D}$ -43.01 (*c* 0.4, $CHCl_3$).

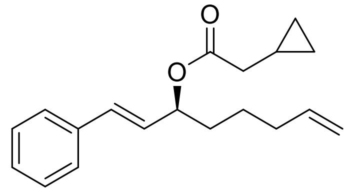

80, 40% yield, 95:5 er

^{1}H NMR (400 MHz, Chloroform-*d*) δ 7.38 (d, *J* = 7.3 Hz, 2H), 7.35 – 7.28 (m, 2H), 7.25 – 7.20 (m, 1H), 6.61 (d, *J* = 15.9 Hz, 1H), 6.13 (dd, *J* = 15.9, 7.1 Hz, 1H), 5.86 – 5.71 (m, 1H), 5.45 (q, *J* = 6.8 Hz, 1H), 5.06 – 4.93 (m, 2H), 2.24 (d, *J* = 7.1 Hz, 2H), 2.09 – 2.07 (m, 2H), 1.82 – 1.63 (m, 2H), 1.54 – 1.38 (m, 2H), 1.16 – 0.98 (m, 1H), 0.61 – 0.51 (m, 2H), 0.21 – 0.13 (m, 2H). ^{13}C NMR (101 MHz, Chloroform-*d*) δ 172.70, 138.43, 136.48, 132.34, 128.65, 127.96, 127.87, 126.64, 114.98, 74.48, 39.84, 34.14, 33.52, 24.54, 7.12, 4.49, 1.13. HRMS (ESI) *m/z* calcd for $[C_{19}H_{24}NaO_2]^+$ ($[M+Na]^+$): 307.1669, found: 307.1669. IR (v/cm^{-1}) 3005, 2921, 2851, 1772, 1734, 1541, 1447, 1260, 750, 695. HPLC (IC, 0.46*25 cm, 2 μm, hexane/isopropanol = 99/1, flow 1 mL/min, detection at 254 nm) retention time = 5.777 min (minor) and 6.557 min (major).

2. Radical trapping experiments

a. With BHT as an additive

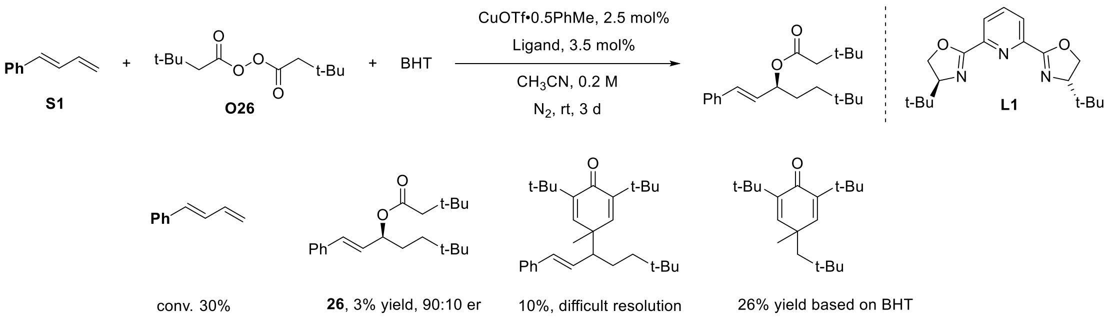

In a flame-dried Schlenk tube, Cu(OTf) 0.5PhMe (1.3 mg, 0.005 mmol, 2.5 mol%) and ligand **L2** (2.3 mg, 0.007 mmol, 3.5 mol%) were dissolved in CH_3CN (1.0 mL) under a nitrogen atmosphere, and the mixture was stirred at room temperature for 30 minutes. Then, diene **S1** (26.0 mg, 0.2 mmol, 1.0 equiv), peroxide **O26** (55.2 mg, 0.24 mmol, 1.2 equiv) and BHT (52.8 mg, 0.24 mmol, 1.2 equiv) were sequentially added. The reaction mixture was stirred at room temperature for 3 days. After the reaction completion, the solvent was evaporated under reduced pressure. The residue was purified by flash column chromatography on silica gel to afford the product.

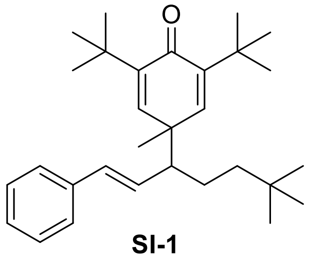

SI-1

A pale-yellow oil (8.4 mg, 10% yield). ^{1}H NMR (600 MHz, Chloroform-*d*) δ 7.36 – 7.29 (m, 4H), 7.24 – 7.21 (m, 1H), 6.49 – 6.44 (m, 2H), 6.37 (d, *J* = 15.8 Hz, 1H), 5.85 (dd, *J* = 16.4, 9.1 Hz, 1H), 2.08 (t, *J* = 10.2 Hz, 1H), 1.40 – 1.27 (m, 4H), 1.24 (s, 9H), 1.21 (s, 9H), 1.17 (s, 3H), 0.77 (s, 9H). ^{13}C NMR (151 MHz, Chloroform-*d*) δ 186.74, 147.15, 147.13, 146.65, 144.72, 137.26, 133.21, 130.33, 128.68, 127.41, 126.26, 54.19, 43.28, 42.48, 34.90, 34.82, 30.45, 29.64, 29.62, 29.37, 25.02, 24.97. HRMS (ESI) *m/z* calcd for $[C_{30}H_{44}NaO]^+$ ($[M+Na]^+$): 443.3284, found: 443.3284. IR (*v*/cm^{-1}) 2957, 2866, 1771, 1637, 1541, 1458, 1260, 750, 701.

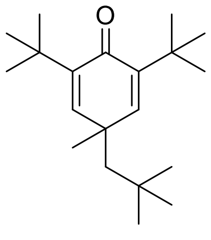

SI-2

A pale-yellow oil (18.1 mg, 26% yield (base on BHT). ^{1}H NMR (600 MHz, Chloroform-*d*) δ 6.56 (s, 2H), 1.71 (s, 2H), 1.22 (s, 18H), 1.09 (s, 3H), 0.80 (s, 9H). ^{13}C NMR (151 MHz, Chloroform-*d*) δ 186.45, 148.84, 144.00, 56.06, 41.04, 34.67, 32.42, 31.30, 31.13, 29.21. HRMS (ESI) *m/z* calcd for $[C_{20}H_{34}NaO]^+$ ($[M+Na]^+$): 313.2502, found: 313.2500. IR (*v*/cm^{-1}) 2958, 2867, 1771, 1637, 1541, 1458, 1260, 750, 701.

b. With TEMPO as an additive

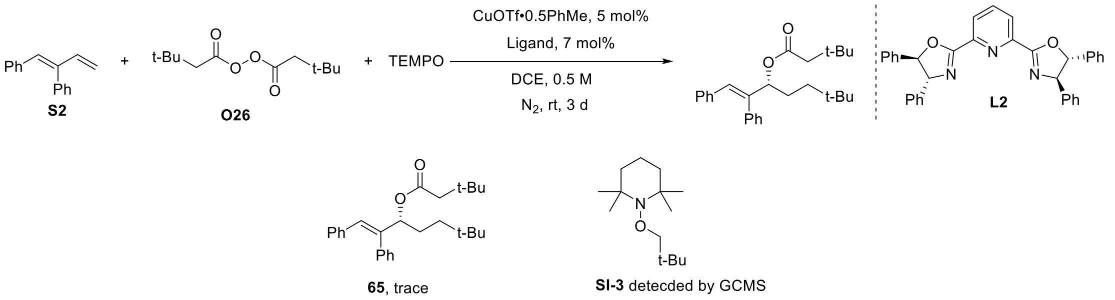

In a flame-dried Schlenk tube, Cu(OTf) 0.5PhMe (2.6 mg, 0.005 mmol, 2.5 mol%) and ligand **L2** (7.2 mg, 0.007 mmol, 3.5 mol%) were dissolved in CH_3CN (1.0 mL) under a nitrogen atmosphere, and the mixture was stirred at room temperature for 30 minutes. Then, diene **S1** (41.2 mg, 0.2 mmol, 1.0 equiv), peroxide **O26** (55.2 mg, 0.24 mmol, 1.2 equiv) and TEMPO (37.4 mg, 0.24 mmol, 1.2 equiv) were sequentially added. The reaction mixture was stirred at room temperature for 3 days. After the reaction completion, the solvent was evaporated under reduced pressure. GC-MS analysis suggested the formation of **SI-3**, indicating that alkyl radical species should be involved in the reaction.

3. Cross-over experiment

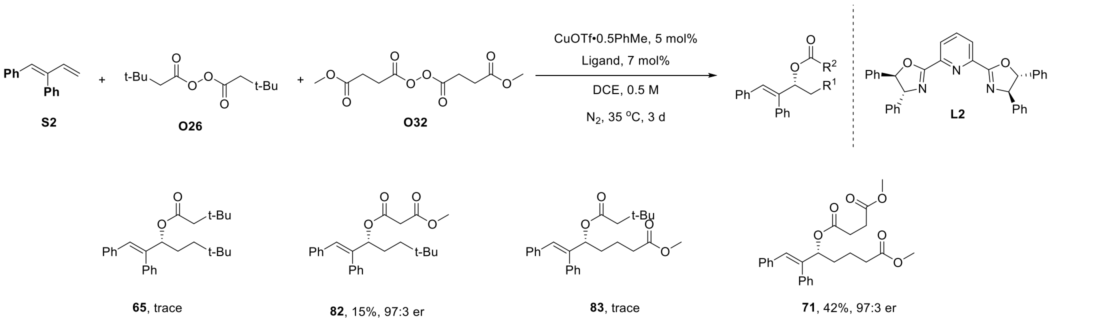

In a flame-dried Schlenk tube, Cu(OTf) • 0.5PhMe (2.6 mg, 0.01 mmol, 5 mol%) and ligand **L2** (7.2 mg, 0.014 mmol, 7 mol%) were dissolved in DCE (0.4 mL) under a nitrogen atmosphere, and the mixture was stirred at room temperature for 30 minutes. Then, **S1** (41.2 mg, 0.2 mmol, 1.0 equiv), **O26** (55.2 mg, 0.24 mmol, 1.2 equiv) and **O32** (62.9 mg, 0.24 mmol, 1.2 equiv) were sequentially added. The reaction mixture was stirred at room temperature for 3 days. After the reaction completion, the solvent was evaporated under reduced pressure. The residue was purified by flash column chromatography on silica gel to afford the product.

4. Reactions catalyzed by single crystals of dimer copper complexes

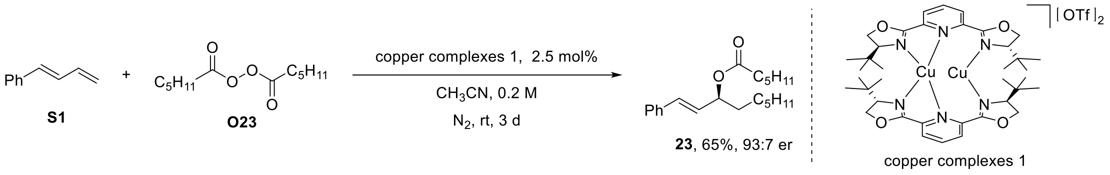

In a flame-dried Schlenk tube, copper complexes **1** (3.6 mg, 0.005 mmol, 2.5 mol%), **S1** (26.0 mg, 0.2 mmol, 1.0 equiv) and **O23** (92.0 mg, 0.4 mmol, 2 equiv) were dissolved in CH_3CN (1 mL) under a nitrogen atmosphere, and the mixture was stirred at room temperature for 3 days. After the reaction completion, the solvent was evaporated under reduced pressure. The residue was purified by flash column chromatography on silica gel to afford the product **23** as a colorless oil (41.1 mg, 65% yield, 93:7 er).

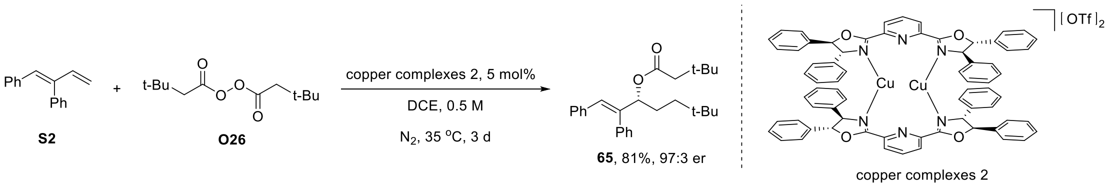

In a flame-dried Schlenk tube, copper complexes **2** (9.8 mg, 0.01 mmol, 5 mol%), **S2** (41.2 mg, 0.2 mmol, 1.0 equiv) and **O26** (55.2 mg, 0.24 mmol, 1.2 equiv) were dissolved in DCE (0.4 mL) under a nitrogen atmosphere, and the mixture was stirred at room temperature for 3 days. After the reaction completion, the solvent was evaporated under reduced pressure. The residue was purified by flash column chromatography on silica gel to afford the product **65** as a white solid (63.5 mg, 81% yield, 97:3 er).

5. MS studies

Experiment a (copper complexe 2)

Procedure: copper complexe 2 (0.005 mmol) was dissolved in DCE (0.5 mL) in an oven-dried Schlenk flask under the protection of dry nitrogen. Then the solution was diluted 1000 times by CH_3CN, subsequently transferred into an injection syringe and injected into the high-resolution electrospray mass spectrometry (SYNAPT G2-S HDMS) by injection pump. MS data were collected and analyzed.

Results:

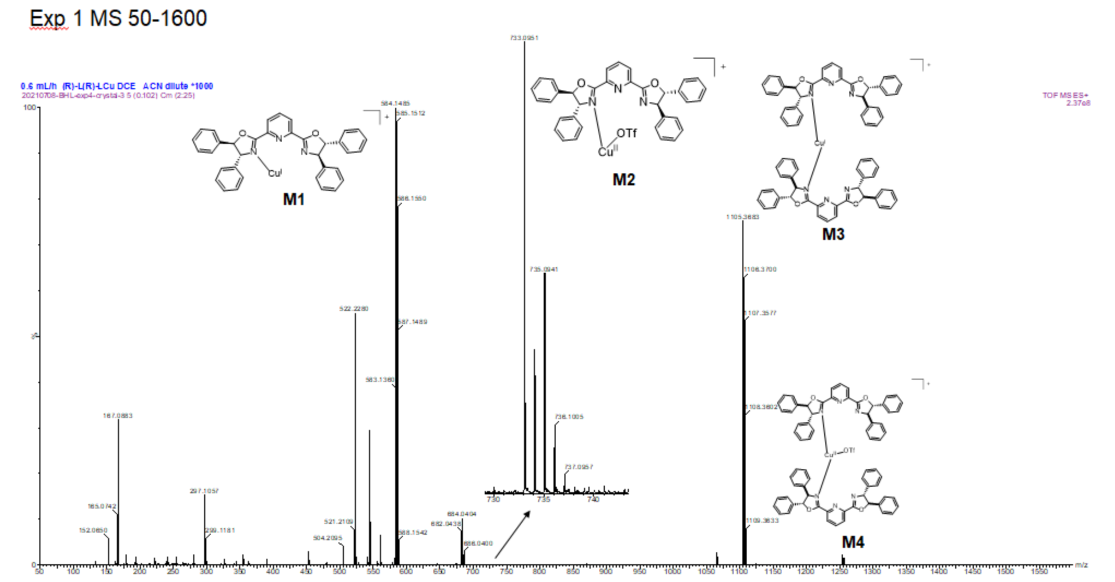

Supplementary Figure 3. The mass spectrum of Exp. a

We successfully detected moderate to high intensity of the key species **M1**, **M2**, **M3** and **M4** signal (**M1**, 584.1485, 15.6 ppm; **M2**, 733.0951, 5.0 ppm; **M3**, 1105.3683, 16.8 ppm; **M4**, 1254.3156, 11.1 ppm) with mass spectra (shown in Figure **S1**), and we also did collision induced dissociation experiments to further determine the structure of species **M1**, **M2**, **M3**, **M4** and the detailed CID data are shown in Figure **S2-S5**.

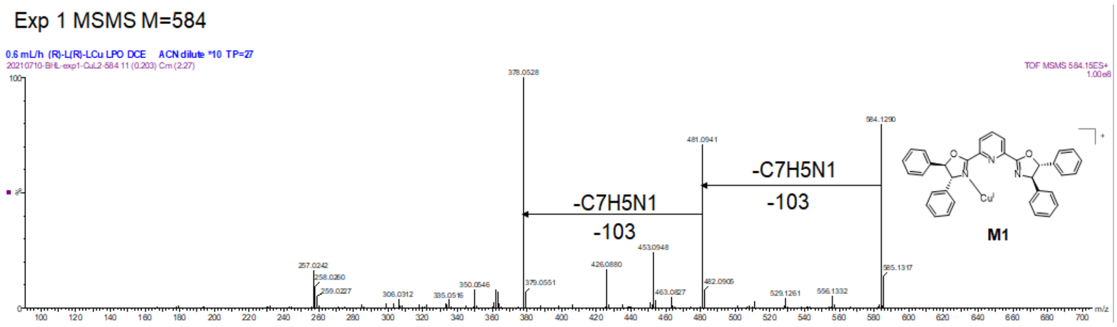

Supplementary Figure 4. The CID spectrum of **M1**.

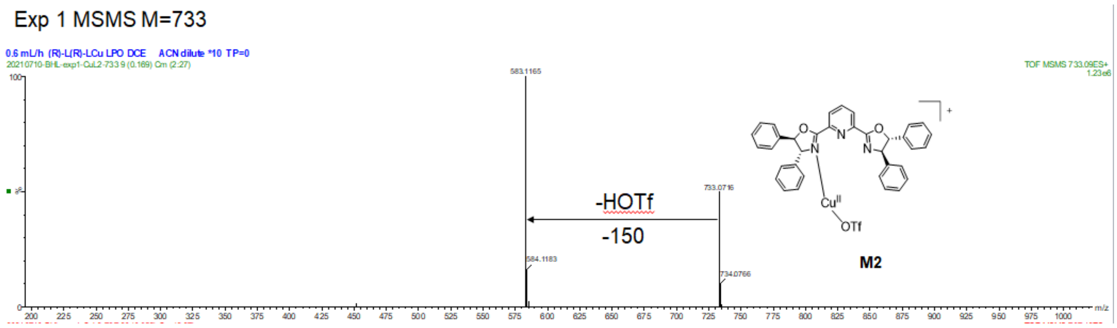

Supplementary Figure 5. The CID spectrum of **M2**

Exp 1 MSMS M=1105

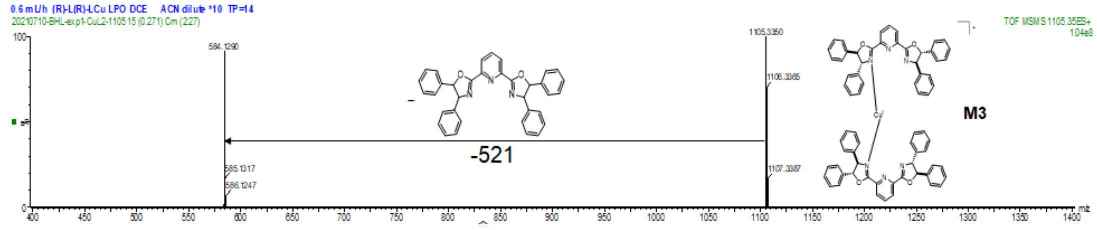

Supplementary Figure 6. The CID spectrum of **M3**.

Exp 1 MSMS M=1254

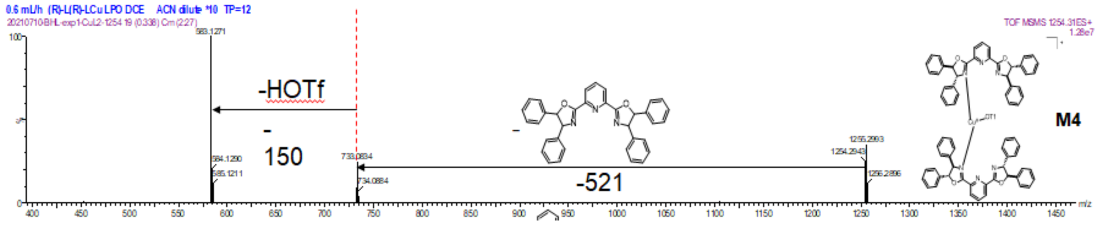

Supplementary Figure 7. The CID spectrum of **M4**

Experiment b (copper complexe 2 + LPO)

Procedure: copper complexe 2 (0.005 mmol) and LPO (0.025mmol) were introduced into an oven-dried Schlenk flask under the protection of dry nitrogen. Then 2 mL DCE was added into Schlenk flask. This mixture was stirred at room temperature under the nitrogen atmosphere for 30 minutes. Then the mixture was diluted 10 times by CH_3CN, subsequently transferred into an injection syringe and injected into the high-resolution electrospray mass spectrometry (SYNAPT G2-S HDMS) by injection pump. MS data were collected and analyzed.

Results:

Exp 2 MS 50-1600

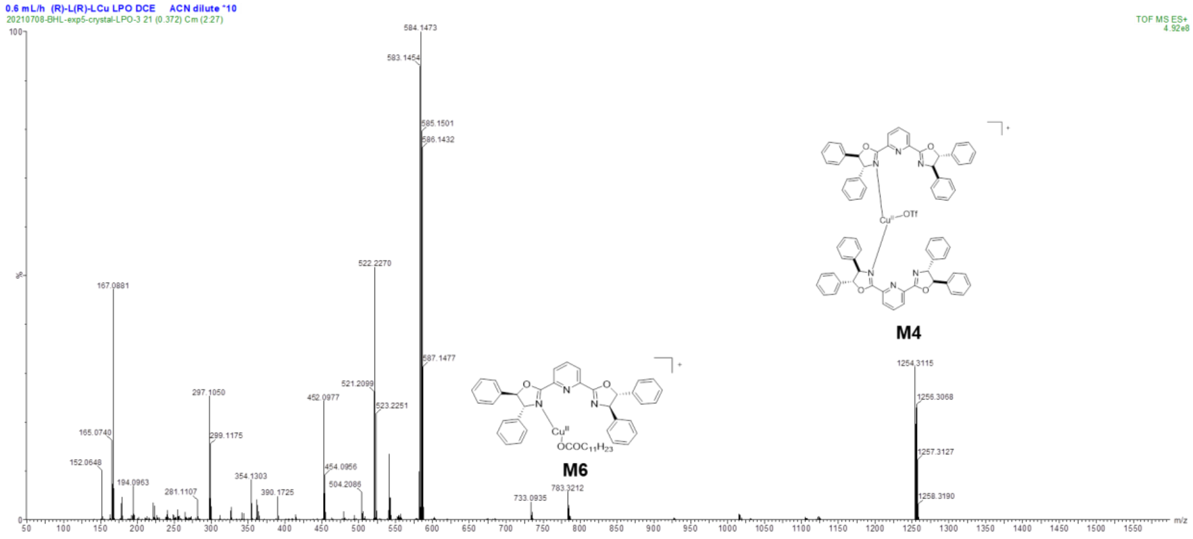

Supplementary Figure 8. The mass spectrum of Exp. 2

Except for species mentioned in Exp. 2 we successfully detected moderate intensity of the key species **M6** signal (**M6**, 783.3212, 15.3 ppm) with mass spectra (shown in Figure **S6**).

6. Kinetic Studies

A. Experiment for determining the dependence of reaction rate on the concentration of catalyst

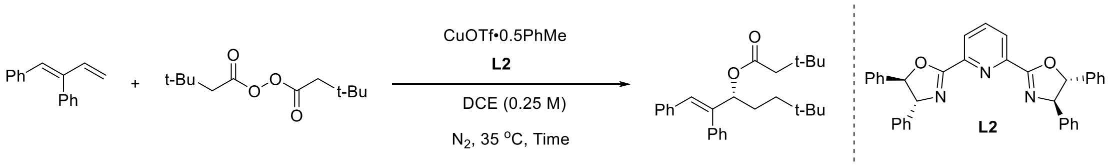

In a flame-dried Schlenk tube, given amount of Cu(OTf) 0.5PhMe and ligand **L2** were dissolved in DCE (3.2 mL, 0.25 M) under a nitrogen atmosphere, and the mixture was stirred at room temperature for 30 mins. Then, **S2** (164.8 mg, 0.8 mmol, 1.0 equiv) and **O26** (220.8 mg, 0.96 mmol, 1.2 equiv) and methyl pentadecanoate (20.5 mg, 0.08 mmol, 0.1 equiv) were sequentially added. The reaction mixture was stirred at 35 °C. An aliquot of the reaction mixture (0.20 mL) was taken out at a proper time interval. The reaction mixture was filtered through a short pad of silica gel and washed with EtOAc. Yields of product **65** were determined by GC analysis with methyl pentadecanoate as an internal standard.

Supplementary Table 7. The yield of product **65** with different initial concentrations of catalyst (ratio of Cu(OTf)·0.5PhMe and **L2** is 1:1.4) at different time intervals.

Time/min	GC yield of the reaction with 1.25 mol% of copper salt
4	0.89
8	1.66
12	2.25
16	3.16
20	3.53
30	4.64
40	6.4

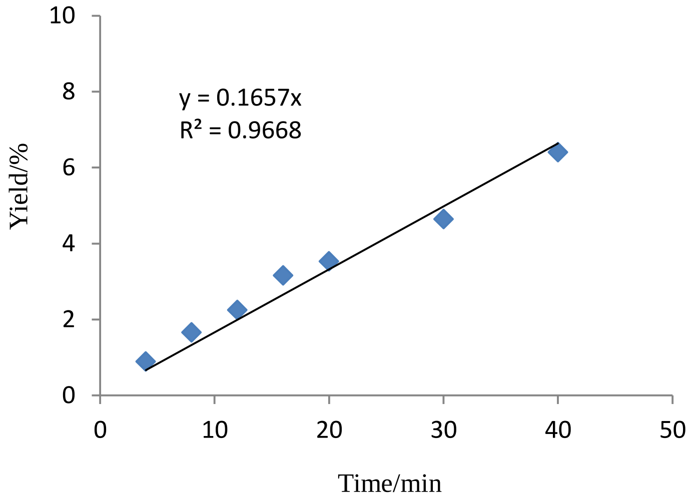

Time/min	GC yield of the reaction with 2.5 mol% of copper salt
2	0.8
4	1.4
6	1.92
8	2.31
10	2.94
15	4.41
20	5.84

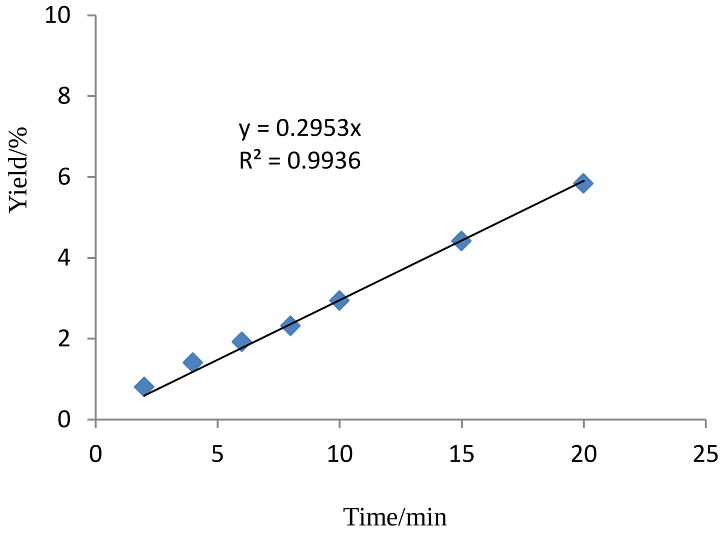

Time/min	GC yield of the reaction with 3.75 mol% of copper salt
2	0.893
4	2.02
6	2.85
8	3.81
10	4.71
15	7.01
20	8.21

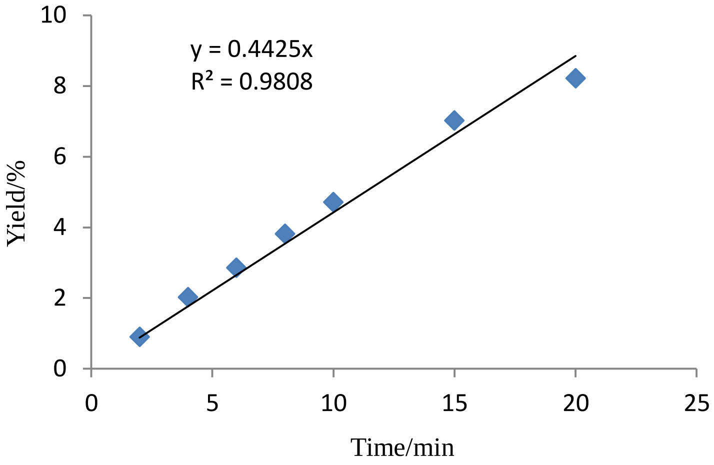

Time/min	GC yield of the reaction with 5 mol% of copper salt
2	1.43
4	2.3
6	3.3
8	4.18
10	6.04
15	8.27
20	9.65

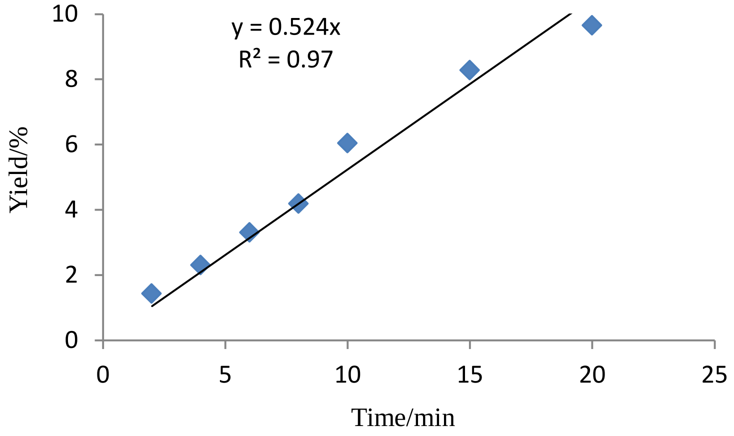

Time/min	GC yield of the reaction with 7.5 mol% of copper salt
2	2.07
4	3.36
6	5.19
8	6.38
10	8.16
15	10.65

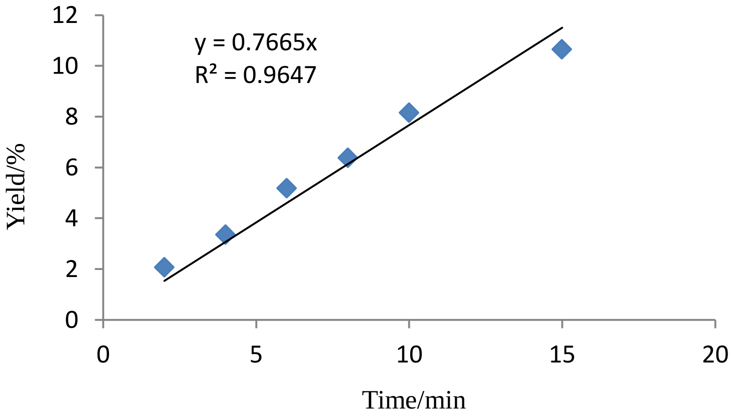

Supplementary Table 8. The *k* values at different initial concentrations of catalyst

catalyst	1.25 mol%	2.5 mol%	3.75 mol%	5 mol%	7.5 mol%
k	0.1657	0.2953	0.4425	0.524	0.7665

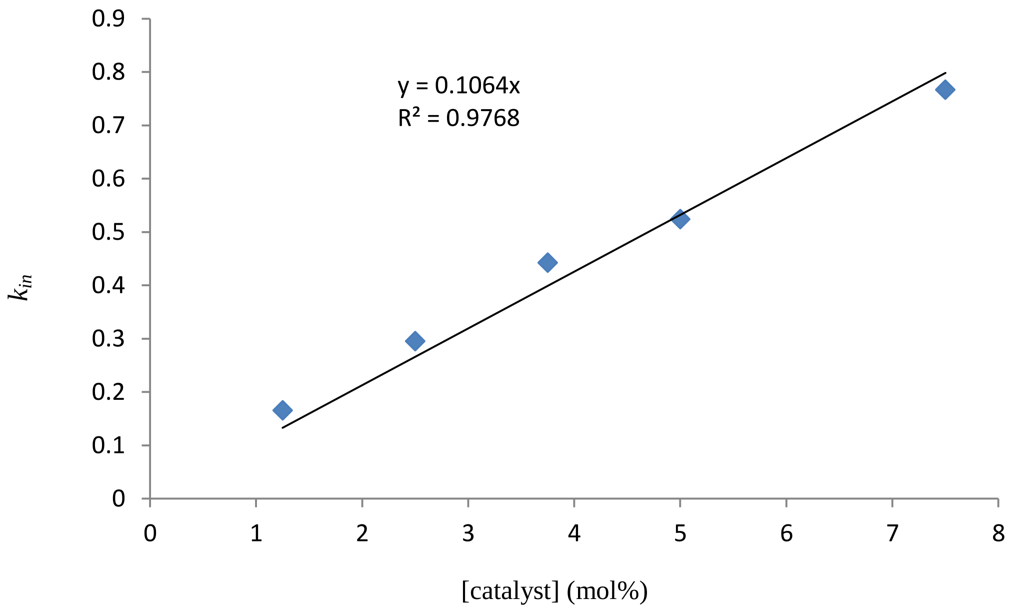

Supplementary Figure 9. Plot on catalyst

B. Experiment for determining the dependence of reaction rate on the concentration of solvent

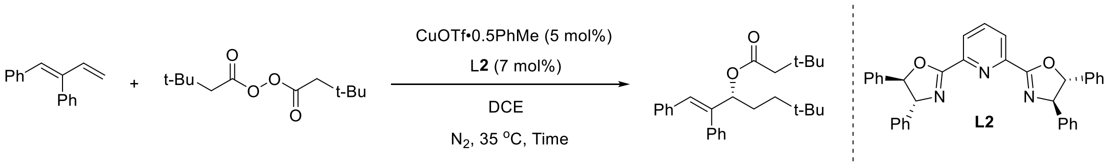

In a flame-dried Schlenk tube, Cu(OTf) 0.5PhMe (7.8 mg, 0.03 mmol, 5 mol%) and ligand **L2** (21.9 mg, 0.042 mmol, 7 mol%) were dissolved in DCE under a nitrogen atmosphere, and the mixture was stirred at room temperature for 30 mins. Then, **S2** (123.6 mg, 0.6 mmol, 1.0 equiv) and **O26** (187.2 mg, 0.72 mmol, 1.2 equiv) and methyl pentadecanoate (15.4 mg, 0.06 mmol, 0.1 equiv) were sequentially added. The reaction mixture was stirred at 35 °C. An aliquot of the reaction mixture (0.20 mL) was taken out at a proper time interval. The reaction mixture was filtered through a short pad of silica gel and washed with EtOAc. Yields of product **65** were determined by GC analysis with methyl pentadecanoate as an internal standard.

Supplementary Table 9. The yield of product **65** with different amount of DCE at different time intervals.

Time/min	GC-Yield/ DCE (DCE, 0.25 M) from Table **S2** with 5 mol% of copper
2	1.43
4	2.3
6	3.3
8	4.18
10	6.04
15	8.27
20	9.65

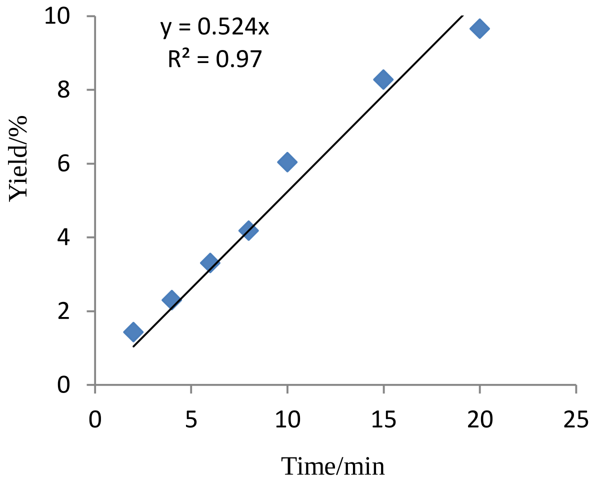

Time/min	GC-Yield (DCE, 0.167 M)
2	1.73
4	3.39
6	4.79
8	6.69
10	7.19
12	8.89

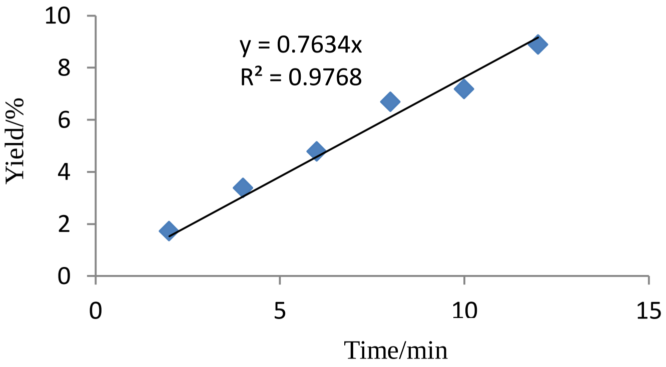

Time/min	GC-Yield/ DCE (DCE, 0.125 M)
2	2.15
4	3.91
6	5.38
8	6.67
10	8.28
12	9.59

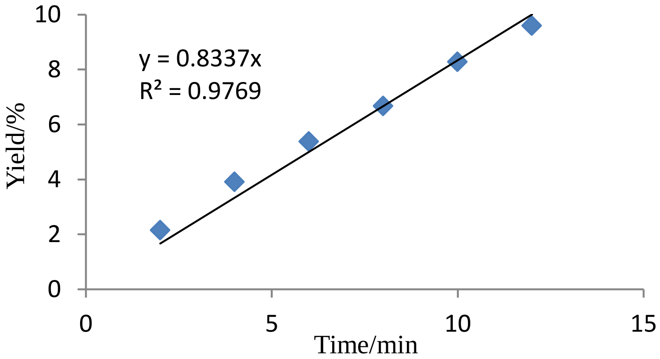

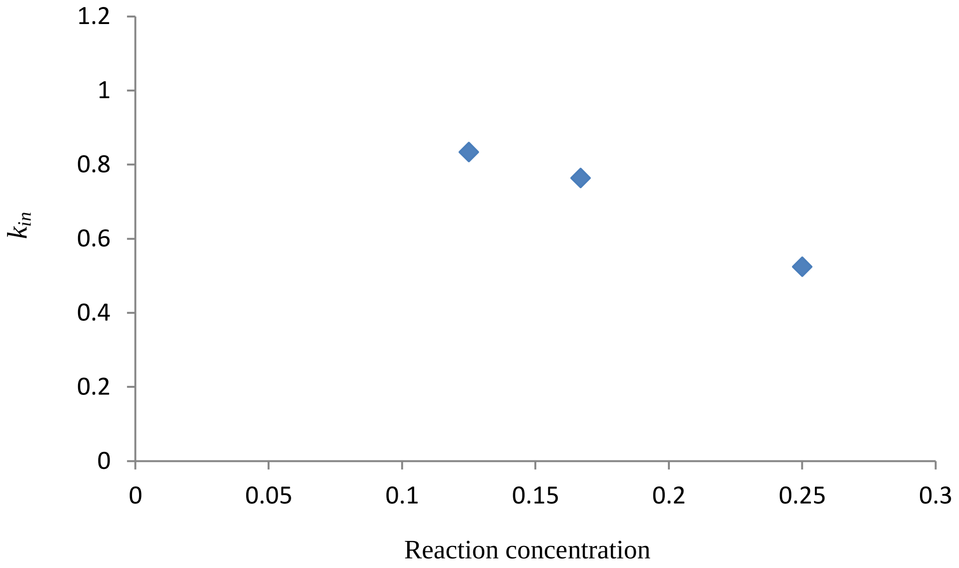

Supplementary Figure 10. The *k* values at different volume of DCE

I. Synthetic applications

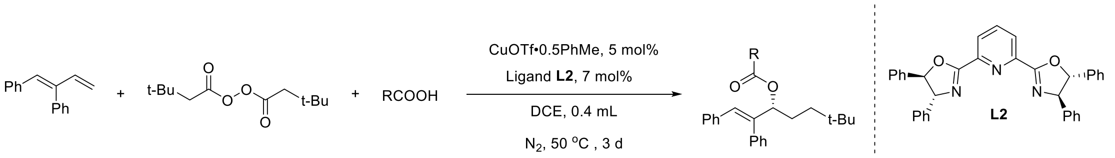

General procedure C: In a flame-dried Schlenk tube, Cu(OTf)·0.5PhMe (0.01 mmol, 5 mol%) and ligand **L2** (0.014 mmol, 5 mol%) were dissolved in DCE (0.4 mL, 0.5 M) under a nitrogen atmosphere, and the mixture was stirred at room temperature for 30 mins. Then, diene (0.2 mmol, 1.0 equiv), peroxide (0.24 mmol, 1.2 equiv) and acid (0.22 mmol, 1.1 equiv) were sequentially added. The reaction mixture was stirred at 50 °C for 3 days. After reaction completion, the solvent was evaporated under reduced pressure. The residue was purified by flash column chromatography on silica gel to afford the product.

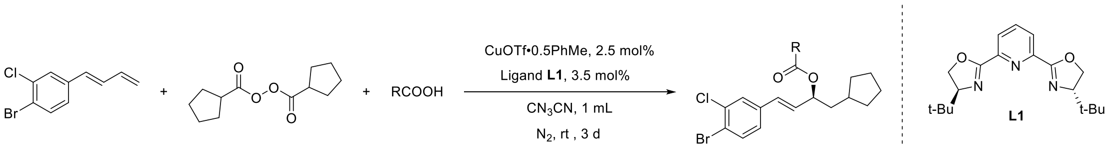

General procedure D: In a flame-dried Schlenk tube, Cu(OTf)·0.5PhMe (0.005 mmol, 2.5 mol%) and ligand **L1** (0.007 mmol, 3.5 mol%) were dissolved in CH_3CN (1.0 mL, 0.2 M) under a nitrogen atmosphere, and the mixture was stirred at room temperature for 30 mins. Then, diene (0.2 mmol, 1.0 equiv), peroxide (0.4 mmol, 2.0 equiv) and acid (0.30 mmol, 1.5 equiv) were sequentially added. The reaction mixture

was stirred at room temperature for 3 days. After reaction completion, the solvent was evaporated under reduced pressure. The residue was purified by flash column chromatography on silica gel to afford the product.

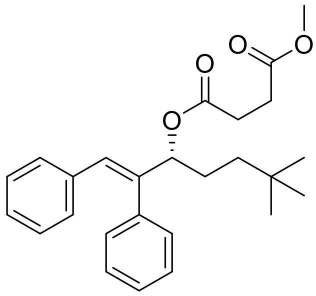

82, 72% yield, 97:3 er

According to General Procedure C, the reaction between diene **S2** (41.2 mg, 0.2 mmol, 1.0 equiv.), peroxide **O26** (55.2 mg, 0.24 mmol, 1.2 equiv.) and methyl hydrogen succinate (29.0, 0.22 mmol, 1.1 equiv) afforded product **82** was obtained as a colorless oil (63.6 mg, 72% yield, 97:3 er). $[\alpha]^{28.3}_{D}$ 39.69 (*c* 0.4, $CHCl_3$). ^1H NMR (400 MHz, Chloroform-*d*) δ 7.36 – 7.29 (m, 3H), 7.24 – 7.19 (m, 2H), 7.14 – 7.03 (m, 3H), 6.96 – 6.82 (m, 2H), 6.60 (s, 1H), 5.49 (t, *J* = 6.5 Hz, 1H), 3.67 (s, 3H), 2.79 – 2.60 (m, 4H), 1.64 – 1.50 (m, 2H), 1.34 – 1.27 (m, 1H), 1.23 – 1.13 (m, 1H), 0.82 (s, 9H). ^{13}C NMR (101 MHz, Chloroform-*d*) δ 172.68, 171.52, 140.75, 138.04, 136.16, 129.33, 129.31, 128.62, 127.86, 127.50, 126.91, 79.78, 51.85, 39.16, 30.05, 29.51, 29.28, 28.98, 28.15. HRMS (ESI) *m/z* calcd for $[C_{26}H_{32}NaO_4]^+$ ($[M+Na]^+$): 431.2193, found: 431.2194. IR (*v*/cm^{-1}) 3006, 2954, 2865, 1735, 1648, 1508, 1451, 1260, 1157, 750, 702. HPLC (IC, 0.46*25 cm, 2 μm, hexane/isopropanol = 97/3, flow 1.0 mL/min, detection at 254 nm) retention time = 6.249 min (major) and 7.304 min (minor).

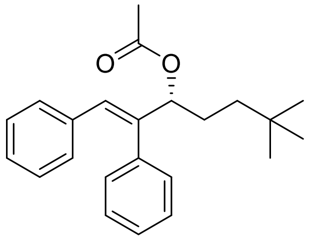

84, 67% yield, 95:5 er

According to General Procedure C, the reaction between diene **S2** (41.2 mg, 0.2 mmol, 1.0 equiv.), peroxide **O26** (55.2 mg, 0.24 mmol, 1.2 equiv.) and acetic acid (13.2, 0.22 mmol, 1.1 equiv) afforded product **84** was obtained as a colorless oil (45.0 mg, 67% yield, 95:5 er). $[\alpha]^{28.3}_{D}$ 42.19 (*c* 0.4, $CHCl_3$). ^1H NMR (600 MHz, Chloroform-*d*) δ 7.35 – 7.29 (m, 3H), 7.24 – 7.20 (m, 2H), 7.10 – 7.05 (m, 3H), 6.93 – 6.89 (m, 2H), 6.61 (s, 1H), 5.48 (t, *J* = 6.4 Hz, 1H), 2.11 (s, 3H), 1.62 – 1.52 (m, 2H), 1.31 – 1.26 (m, 1H), 1.23 – 1.13 (m, 1H), 0.82 (s, 9H). ^{13}C NMR (151 MHz, Chloroform-*d*) δ 170.50, 141.06, 138.22, 136.31, 129.42, 128.72, 128.64, 127.98, 127.59, 127.00, 79.46, 39.32, 30.16, 29.39, 28.23, 21.51. HRMS (ESI) *m/z* calcd for $[C_{23}H_{28}NaO_2]^+$ ($[M+Na]^+$): 359.1982, found: 359.1981. IR (*v*/cm^{-1}) 2955, 2865, 1736, 1637, 1542, 1458, 1260, 750, 702. HPLC (OD-H, 0.46*25 cm, 2 μm, hexane/isopropanol = 99.5/0.5, flow 1.0 mL/min, detection at 254 nm) retention time = 5.615 min (major) and 10.828 min (minor).

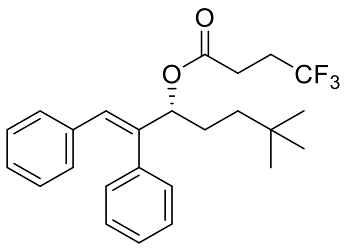

85, 62% yield, 95:5 er

According to General Procedure C, the reaction between diene **S2** (41.2 mg, 0.2 mmol, 1.0 equiv.), peroxide **O26** (55.2 mg, 0.24 mmol, 1.2 equiv.) and 4,4,4-trifluorobutyric acid (31.2, 0.22 mmol, 1.1 equiv) afforded product **85** was obtained as a colorless oil (51.8 mg, 62% yield, 95:5 er). $[\alpha]^{28.3}_{D}$ 34.02 (*c* 0.4, $CHCl_3$). ^1H NMR (600 MHz, Chloroform-*d*) δ 7.38 – 7.27 (m, 3H), 7.21 – 7.19 (m, 2H), 7.12 – 7.04 (m, 3H), 6.92 – 6.90 (m, 2H), 6.60 (s, 1H), 5.50 (t, *J* = 6.4 Hz, 1H), 2.69 – 2.54 (m, 2H), 2.53 – 2.40 (m, 2H), 1.65 – 1.53 (m, 2H), 1.31 – 1.26 (m, 1H), 1.22 – 1.16 (m, 1H), 0.82 (s, 9H). ^{13}C NMR (151 MHz, Chloroform-*d*) δ 170.38, 140.61, 137.97, 136.08, 129.44, 129.35, 129.02, 128.80, 128.03, 127.73, 127.16, 80.39, 39.32, 30.16, 29.50, 29.36, 29.31, 28.26, 27.55, 27.53, 27.51, 27.49. ^{19}F NMR (565 MHz, Chloroform-*d*) δ -66.78. HRMS (ESI) *m/z* calcd for $[C_{25}H_{29}F_3NaO_4S]^+$ ($[M+Na]^+$): 441.2012, found: 441.2013. IR (v/cm^{-1}) 3007, 2956, 2851, 1734, 1637, 1541, 1458, 1260, 750, 702. HPLC (OD-H, 0.46*25 cm, 2 μm, hexane/isopropanol = 99.5/0.5, flow 1.0 mL/min, detection at 254 nm) retention time = 5.087 min (major) and 6.928 min (minor).

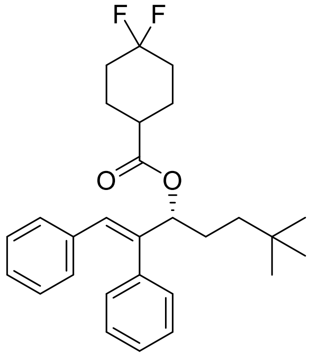

86, 60% yield, 95:5 er

According to General Procedure C, the reaction between diene **S2** (41.2 mg, 0.2 mmol, 1.0 equiv.), peroxide **O26** (55.2 mg, 0.24 mmol, 1.2 equiv.) and 4,4-difluorocyclohexanecarboxylic acid (36.1, 0.22 mmol, 1.1 equiv) afforded product **86** was obtained as a colorless oil (52.8 mg, 60% yield, 95:5 er). $[\alpha]^{28.3}_{D}$ 44.19 (*c* 0.4, $CHCl_3$). ^1H NMR (400 MHz, Chloroform-*d*) δ 7.36 – 7.30 (m, 3H), 7.24 – 7.22 (m, 2H), 7.12 – 7.04 (m, 3H), 6.92 – 6.90 (m, 2H), 6.59 (s, 1H), 5.50 (t, *J* = 6.5 Hz, 1H), 2.50 – 2.40 (m, 1H), 2.14 – 1.95 (m, 4H), 1.92 – 1.75 (m, 4H), 1.67 – 1.45 (m, 3H), 1.35 – 1.28 (m, 1H), 1.23 – 1.15 (m, 1H), 0.83 (s, 9H). ^{13}C NMR (101 MHz, Chloroform-*d*) δ 173.47, 141.02, 138.06, 136.12, 129.32, 129.29, 128.67, 128.41, 127.93, 127.59, 126.99, 122.71 (t, *J* = 240.0 Hz), 79.37, 40.84, 39.36, 32.59 (t, *J* = 24.6 Hz), 32.56 (t, *J* = 24.5 Hz), 30.09, 29.72, 29.30, 28.34, 25.11 (dd, *J* = 6.4, 3.9 Hz). HRMS (ESI) *m/z* calcd for $[C_{28}H_{34}F_2NaO_2]^+$ ($[M+Na]^+$): 463.2419, found: 463.2420. IR (v/cm^{-1}) 3006, 2956, 2852, 1733, 1648, 1541, 1445, 1260, 750, 703. HPLC (OD-H, 0.46*25 cm, 2 μm, hexane/isopropanol = 100/0, flow 1.0 mL/min, detection at 254 nm) retention time = 17.453 min (major) and 53.489 min (minor).

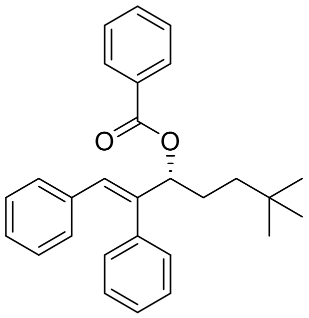

87, 78% yield, 96:4 er

According to General Procedure C, the reaction between diene **S2** (41.2 mg, 0.2 mmol, 1.0 equiv.), peroxide **O26** (55.2 mg, 0.24 mmol, 1.2 equiv.) and benzoic acid (26.8, 0.22 mmol, 1.1 equiv) afforded product **87** was obtained as a colorless oil (62.1 mg, 78% yield, 96:4 er). $[\alpha]^{28.3}_{D}$ 32.28 (*c* 0.4, $CHCl_3$). ^1H NMR (600 MHz, Chloroform-*d*) δ 8.10 (d, *J* = 7.5 Hz, 2H), 7.57 (t, *J* = 7.4 Hz, 1H), 7.47 (t, *J* = 7.7 Hz, 2H), 7.37 – 7.33 (m, 3H), 7.30 (d, *J* = 8.0 Hz, 2H), 7.09 – 7.05 (m, 3H), 6.93 (d, *J* = 3.7 Hz, 2H), 6.72 (s, 1H), 5.75 (t, *J* = 6.5 Hz, 1H), 1.76 – 1.68 (m, 2H), 1.42 – 1.35 (m, 1H), 1.33 – 1.26 (m, 1H), 0.85 (s, 9H). ^{13}C NMR (151 MHz, Chloroform-*d*) δ 165.96, 140.98, 138.25, 136.27, 133.03, 130.71, 129.75, 129.52, 129.47, 128.91, 128.76, 128.53, 127.97, 127.63, 127.02, 80.08, 39.41, 30.23, 29.42, 28.40. HRMS (ESI) *m/z* calcd for $[C_{28}H_{30}NaO_2]^+$ ($[M+Na]^+$): 421.2138, found: 421.2138. IR (ν/cm^{-1}) 2956, 2866, 1718, 1654, 1541, 1449, 1260, 750, 711. HPLC (OD-H, 0.46*25 cm, 2 μm, hexane/isopropanol = 100/0, flow 1.0 mL/min, detection at 254 nm) retention time = 13.600 min (major) and 17.672 min (minor).

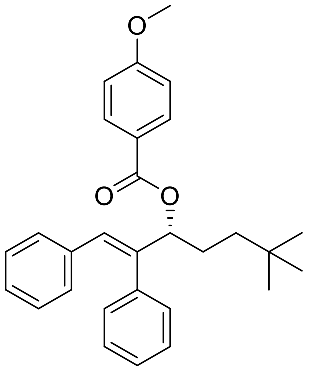

88, 40% yield, 95:5 er

According to General Procedure C, the reaction between diene **S2** (41.2 mg, 0.2 mmol, 1.0 equiv.), peroxide **O26** (55.2 mg, 0.24 mmol, 1.2 equiv.) and 4-methoxybenzoic acid (33.4, 0.22 mmol, 1.1 equiv) afforded product **88** was obtained as a colorless oil (34.2 mg, 40% yield, 95:5 er). $[\alpha]^{28.3}_{D}$ 36.02 (*c* 0.4, $CHCl_3$). ^1H NMR (600 MHz, Chloroform-*d*) δ 8.04 (d, *J* = 8.6 Hz, 2H), 7.36 – 7.31 (m, 3H), 7.28 (d, *J* = 7.5 Hz, 2H), 7.07 – 7.03 (m, 3H), 6.94 (d, *J* = 8.8 Hz, 2H), 6.92 – 6.88 (m, 2H), 6.69 (s, 1H), 5.70 (t, *J* = 6.5 Hz, 1H), 3.86 (s, 3H), 1.73 – 1.65 (m, 2H), 1.39 – 1.27 (m, 2H), 0.83 (s, 9H). ^{13}C NMR (151 MHz, Chloroform-*d*) δ 165.70, 163.44, 141.14, 138.33, 136.33, 131.75, 129.52, 129.45, 128.72, 127.94, 127.56, 126.95, 123.12, 113.76, 79.67, 55.55, 39.39, 30.21, 29.41, 28.39. HRMS (ESI) *m/z* calcd for $[C_{29}H_{32}NaO_3]^+$ ($[M+Na]^+$): 451.2244, found: 451.2243. IR (ν/cm^{-1}) 2955, 2865, 1708, 1637, 1510, 1458, 1260, 750, 697. HPLC (IC, 0.46*25 cm, 2 μm, hexane/isopropanol = 99.6/0.4, flow 1.0 mL/min, detection at 254 nm) retention time = 13.418 min (major) and 14.698 min (minor).

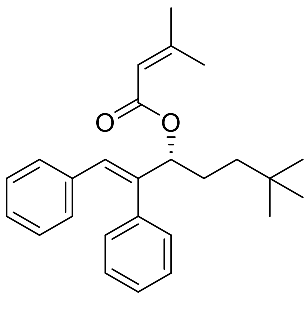

89, 52% yield, 95:5 er

According to General Procedure C, the reaction between diene **S2** (41.2 mg, 0.2 mmol, 1.0 equiv.), peroxide **O26** (55.2 mg, 0.24 mmol, 1.2 equiv.) and 3-methylbutenoic acid (22.0, 0.22 mmol, 1.1 equiv) afforded product **89** was obtained as a white solid (39.1 mg, 52% yield, 95:5 er). $[\alpha]^{28.3}_{D}$ 26.78 (*c* 0.4, $CHCl_3$). Mp 69.5-71.8 °C. ^1H NMR (600 MHz, Chloroform-*d*) δ 7.36 – 7.26 (m, 3H), 7.25 – 7.23 (m, 2H), 7.09 – 7.02 (m, 3H), 6.96 – 6.85 (m, 2H), 6.60 (s, 1H), 5.76 (s, 1H), 5.50 (t, *J* = 7.1 Hz, 1H), 2.18 (s, 3H), 1.91 (s, 3H), 1.56 (td, *J* = 13.3, 6.7 Hz, 2H), 1.35 – 1.26 (m, 1H), 1.22 – 1.12 (m, 1H), 0.81 (s, 9H). ^{13}C NMR (151 MHz, Chloroform-*d*) δ 166.06, 157.19, 141.45, 138.45, 136.50, 129.46, 129.41, 128.67, 128.18, 127.92, 127.49, 126.84, 116.35, 78.37, 39.36, 30.16, 29.40, 28.31, 27.61, 20.40. HRMS (ESI) *m/z* calcd for $[C_{26}H_{32}NaO_2]^+$ ($[M+Na]^+$): 399.2295, found: 399.2296. IR (ν/cm^{-1}) 3006, 2956, 2852, 1733, 1648, 1541, 1458, 1260, 750, 703. HPLC (OD-H, 0.46*25 cm, 2 μm, hexane/isopropanol = 100/0, flow 1.0 mL/min, detection at 254 nm) retention time = 9.459 min (major) and 14.628 min (minor).

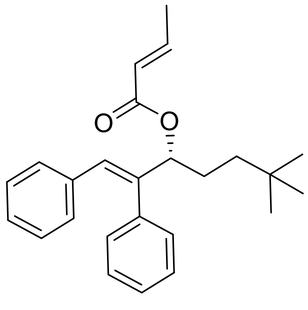

90, 63% yield, 92:8 er

According to General Procedure C, the reaction between diene **S2** (41.2 mg, 0.2 mmol, 1.0 equiv.), peroxide **O26** (55.2 mg, 0.24 mmol, 1.2 equiv.) and crotonic acid (18.9, 0.22 mmol, 1.1 equiv) afforded product **90** was obtained as a colorless oil (45.6 mg, 63% yield, 92:8 er). $[\alpha]^{28.3}_{D}$ 35.43 (*c* 0.4, $CHCl_3$). ^1H NMR (400 MHz, Chloroform-*d*) δ 7.37 – 7.29 (m, 3H), 7.25 – 7.20 (m, 2H), 7.10 – 6.97 (m, 4H), 6.94 – 6.87 (m, 2H), 6.62 (s, 1H), 5.93 (dd, *J* = 15.5, 1.8 Hz, 1H), 5.54 (t, *J* = 6.5 Hz, 1H), 1.91 – 1.89 (m, 3H), 1.66 – 1.52 (m, 2H), 1.36 – 1.28 (m, 1H), 1.24 – 1.15 (m, 1H), 0.82 (s, 9H). ^{13}C NMR (101 MHz, Chloroform-*d*) δ 165.84, 144.76, 141.06, 138.22, 136.29, 129.37, 129.32, 128.57, 128.45, 127.83, 127.42, 126.81, 123.02, 79.02, 39.22, 30.06, 29.29, 28.20, 18.05. HRMS (ESI) *m/z* calcd for $[C_{25}H_{30}NaO_2]^+$ ($[M+Na]^+$): 385.2138, found: 385.2136. IR (ν/cm^{-1}) 2955, 2865, 1719, 1654, 1541, 1446, 1260, 750, 702. HPLC (OD-H, 0.46*25 cm, 2 μm, hexane/isopropanol = 99.5/0.5, flow 1.0 mL/min, detection at 254 nm) retention time = 5.748 min (major) and 7.404 min (minor).

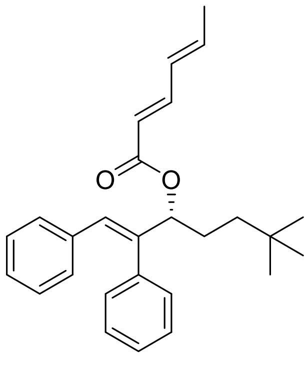

91, 45% yield, 93:7 er

According to General Procedure C, the reaction between diene **S2** (41.2 mg, 0.2 mmol, 1.0 equiv.), peroxide **O26** (55.2 mg, 0.24 mmol, 1.2 equiv.) and sorbic acid (24.6, 0.22 mmol, 1.1 equiv) afforded product **91** was obtained as a colorless oil (34.9 mg, 45% yield, 93:7 er). $[\alpha]^{28.3}_{D}$ 27.04 (*c* 0.4, $CHCl_3$). ^{1}H NMR (600 MHz, Chloroform-*d*) δ 7.35 – 7.26 (m, 4H), 7.25 – 7.21 (m, 2H), 7.09 – 7.03 (m, 3H), 6.92 – 6.87 (m, 2H), 6.61 (s, 1H), 6.26 – 6.08 (m, 2H), 5.84 (d, *J* = 15.3 Hz, 1H), 5.54 (t, *J* = 6.3 Hz, 1H), 1.85 (d, *J* = 6.4 Hz, 3H), 1.33 – 1.25 (m, 2H), 1.24 – 1.16 (m, 2H), 0.81 (s, 9H). ^{13}C NMR (151 MHz, Chloroform-*d*) δ 166.74, 145.33, 141.12, 139.60, 138.32, 136.37, 129.89, 129.48, 129.43, 128.68, 128.59, 127.93, 127.52, 126.92, 119.28, 79.22, 39.33, 30.17, 29.39, 28.29, 18.80. HRMS (ESI) *m/z* calcd for $[C_{27}H_{32}NaO_2]^+$ ($[M+Na]^+$): 411.2295, found: 411.2296. IR (*v*/cm^{-1}) 2955, 2855, 1716, 1647, 1508, 1458, 1260, 750, 702. HPLC (OD-H, 0.46*25 cm, 2 μm, hexane/isopropanol = 0.995/0.5, flow 1.0 mL/min, detection at 254 nm) retention time = 7.238 min (major) and 19.134 min (minor).

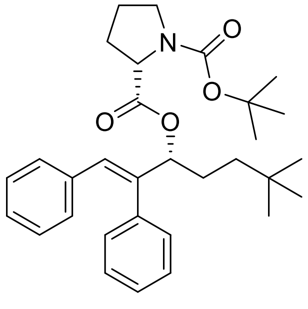

92, 28% yield, 84:16 dr

According to General Procedure C, the reaction between diene **S2** (41.2 mg, 0.2 mmol, 1.0 equiv.), peroxide **O26** (55.2 mg, 0.24 mmol, 1.2 equiv.) and *N*-(*tert*-butoxycarbonyl)-L-proline (47.3, 0.22 mmol, 1.1 equiv) afforded product **92** was obtained as a colorless oil (27.5 mg, 28% yield, 84:16 dr). $[\alpha]^{28.3}_{D}$ 25.35 (*c* 0.4, $CHCl_3$). ^{1}H NMR (400 MHz, Chloroform-*d*) δ 7.28 – 7.22 (m, 3H), 7.19 – 7.14 (m, 2H), 7.05 – 6.96 (m, 3H), 6.88 – 6.81 (m, 2H), 6.61 – 6.52 (m, 1H), 5.47 – 5.39 (m, 1H), 4.36 – 4.18 (m, 1H), 3.61 – 3.26 (m, 3H), 2.24 – 2.06 (m, 1H), 1.94 – 1.74 (m, 3H), 1.54 – 1.46 (m, 2H), 1.36 (s, 1.62H), 1.26 (s, 7.38H), 0.76 (s, .162H), 0.74 (s, 7.38H). ^{13}C NMR (151 MHz, Chloroform-*d*) δ 172.37, 154.02, 140.67, 140.32, 138.16, 137.95, 136.36, 136.13, 129.57, 129.55, 129.50, 129.47, 129.46, 129.44, 128.74, 128.64, 128.00, 127.95, 127.90, 127.68, 127.10, 80.22, 80.07, 80.04, 79.69, 59.49, 59.37, 59.27, 59.02, 46.60, 46.44, 39.53, 39.30, 38.92, 31.09, 30.82, 30.17, 30.13, 30.04, 29.80, 29.38, 29.37, 29.35, 29.24, 28.57, 28.54, 28.47, 28.41, 28.40, 28.32, 28.03, 24.42, 23.61, 23.59. HRMS (ESI) *m/z* calcd for $[C_{31}H_{41}NNaO_4]^+$ ($[M+Na]^+$): 514.2928, found: 514.2928. IR (v/cm^{-1}) 2956, 2868, 1743, 1701, 1541, 1468, 1260, 750, 702. HPLC (IC, 0.46*25 cm, 2 μm, hexane/isopropanol = 97/3, flow 1.0 mL/min, detection at 254 nm) retention time = 8.618 min (major) and 9.760 min (minor).

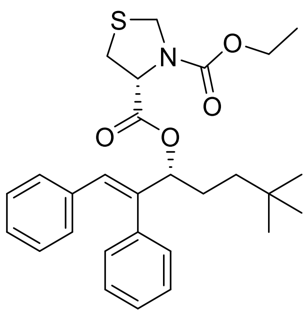

93, 54% yield, 86:14 dr

According to General Procedure C, the reaction between diene **S2** (41.2 mg, 0.2 mmol, 1.0 equiv.), peroxide **O26** (55.2 mg, 0.24 mmol, 1.2 equiv.) and telmesteine (45.1, 0.22 mmol, 1.1 equiv) afforded product **93** was obtained as a colorless oil (51.9 mg, 54% yield, 86:14 dr). $[\alpha]^{28.3}_{D}$ 34.43 (*c* 0.4, $CHCl_3$). ^{1}H NMR (400 MHz, Chloroform-*d*) δ 7.30 – 7.26 (m, 3H), 7.20 – 7.11 (m, 2H), 7.05 – 6.97 (m, 3H), 6.88 – 6.80 (m, 2H), 6.58 (s, 1H), 5.43 (t, *J* = 6.5 Hz, 1H), 4.99 – 4.72 (m, 1H), 4.62 (dd, *J* = 31.5, 8.4 Hz, 1H), 4.42 (dd, *J* = 30.2, 8.4 Hz, 1H), 4.20 – 3.89 (m, 2H), 3.36 – 3.18 (m, 1H), 3.18 – 2.99 (m, 1H), 1.58 – 1.50 (m, 2H), 1.24 – 1.10 (m, 5H), 0.77 (s, 1.55H), 0.75 (s, 7.45H). ^{13}C NMR (101 MHz, Chloroform-*d*) δ 168.80, 168.56, 153.03, 152.95, 139.16, 138.85, 136.79, 135.01, 134.94, 128.34, 128.29, 128.01, 127.94, 127.68, 127.64, 126.87, 126.64, 126.51, 126.05, 125.96, 79.94, 76.20, 61.08, 60.53, 48.31, 46.86, 38.21, 37.98, 33.62, 32.22, 29.05, 29.02, 28.67, 28.24, 27.16, 27.06, 13.60, 13.50. HRMS (ESI) *m/z* calcd for $[C_{28}H_{35}NNaO_4S]^+$ ($[M+Na]^+$): 504.2179, found: 504.2178. IR (v/cm^{-1}) 2955, 2866, 1735, 1716, 1541, 1445, 1407, 1276, 1260, 750, 702. HPLC (IC, 0.46*25 cm, 2 μm, hexane/isopropanol = 97/3, flow 1.0 mL/min, detection at 254 nm) retention time = 9.537 min (major) and 13.167 min (minor).

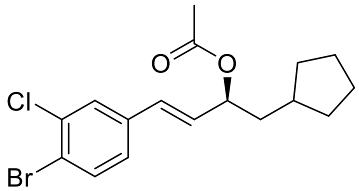

94, 54% yield, 92:8 er

According to General Procedure D, the reaction between diene **S1** (26.0 mg, 0.2 mmol, 1.0 equiv.), peroxide **O2** (90.4 mg, 0.4 mmol, 2.0 equiv.) and acetic acid (18.0, 0.30 mmol, 1.5 equiv) afforded product **94** was obtained as a colorless oil (40.0 mg, 54% yield, 92:8 er). $[\alpha]^{28.3}_{D}$ -83.37 (*c* 0.4, $CHCl_3$). ^{1}H NMR (600 MHz, Chloroform-*d*) δ 7.52 (d, *J* = 8.3 Hz, 1H), 7.44 (d, *J* = 2.0 Hz, 1H), 7.09 (dd, *J* = 8.3, 2.0 Hz, 1H), 6.47 (d, *J* = 15.9 Hz, 1H), 6.12 (dd, *J* = 15.9, 7.2 Hz, 1H), 5.39 (q, *J* = 6.7 Hz, 1H), 2.06 (s, 3H), 1.82 – 1.73 (m, 4H), 1.66 – 1.60 (m, 3H), 1.53 – 1.46 (m, 2H), 1.17 – 1.06 (m, 2H). ^{13}C NMR (151 MHz, Chloroform-*d*) δ 170.58, 137.31, 134.75, 133.81, 130.37, 129.93, 128.18, 126.03, 121.41, 74.10, 40.78, 36.42, 32.77, 25.20, 21.47. HRMS (ESI) *m/z* calcd for $[C_{17}H_{20}BrClNaO_2]^+$ ($[M+Na]^+$): 393.0227, found: 393.0230. IR (*v*/cm^{-1}) 2955, 2865, 1719, 1655, 1541, 1445, 1260, 750, 701. HPLC (OD-H, 0.46*25 cm, 2 μm, hexane/isopropanol = 99.5/0.5, flow 1.0 mL/min, detection at 254 nm) retention time = 7.318 min (minor) and 8.469 min (major).

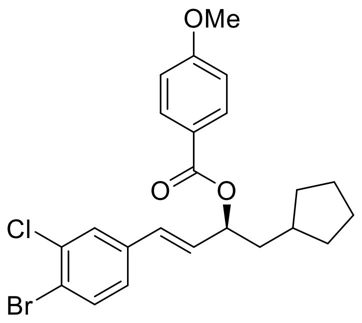

95, 55% yield, 95:5 er

According to General Procedure D, the reaction between diene **S1** (26.0 mg, 0.2 mmol, 1.0 equiv.), peroxide **O2** (90.4 mg, 0.4 mmol, 2.0 equiv.) and 4-methoxybenzoic acid (45.6, 0.30 mmol, 1.5 equiv) afforded product **95** was obtained as a colorless oil (50.8 mg, 55% yield, 95:5 er). $[\alpha]^{28.3}_{D}$ -90.15 (*c* 0.4, $CHCl_3$). ^1H NMR (400 MHz, Chloroform-*d*) δ 7.96 (d, *J* = 8.9 Hz, 2H), 7.45 (d, *J* = 8.3 Hz, 1H), 7.39 (d, *J* = 2.1 Hz, 1H), 7.04 (dd, *J* = 8.3, 2.2 Hz, 1H), 6.86 (d, *J* = 8.9 Hz, 2H), 6.48 (d, *J* = 15.9 Hz, 1H), 6.19 (dd, *J* = 15.9, 6.8 Hz, 1H), 5.57 (q, *J* = 6.3 Hz, 1H), 3.80 (s, 3H), 1.90 – 1.69 (m, 4H), 1.58 – 1.50 (m, 3H), 1.48 – 1.40 (m, 2H), 1.16 – 1.05 (m, 2H). ^{13}C NMR (151 MHz, Chloroform-*d*) δ 165.72, 163.49, 137.40, 134.70, 133.77, 131.76, 130.67, 129.63, 128.21, 126.05, 122.86, 121.32, 113.73, 74.24, 55.56, 40.97, 36.59, 32.97, 25.08. HRMS (ESI) *m/z* calcd for $[C_{23}H_{24}BrClNaO_3]^+$ ($[M+Na]^+$): 485.0490, found: 485.0491. IR (ν/cm^{-1}) 2955, 2866, 1716, 1647, 1541, 1457, 1260, 750, 701. HPLC (IC, 0.46*25 cm, 2 μm, hexane/isopropanol = 99/1, flow 1.0 mL/min, detection at 254 nm) retention time = 16.033 min (major) and 20.138 min (minor).

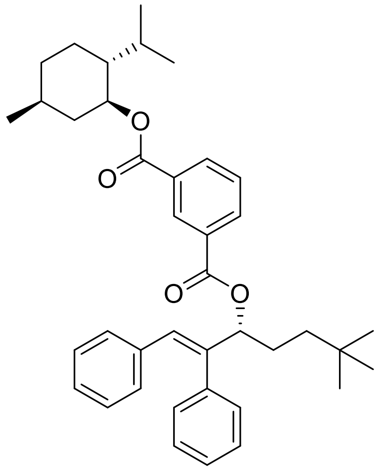

96, 71% yield, 94:6 dr

According to General Procedure C, the reaction between diene **S2** (41.2 mg, 0.2 mmol, 1.0 equiv.), peroxide **O26** (55.2 mg, 0.24 mmol, 1.2 equiv.) and 3-((-)-menthoxycarbonyl)benzoic acid (66.9, 0.22 mmol, 1.1 equiv) afforded product **96** was obtained as a colorless oil (82.4 mg, 71% yield, 94:6 dr). $[\alpha]^{28.3}_{D}$ 43.53 (*c* 0.4, $CHCl_3$). ^1H NMR (400 MHz, Chloroform-*d*) δ 8.73 (s, 1H), 8.25 (d, *J* = 7.7 Hz, 2H), 7.55 (t, *J* = 7.8 Hz, 1H), 7.41 – 7.30 (m, 3H), 7.31 – 7.27 (m, 2H), 7.11 – 7.03 (m, 3H), 6.96 – 6.90 (m, 2H), 6.72 (s, 1H), 5.75 (t, *J* = 6.5 Hz, 1H), 4.98 (td, *J* = 10.9, 4.5 Hz, 1H), 2.17 – 2.11 (m, 1H), 1.99 – 1.90 (m, 1H), 1.81 – 1.70 (m, 4H), 1.60 – 1.52 (m, 3H), 1.43 – 1.30 (m, 2H), 1.20 – 1.08 (m, 2H), 0.97 – 0.92 (m, 3H), 0.92 – 0.90 (m, 3H), 0.85 (s, 9H), 0.82 – 0.77 (m, 3H). ^{13}C NMR (101 MHz, Chloroform-*d*) δ 165.28, 165.14, 140.71, 138.03, 136.07, 133.87, 133.61, 131.36, 130.97, 130.71, 129.38, 129.36, 128.89, 128.70, 128.59, 127.87, 127.58, 126.98, 80.44, 75.30, 47.20, 40.97, 39.34,

34.31, 31.47, 30.13, 29.70, 29.31, 28.33, 23.62, 22.05, 20.76, 16.52. HRMS (ESI) *m/z* calcd for $[C_{39}H_{48}NaO_4]^+$ ($[M+Na]^+$): 603.3445, found: 603.3442. IR (v/cm^{-1}) 2957, 2869, 1720, 1608, 1458, 1260, 720, 702. HPLC (AD-H, 0.46*25 cm, 2 μm, hexane/isopropanol = 99/1, flow 1.0 mL/min, detection at 254 nm) retention time = 6.044 min (major) and 7.632 min (minor).

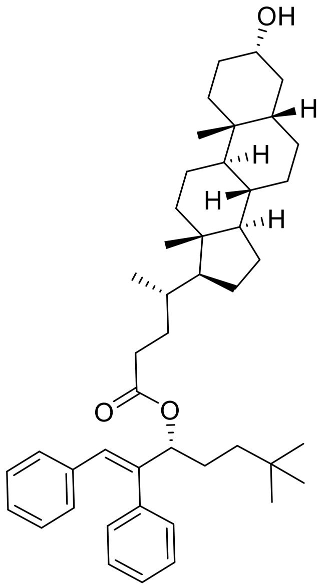

97, 45% yield, 95:5 dr

According to General Procedure C, the reaction between diene **S2** (42.8 mg, 0.4 mmol, 2.0 equiv.), peroxide **O26** (92.0 mg, 0.4 mmol, 2.0 equiv.) and lithocholic acid (75.4, 0.20 mmol, 1.0 equiv) afforded product **97** was obtained as a colorless oil (58.7 mg, 45% yield, 95:5 dr). $[\alpha]^{28.3}_{D}$ 52.53 (*c* 0.4, $CHCl_3$). ^{1}H NMR (400 MHz, Chloroform-*d*) δ 7.33 – 7.31 (m, 3H), 7.25 – 7.20 (m, 2H), 7.09 – 7.07 (m, 3H), 6.94 – 6.87 (m, 2H), 6.61 (s, 1H), 5.48 (t, *J* = 6.5 Hz, 1H), 3.62 (td, *J* = 10.8, 5.3 Hz, 1H), 2.48 – 2.35 (m, 1H), 2.33 – 2.24 (m, 1H), 1.95 (d, *J* = 12.2 Hz, 1H), 1.88 – 1.76 (m, 4H), 1.65 (d, *J* = 15.2 Hz, 2H), 1.58 – 1.48 (m, 4H), 1.43 – 1.27 (m, 11H), 1.20 – 0.95 (m, 9H), 0.94 – 0.90 (m, 6H), 0.82 (s, 9H), 0.62 (s, 3H). ^{13}C NMR (101 MHz, Chloroform-*d*) δ 173.61, 141.20, 138.24, 136.29, 129.36, 129.33, 128.59, 128.39, 127.86, 127.46, 126.85, 79.04, 71.90, 56.49, 56.01, 42.75, 42.10, 40.44, 40.18, 39.29, 36.47, 35.85, 35.33, 34.58, 31.62, 31.08, 30.56, 30.07, 29.72, 29.31, 28.26, 28.23, 27.20, 26.42, 24.20, 23.39, 20.83, 18.28, 12.07. HRMS (ESI) *m/z* calcd for $[C_{45}H_{64}NaO_3]^+$ ($[M+Na]^+$): 675.4748, found: 675.4747. IR (*v*/cm^{-1}) 2937, 2853, 1717, 1637, 1541, 1458, 1260, 750, 696. HPLC (IC, 0.46*25 cm, 2 μm, hexane/isopropanol = 95/5, flow 1.0 mL/min, detection at 254 nm) retention time = 9.854 min (major) and 14.733 min (minor).

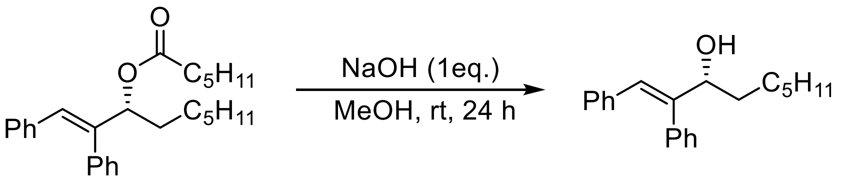

63, 0.1 mmol

98, 98% yield, 96:4 er

In a flame-dried Schlenk tube, **63** (96:4 er by chiral HPLC, 39.2 mg, 0.1 mmol) and NaOH (4 mg, 0.1 mmol, 1 equiv) were dissolved in MeOH (0.4 mL, 0.25 M), and the mixture was stirred at room temperature for 24 h. After the reaction completion, the solvent was evaporated under reduced pressure. The residue was purified by flash column chromatography on silica gel to afford the product **98** as a

colorless oil (28.8 mg, 98% yield, 96:4 er). $[\alpha]^{28.3}_{D}$ -42.32 (*c* 0.4, $CHCl_3$).[9]

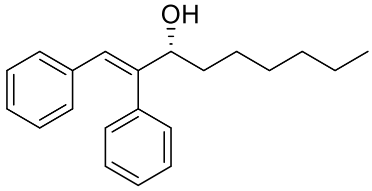

98, 98% yield, 96:4 er

^{1}H NMR (600 MHz, Chloroform-*d*) δ 7.35 – 7.29 (m, 3H), 7.20 – 7.17 (m, 2H), 7.11 – 7.06 (m, 3H), 6.97 – 6.85 (m, 2H), 6.65 (s, 1H), 4.45 (t, *J* = 6.0 Hz, 1H), 1.59 – 1.51 (m, 1H), 1.51 – 1.43 (m, 2H), 1.40 – 1.32 (m, 1H), 1.31 – 1.18 (m, 6H), 0.88 – 0.84 (m, 3H). ^{13}C NMR (151 MHz, Chloroform-*d*) δ 145.05, 138.54, 136.66, 129.35, 129.31, 128.77, 128.00, 127.44, 126.90, 126.78, 77.55, 35.57, 31.89, 29.27, 25.77, 22.70, 14.18. HRMS (ESI) *m/z* calcd for $[C_{21}H_{26}NaO]^+$ ($[M+Na]^+$): 317.1876, found: 317.1880. IR (*v*/cm^{-1}) 3005, 2940, 2850, 1654, 1541, 1445, 1260, 750, 703. HPLC (OD-H, 0.46*25 cm, 2 μm, hexane/isopropanol = 95/5, flow 1 mL/min, detection at 254 nm) retention time = 6.113 min (minor) and 6.893 min (major).

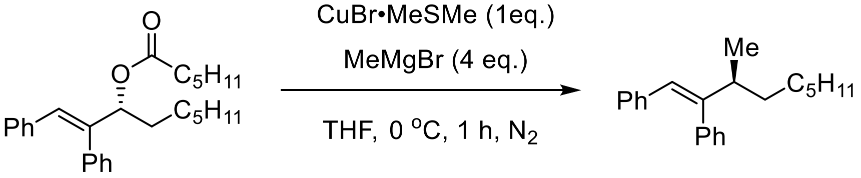

63, 0.1 mmol **99**, 78% yield, 95:5 er

To an ice-cold suspension of CuBr·Me_2S (20.5 mg, 0.1 mmol, 1 equiv) in THF (1 mL) was added MeMgBr (0.13 mL, 3 M in THF, 0.4 mmol, 4 equiv) dropwise. The resulting mixture was stirred at 0 ^{o}C for 30 min and **63** (96:4 er by chiral HPLC, 39.2 mg, 0.1 mmol) in THF (1 mL) was added dropwise. After 1 h at 0 ^{o}C, saturated NH_4Cl was added to the solution and the resulting mixture was extracted with EtOAc three times. The combined extracts were dried over $MgSO_4$ and concentrated to afford an oil, which was purified by chromatography on silica gel with hexane/EtOAc to afford product **99** as a colorless oil (22.4 mg, 78% yield, 95:5 er). $[\alpha]^{28.3}_{D}$ -47.95 (*c* 0.4, $CHCl_3$).[10]

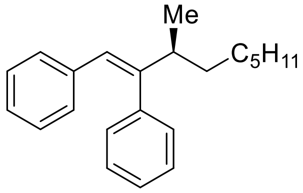

99, 78% yield, 95:5 er

^{1}H NMR (400 MHz, Chloroform-*d*) δ 7.34 – 7.26 (m, 3H), 7.13 – 6.99 (m, 5H), 6.89 – 6.80 (m, 2H), 6.38 (s, 1H), 2.59 – 2.46 (m, 1H), 1.53 – 1.25 (m, 10H), 1.10 (d, *J* = 6.8 Hz, 3H), 0.91 – 0.80 (m, 3H). ^{13}C NMR (101 MHz, Chloroform-*d*) δ 148.18, 141.02, 137.58, 129.03, 128.98, 128.33, 127.76, 126.67, 125.97, 125.62, 43.32, 35.35, 31.90, 29.48, 27.54, 22.70, 20.00, 14.12. HRMS (DART) *m/z* calcd for $[C_{22}H_{32}N]^+$ ($M+NH_4^+$): 310.2529, found: 310.2528. IR (*v*/cm^{-1}) 2958, 2926, 2856, 1770, 1374, 1245, 1056, 751, 699. HPLC (OD-H, 0.46*25 cm, 5 μm, hexane/isopropanol = 100/0, flow 0.25 mL/min, detection at 254 nm) retention time = 25.221 min (major) and 26.362 min (minor).

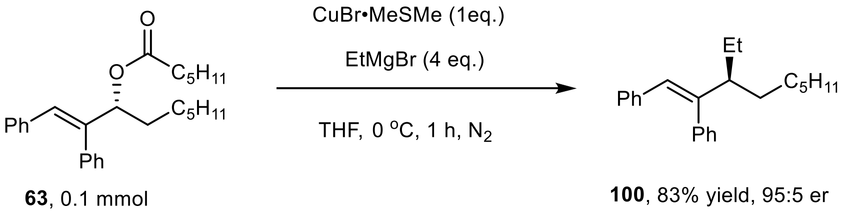

To an ice-cold suspension of CuBr ·Me_2S (20.5 mg, 0.1 mmol, 1 equiv) in THF (1 mL) was added EtMgBr (0.4 mL, 1 M in THF, 0.4 mmol, 4 equiv) dropwise. The resulting mixture was stirred at 0 °C for 30 min and **63** (96:4 er by chiral HPLC, 39.2 mg, 0.1 mmol) in THF (1 mL) was added dropwise. After 1 h at 0 °C, saturated NH_4Cl was added to the solution and the resulting mixture was extracted with EtOAc three times. The combined extracts were dried over $MgSO_4$ and concentrated to afford an oil, which was purified by chromatography on silica gel with hexane/EtOAc to afford product **100** as a colorless oil (47.1 mg, 83% yield, 95:5 er). $[\alpha]^{28.3}_D$ -43.20 (*c* 0.4, $CHCl_3$).[10]

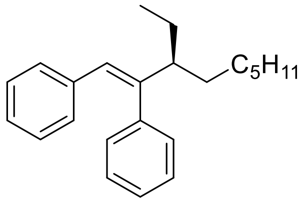

100, 83% yield, 95:5 er

^{1}H NMR (400 MHz, Chloroform-*d*) δ 7.28 – 7.19 (m, 3H), 7.05 – 6.93 (m, 5H), 6.77 (dd, *J* = 8.0, 1.8 Hz, 2H), 6.29 (s, 1H), 2.27 – 2.15 (m, 1H), 1.40 – 1.18 (m, 12H), 0.90 (t, *J* = 7.3 Hz, 3H), 0.83 – 0.78 (m, 3H). ^{13}C NMR (101 MHz, Chloroform-*d*) δ 144.72, 139.89, 136.51, 127.99, 127.97, 127.30, 126.72, 126.47, 125.63, 124.94, 50.39, 32.34, 30.86, 28.51, 26.57, 25.31, 21.67, 13.08, 11.09. HRMS (DART) *m/z* calcd for $[C_{23}H_{31}]^+$ (M+H$^+$): 307.2420, found: 307.2421. IR (ν/cm^{-1}) 2958, 2925, 2854, 1637, 1542, 1458, 1260, 750, 700. HPLC (OD-H, 0.46*25 cm, 2 μm, hexane/isopropanol = 100/0, flow 0.25 mL/min, detection at 254 nm) retention time = 22.204 min (minor) and 22.557 min (major).

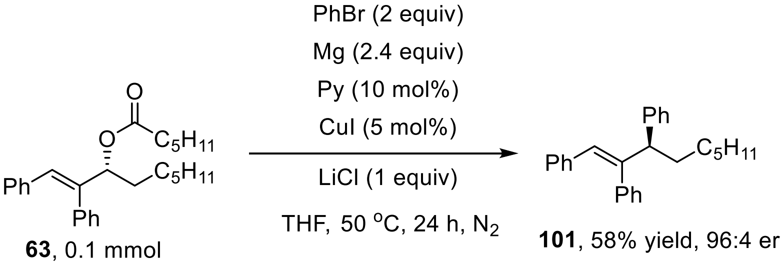

In a flame-dried Schlenk tube, CuI (1 mg, 0.005 mmol, 5 mol%), Mg (5.8 mg, 0.24 mmol, 2.4 equiv), pyridine (0.79 mg, 0.01 mmol, 10 mol%), LiCl (4.2 mg, 0.1 mmol, 1 equiv), **63** (96:4 er by chiral HPLC, 39.2 mg, 0.1 mmol) and PhBr (31.2 mg, 0.2 mmol, 2 equiv) were dissolved in THF (0.5 mL, 0.2 M), and the mixture was stirred at 50 ℃ for 24 h. After the reaction completion, the solvent was evaporated under reduced pressure. The residue was purified by flash column chromatography on silica gel to afford the product **101** as a colorless oil (20.5 mg, 58% yield, 96:4 er). $[\alpha]^{28.3}_D$ -39.54 (*c* 0.4, $CHCl_3$).

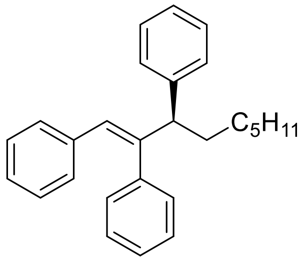

101, 58% yield, 96:4 er

^{1}H NMR (400 MHz, Chloroform-*d*) δ 7.28 – 7.02 (m, 11H), 6.94 – 6.74 (m, 4H), 6.56 (s, 1H), 3.64 (t, *J* = 7.6 Hz, 1H), 1.98 – 1.87 (m, 1H), 1.85 – 1.75 (m, 1H), 1.44 – 1.23 (m, 8H), 0.86 (t, *J* = 7.0 Hz, 3H). ^{13}C NMR (101 MHz, Chloroform-*d*) δ 146.03, 143.09, 140.84, 137.29, 129.05, 128.99, 128.42, 128.13, 128.05, 127.77, 126.74, 126.68, 126.22, 126.15, 55.20, 33.38, 31.78, 29.37, 27.89, 22.66, 14.08. IR (ν/cm^{-1}) 2995, 2926, 2853, 1770, 1374, 1245, 1056, 751, 700. HRMS (DART) *m/z* calcd for $[C_{27}H_{34}N]^+$ $(M+NH_4^+)$: 372.2686, found 372.2684. HPLC (OD-H, 0.46*25 cm, 10 μm, hexane/isopropanol = 100/0, flow 0.25 mL/min, detection at 254 nm) retention time = 41.351 min (minor) and 48.694 min (major).

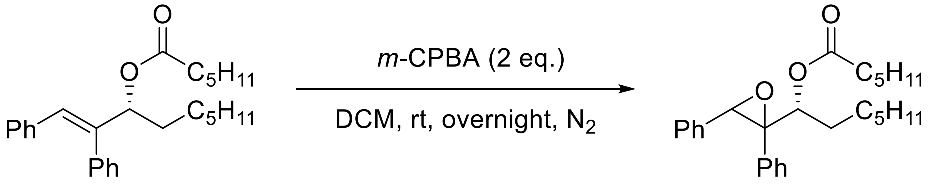

63, 0.1 mmol

102, 75% yield, 2.9:1 dr
for major 96:4 er
for minor 96:4 er

A solution of **63** (96:4 er by chiral HPLC, 39.2 mg, 0.1 mmol) in DCM (2 mL) was treated with *m*-CPBA (34.5 mg, 0.2 mmol, 2 equiv) at 0 °C. The mixture was stirred overnight at room temperature. Then, the reaction was diluted with DCM, the organic layers were washed with $NaHCO_3$(aq.), Na_2SO_3 (aq.), and brine, dried over Na_2SO_4 and evaporated under reduced pressure. The residue was purified by flash column chromatography on silica gel to afford the product **102** as a pale yellow oil (61.2 mg, 75% yield, 2.9:1 dr, for major 96:4 er, for minor 96:4 er). $[\alpha]^{28.3}_{D}$ 32.39 (*c* 0.4, $CHCl_3$).[11]

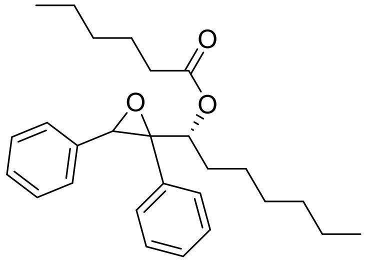

102, 75% yield, 2.9:1 dr
for major 96:4 er
for minor 96:4 er

^{1}H NMR (400 MHz, Chloroform-*d*) δ 7.24 – 7.11 (m, 5H), 7.11 – 7.04 (m, 3H), 6.99 – 6.90 (m, 2H), 5.22 (dd, *J* = 9.3, 4.1 Hz, 0.23H), 4.93 (dd, *J* = 9.6, 3.7 Hz, 0.77H), 4.37 (s, 0.77H), 4.17 (s, 0.23H), 2.43 (t, *J* = 7.5 Hz, 0.46H), 2.37 (t, *J* = 7.5 Hz, 1.54H), 1.75 – 1.64 (m, 3H), 1.56 – 1.43 (m, 1H), 1.39 – 1.19 (m, 12H), 0.94 – 0.82 (m, 6H). ^{13}C NMR (101 MHz, Chloroform-*d*) δ 173.67, 173.39, 134.83, 134.54, 134.40, 133.30, 129.02, 128.36, 127.74, 127.70, 127.63, 127.60, 127.56, 127.48, 126.48,

126.33, 76.79, 75.58, 69.76, 68.06, 63.62, 62.33, 34.50, 34.45, 31.69, 31.65, 31.40, 31.35, 30.50, 29.89, 29.72, 29.00, 25.68, 25.42, 24.79, 24.75, 22.55, 22.37, 14.05, 13.93. HRMS (ESI) *m/z* calcd for $[C_{27}H_{36}NaO_3]^+$ ($[M+Na]^+$): 431.2557, found: 431.2557. IR (ν/cm^{-1}) 3063, 3032, 2956, 2929, 2859, 1736, 1458, 1377, 1167, 751, 699. HPLC (IC, 0.46*25 cm, 2 μm, hexane/isopropanol = 99.5/0.5, flow 1.0 mL/min, detection at 254 nm) retention time = 24.125 min (major), 25.452 min (minor) and 25.083 min (minor) 25.452 min (major).

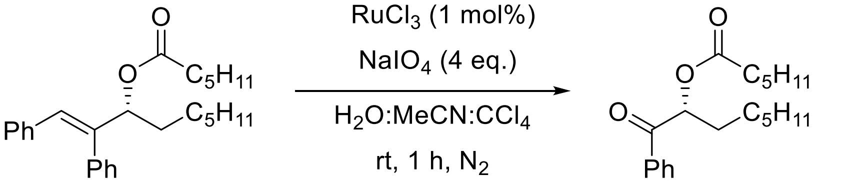

63, 0.1 mmol

103, 95% yield, 96:4 er

To a solution of **63** (96:4 er by chiral HPLC, 39.2 mg, 0.1 mmol) and $NaIO_4$ (85.6 mg, 0.4 mmol, 4equiv) in CCl_4 (0.2 mL), CH_3CN (0.2 mL) and H_2O (0.3 mL), was added $RuCl_3$ (0.27 mg, 0.001 mmol, 1 mol%) and the mixture was vigorously stirred at rt for 1 h. Water was added and the aqueous layer was extracted with DCM. The organic layer was dried over $MgSO_4$ and evaporated under reduced pressure. Water was added and the aqueous layer was extracted with CH_2Cl_2. The organic layer was dried over MgSO4 and evaporated under reduced pressure. The residue was purified by flash column chromatography on silica gel to afford the product **103** as a colorless oil (60.4 mg, 95% yield, 96:4 er). $[\alpha]^{28.3}_D$ 29.94 (*c* 0.4, $CHCl_3$).[12]

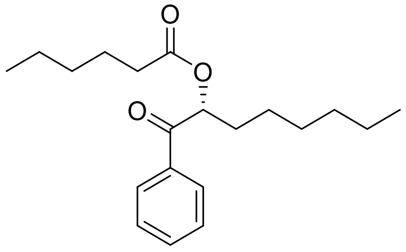

103, 95% yield, 96:4 er

^{1}H NMR (400 MHz, Chloroform-*d*) δ 7.87 (d, *J* = 7.4 Hz, 2H), 7.51 (t, *J* = 7.4 Hz, 1H), 7.40 (t, *J* = 7.7 Hz, 2H), 5.78 (dd, *J* = 8.3, 4.6 Hz, 1H), 2.34 (t, *J* = 7.5 Hz, 2H), 1.83 – 1.71 (m, 2H), 1.64 – 1.51 (m, 2H), 1.44 – 1.32 (m, 2H), 1.27 – 1.16 (m, 10H), 0.86 – 0.73 (m, 6H). ^{13}C NMR (101 MHz, Chloroform-*d*) δ 195.92, 172.52, 127.71, 127.36, 133.86, 132.41, 74.08, 32.93, 30.52, 30.33, 30.20, 27.83, 24.48, 23.53, 21.49, 21.29, 13.00, 12.87. HRMS (ESI) *m/z* calcd for $[C_{20}H_{30}NaO_3]^+$ ($[M+Na]^+$): 341.2087, found: 341.2088. IR (ν/cm^{-1}) 2959, 2858, 1637, 1541, 1458, 1260, 750, 695. HPLC (OD-H, 0.46*25 cm, 2 μm, hexane/isopropanol = 98/2, flow 1.0 mL/min, detection at 254 nm) retention time = 4.777 min (major) and 7.807 min (minor).

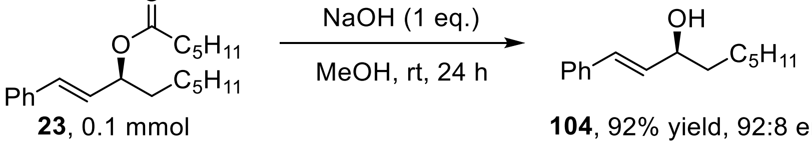

23, 0.1 mmol

104, 92% yield, 92:8 er

In a flame-dried Schlenk tube, **23** (93:7 er by chiral HPLC, 31.6 mg, 0.1 mmol) and NaOH (4 mg, 0.1 mmol, 1 equiv) were dissolved in MeOH (0.4 mL, 0.25 M), and the mixture was stirred at room

temperature for 24 h. After the reaction completion, the solvent was evaporated under reduced pressure. The residue was purified by flash column chromatography on silica gel to afford the product **104** as a white solid (40.1 mg, 92% yield, 92:8 er). $[\alpha]^{28.3}_D$ 12.97 (*c* 0.4, $CHCl_3$). Mp 52.0-53.5 °C.[9]

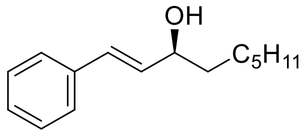

104, 92% yield, 92:8 er

^{1}H NMR (400 MHz, Chloroform-*d*) δ 7.33 – 7.28 (m, 2H), 7.23 (t, *J* = 7.5 Hz, 2H), 7.18 – 7.13 (m, 1H), 6.48 (d, *J* = 16.0 Hz, 1H), 6.14 (dd, *J* = 15.9, 6.8 Hz, 1H), 4.18 (q, *J* = 6.8, 6.4 Hz, 1H), 1.69 (d, *J* = 16.5 Hz, 1H), 1.61 – 1.45 (m, 2H), 1.39 – 1.16 (m, 8H), 0.86 – 0.74 (m, 3H). ^{13}C NMR (101 MHz, Chloroform-*d*) δ 136.78, 132.65, 130.20, 128.58, 127.61, 126.46, 73.14, 37.41, 31.83, 29.28, 25.45, 22.64, 14.11. HRMS (ESI) *m/z* calcd for $[C_{15}H_{22}NaO_2]^+$ ($[M+Na]^+$): 241.1563, found: 241.1571. IR (v/cm^{-1}) 2955, 2959, 1762, 1647, 1541, 1445, 1224, 750, 700. IR (v/cm^{-1}) 3005, 2940, 2850, 1654, 1541, 1445, 1260, 750, 703. HPLC (OD-H, 0.46*25 cm, 2 µm, hexane/isopropanol = 95/5, flow 1 mL/min, detection at 254 nm) retention time = 9.681 min (minor) and 15.658 min (major).

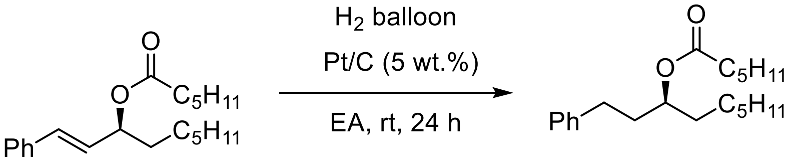

23, 0.1 mmol **105**, 90% yield, 93:7 er

Pt/C (5 wt.%, 11 mg) was added to a solution of **23** (93:7 er by chiral HPLC, 31.6 mg, 0.1 mmol) in EA (0.7 mL). The mixture was stirred for 24 h at room temperature under hydrogen (1-2 atmospheres). The suspension was filtered through a short path of Celite®. The organic layer was extracted with DCM, dried over $MgSO_4$ and evaporated under reduced pressure. Purification by flash chromatography on silica gel gave **105** as a colorless oil (57.2 mg, 90% yield, 93:7 er). $[\alpha]^{28.3}_D$ -5.84 (*c* 0.4, $CHCl_3$).[13]

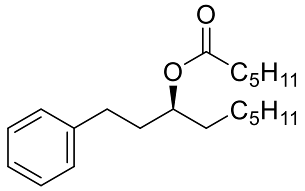

105, 90% yield, 93:7 er

^{1}H NMR (400 MHz, Chloroform-*d*) δ 7.19 (d, *J* = 7.2 Hz, 2H), 7.13 – 7.07 (m, 3H), 4.91 – 4.83 (m, 1H), 2.61 – 2.46 (m, 2H), 2.21 (t, *J* = 7.5 Hz, 2H), 1.85 – 1.71 (m, 2H), 1.63 – 1.42 (m, 4H), 1.30 – 1.13 (m, 12H), 0.88 – 0.73 (m, 6H). ^{13}C NMR (101 MHz, Chloroform-*d*) δ 172.70, 140.78, 127.37, 127.28, 124.84, 72.63, 34.91, 33.62, 33.16, 30.78, 30.71, 30.35, 28.15, 24.17, 23.83, 21.54, 21.33, 13.04, 12.91. HRMS (ESI) *m/z* calcd for $[C_{21}H_{34}NaO_2]^+$ ($[M+Na]^+$): 341.2451, found: 341.2451. IR (v/cm^{-1}) 3027, 2956, 2928, 2858, 1733, 1637, 1541, 1457, 1260, 750, 699. HPLC (OD-H, 0.46*25 cm, 2 µm, hexane/isopropanol = 99.5/0.5, flow 1.0 mL/min, detection at 254 nm) retention time = 6.074 min (major) and 6.925 min (minor).

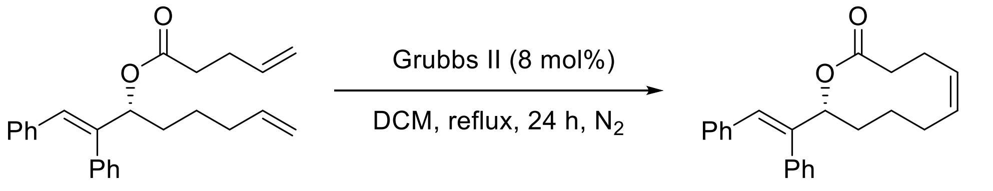

75, 0.1 mmol

106, 65% yield, 96:4 er

In a flame-dried Schlenk tube, Grubbs II (8 mol%) and **75** (97:3 er by chiral HPLC, 36.0 mg, 0.1 mmol) were dissolved in DCM (20 mL, 0.005 M) under a nitrogen atmosphere, and the mixture was reflux for 24 h. After the reaction completion, the solvent was evaporated under reduced pressure. The residue was purified by flash column chromatography on silica gel to afford the product **106** as a colorless oil (45.8 mg, 65% yield, 96:4 er). $[\alpha]^{28.3}_{D}$ 39.18 (*c* 0.4, $CHCl_3$).[14]

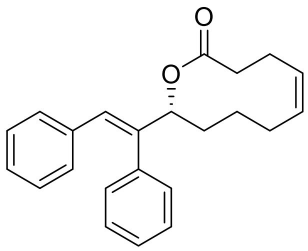

106, 65% yield, 96:4 er

^{1}H NMR (600 MHz, Chloroform-*d*) δ 7.33 – 7.27 (m, 3H), 7.24 – 7.20 (m, 2H), 7.13 – 7.06 (m, 3H), 6.98 – 6.92 (m, 2H), 6.79 (s, 1H), 5.80 (s, 1H), 5.52 (td, *J* = 11.0, 5.4 Hz, 1H), 5.44 – 5.37 (m, 1H), 3.04 – 2.87 (m, 1H), 2.86 – 2.75 (m, 1H), 2.75 – 2.66 (m, 1H), 2.41 – 2.32 (m, 1H), 2.12 – 2.03 (m, 1H), 2.03 – 1.95 (m, 1H), 1.93 – 1.84 (m, 1H), 1.84 – 1.76 (m, 1H), 1.43 – 1.30 (m, 2H). ^{13}C NMR (151 MHz, Chloroform-*d*) δ 171.51, 138.41, 136.60, 132.56, 129.33, 129.19, 128.86, 127.98, 127.71, 127.08, 126.74, 125.91, 100.00, 35.08, 26.01, 22.62. HRMS (ESI) *m/z* calcd for $[C_{23}H_{24}NaO_2]^+$ ($[M+Na]^+$): 355.1669, found: 355.1669. IR (ν/cm^{-1}) 2955, 2959, 1762, 1647, 1541, 1445, 1224, 750, 700. HPLC (IC, 0.46*25 cm, 2 μm, hexane/isopropanol = 99/1, flow 1 mL/min, detection at 254 nm) retention time = 8.009 min (major) and 9.545 min (minor).

J. Single crystal data

X-ray diffractions for single crystals of **3**, **19**, **65**, **[(S)-L1]$_2$(Cu)$_2$(OTf)$_2$**, **[(R,R)-L2]$_2$(Cu)$_2$(OTf)$_2$**, **85 and 89** were carried out on Rikagu Synergy Custom (Liquid MetalJet D2 Plus) diffractometer using Ga Kα radiation (λ = 1.3405 Å). Data collection and unit cell refinement were executed by using CrysAlisPro software. Data processing and absorption correction, giving minimum and maximum transmission factors, were accomplished with CrysAlisPro. The structure was solved with the SHELXT and refined with the SHELXL using least-squares minimisation. All non-hydrogen atoms were refined with anisotropic displacement parameters. All carbon bound hydrogen atom positions were determined by geometry and refined by a riding model. CCDC 2094205 **3**, 2094206 **19**, 2101770 **65**, 2094208 **[(S)-L1]$_2$(Cu)$_2$(OTf)$_2$,** 2094209 **[(R,R)-L2]$_2$(Cu)$_2$(OTf)$_2$,** 2094207 **85** and 2094210 **89** contain the supplementary crystallographic data. Crystal data and structure refinements of **3**, **19**, **[(S)-L1]$_2$(Cu)$_2$(OTf)$_2$**, **[(R,R)-L2]$_2$(Cu)$_2$(OTf)$_2$**, **85 and 89** are listed in **Table S5**, **Table S6**, **Table S7**, **Table S8**, **Table S9**, **Table S10** and Table **S11**. These data can be obtained free of charge from the Cambridge Crystallographic Data Centre via https://www.ccdc.cam.ac.uk/

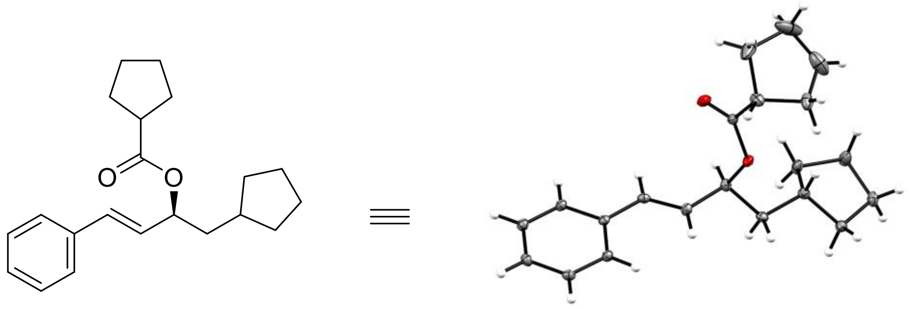

3

Single crystal for **3**
CCDC 2094205

Table S5. Crystal data and structure refinement for **3**.

Identification code	**3**		
Empirical formula	$C_{21}H_{27}O_2$		
Formula weight	311.42		
Temperature (K)	100.00(11)		
Wavelength (Å)	1.3405		
Crystal system	monoclinic		
Space group	*P*2$_1$		
Unit cell dimensions (Å, °)	*a* = 9.0735(2)	α =	90
	b = 6.06060(10)	β =	95.872(2)
	c = 16.0530(4)	γ =	90
Volume (Å)	878.14(3)		

Z	2
Calculated density (g cm^{-3})	1.178
Absorption coefficient (mm^{-1})	0.365
F_{000}	338
Crystal size (mm^3)	0.26 × 0.21 × 0.17
θ range for data collection (°)	2.406 to 56.874
Miller index ranges	$-11 \le h \le 11$, $-7 \le k \le 7$, $-20 \le l \le 20$
Reflections collected	21182
Independent reflections	3593 [R_{int} = 0.0713]
Completeness to θ_{max} (%)	0.997
Max. and min. transmission	0.59698 and 1.00000
Refinement method	Full-matrix least-squares on F^2
Data / restraints / parameters	3593 / 19 / 208
Goodness-of-fit on F^2	1.111
Final R indices [$I > 2\sigma(I)$]	$R1$ = 0.0793, $wR2$ = 0.2181
R indices (all data)	$R1$ = 0.0819, $wR2$ = 0.2201
Largest diff. peak and hole (e Å^{-3})	0.775 and -0.413
Absolute structure parameter	.36(17)

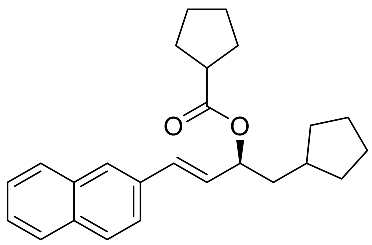

19

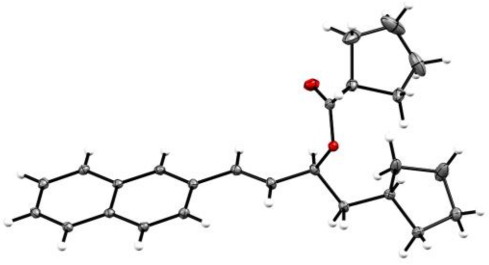

Single crystal for **19**
CCDC 2094206

Table S6. Crystal data and structure refinement for **19**.

Identification code	**19**	
Empirical formula	$C_{25}H_{30}O_2$	
Formula weight	362.49	
Temperature (K)	100.00(13)	
Wavelength (Å)	1.3405	
Crystal system	monoclinic	
Space group	$P2_1$	
Unit cell dimensions (Å, °)	a = 8.8532(2)	α = 90
	b = 6.04590(10)	β = 93.009(2)
	c = 18.7697(3)	γ = 90
Volume (Å)	1003.27(3)	

Z	2
Calculated density (g cm^{-3})	1.200
Absorption coefficient (mm^{-1})	0.365
F_{000}	392
Crystal size (mm^3)	0.22 × 0.18 × 0.11
θ range for data collection (°)	2.049 to 60.546
Miller index ranges	$-10 \le h \le 11$, $-7 \le k \le 7$, $-24 \le l \le 24$
Reflections collected	26298
Independent reflections	4508 [R_{int} = 0.0759]
Completeness to θ_{max} (%)	0.988
Max. and min. transmission	0.65236 and 1.00000
Refinement method	Full-matrix least-squares on F^2
Data / restraints / parameters	4508 / 19 / 244
Goodness-of-fit on F^2	1.095
Final R indices [$I > 2\sigma(I)$]	$R1$ = 0.0622, $wR2$ = 0.1614
R indices (all data)	$R1$ = 0.0674, $wR2$ = 0.1645
Largest diff. peak and hole (e Å^{-3})	0.553 and -0.399
Absolute structure parameter	.19(18)

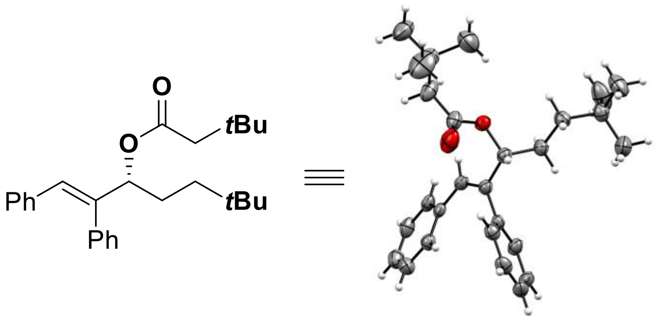

Single crystal for **65**
CCDC 2101770

Table S7. Crystal data and structure refinement for **65**.

Identification code	**65**	
Empirical formula	$C_{27}H_{36}O_2$	
Formula weight	392.56	
Temperature (K)	150.00(10)	
Wavelength (Å)	1.54184	
Crystal system	orthorhombic	
Space group	$P2_12_12_1$	
Unit cell dimensions (Å, °)	a = 8.8277(2)	α = 90
	b = 9.0862(2)	β = 90
	c = 30.8412(10)	γ = 90

Volume (Å)	2473.78(11)
Z	4
Calculated density (g cm^{-3})	1.054
Absorption coefficient (mm^{-1})	0.495
F_{000}	856
Crystal size (mm^3)	0.21 × 0.17 × 0.14
θ range for data collection (°)	5.074 to 72.175
Miller index ranges	$-10 \le h \le 6$, $-10 \le k \le 11$, $-37 \le l \le 38$
Reflections collected	16234
Independent reflections	4764 [R_{int} = 0.0374]
Completeness to θ_{max} (%)	0.987
Max. and min. transmission	0.46222 and 1.00000
Refinement method	Full-matrix least-squares on F^2
Data / restraints / parameters	4764 / 0 / 268
Goodness-of-fit on F^2	1.051
Final *R* indices [$I > 2\sigma(I)$]	$R1$ = 0.0506, $wR2$ = 0.1415
R indices (all data)	$R1$ = 0.0582, $wR2$ = 0.1464
Largest diff. peak and hole (e $Å^{-3}$)	0.286 and -0.214
Absolute structure parameter	.06(14)

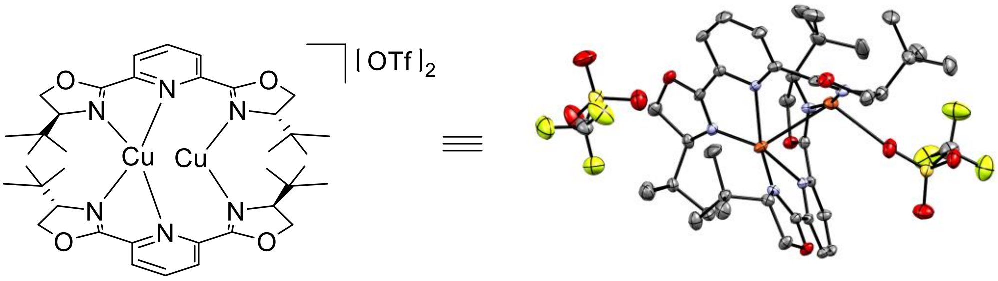

Copper Complex 1
[(*S*)-L1]$_2$(Cu)$_2$(OTf)$_2$
CCDC 2094208

Table S8. Crystal data and structure refinement for **[(S)-L1]$_2$(Cu)$_2$(OTf)$_2$**.

Identification code	**[(S)-L1]$_2$(Cu)$_2$(OTf)$_2$**		
Empirical formula	$C_{40}H_{54}Cu_2F_6N_6O_{10}S_2$		
Formula weight	1084.09		
Temperature (K)	296.8(3)		
Wavelength (Å)	1.3405		
Crystal system	orthorhombic		
Space group	$P2_12_12$		
Unit cell dimensions (Å, °)	*a* = 30.2598(5)	α =	90
	b = 16.2567(4)	β =	90

	c = 10.3123(2)	γ = 90
Volume (Å)	5072.87(18)	
Z	4	
Calculated density (g cm^{-3})	1.419	
Absorption coefficient (mm^{-1})	5.471	
F_{000}	2240	
Crystal size (mm^3)	0.26 × 0.11 × 0.10	
θ range for data collection (°)	2.683 to 50.776	
Miller index ranges	$-34 \le h \le 28$, $-18 \le k \le 18$, $-11 \le l \le 11$	
Reflections collected	51318	
Independent reflections	8135 [R_{int} = 0.0635]	
Completeness to θ_{max} (%)	0.994	
Max. and min. transmission	0.67119 and 1.00000	
Refinement method	Full-matrix least-squares on F^2	
Data / restraints / parameters	8135 / 0 / 601	
Goodness-of-fit on F^2	1.073	
Final R indices [$I > 2\sigma(I)$]	$R1$ = 0.0640, $wR2$ = 0.1244	
R indices (all data)	$R1$ = 0.1045, $wR2$ = 0.1388	
Largest diff. peak and hole (e Å^{-3})	0.447 and -0.283	
Absolute structure parameter	.048(3)	

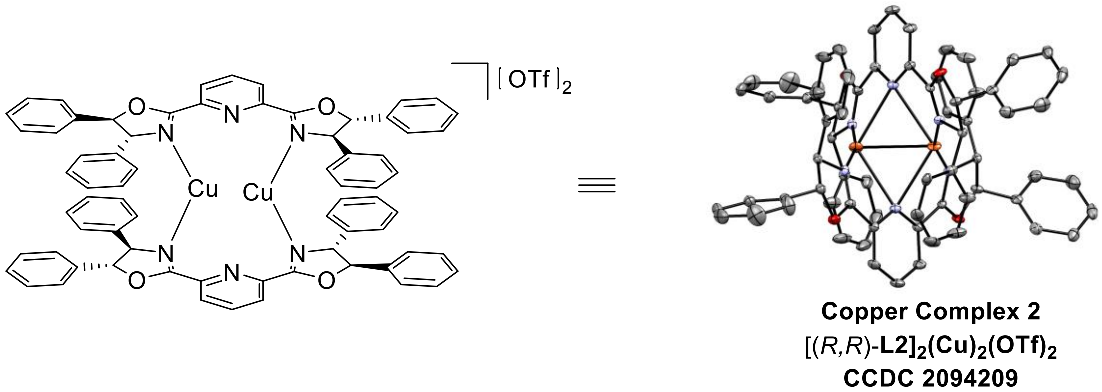

Copper Complex 2
[(*R,R*)-L2]$_2$(Cu)$_2$(OTf)$_2$
CCDC 2094209

Table S9. Crystal data and structure refinement for **[(R,R)-L2]$_2$(Cu)$_2$(OTf)$_2$**.

Identification code	**[(R,R)-L2]$_2$(Cu)$_2$(OTf)$_2$**	
Empirical formula	$C_{36.50}H_{28}ClCuF_3N_3O_5S$	
Formula weight	776.67	
Temperature (K)	99.99(13)	
Wavelength (Å)	1.3405	
Crystal system	orthorhombic	
Space group	$P2_12_12$	
Unit cell dimensions (Å, °)	a = 18.4767(2)	α = 90

	b = 16.2417(2)	β = 90
	c = 11.29770(10)	γ = 90
Volume (Å)	3390.36(6)	
Z	4	
Calculated density (g cm^{-3})	1.522	
Absorption coefficient (mm^{-1})	4.703	
F_{000}	1588	
Crystal size (mm^3)	0.34 × 0.30 × 0.25	
θ range for data collection (°)	3.150 to 60.561	
Miller index ranges	$-23 \le h \le 23$, $-20 \le k \le 20$, $-14 \le l \le 14$	
Reflections collected	90146	
Independent reflections	7684 [R_{int} = 0.1017]	
Completeness to θ_{max} (%)	0.991	
Max. and min. transmission	0.33377 and 1.00000	
Refinement method	Full-matrix least-squares on F^2	
Data / restraints / parameters	7684 / 14 / 526	
Goodness-of-fit on F^2	1.113	
Final R indices [$I > 2\sigma(I)$]	$R1$ = 0.0612, $wR2$ = 0.1076	
R indices (all data)	$R1$ = 0.0666, $wR2$ = 0.1097	
Largest diff. peak and hole (e Å^{-3})	0.530 and -0.368	
Absolute structure parameter	.218(6)	

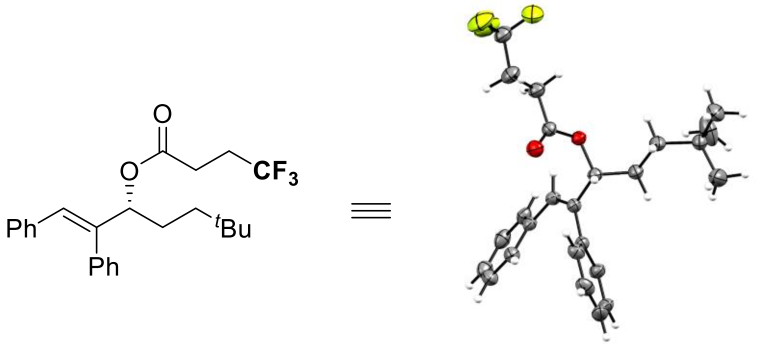

85

single crystal of **86**
CCDC 2094207

Table S10. Crystal data and structure refinement for **85**.

Identification code	**85**
Empirical formula	C25 H29 F3 O2
Formula weight	418.48
Temperature	193(2) K
Wavelength	1.3405 Å
Crystal system	Monoclinic
Space group	$P2_1$

Unit cell dimensions	a = 8.9187(5) Å	α= 90 °.
	b = 9.0784(5) Å	β= 102.370(6) °.
	c = 14.8553(9) Å	γ = 90 °.
Volume	1174.87(12) $Å^3$	
Z	2	
Density (calculated)	1.183 Mg/m^3	
Absorption coefficient	0.474 mm^{-1}	
F(000)	444	
Crystal size	0.2 x 0.1 x 0.1 mm^3	
Theta range for data collection	2.647 to 61.542 °.	
Index ranges	-11<=h<=11, -11<=k<=11, -19<=l<=19	
Reflections collected	33525	
Independent reflections	5373 [R(int) = 0.0385]	
Completeness to theta = 53.543 °	100.0 %	
Absorption correction	Semi-empirical from equivalents	
Max. and min. transmission	1.00000 and 0.74669	
Refinement method	Full-matrix least-squares on F^2	
Data / restraints / parameters	5373 / 61 / 275	
Goodness-of-fit on F^2	1.124	
Final R indices [I>2sigma(I)]	R1 = 0.0551, wR2 = 0.1623	
R indices (all data)	R1 = 0.0663, wR2 = 0.1736	
Absolute structure parameter	0.20(7)	
Extinction coefficient	0.010(3)	
Largest diff. peak and hole	0.431 and -0.205 e.$Å^{-3}$	

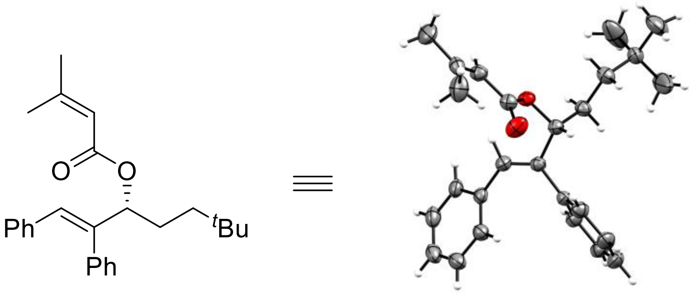

89

single crystal of **90**
CCDC 2094210

Table S11. Crystal data and structure refinement for **89**.

Identification code	**89**
Empirical formula	$C_{26}H_{32}O_2$
Formula weight	376.51
Temperature (K)	293(2)
Wavelength (Å)	1.3405

Crystal system	orthorhombic
Space group	$P2_12_12_1$
Unit cell dimensions (Å, °)	a = 9.0491(2) α = 90
	b = 9.2231(2) β = 90
	c = 27.9292(5) γ = 90
Volume (Å)	2330.99(8)
Z	4
Calculated density (g cm^{-3})	1.073
Absorption coefficient (mm^{-1})	0.324
F_{000}	816
Crystal size (mm^3)	× ×
θ range for data collection (°)	2.751 to 60.532
Miller index ranges	$-11 \leq h \leq 11$, $-11 \leq k \leq 11$, $-35 \leq l \leq 36$
Reflections collected	65508
Independent reflections	5317 [R_{int} = 0.0377]
Completeness to θ_{max} (%)	0.996
Max. and min. transmission	0.79654 and 1.00000
Refinement method	Full-matrix least-squares on F^2
Data / restraints / parameters	5317 / 0 / 260
Goodness-of-fit on F^2	1.085
Final R indices [$I > 2\sigma(I)$]	$R1$ = 0.0481, $wR2$ = 0.1460
R indices (all data)	$R1$ = 0.0573, $wR2$ = 0.1552
Extinction coefficient	0.0026(8)
Largest diff. peak and hole (e Å^{-3})	0.274 and -0.189
Absolute structure parameter	.2(5)

I. NMR spectra

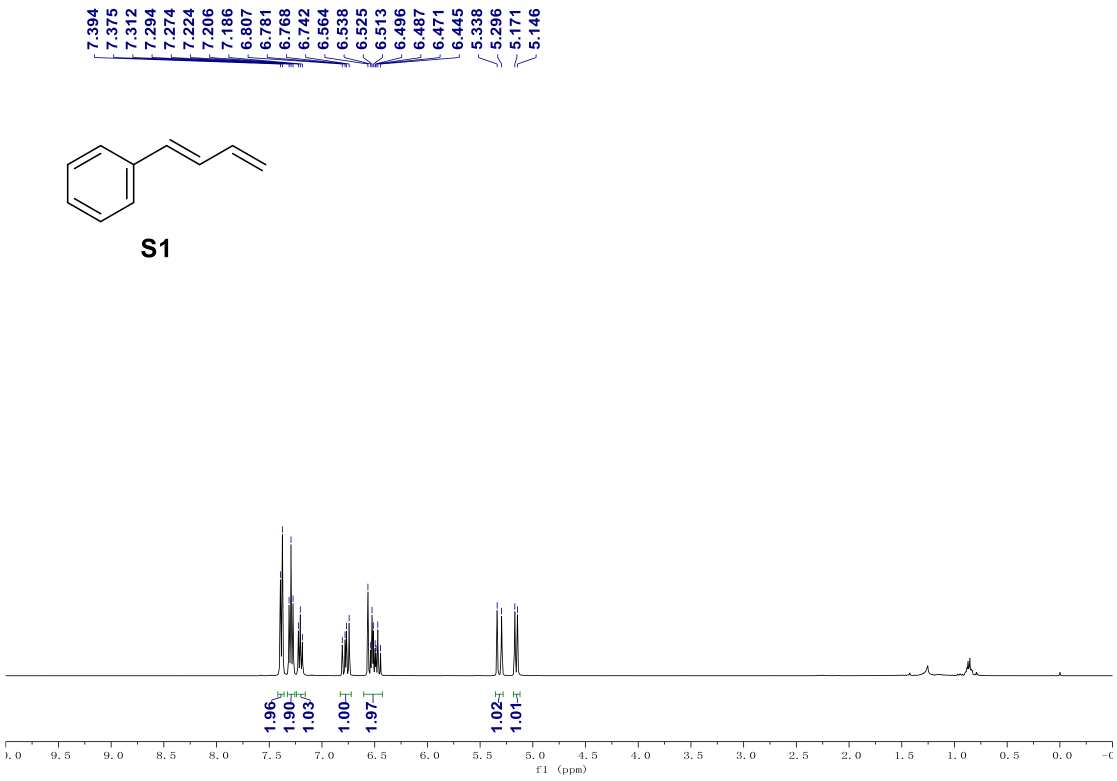

Supplementary Figure 11. ^{1}H NMR spectra of compound **S1**

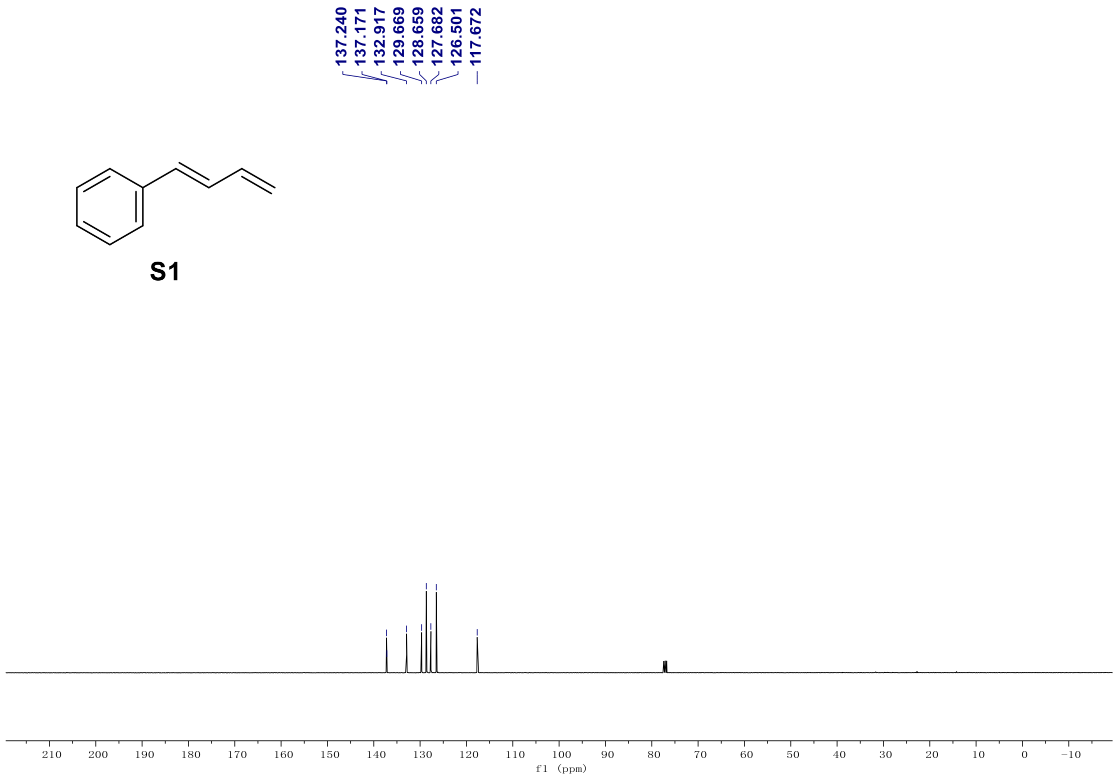

Supplementary Figure 12. ^{13}C NMR spectra of compound **S1**

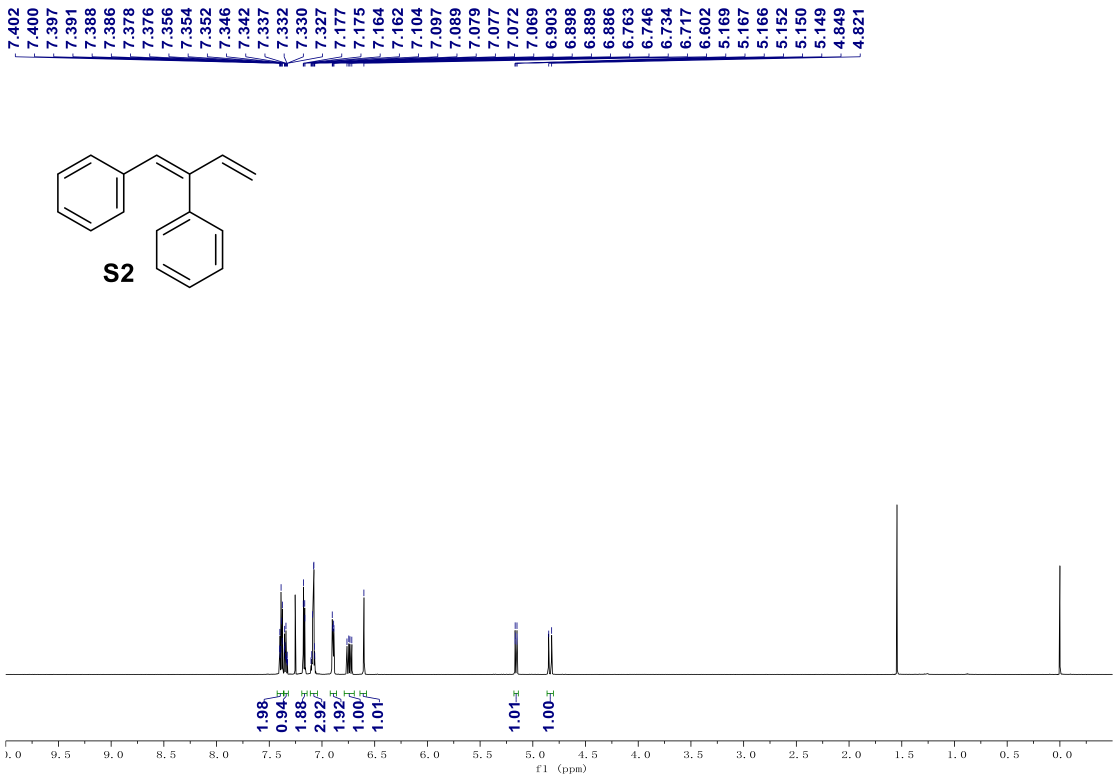

Supplementary Figure 13. ^{1}H NMR spectra of compound **S2**

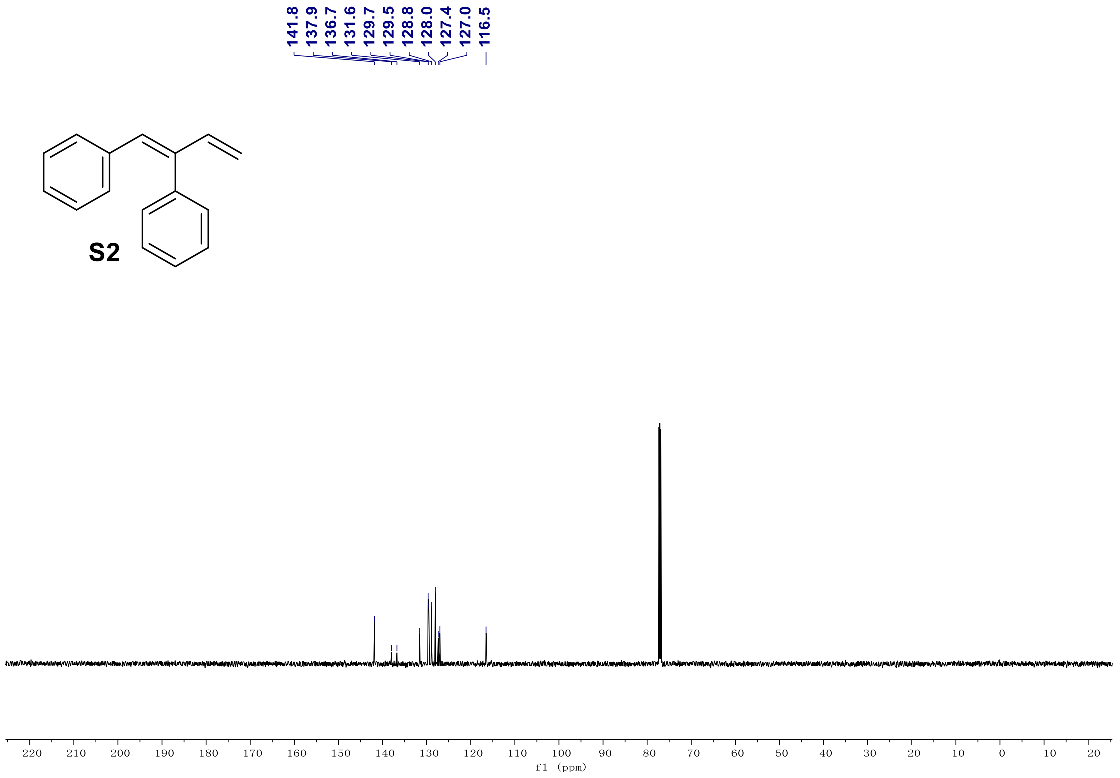

Supplementary Figure 14. ^{13}C NMR spectra of compound **S2**

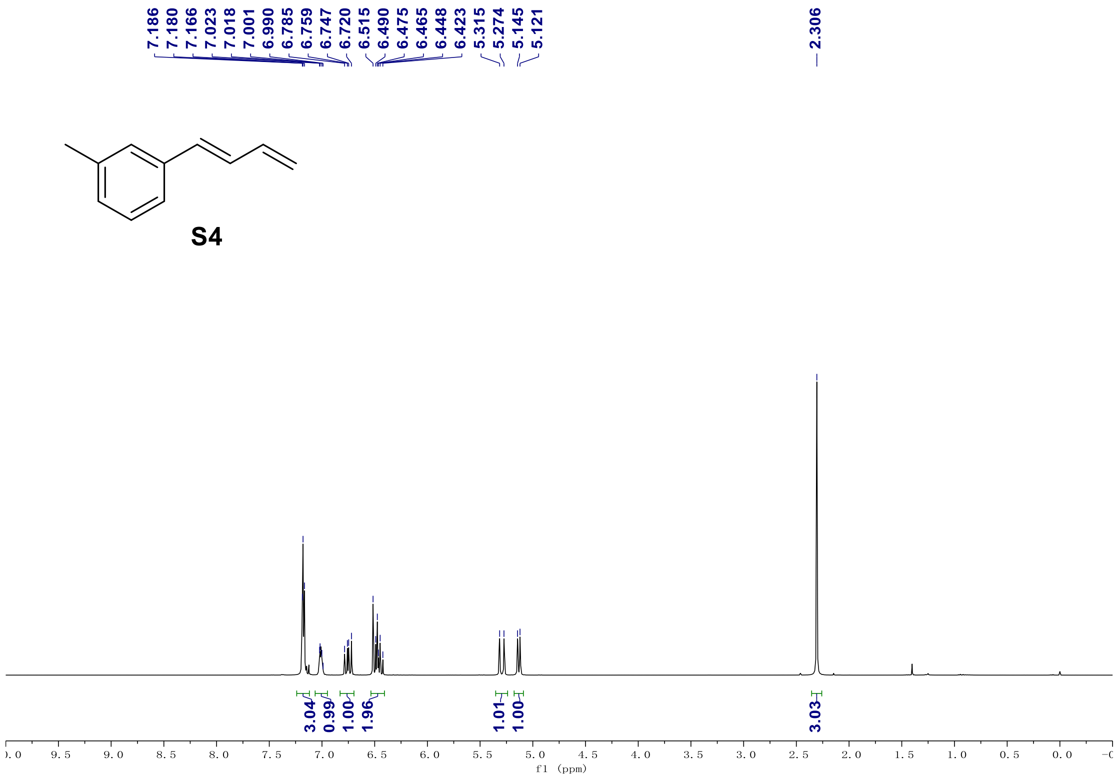

Supplementary Figure 15. ^{1}H NMR spectra of compound **S4**

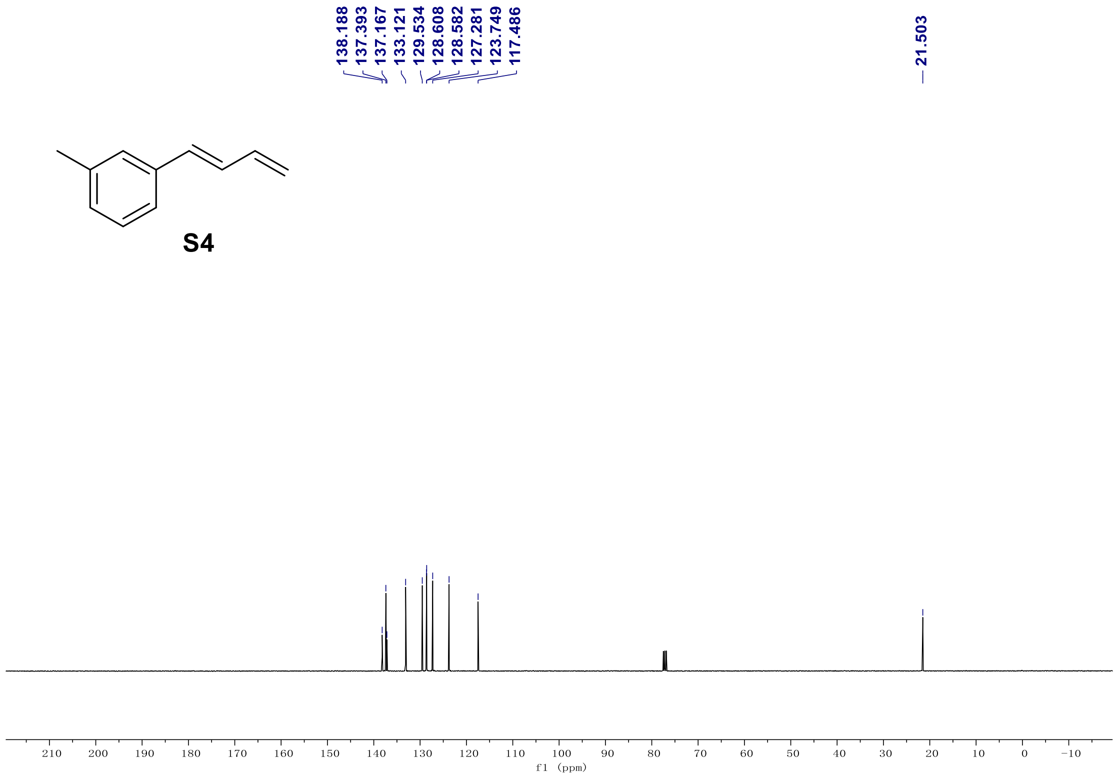

Supplementary Figure 16. ^{13}C NMR spectra of compound **S4**

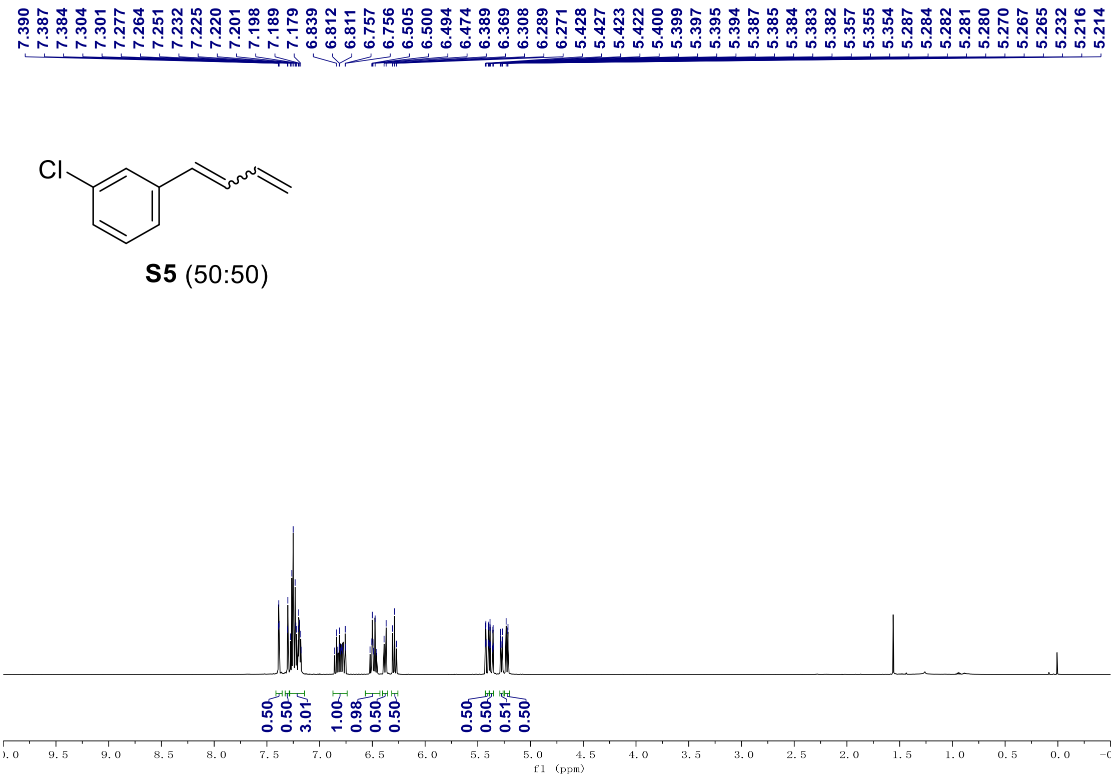

Supplementary Figure 17. ^{1}H NMR spectra of compound **S5**

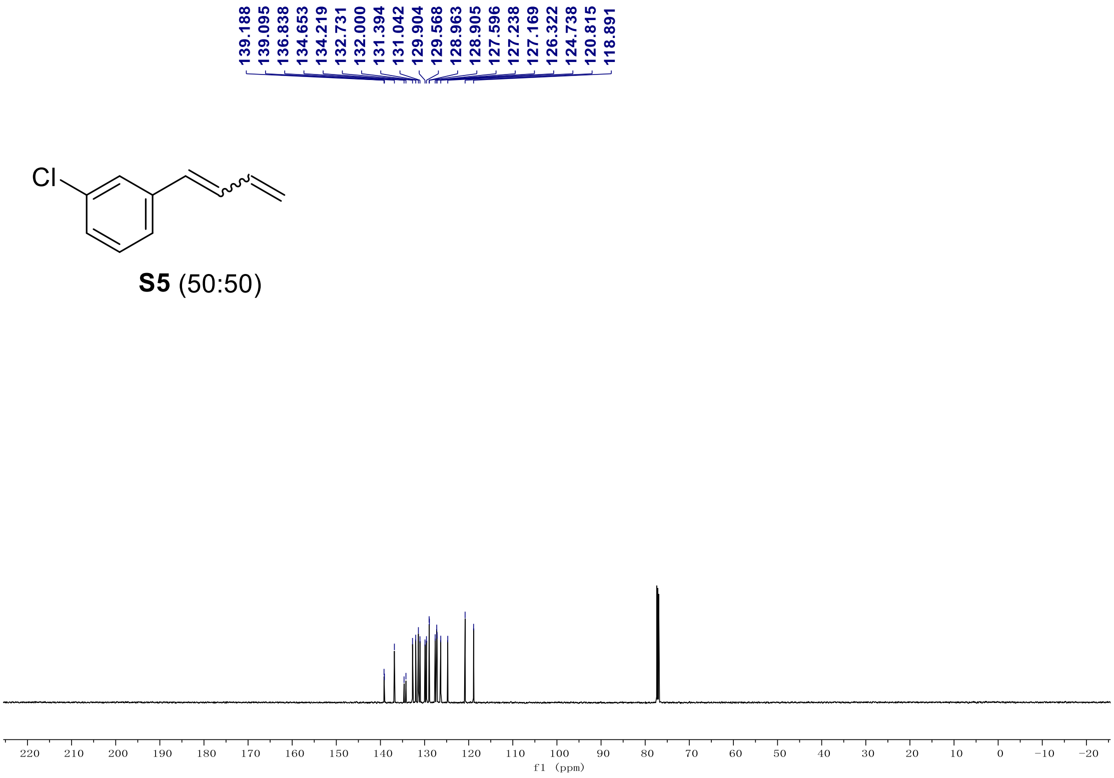

Supplementary Figure 18. ^{13}C NMR spectra of compound **S5**

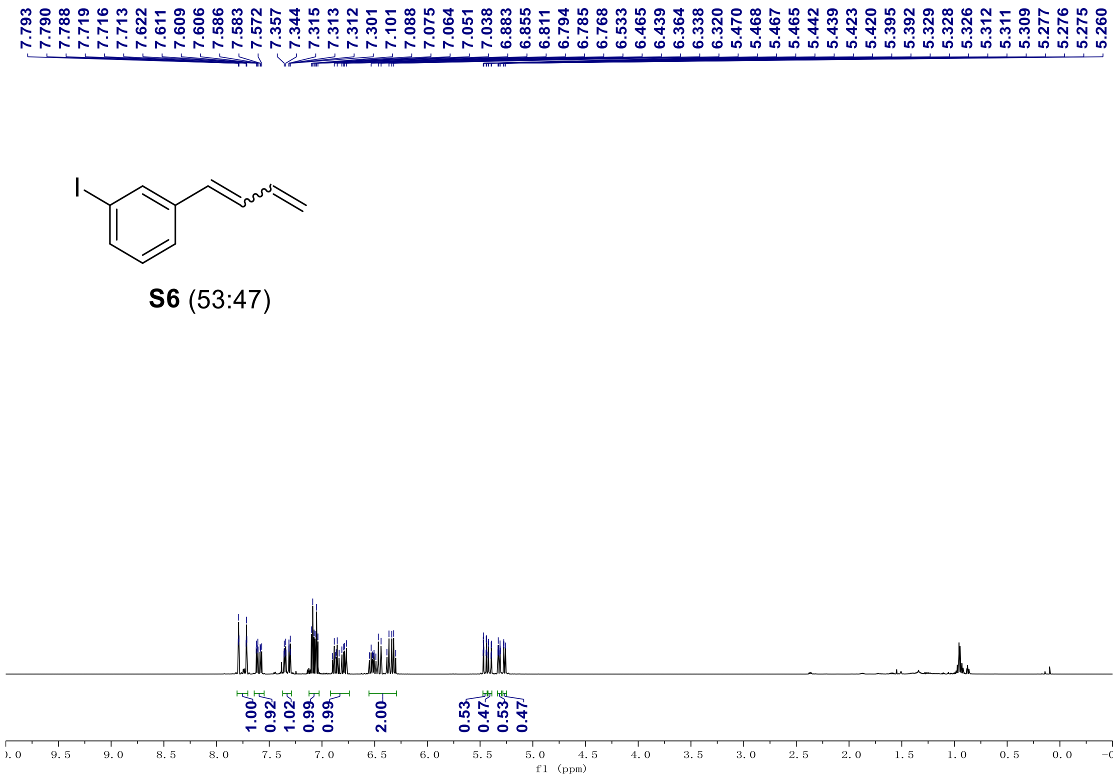

Supplementary Figure 19. ^{1}H NMR spectra of compound **S6**

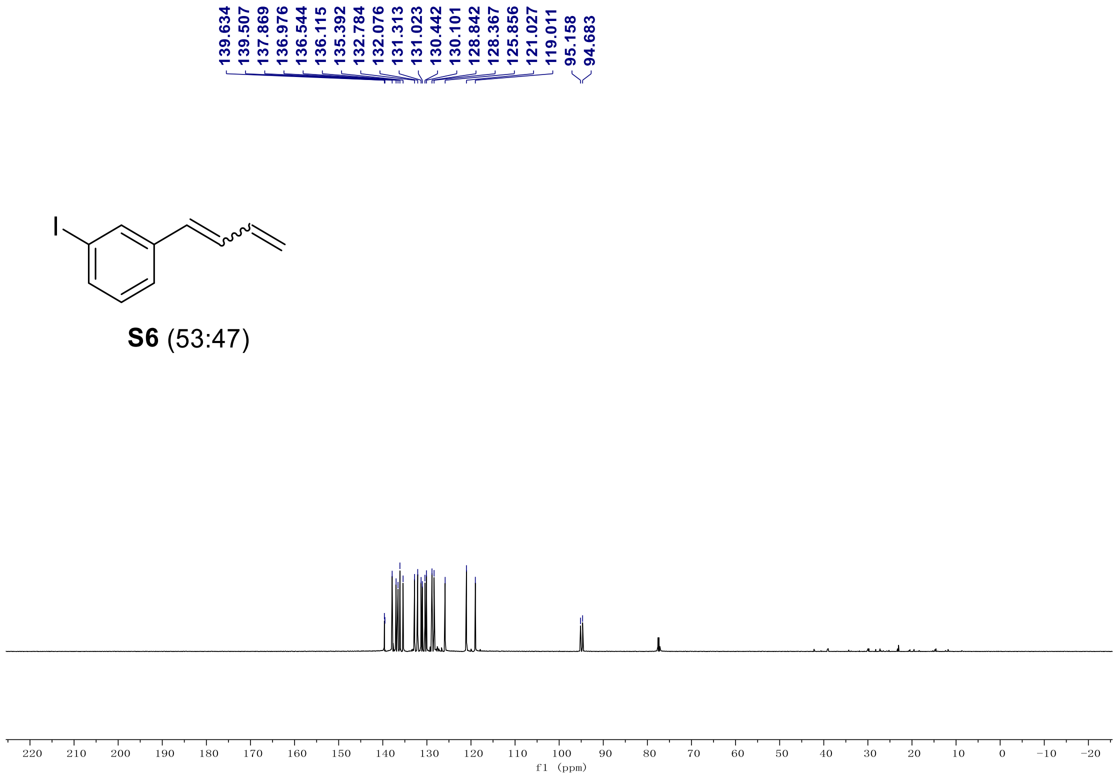

Supplementary Figure 20. ^{13}C NMR spectra of compound **S6**

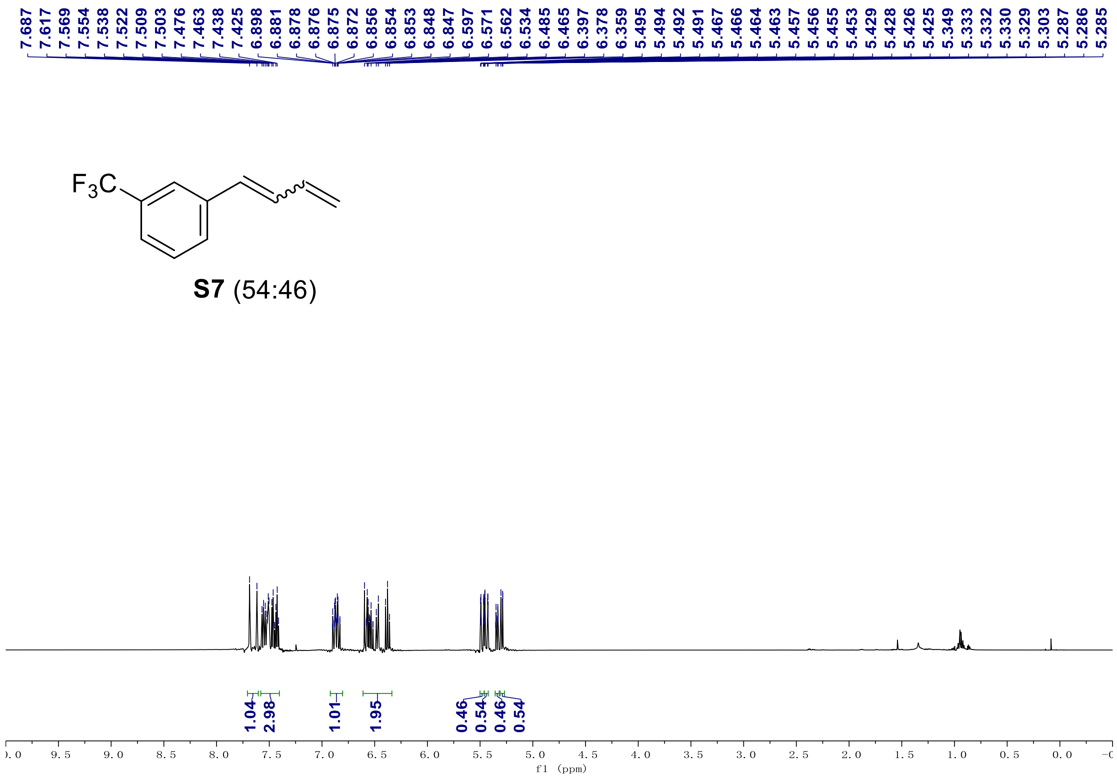

Supplementary Figure 21. ¹H NMR spectra of compound **S7**

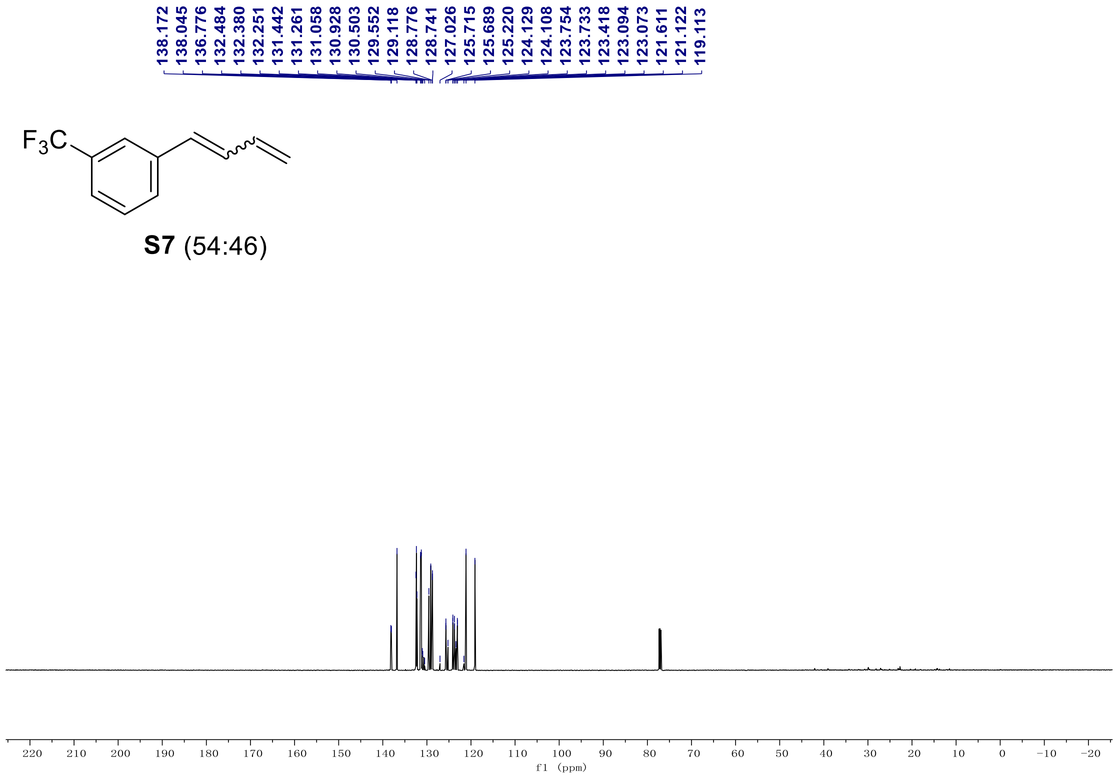

Supplementary Figure 22. ¹³C NMR spectra of compound **S7**

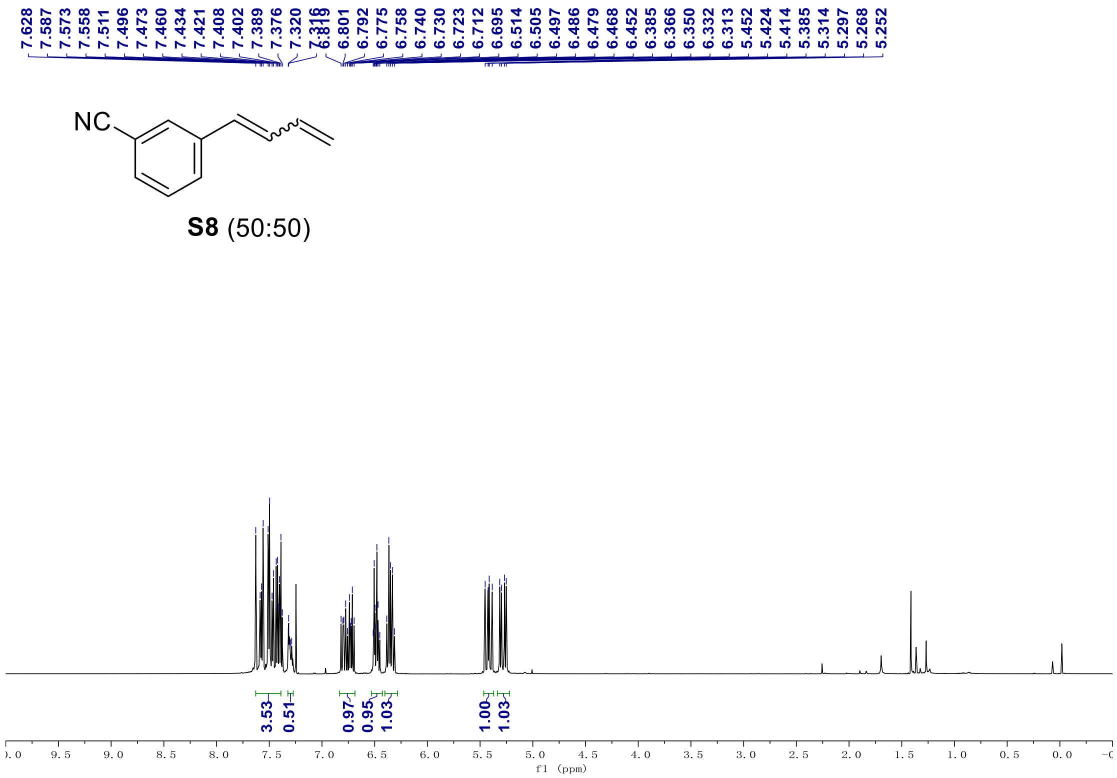

Supplementary Figure 23. ^{1}H NMR spectra of compound **S8**

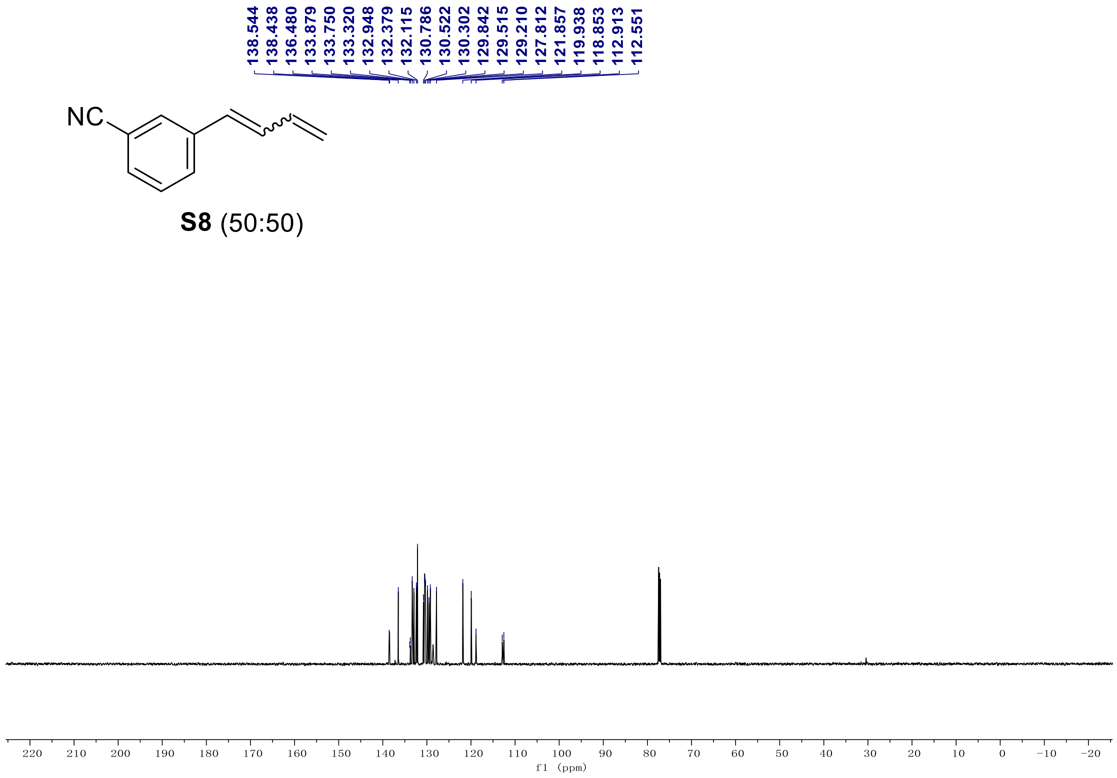

Supplementary Figure 24. ^{13}C NMR spectra of compound **S8**

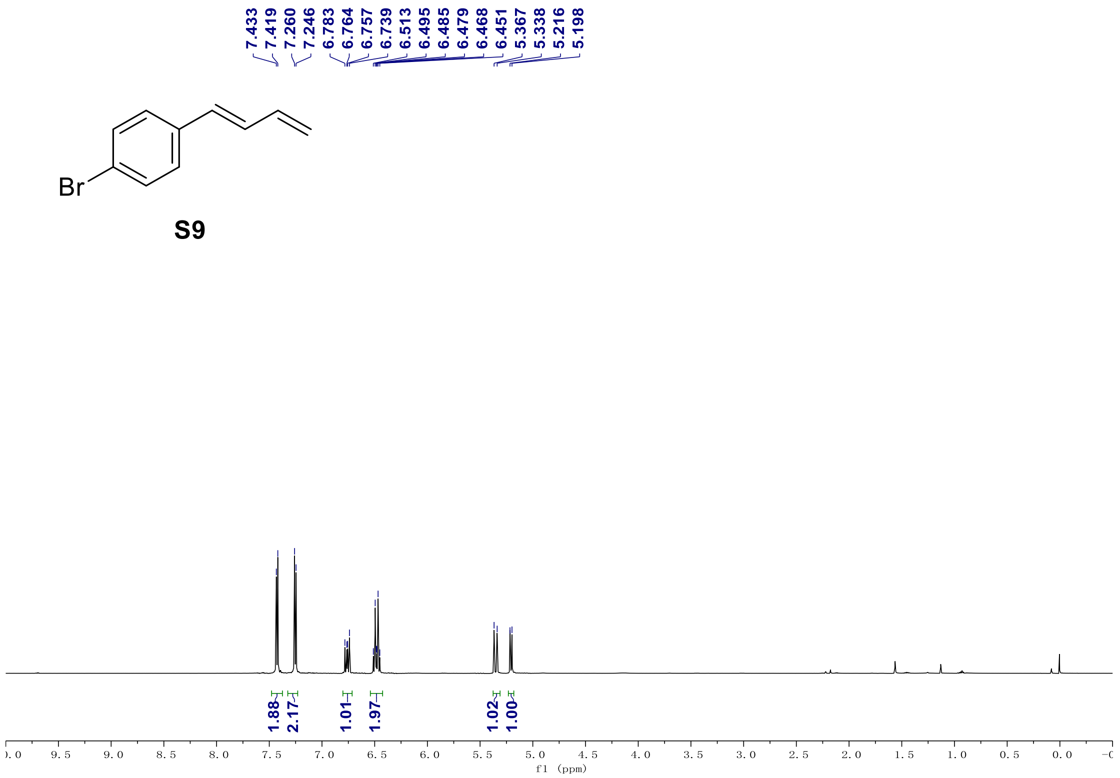

Supplementary Figure 25. ^{1}H NMR spectra of compound **S9**

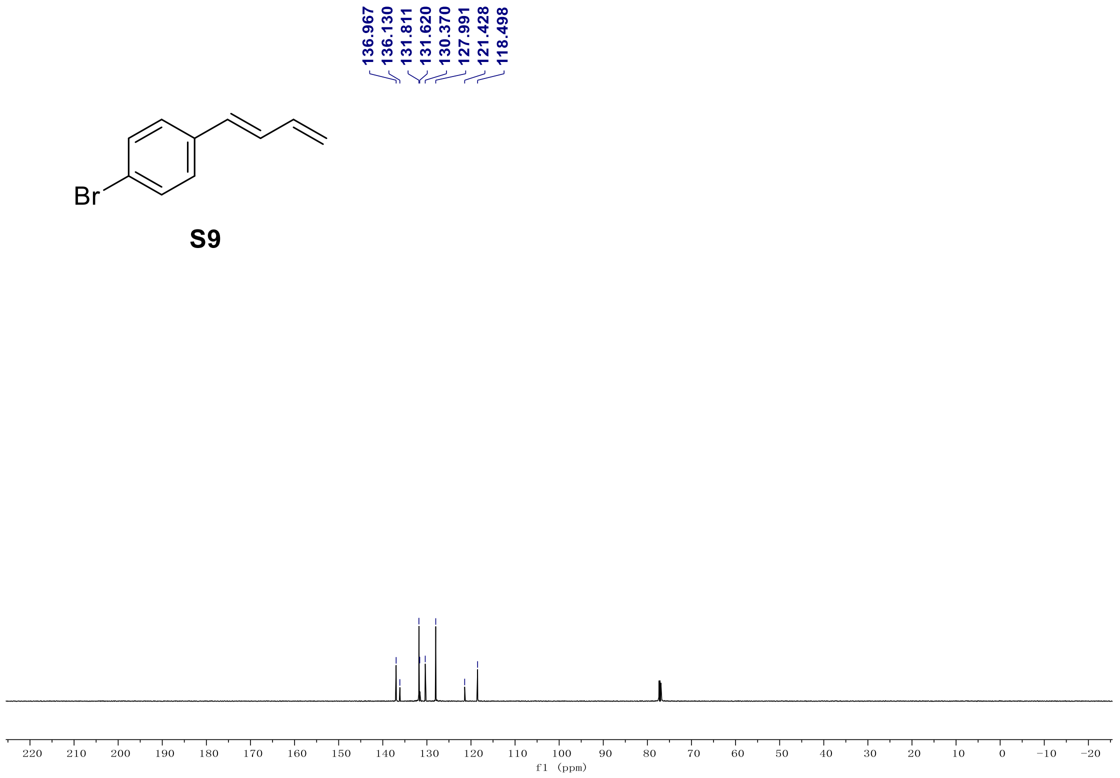

Supplementary Figure 26. ^{13}C NMR spectra of compound **S9**

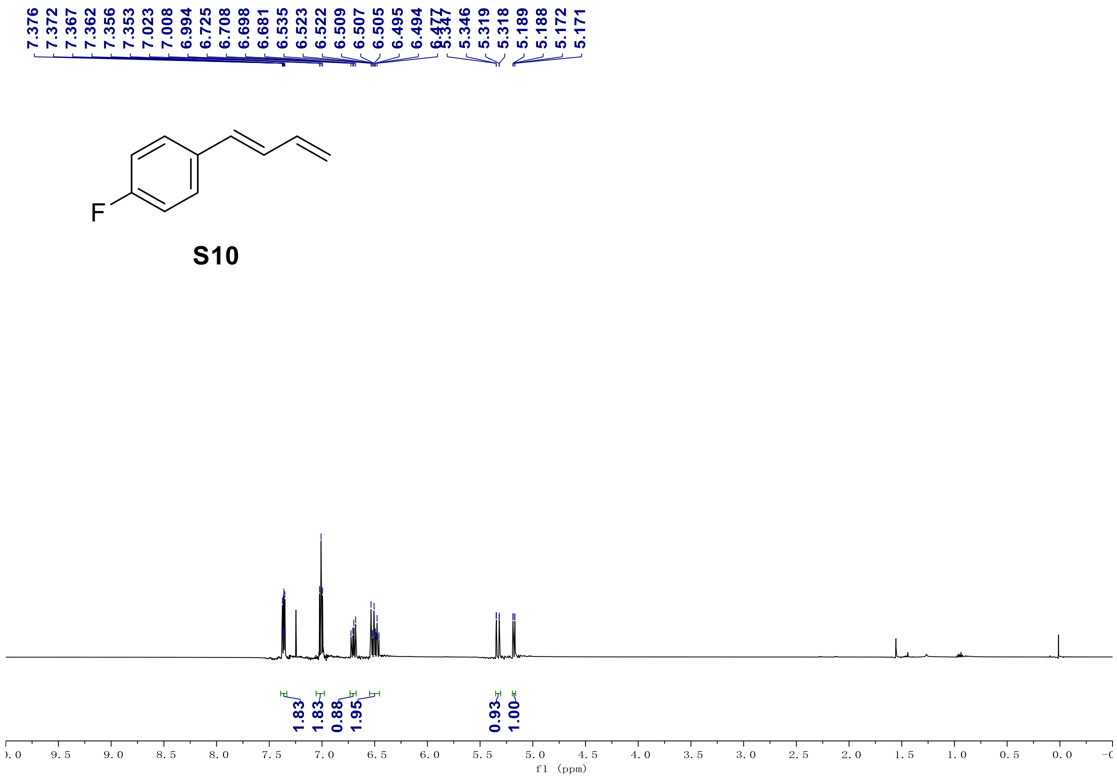

Supplementary Figure 27. ^{1}H NMR spectra of compound **S10**

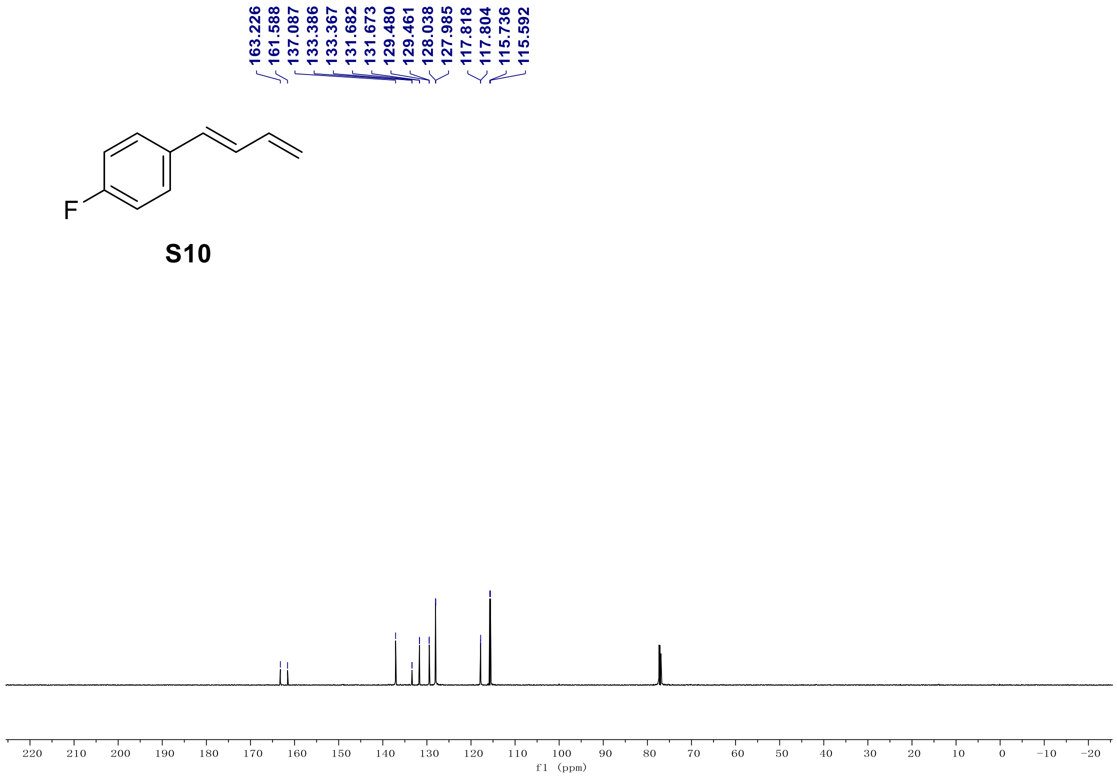

Supplementary Figure 28. ^{13}C NMR spectra of compound **S10**

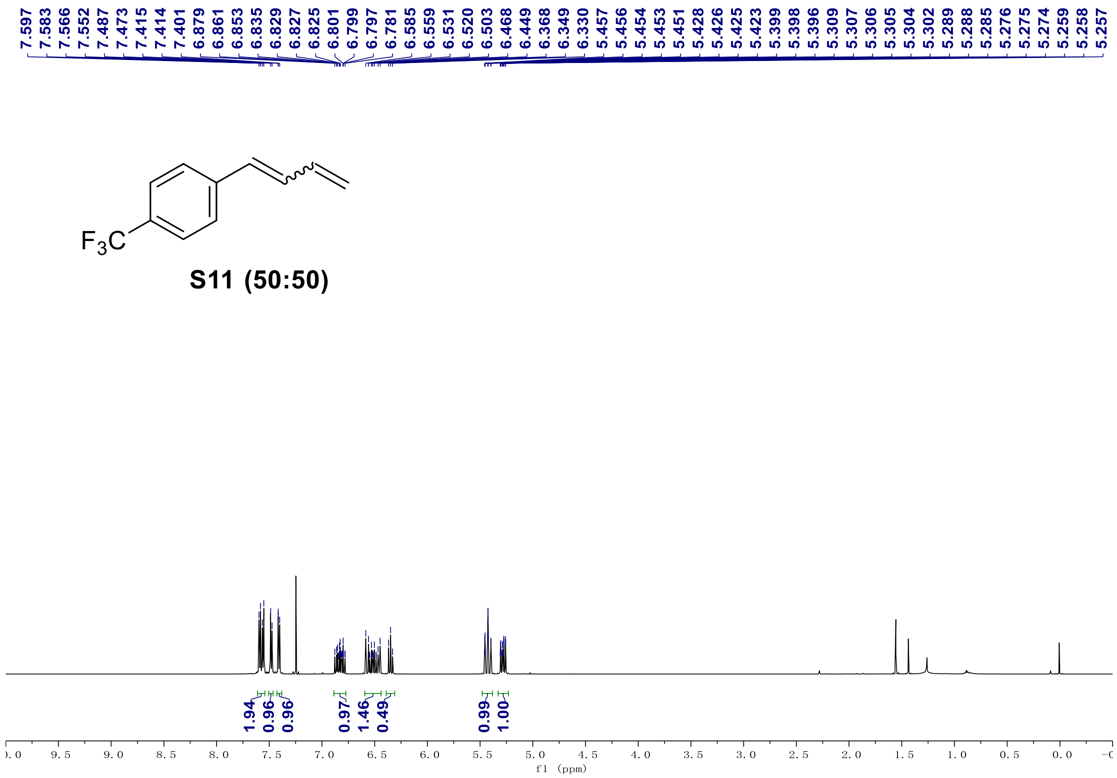

Supplementary Figure 29. ^{1}H NMR spectra of compound **S11**

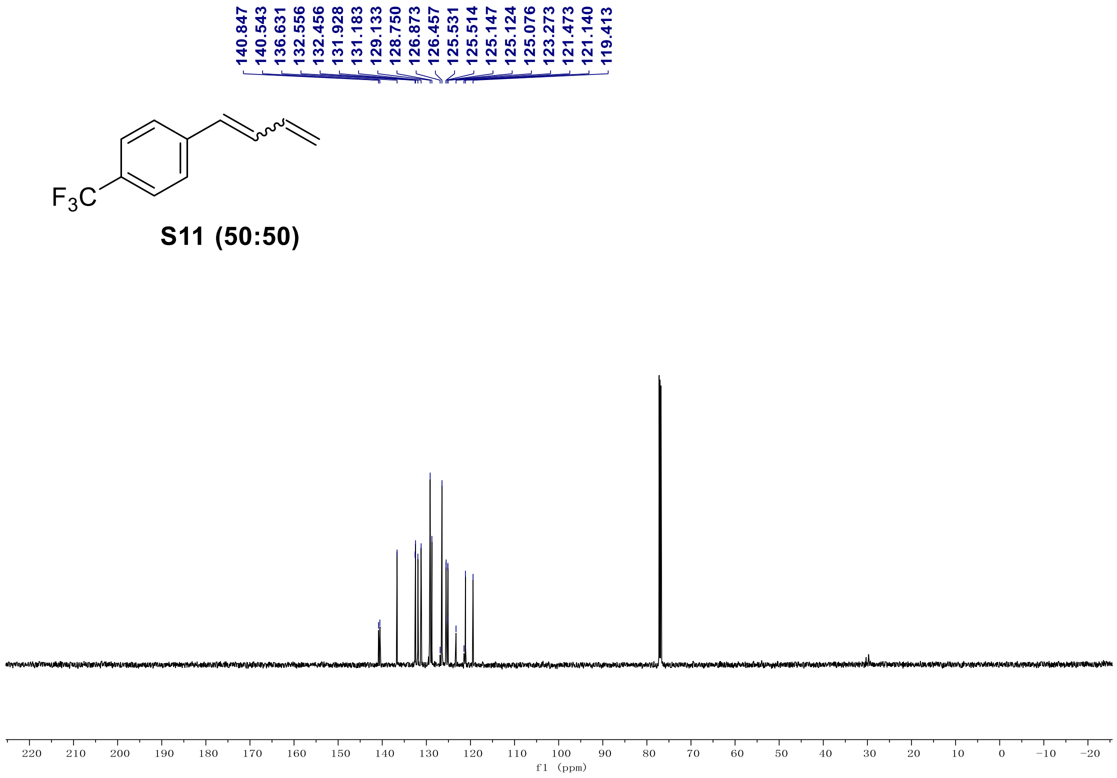

Supplementary Figure 30. ^{13}C NMR spectra of compound **S11**

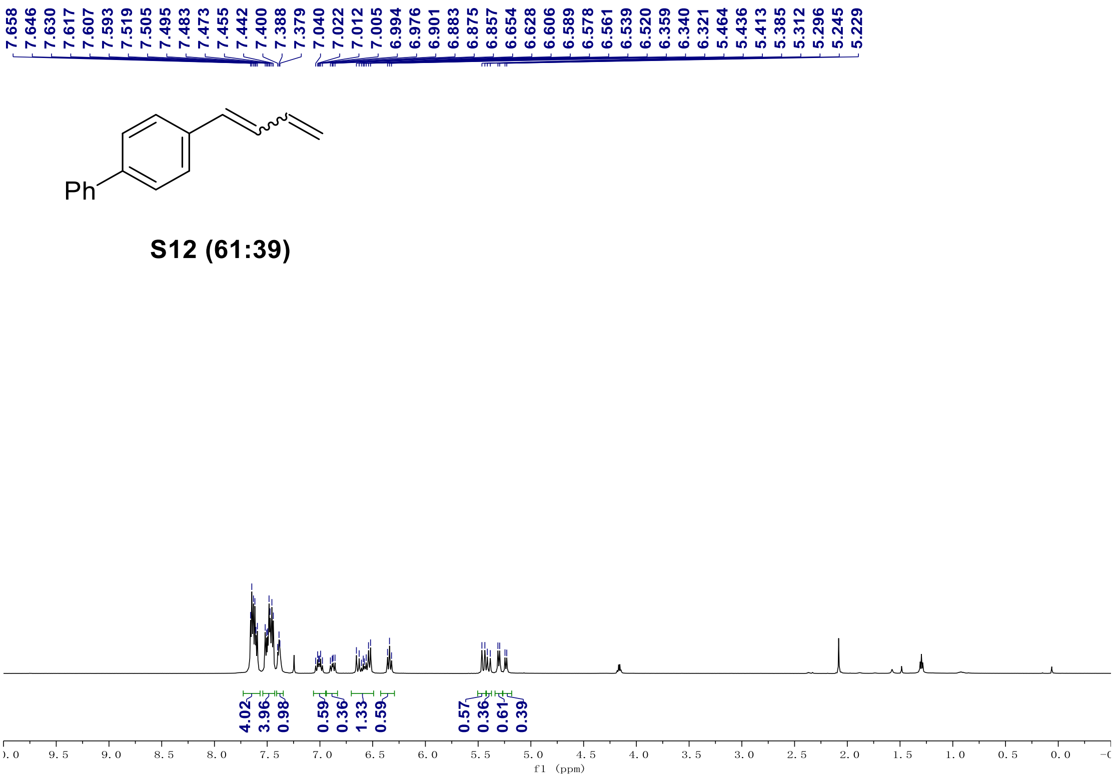

Supplementary Figure 31. ^{1}H NMR spectra of compound **S12**

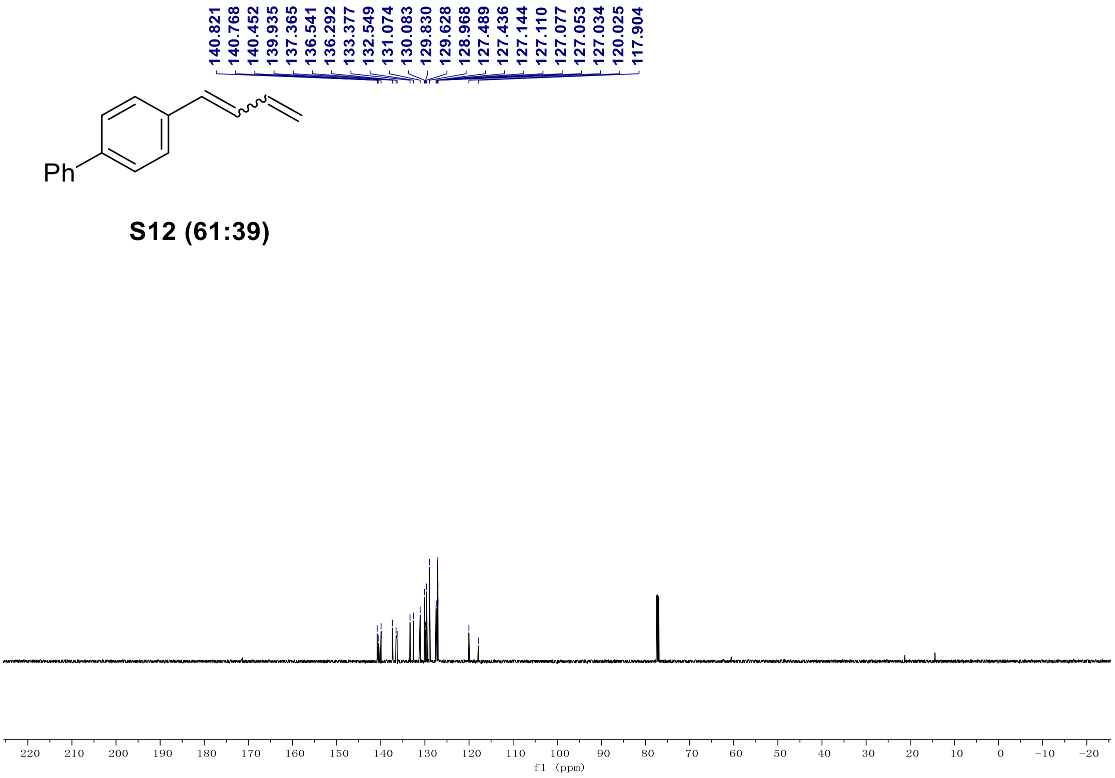

Supplementary Figure 32. ^{13}CNMR spectra of compound **S12**

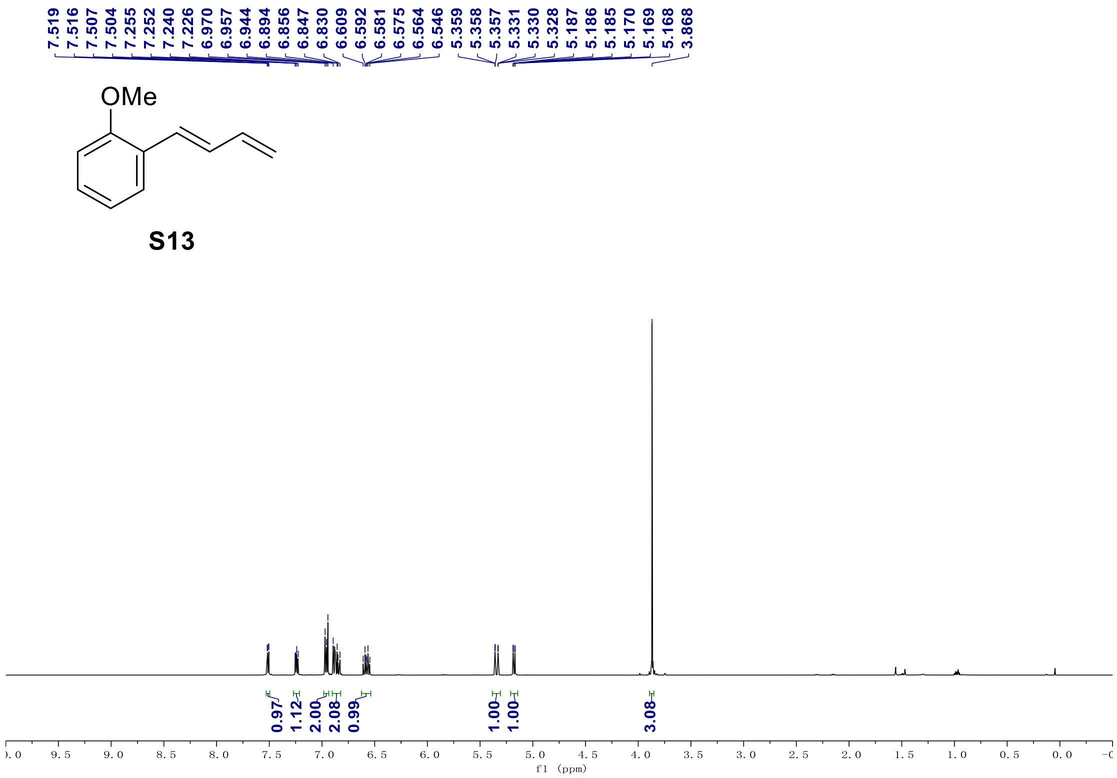

Supplementary Figure 33. ^{1}H NMR spectra of compound **S13**

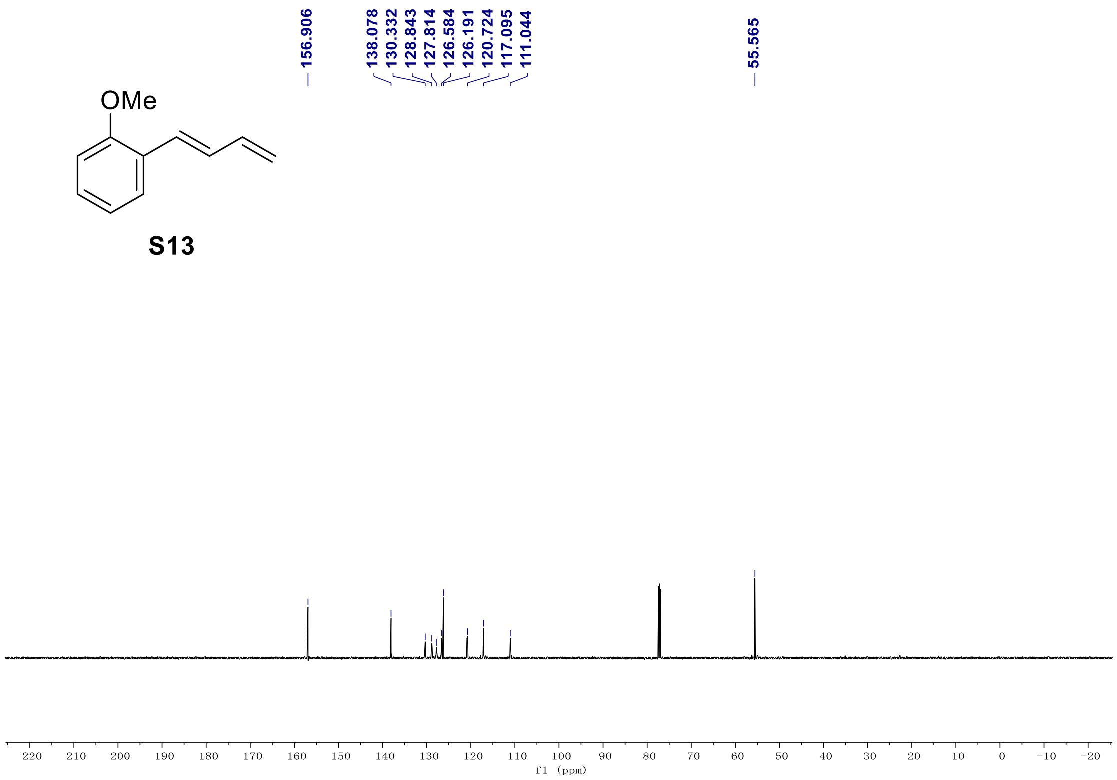

Supplementary Figure 34. ^{13}C NMR spectra of compound **S13**

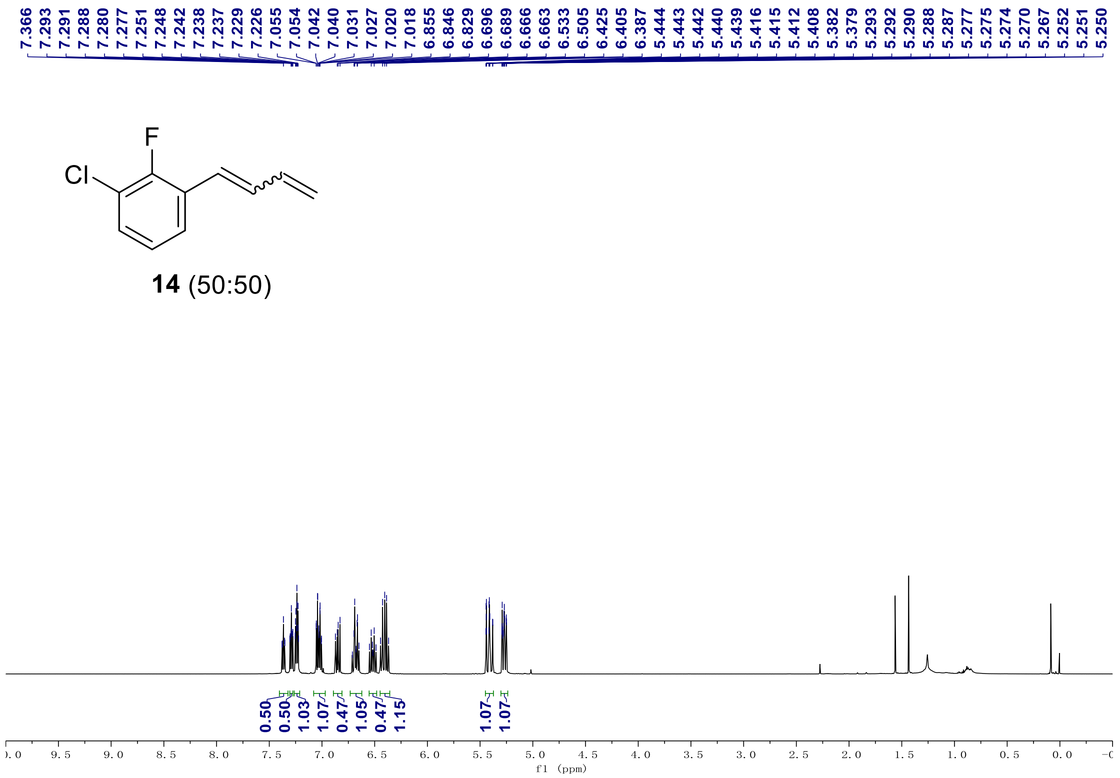

Supplementary Figure 35. ^{1}H NMR spectra of compound **S14**

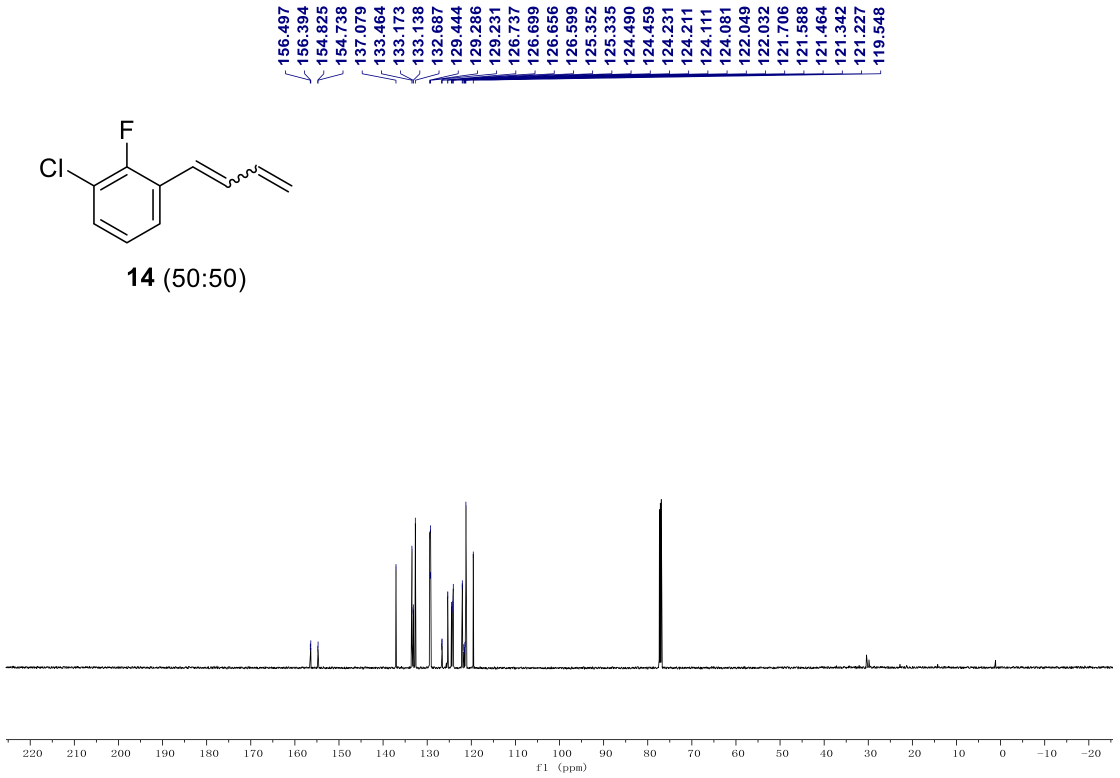

Supplementary Figure 36. ^{13}C NMR spectra of compound **S14**

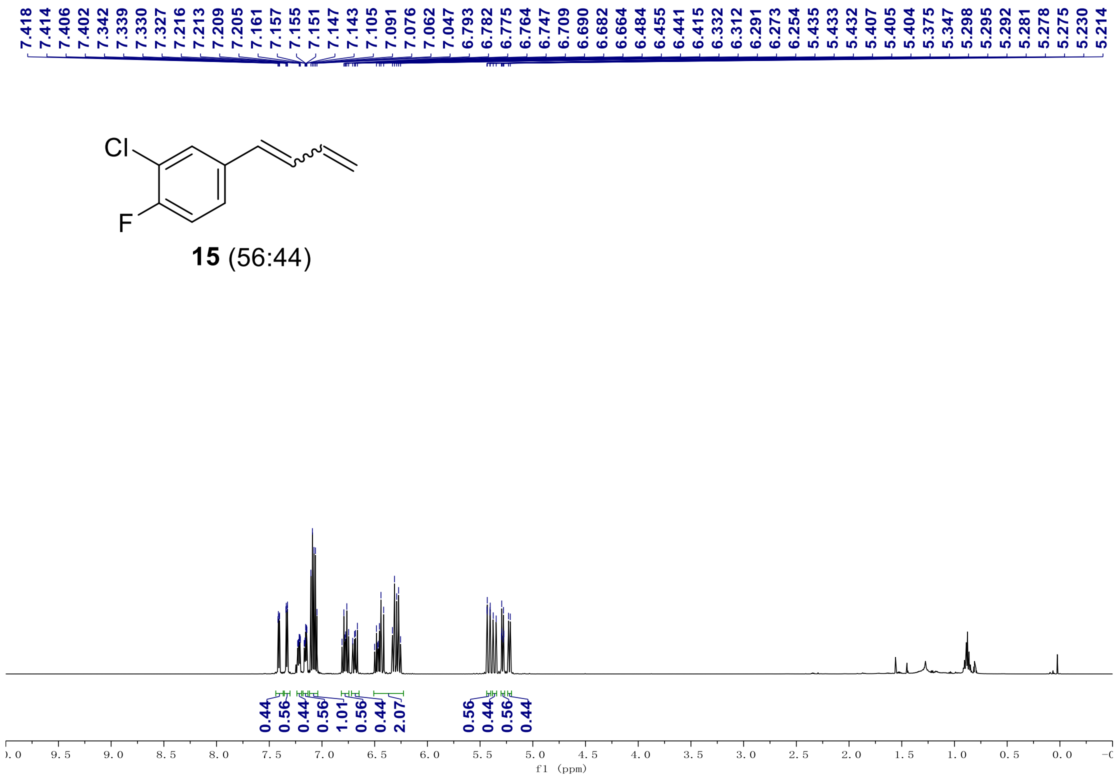

Supplementary Figure 37. ^{1}H NMR spectra of compound **S15**

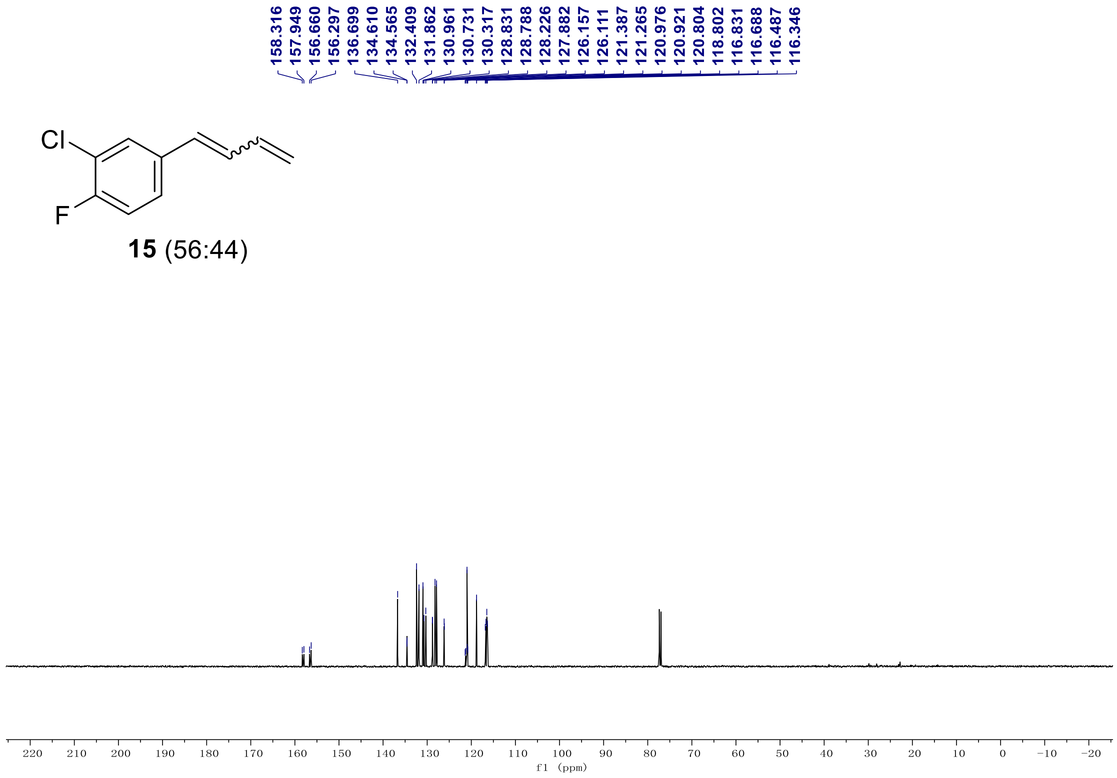

Supplementary Figure 38. ^{13}C NMR spectra of compound **S15**

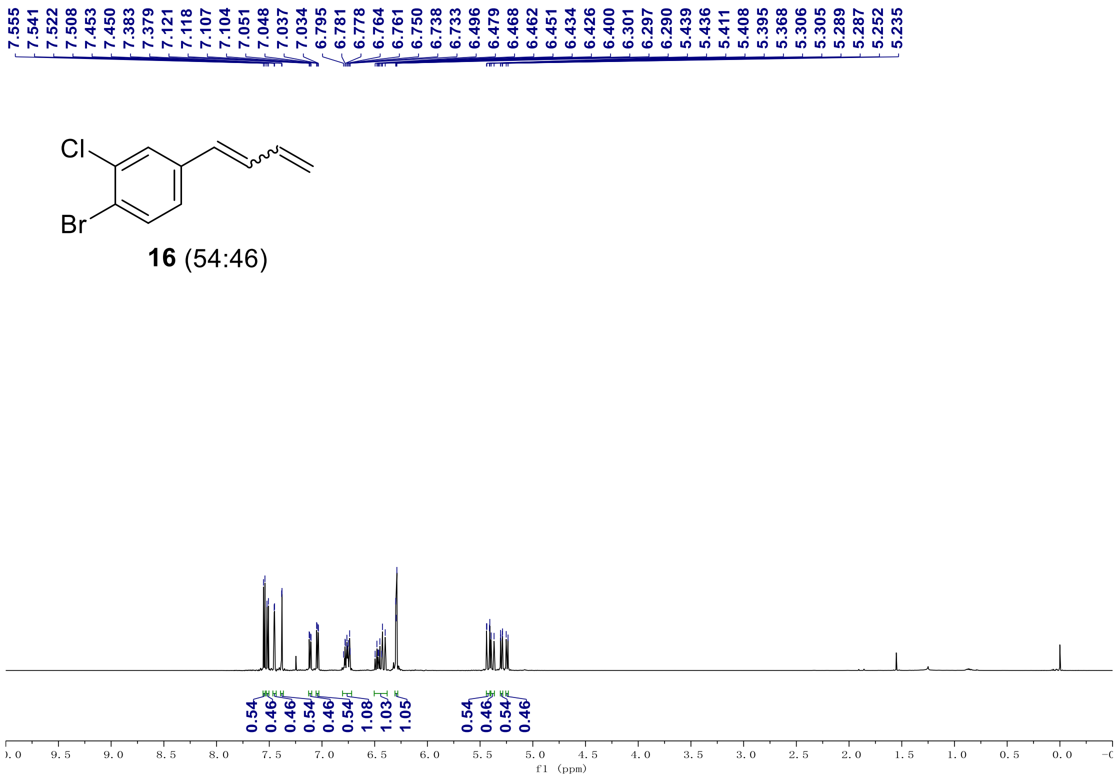

Supplementary Figure 39. ¹H NMR spectra of compound **S15**

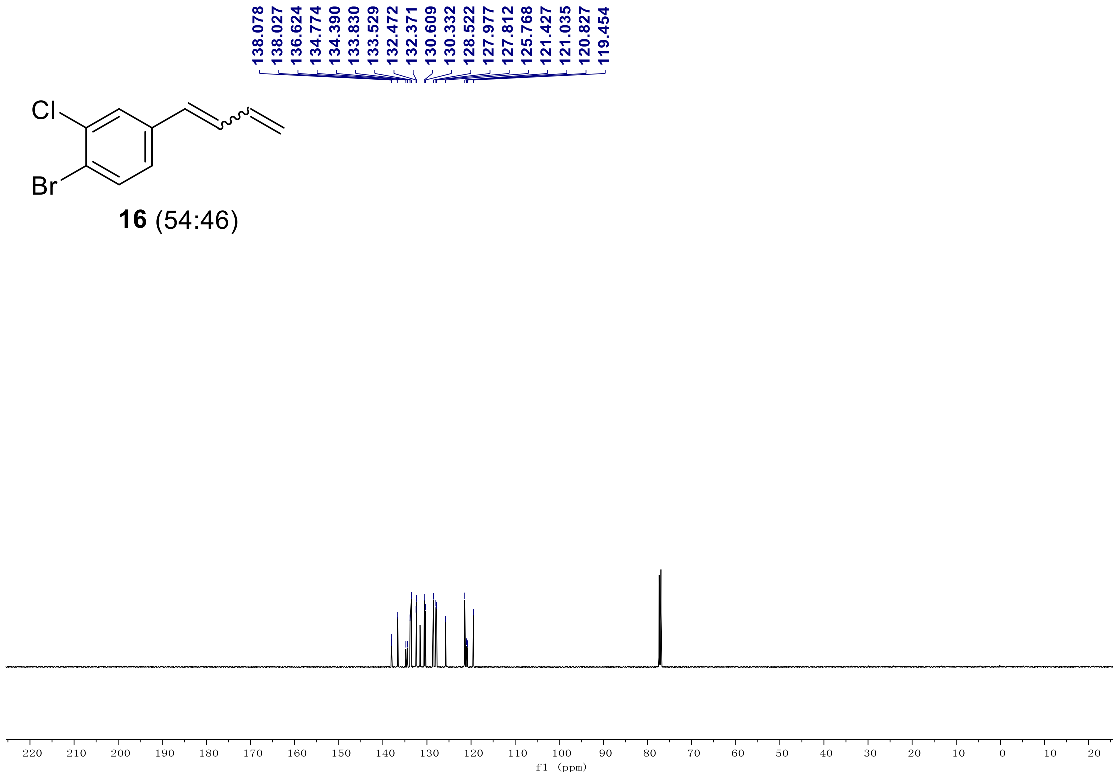

Supplementary Figure 40. ¹³C NMR spectra of compound **S16**

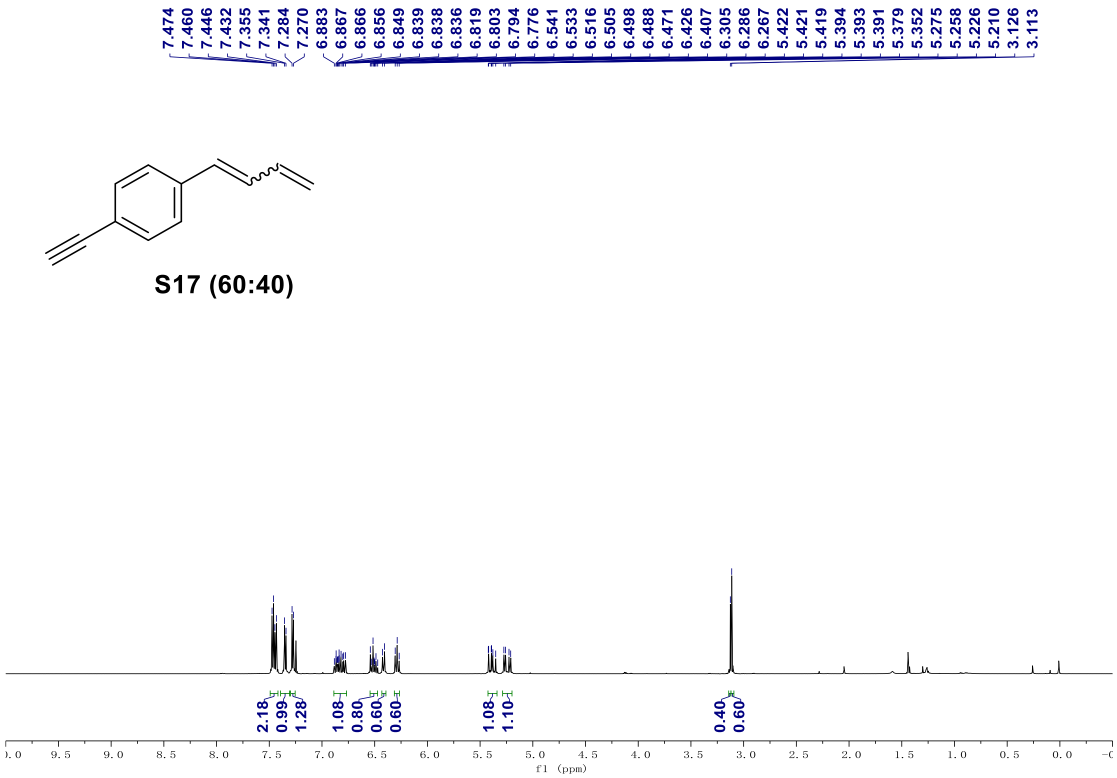

Supplementary Figure 41. ^{1}H NMR spectra of compound **S17**

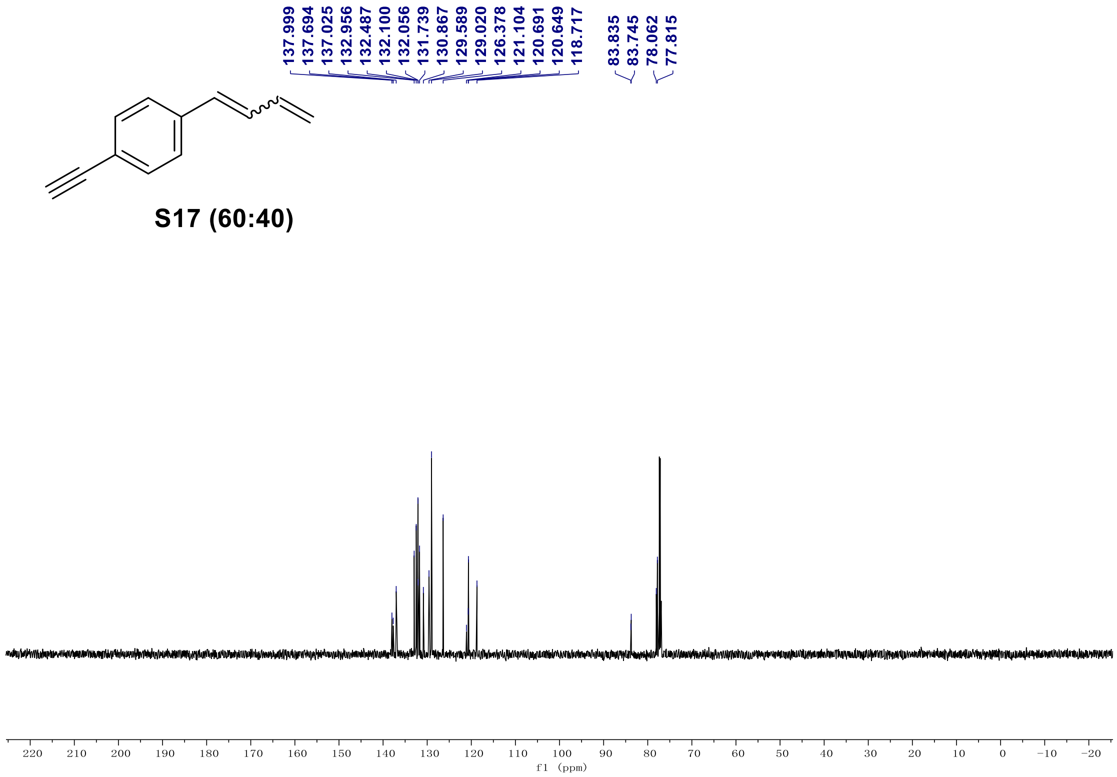

Supplementary Figure 42. ^{13}C NMR spectra of compound **S18**

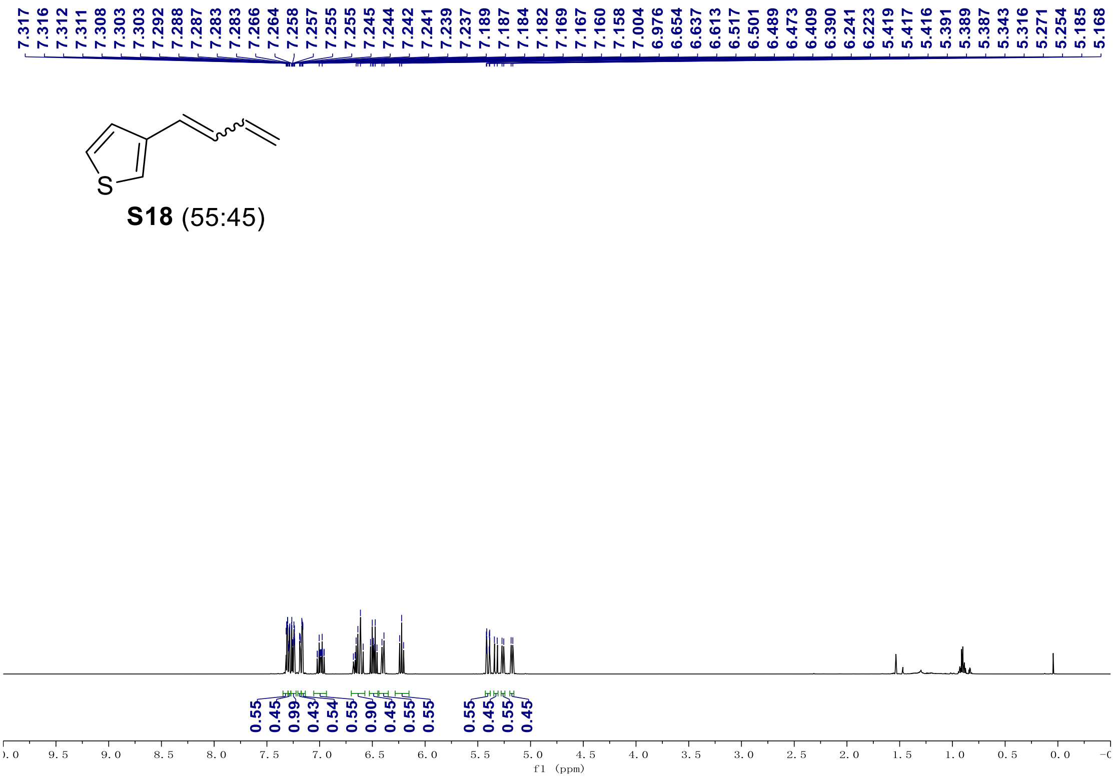

Supplementary Figure 43. ^{1}H NMR spectra of compound **S18**

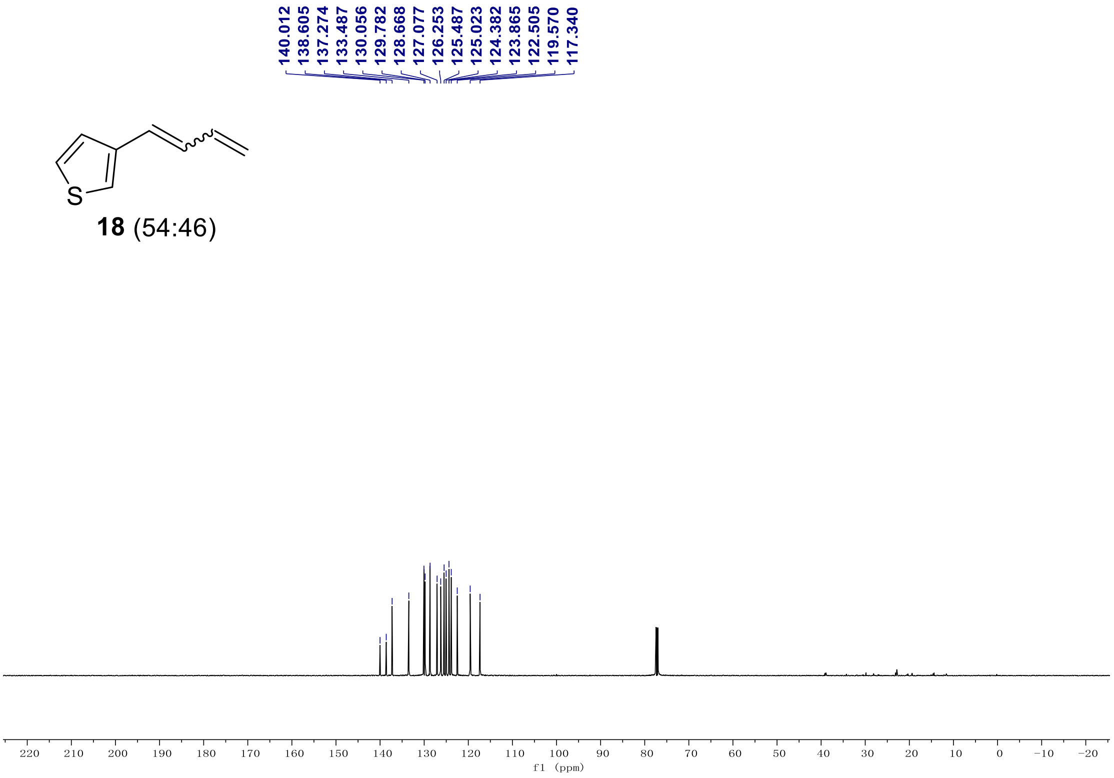

Supplementary Figure 44. ^{13}C NMR spectra of compound **S18**

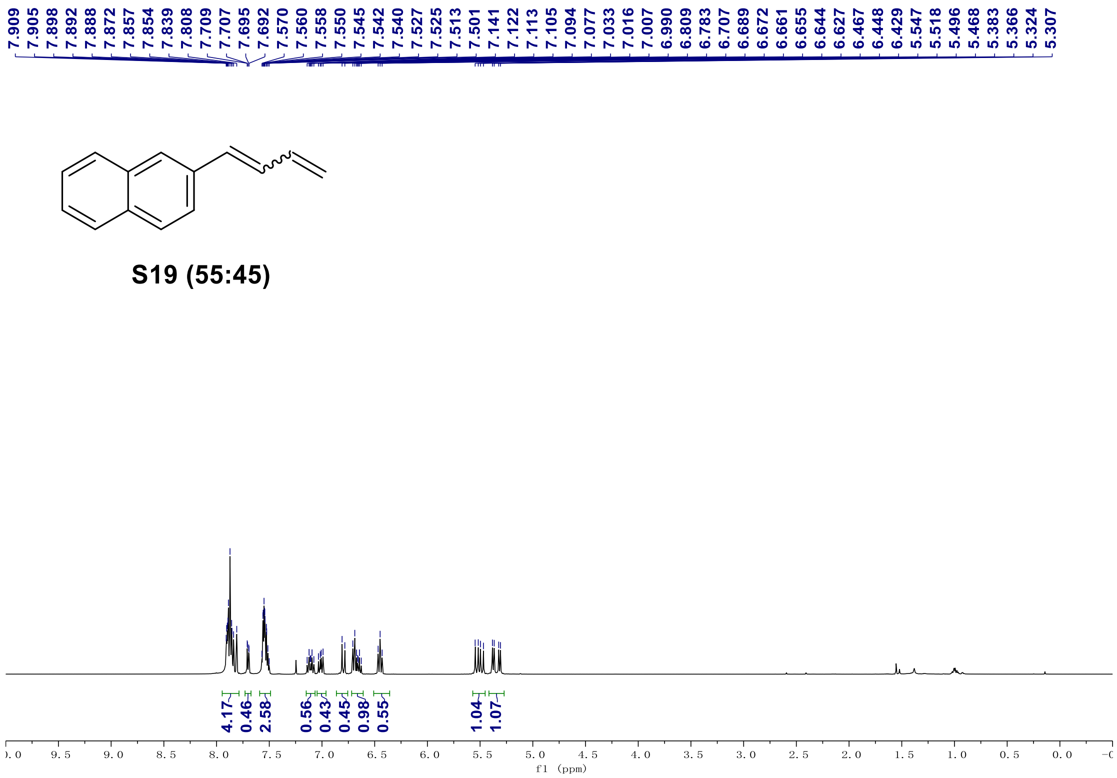

Supplementary Figure 45. ^{1}H NMR spectra of compound **S19**

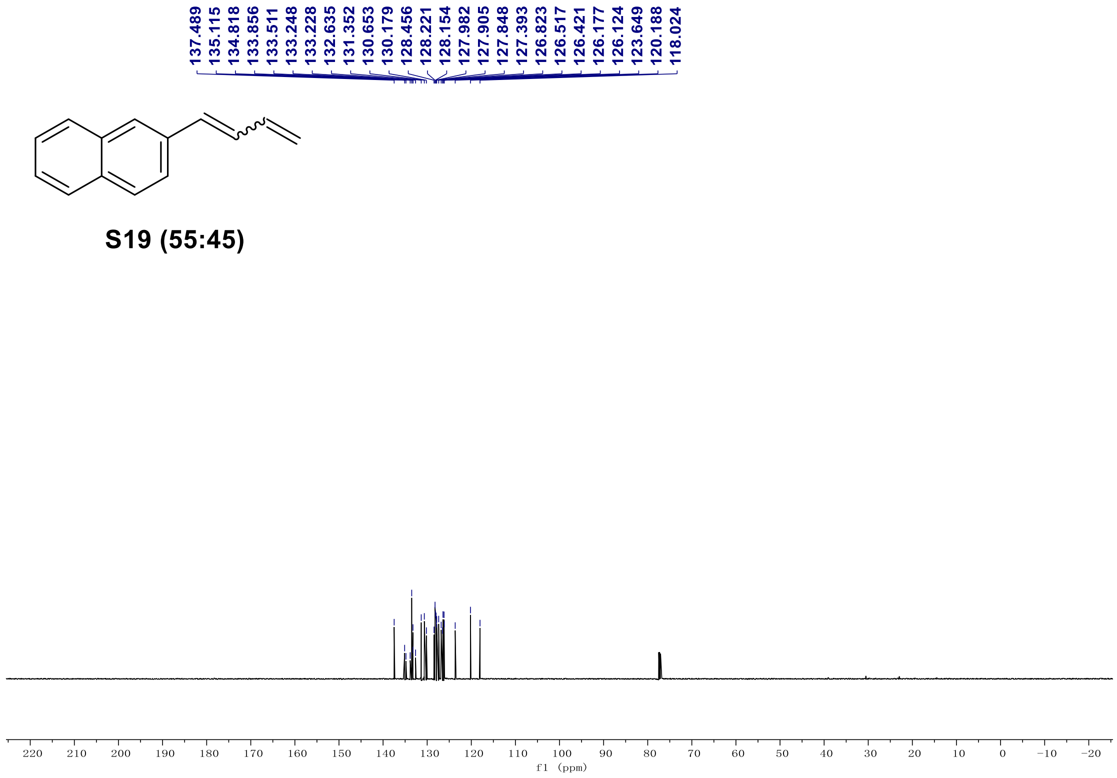

Supplementary Figure 46. ^{13}C NMR spectra of compound **S19**

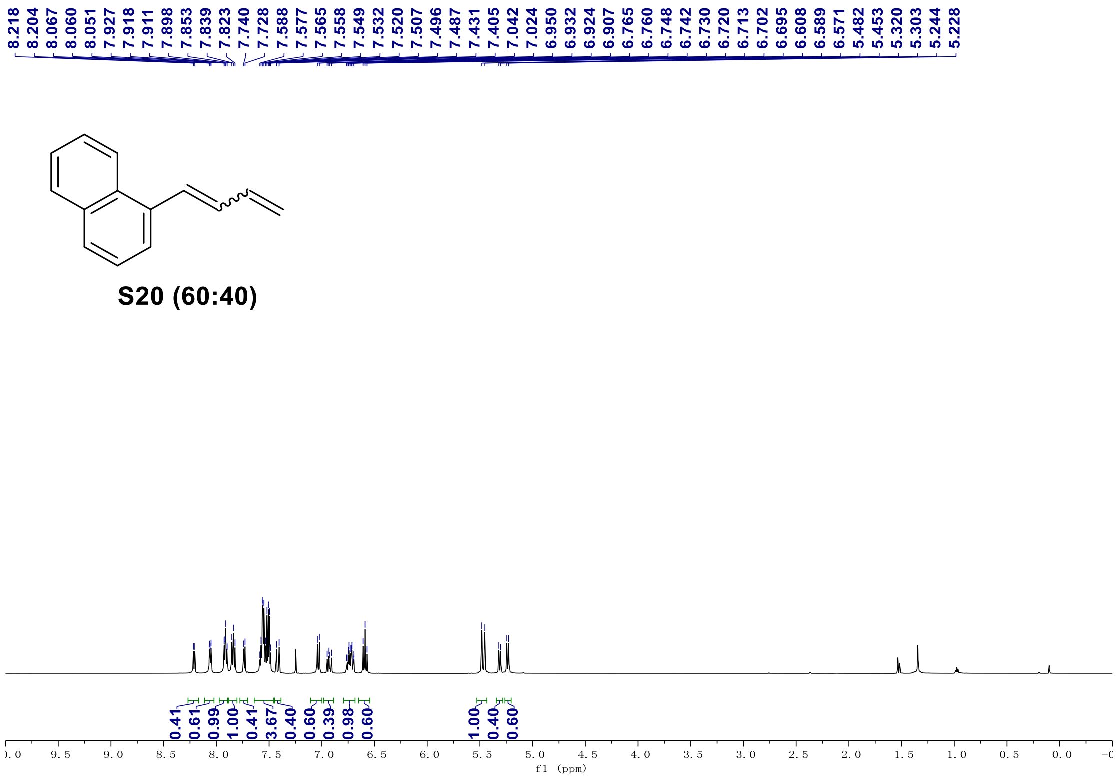

Supplementary Figure 47. ^{1}H NMR spectra of compound **S20**

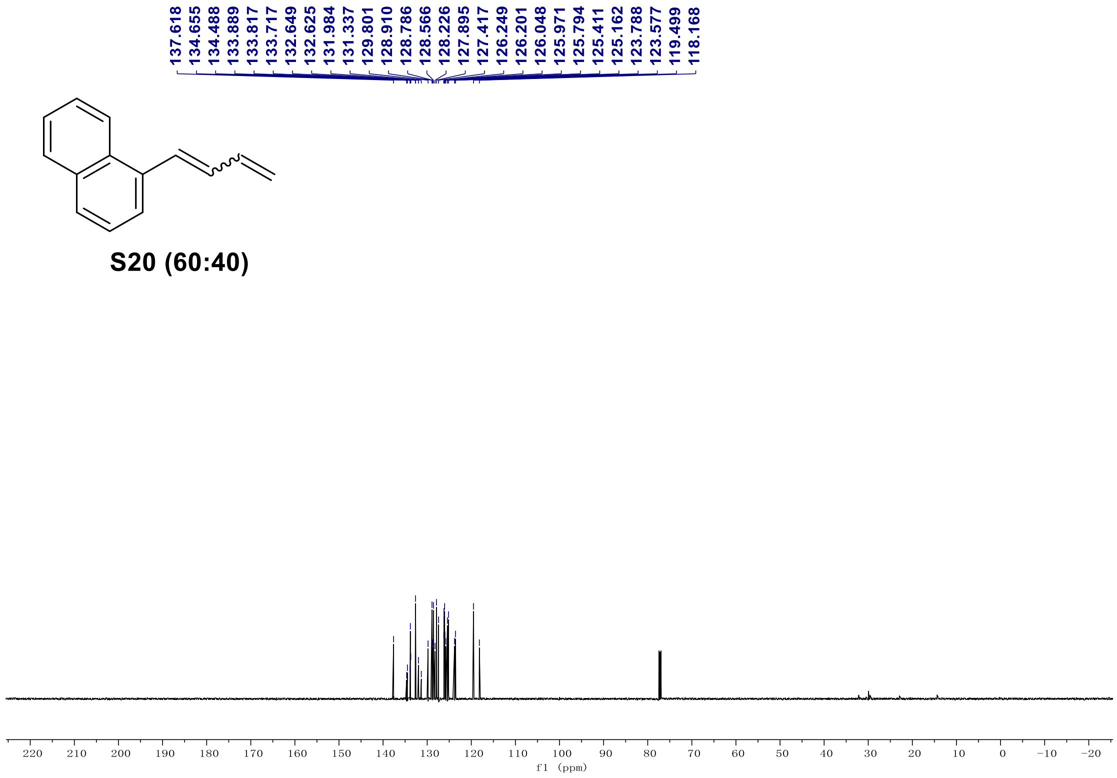

Supplementary Figure 48. ^{13}C NMR spectra of compound **S20**

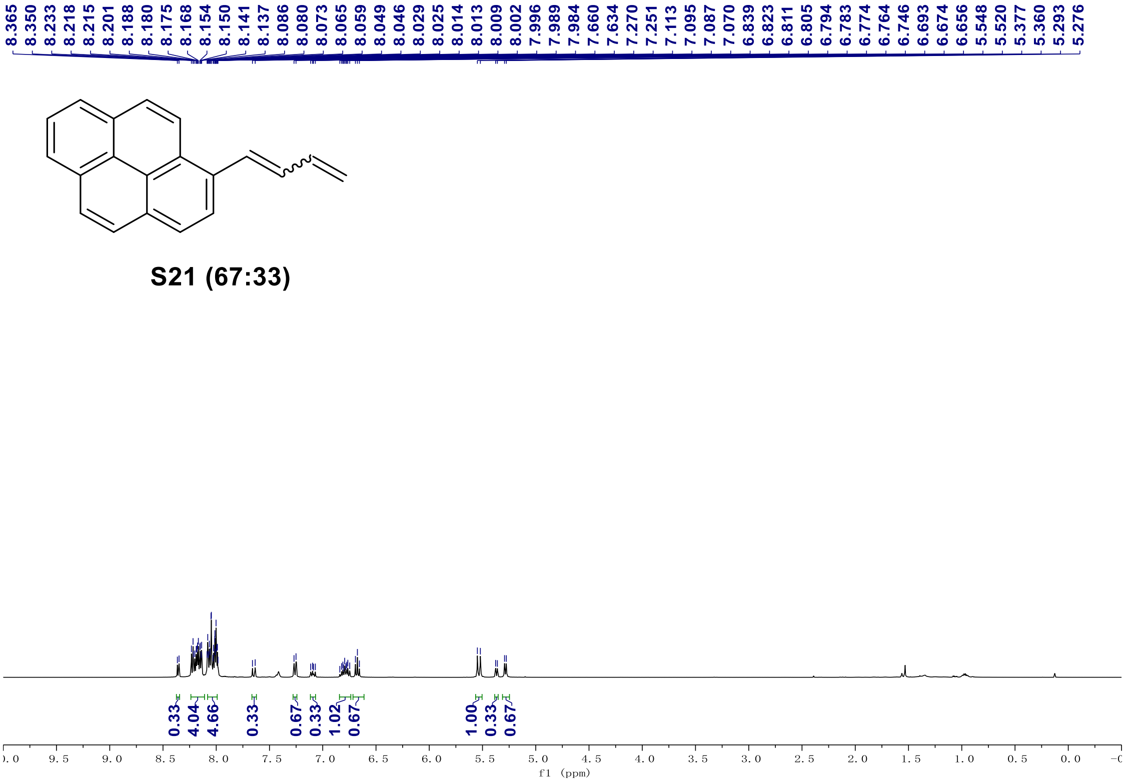

Supplementary Figure 49. ^{1}H NMR spectra of compound **S21**

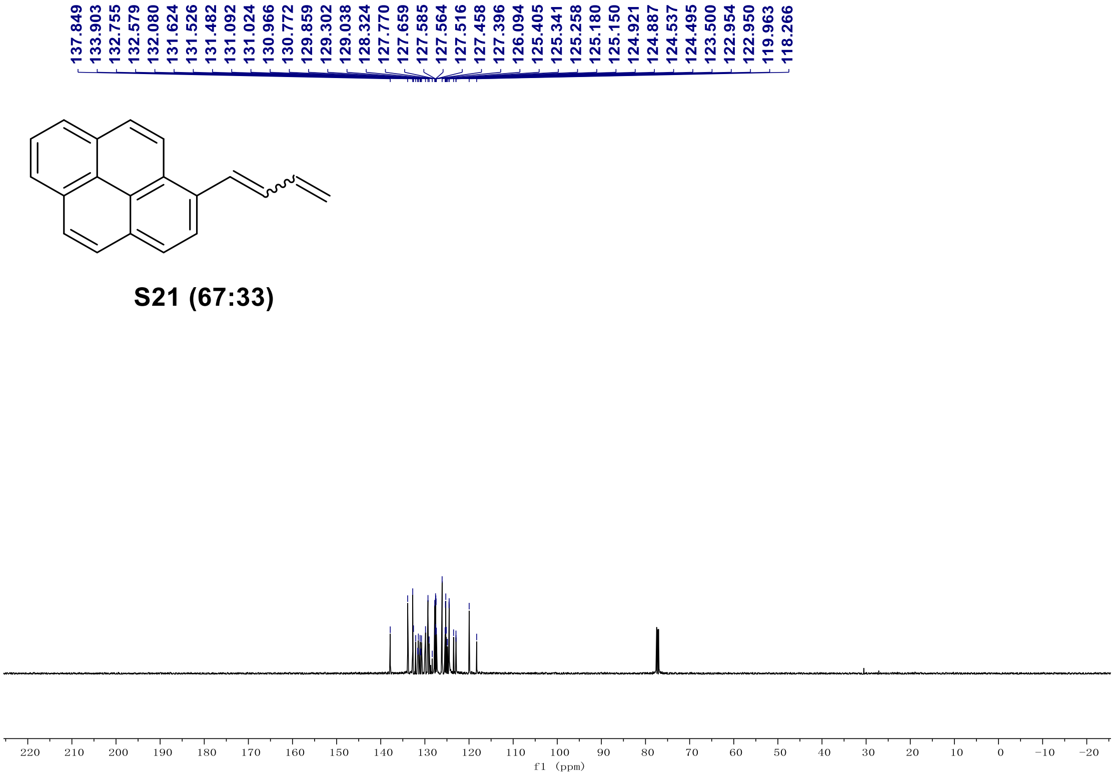

Supplementary Figure 50. ^{13}C NMR spectra of compound **S21**

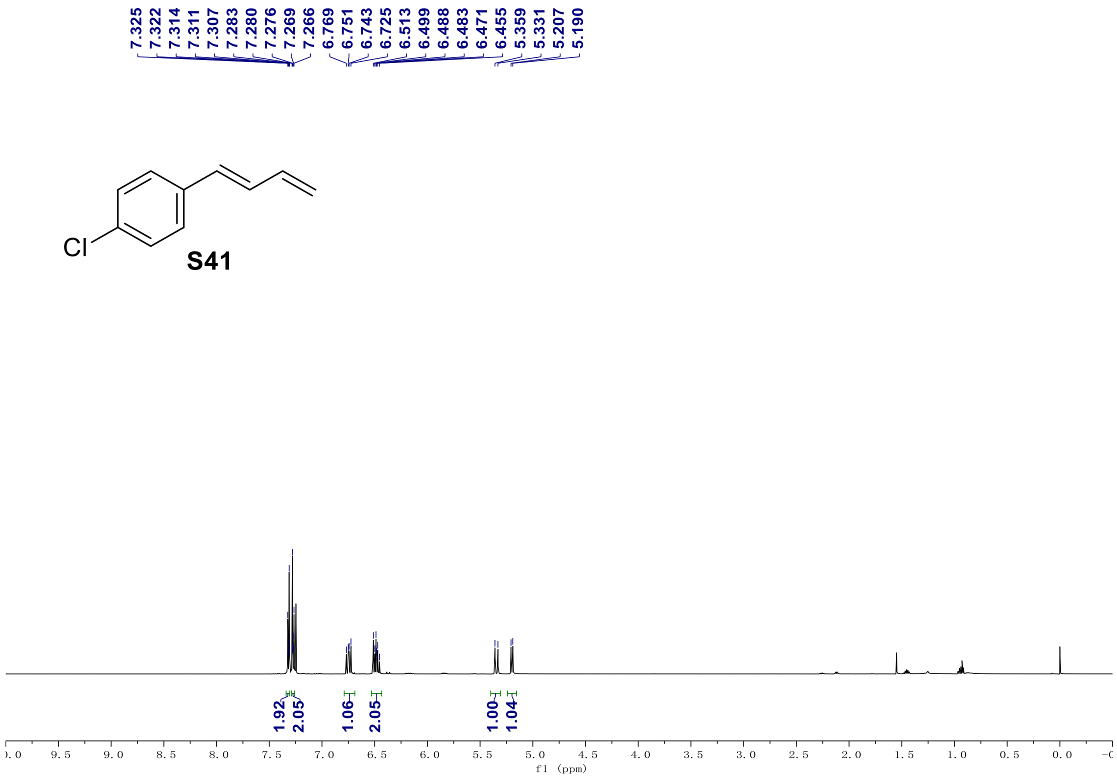

Supplementary Figure 51. ^{1}H NMR spectra of compound **S41**

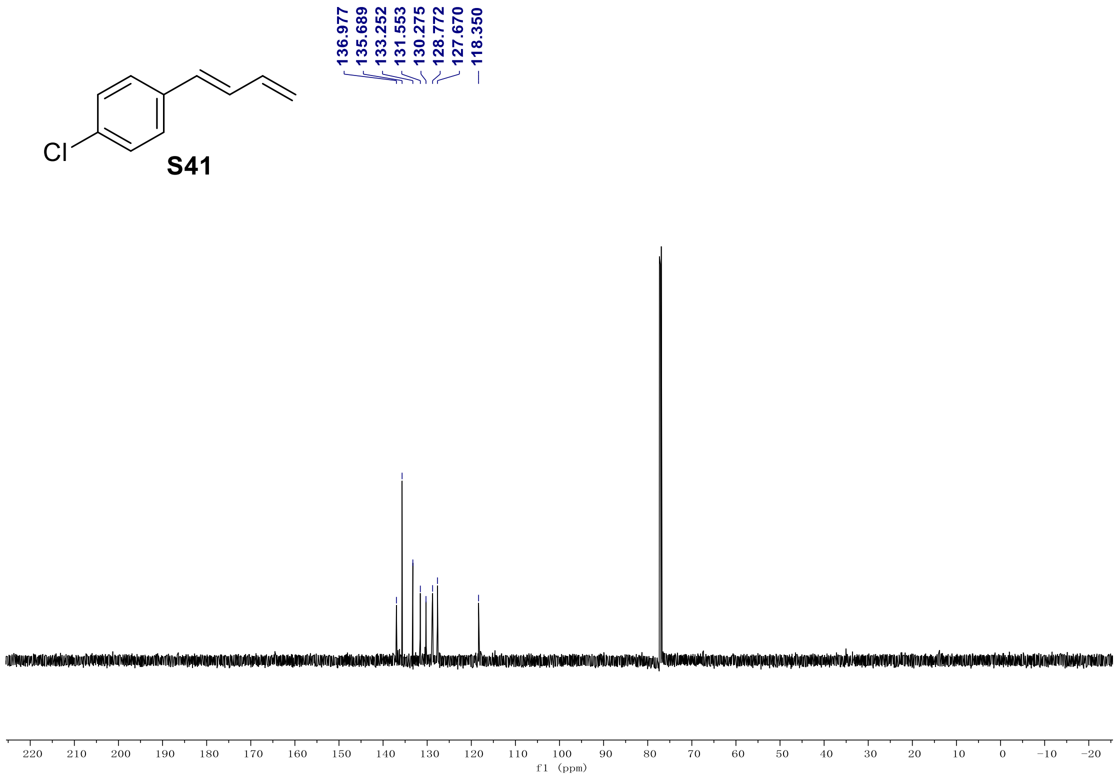

Supplementary Figure 52. ^{13}C NMR spectra of compound **S41**

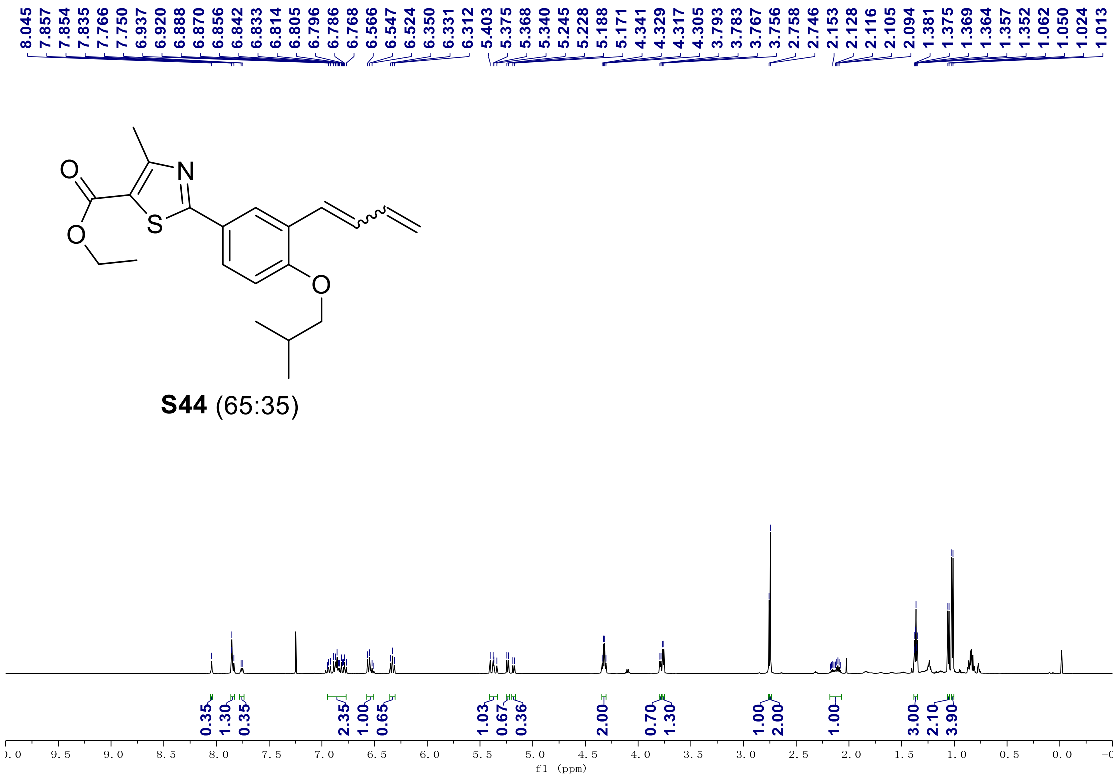

Supplementary Figure 53. ^{1}H NMR spectra of compound **S44**

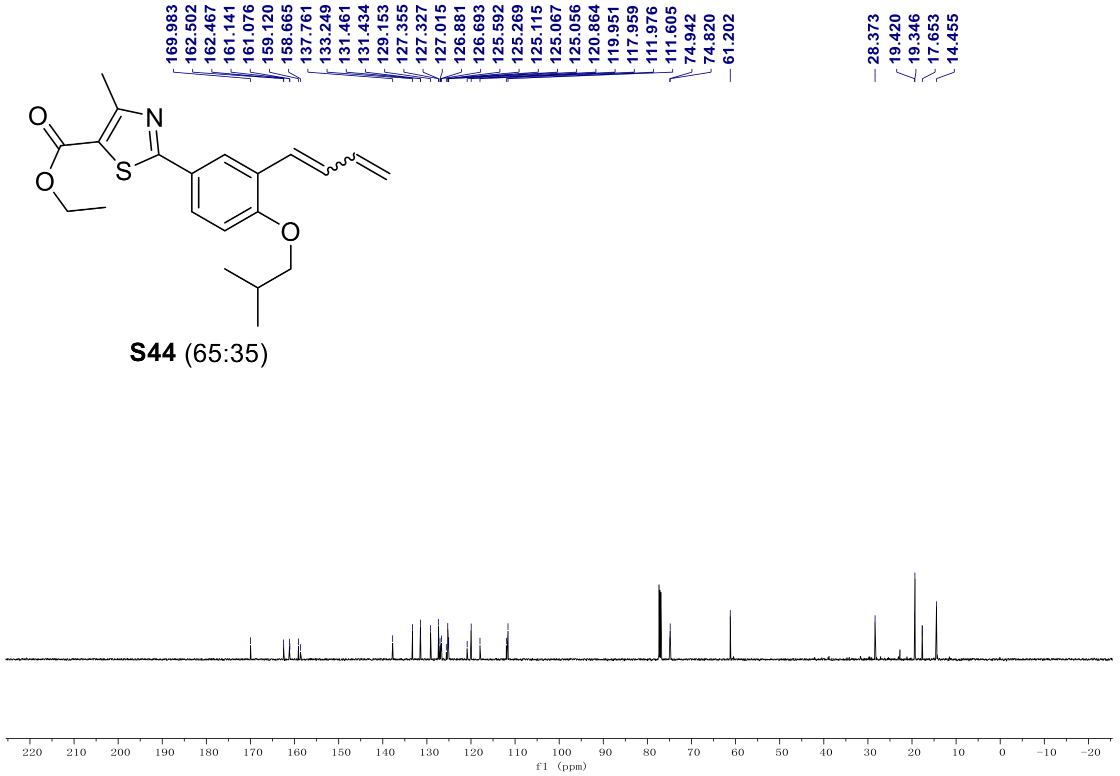

Supplementary Figure 54. ^{13}C NMR spectra of compound **S44**

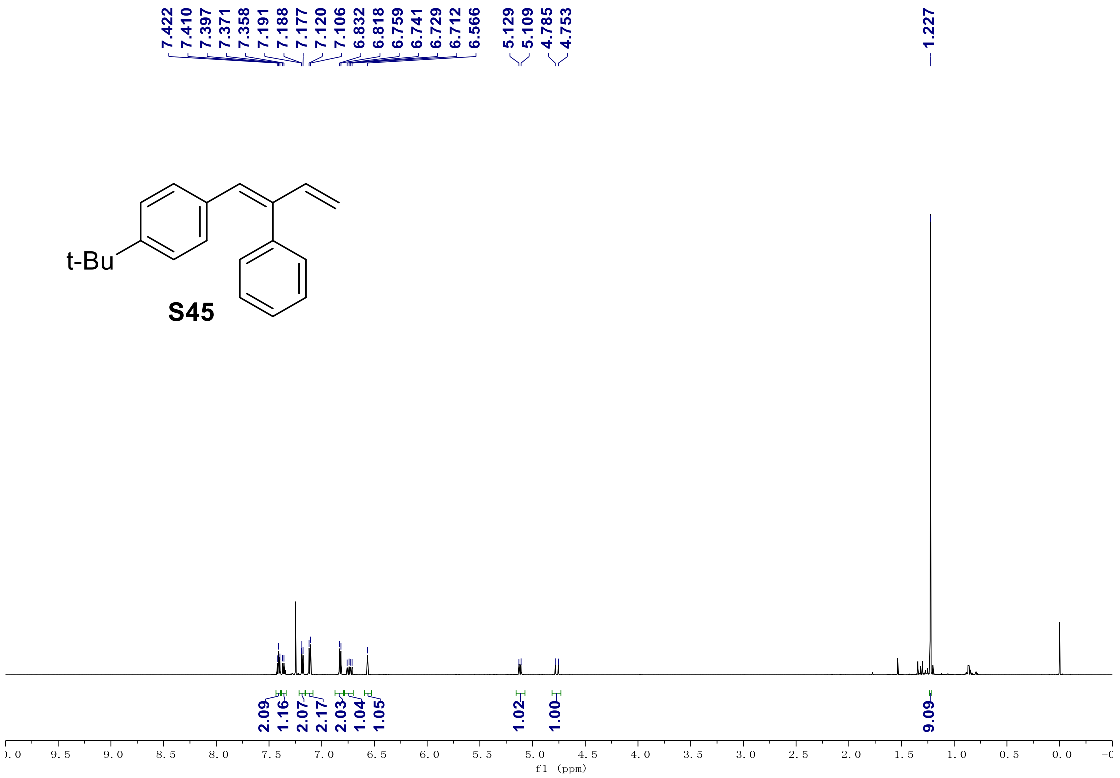

Supplementary Figure 55. ^{1}H NMR spectra of compound **S45**

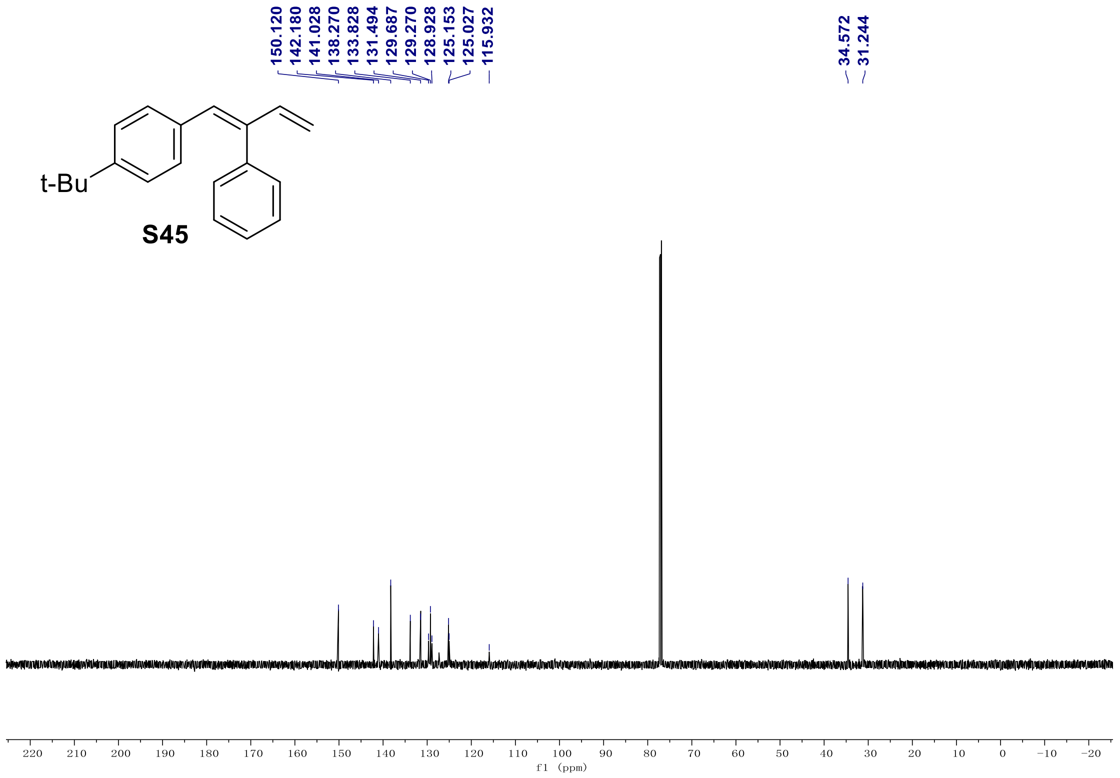

Supplementary Figure 56. ^{13}C NMR spectra of compound **S45**

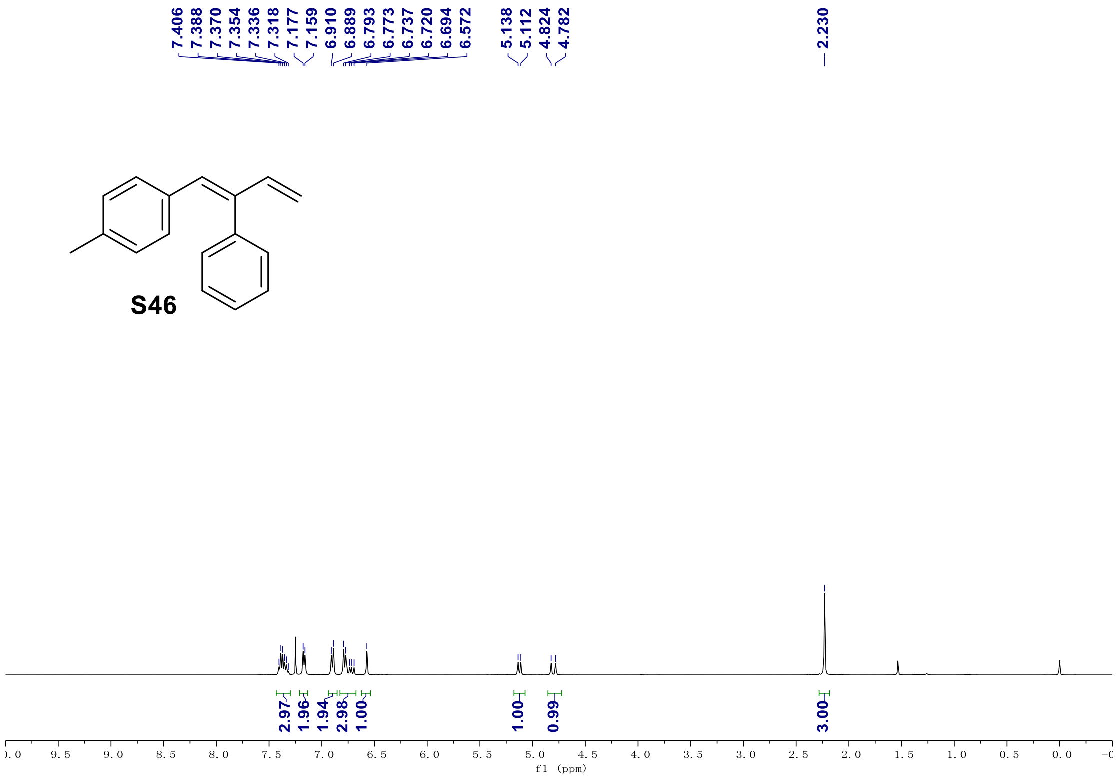

Supplementary Figure 57. ^{1}H NMR spectra of compound **S46**

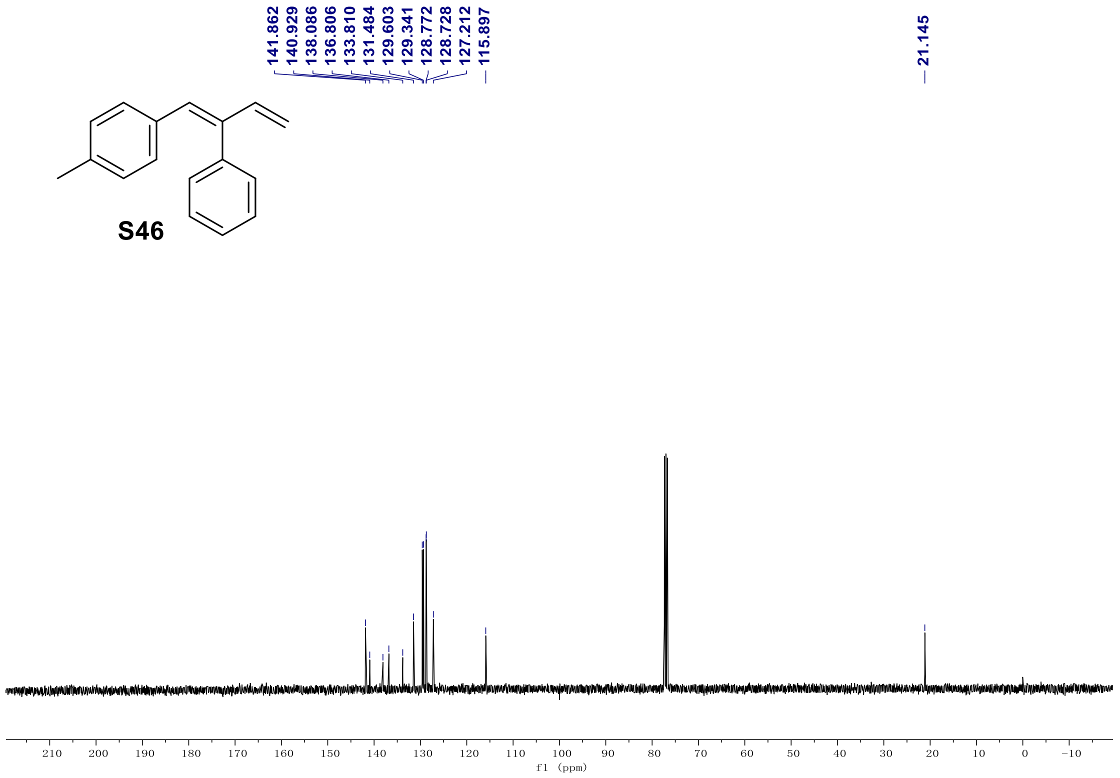

Supplementary Figure 58. ^{13}C NMR spectra of compound **S46**

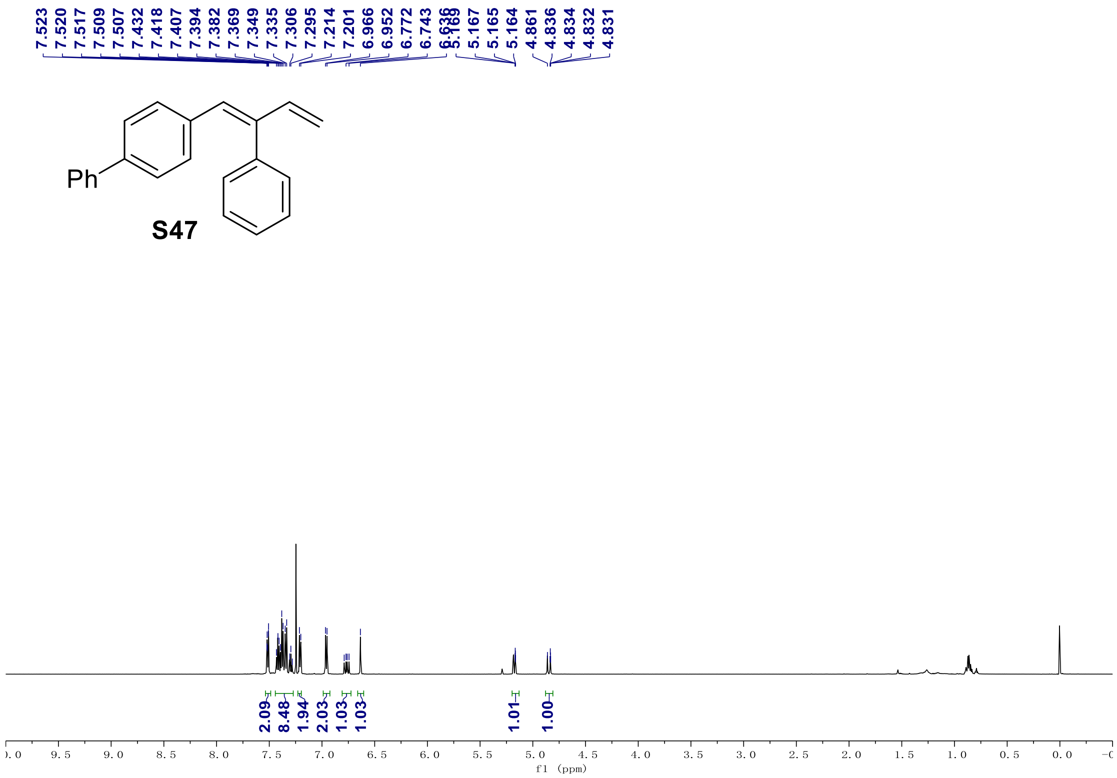

Supplementary Figure 59. ^{1}H NMR spectra of compound **S47**

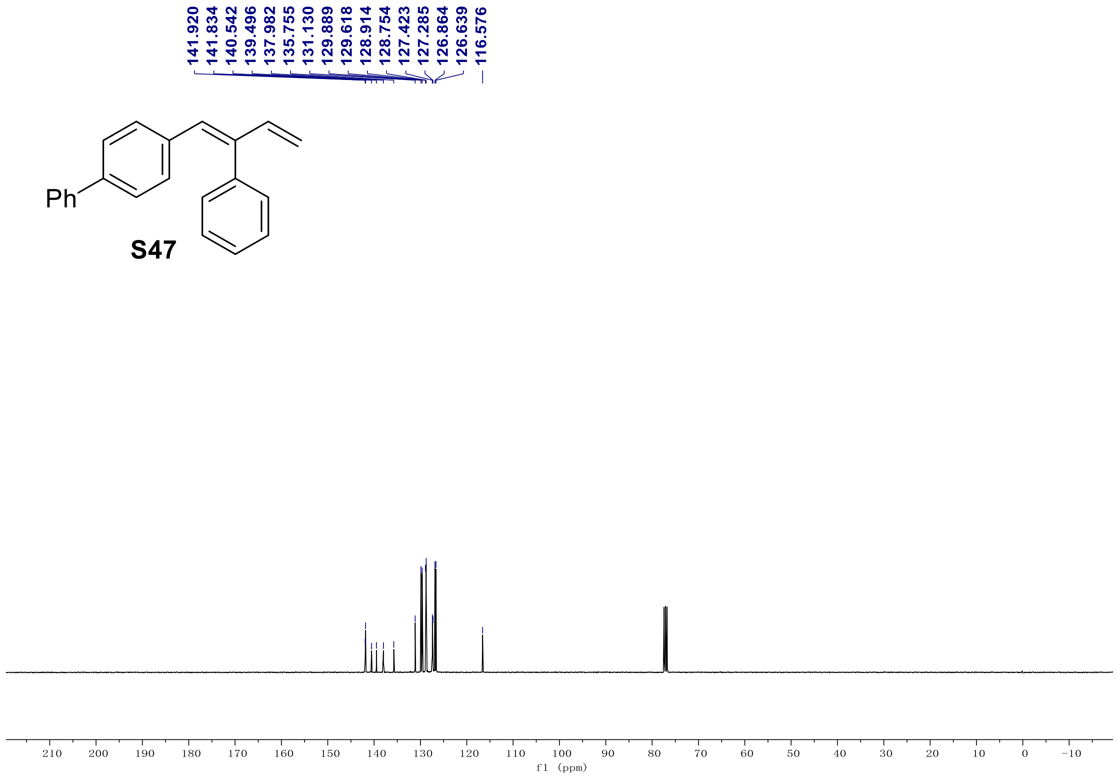

Supplementary Figure 60. ^{13}C NMR spectra of compound **S47**

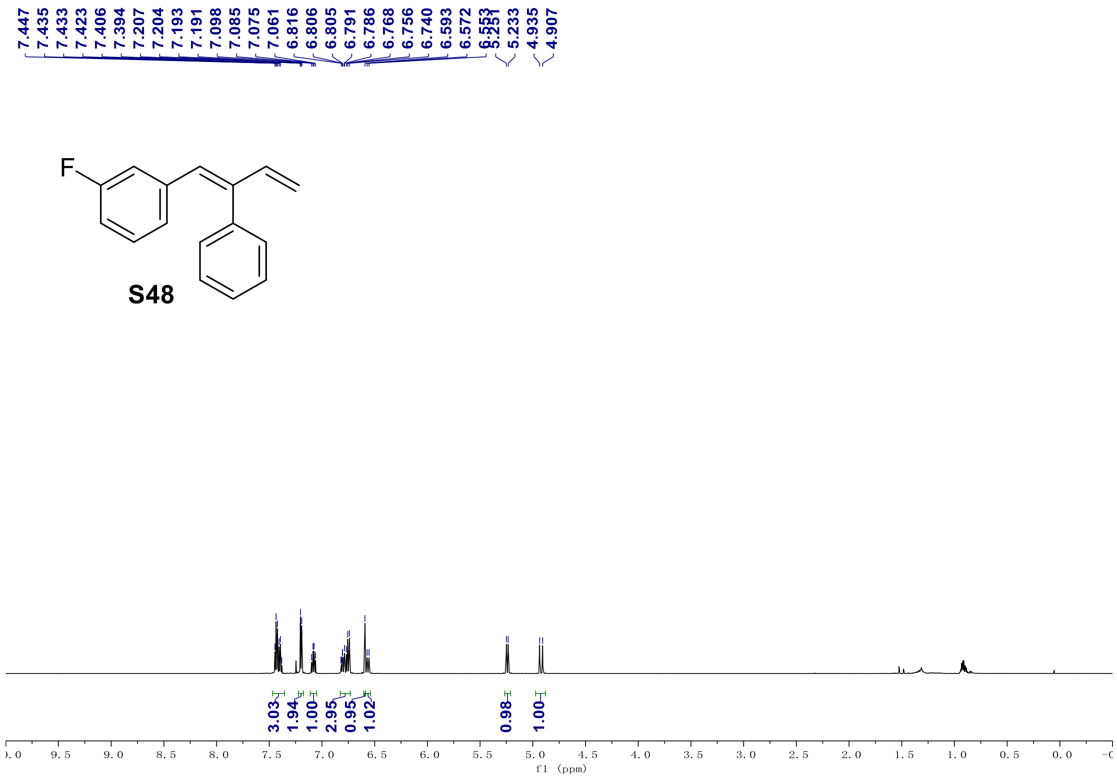

Supplementary Figure 61. ^{1}H NMR spectra of compound **S48**

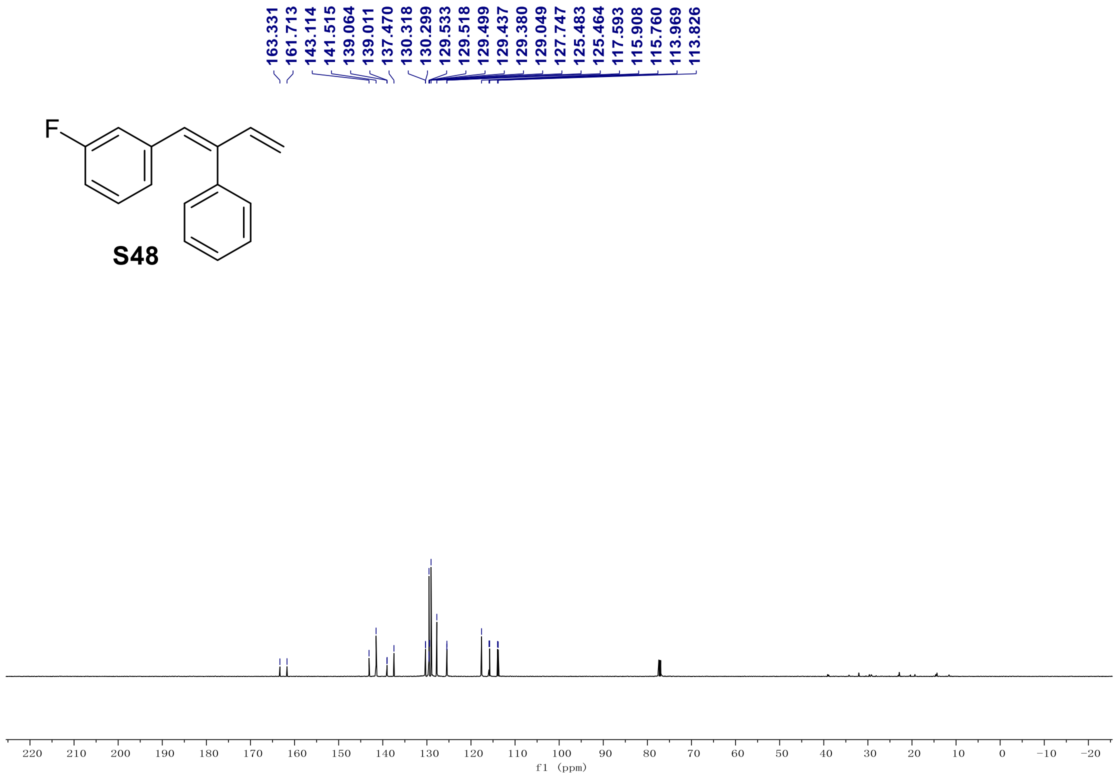

Supplementary Figure 62. ^{13}C NMR spectra of compound **S48**

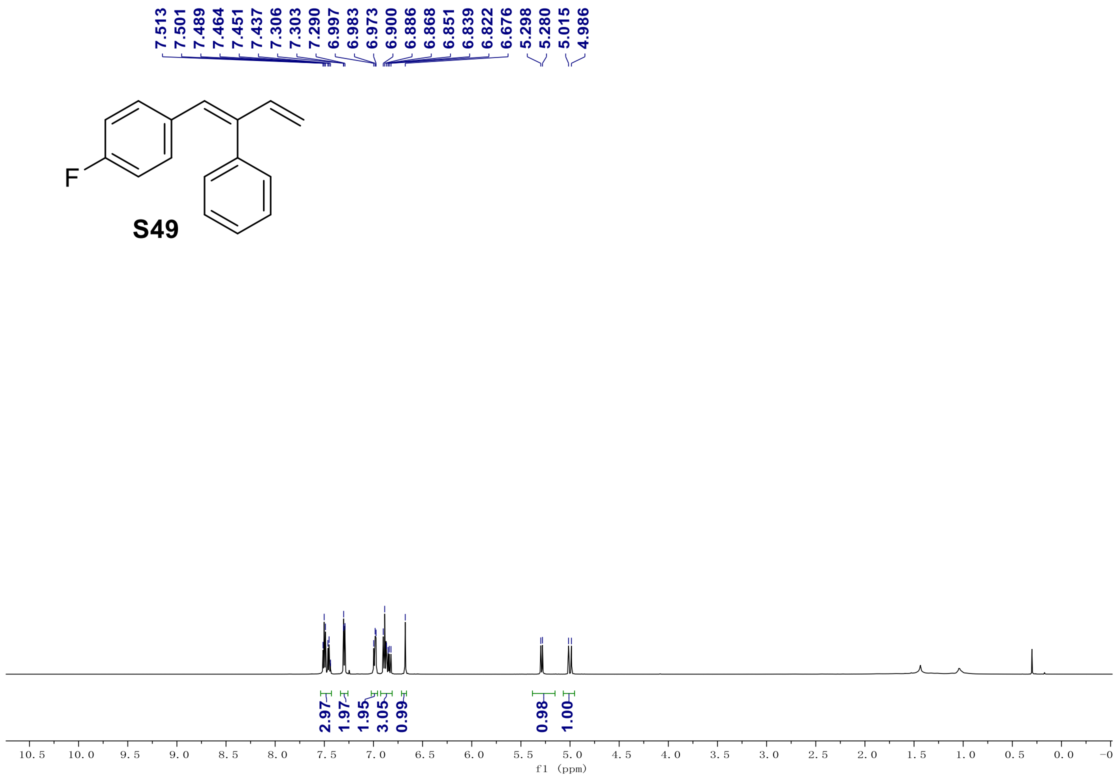

Supplementary Figure 63. ^{1}H NMR spectra of compound **S49**

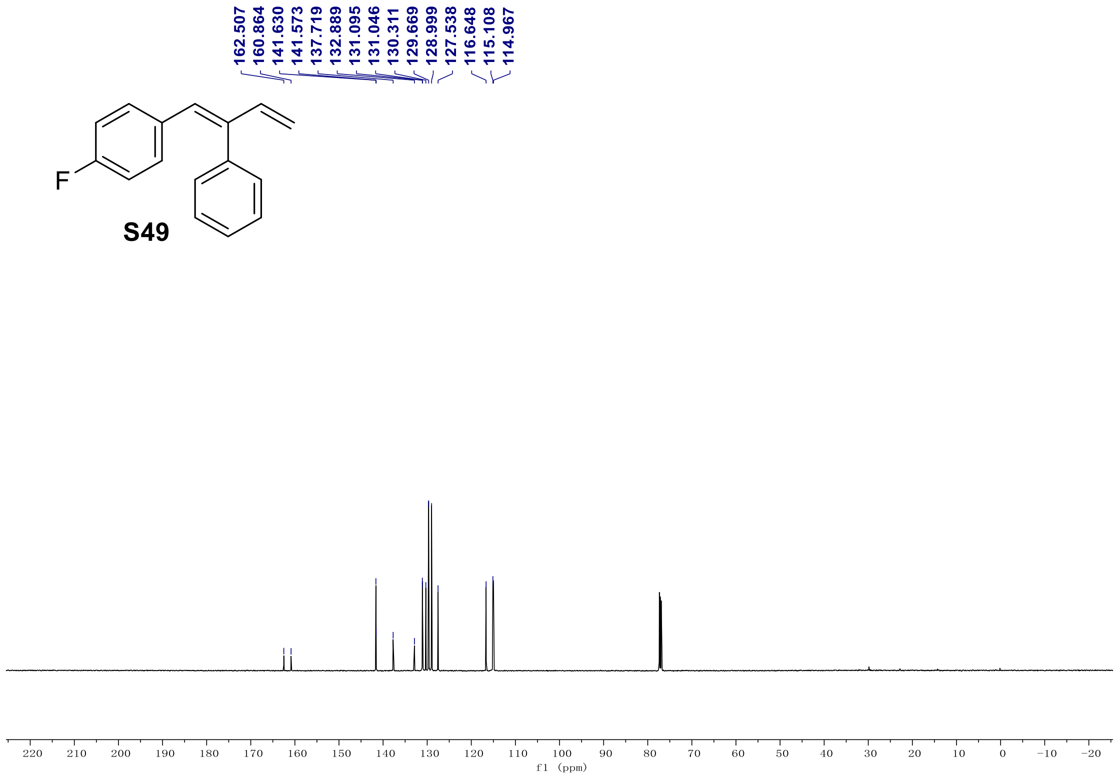

Supplementary Figure 64. ^{13}C NMR spectra of compound **S49**

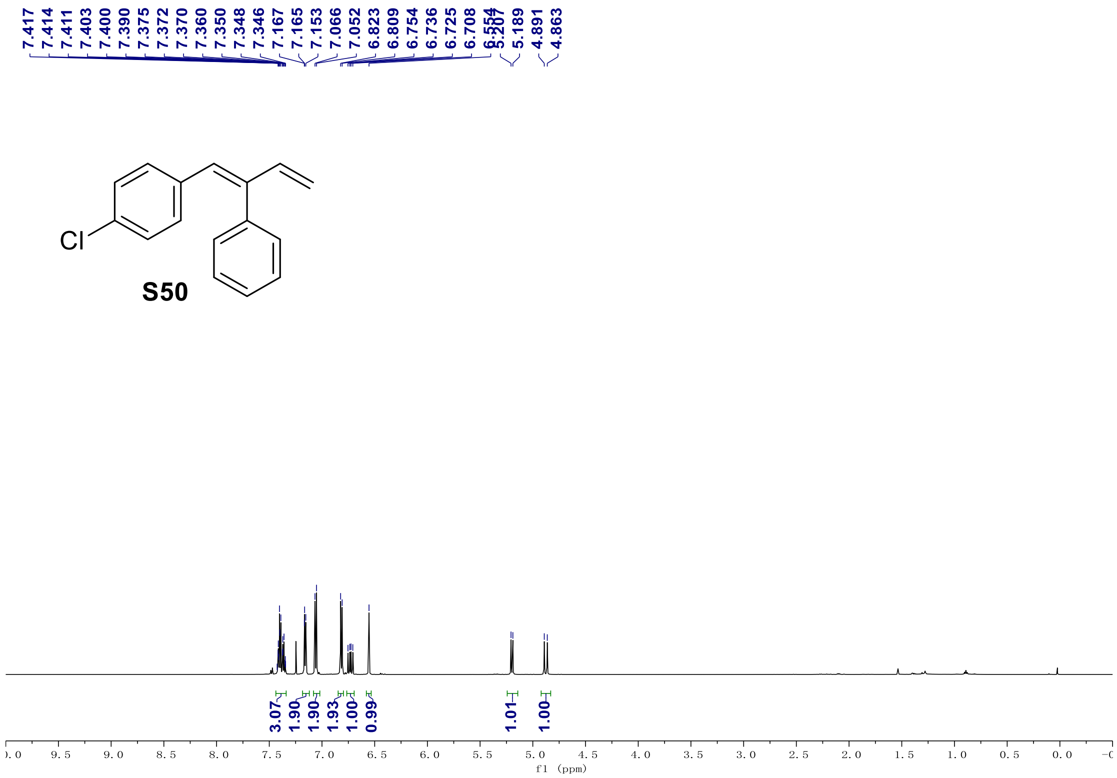

Supplementary Figure 65. ^{1}H NMR spectra of compound **S50**

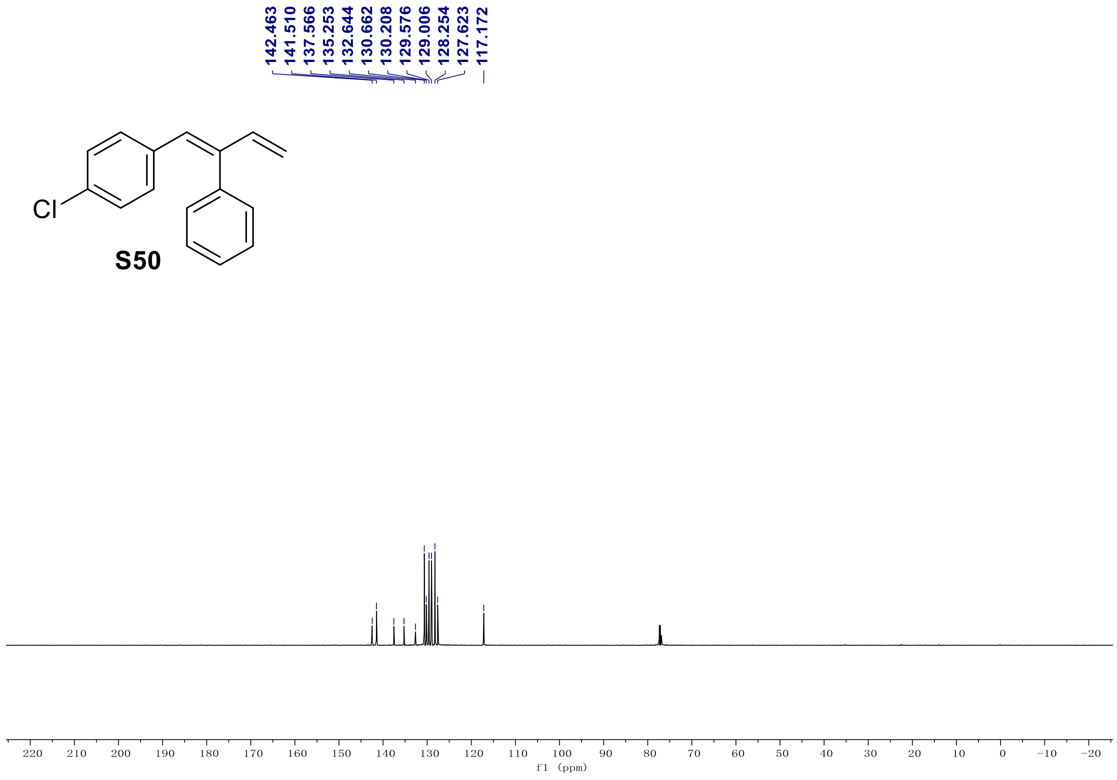

Supplementary Figure 66. ^{13}C NMR spectra of compound **S50**

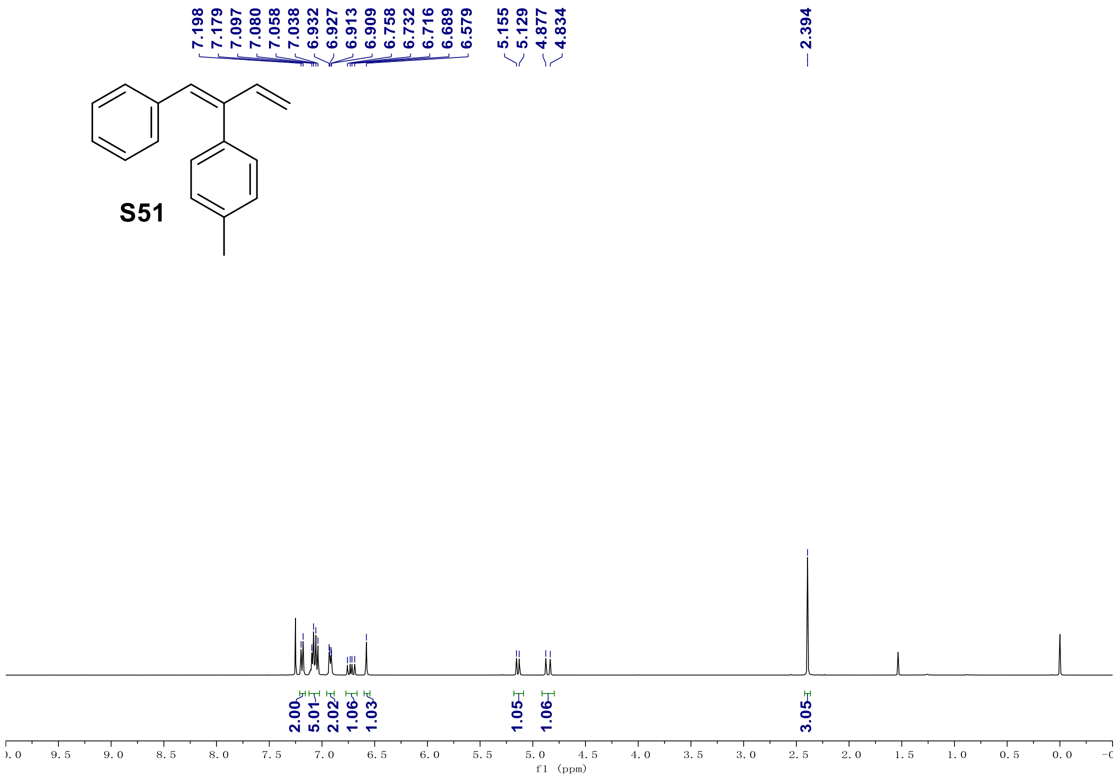

Supplementary Figure 67. ^{1}H NMR spectra of compound **S51**

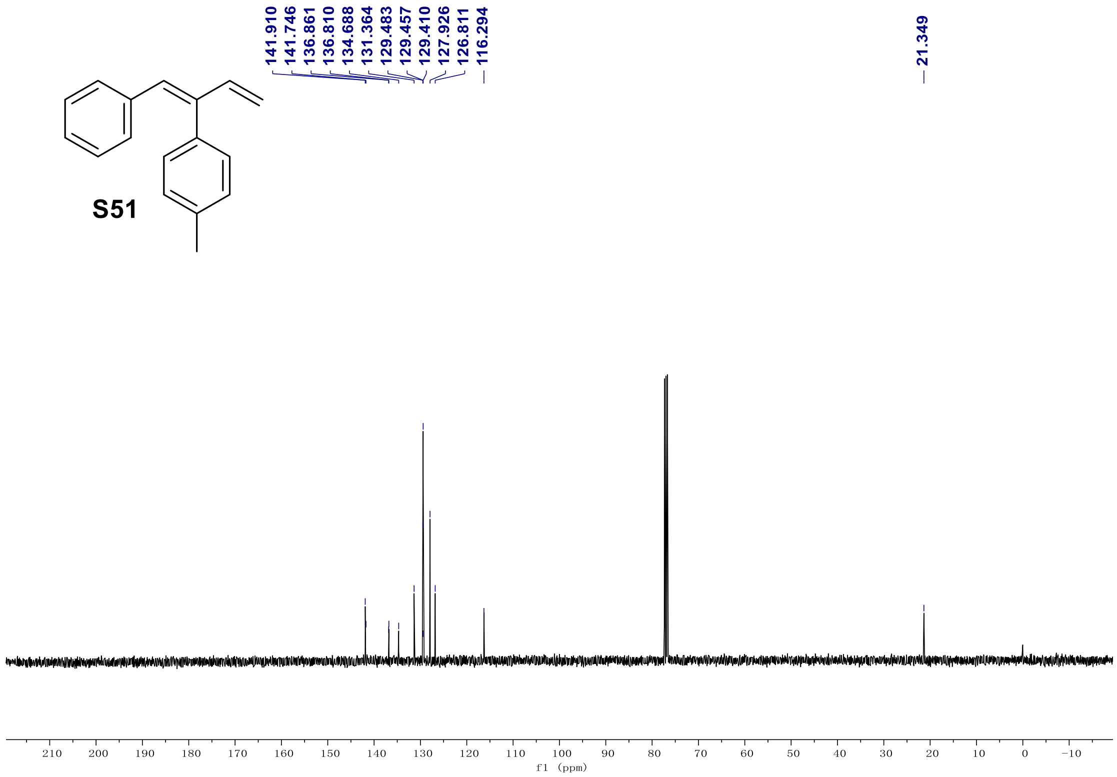

Supplementary Figure 68. ^{13}C NMR spectra of compound **S51**

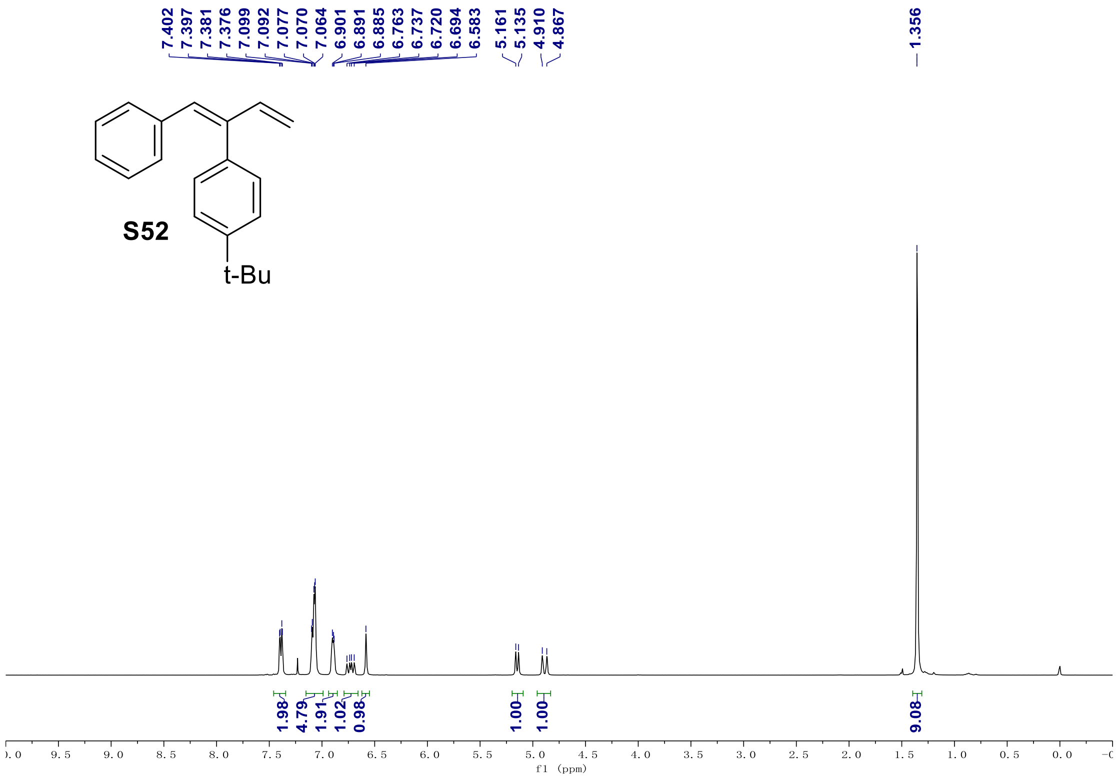

Supplementary Figure 69. ^{1}H NMR spectra of compound **S52**

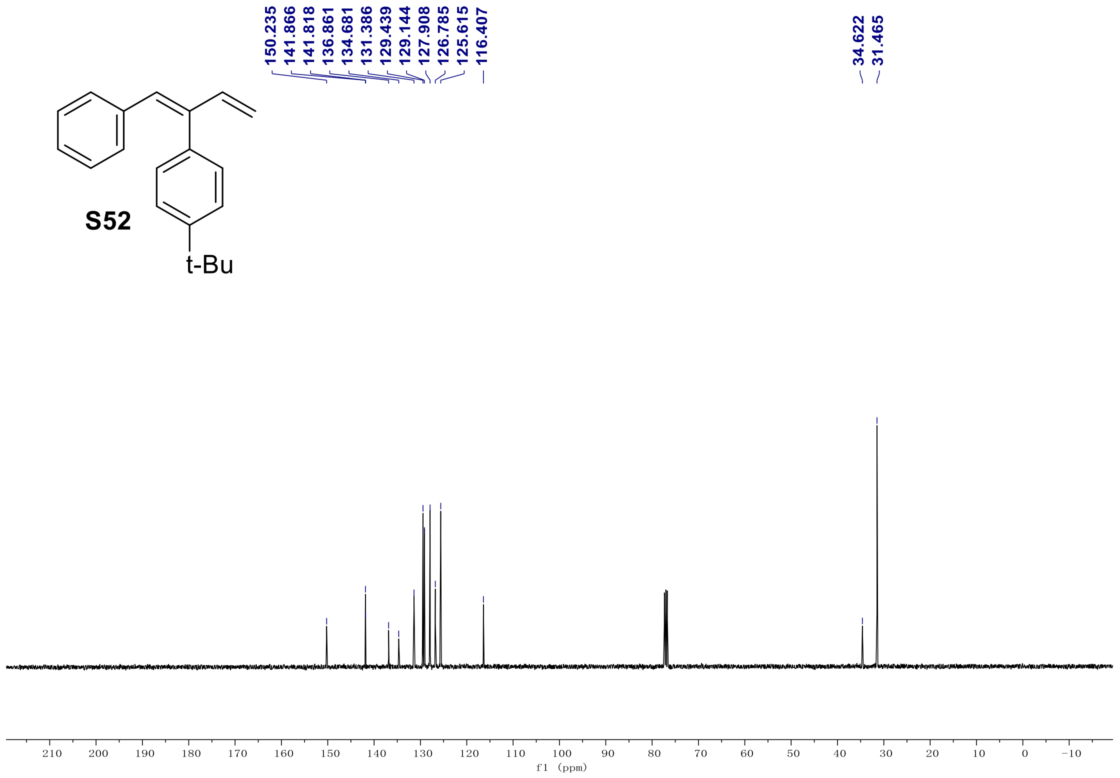

Supplementary Figure 70. ^{13}C NMR spectra of compound **S52**

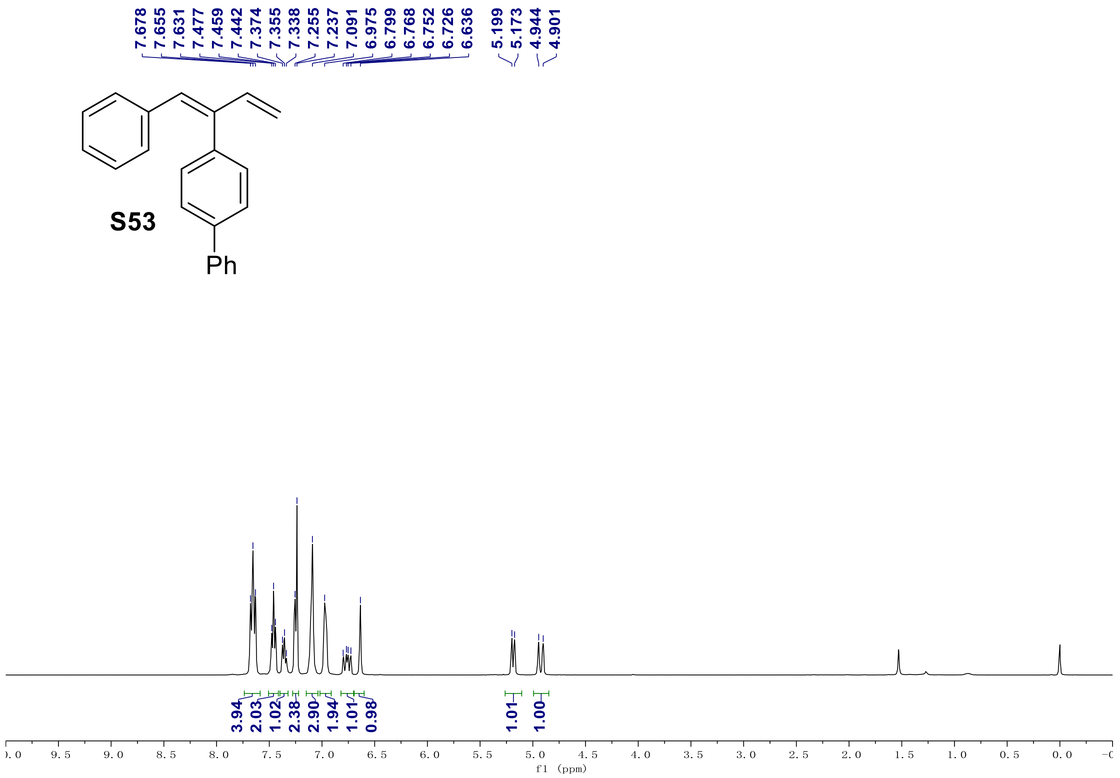

Supplementary Figure 71. ^{1}H NMR spectra of compound **S53**

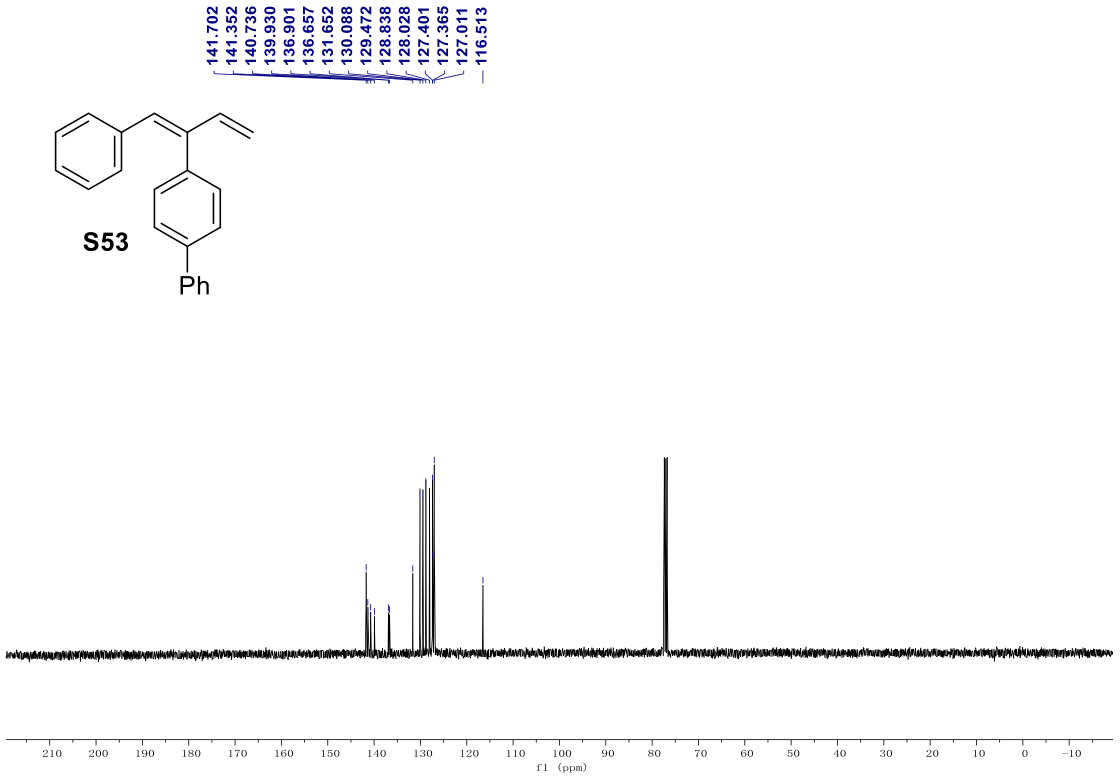

Supplementary Figure 72. ^{13}C NMR spectra of compound **S53**

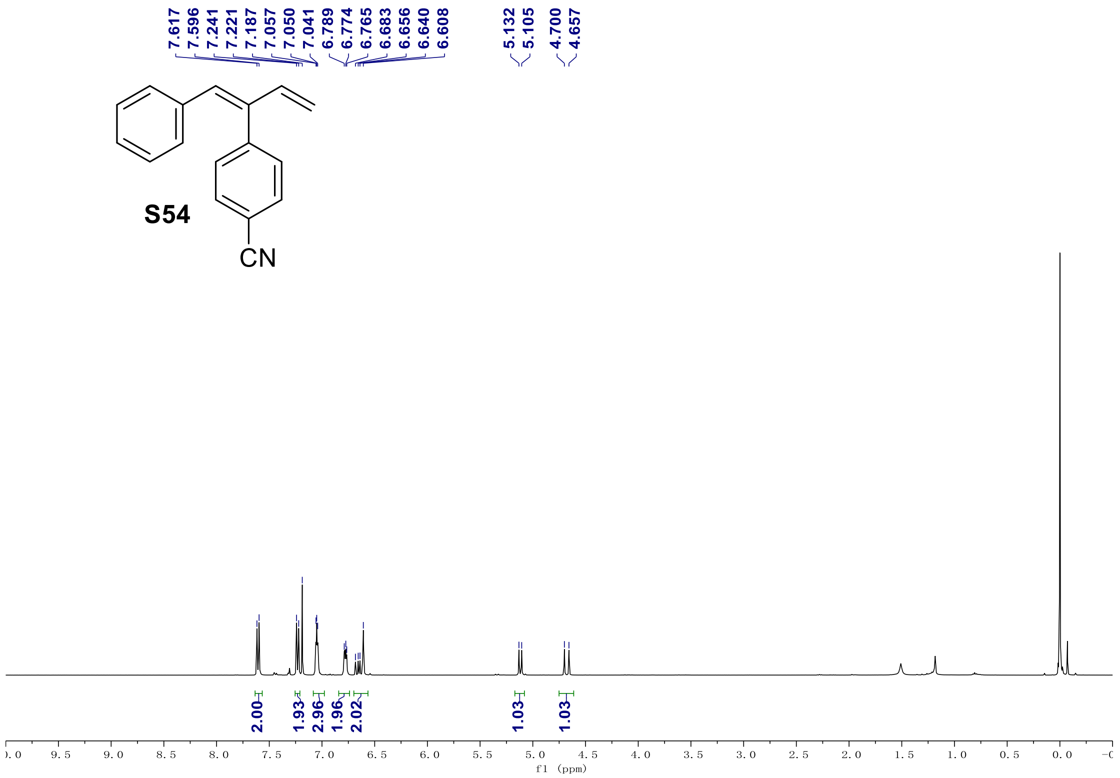

Supplementary Figure 73. ^{1}H NMR spectra of compound **S54**

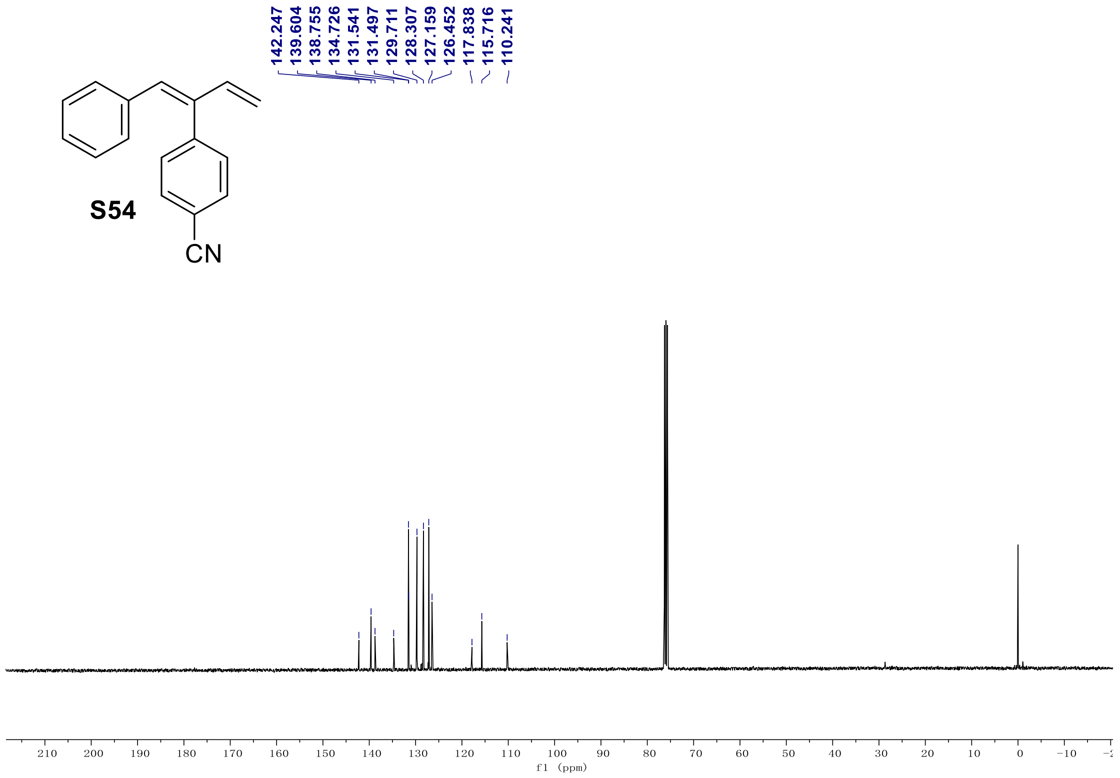

Supplementary Figure 74. ^{13}C NMR spectra of compound **S54**

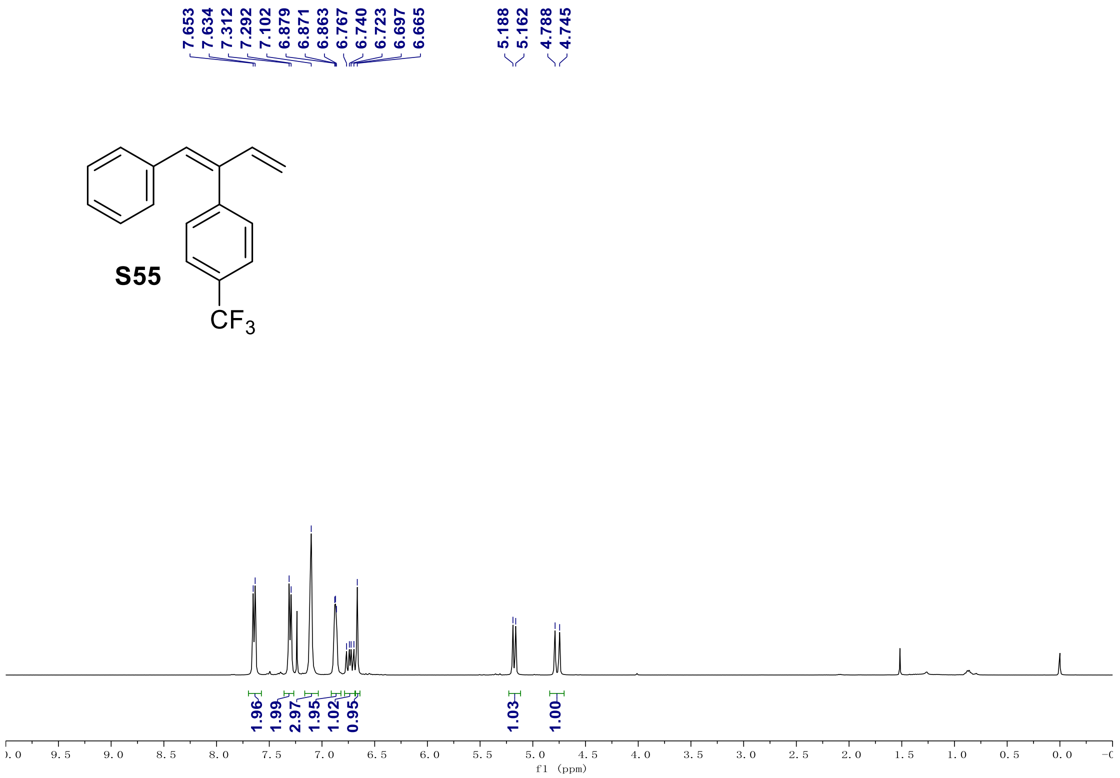

Supplementary Figure 75. ^{1}H NMR spectra of compound **S55**

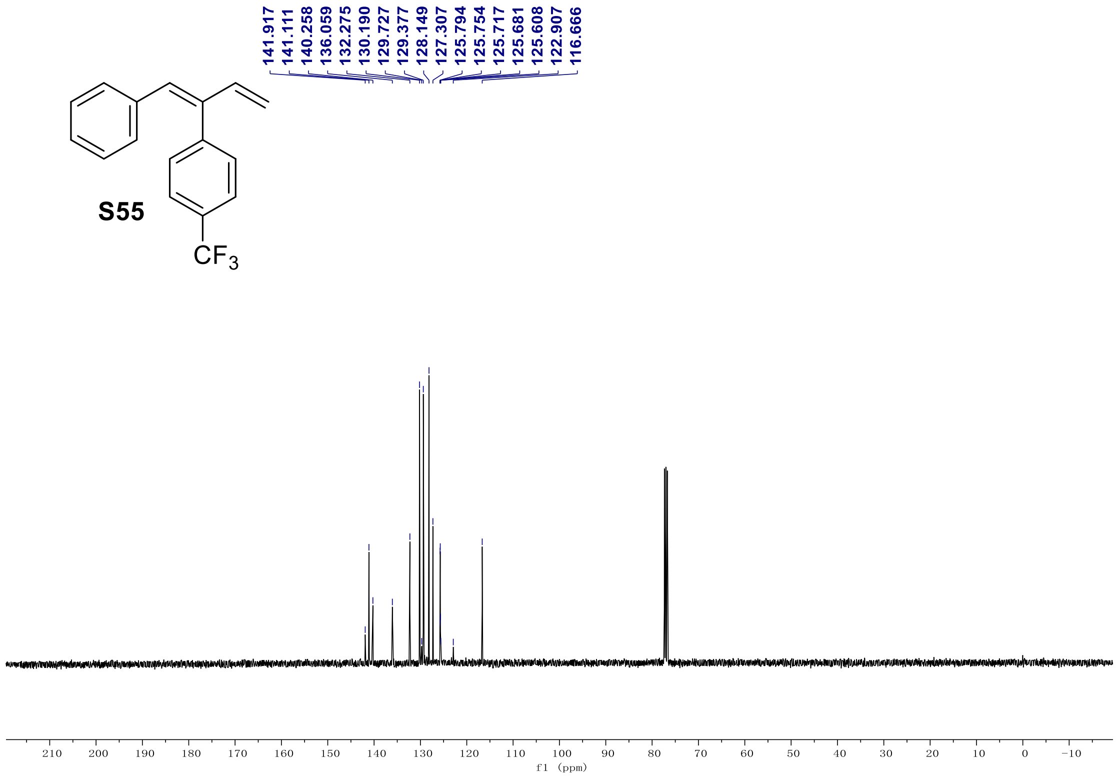

Supplementary Figure 76. ^{13}C NMR spectra of compound **S55**

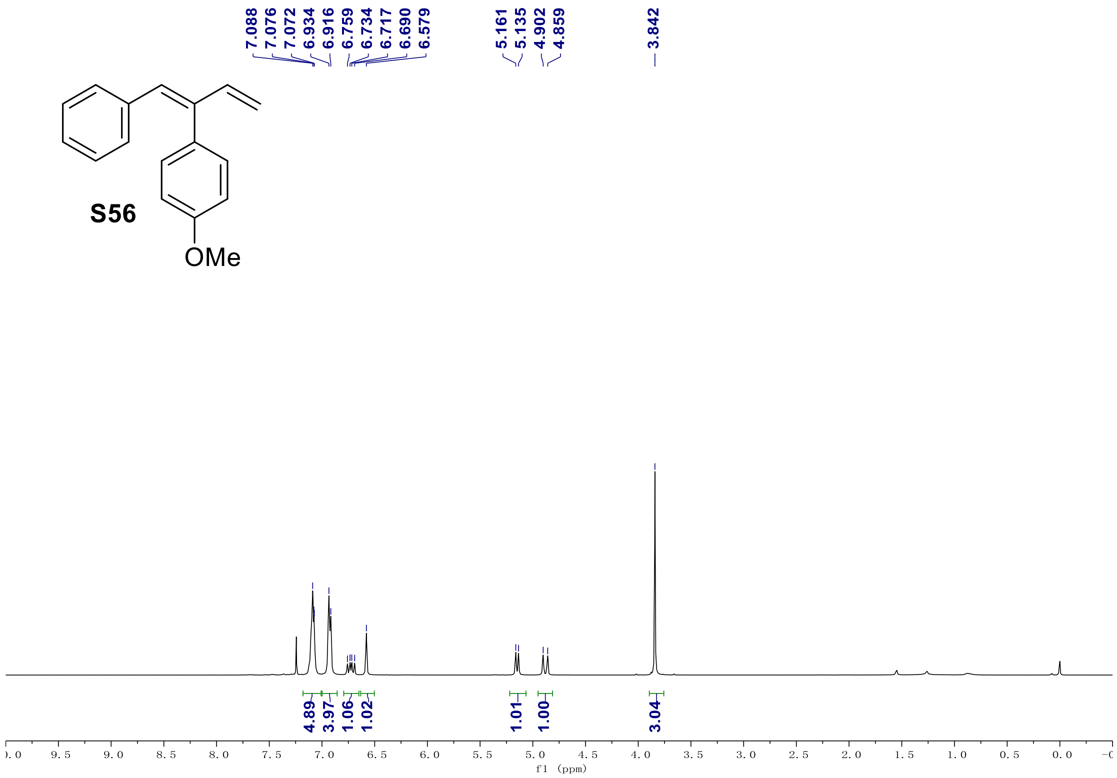

Supplementary Figure 77. ^{1}H NMR spectra of compound **S56**

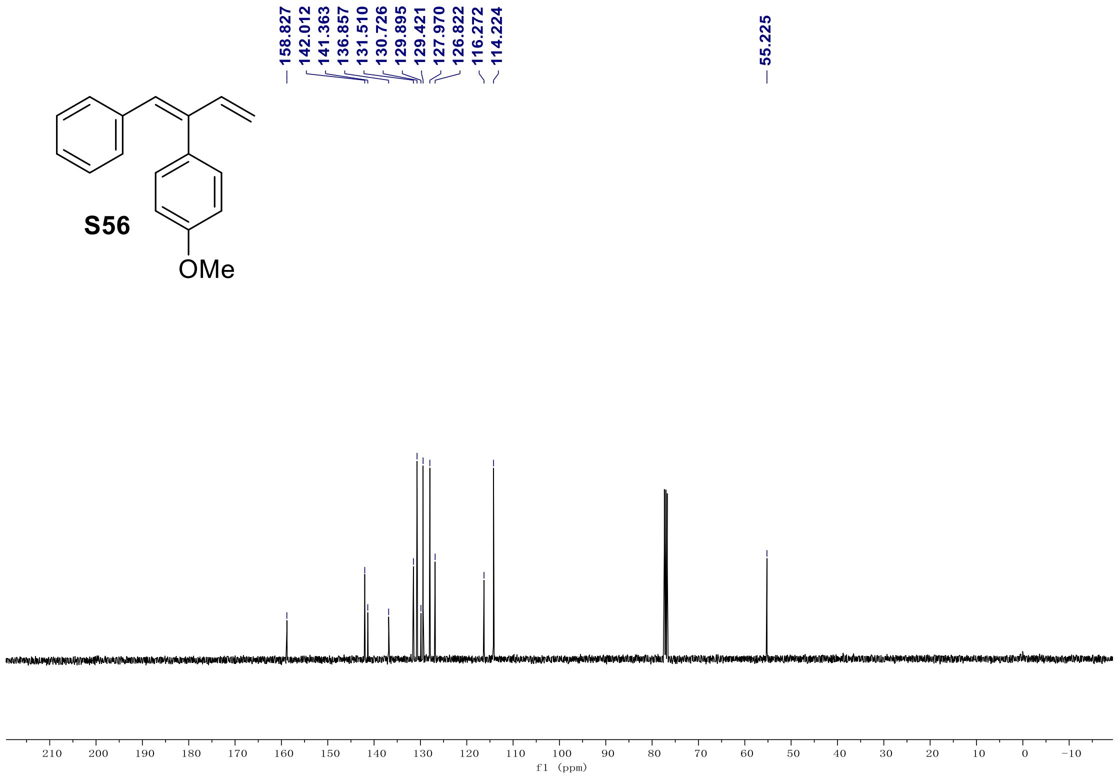

Supplementary Figure 78. ^{13}C NMR spectra of compound **S56**

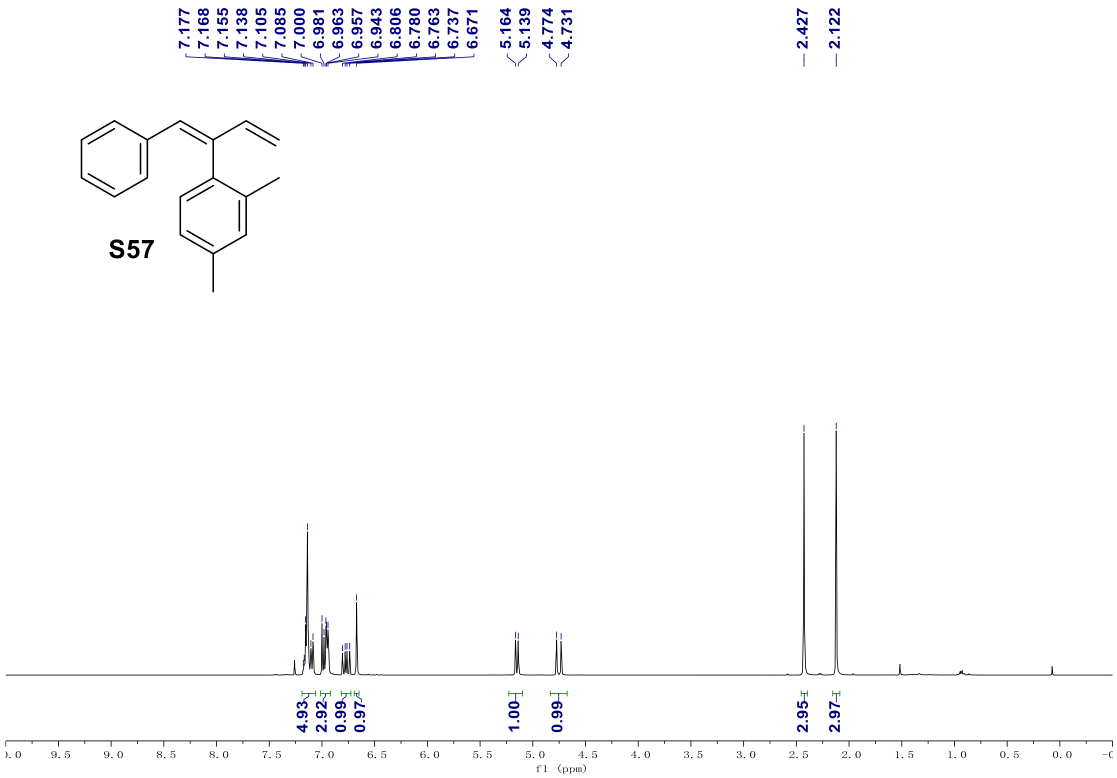

Supplementary Figure 79. ¹H NMR spectra of compound **S57**

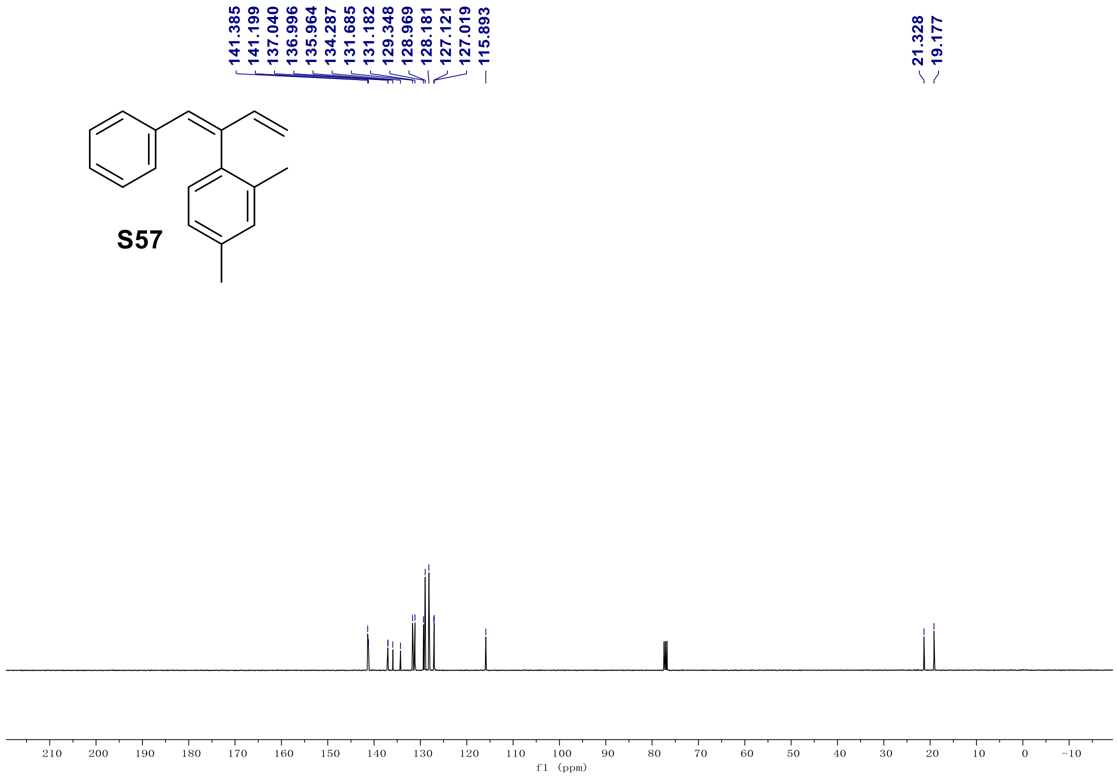

Supplementary Figure 80. ¹³C NMR spectra of compound **S57**

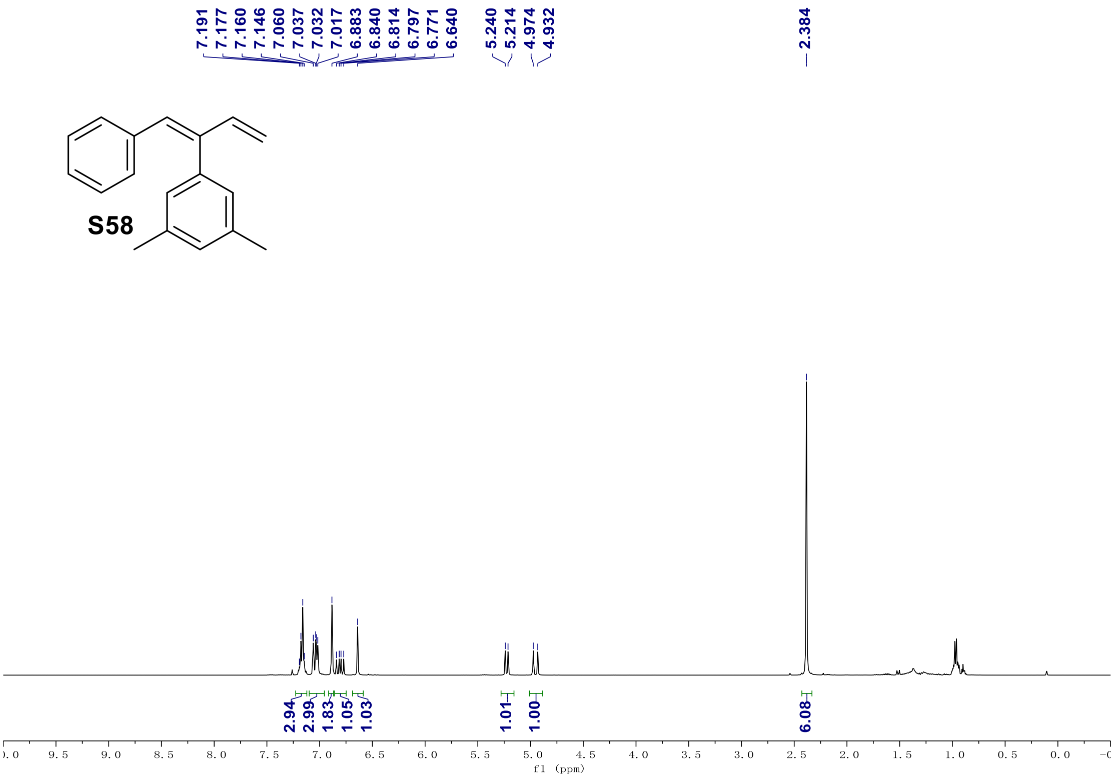

Supplementary Figure 81. ^{1}H NMR spectra of compound **S58**

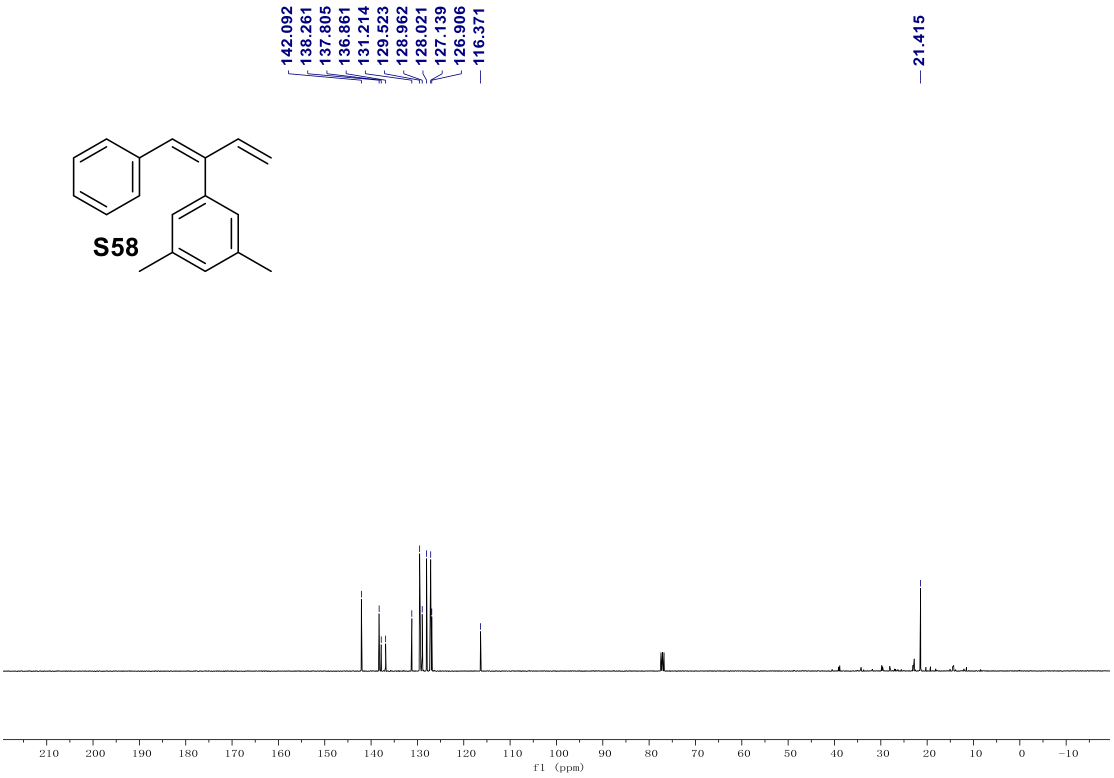

Supplementary Figure 82. ^{13}C NMR spectra of compound **S58**

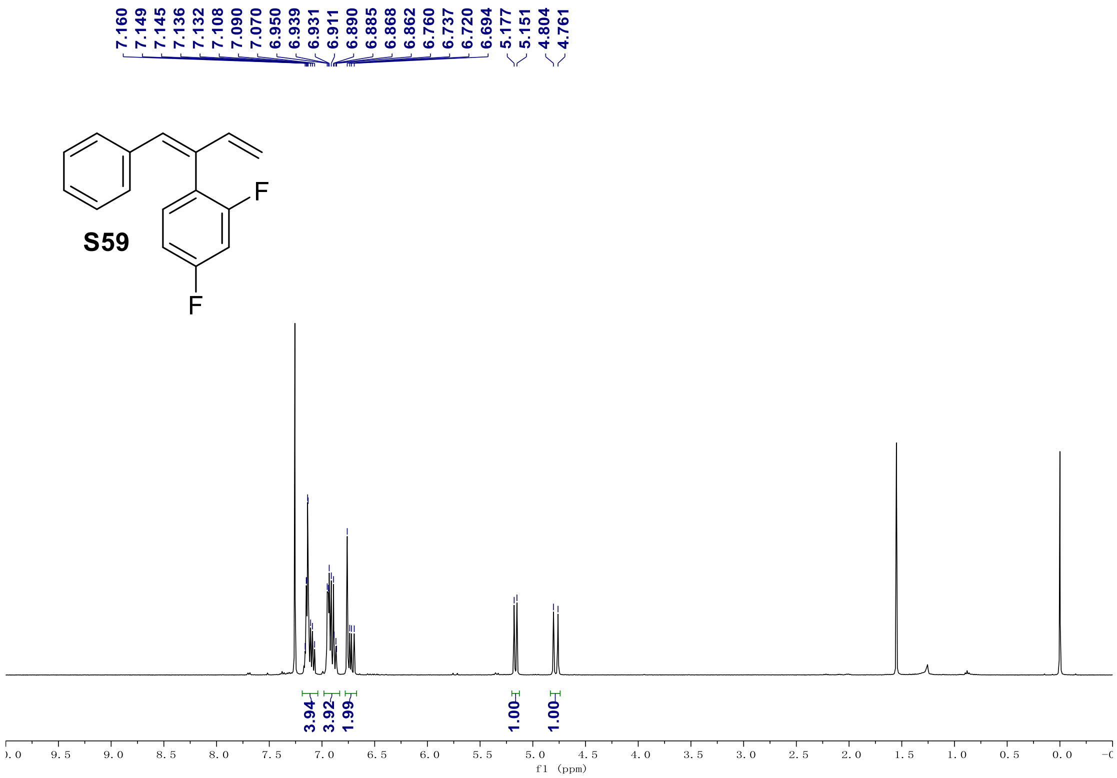

Supplementary Figure 83. ^{1}H NMR spectra of compound **S59**

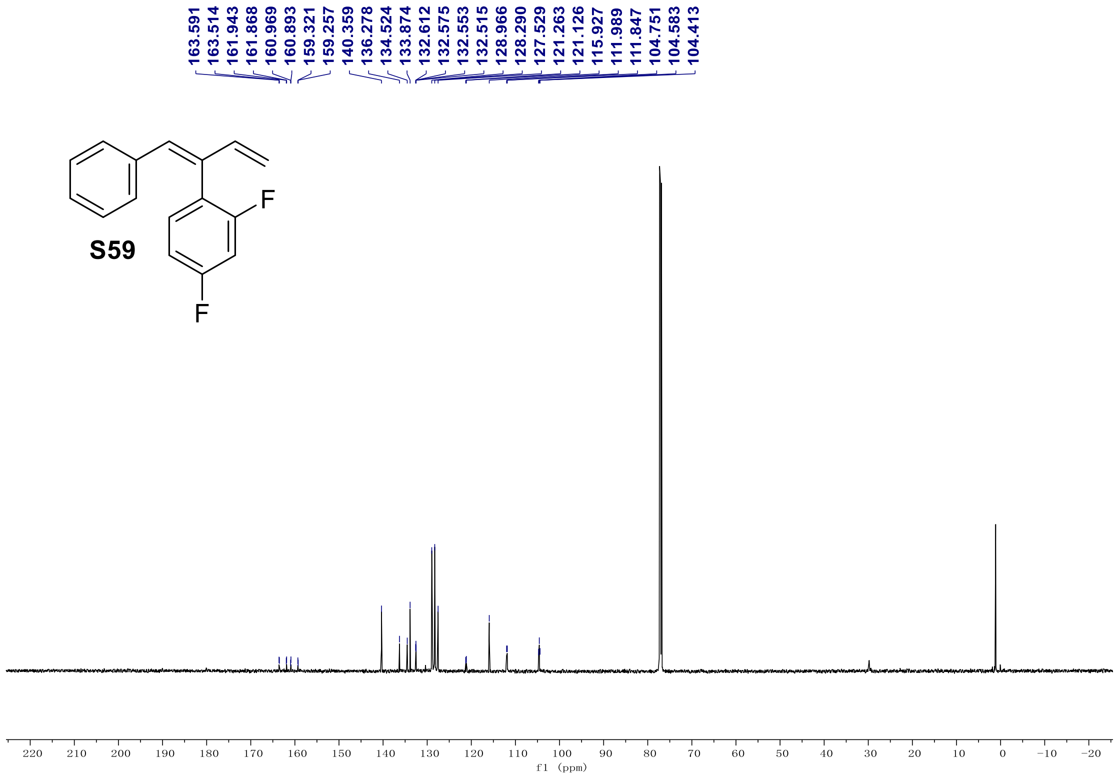

Supplementary Figure 84. ^{13}C NMR spectra of compound **S59**

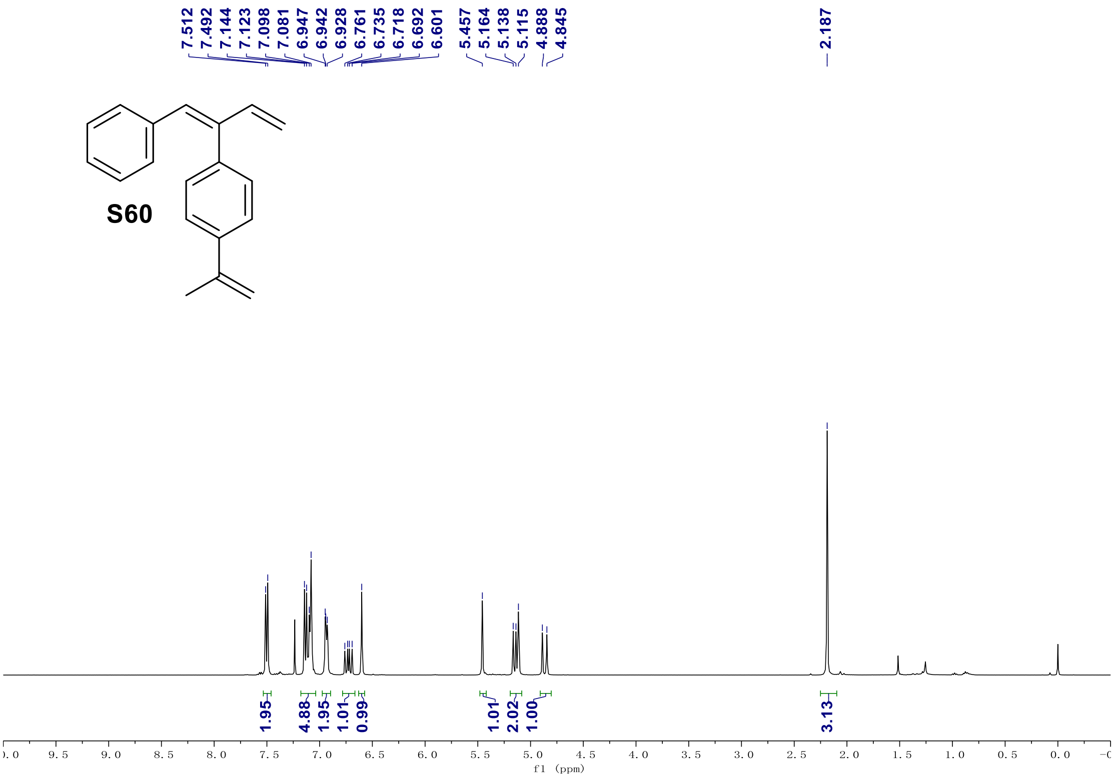

Supplementary Figure 85. ^{1}H NMR spectra of compound **S60**

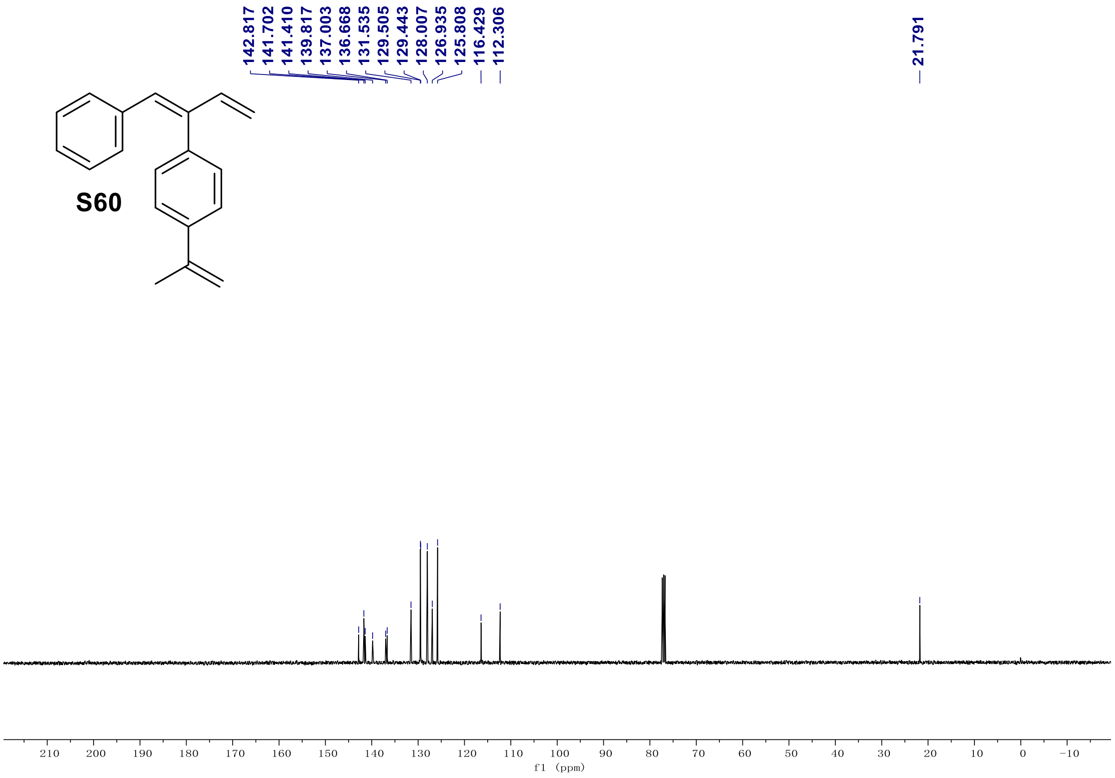

Supplementary Figure 86. ^{13}C NMR spectra of compound **S60**

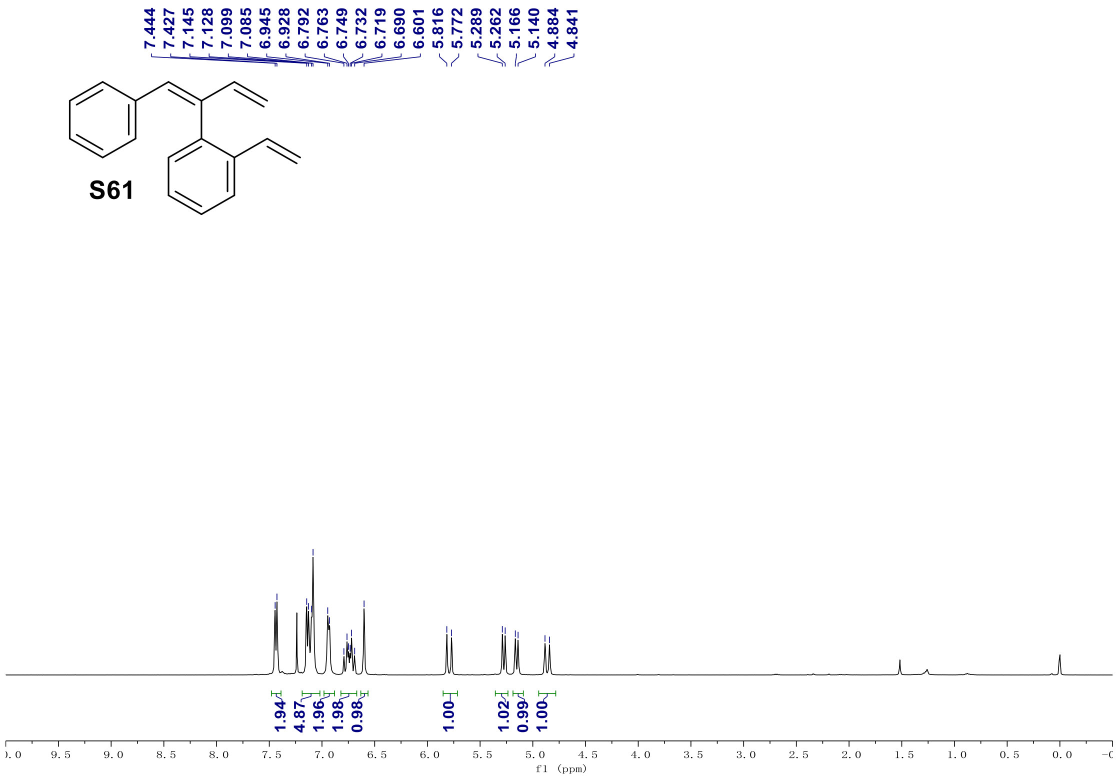

Supplementary Figure 87. ^{1}H NMR spectra of compound **S61**

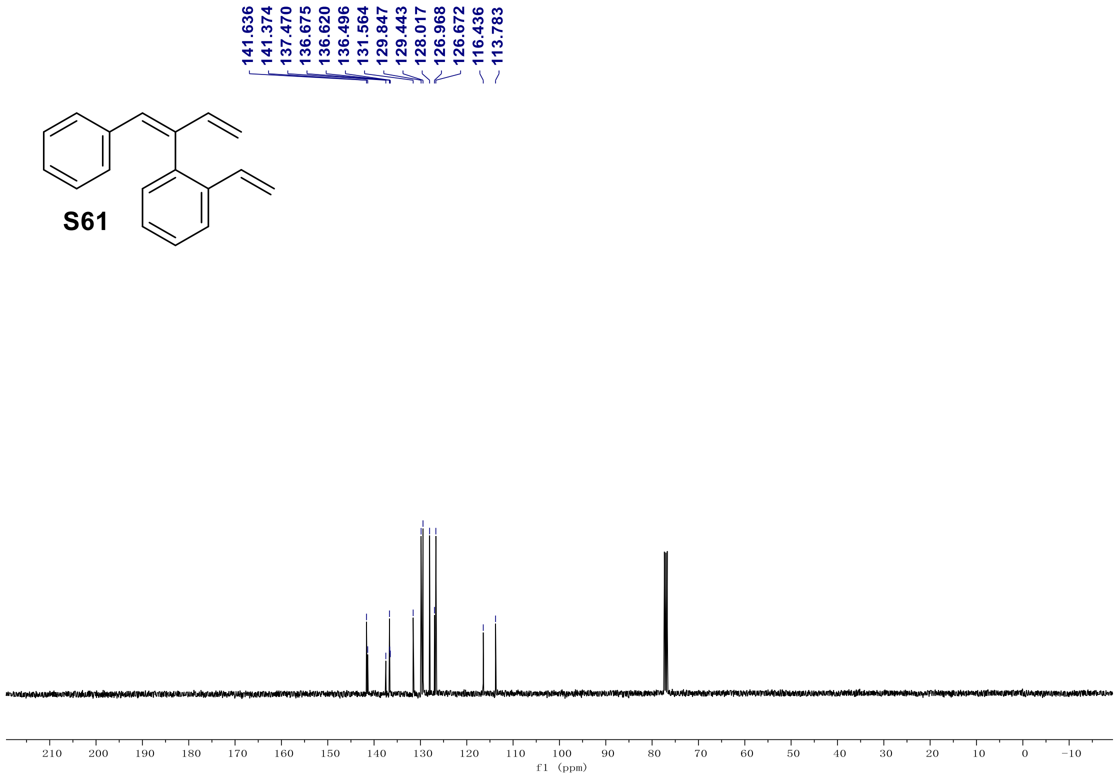

Supplementary Figure 88. ^{13}C NMR spectra of compound **S61**

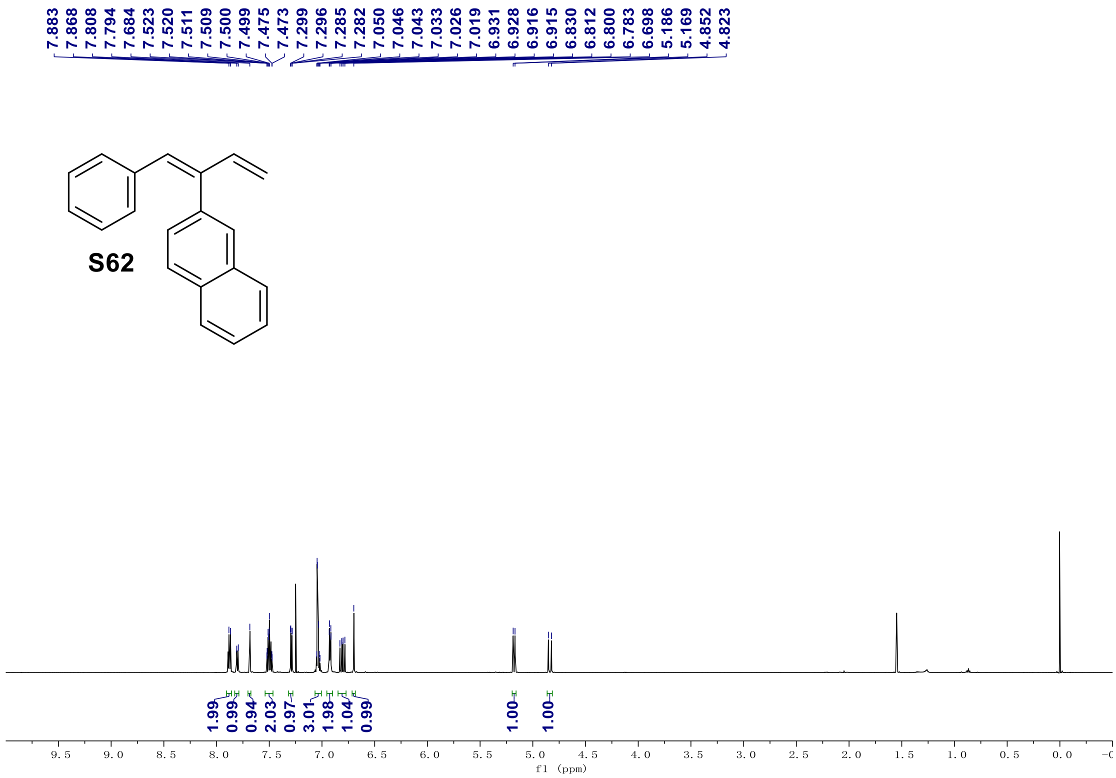

Supplementary Figure 89. ^{1}H NMR spectra of compound **S62**

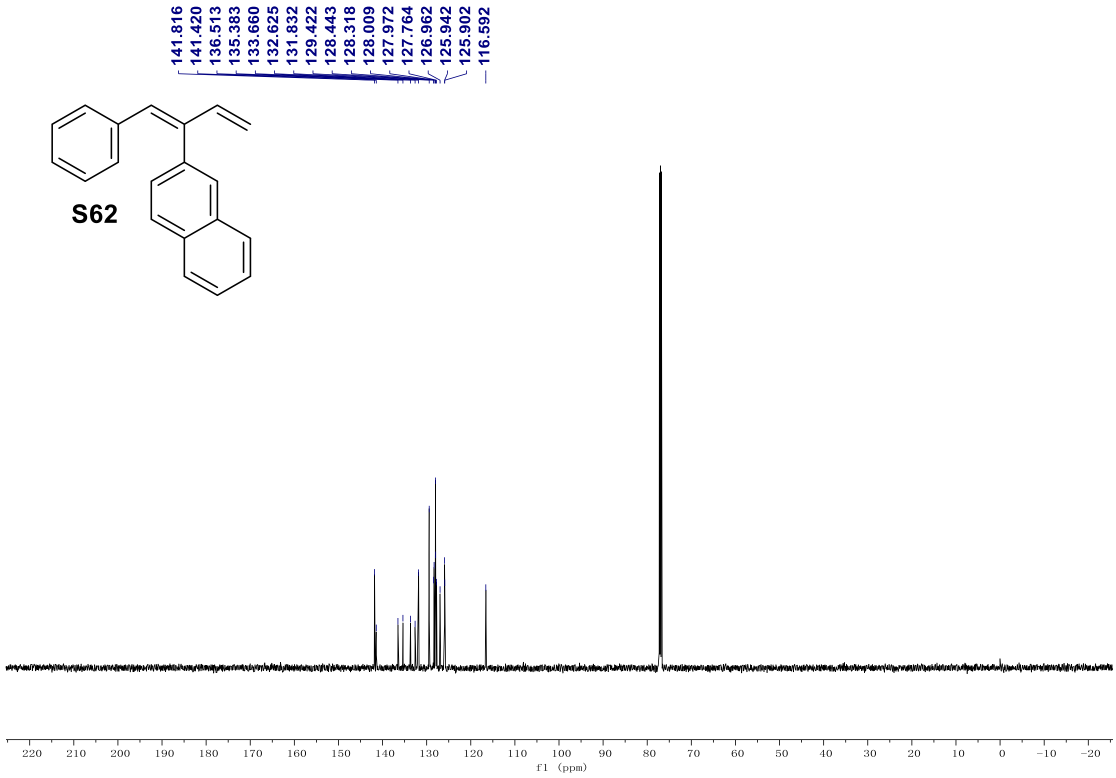

Supplementary Figure 90. ^{13}C NMR spectra of compound **S62**

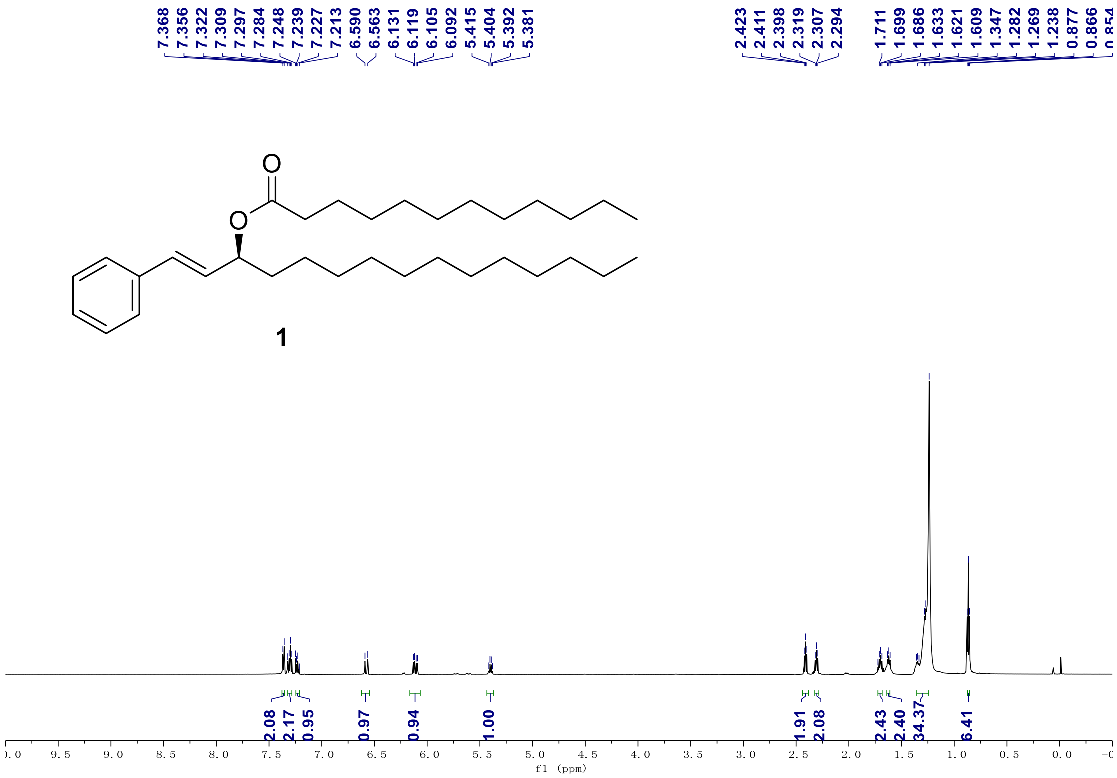

Supplementary Figure 91. ^{1}H NMR spectra of compound **1**

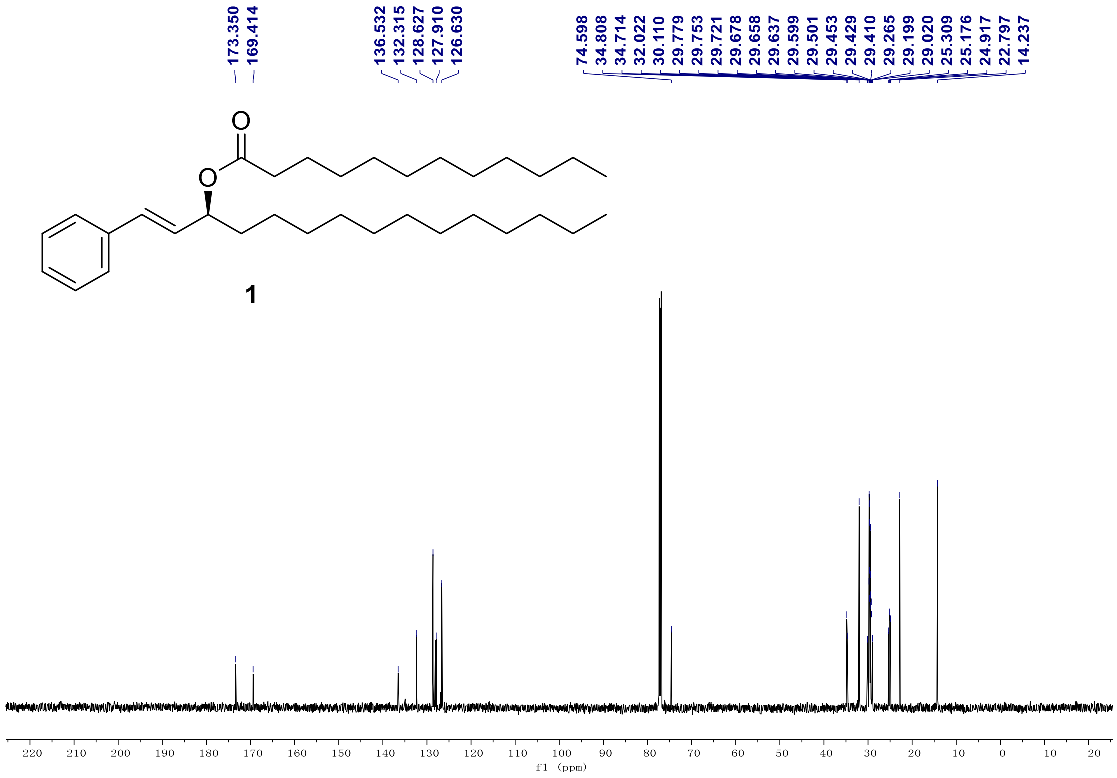

Supplementary Figure 92. ^{13}C NMR spectra of compound **1**

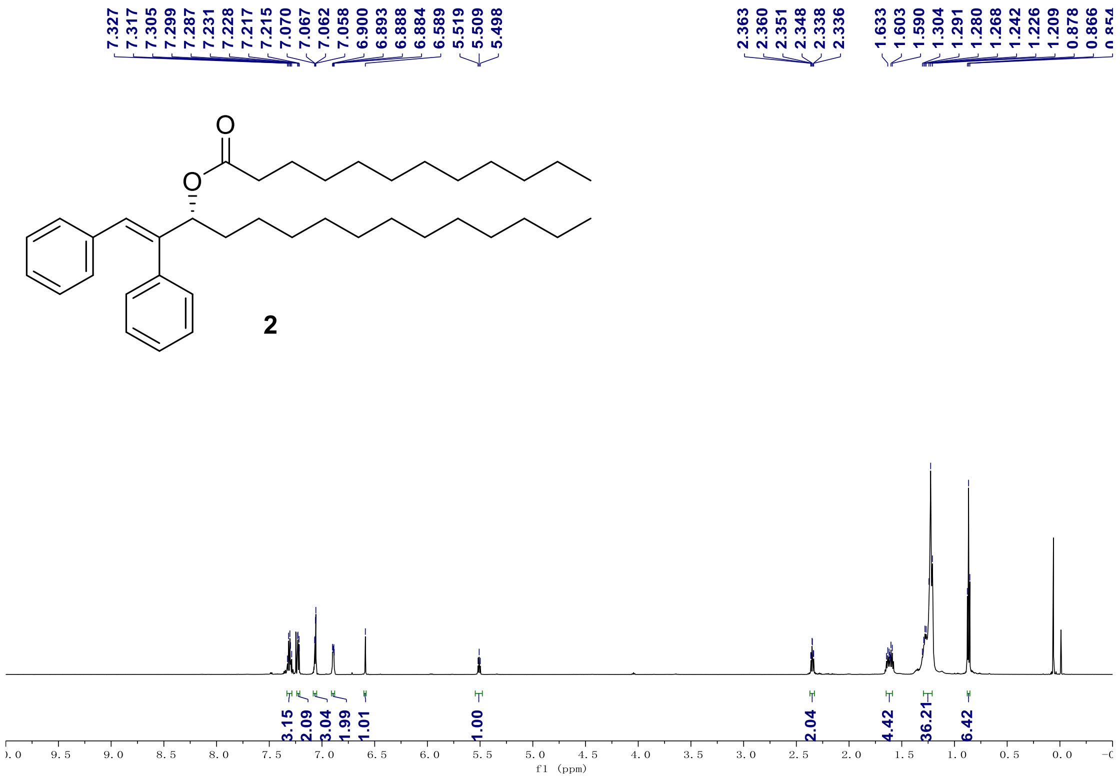

Supplementary Figure 93. ^{1}H NMR spectra of compound **2**

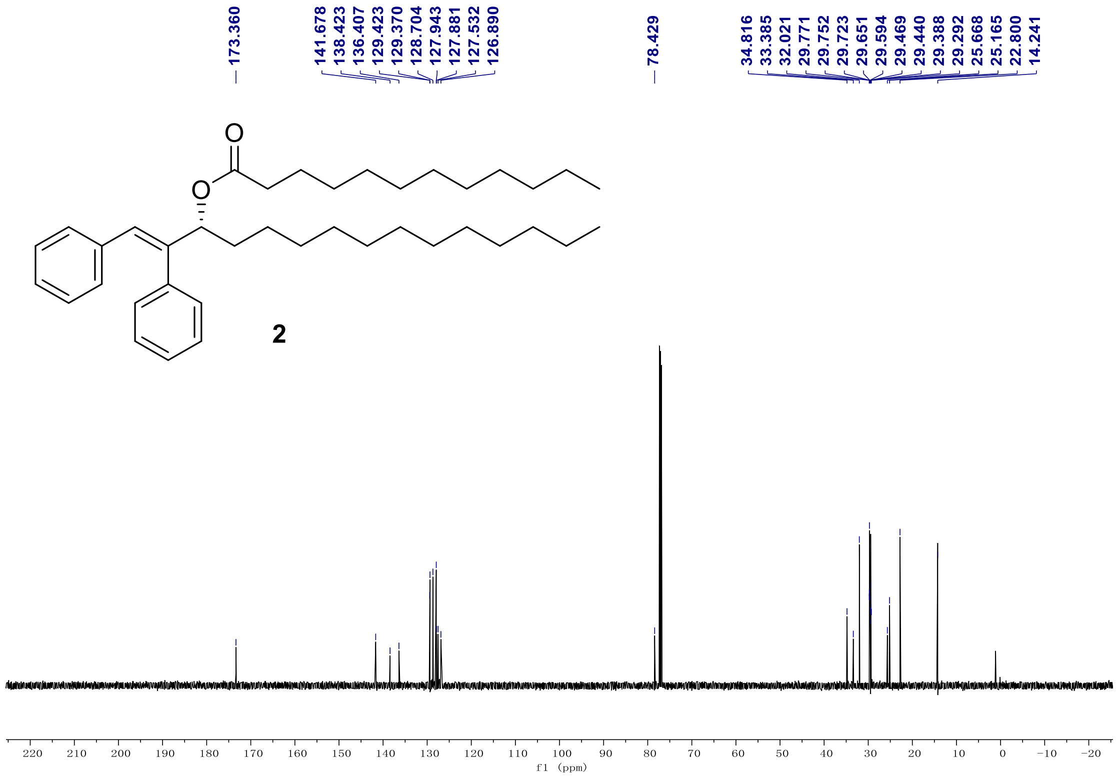

Supplementary Figure 94. ^{13}C NMR spectra of compound **2**

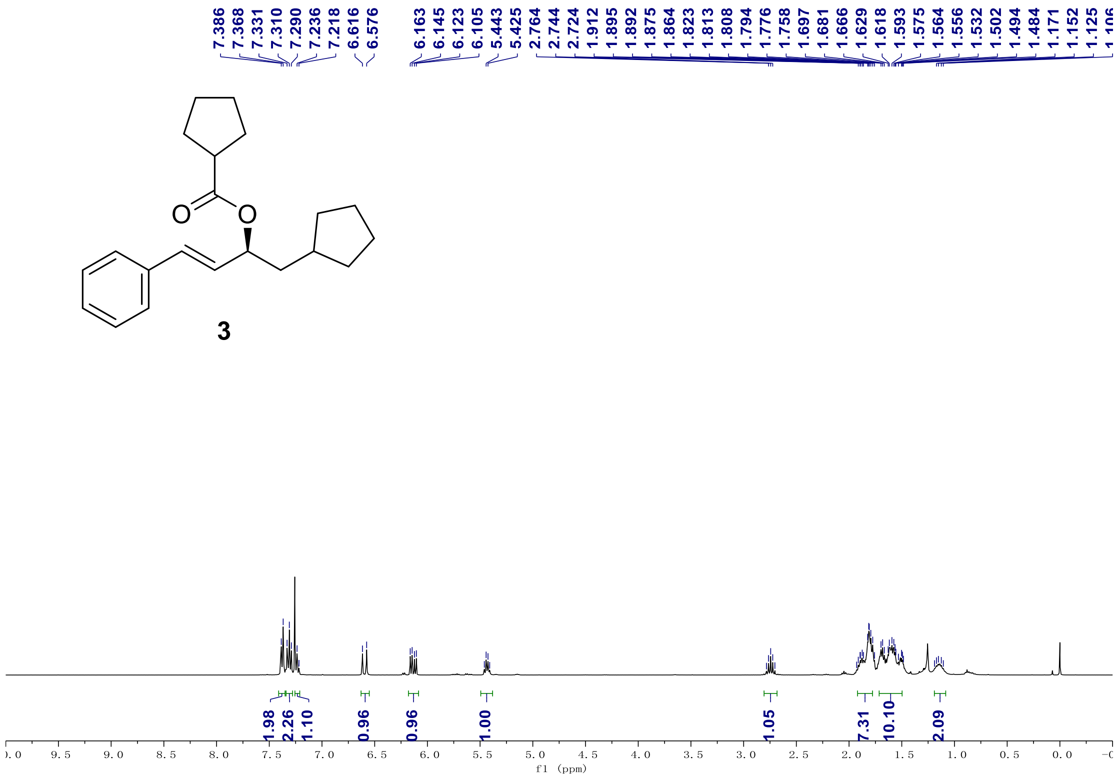

Supplementary Figure 95. ^{1}H NMR spectra of compound 3

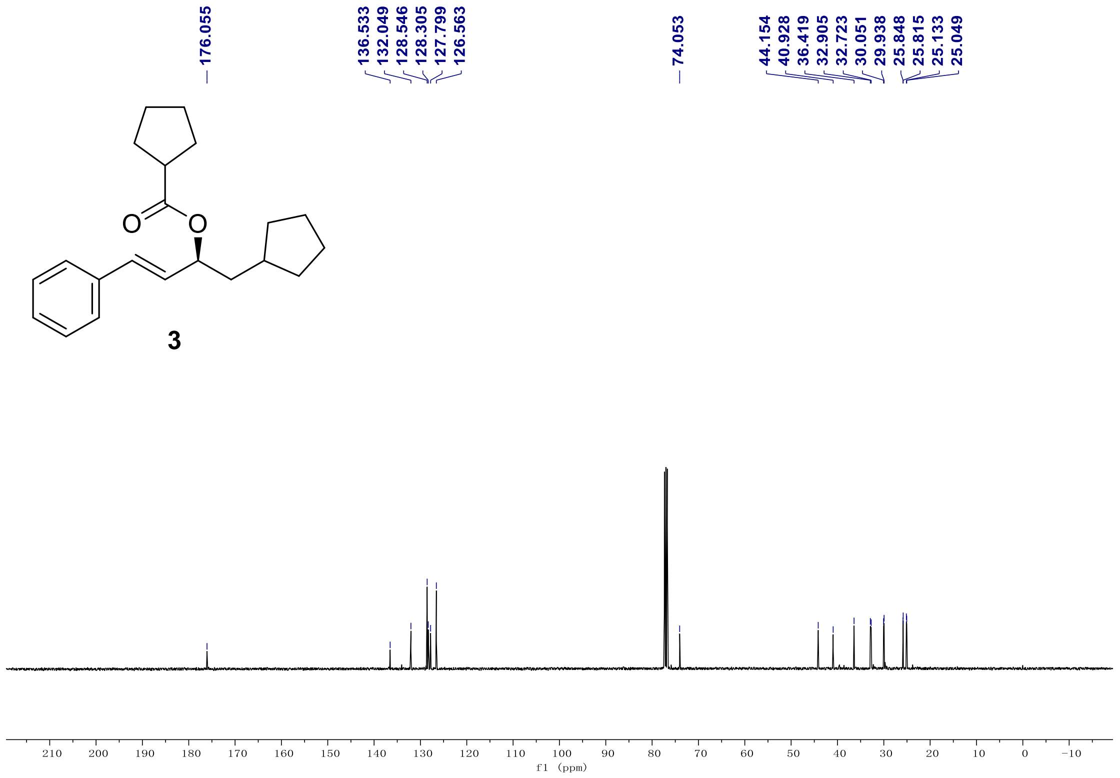

Supplementary Figure 96. ^{13}C NMR spectra of compound 3

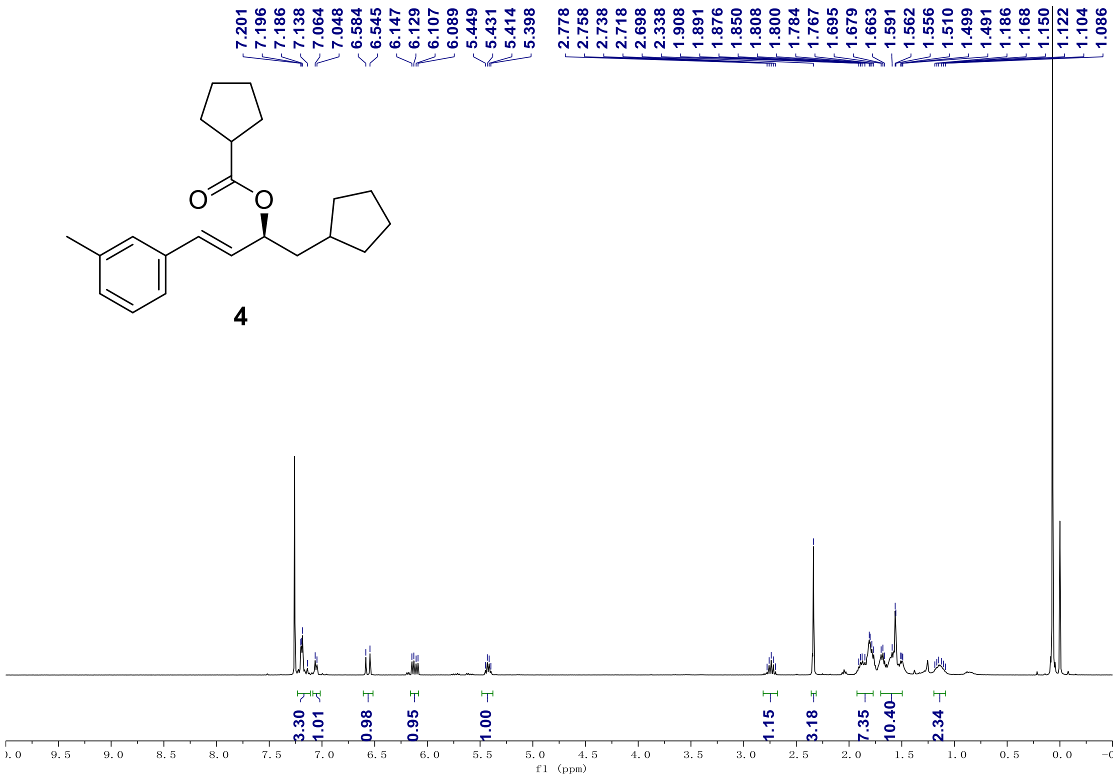

Supplementary Figure 97. ^{1}H NMR spectra of compound **4**

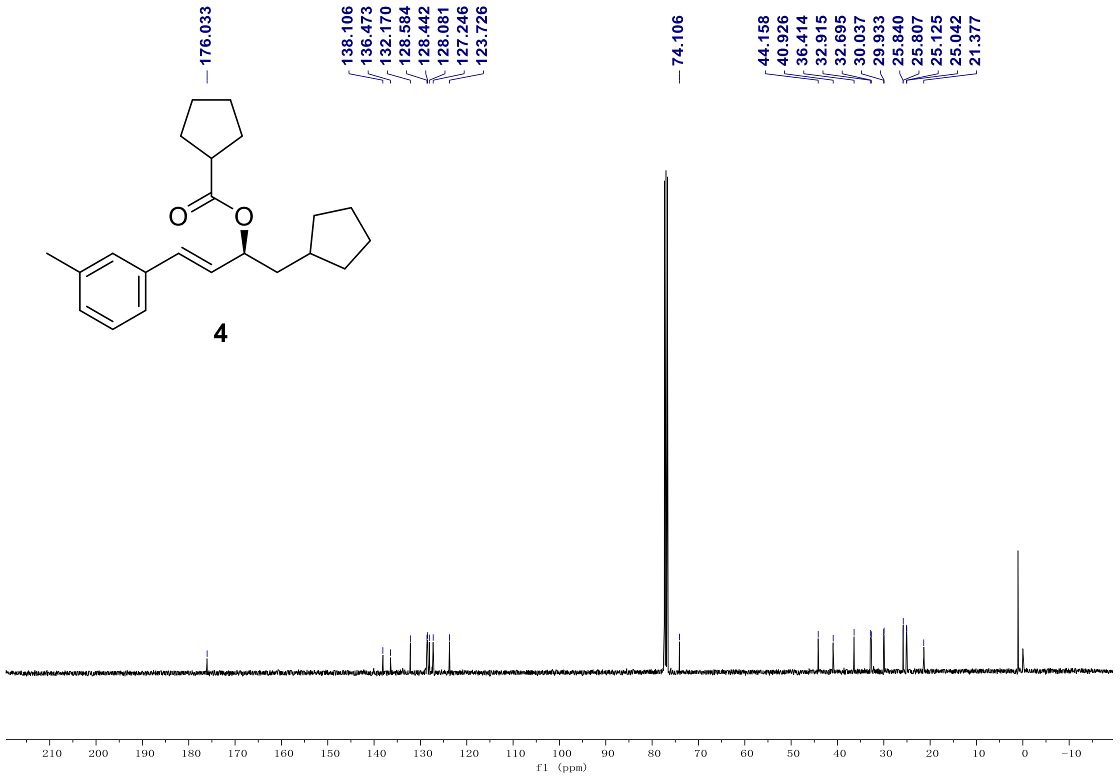

Supplementary Figure 98. ^{13}C NMR spectra of compound **4**

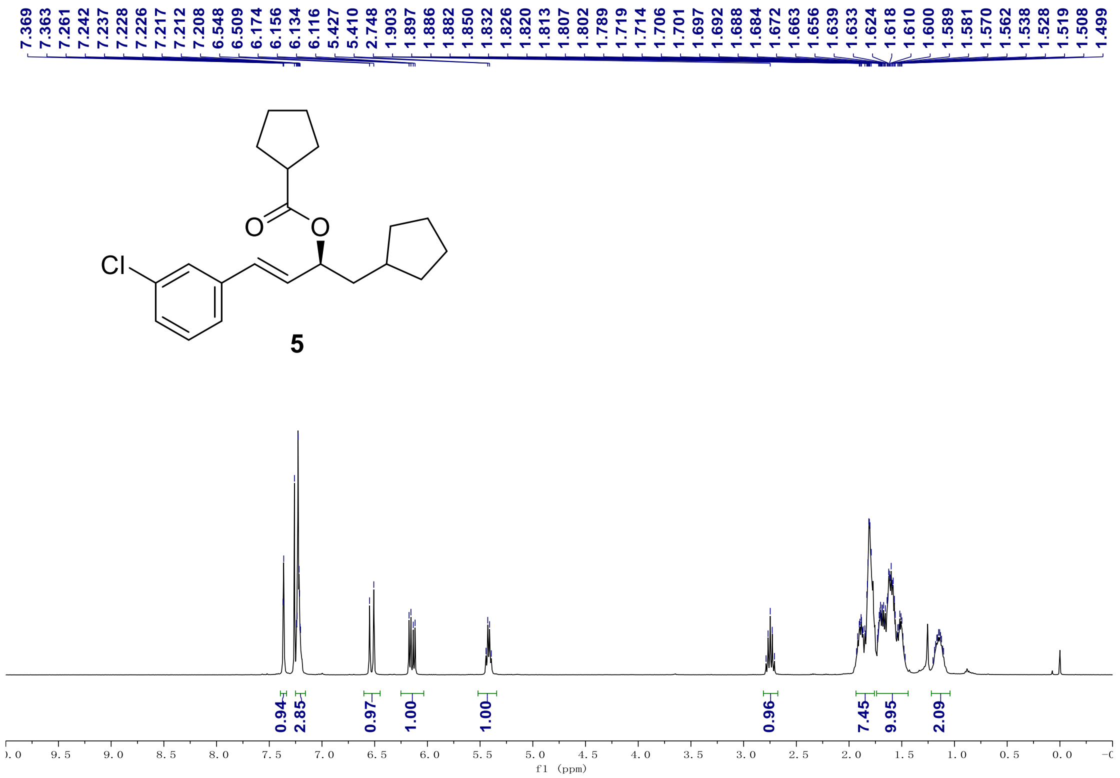

Supplementary Figure 99. ^{1}H NMR spectra of compound **5**

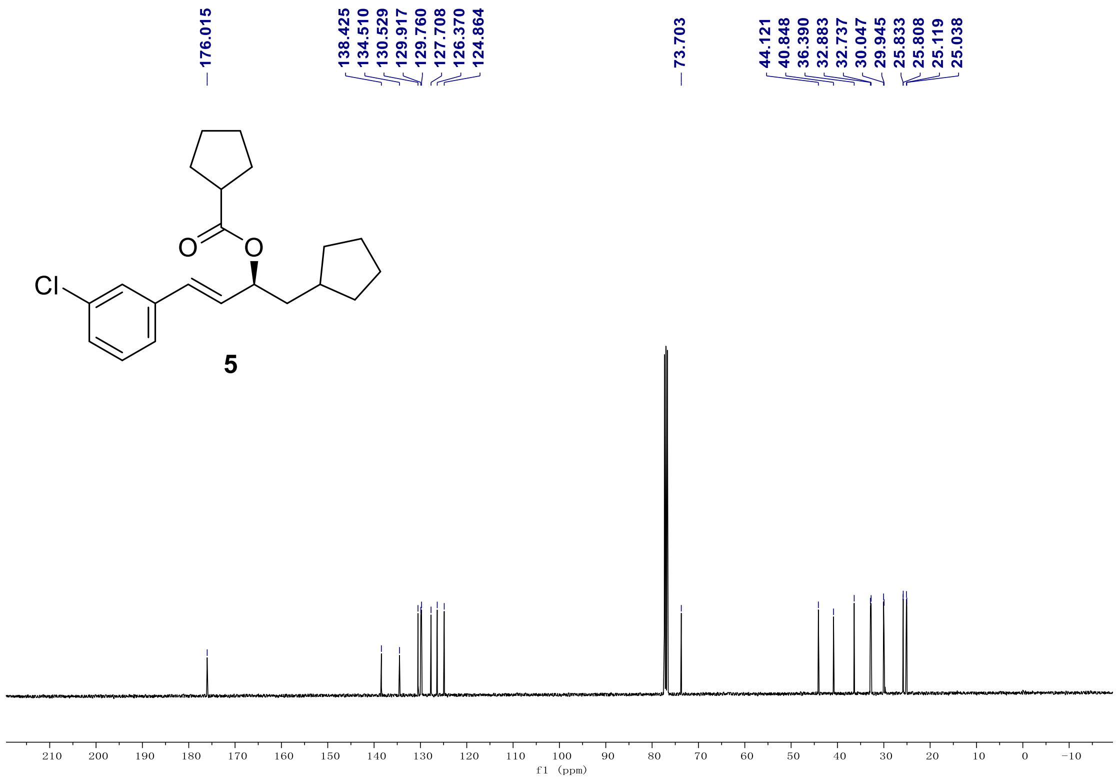

Supplementary Figure 100. ^{13}C NMR spectra of compound **5**

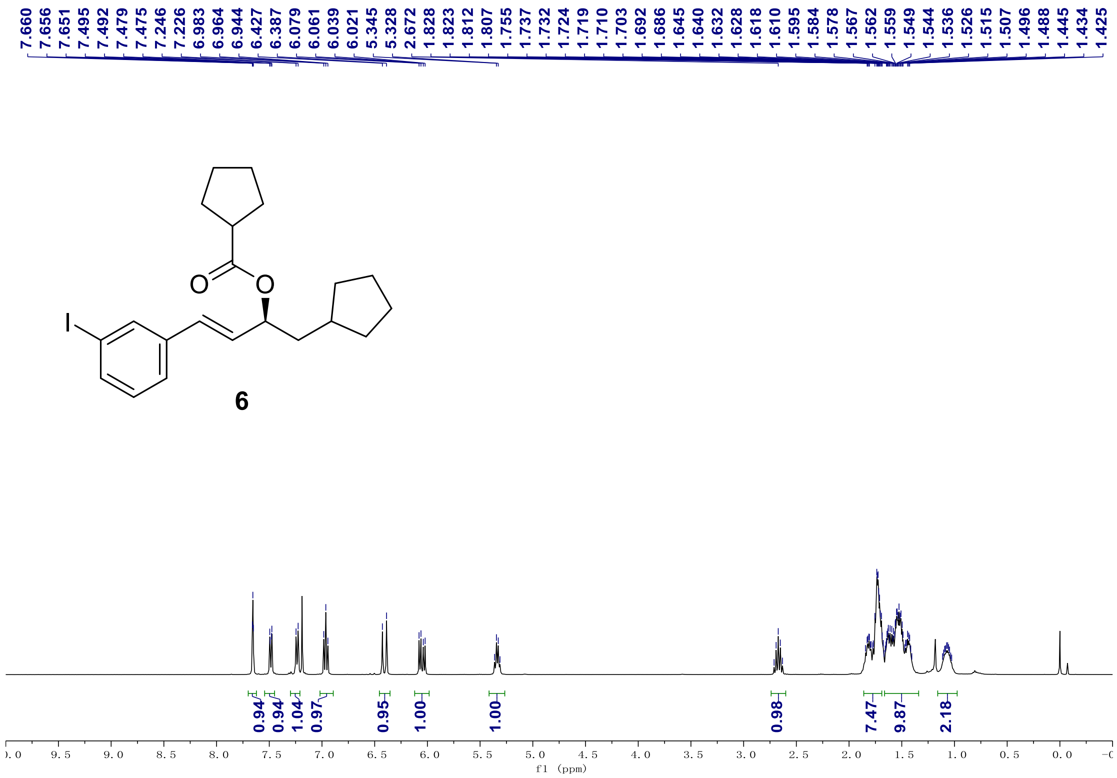

Supplementary Figure 101. ^{1}H NMR spectra of compound **6**

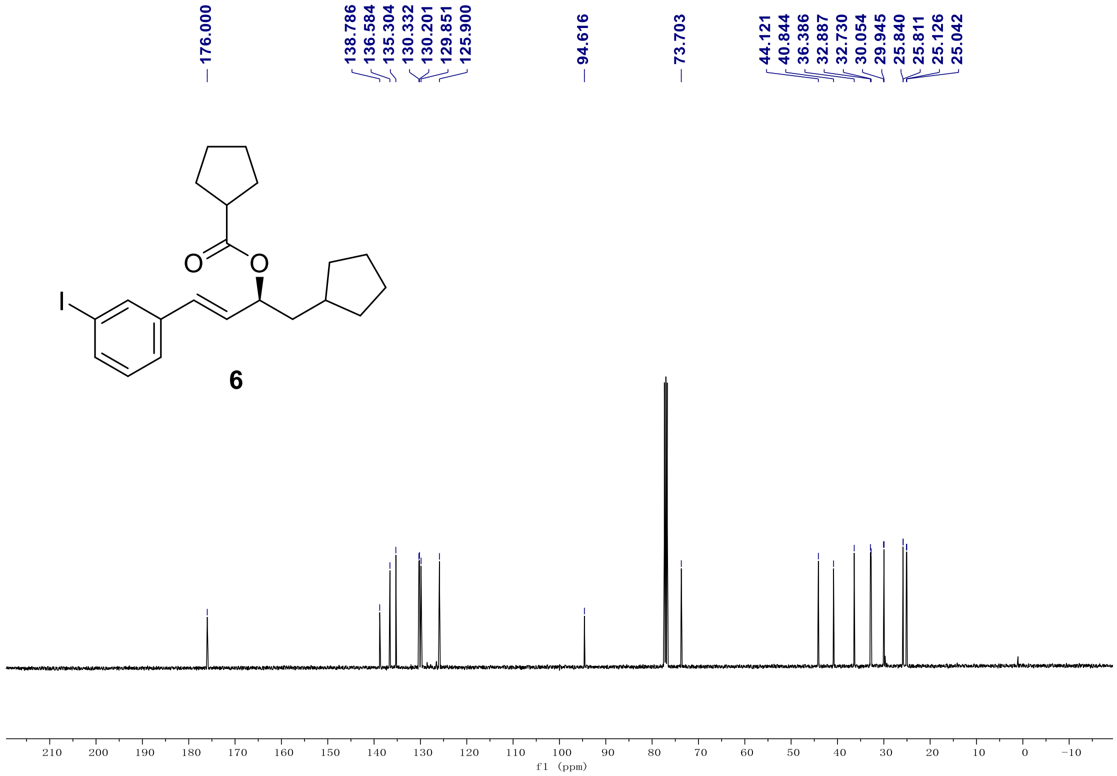

Supplementary Figure 102. ^{13}C NMR spectra of compound **6**

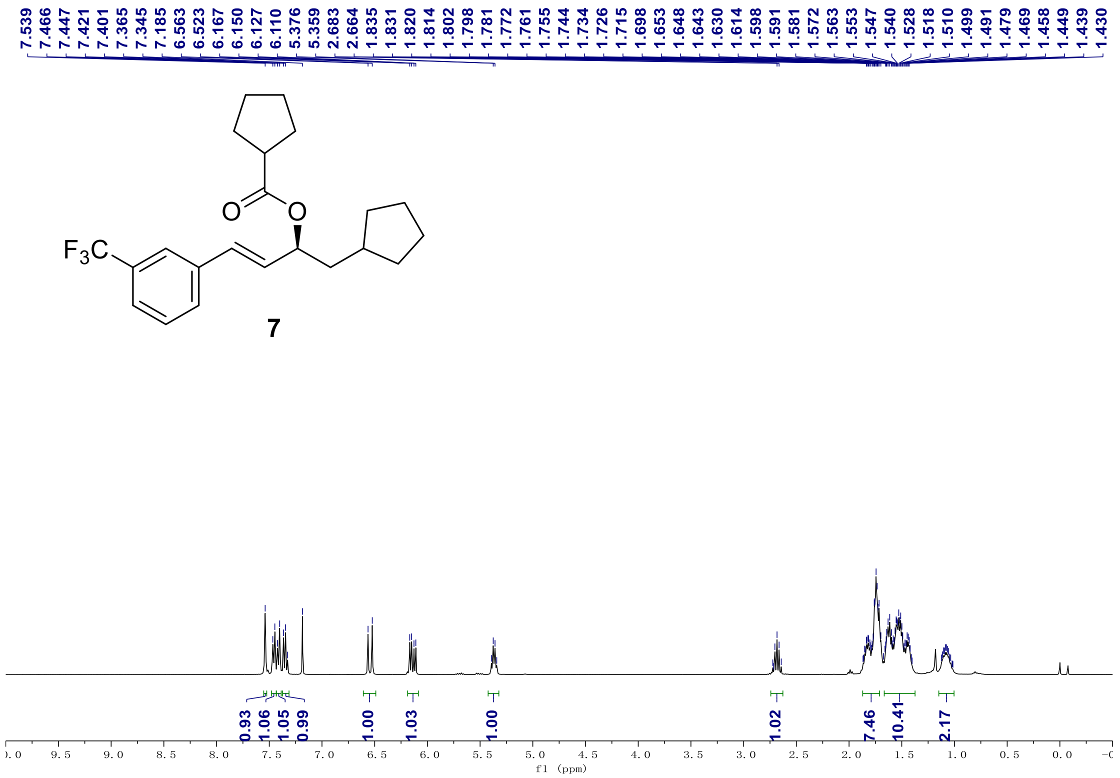

Supplementary Figure 103. ^{1}H NMR spectra of compound **7**

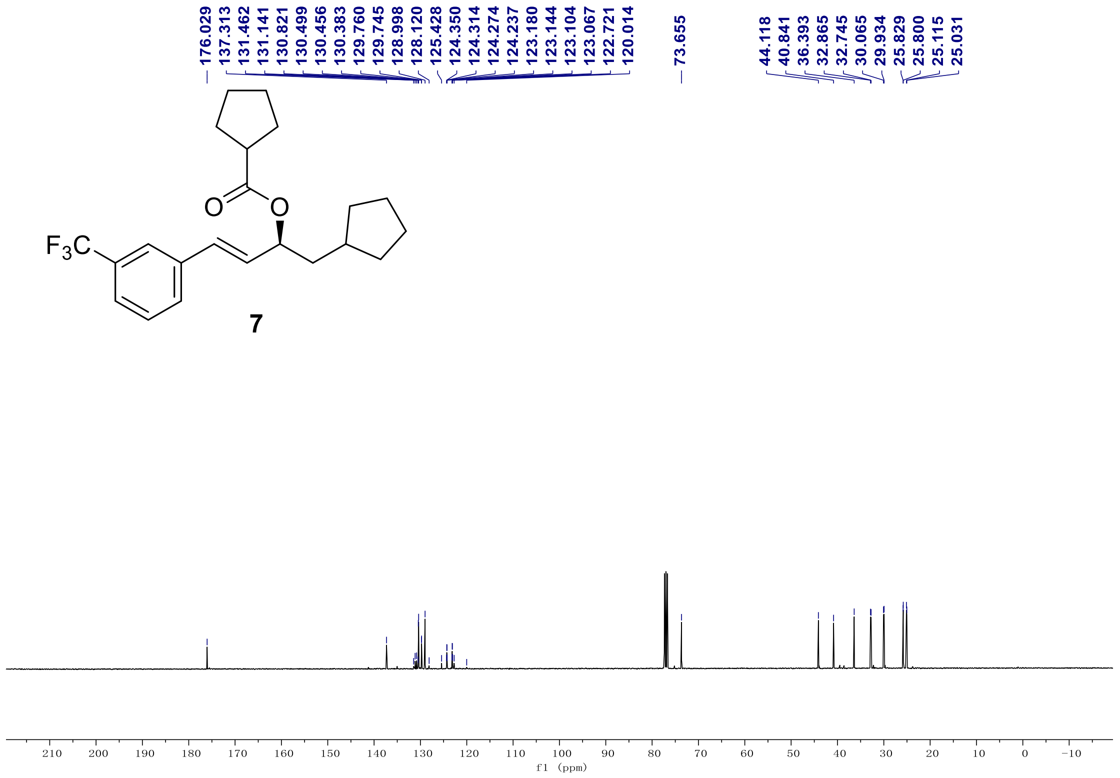

Supplementary Figure 104. ^{13}C NMR spectra of compound **7**

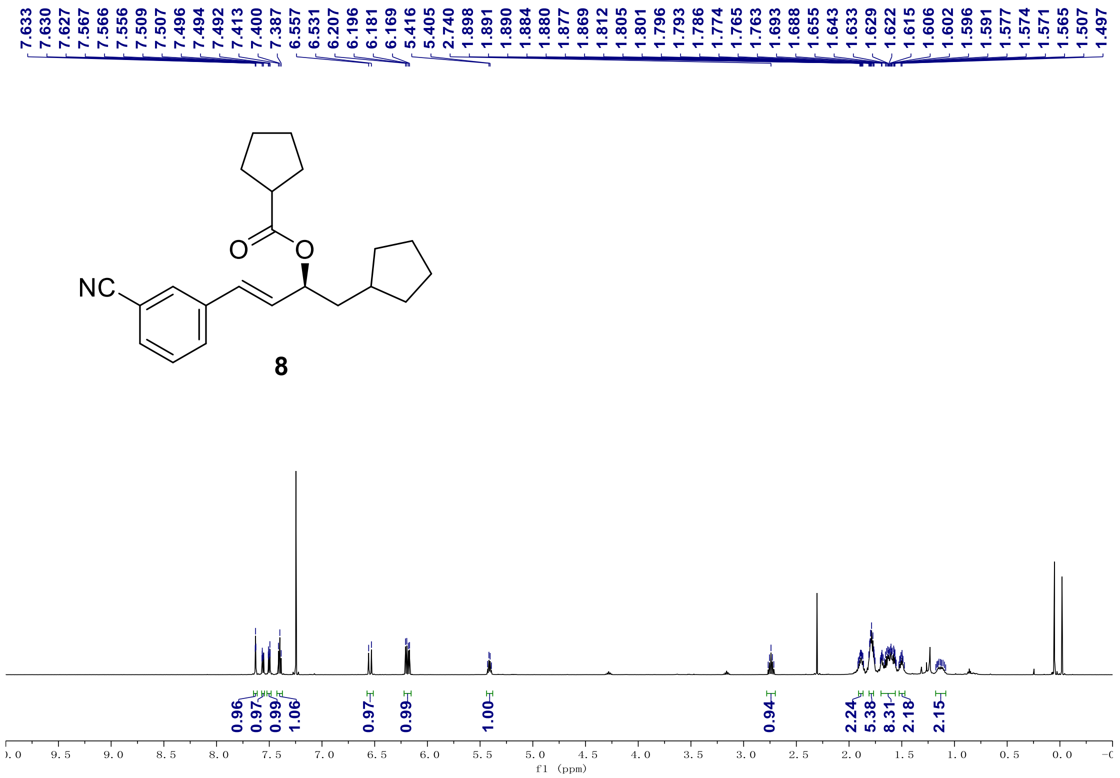

Supplementary Figure 105. ^{1}H NMR spectra of compound **8**

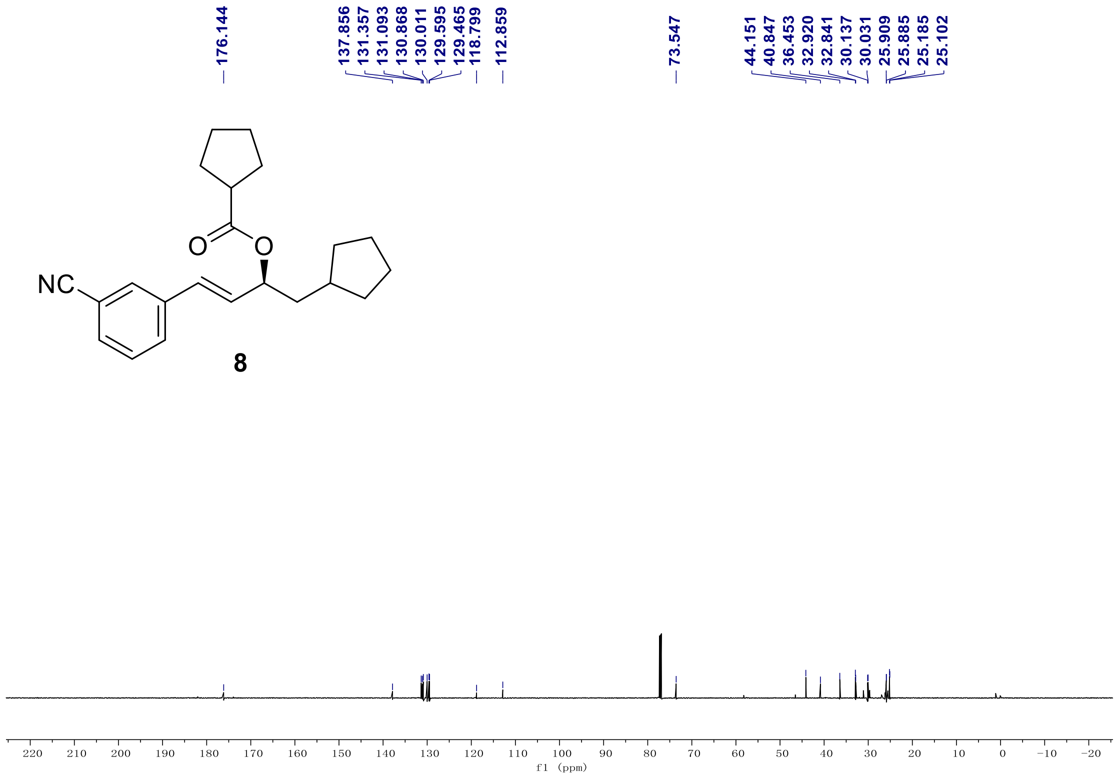

Supplementary Figure 106. ^{13}C NMR spectra of compound **8**

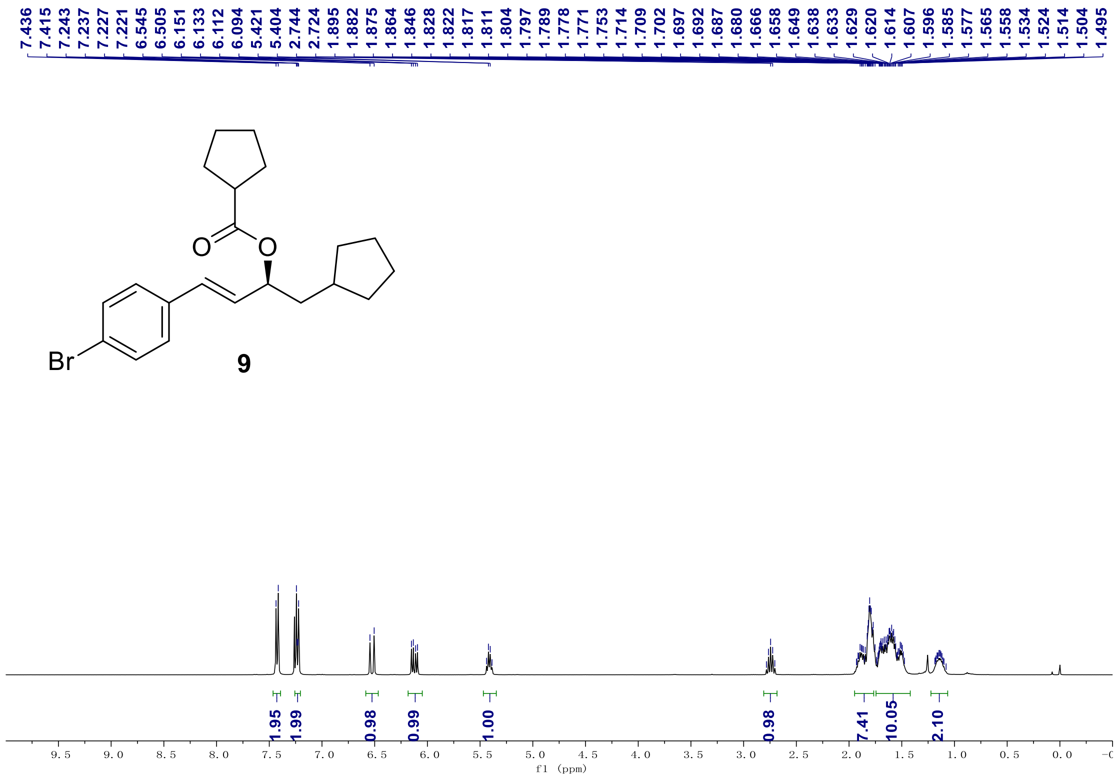

Supplementary Figure 107. ^{1}H NMR spectra of compound **9**

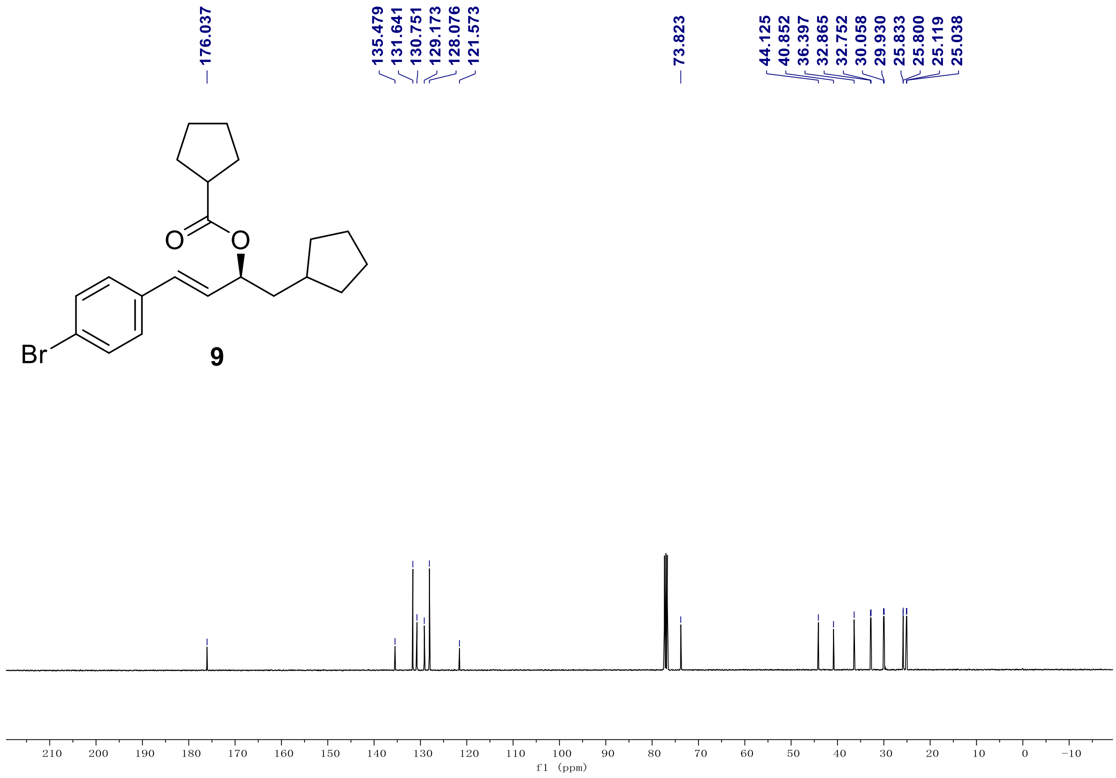

Supplementary Figure 108. ^{13}C NMR spectra of compound **9**

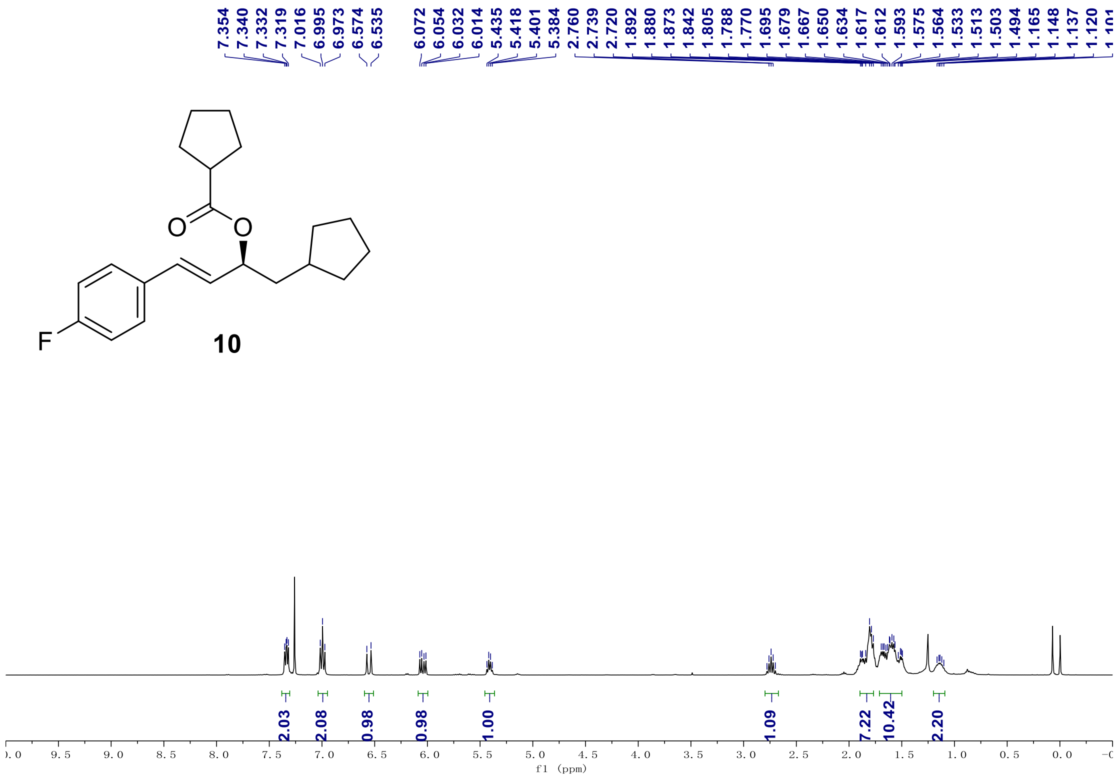

Supplementary Figure 109. ^{1}H NMR spectra of compound **10**

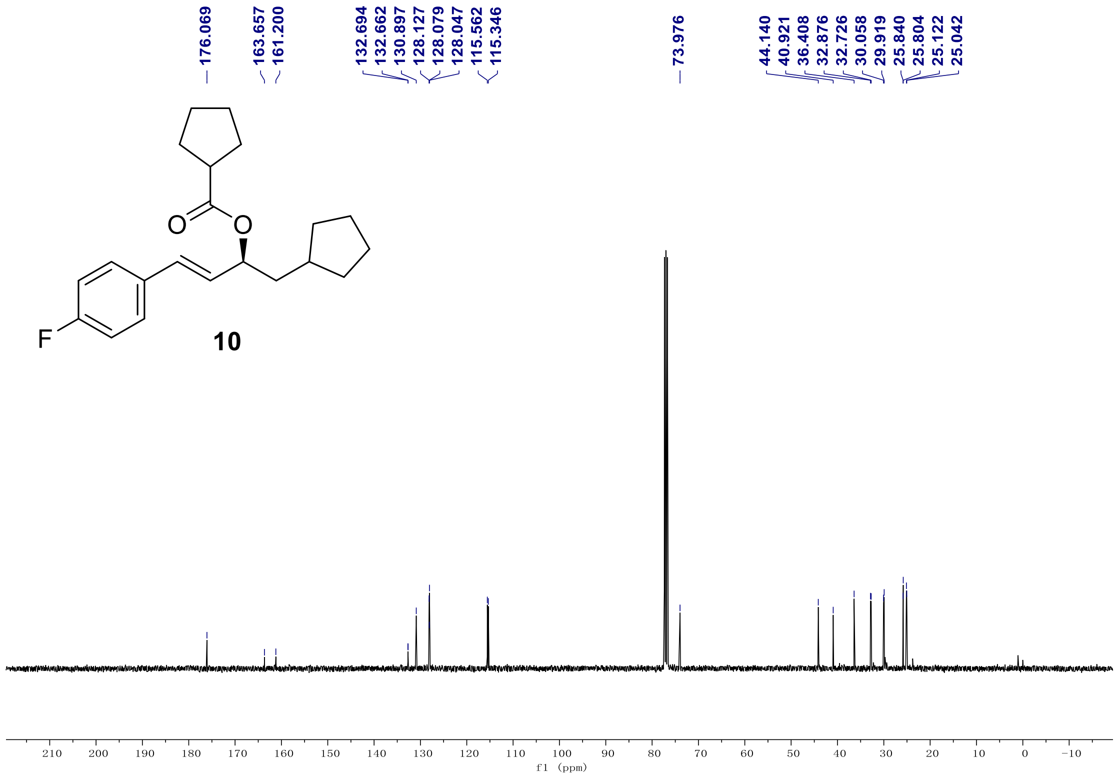

Supplementary Figure 110. ^{13}C NMR spectra of compound **10**

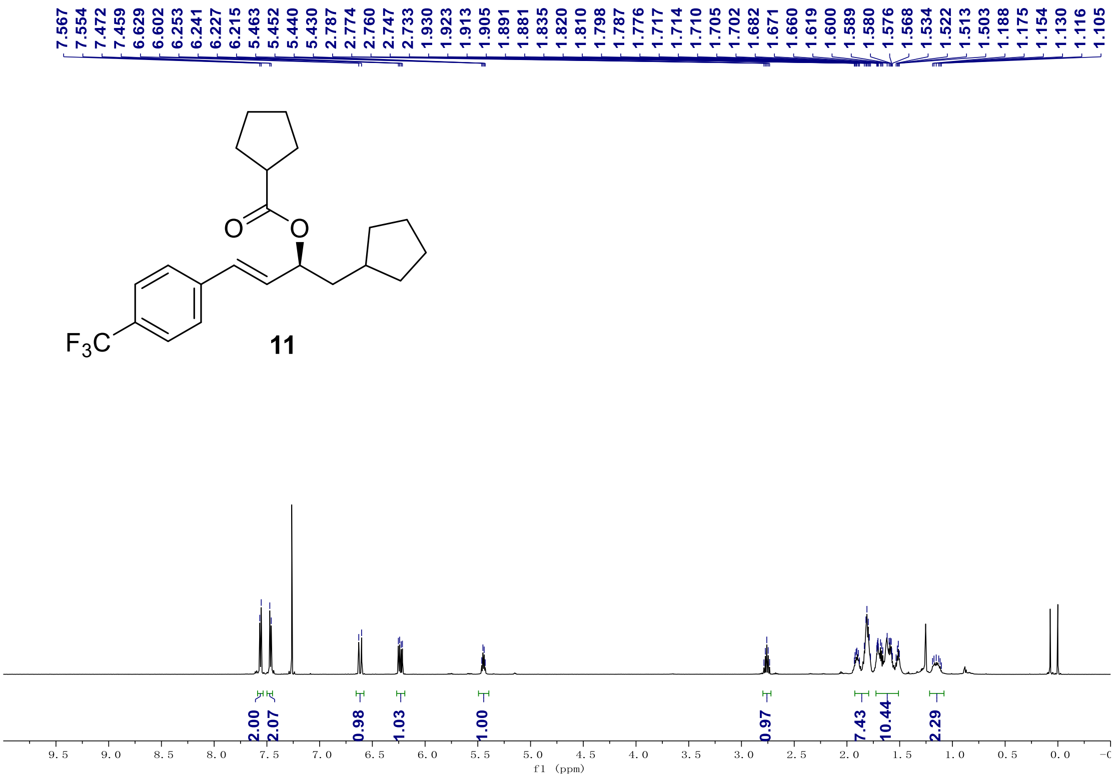

Supplementary Figure 111. ^{1}H NMR spectra of compound **11**

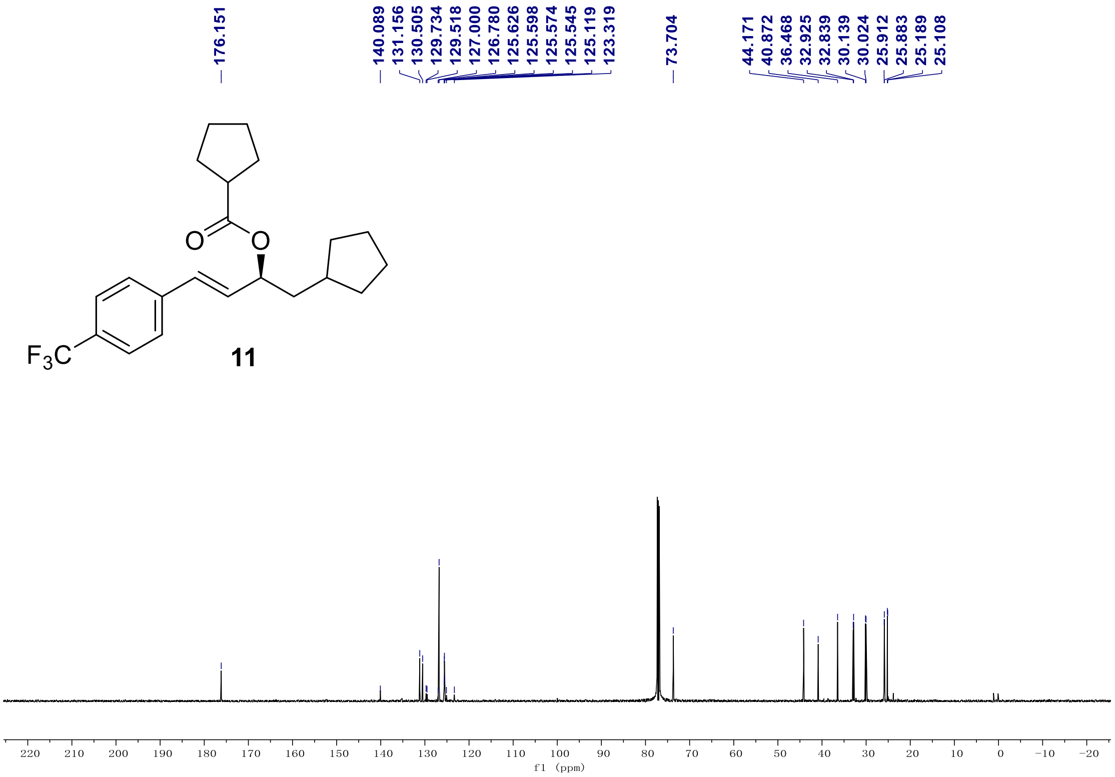

Supplementary Figure 112. ^{13}C NMR spectra of compound **11**

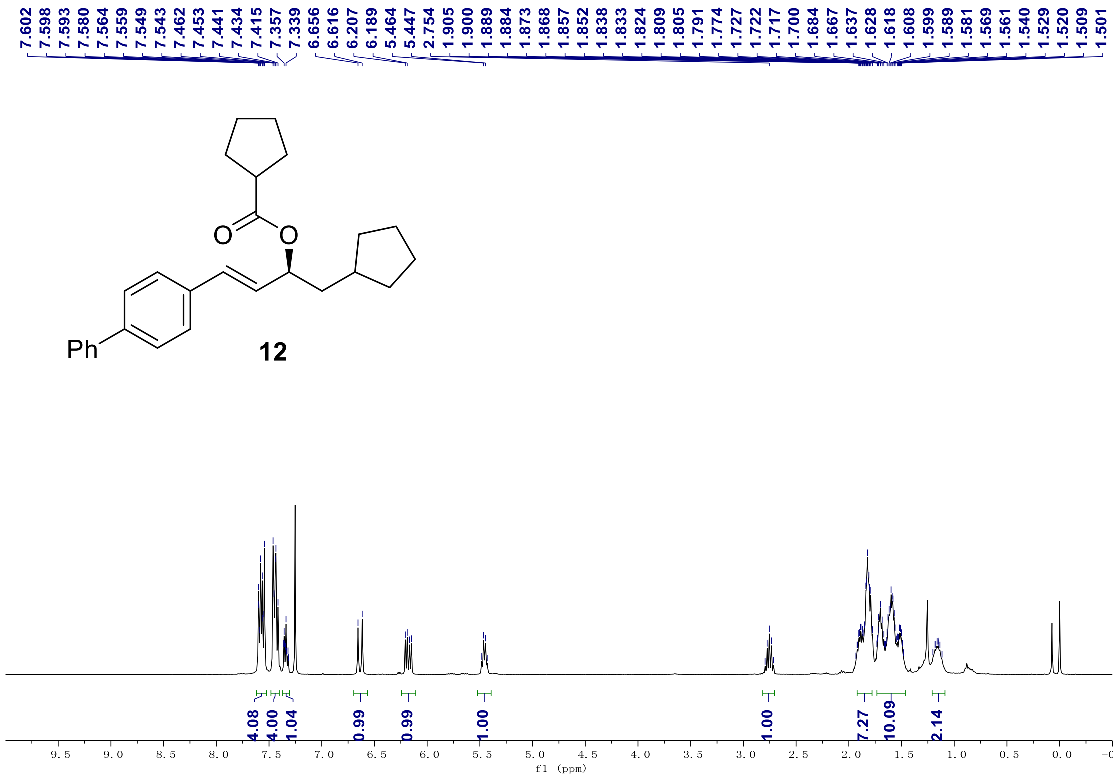

Supplementary Figure 113. ^{1}H NMR spectra of compound **12**

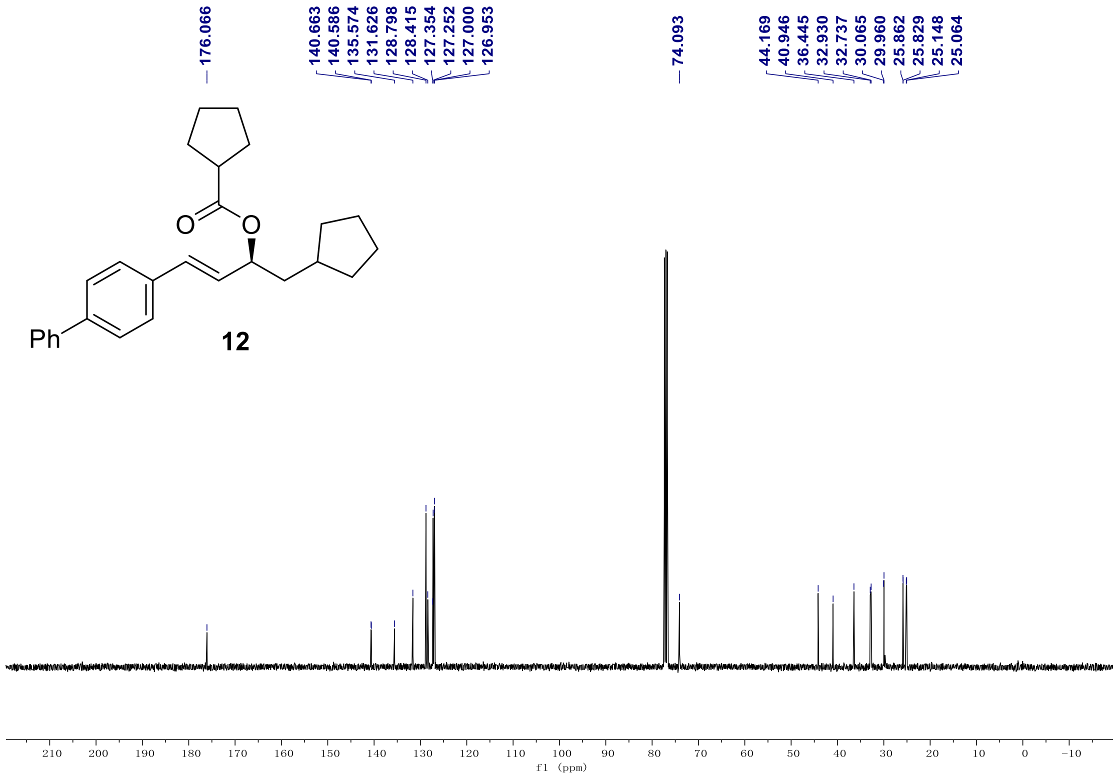

Supplementary Figure 114. ^{13}C NMR spectra of compound **12**

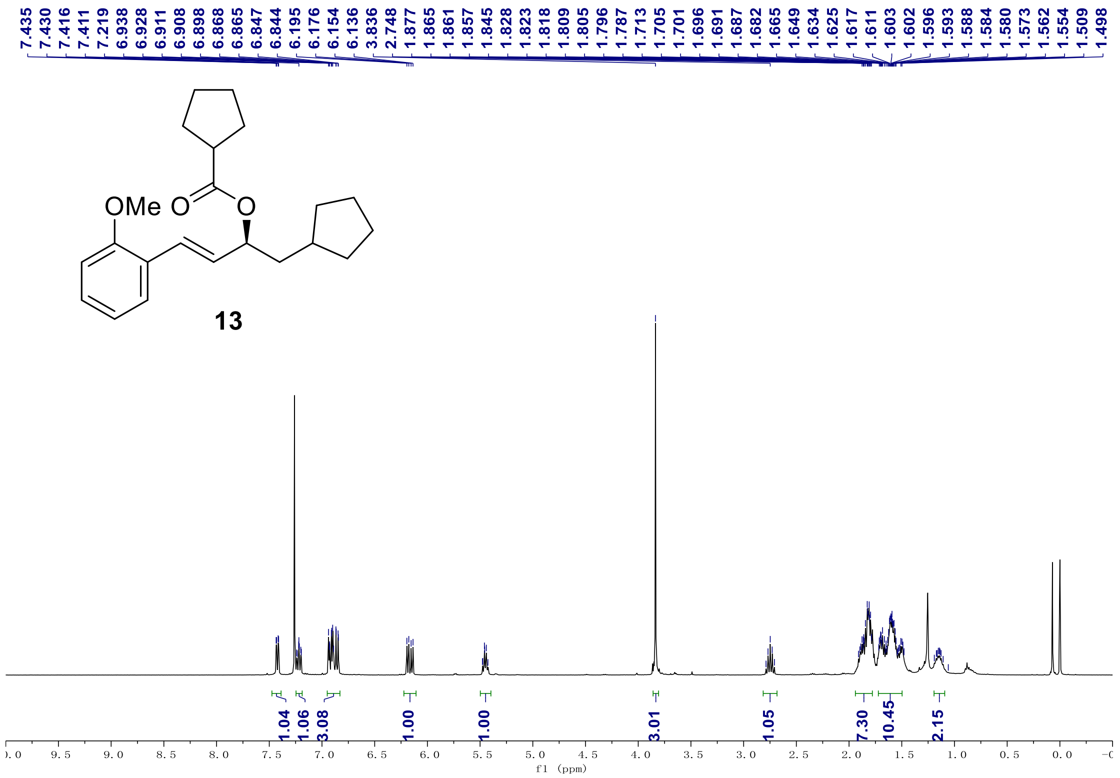

Supplementary Figure 115. ^{1}H NMR spectra of compound **13**

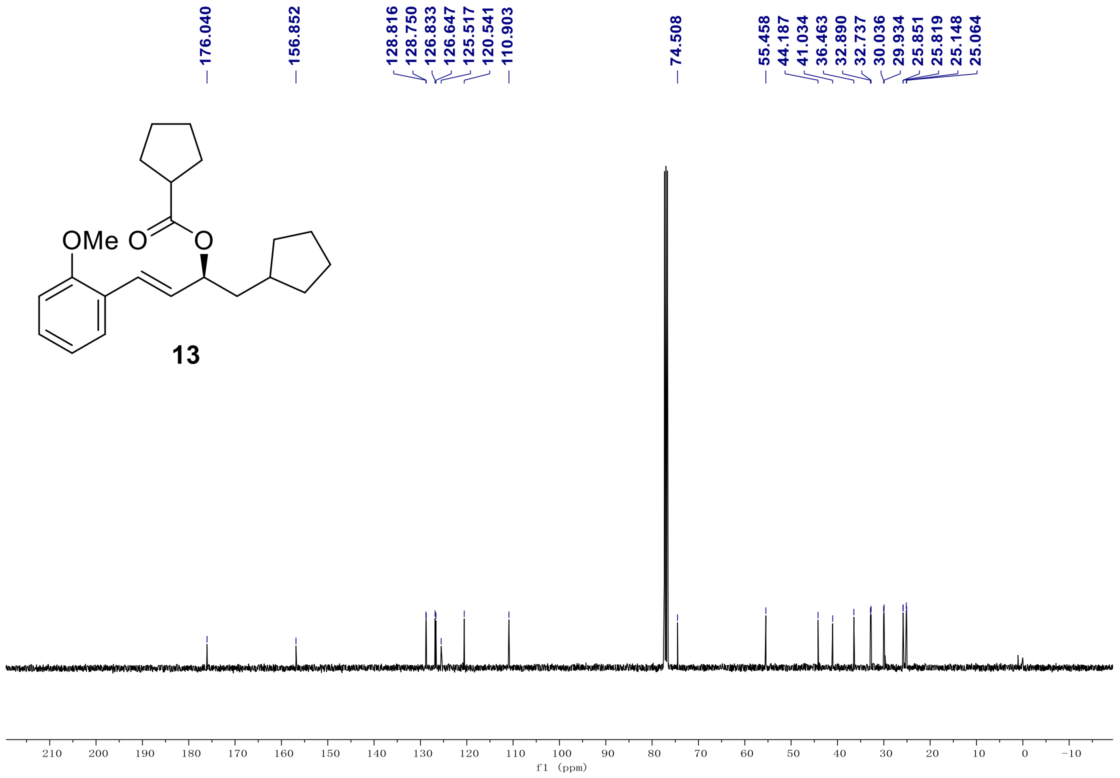

Supplementary Figure 116. ^{13}C NMR spectra of compound **13**

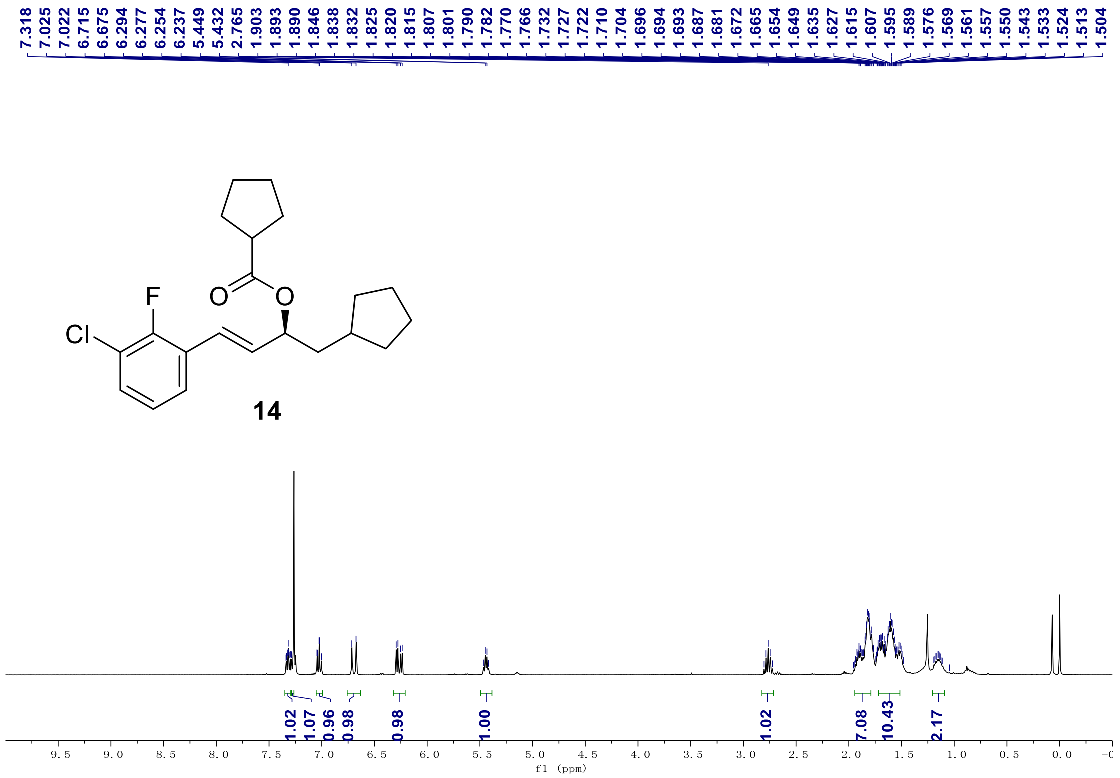

Supplementary Figure 117. ^{1}H NMR spectra of compound **14**

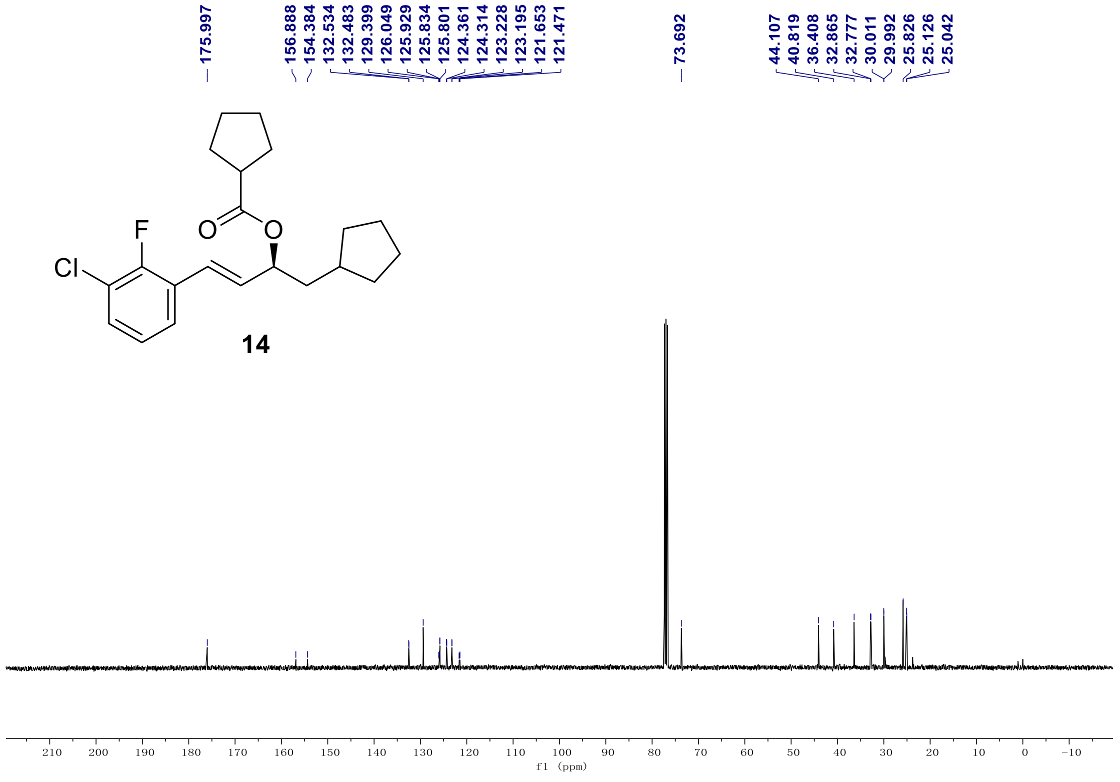

Supplementary Figure 118. ^{13}C NMR spectra of compound **14**

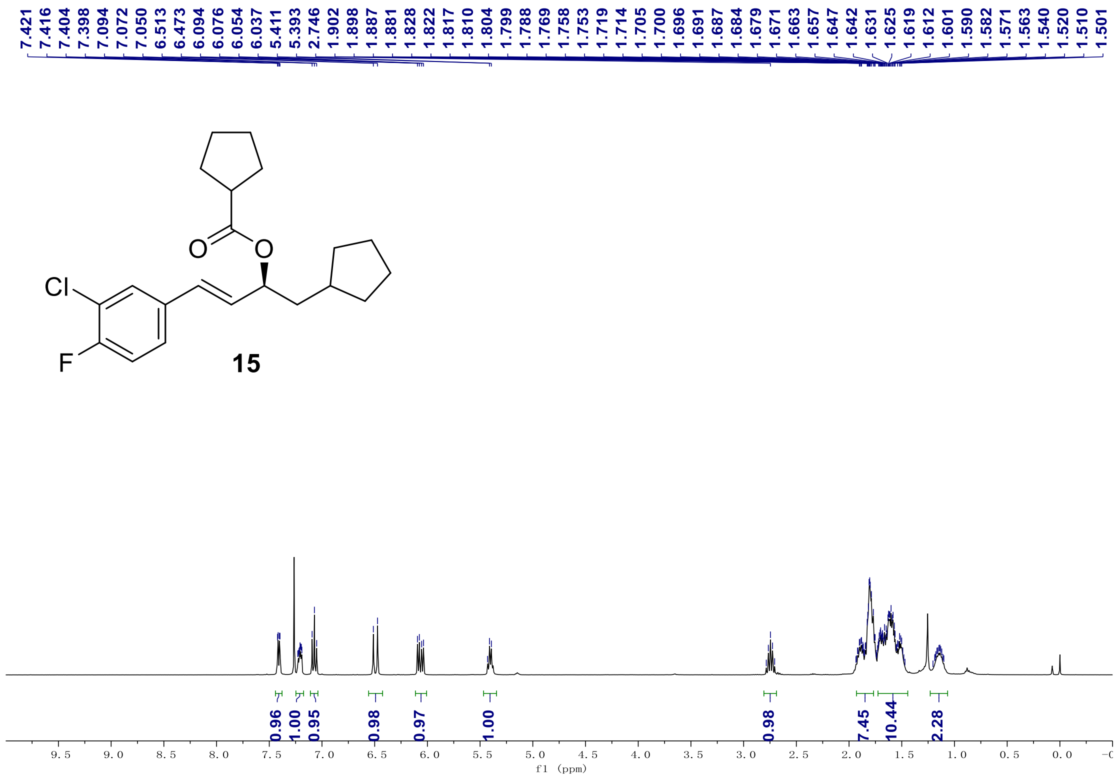

Supplementary Figure 119. ^{1}H NMR spectra of compound **15**

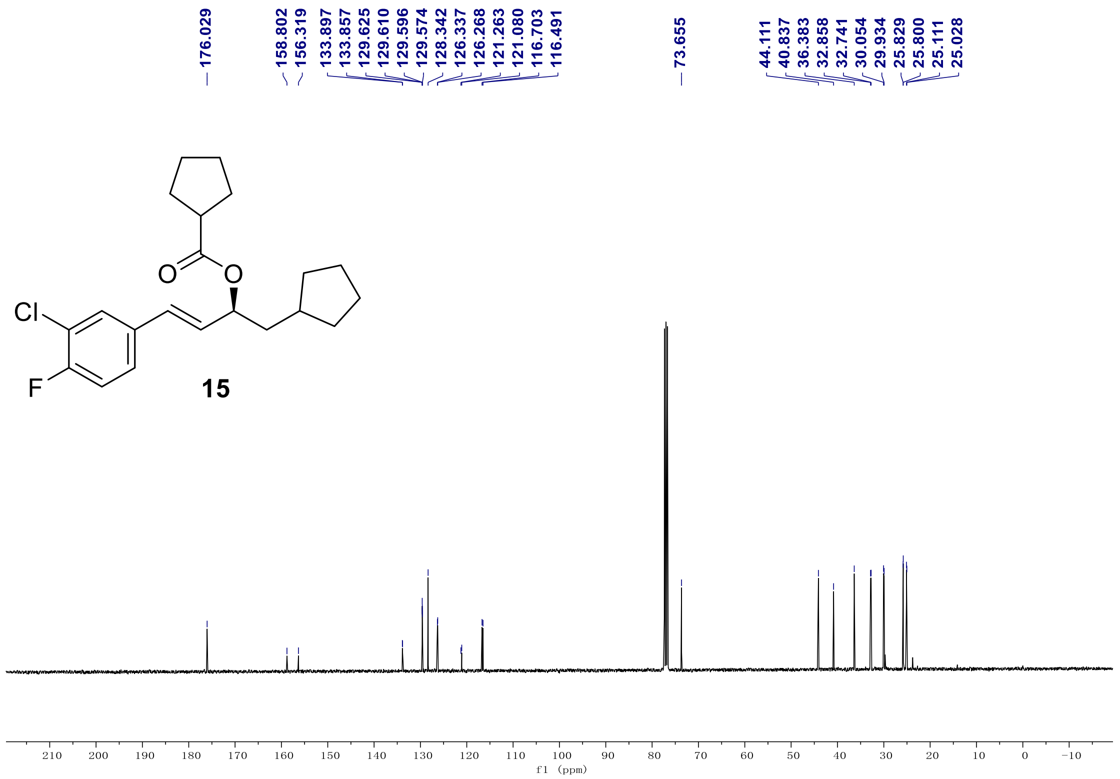

Supplementary Figure 120. ^{13}C NMR spectra of compound **15**

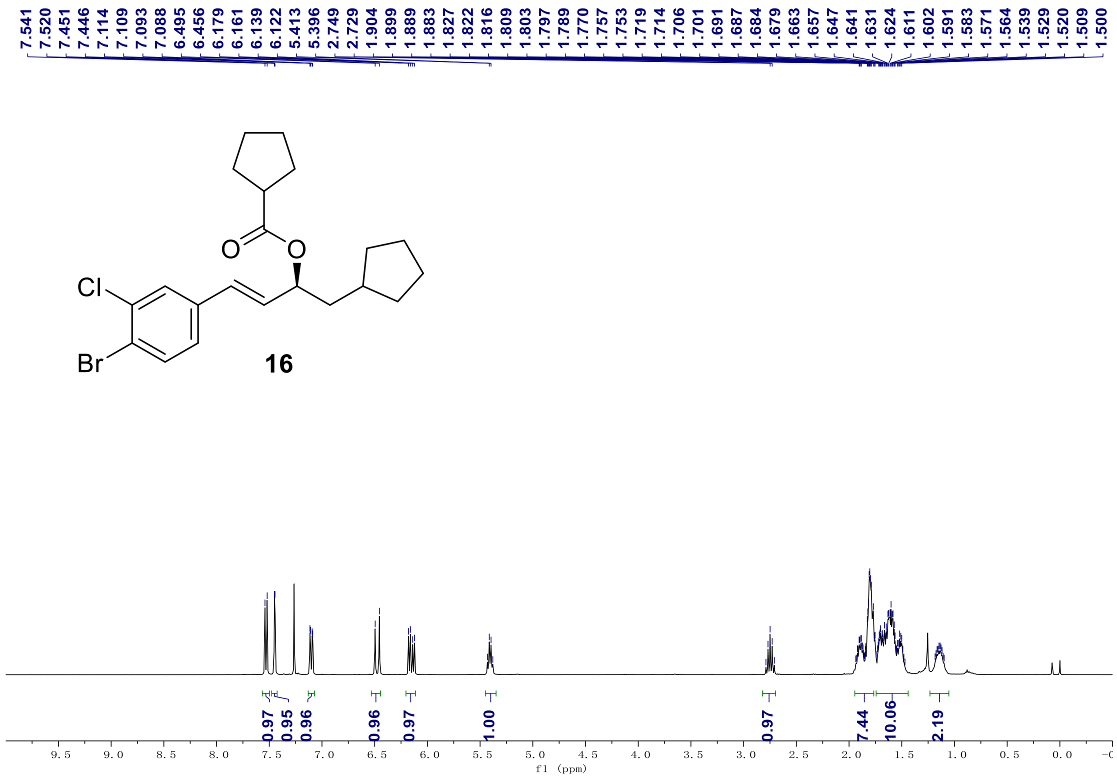

Supplementary Figure 121. ^{1}H NMR spectra of compound **16**

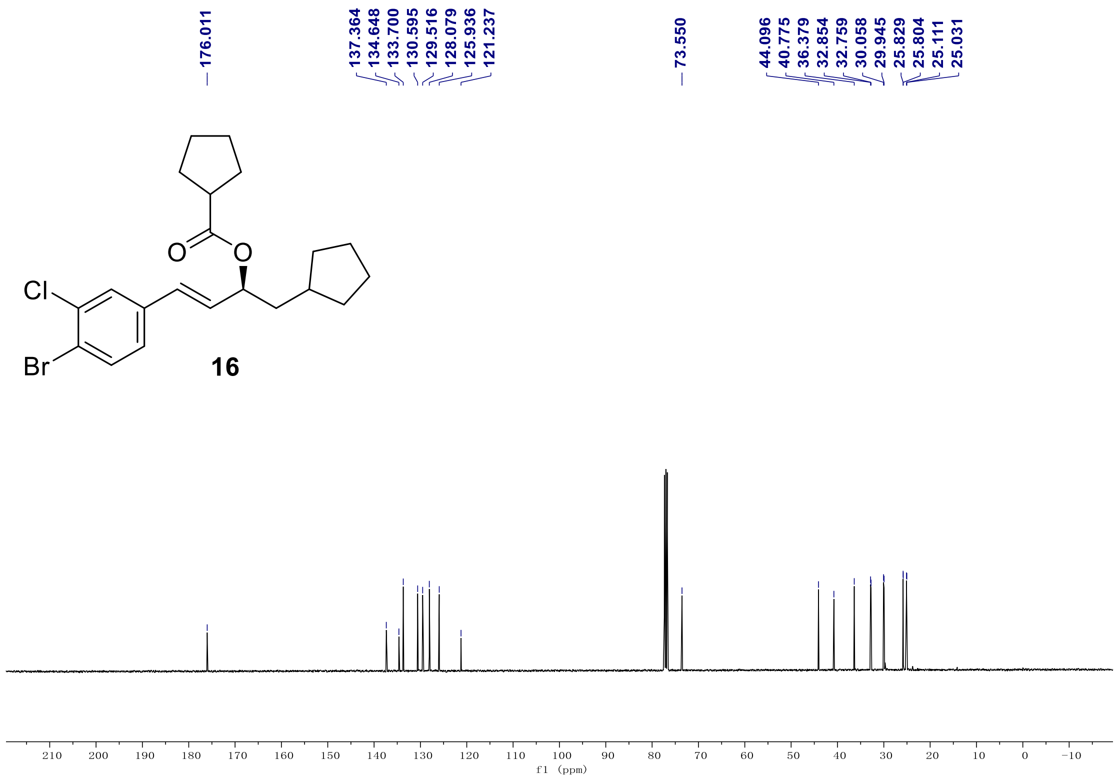

Supplementary Figure 122. ^{13}C NMR spectra of compound **16**

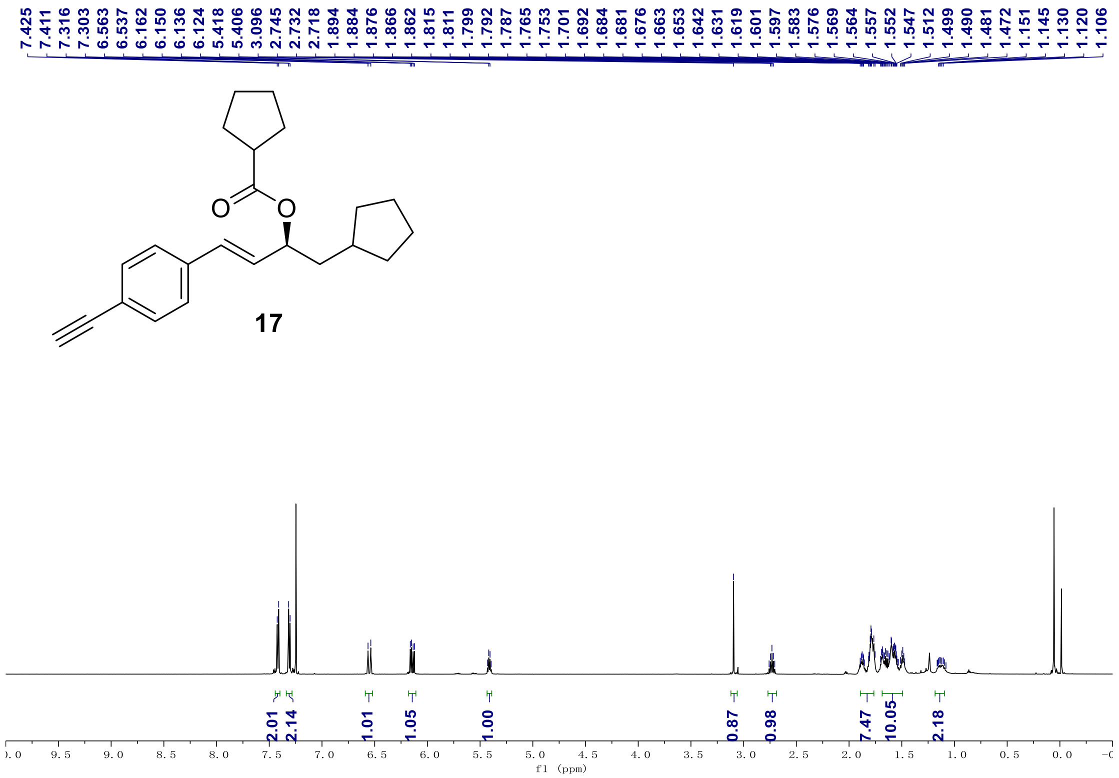

Supplementary Figure 123. ^{1}H NMR spectra of compound **17**

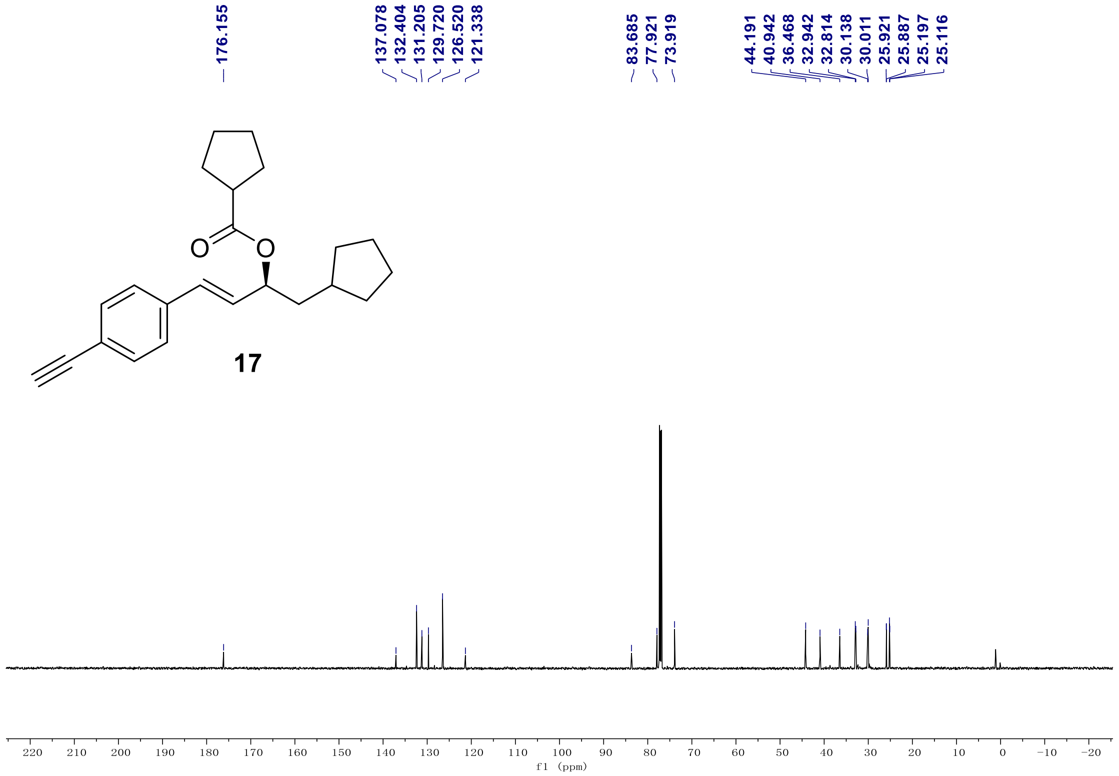

Supplementary Figure 124. ^{13}C NMR spectra of compound **17**

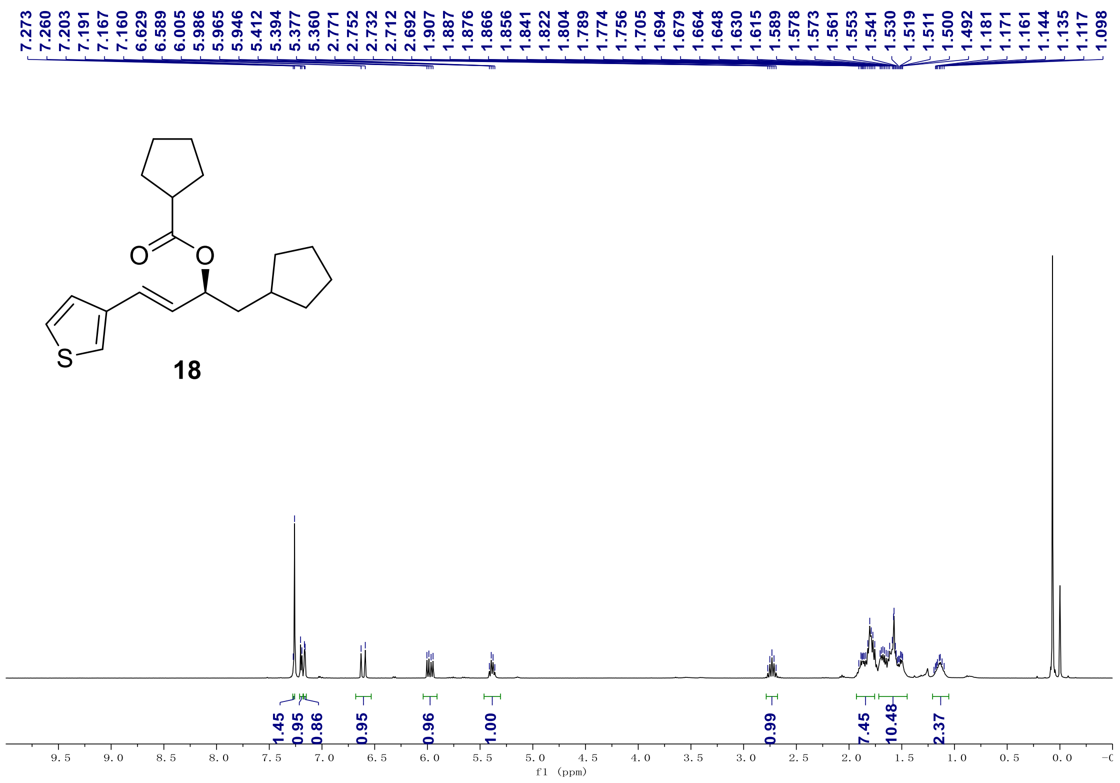

Supplementary Figure 125. ^{1}H NMR spectra of compound **18**

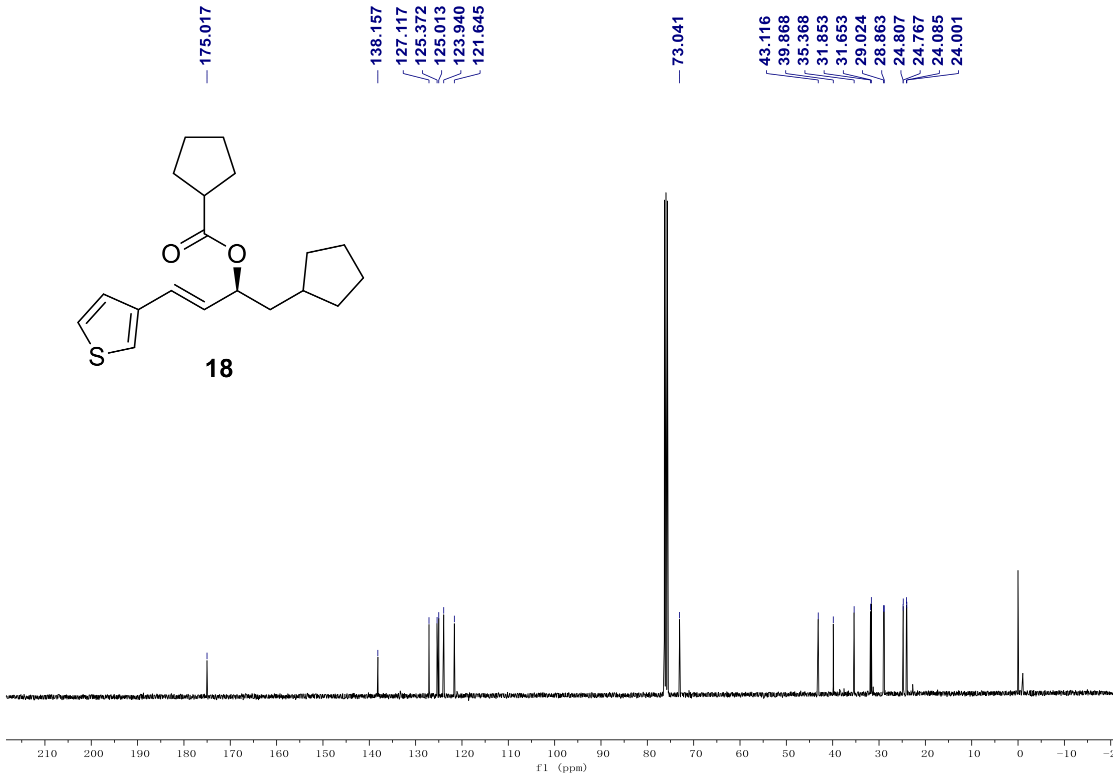

Supplementary Figure 126. ^{13}C NMR spectra of compound **18**

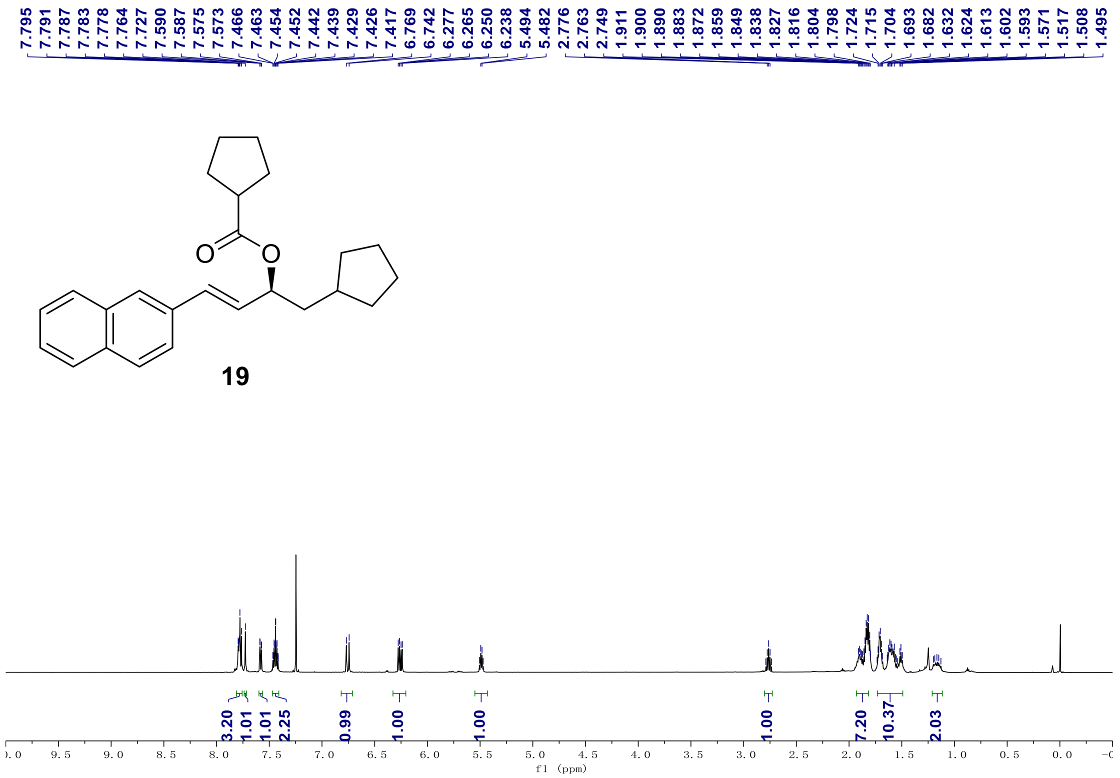

Supplementary Figure 127. ^{1}H NMR spectra of compound **19**

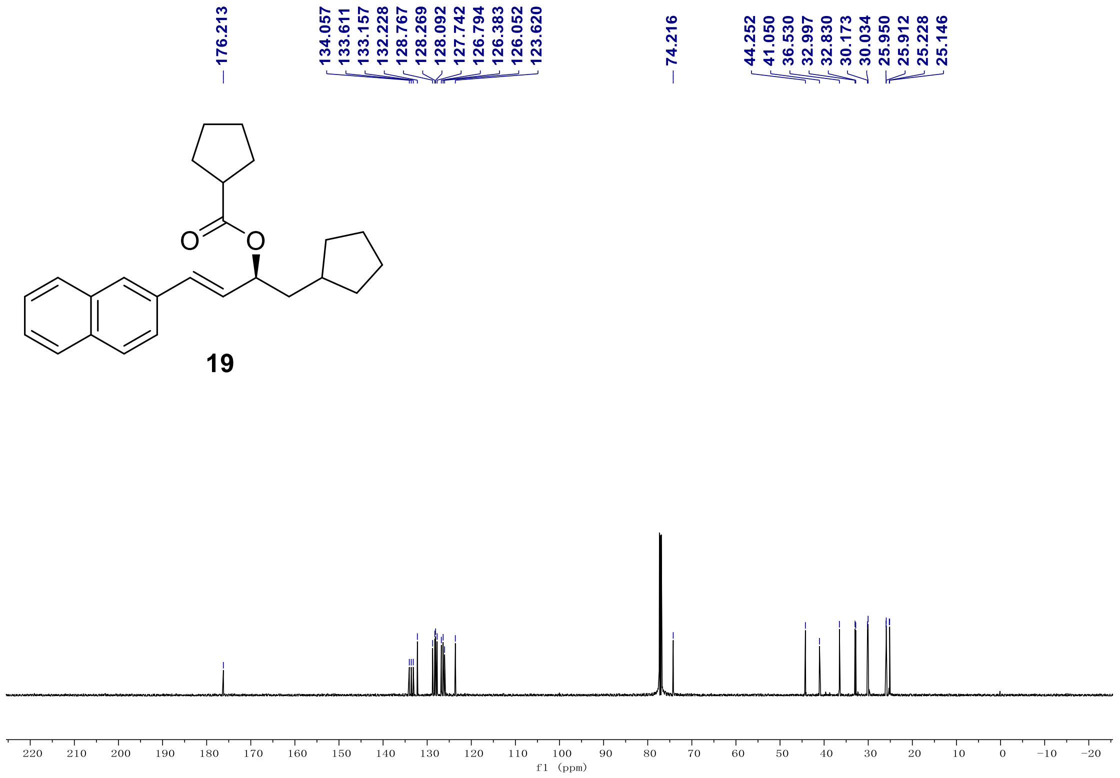

Supplementary Figure 128. ^{13}C NMR spectra of compound **19**

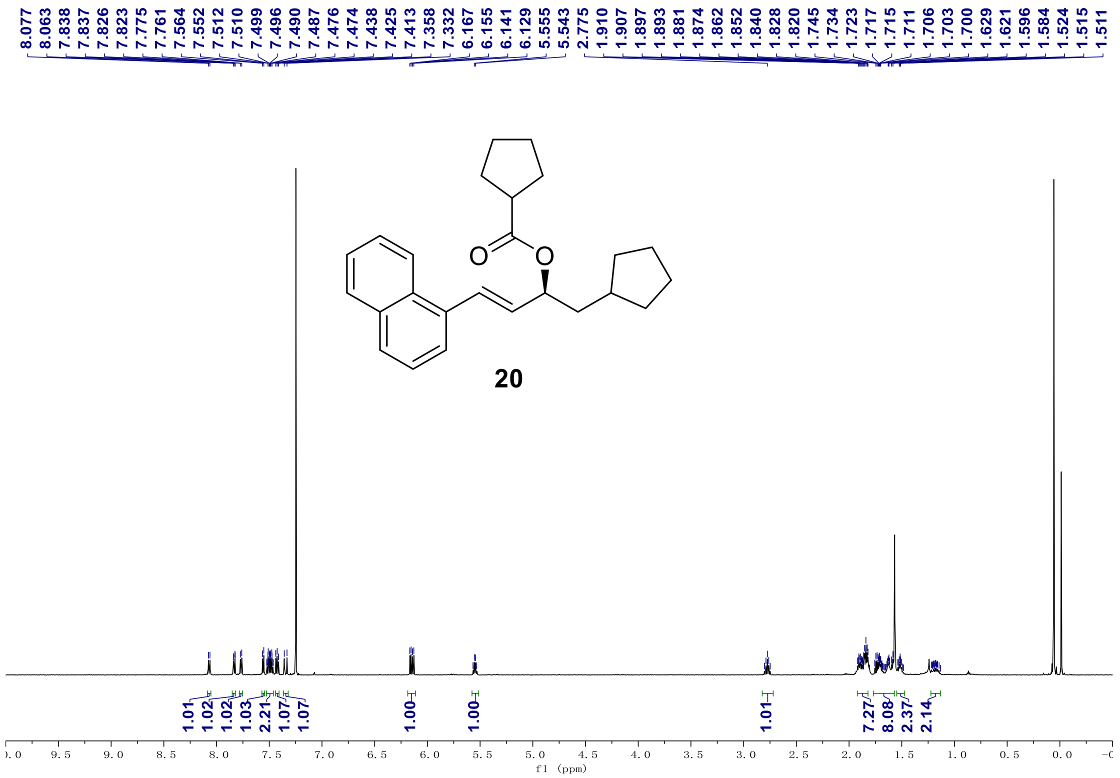

Supplementary Figure 129. ^{1}H NMR spectra of compound **20**

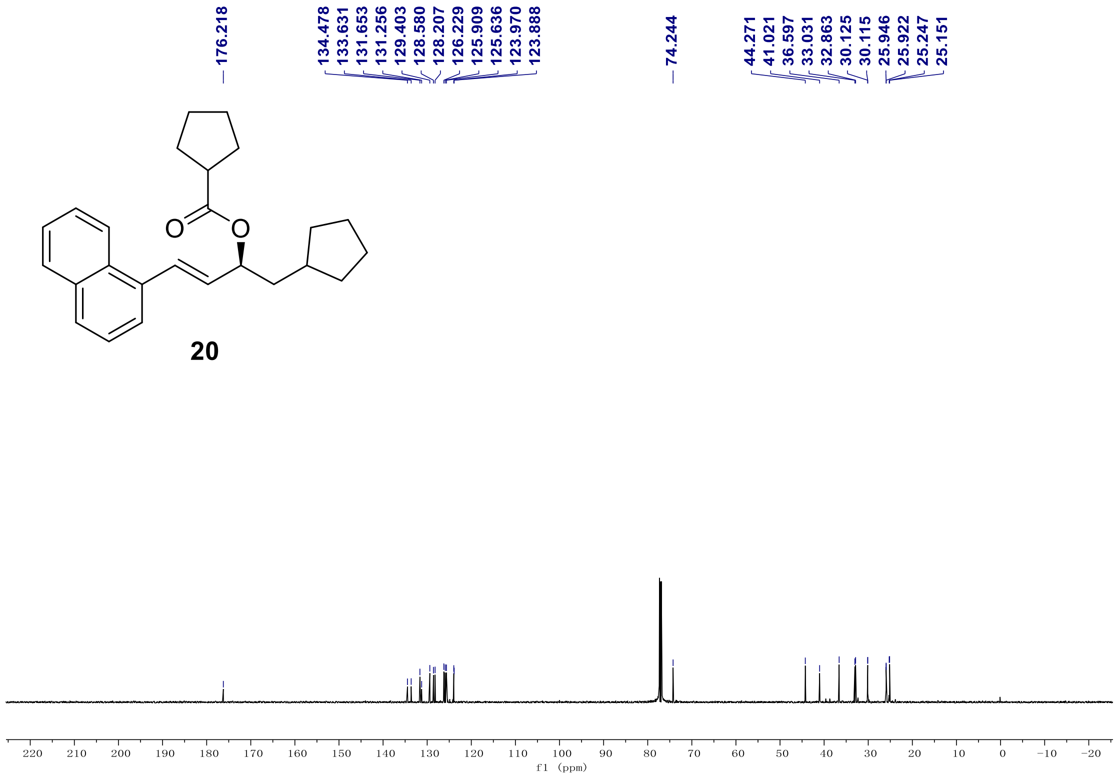

Supplementary Figure 130. ^{13}C NMR spectra of compound **20**

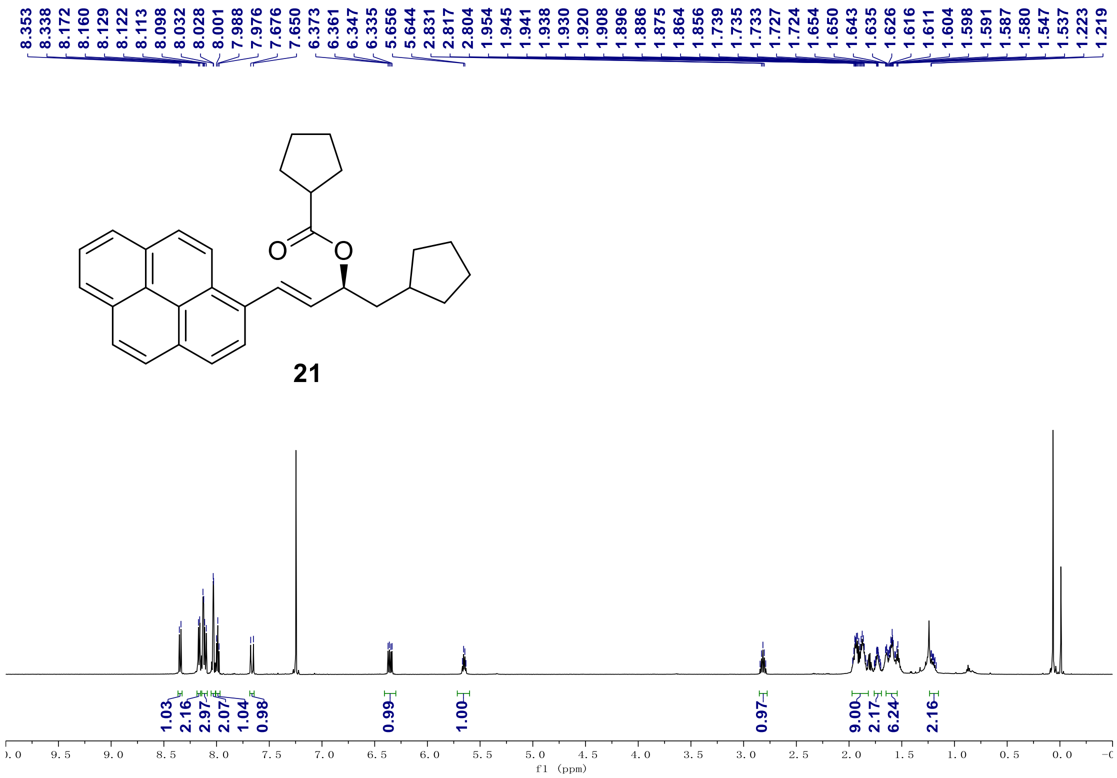

Supplementary Figure 131. ^{1}H NMR spectra of compound **21**

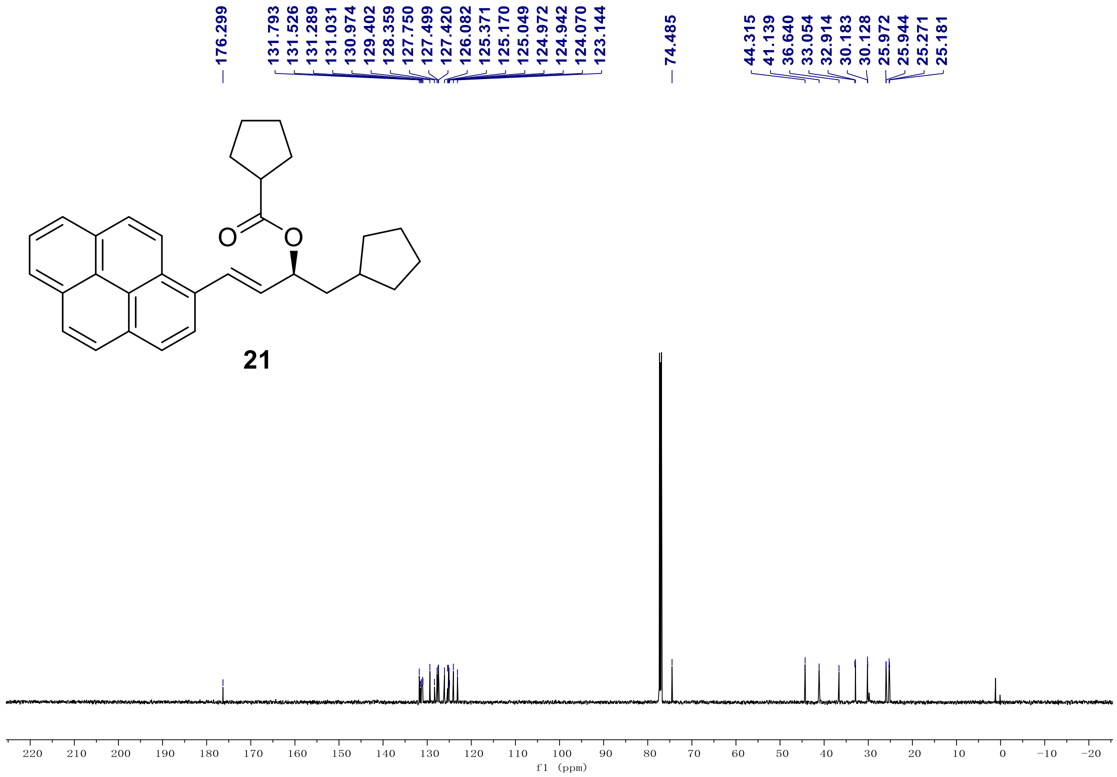

Supplementary Figure 132. ^{13}C NMR spectra of compound **21**

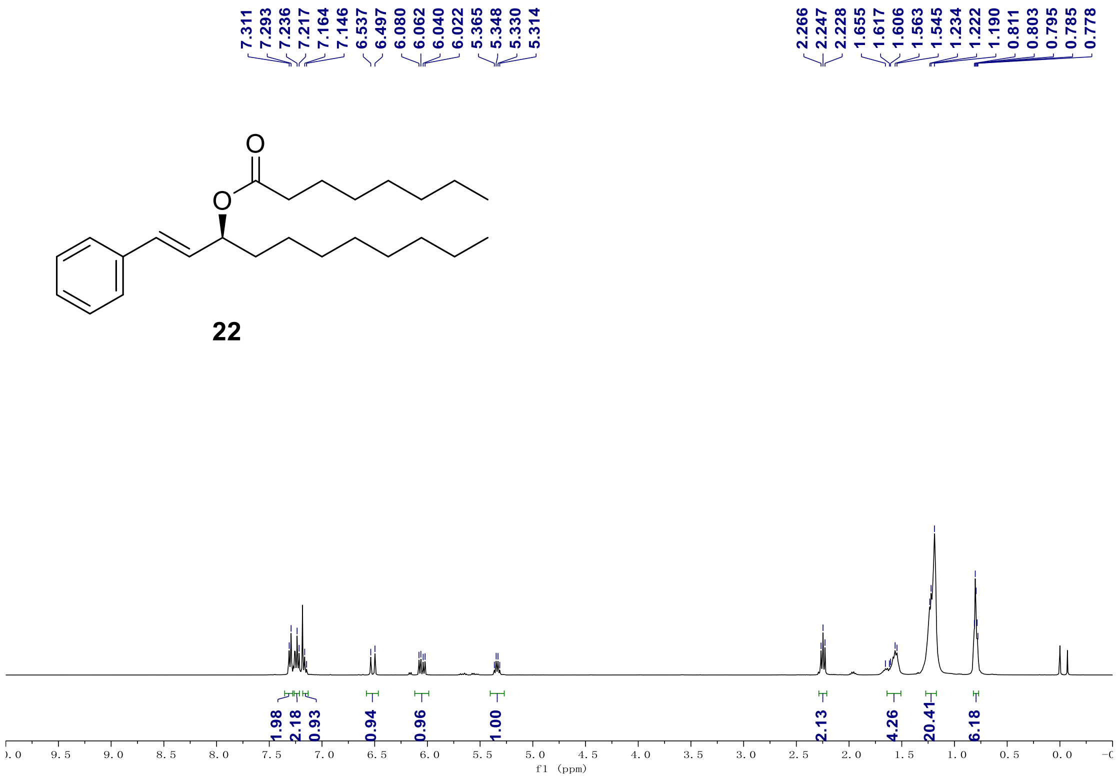

Supplementary Figure 133. ^{1}H NMR spectra of compound **22**

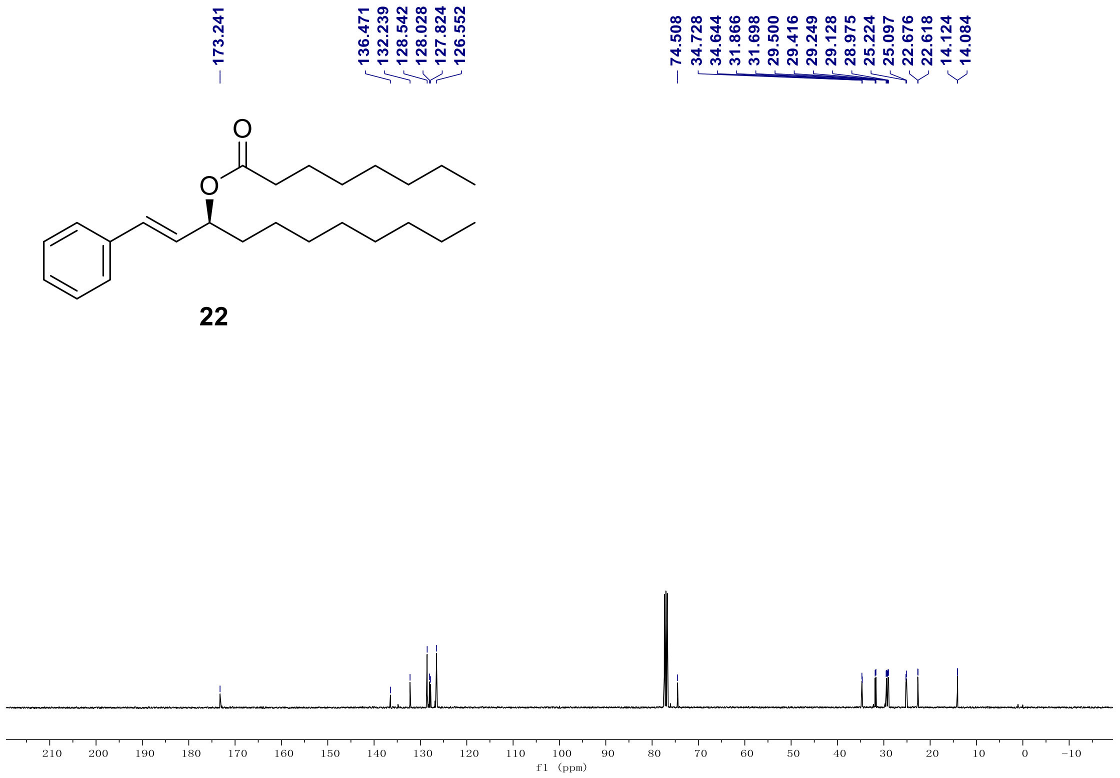

Supplementary Figure 134. ^{13}C NMR spectra of compound **22**

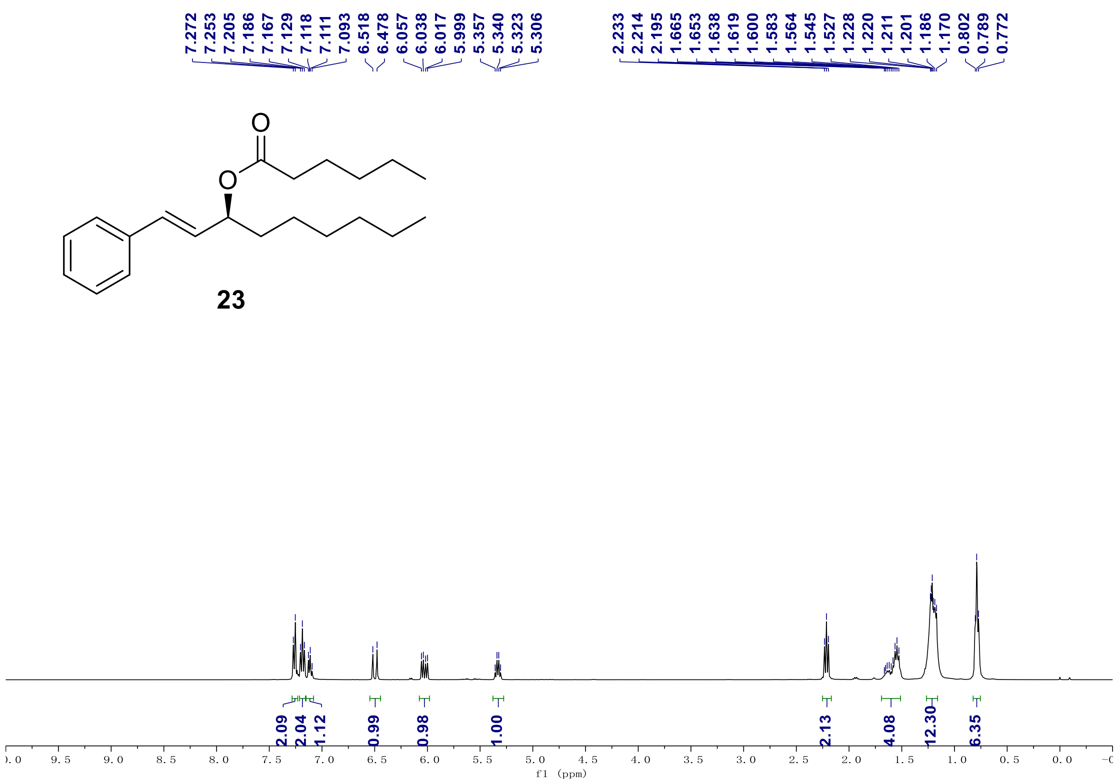

Supplementary Figure 135. ^{1}H NMR spectra of compound **23**

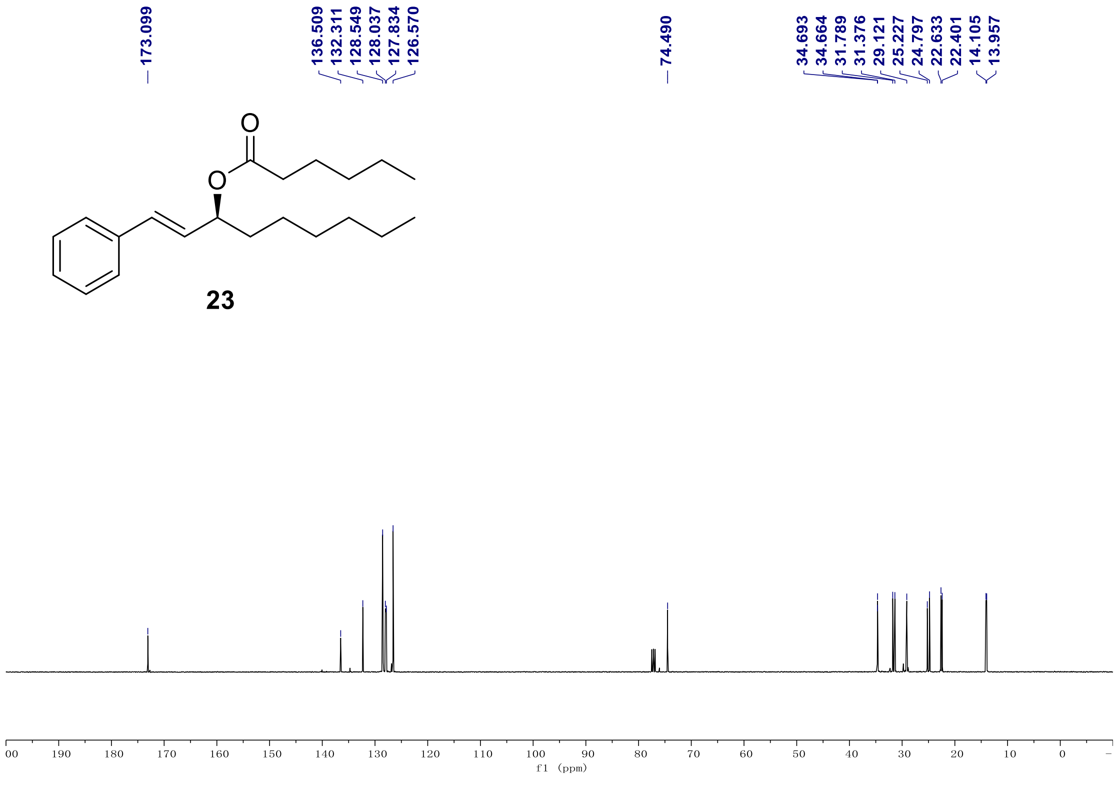

Supplementary Figure 134. ^{13}C NMR spectra of compound **23**

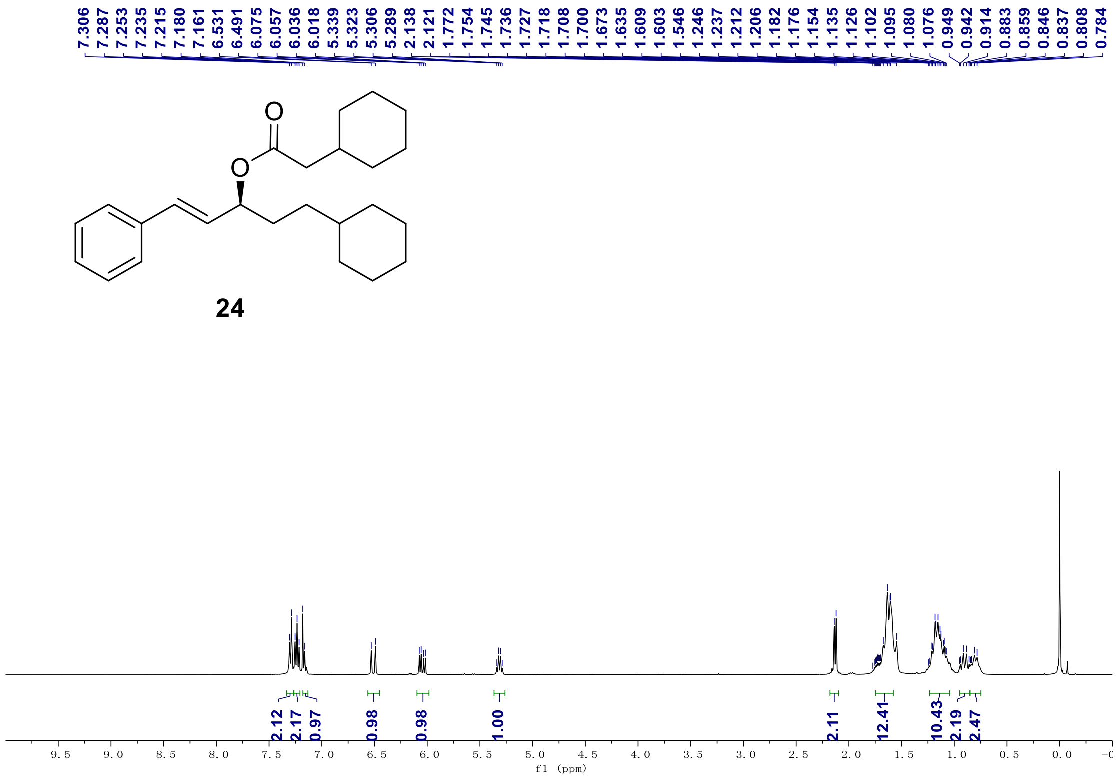

Supplementary Figure 135. ^{1}H NMR spectra of compound **24**

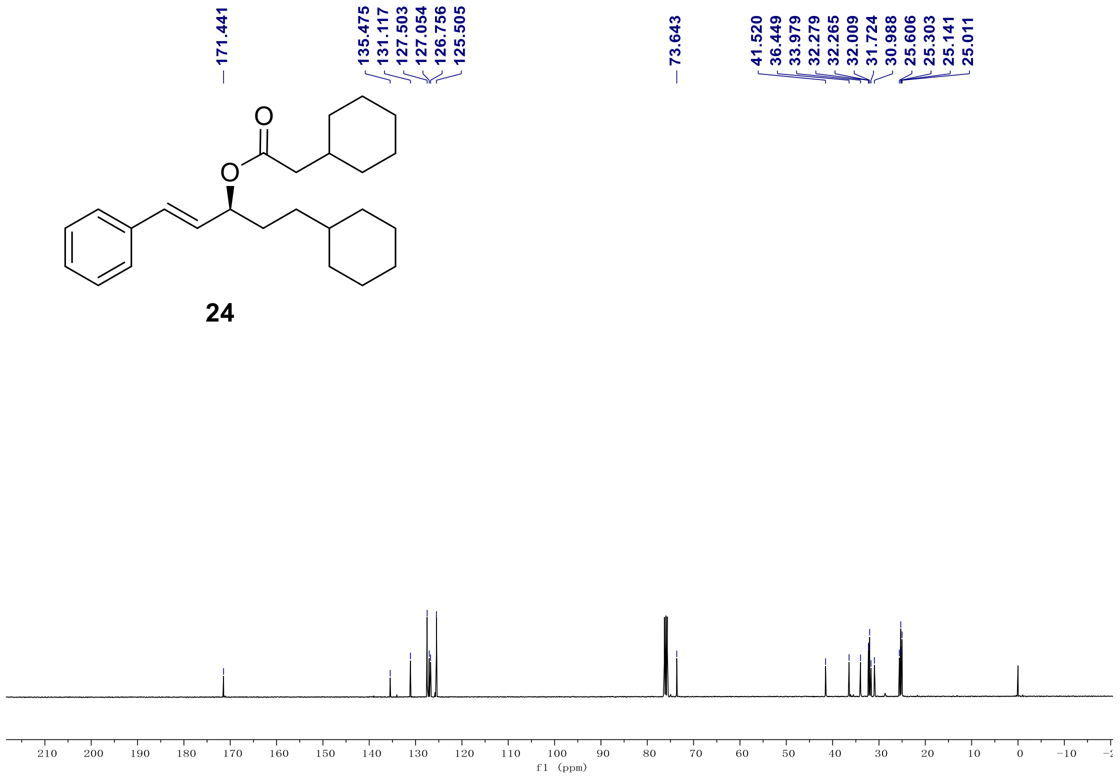

Supplementary Figure 136. ^{13}C NMR spectra of compound **24**

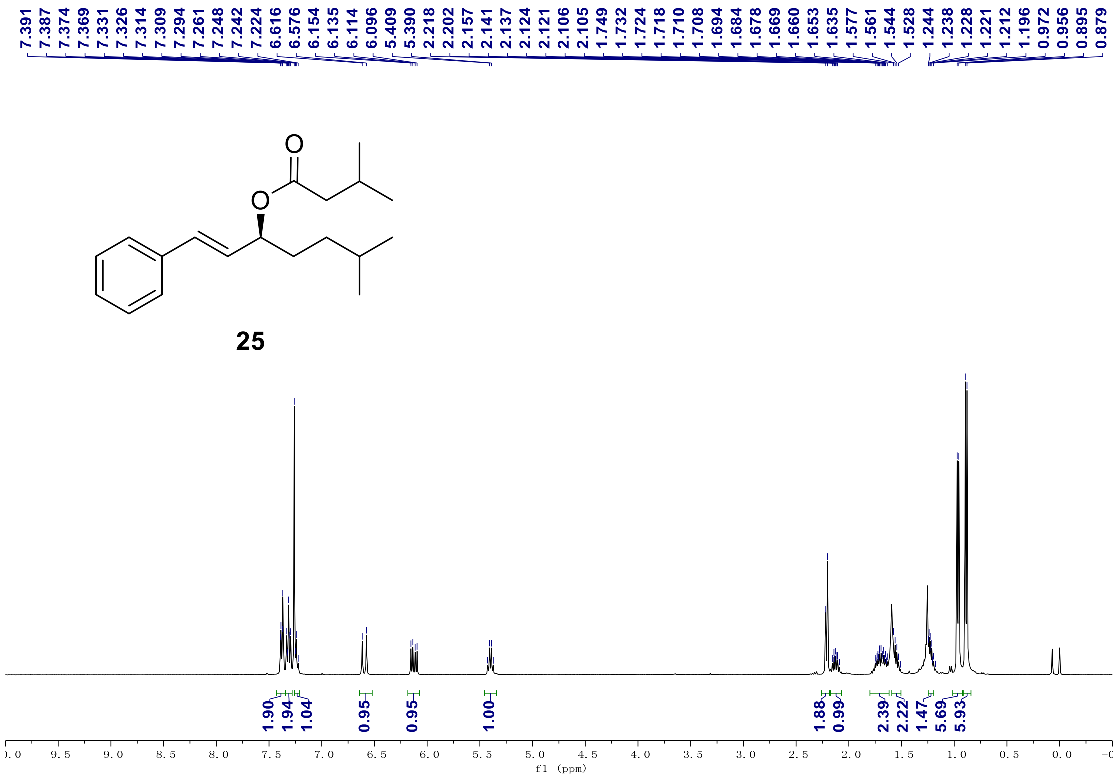

Supplementary Figure 137. ^{1}H NMR spectra of compound **25**

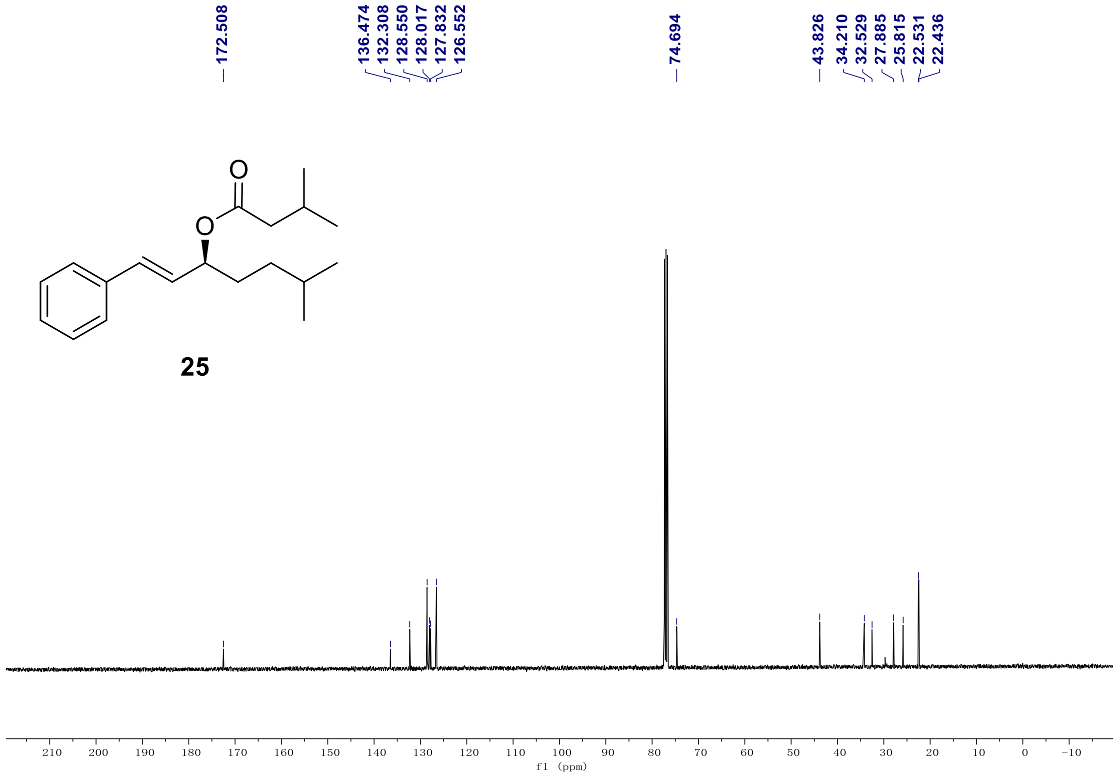

Supplementary Figure 138. ^{13}C NMR spectra of compound **25**

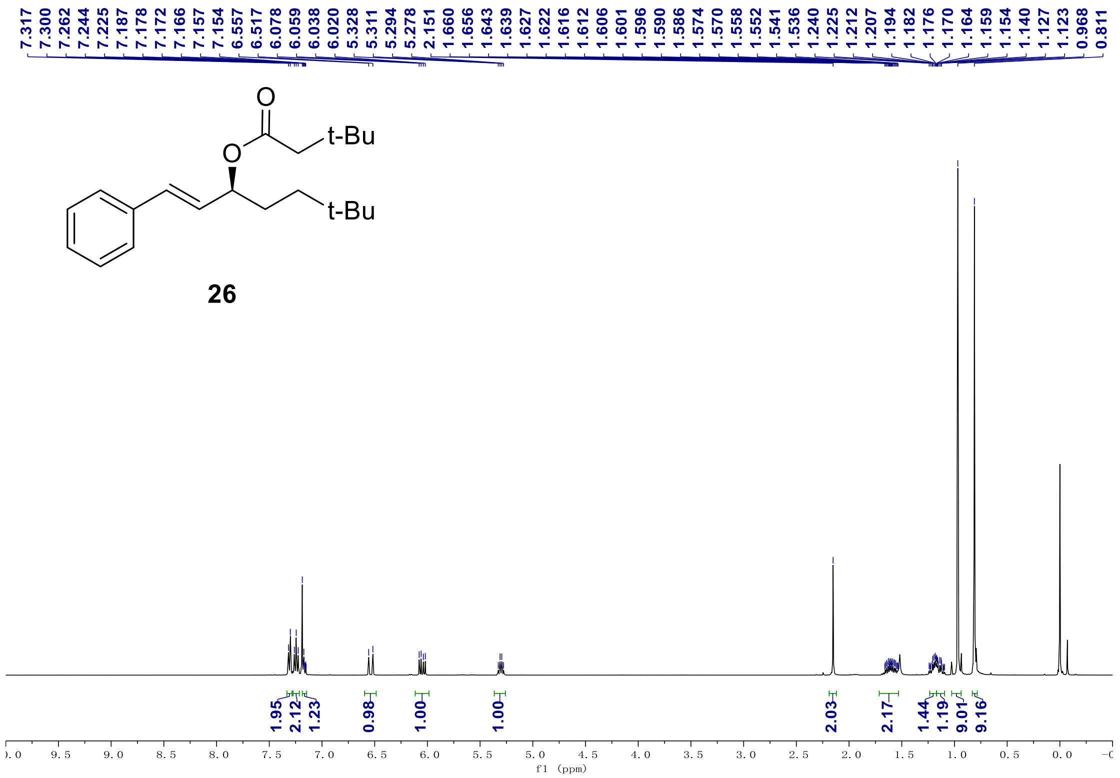

Supplementary Figure 139. ^{1}H NMR spectra of compound **26**

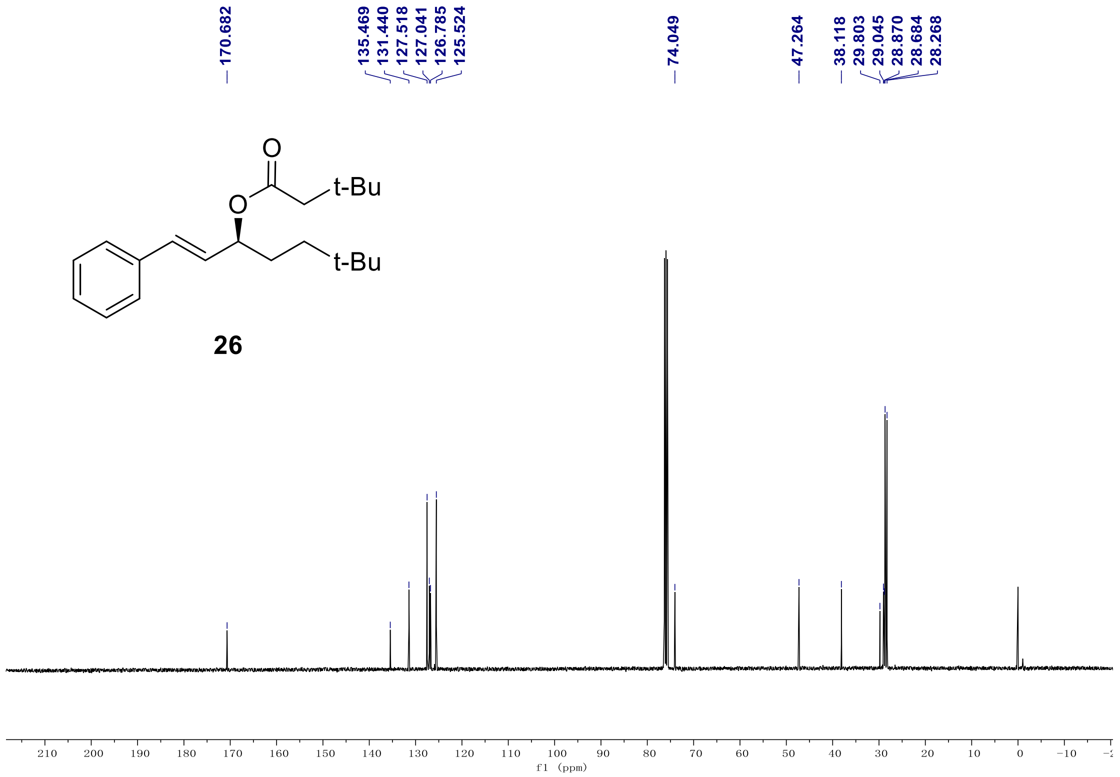

Supplementary Figure 140. ^{13}C NMR spectra of compound **26**

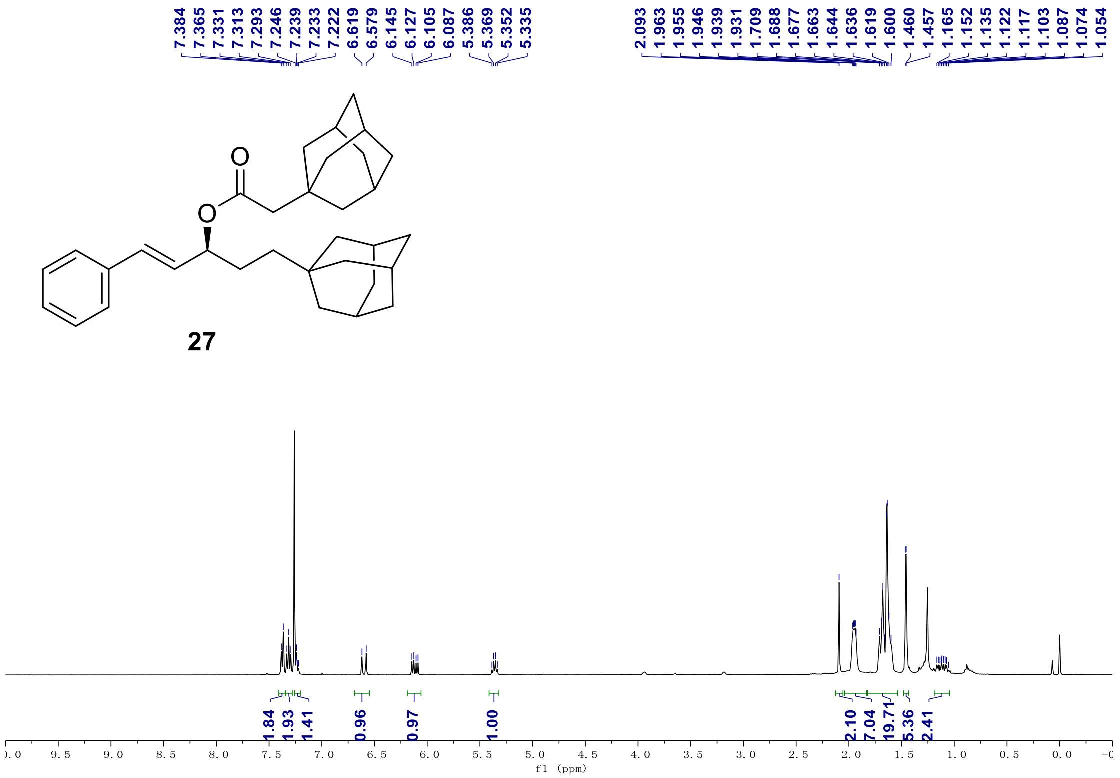

Supplementary Figure 141. ^{1}H NMR spectra of compound **27**

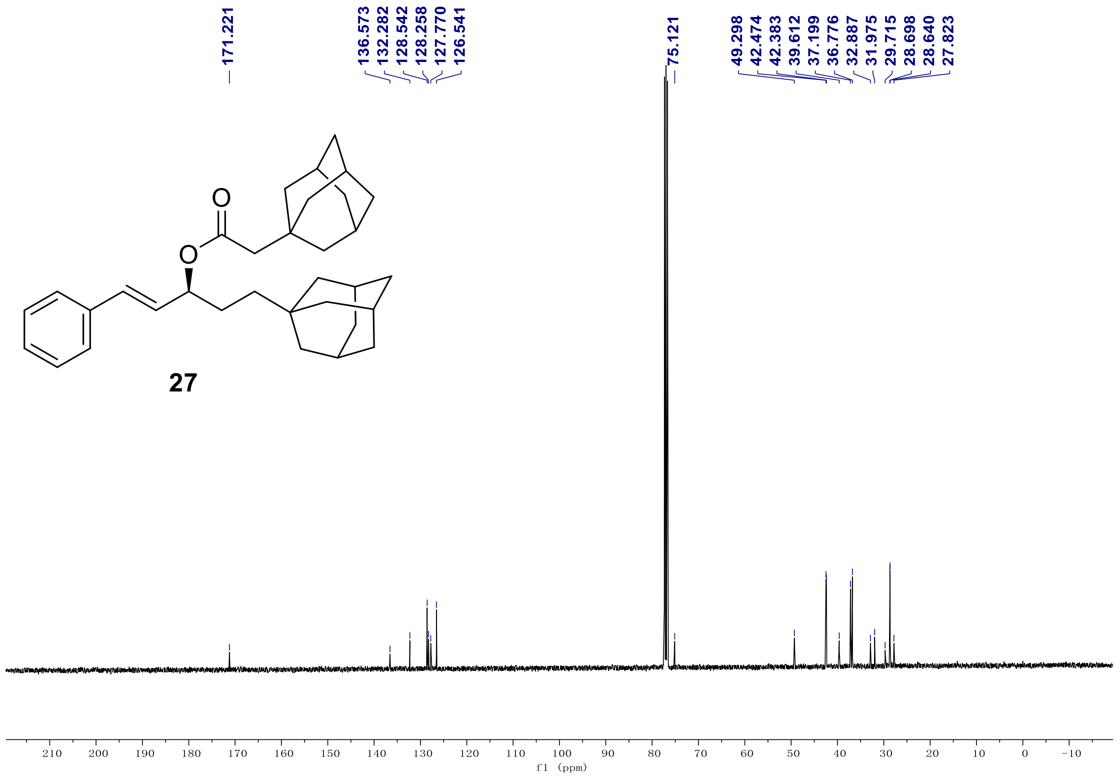

Supplementary Figure 142. ^{13}C NMR spectra of compound **27**

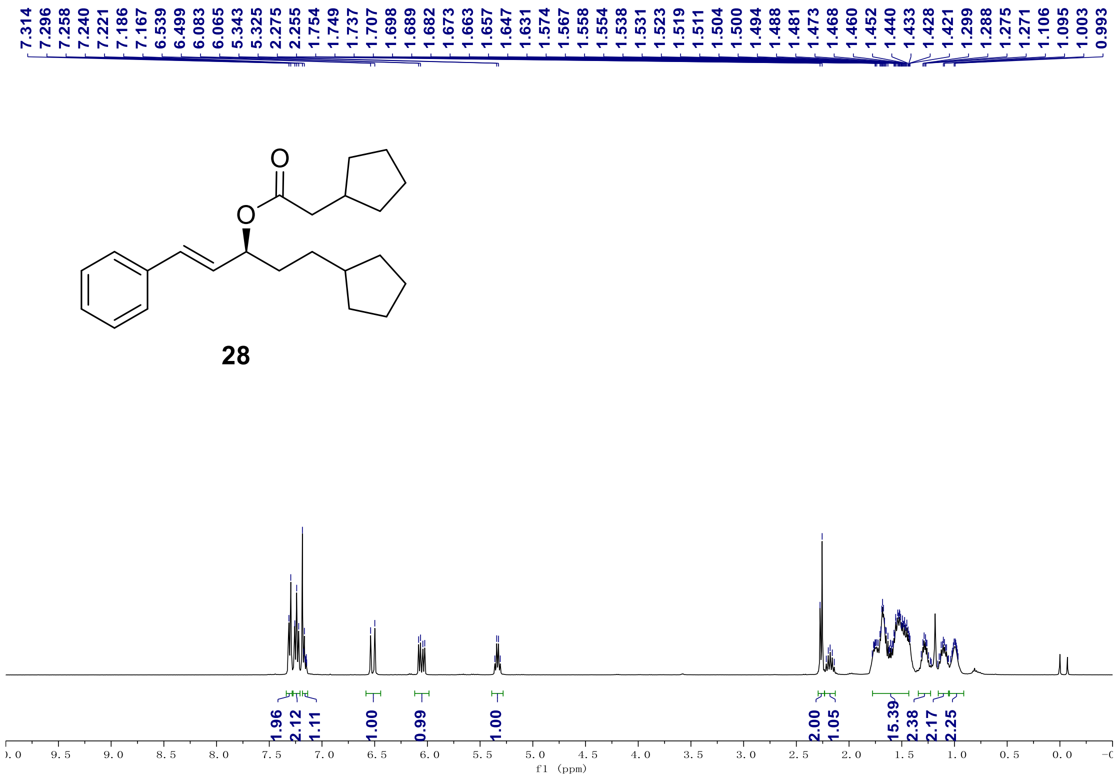

Supplementary Figure 143. ^{1}H NMR spectra of compound **28**

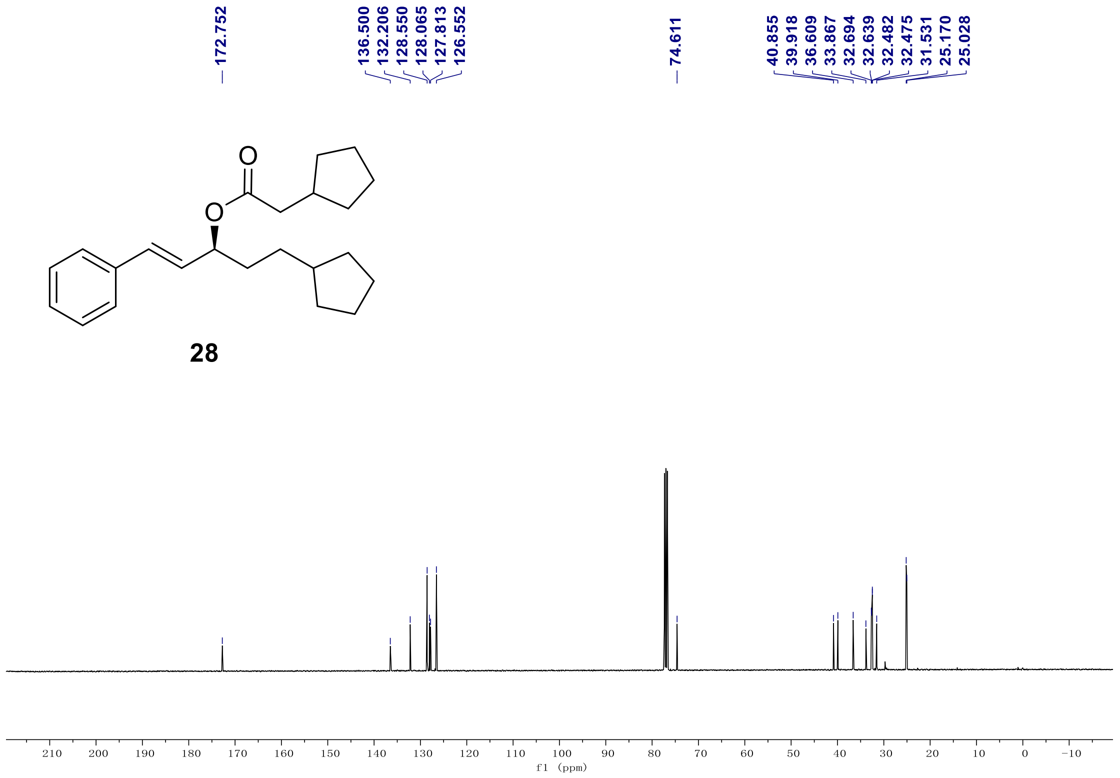

Supplementary Figure 144. ^{13}C NMR spectra of compound **28**

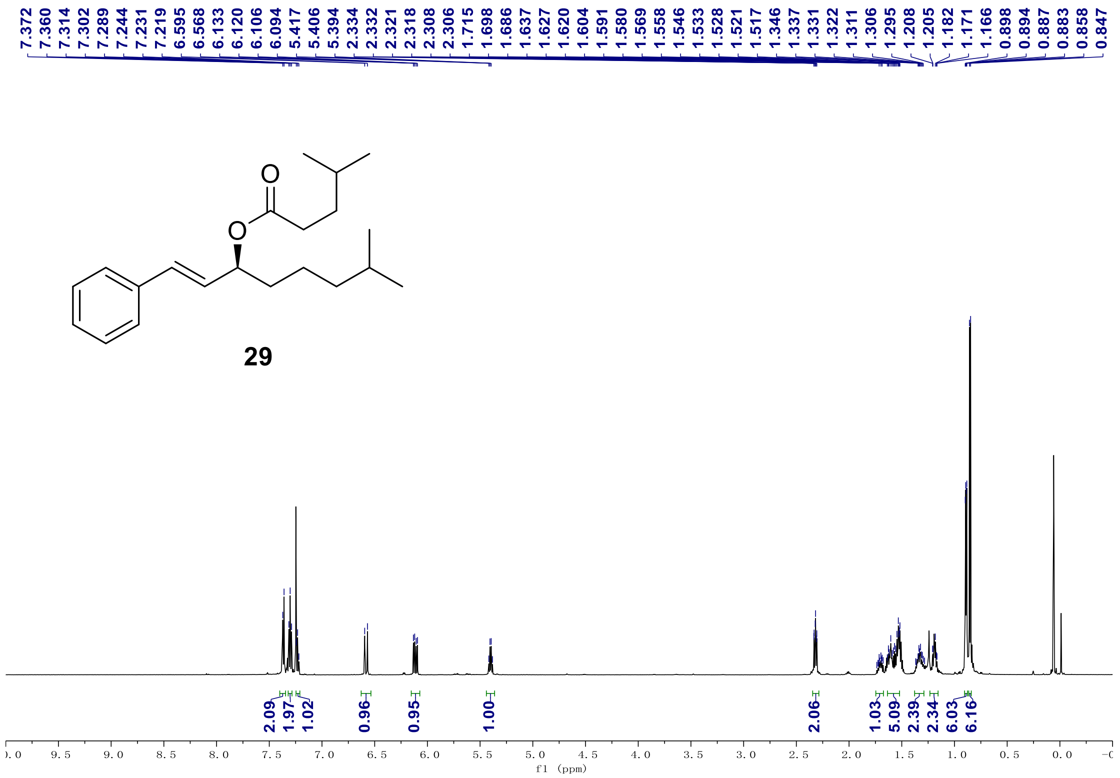

Supplementary Figure 145. ^{1}H NMR spectra of compound **29**

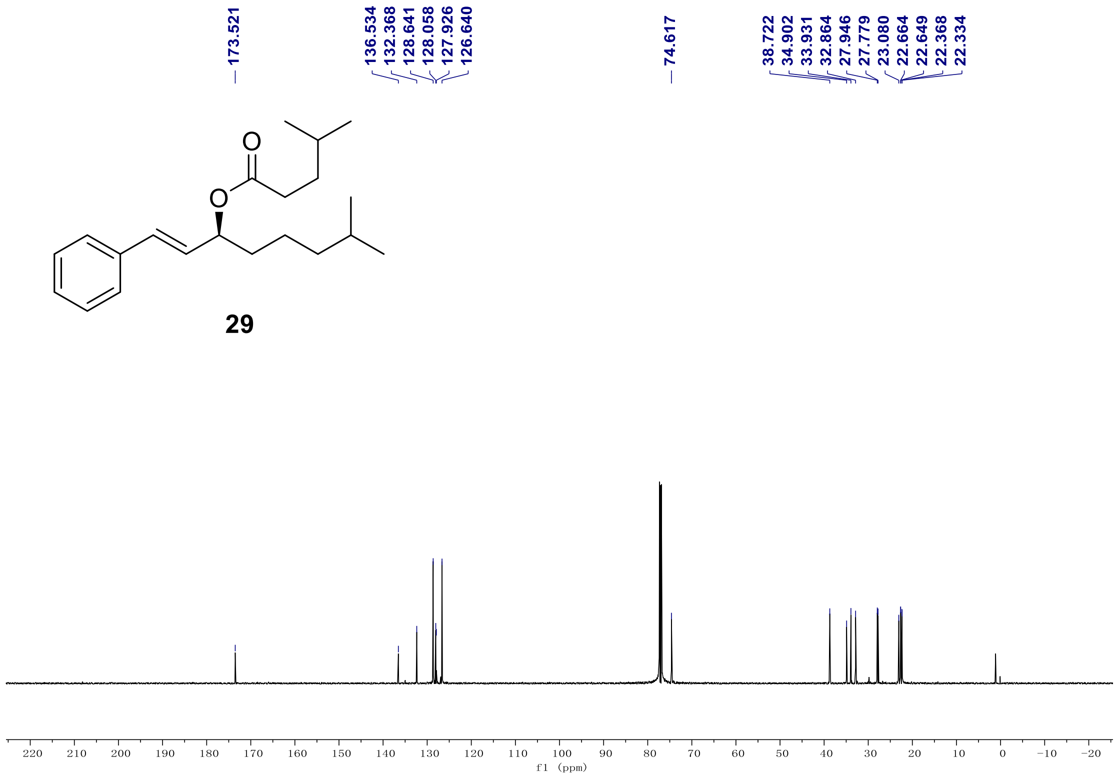

Supplementary Figure 146. ^{13}C NMR spectra of compound **29**

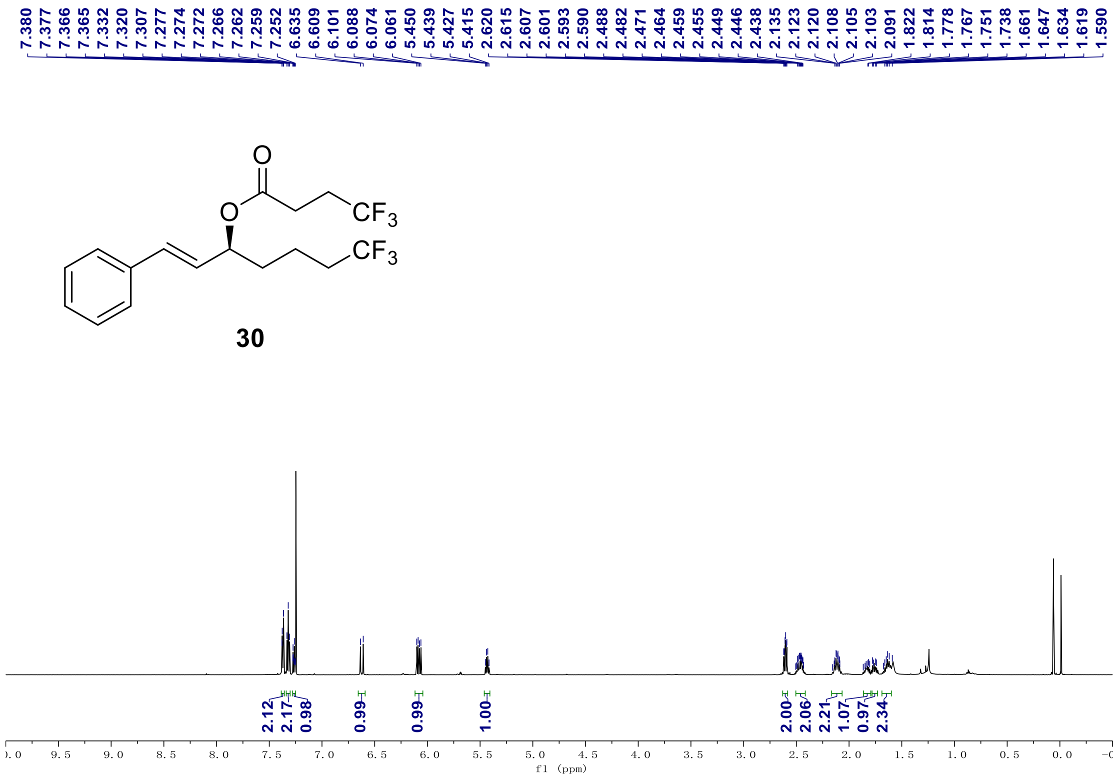

Supplementary Figure 147. ^{1}H NMR spectra of compound **30**

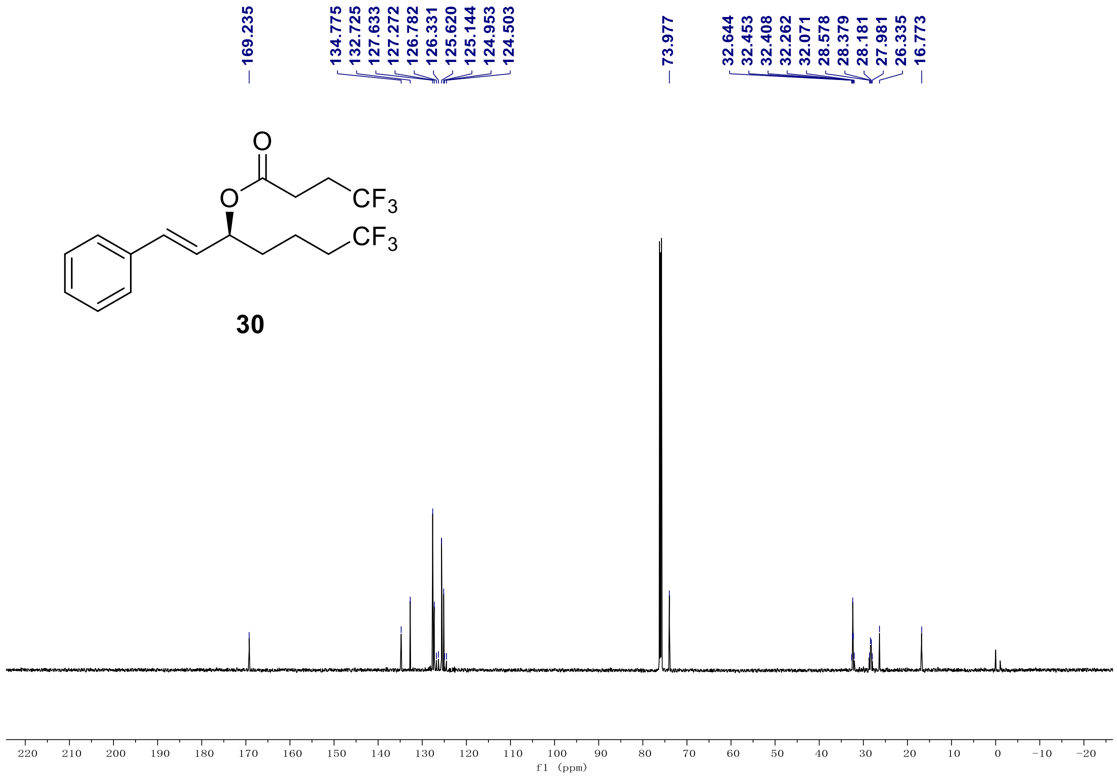

Supplementary Figure 148. ^{13}C NMR spectra of compound **30**

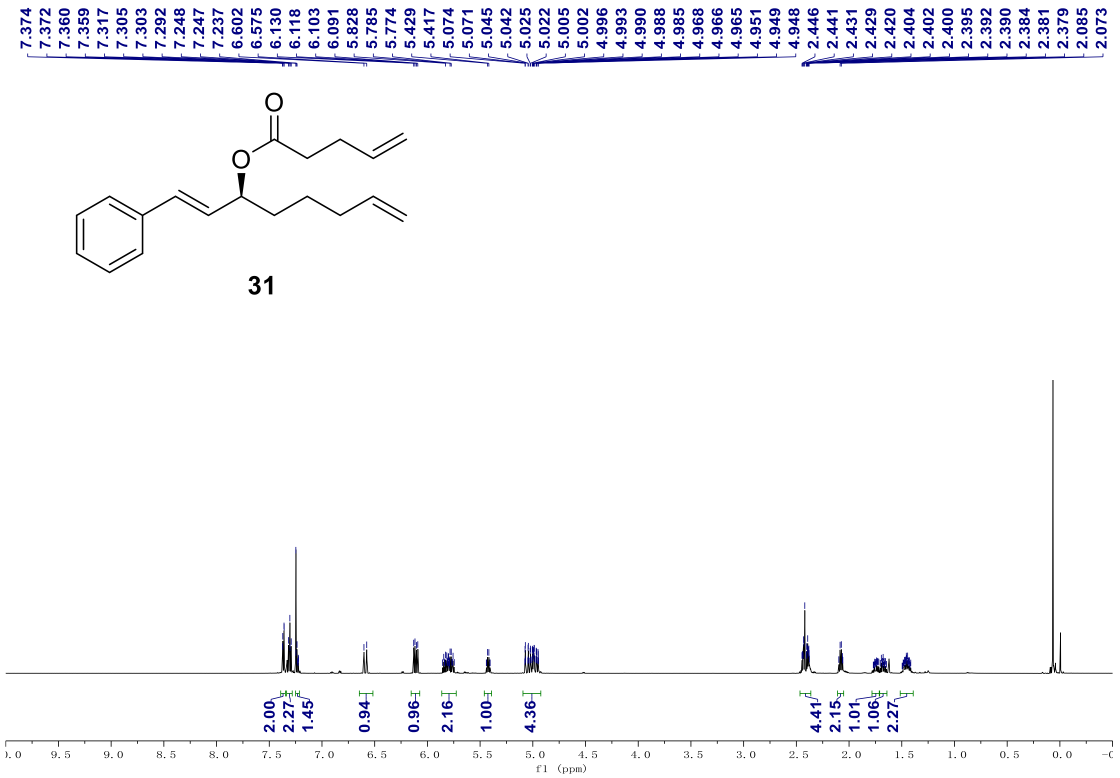

Supplementary Figure 149. ^{1}H NMR spectra of compound **31**

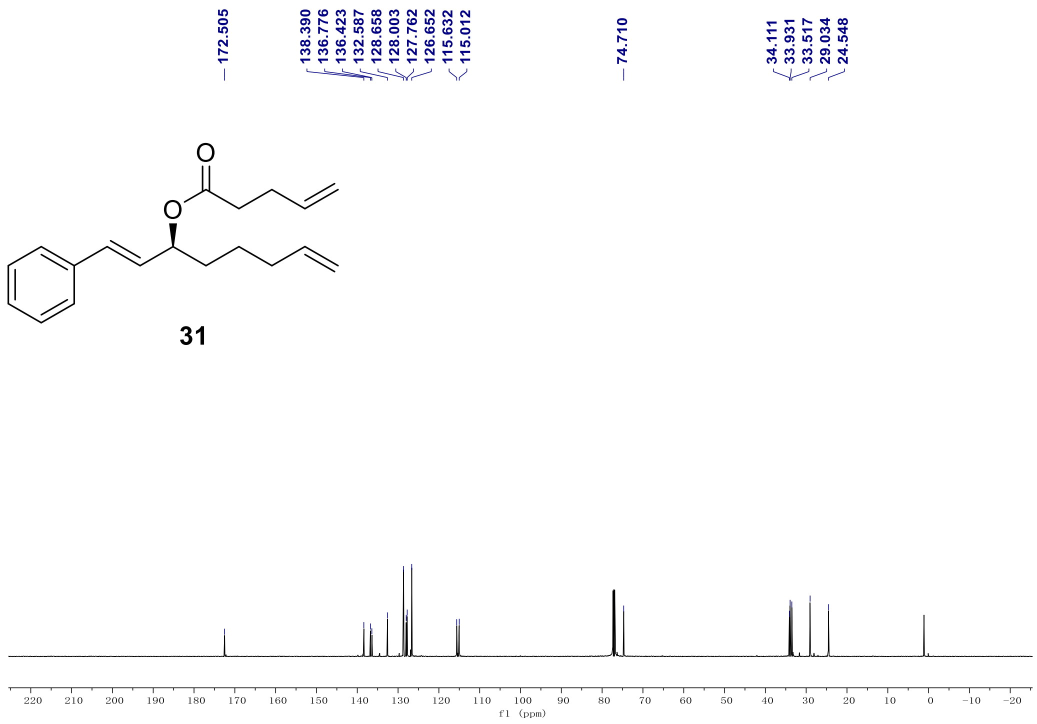

Supplementary Figure 150. ^{13}C NMR spectra of compound **31**

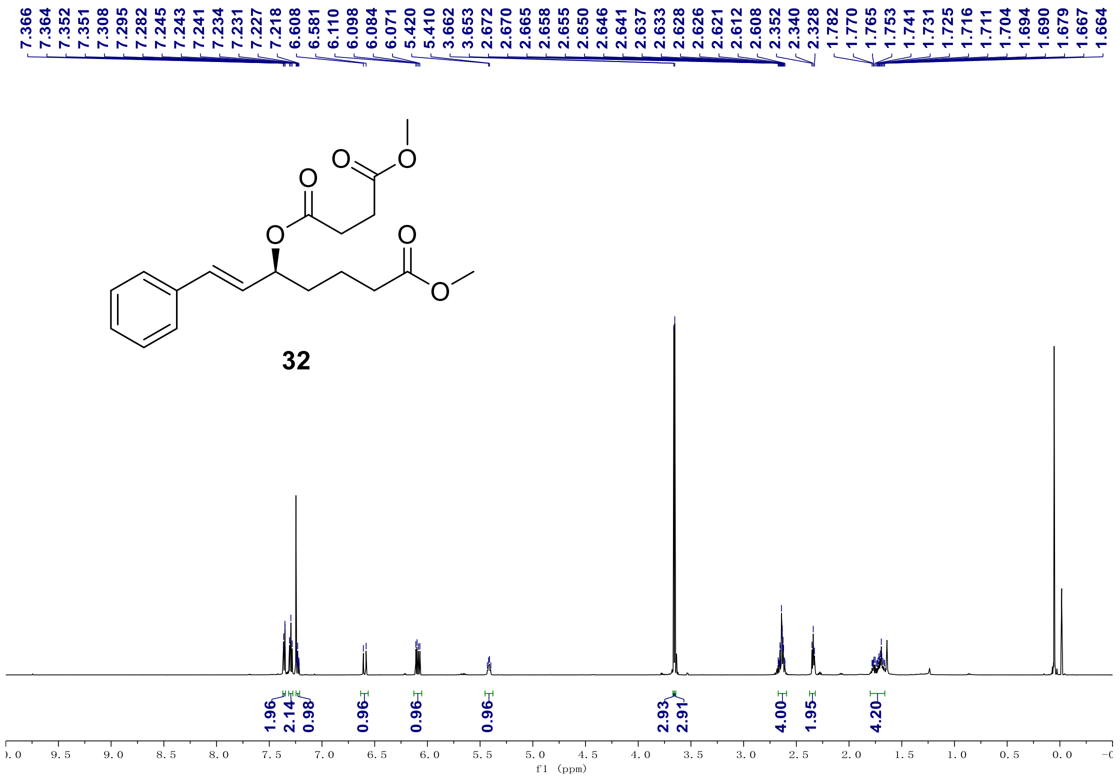

Supplementary Figure 151. ^{1}H NMR spectra of compound **32**

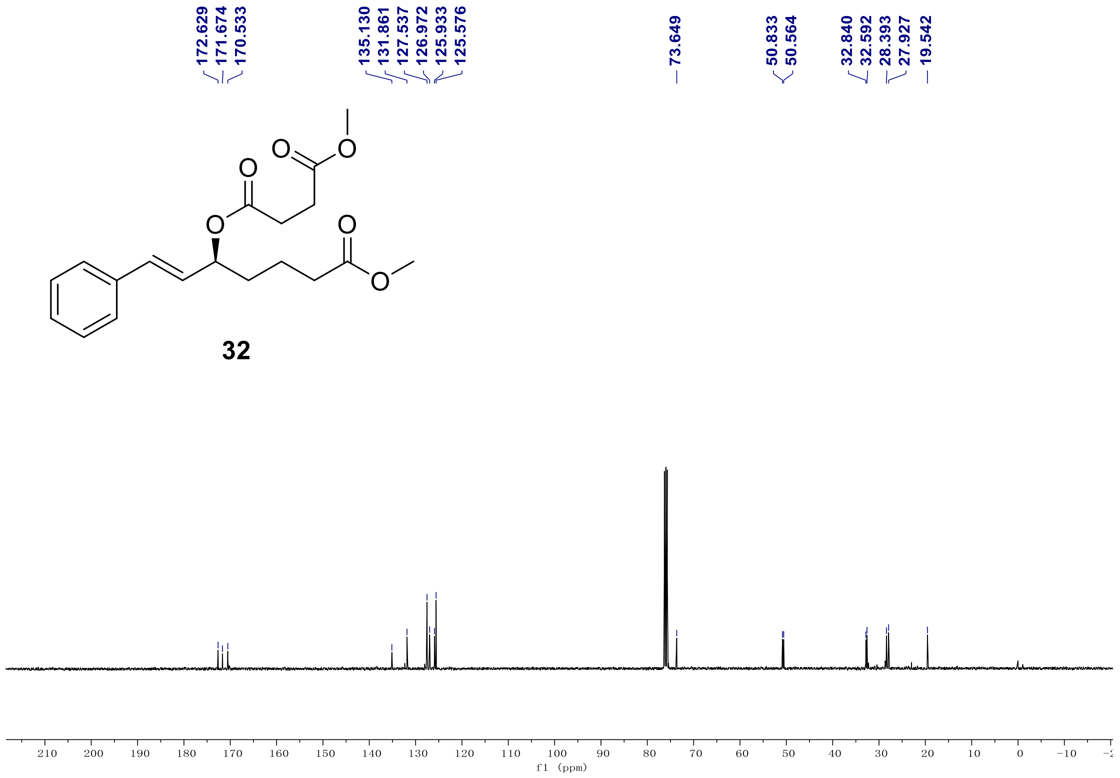

Supplementary Figure 152. ^{13}C NMR spectra of compound **32**

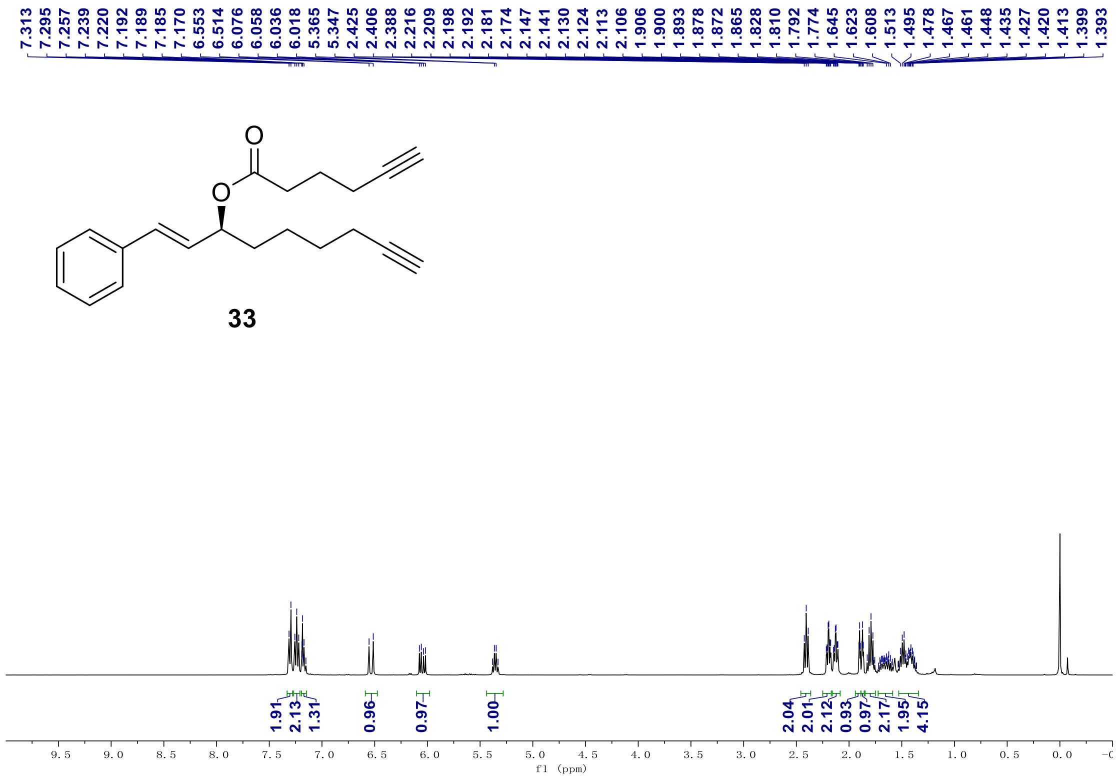

Supplementary Figure 153. ^{1}H NMR spectra of compound **33**

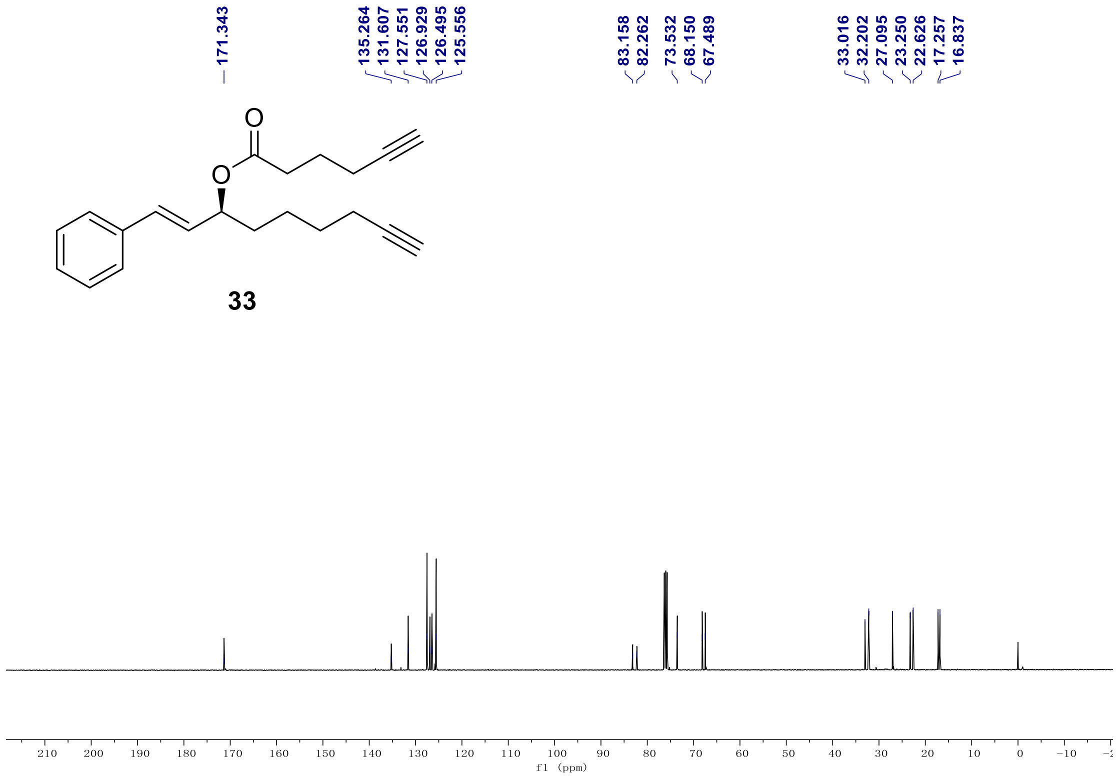

Supplementary Figure 154. ^{13}C NMR spectra of compound **33**

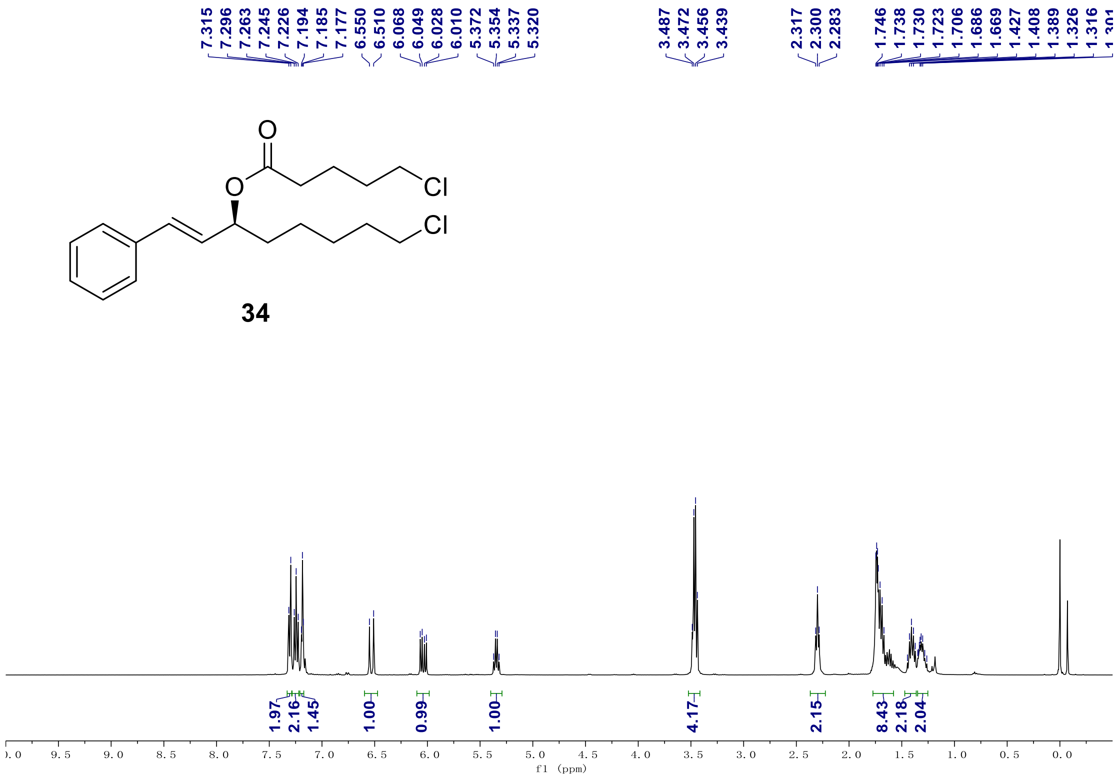

Supplementary Figure 155. ^{1}H NMR spectra of compound **34**

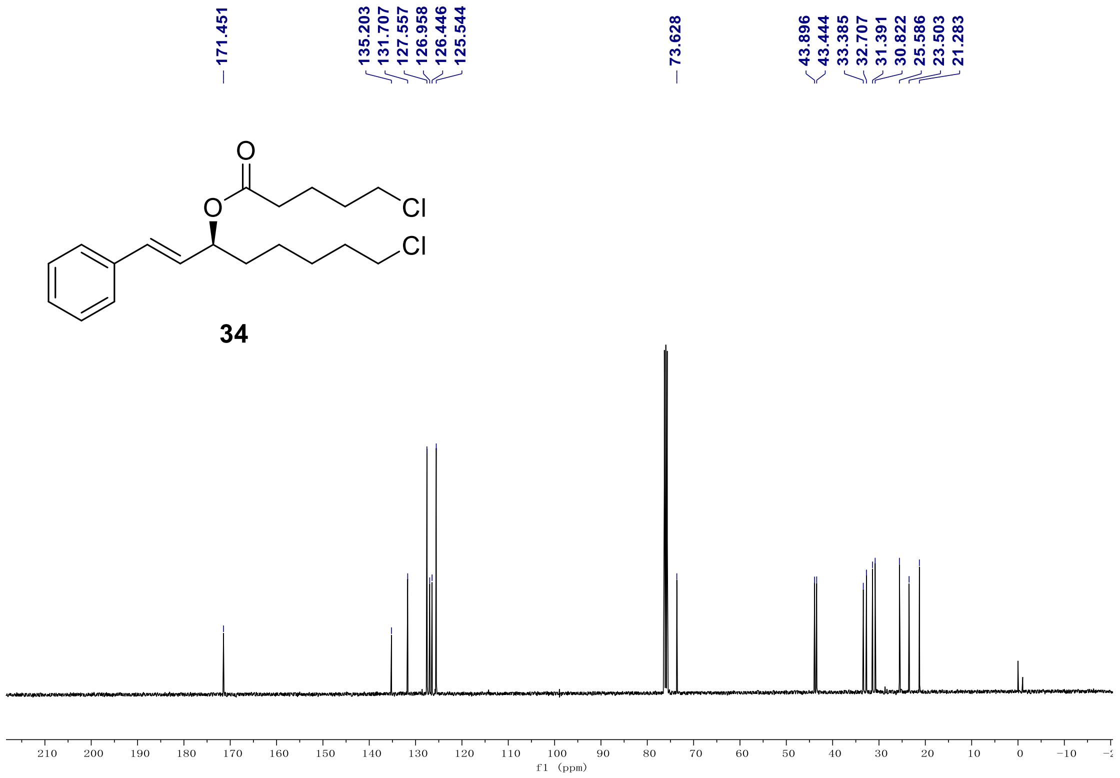

Supplementary Figure 156. ^{13}C NMR spectra of compound **34**

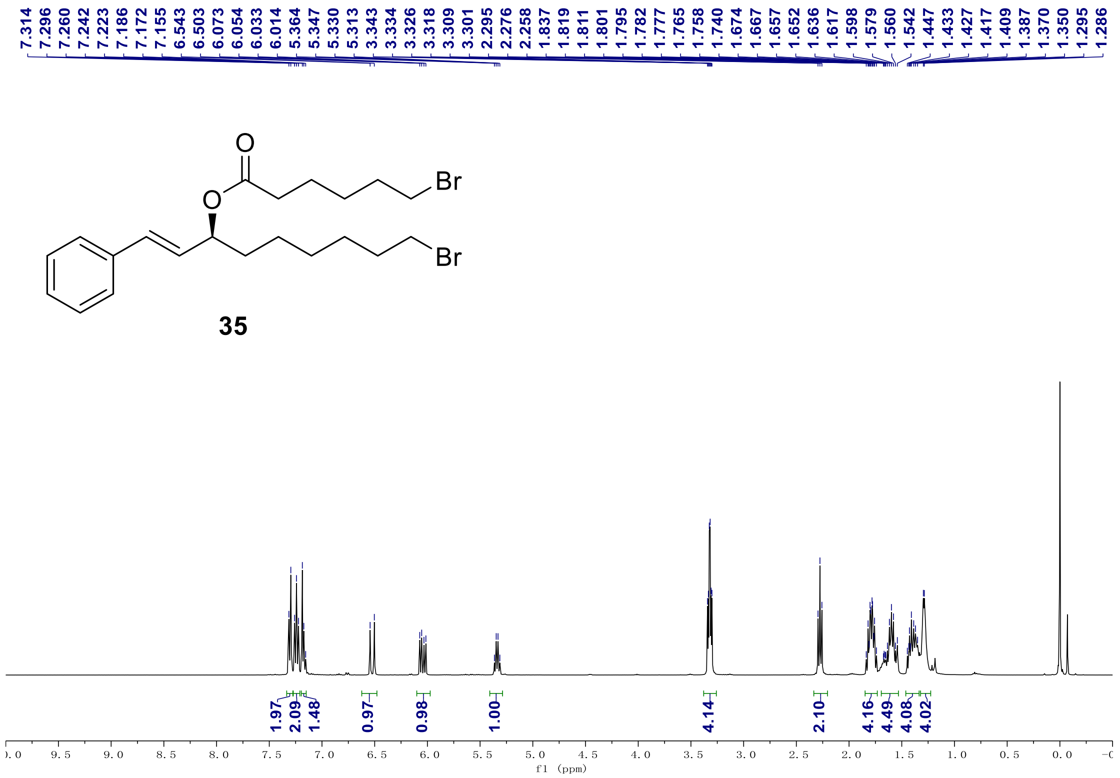

Supplementary Figure 157. ^{1}H NMR spectra of compound **35**

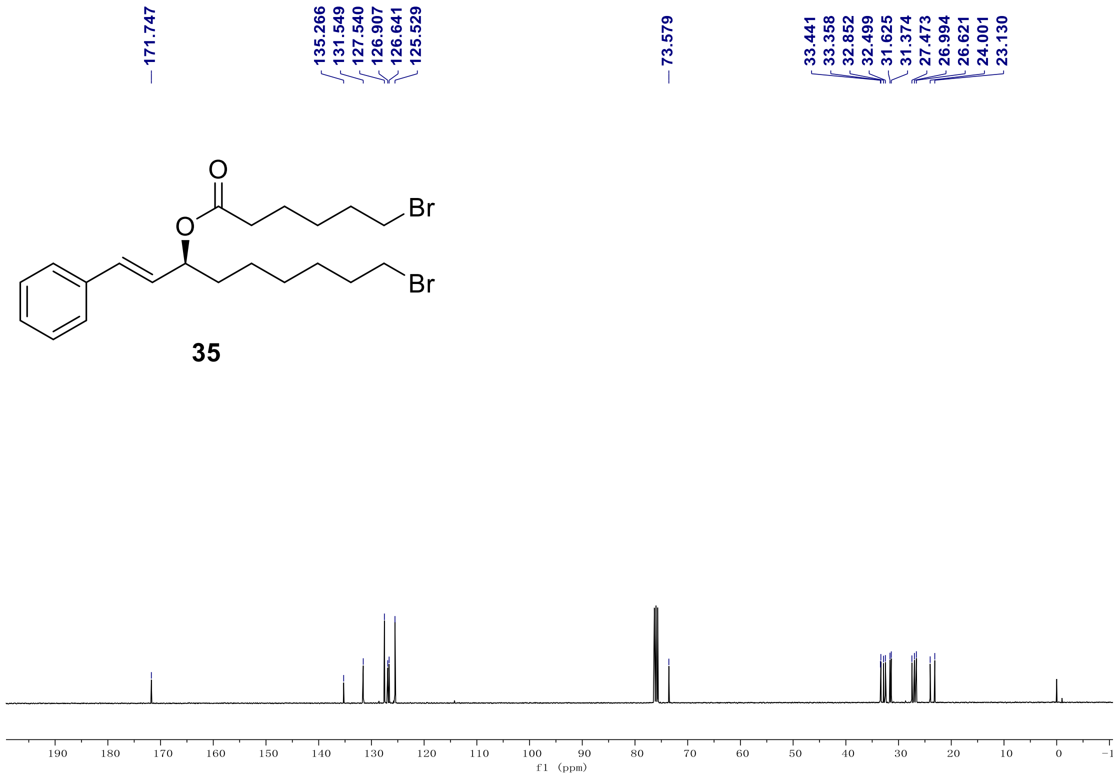

Supplementary Figure 158. ^{13}C NMR spectra of compound **35**

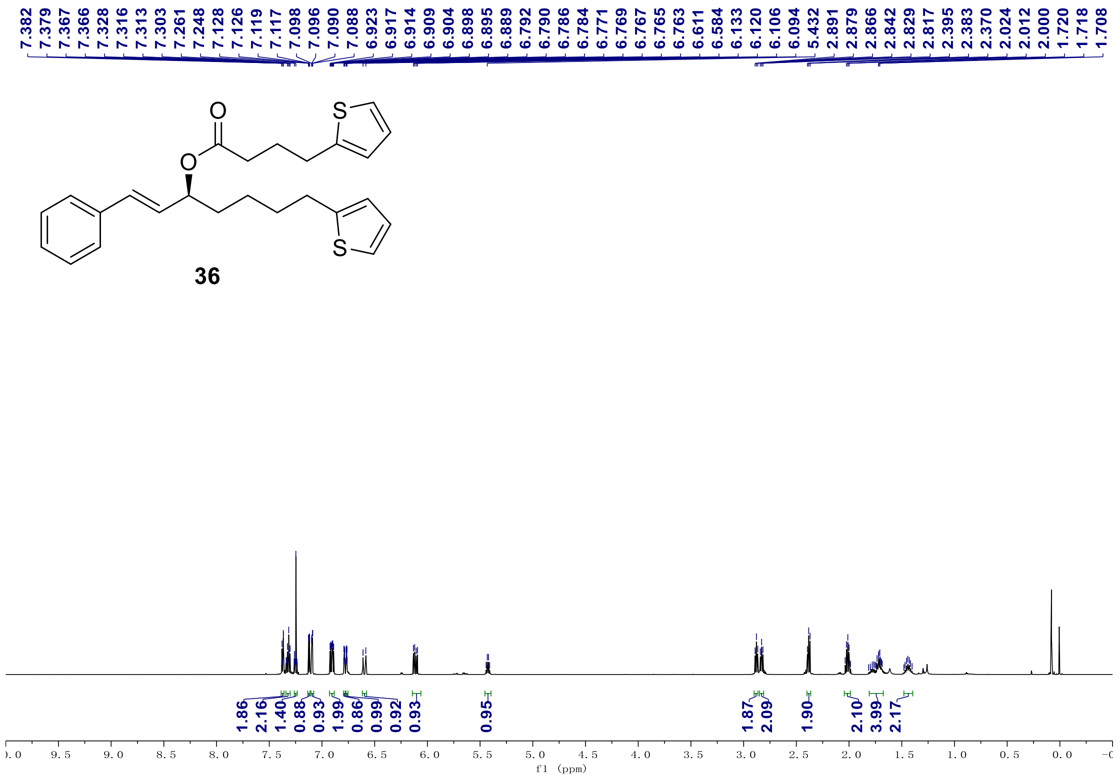

Supplementary Figure 159. ^{1}H NMR spectra of compound **36**

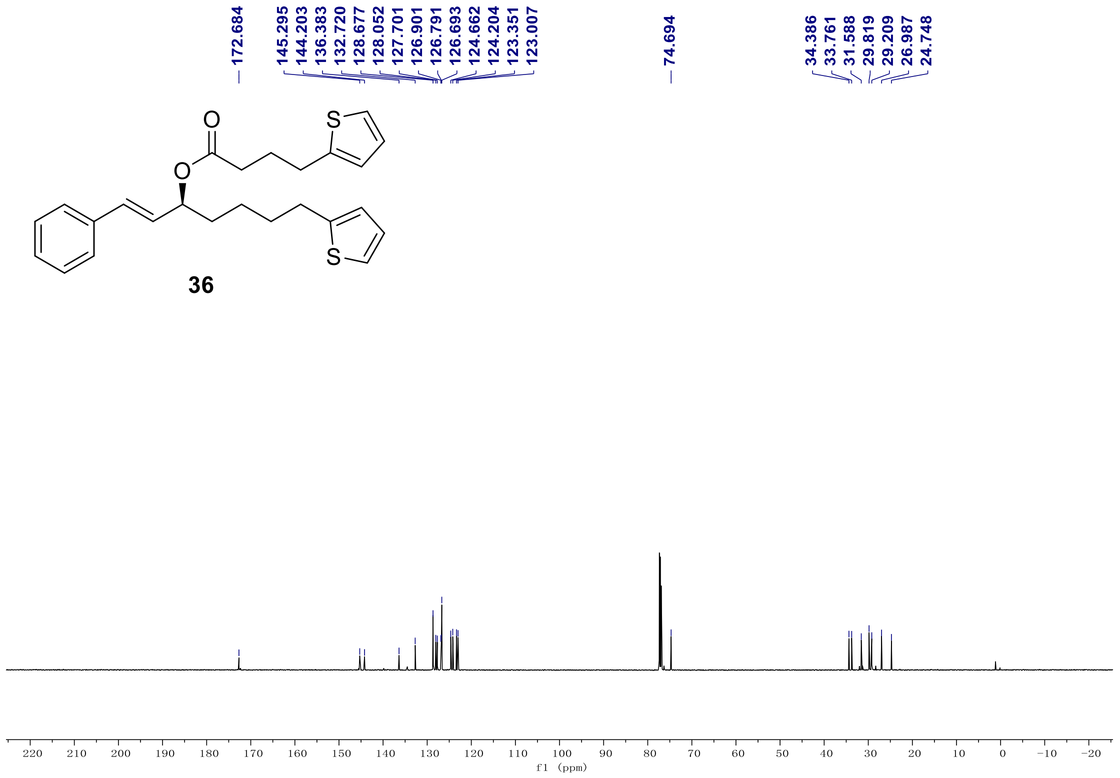

Supplementary Figure 160. ^{13}C NMR spectra of compound **36**

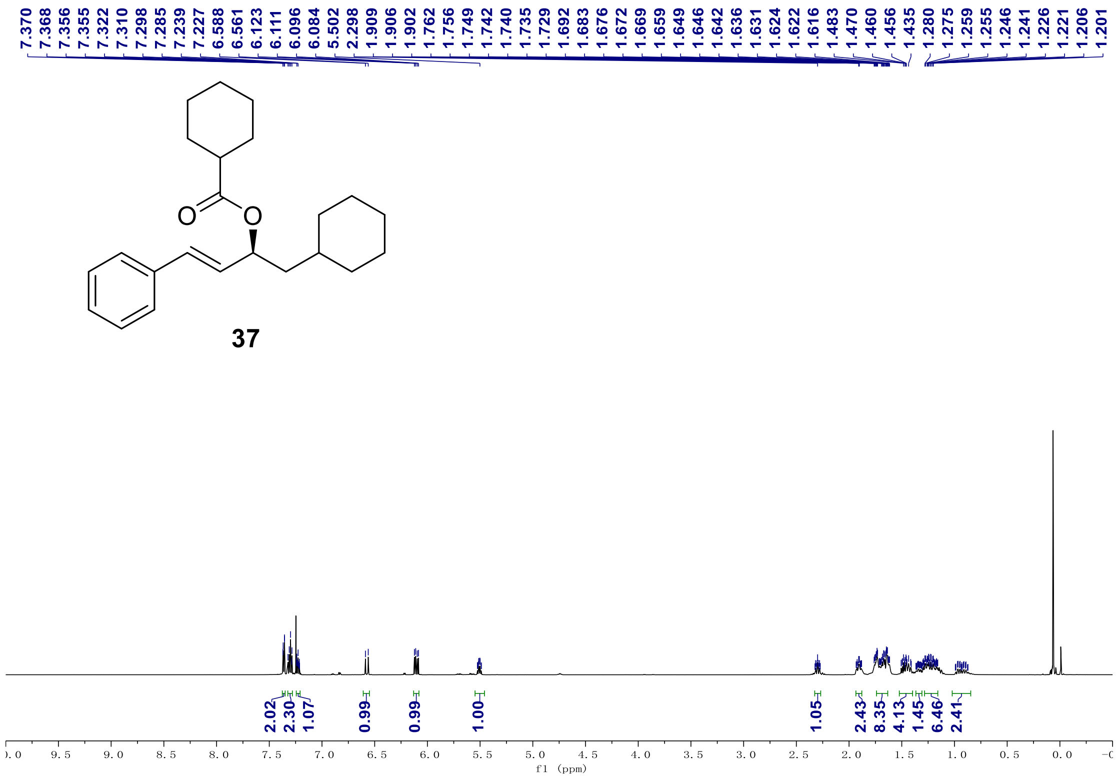

Supplementary Figure 161. ^{1}H NMR spectra of compound **37**

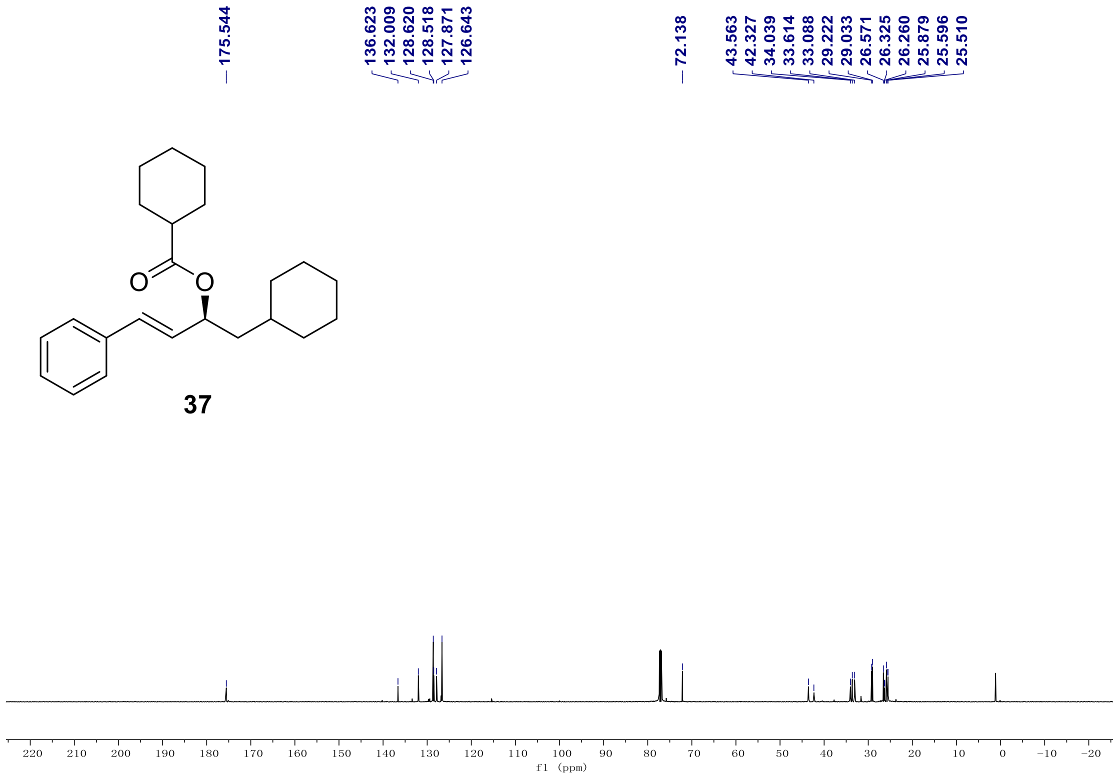

Supplementary Figure 162. ^{13}C NMR spectra of compound **37**

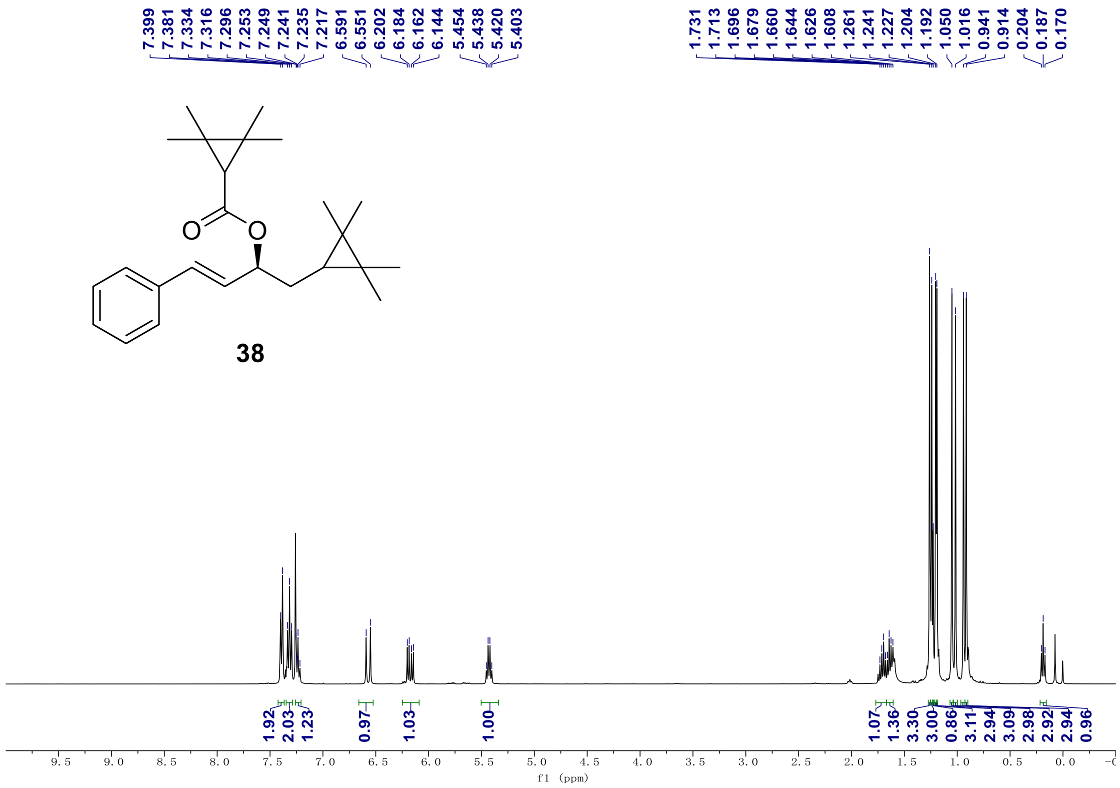

Supplementary Figure 163. ^{1}H NMR spectra of compound **38**

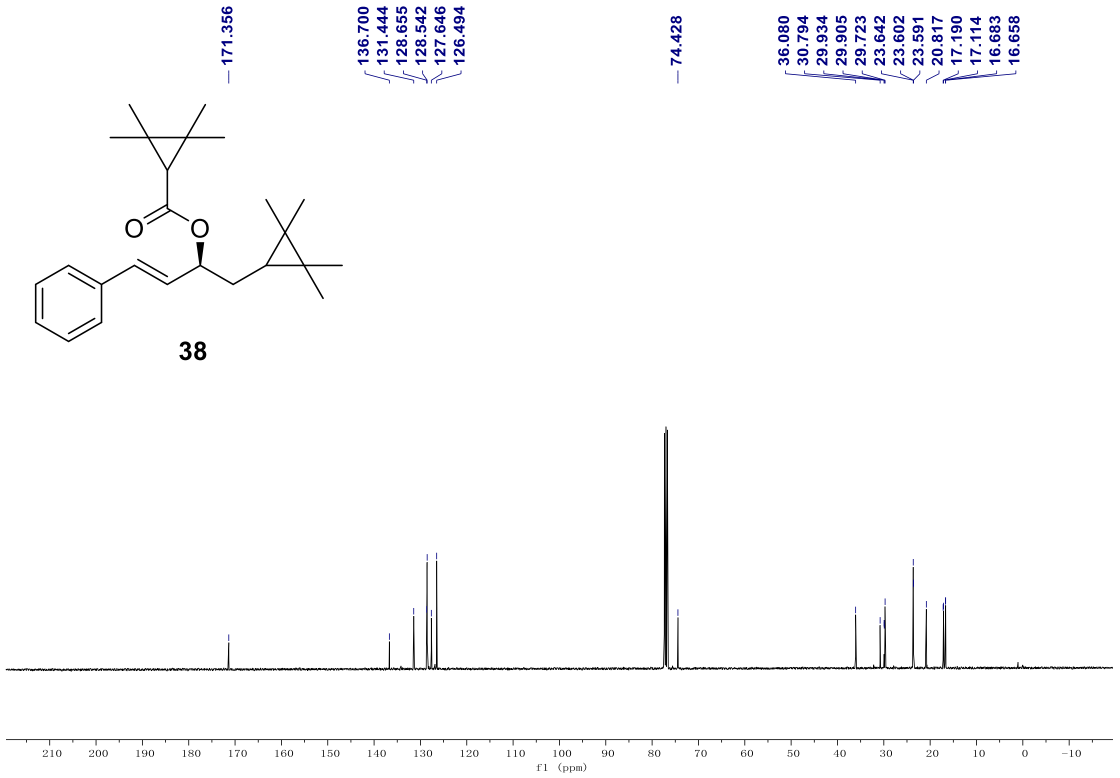

Supplementary Figure 164. ^{13}C NMR spectra of compound **38**

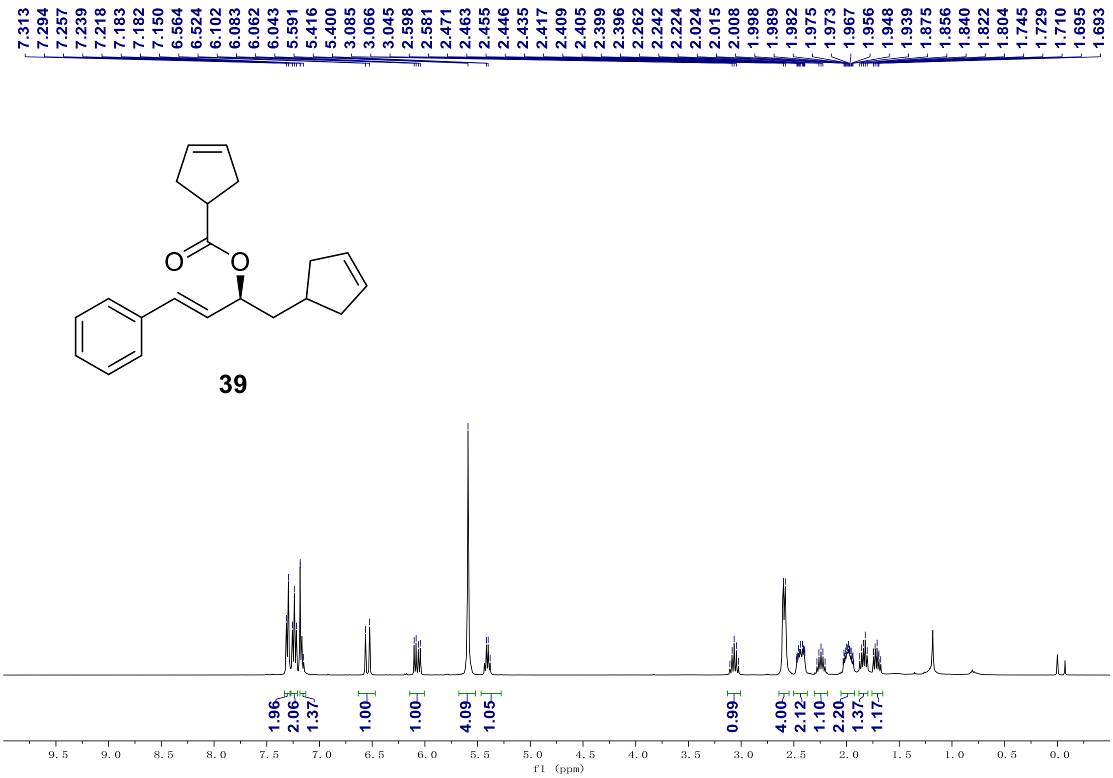

Supplementary Figure 165. ^{1}H NMR spectra of compound **39**

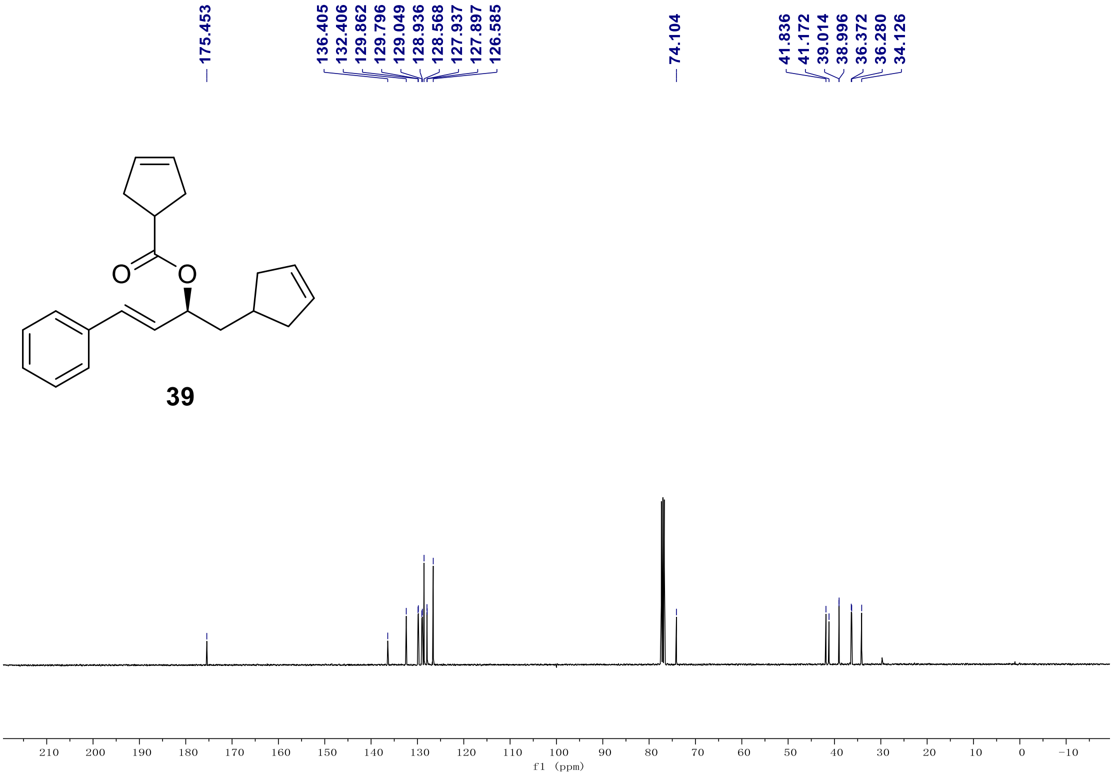

Supplementary Figure 166. ^{13}C NMR spectra of compound **39**

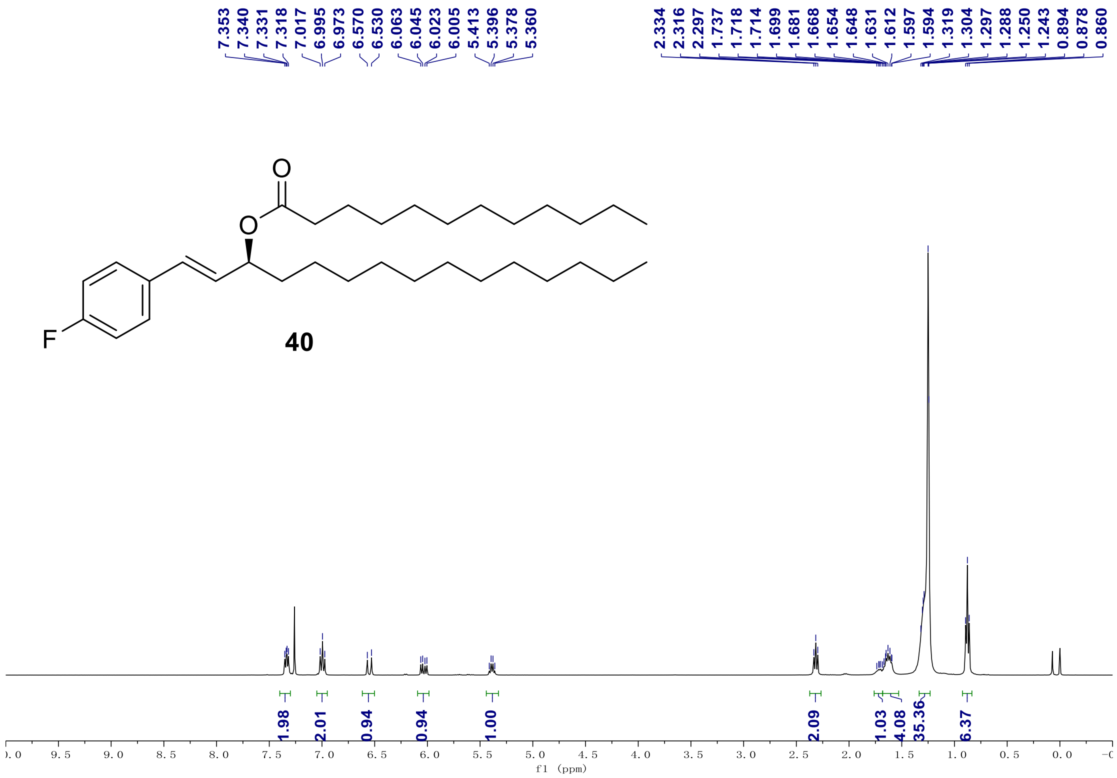

Supplementary Figure 167. ^{1}H NMR spectra of compound **40**

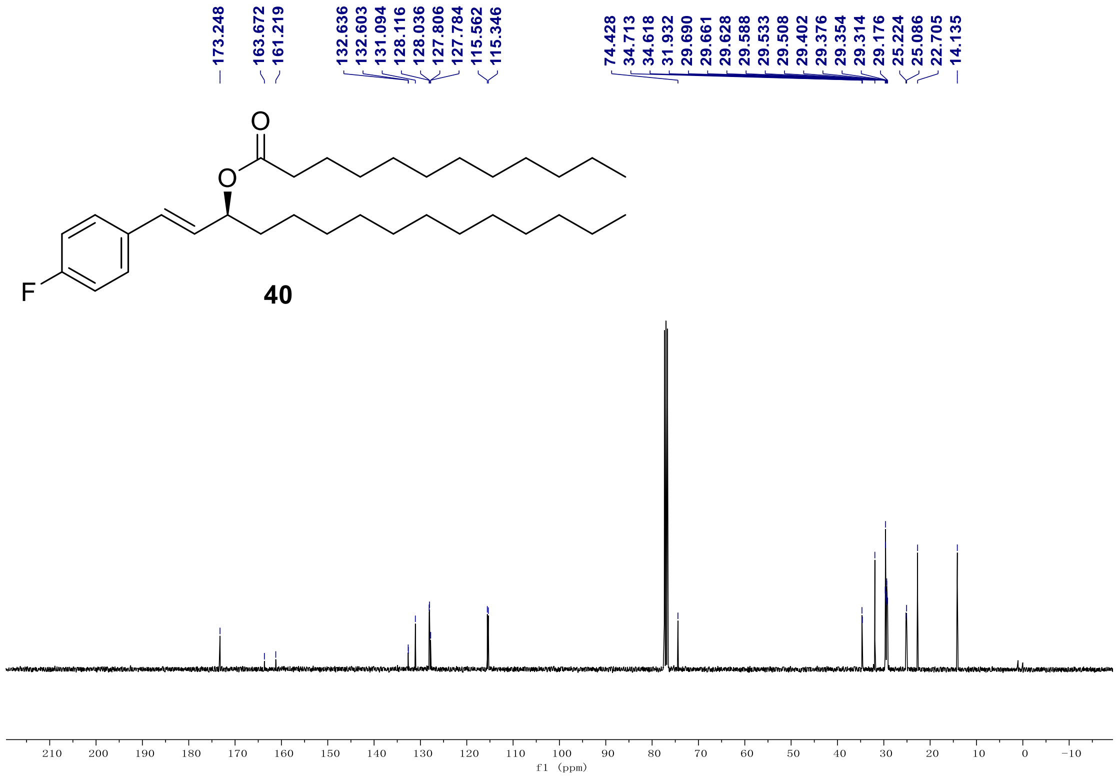

Supplementary Figure 168. ^{13}C NMR spectra of compound **40**

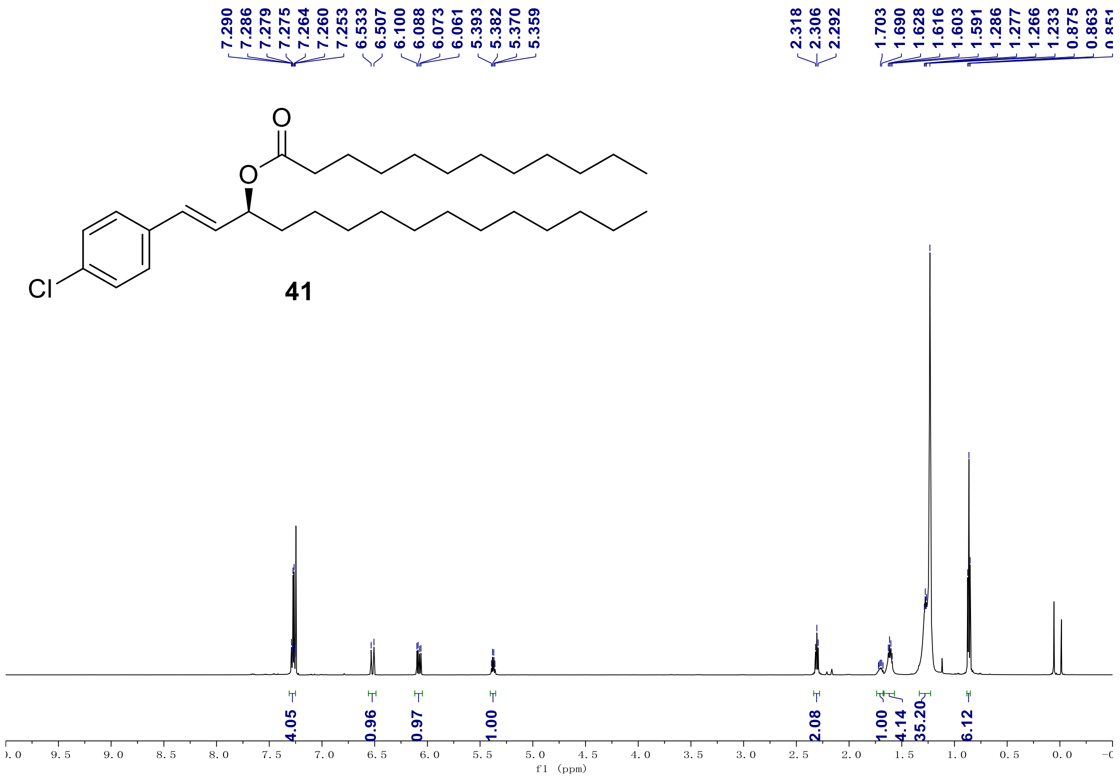

Supplementary Figure 169. ^{1}H NMR spectra of compound **41**

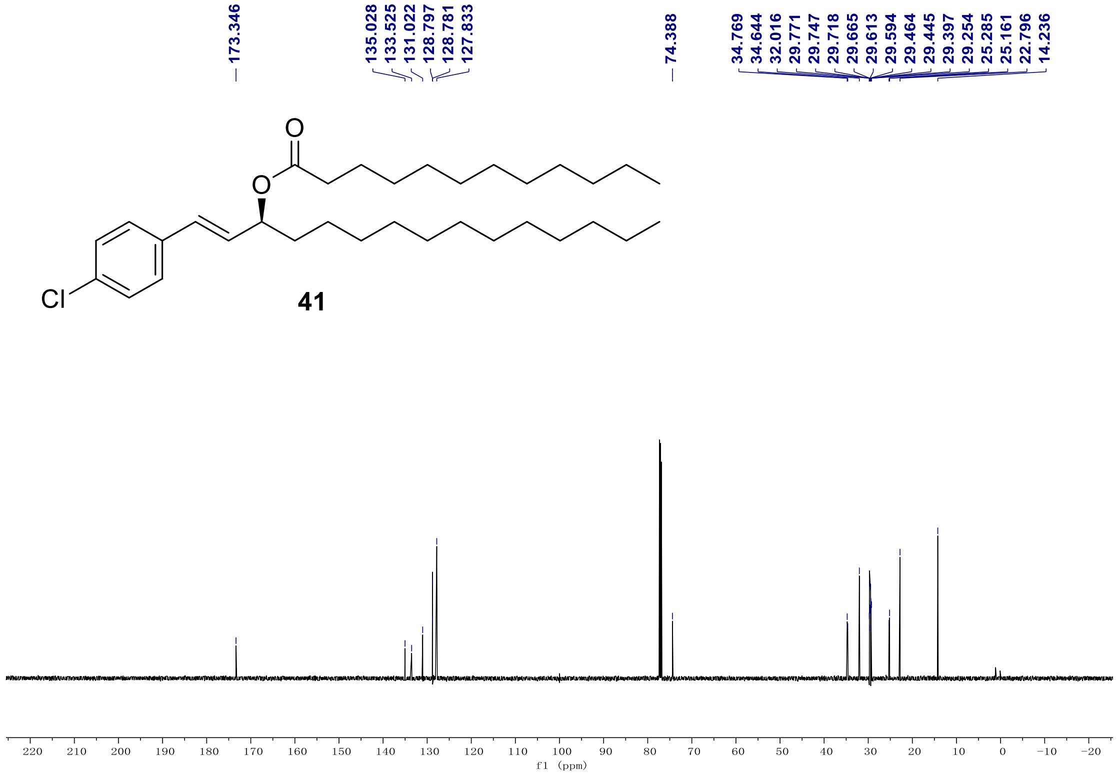

Supplementary Figure 170. ^{13}C NMR spectra of compound **41**

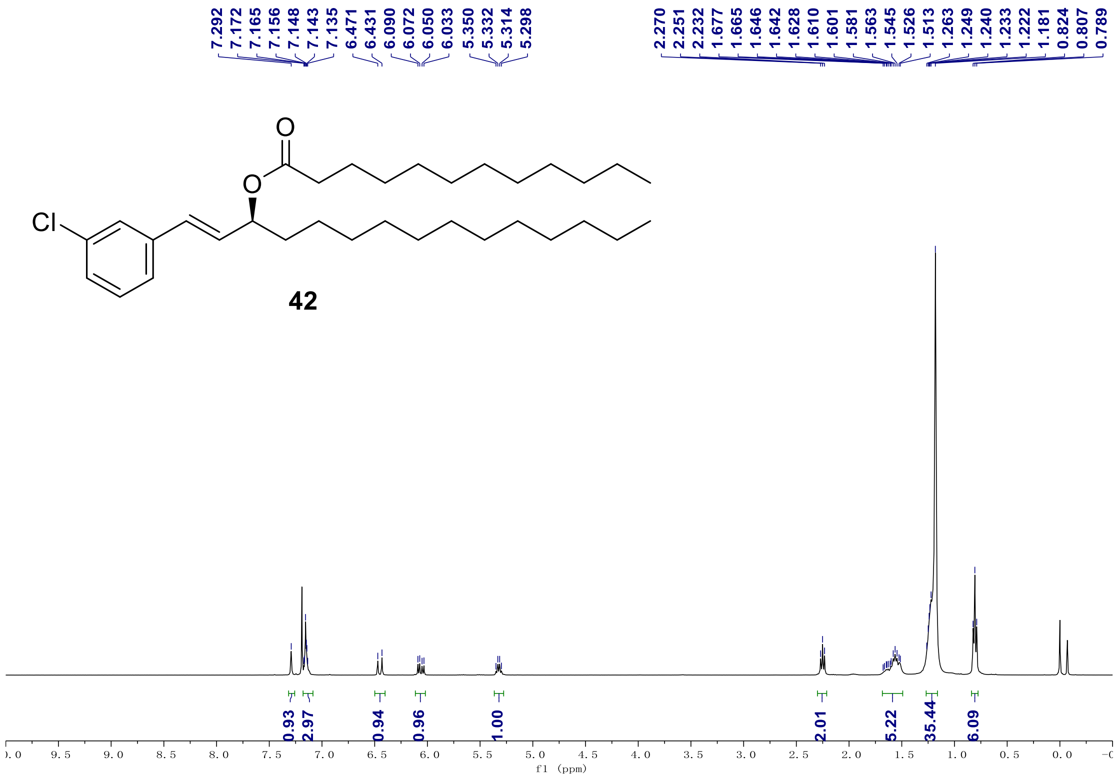

Supplementary Figure 171. ^{1}H NMR spectra of compound **42**

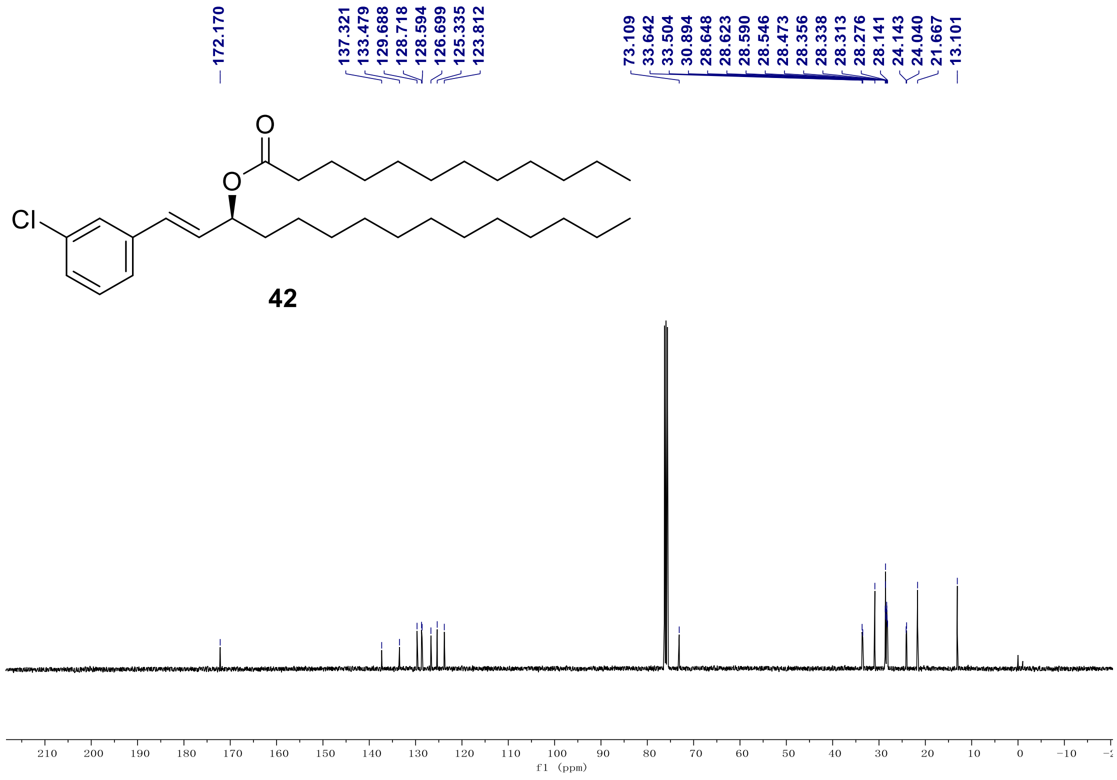

Supplementary Figure 172. ^{13}C NMR spectra of compound **42**

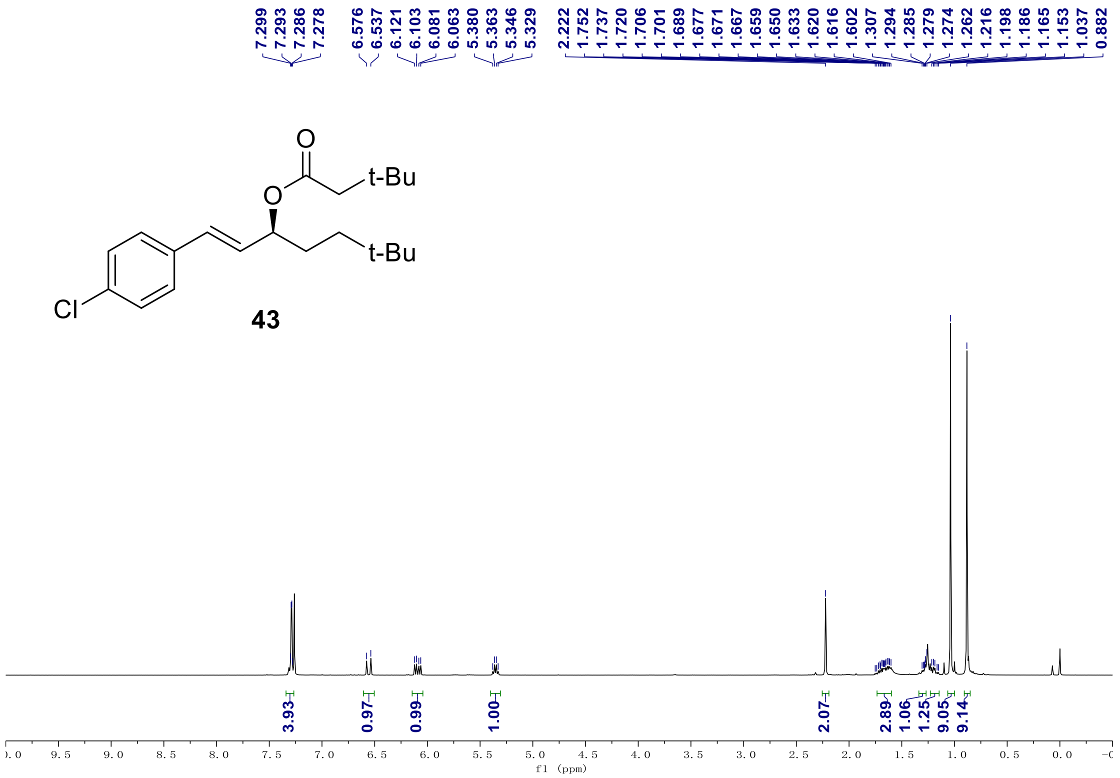

Supplementary Figure 173. ^{1}H NMR spectra of compound **43**

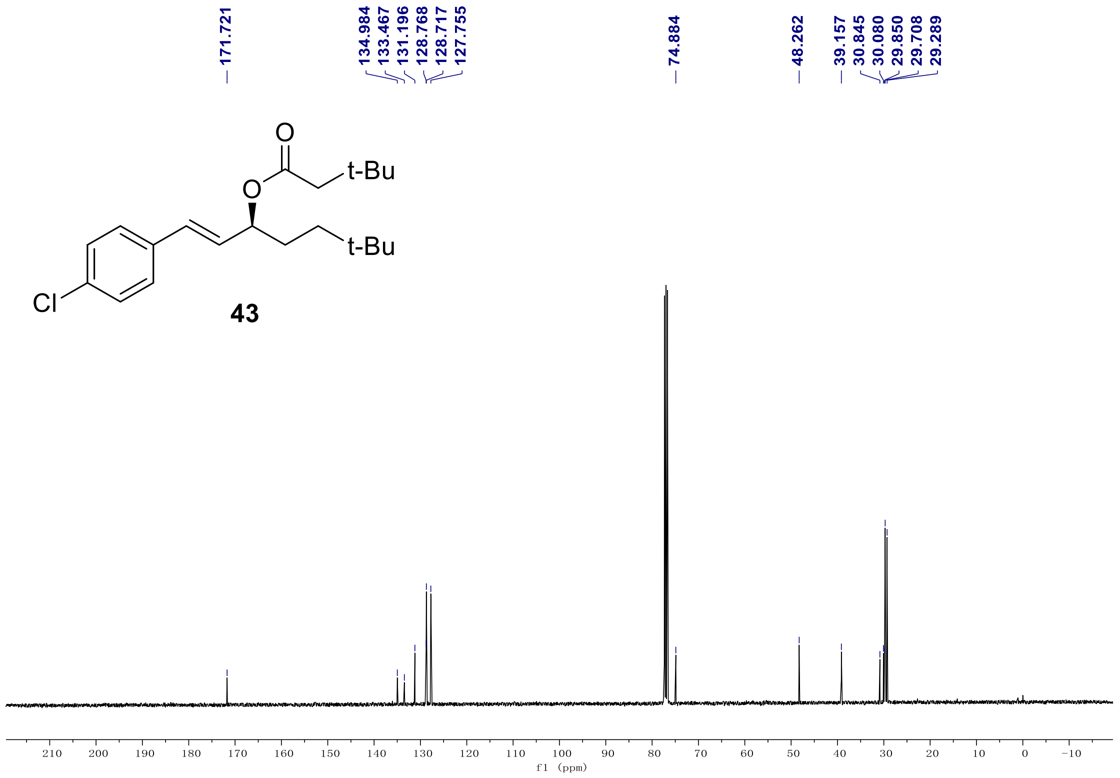

Supplementary Figure 174. ^{13}C NMR spectra of compound **43**

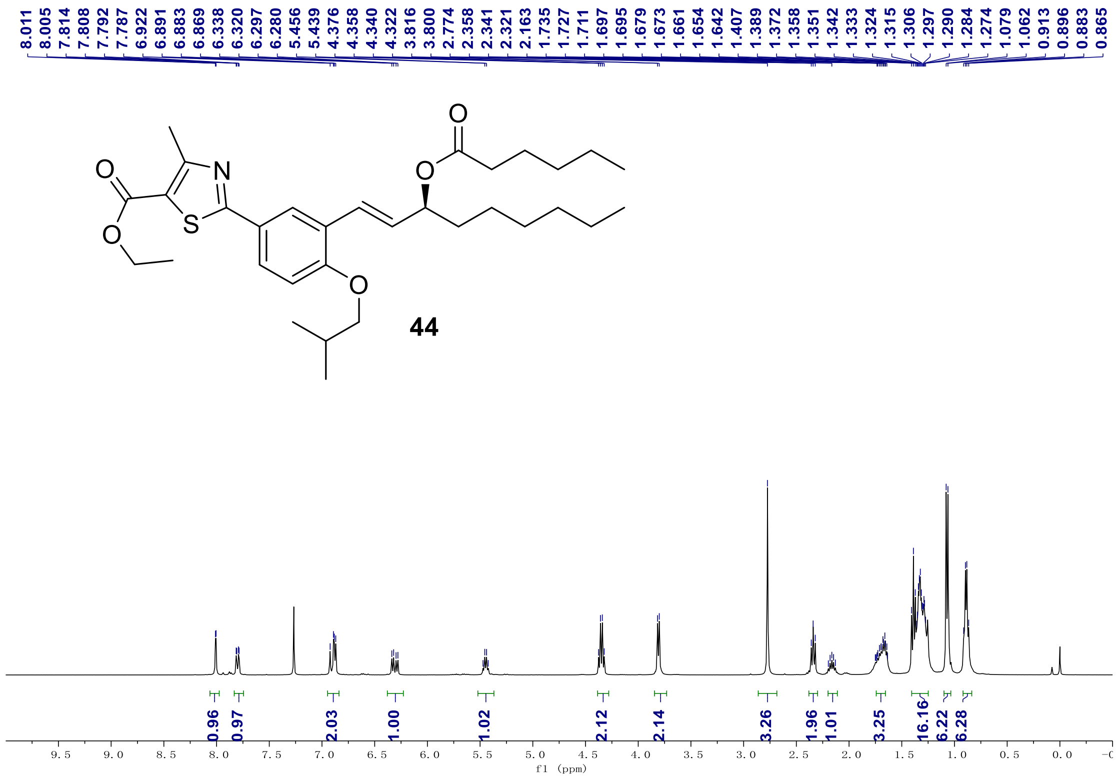

Supplementary Figure 175. ^{1}H NMR spectra of compound **44**

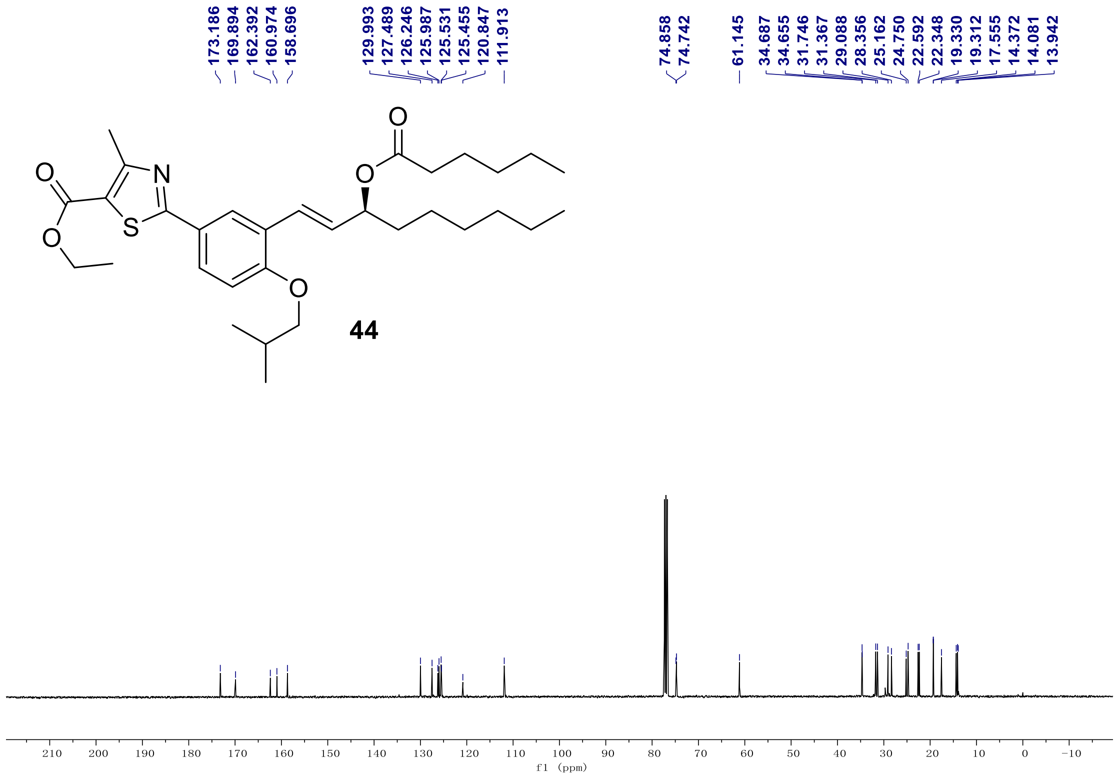

Supplementary Figure 176. ^{13}C NMR spectra of compound **44**

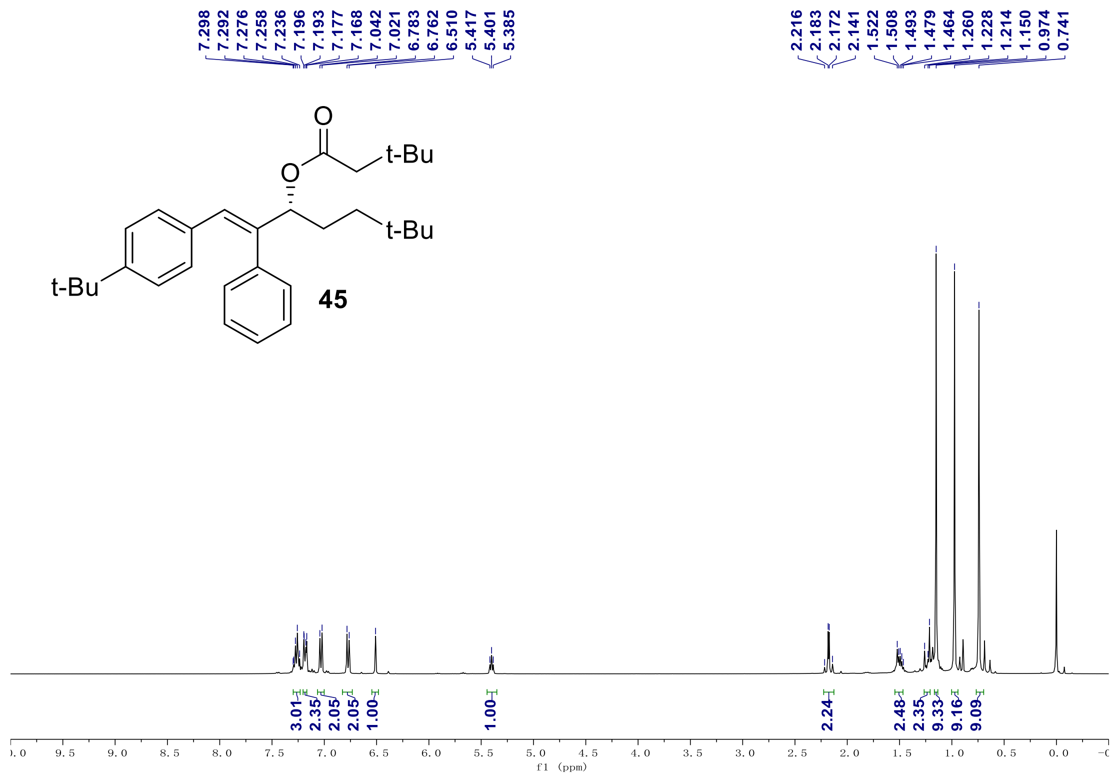

Supplementary Figure 177. ^{1}H NMR spectra of compound **45**

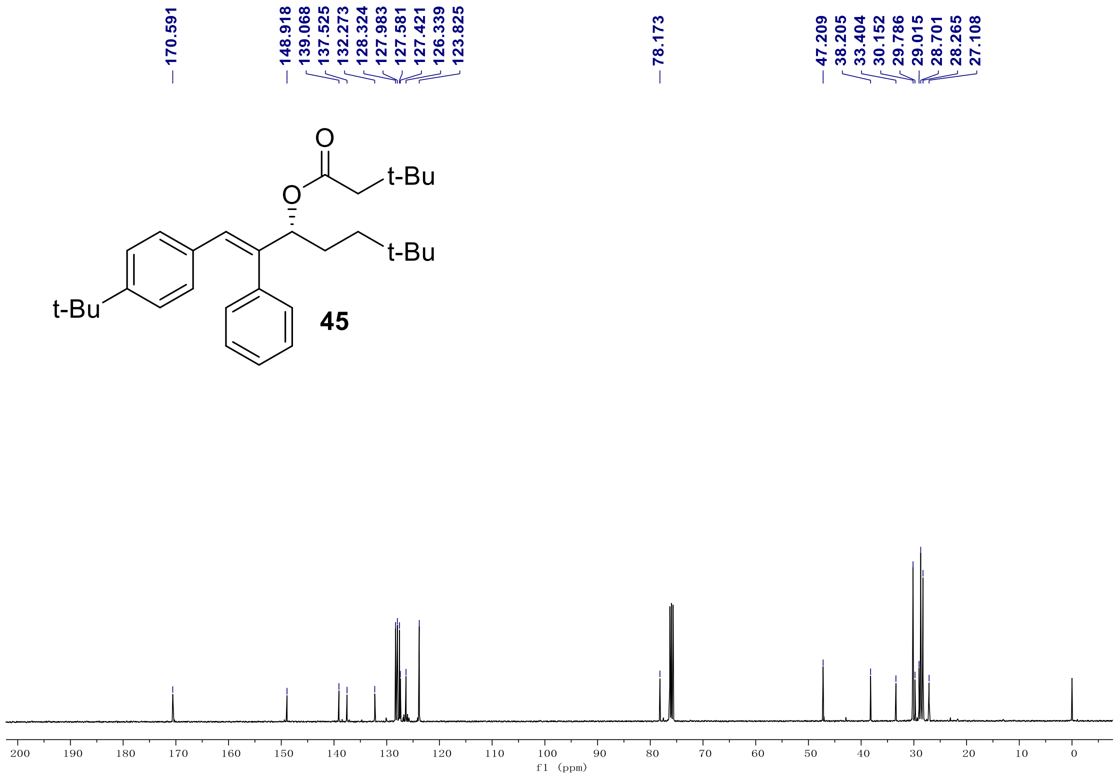

Supplementary Figure 178. ^{13}C NMR spectra of compound **45**

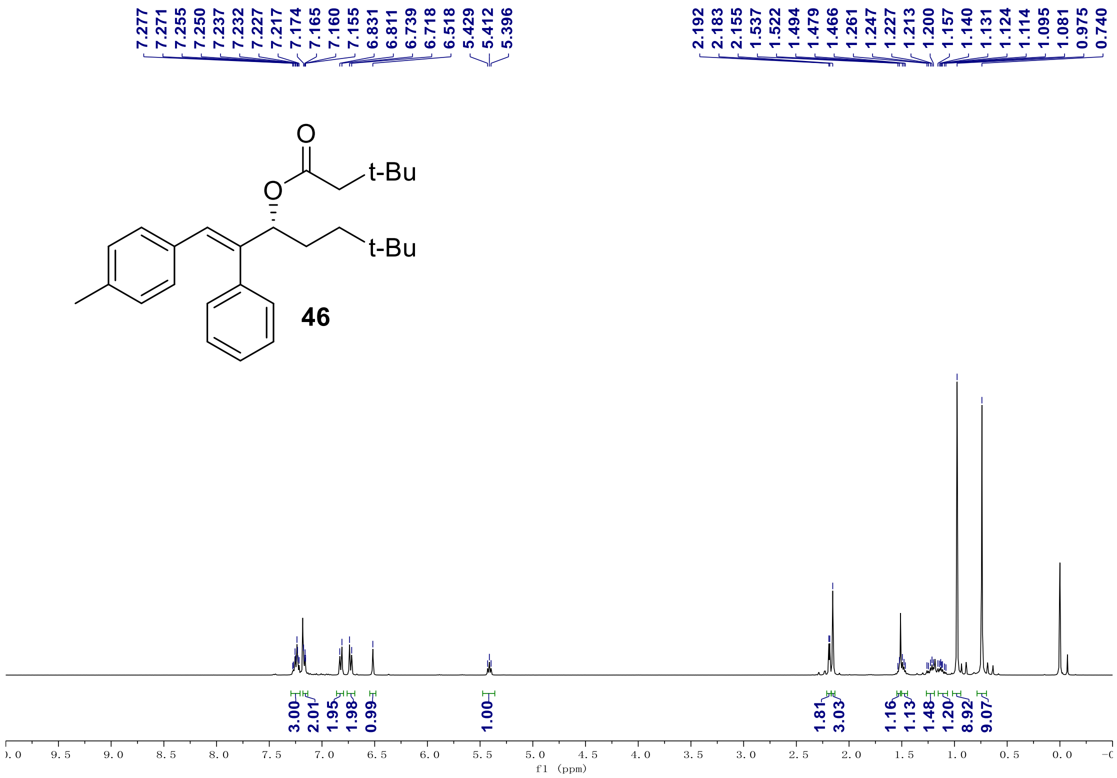

Supplementary Figure 179. ^{1}H NMR spectra of compound **46**

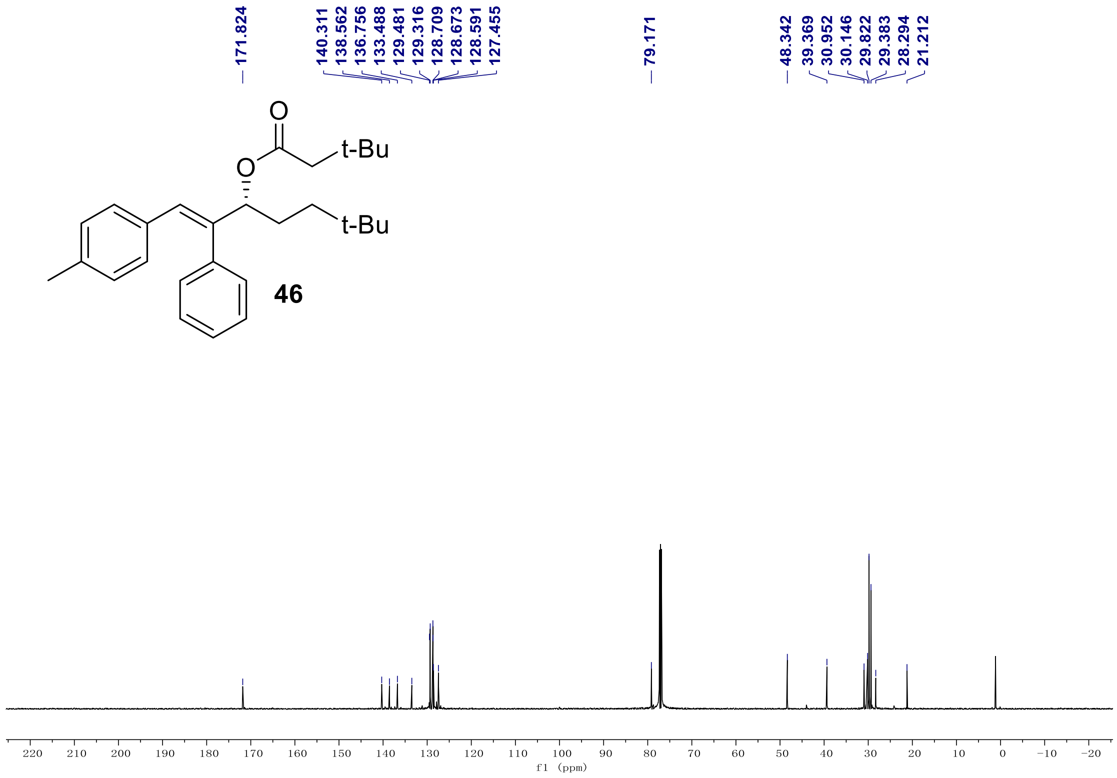

Supplementary Figure 180. ^{13}C NMR spectra of compound **46**

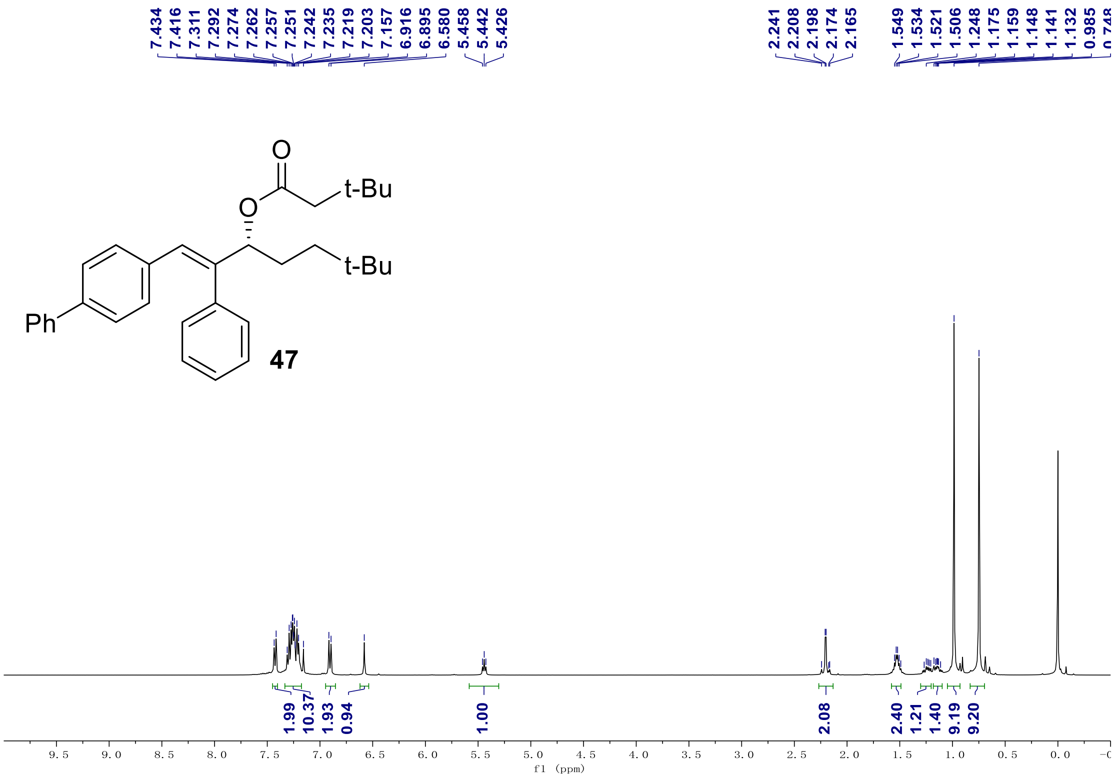

Supplementary Figure 181. ^{1}H NMR spectra of compound **47**

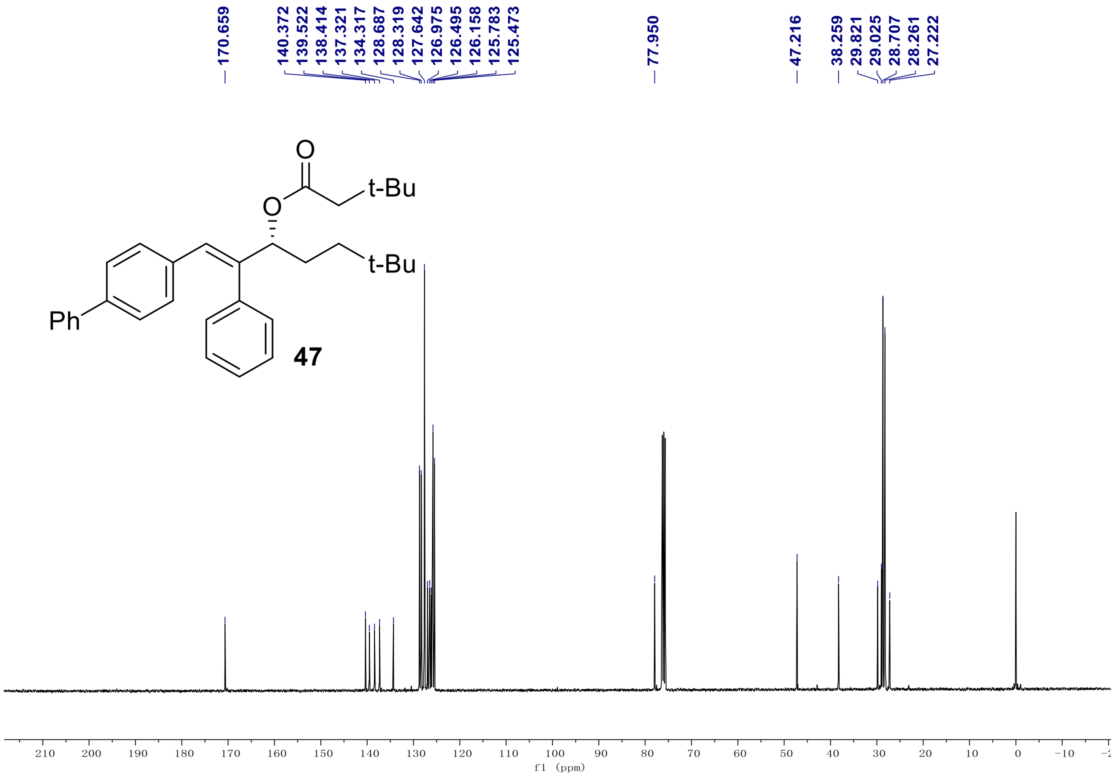

Supplementary Figure 182. ^{13}C NMR spectra of compound **47**

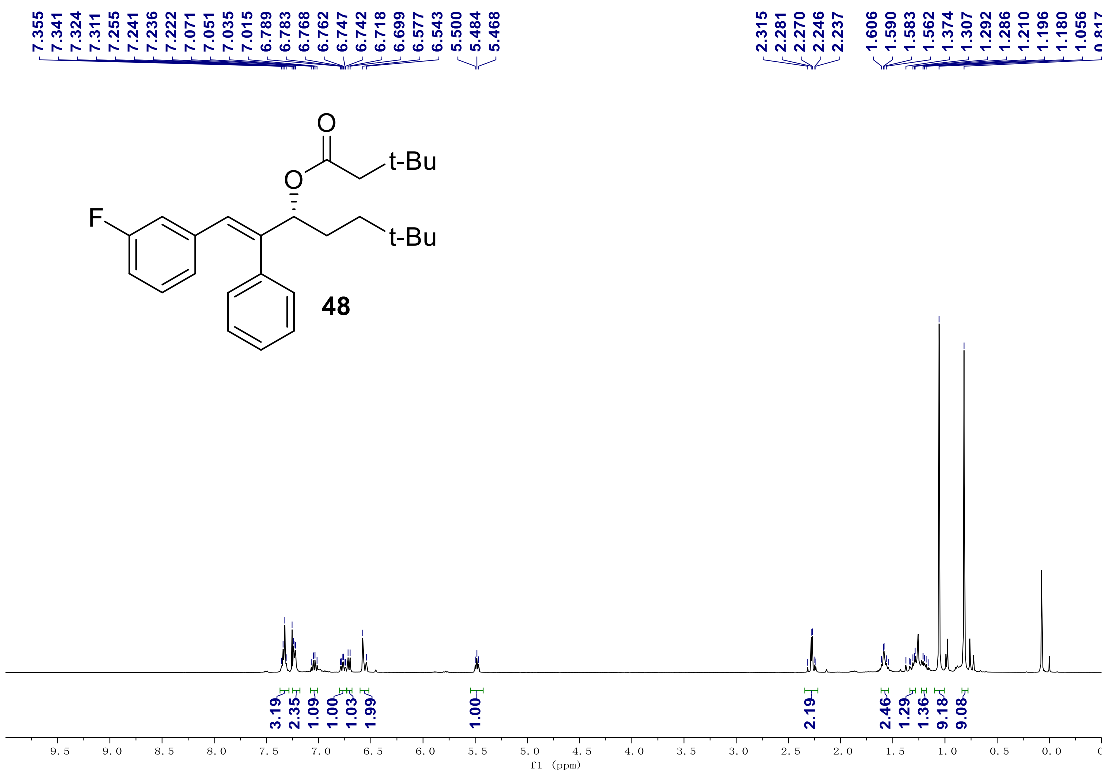

Supplementary Figure 183. ^{1}H NMR spectra of compound **48**

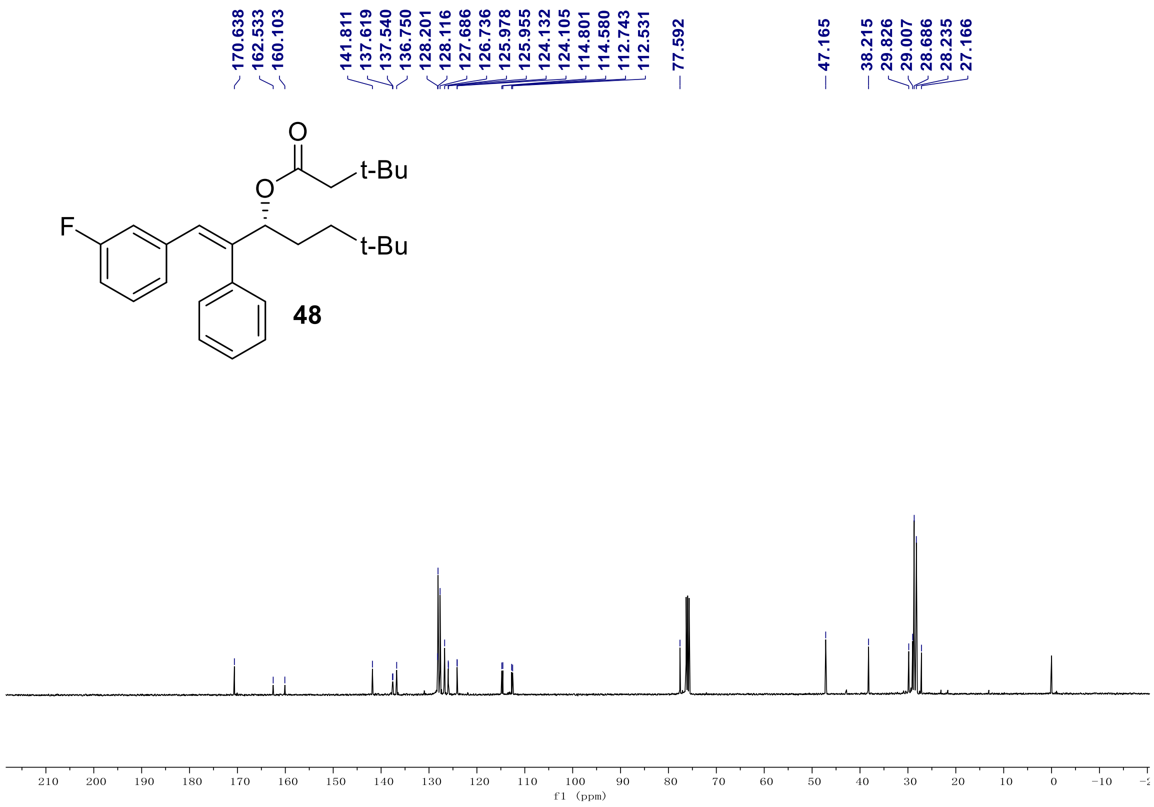

Supplementary Figure 184. ^{13}C NMR spectra of compound **48**

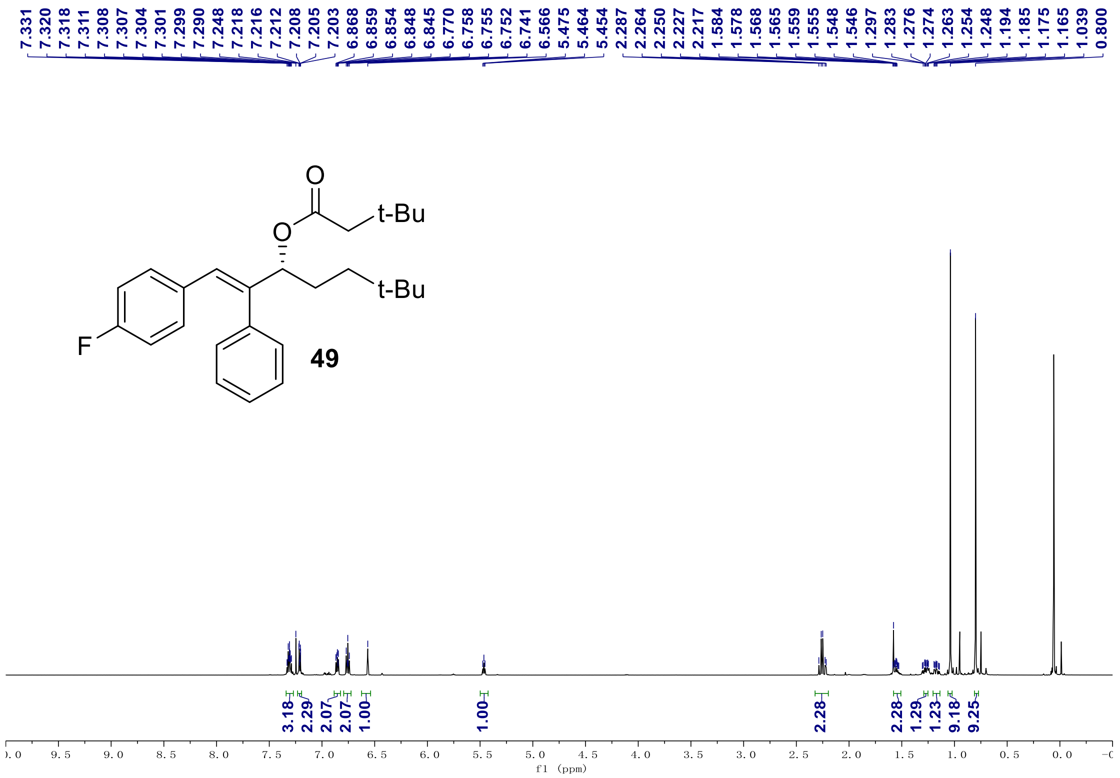

Supplementary Figure 185. ^{1}H NMR spectra of compound **49**

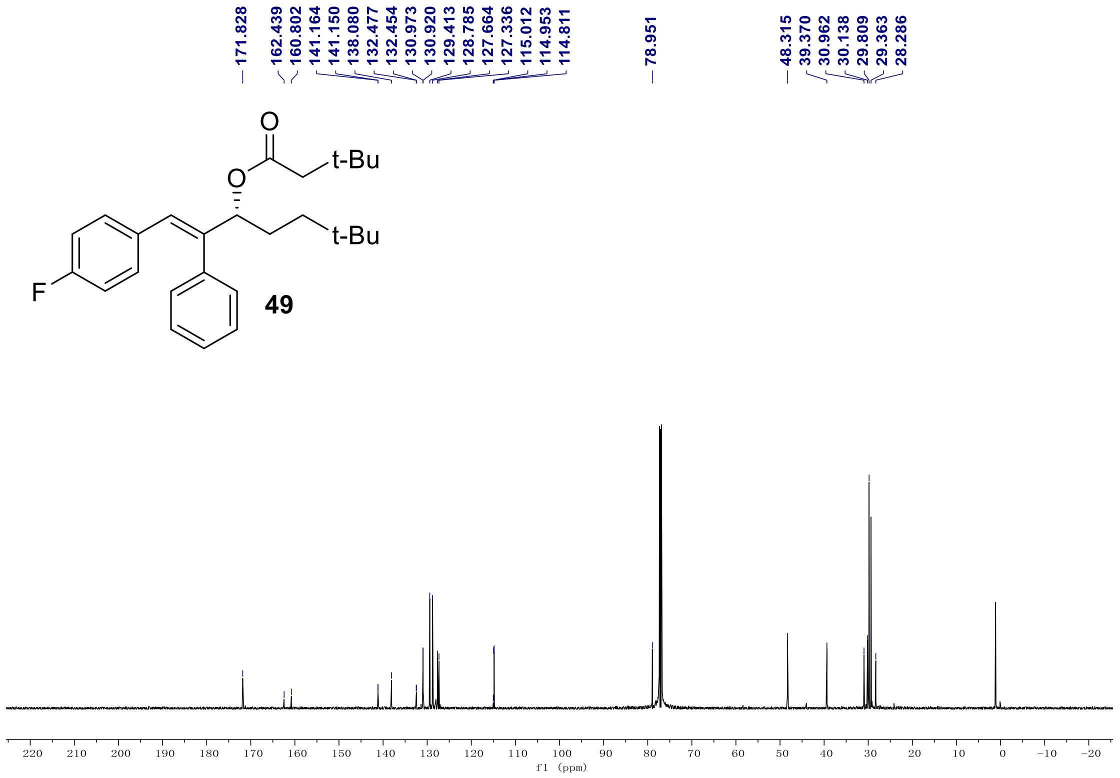

Supplementary Figure 186. ^{13}C NMR spectra of compound **49**

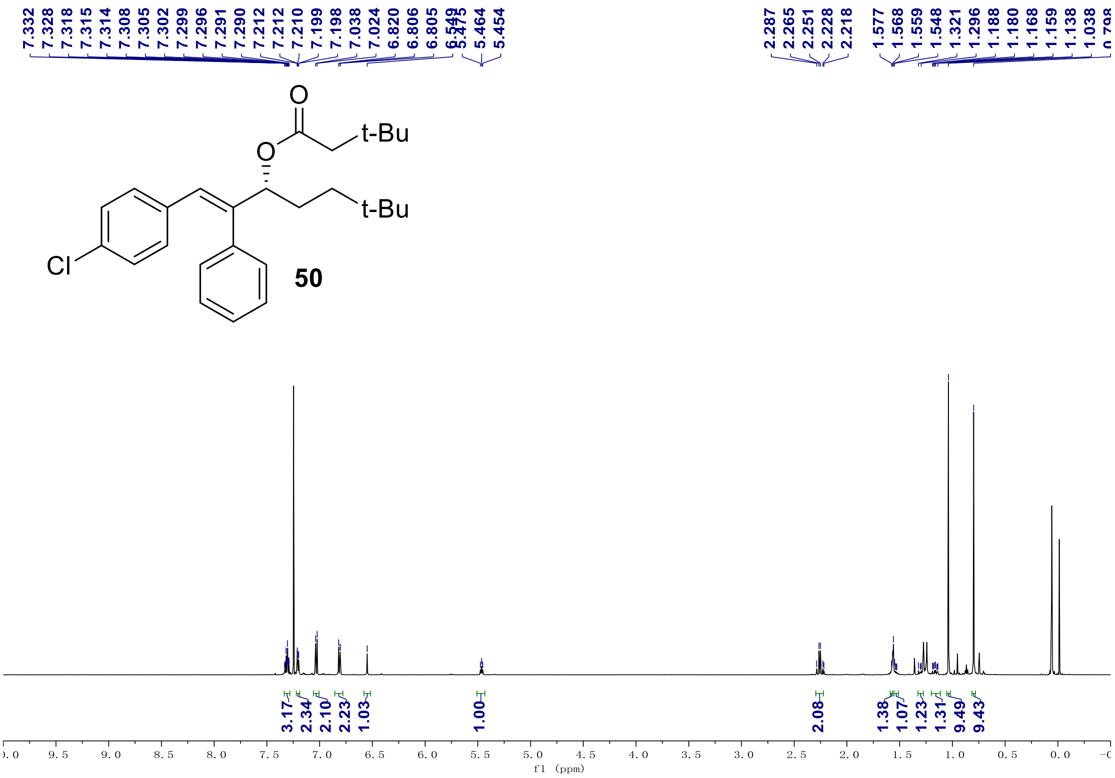

Supplementary Figure 187. ^{1}H NMR spectra of compound **50**

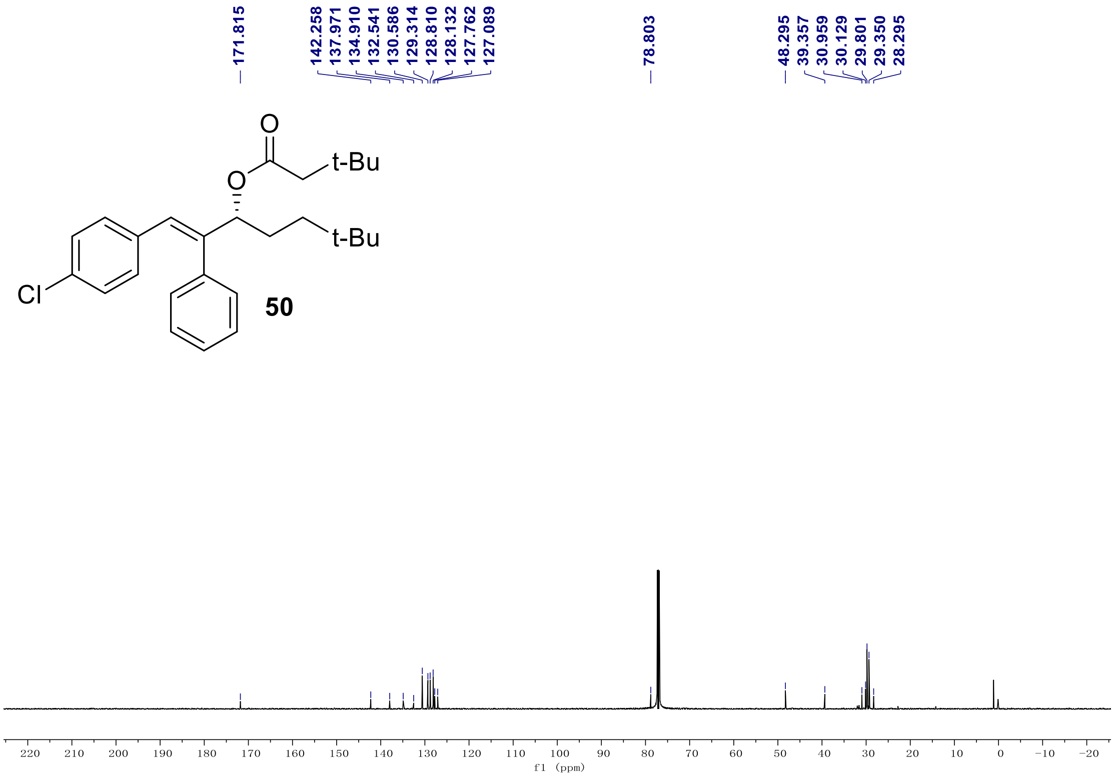

Supplementary Figure 188. ^{13}C NMR spectra of compound **50**

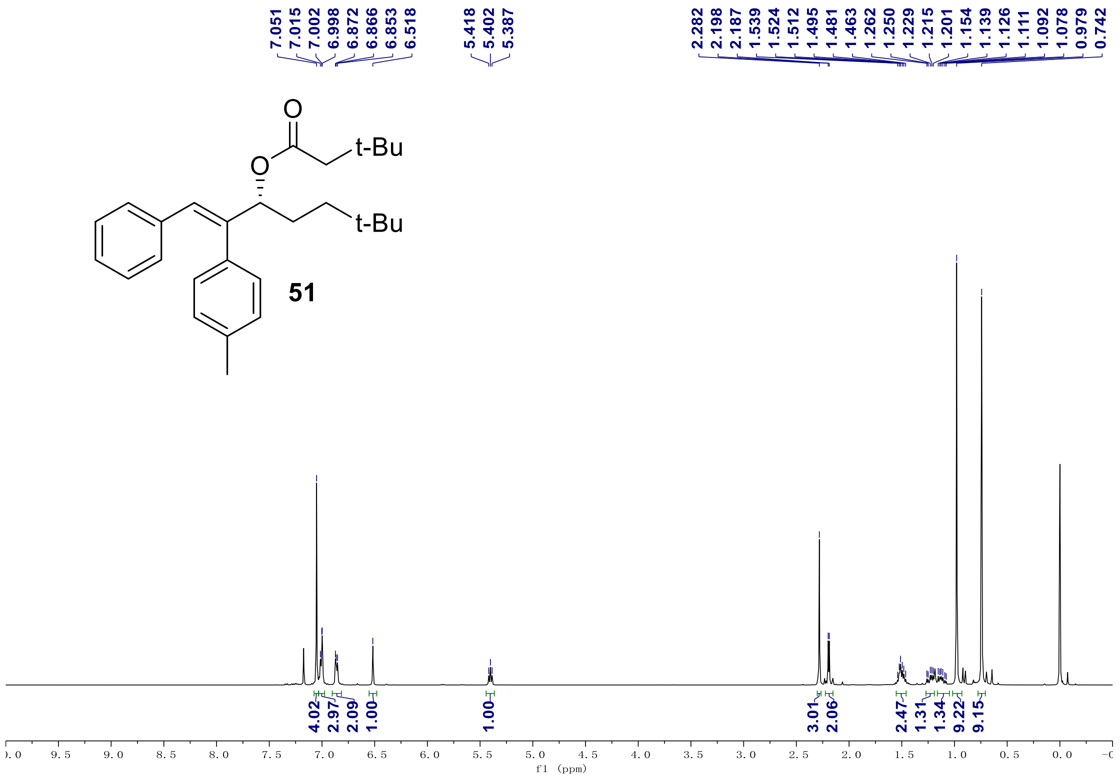

Supplementary Figure 189. ^{1}H NMR spectra of compound **51**

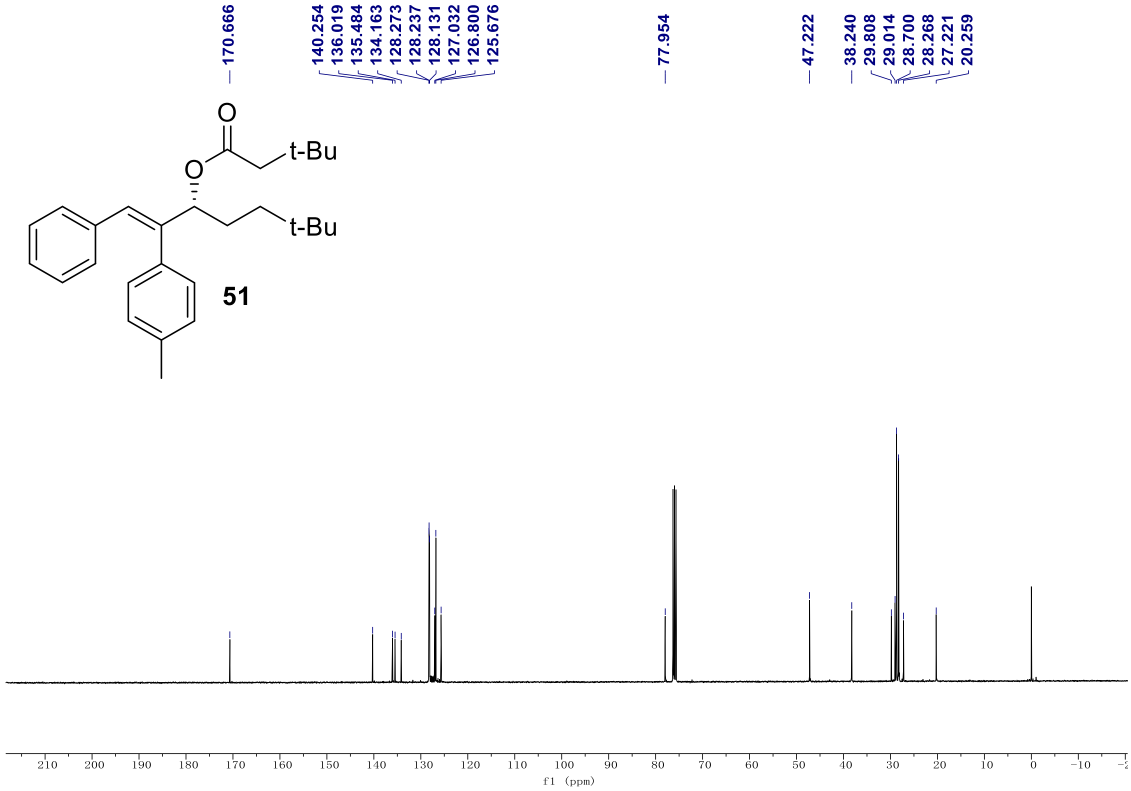

Supplementary Figure 190. ^{13}C NMR spectra of compound **51**

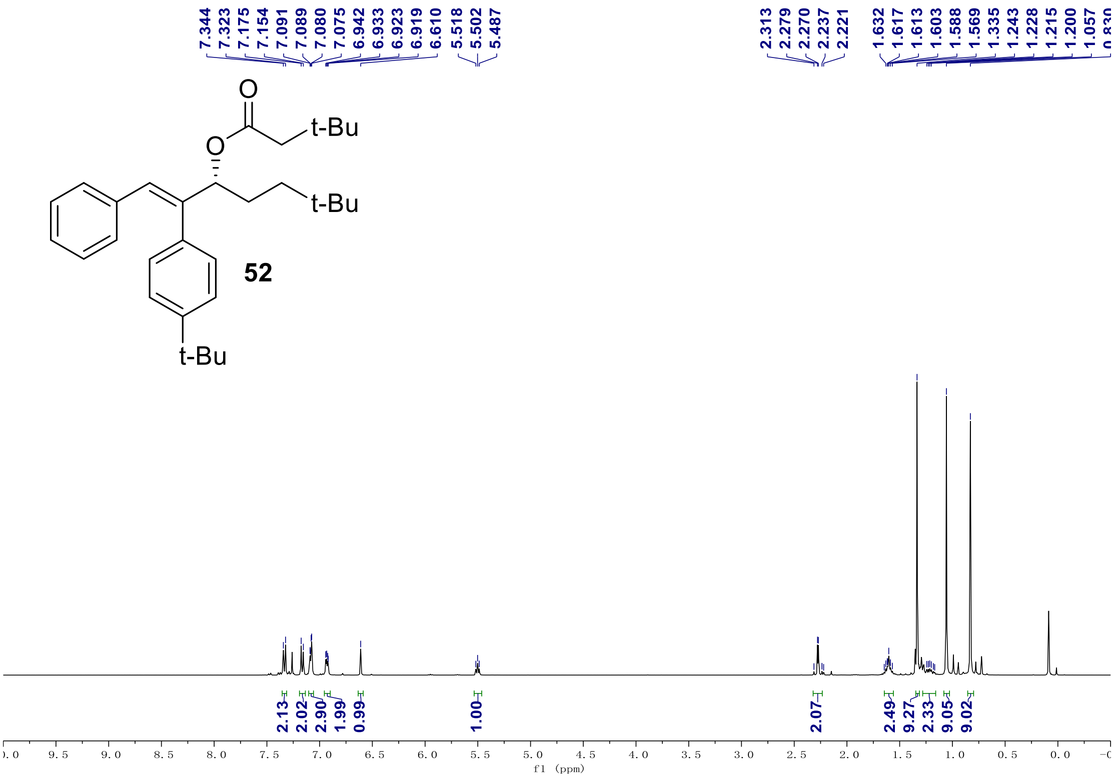

Supplementary Figure 191. ^{1}H NMR spectra of compound **52**

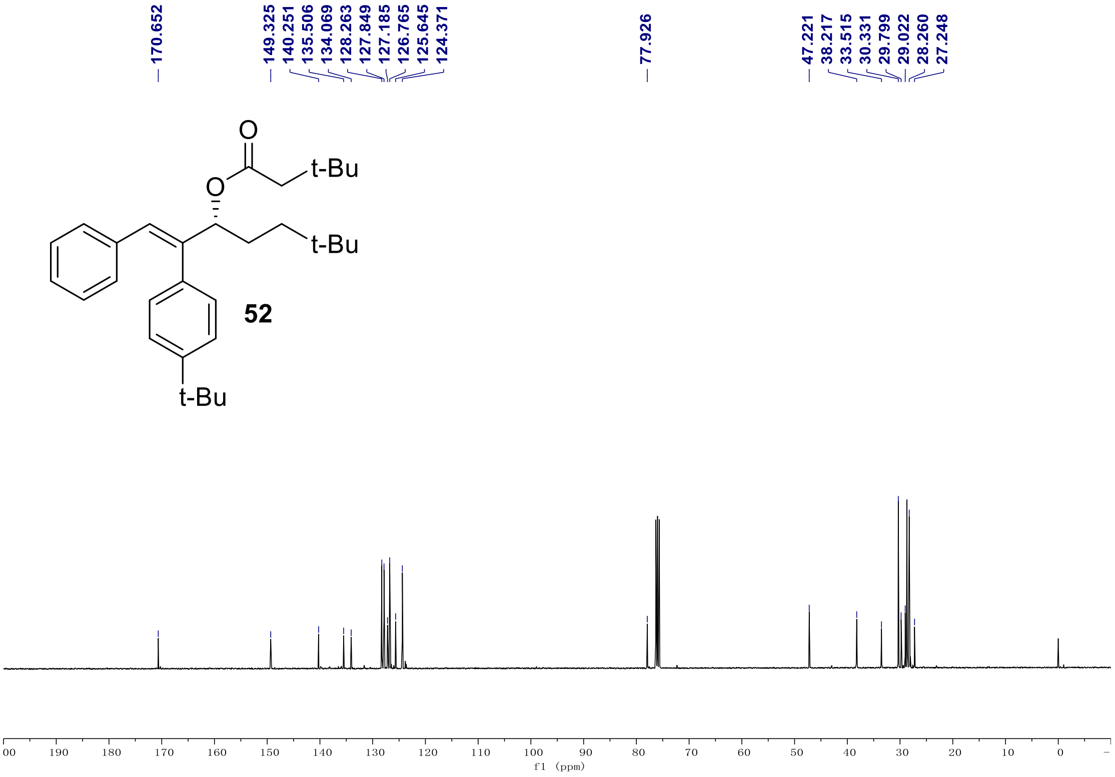

Supplementary Figure 192. ^{13}C NMR spectra of compound **52**

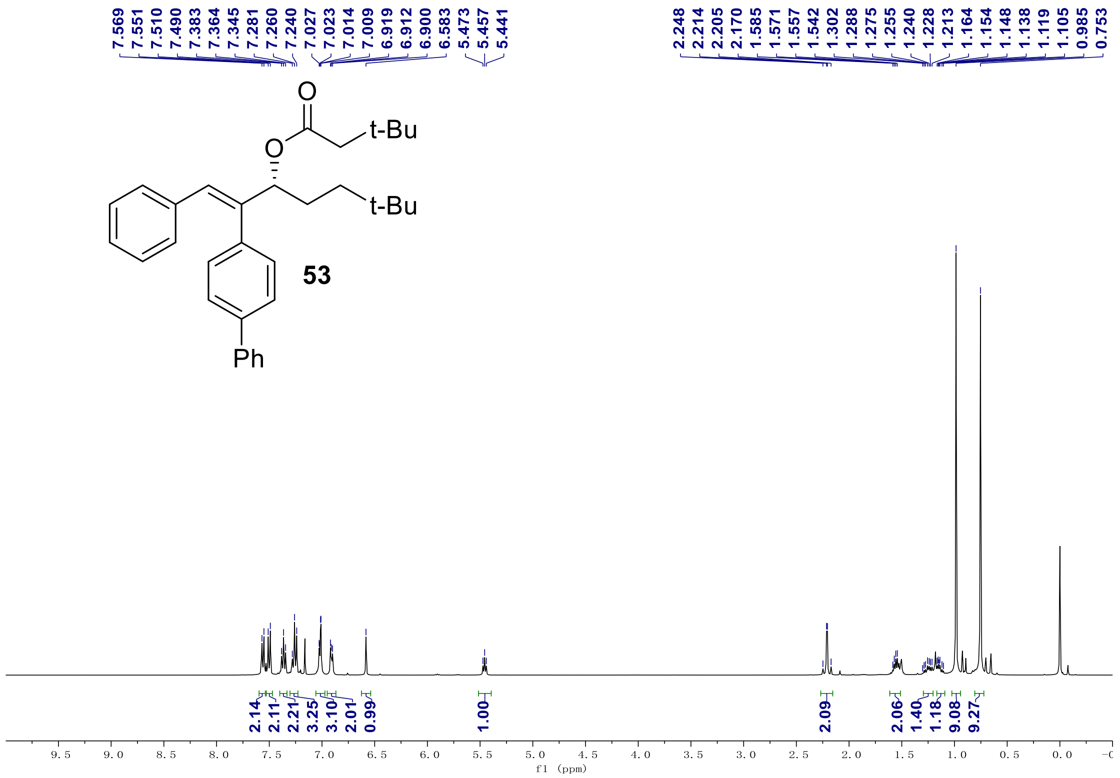

Supplementary Figure 193. ^{1}H NMR spectra of compound **53**

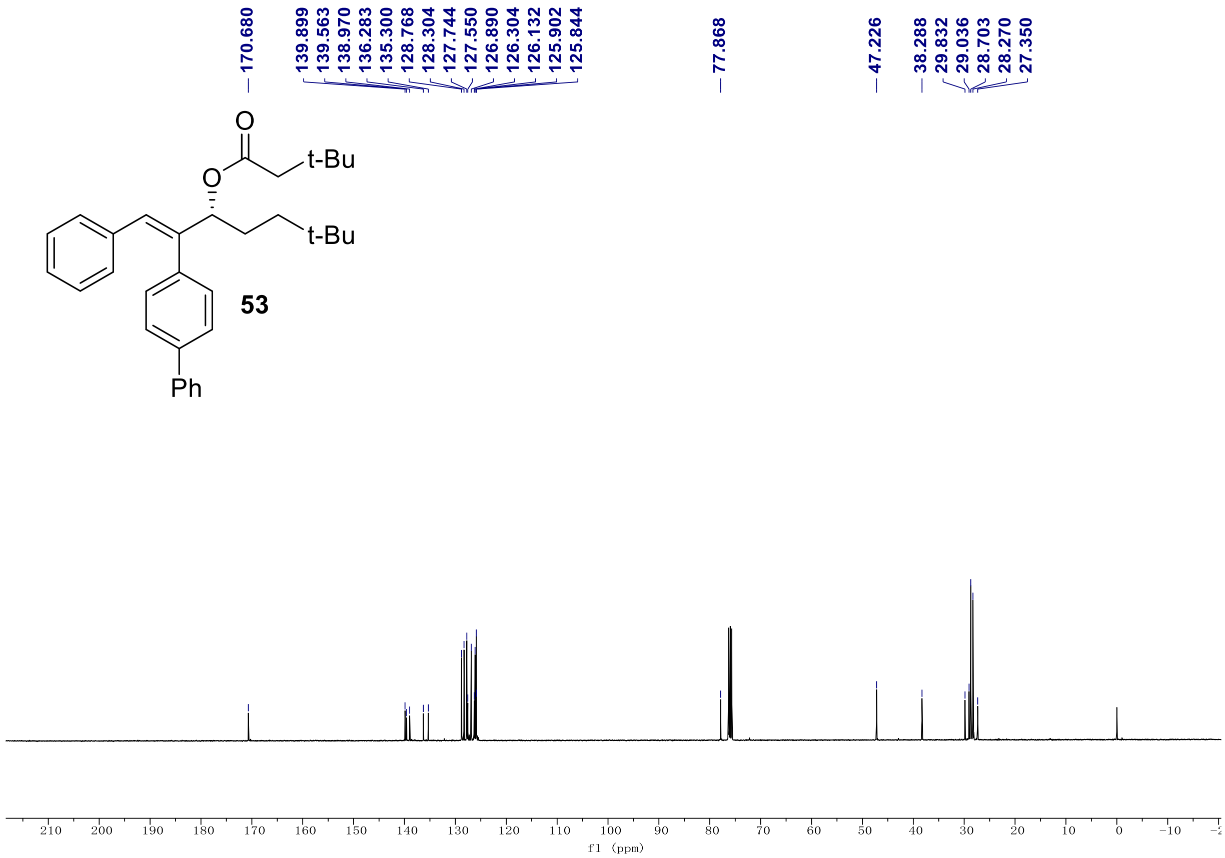

Supplementary Figure 194. ^{13}C NMR spectra of compound **53**

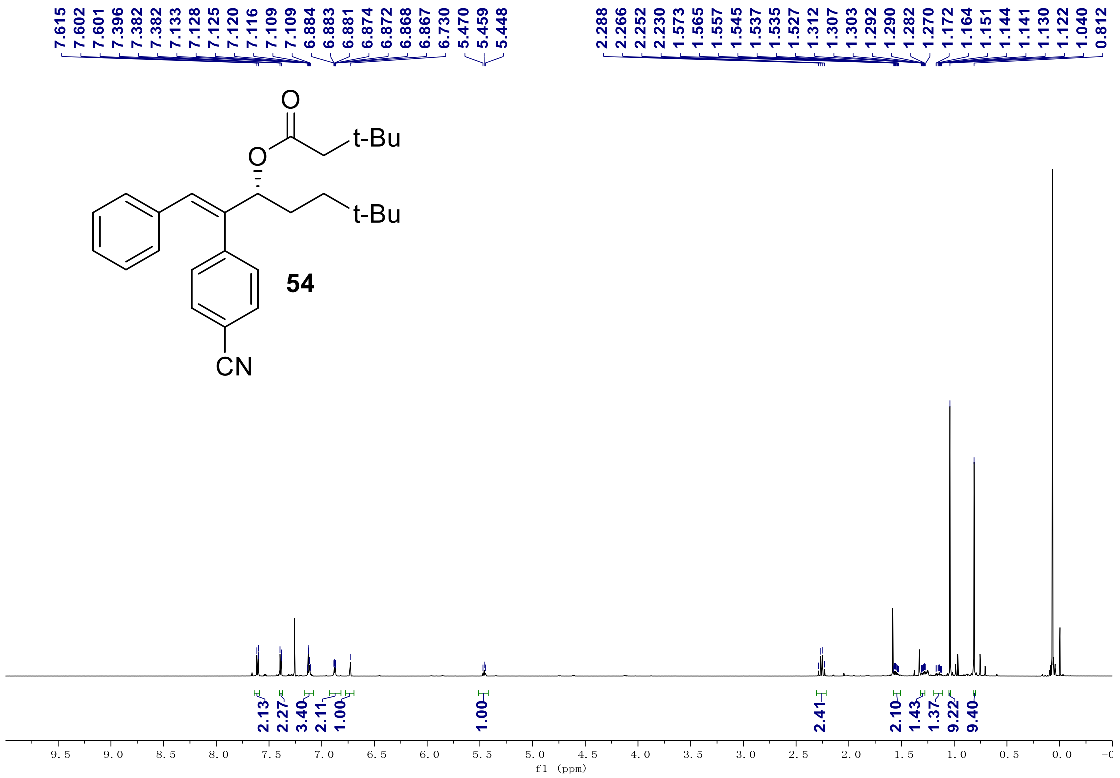

Supplementary Figure 195. ^{1}H NMR spectra of compound **54**

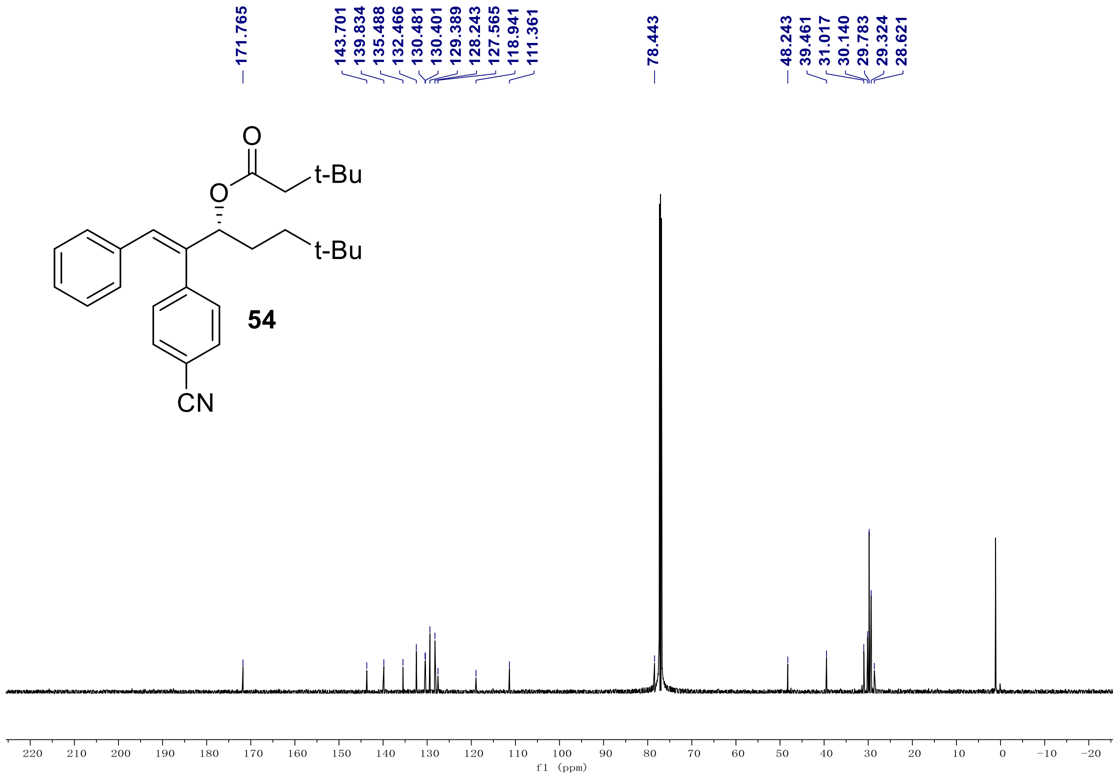

Supplementary Figure 196. ^{13}C NMR spectra of compound **54**

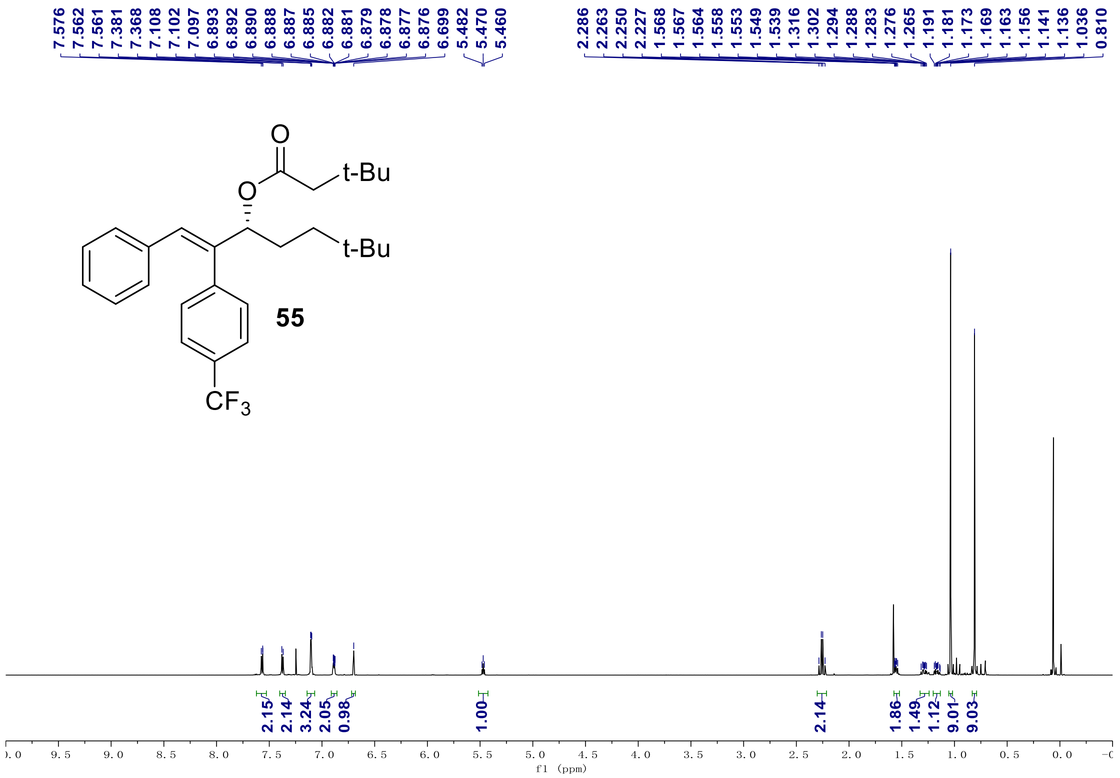

Supplementary Figure 197. ^{1}H NMR spectra of compound **55**

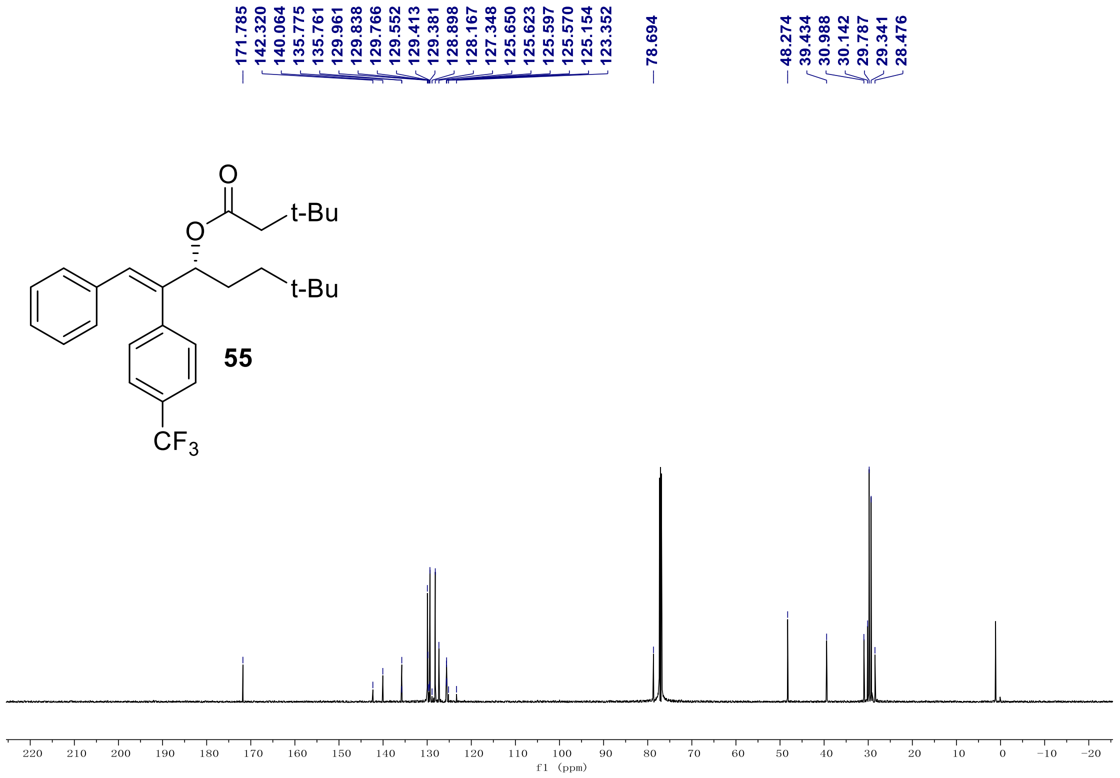

Supplementary Figure 198. ^{13}C NMR spectra of compound **55**

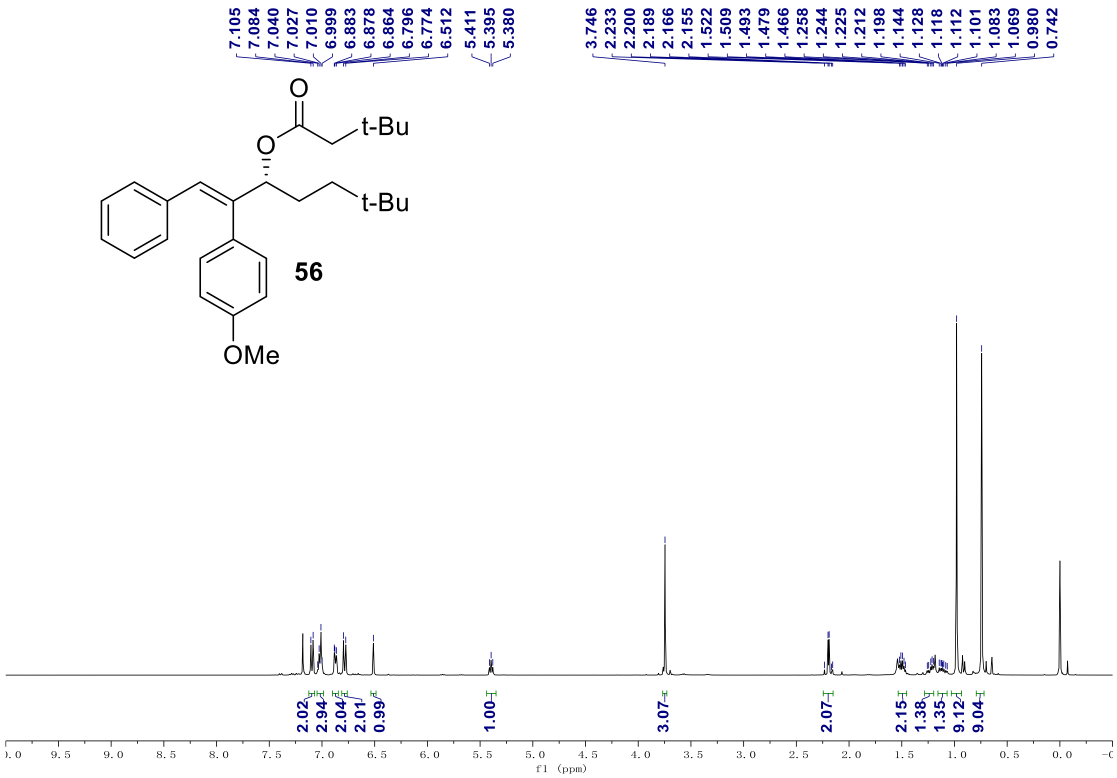

Supplementary Figure 199. ¹H NMR spectra of compound **56**

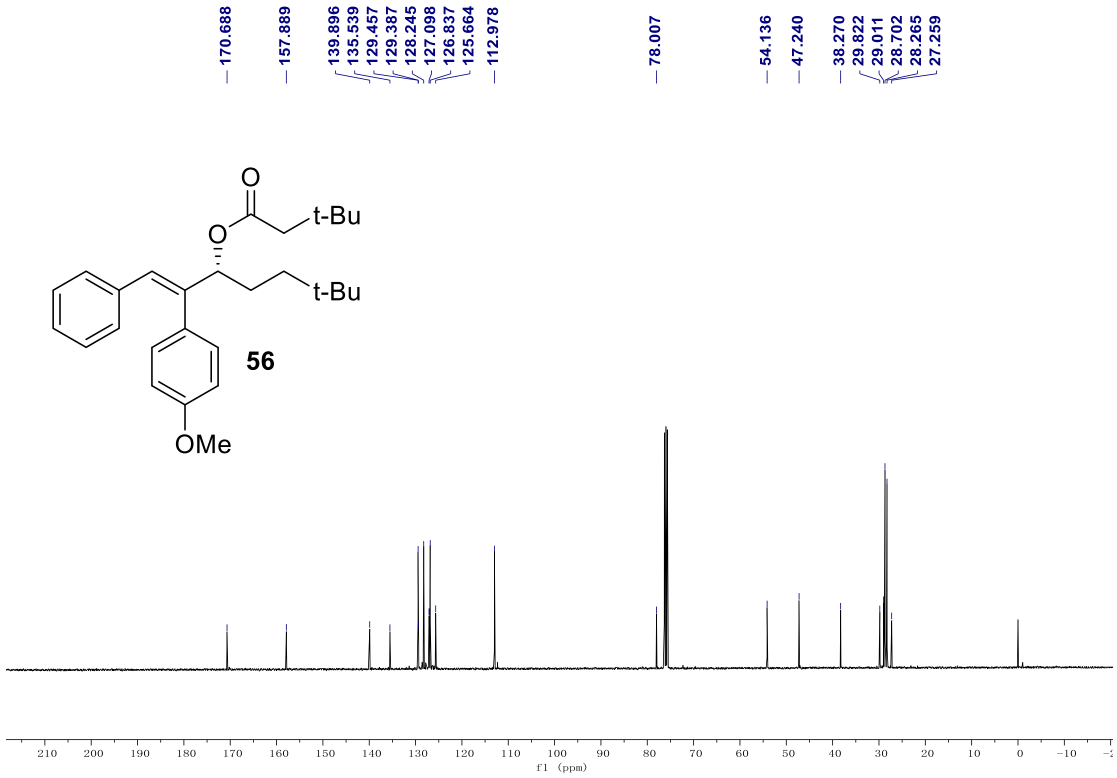

Supplementary Figure 200. ¹³C NMR spectra of compound **56**

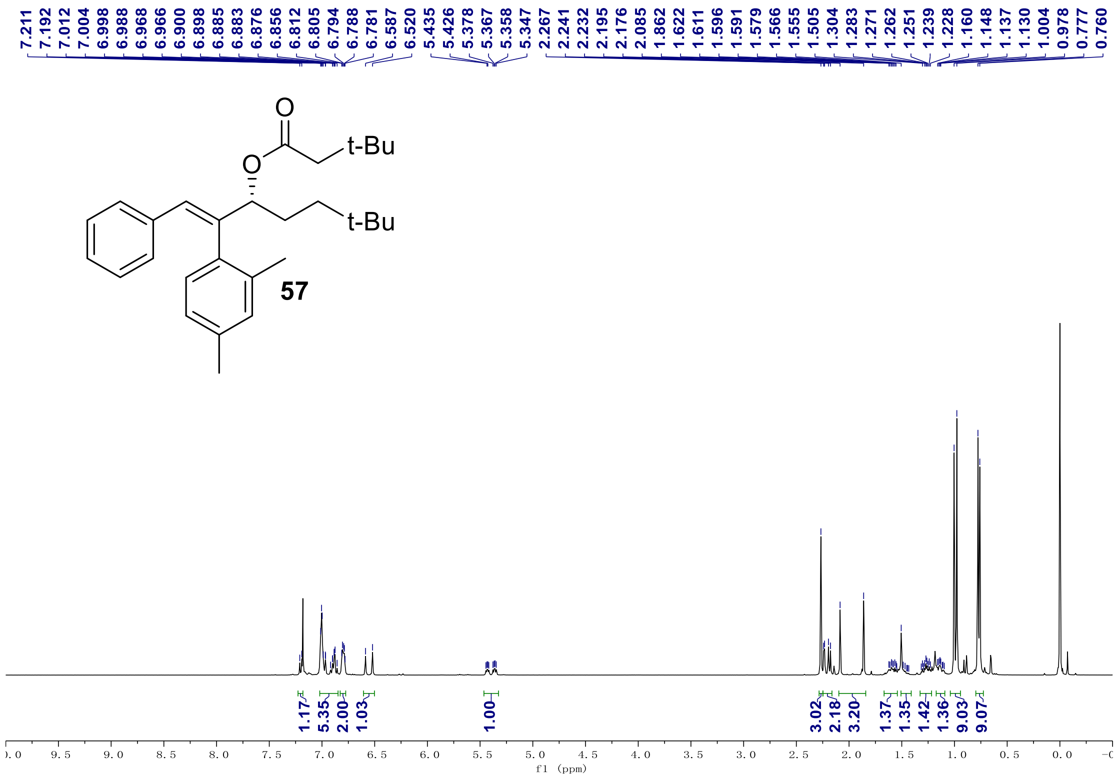

Supplementary Figure 201. ^{1}H NMR spectra of compound **57**

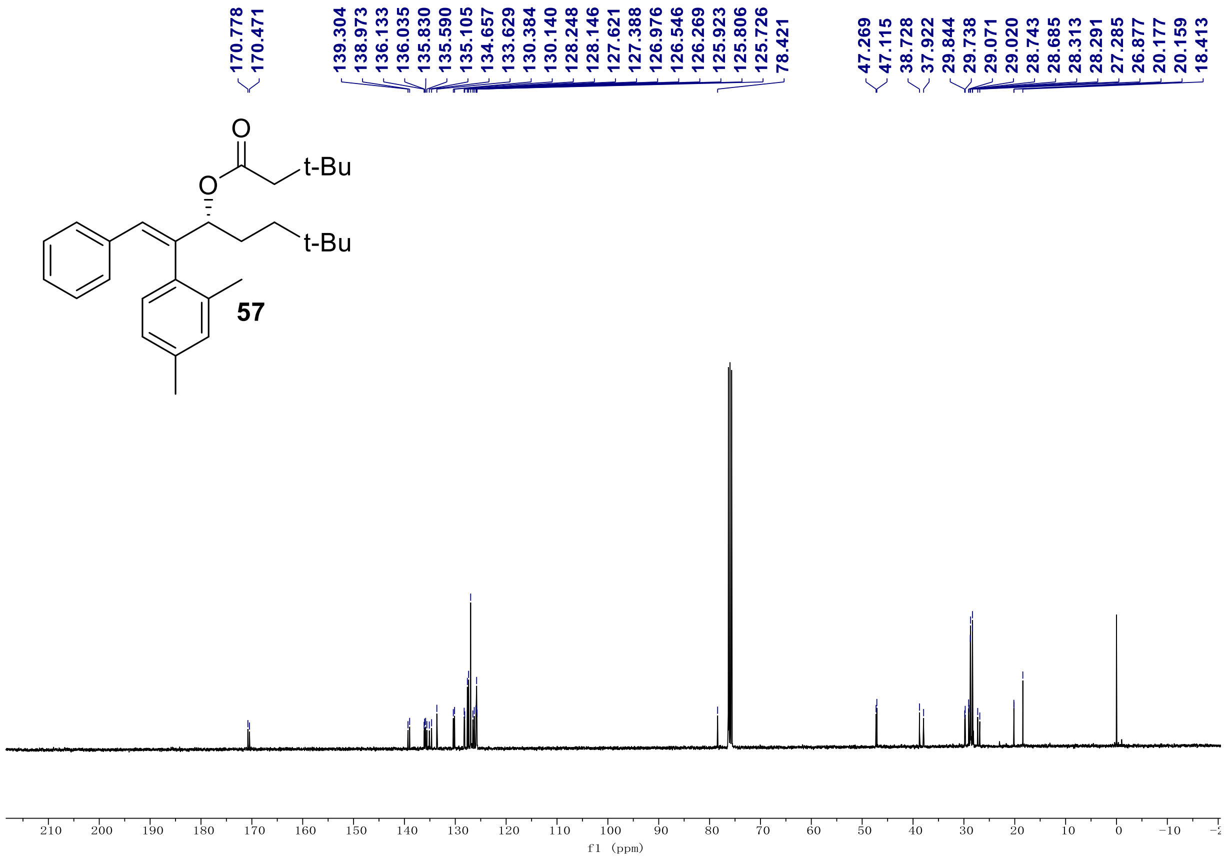

Supplementary Figure 202. ^{13}C NMR spectra of compound **57**

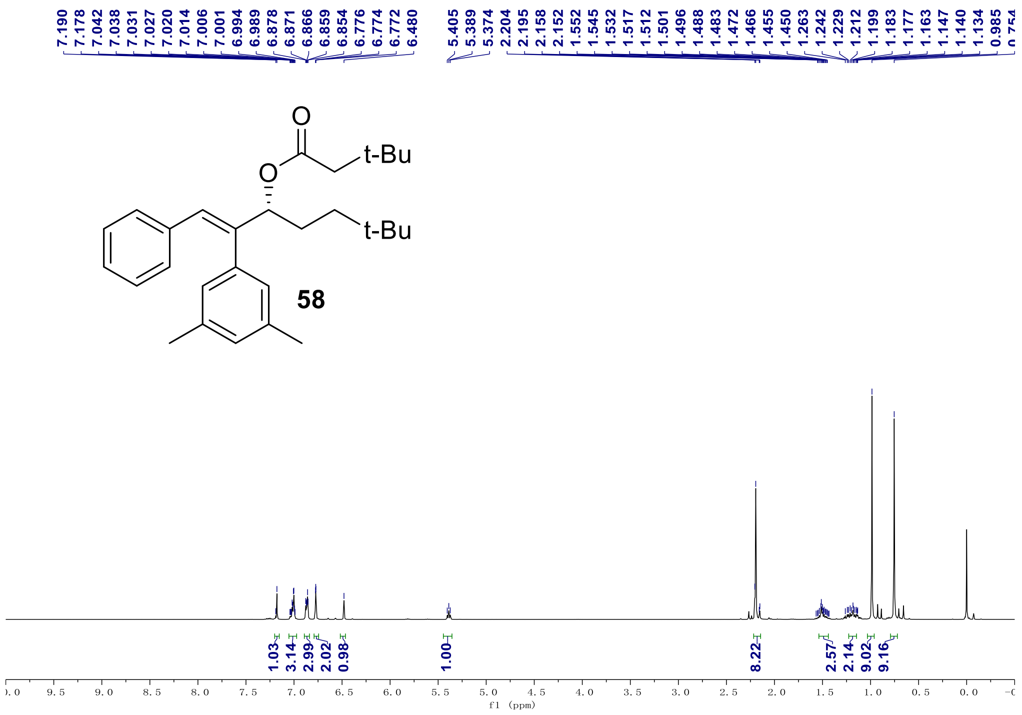

Supplementary Figure 203. ^{1}H NMR spectra of compound **58**

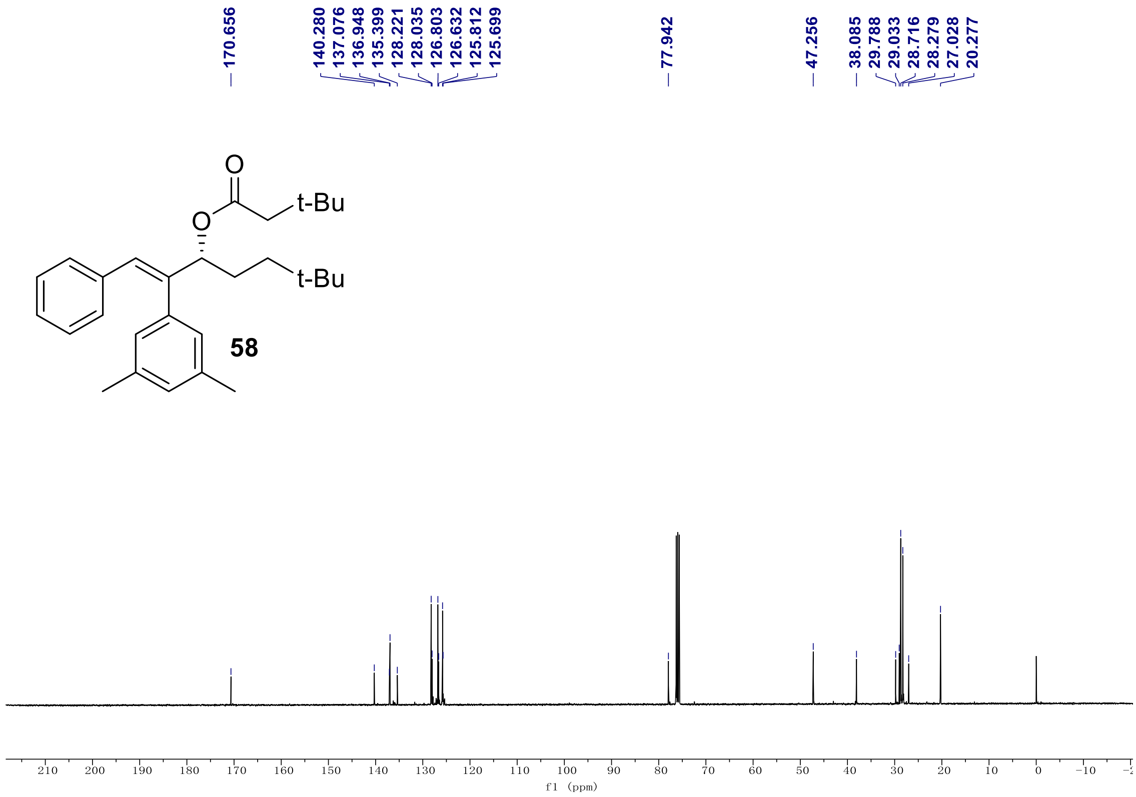

Supplementary Figure 204. ^{13}C NMR spectra of compound **58**

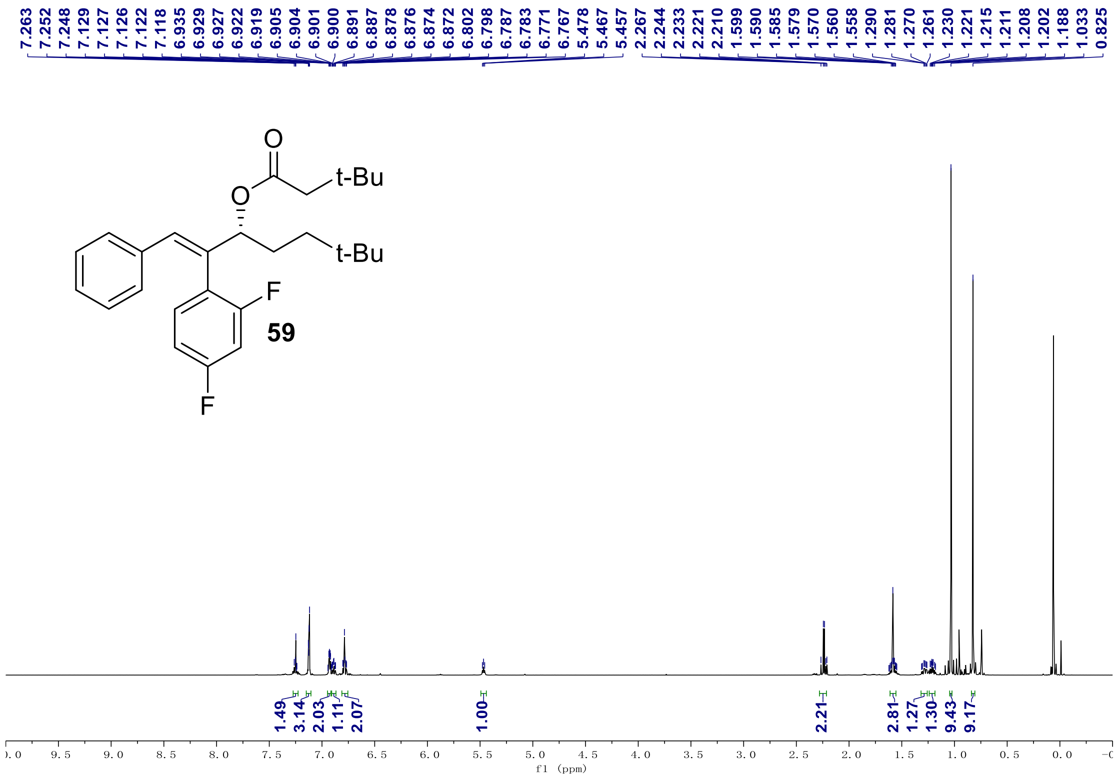

Supplementary Figure 205. ^{1}H NMR spectra of compound **57**

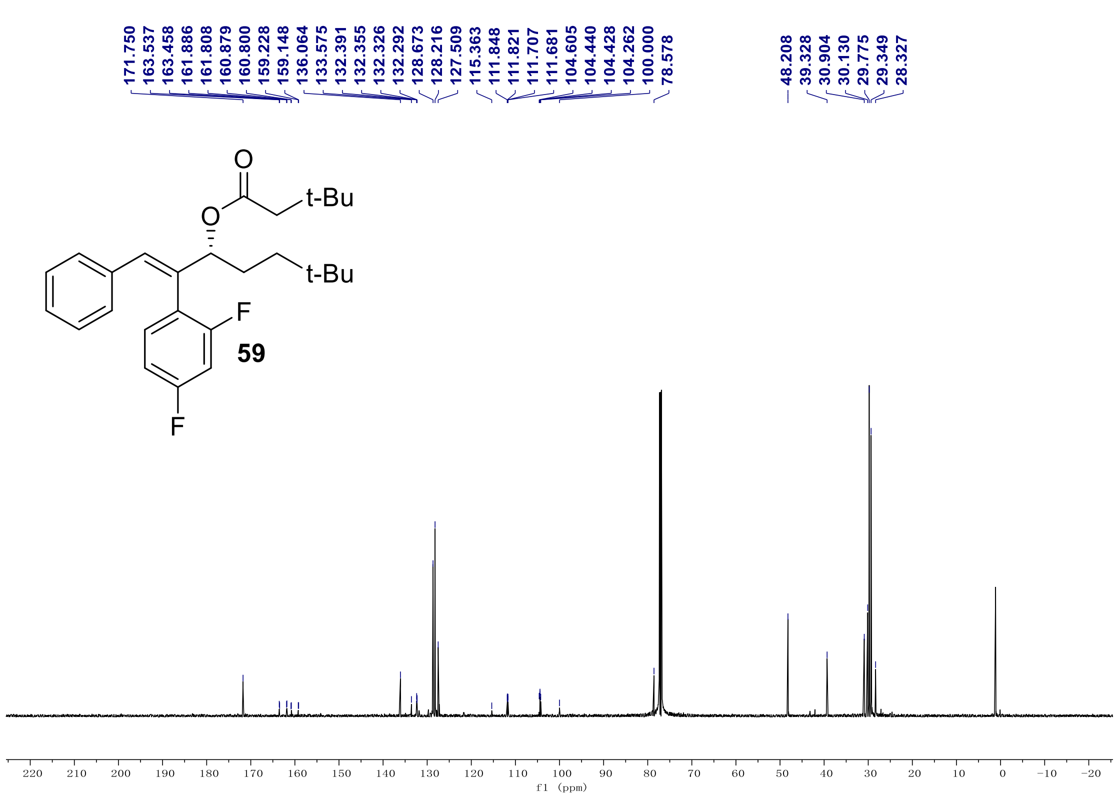

Supplementary Figure 206. ^{13}C NMR spectra of compound **59**

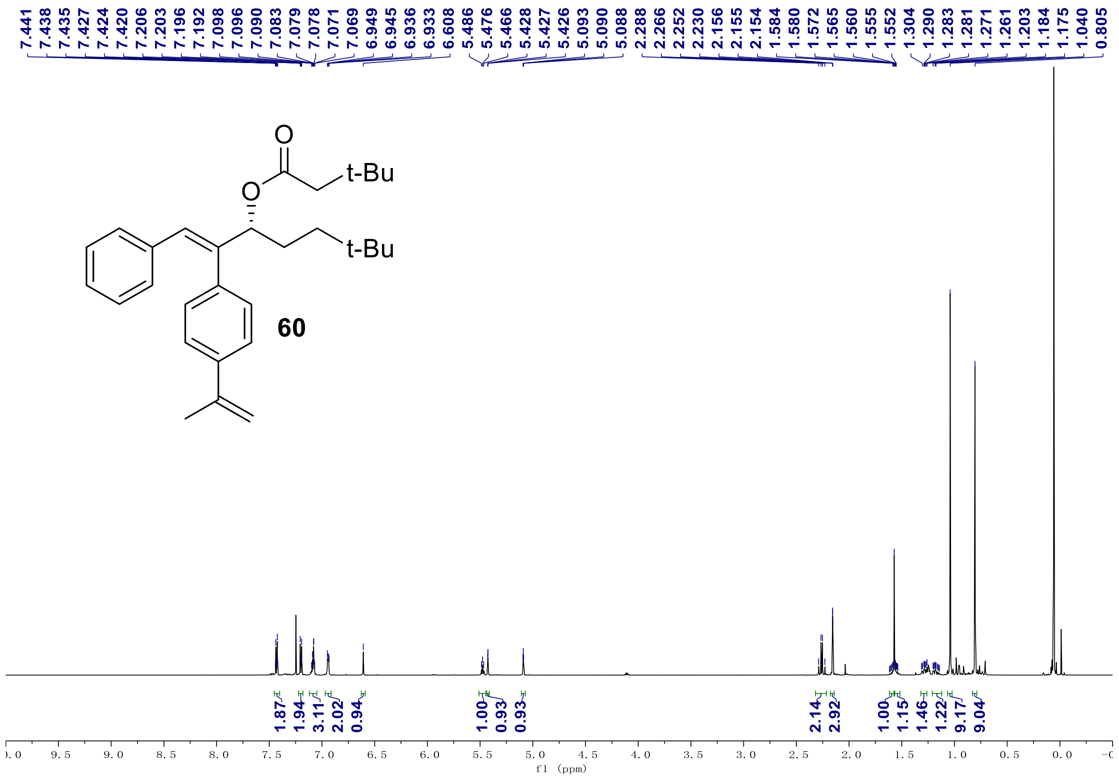

Supplementary Figure 207. ^{1}H NMR spectra of compound **60**

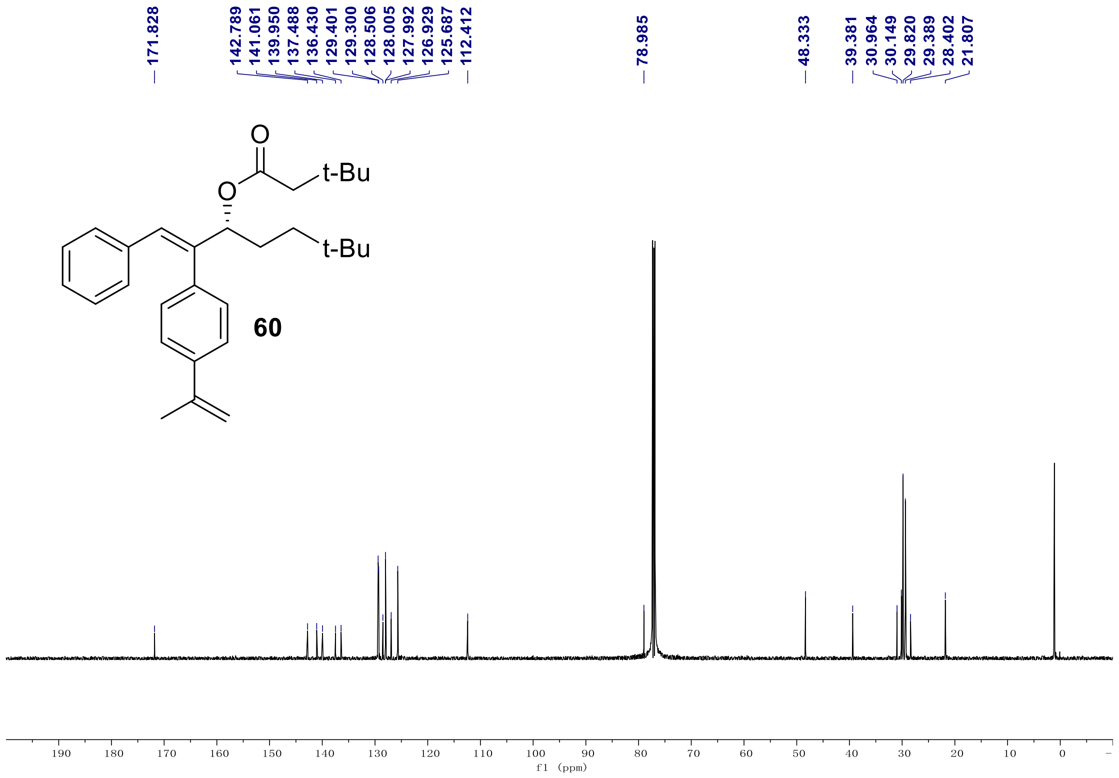

Supplementary Figure 208. ^{13}C NMR spectra of compound **60**

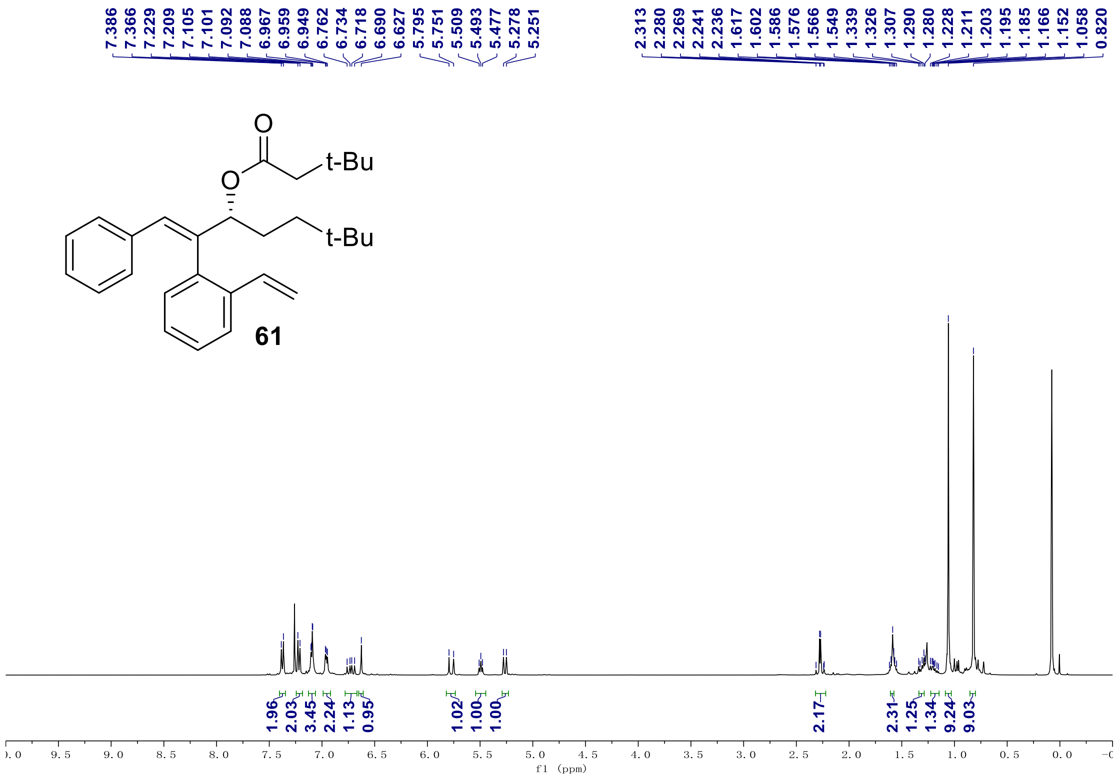

Supplementary Figure 209. ^{1}H NMR spectra of compound **61**

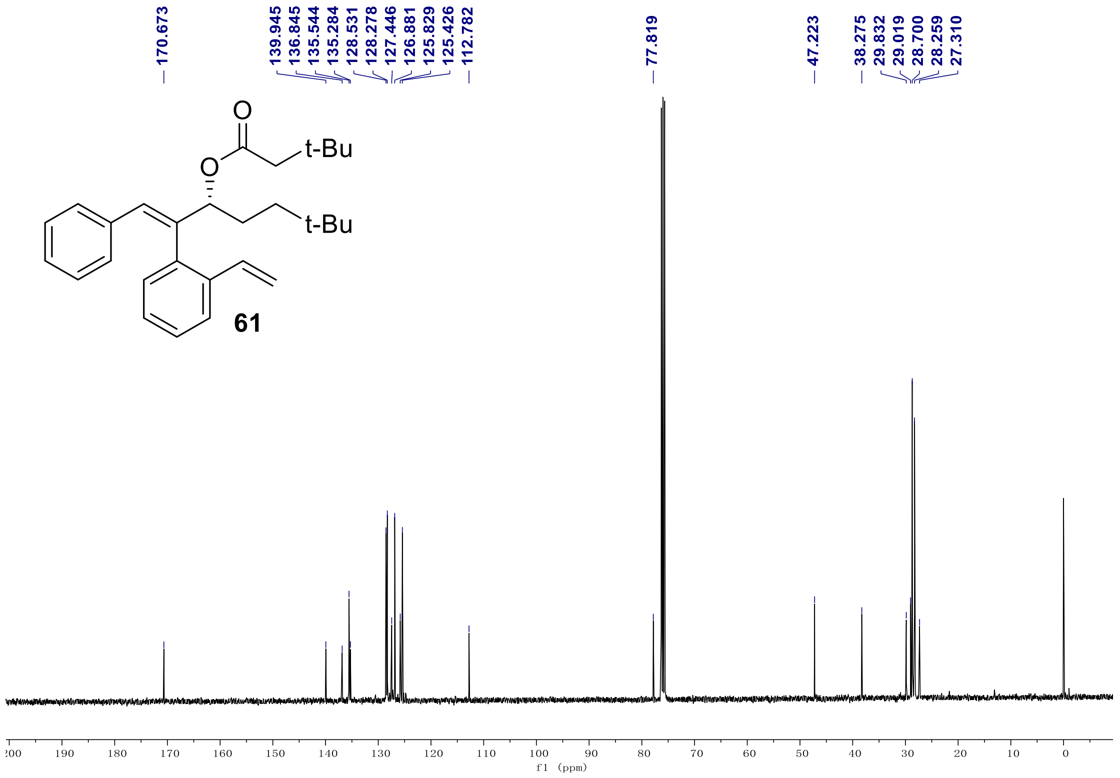

Supplementary Figure 210. ^{13}C NMR spectra of compound **61**

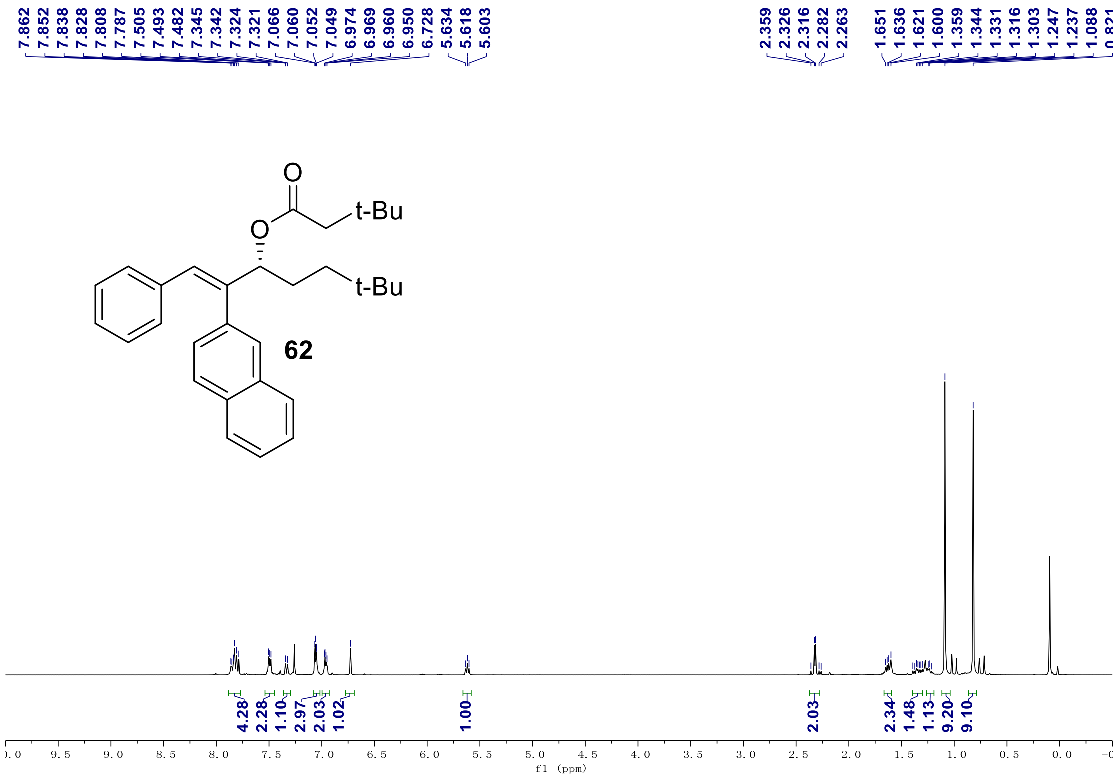

Supplementary Figure 211. ^{1}H NMR spectra of compound **62**

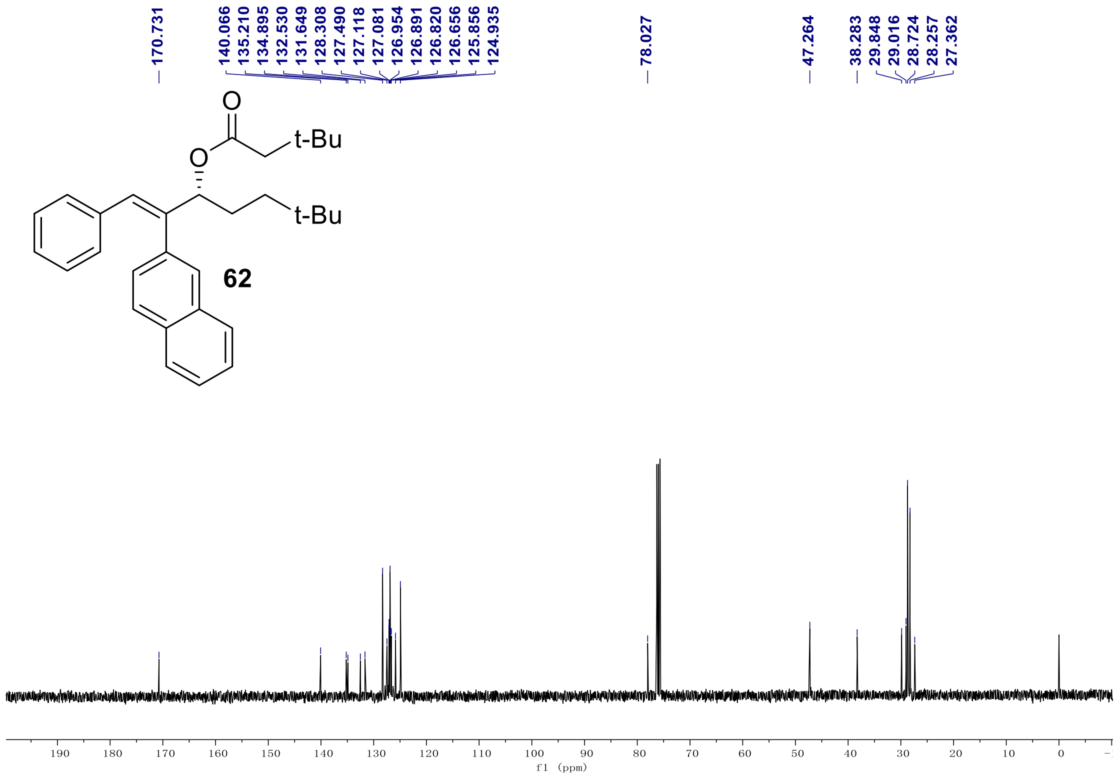

Supplementary Figure 212. ^{13}C NMR spectra of compound **62**

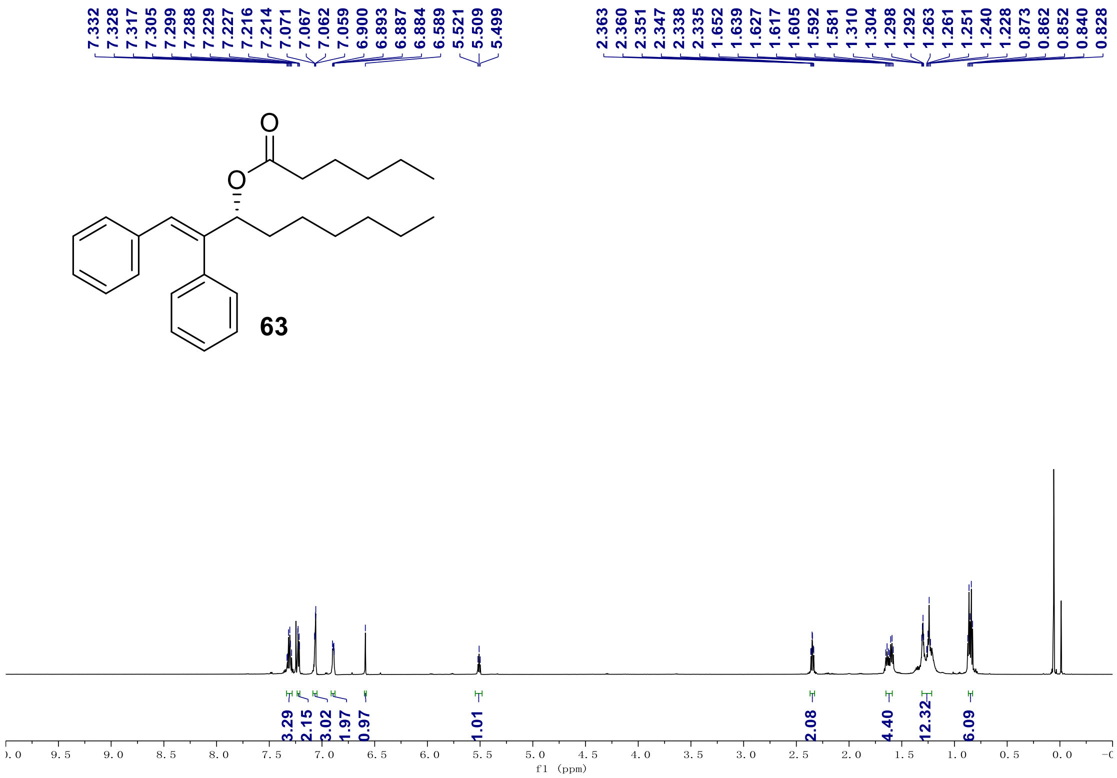

Supplementary Figure 213. ^{1}H NMR spectra of compound **63**

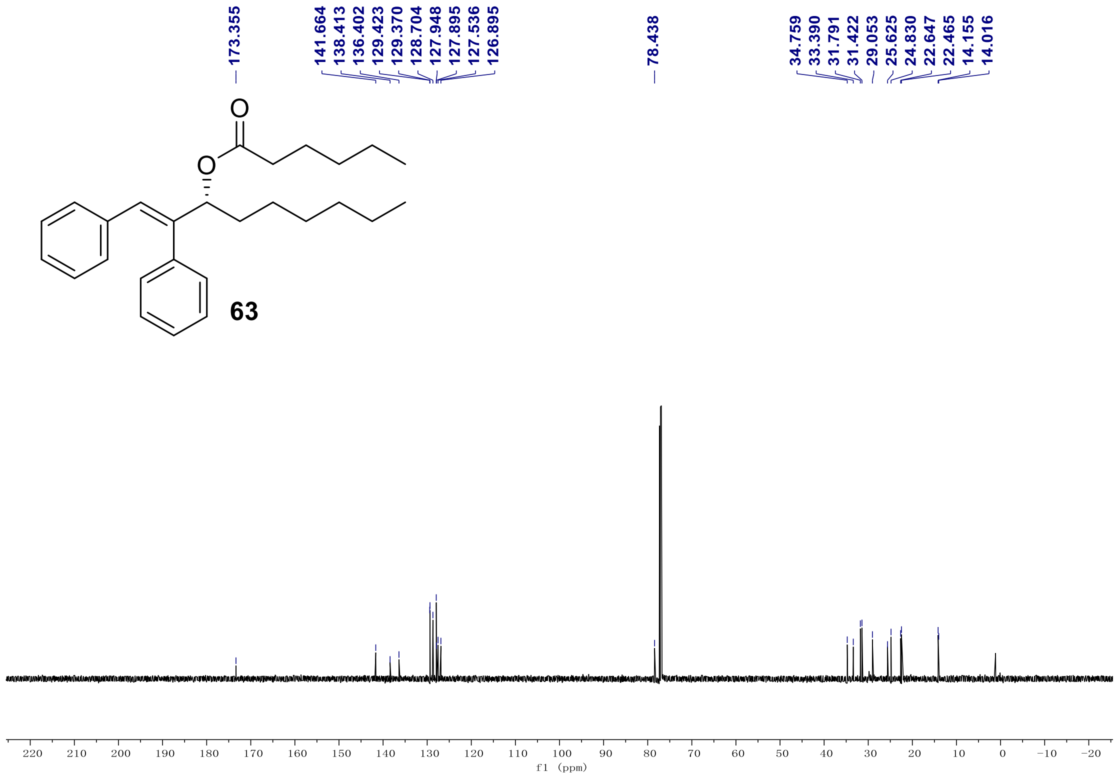

Supplementary Figure 214. ^{13}C NMR spectra of compound **63**

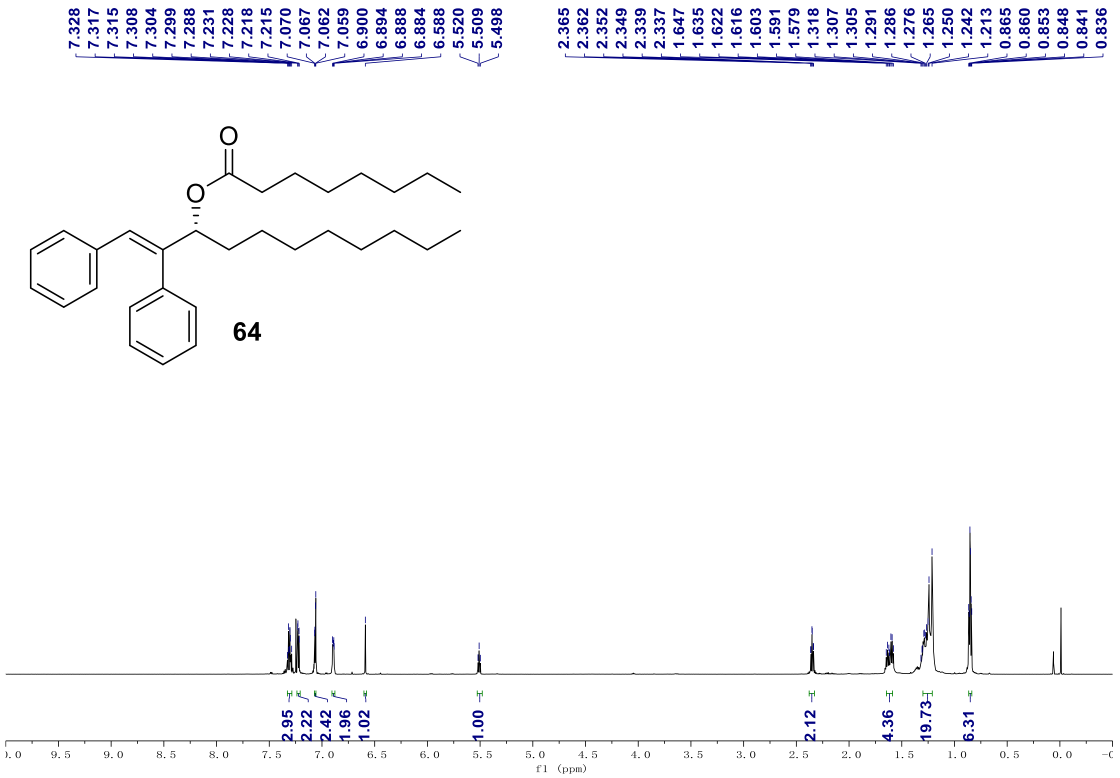

Supplementary Figure 215. ^{1}H NMR spectra of compound **64**

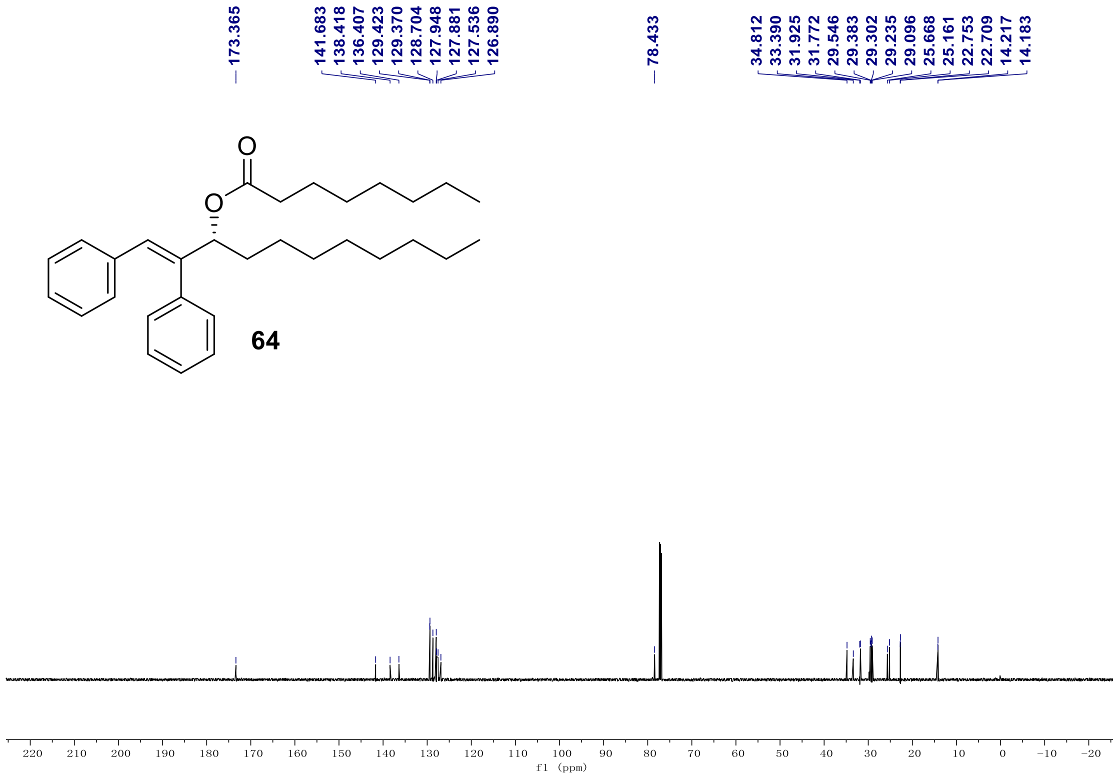

Supplementary Figure 216. ^{13}C NMR spectra of compound **64**

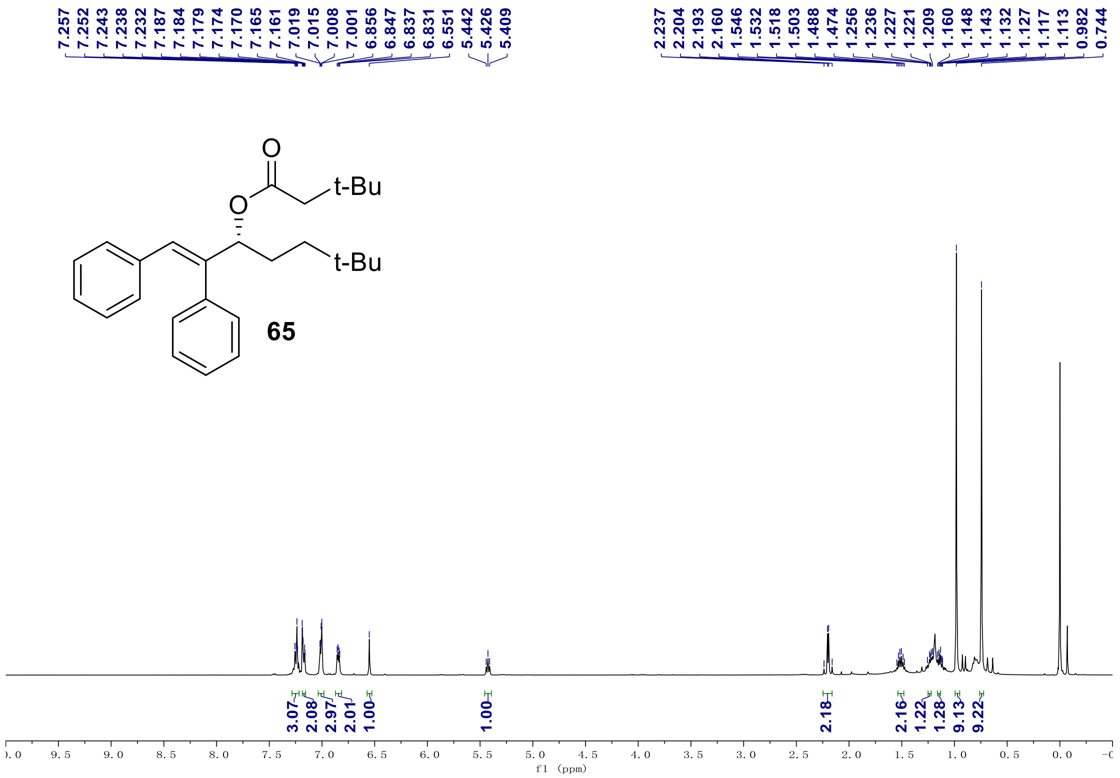

Supplementary Figure 217. ^{1}H NMR spectra of compound **65**

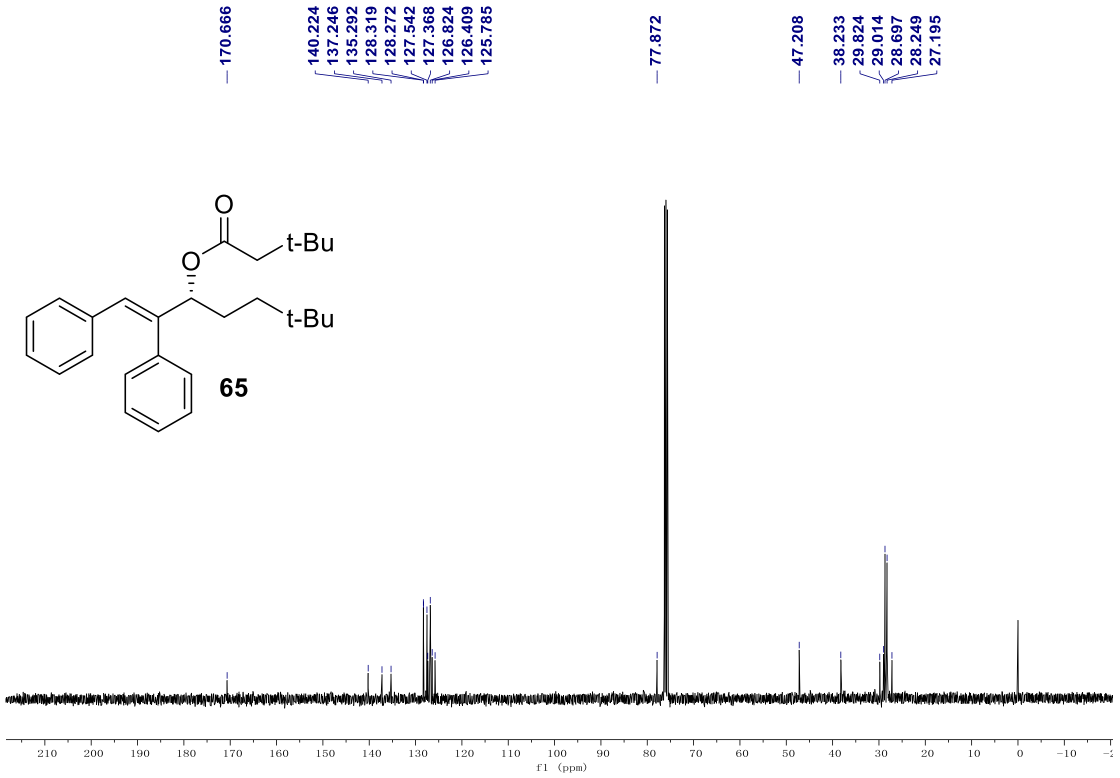

Supplementary Figure 218. ^{13}C NMR spectra of compound **65**

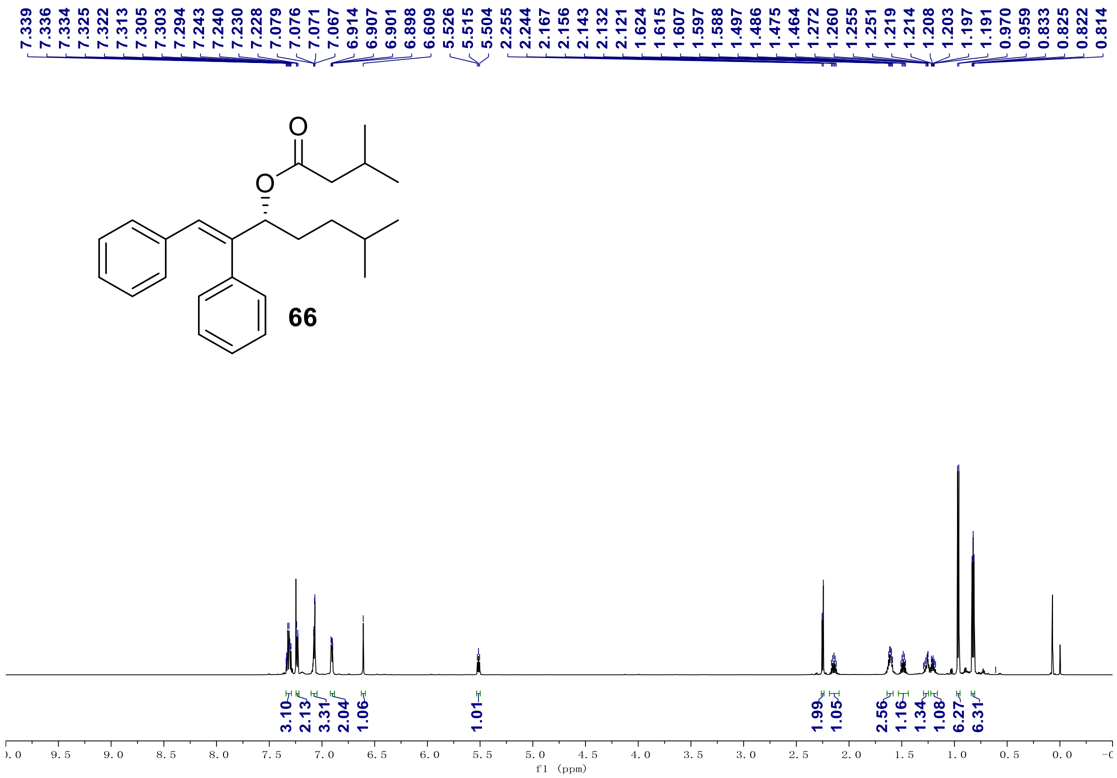

Supplementary Figure 219. ^{1}H NMR spectra of compound **66**

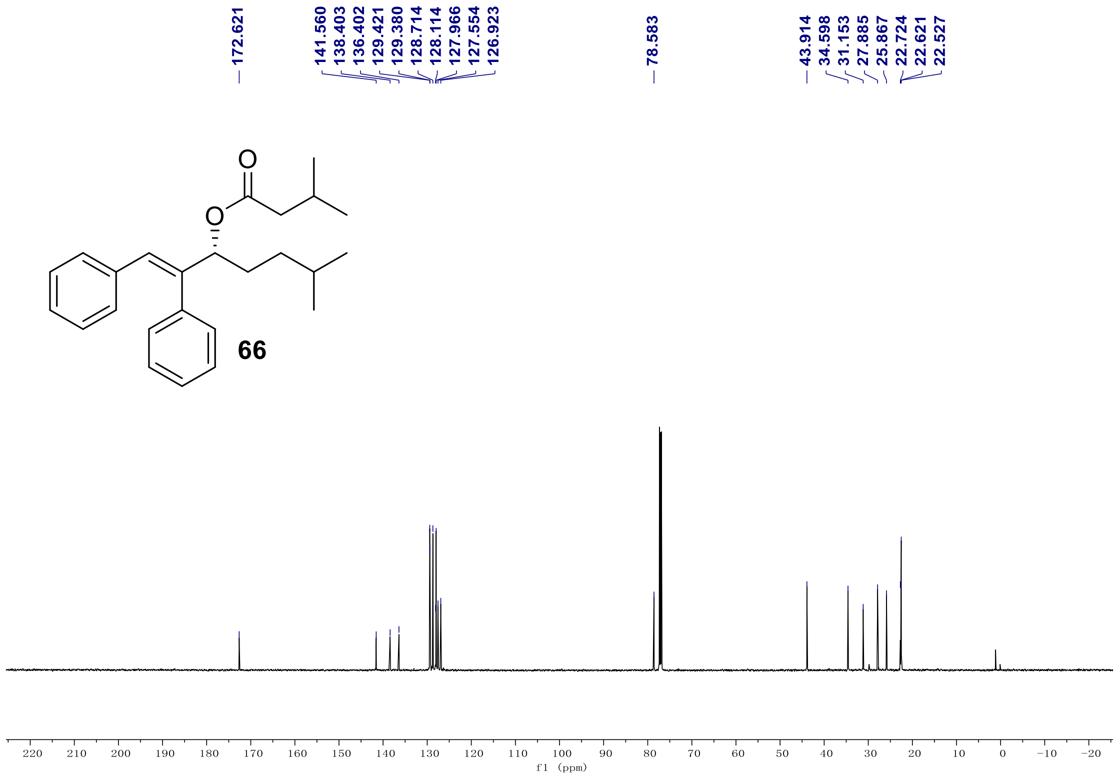

Supplementary Figure 220. ^{13}C NMR spectra of compound **66**

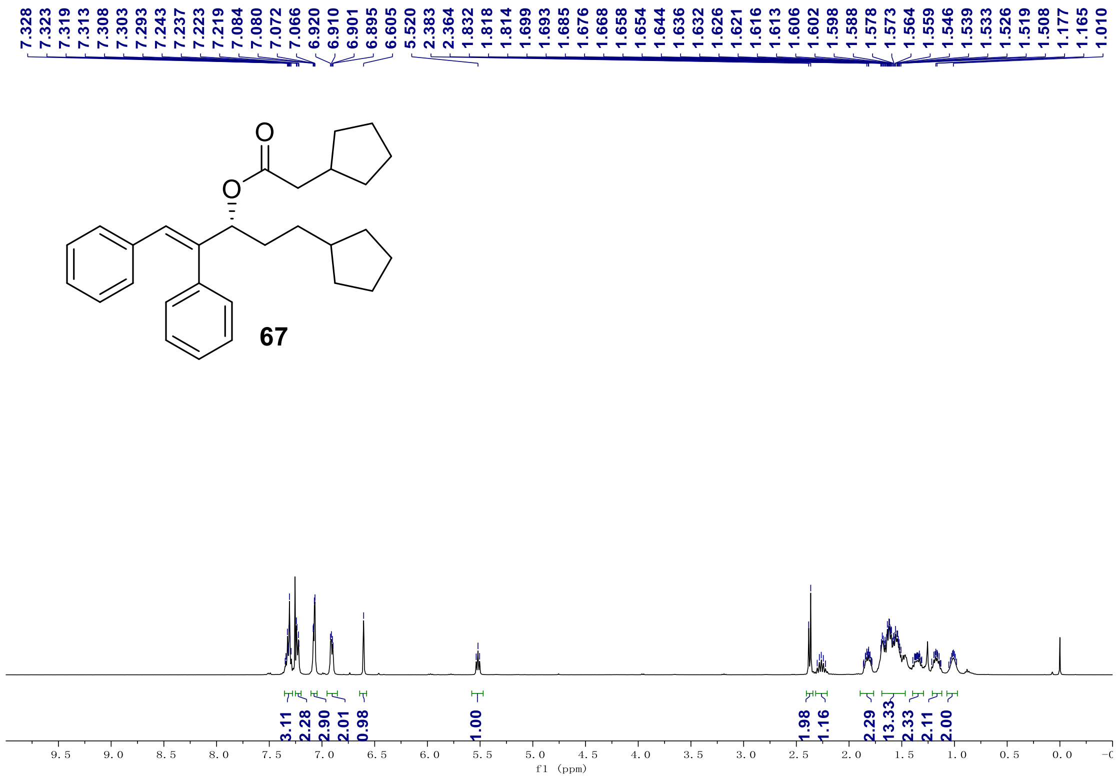

Supplementary Figure 221. ¹H NMR spectra of compound **67**

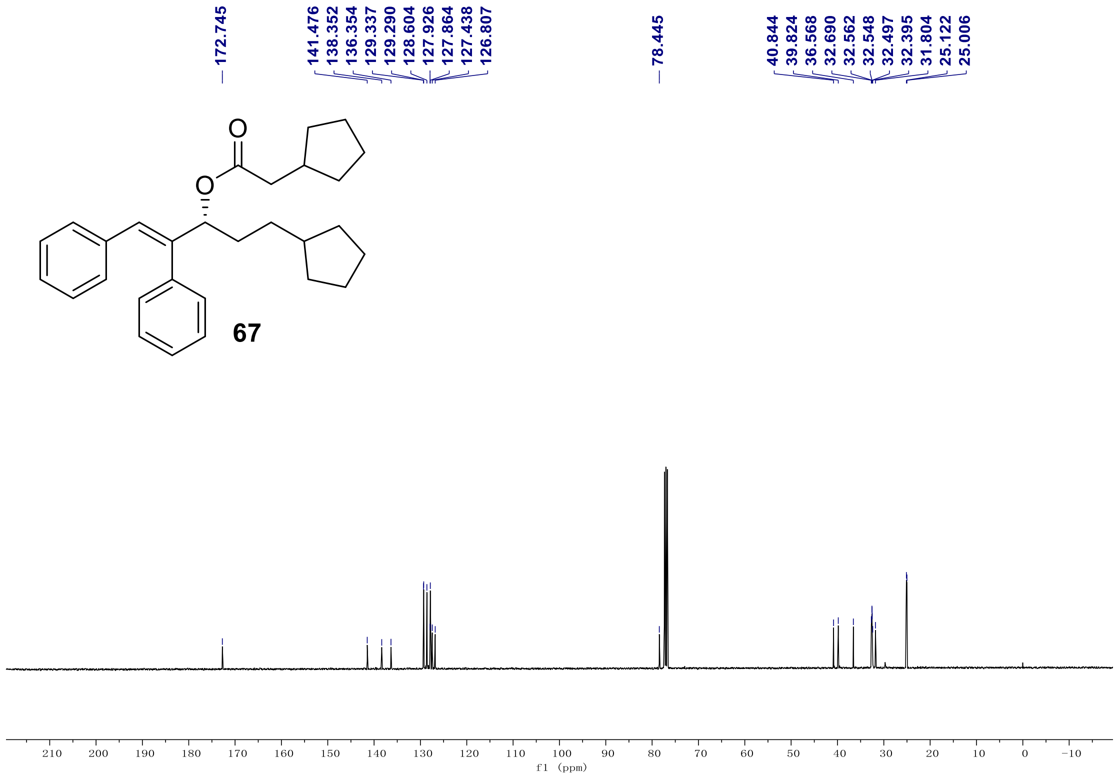

Supplementary Figure 222. ¹³C NMR spectra of compound **67**

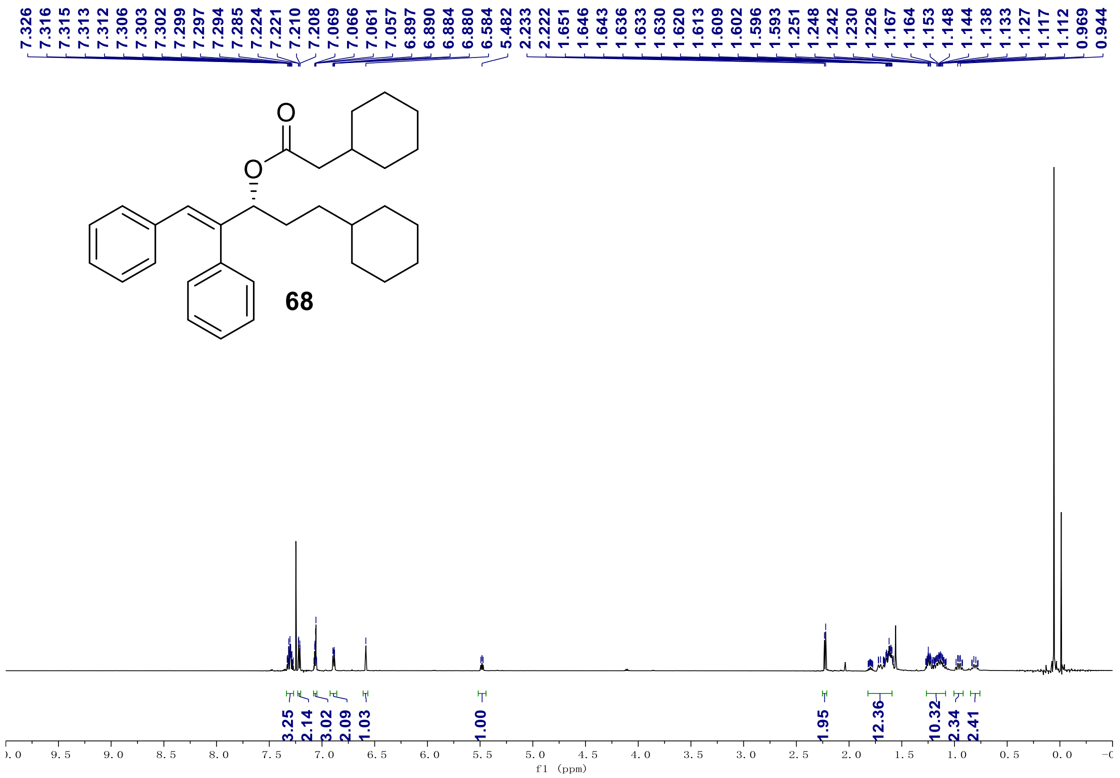

Supplementary Figure 223. ^{1}H NMR spectra of compound **68**

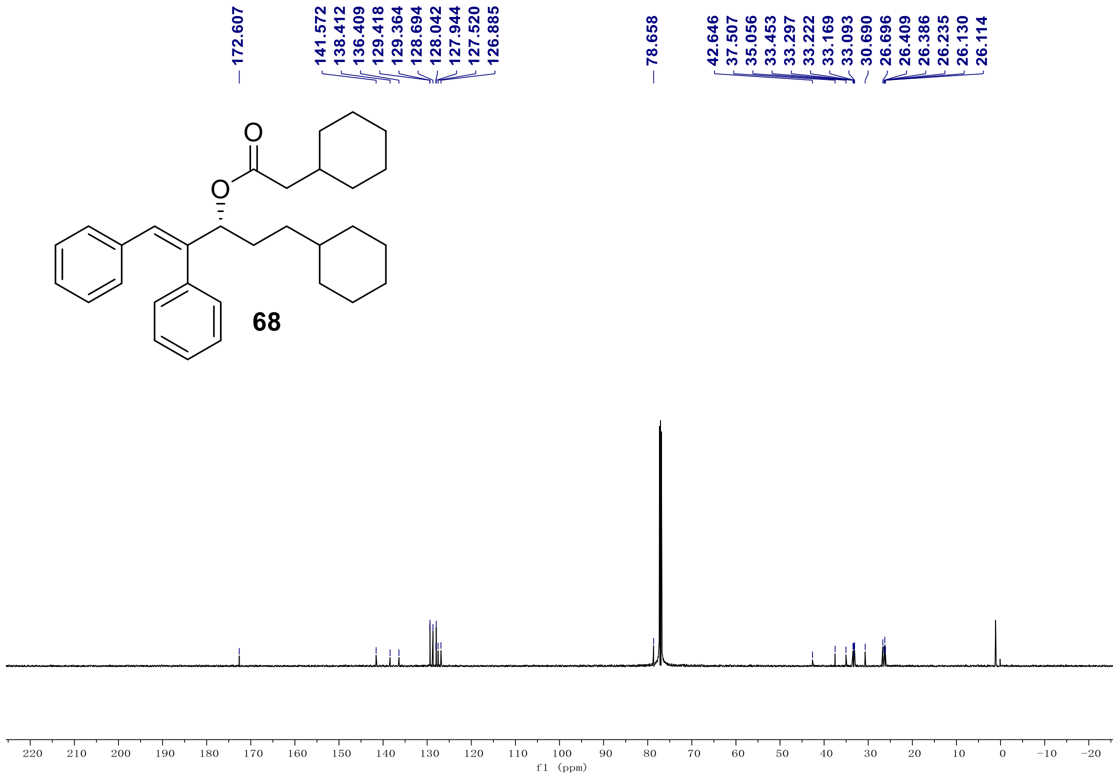

Supplementary Figure 224. ^{13}C NMR spectra of compound **68**

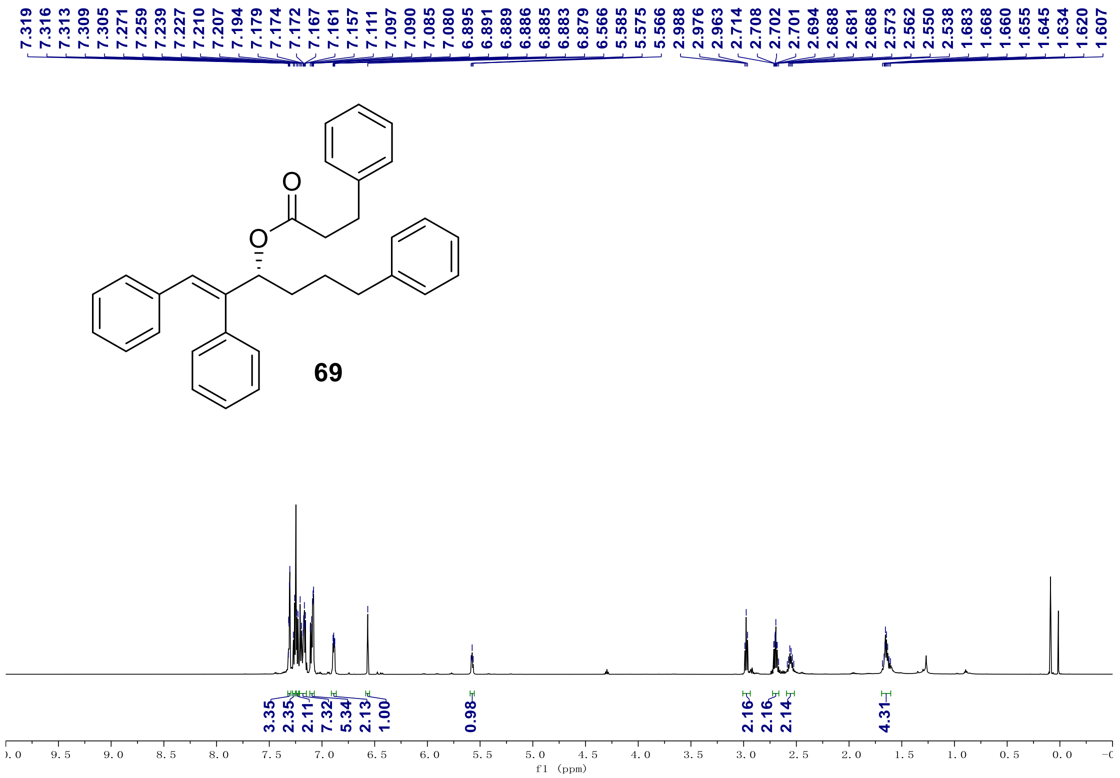

Supplementary Figure 225. ^{1}H NMR spectra of compound **69**

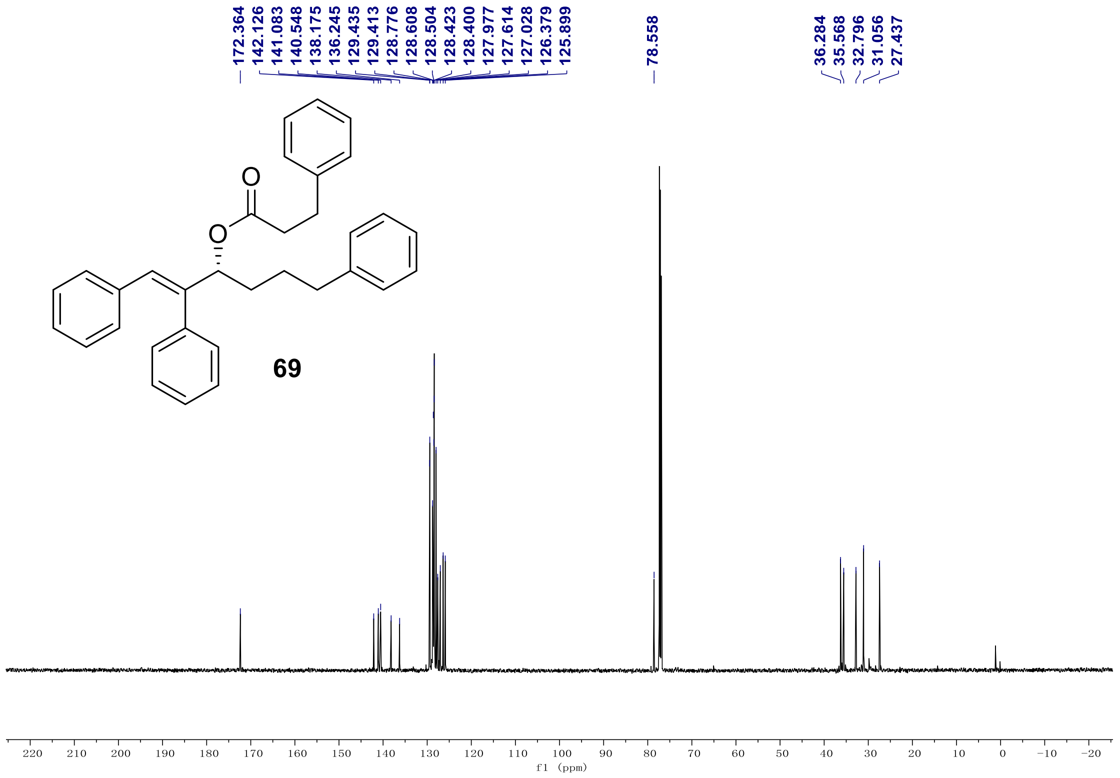

Supplementary Figure 226. ^{13}C NMR spectra of compound **69**

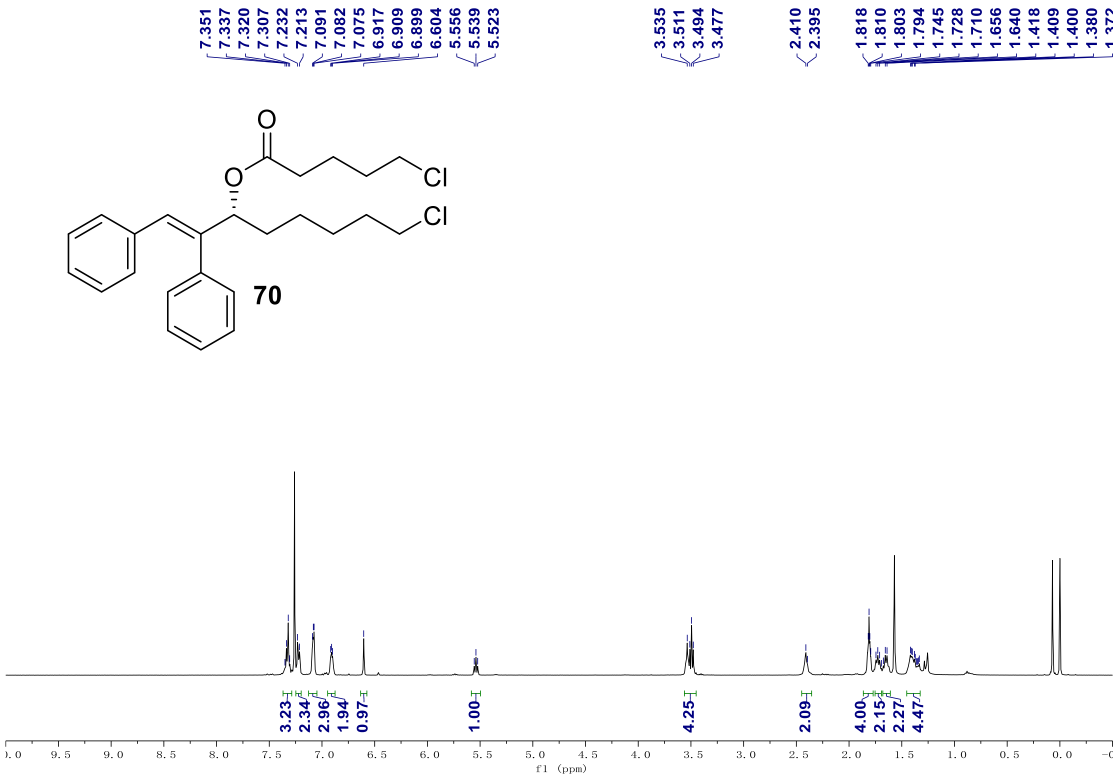

Supplementary Figure 227. ^{1}H NMR spectra of compound **70**

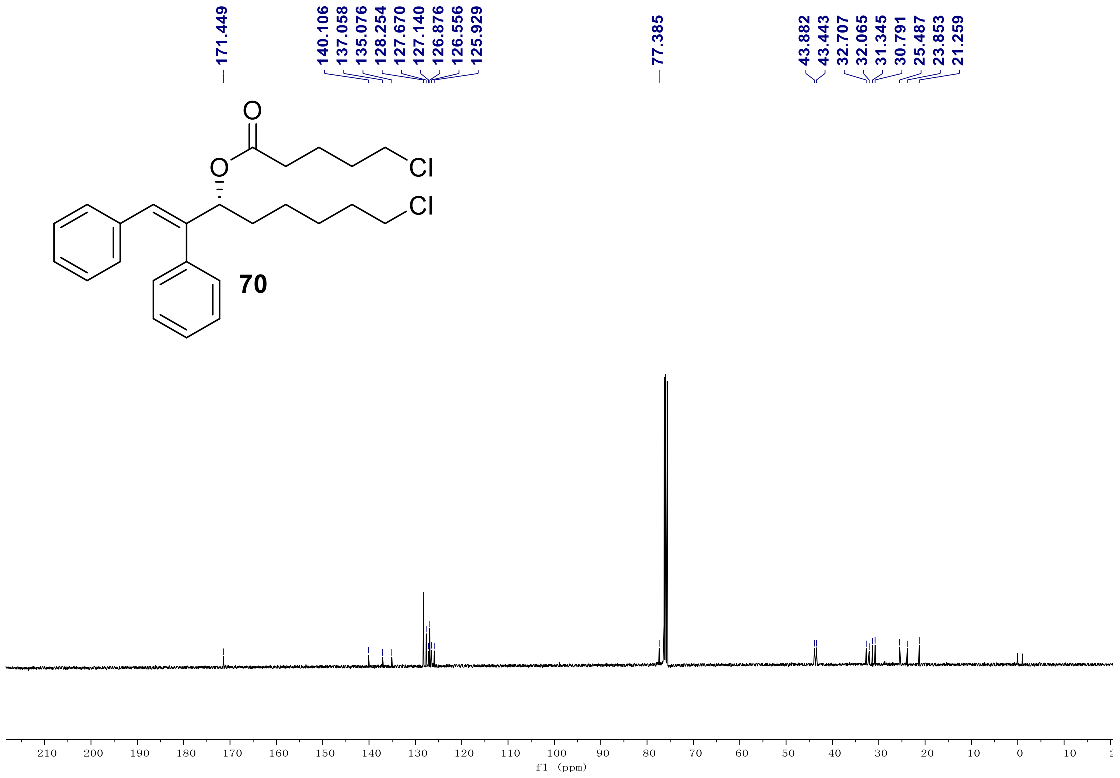

Supplementary Figure 228. ^{13}C NMR spectra of compound **70**

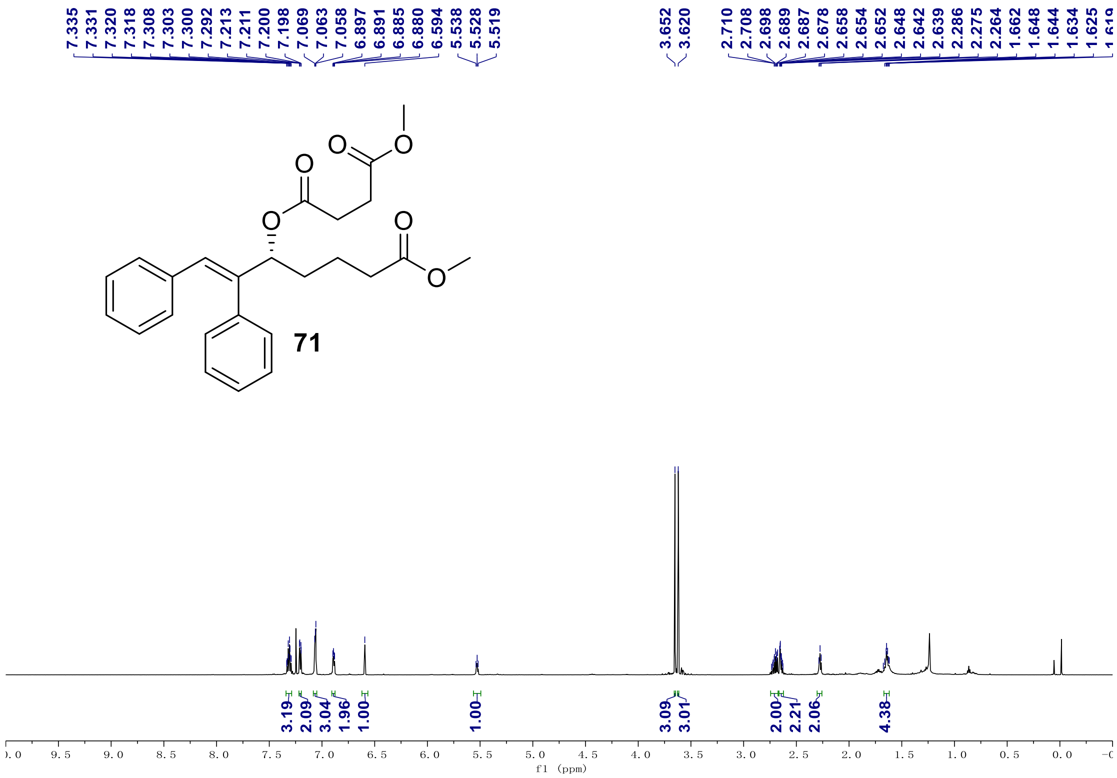

Supplementary Figure 229. ^{1}H NMR spectra of compound **71**

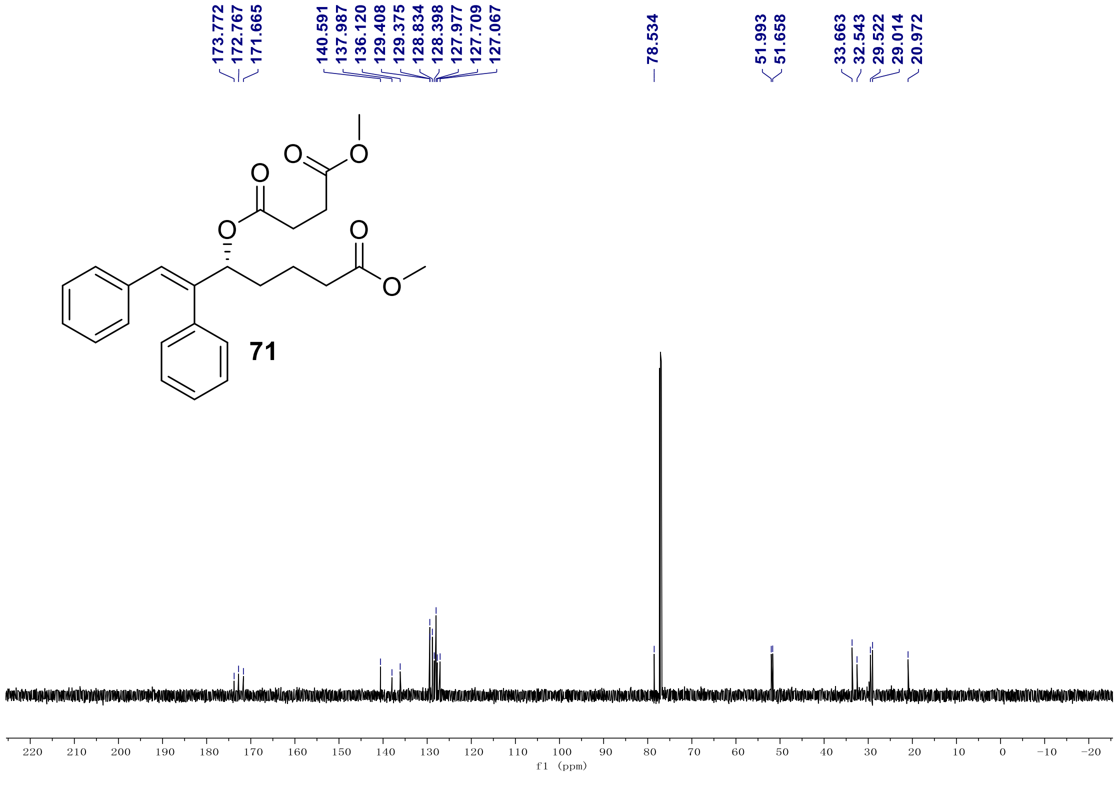

Supplementary Figure 230. ^{13}C NMR spectra of compound **71**

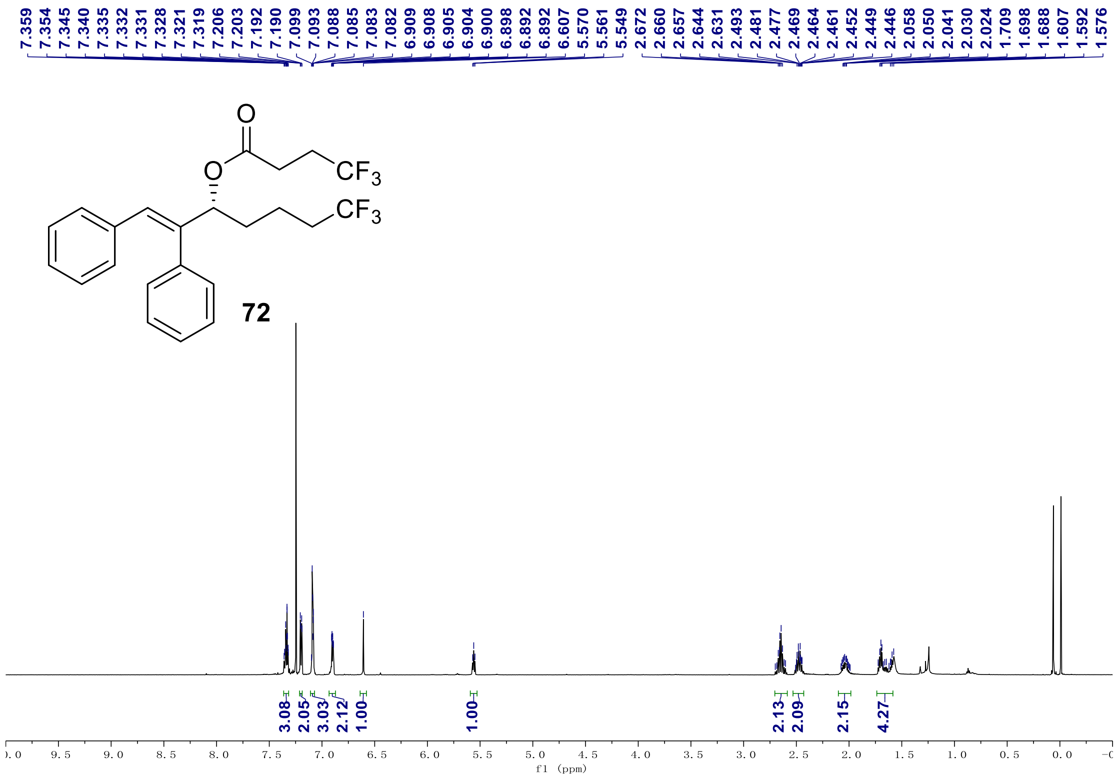

Supplementary Figure 231. ^{1}H NMR spectra of compound **72**

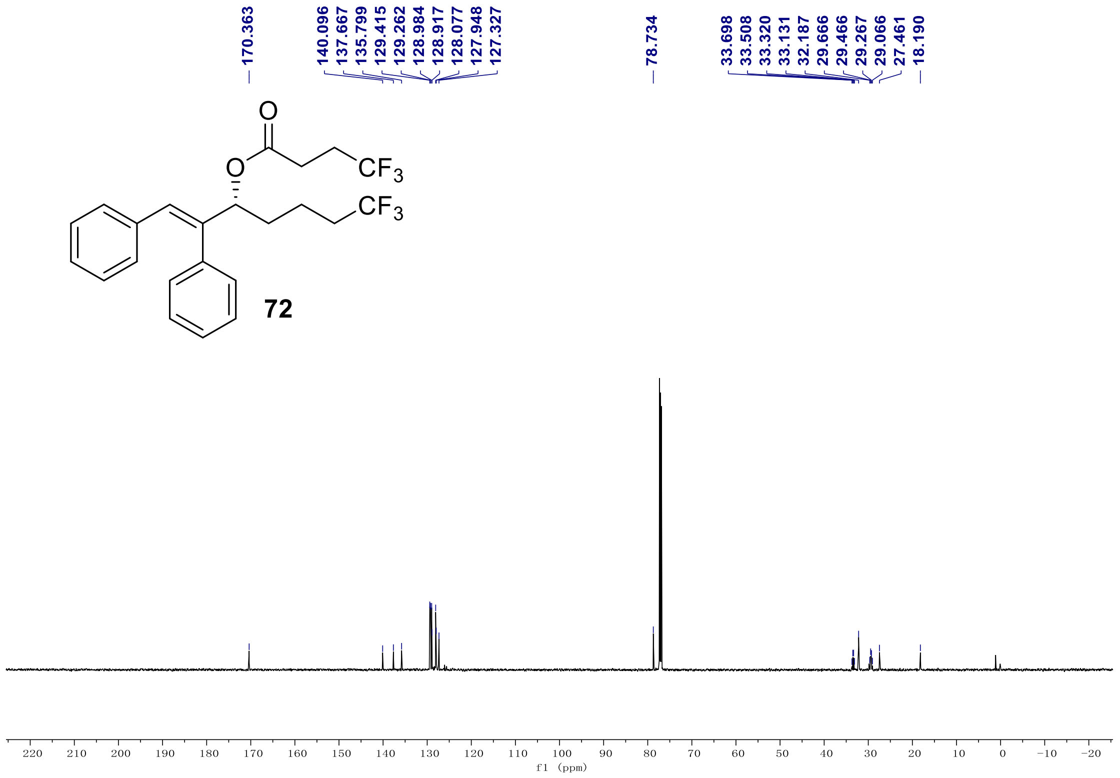

Supplementary Figure 232. ^{13}C NMR spectra of compound **72**

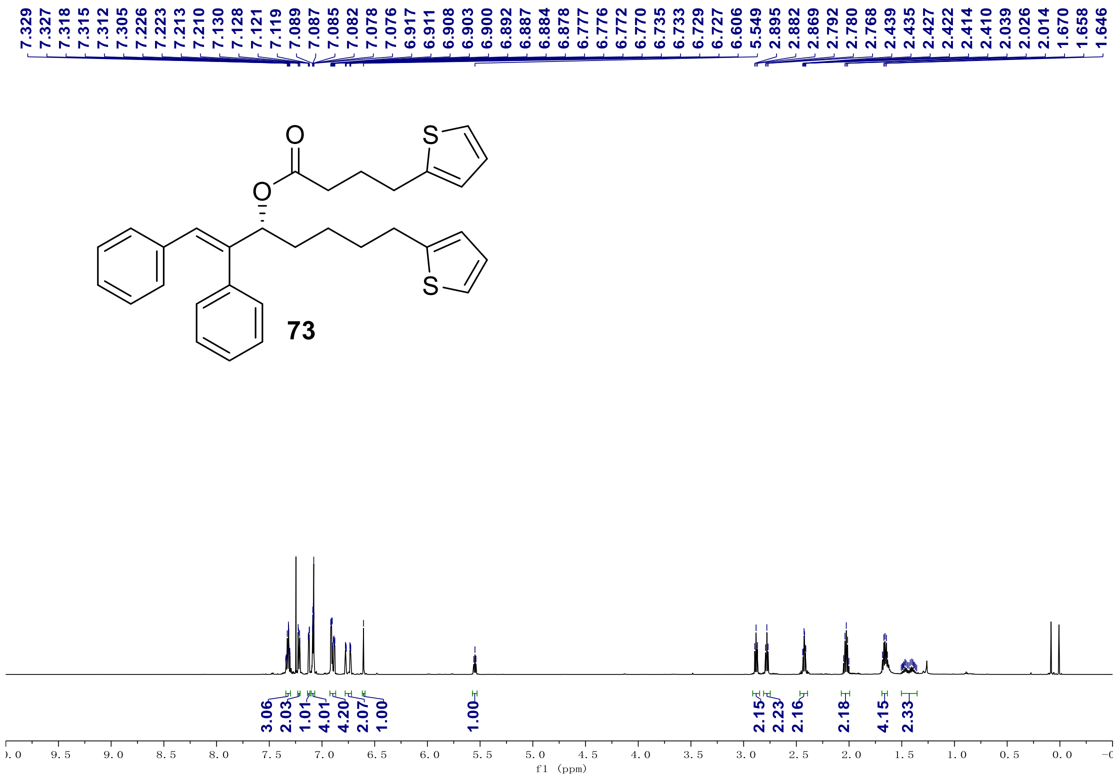

Supplementary Figure 233. ^{1}H NMR spectra of compound **73**

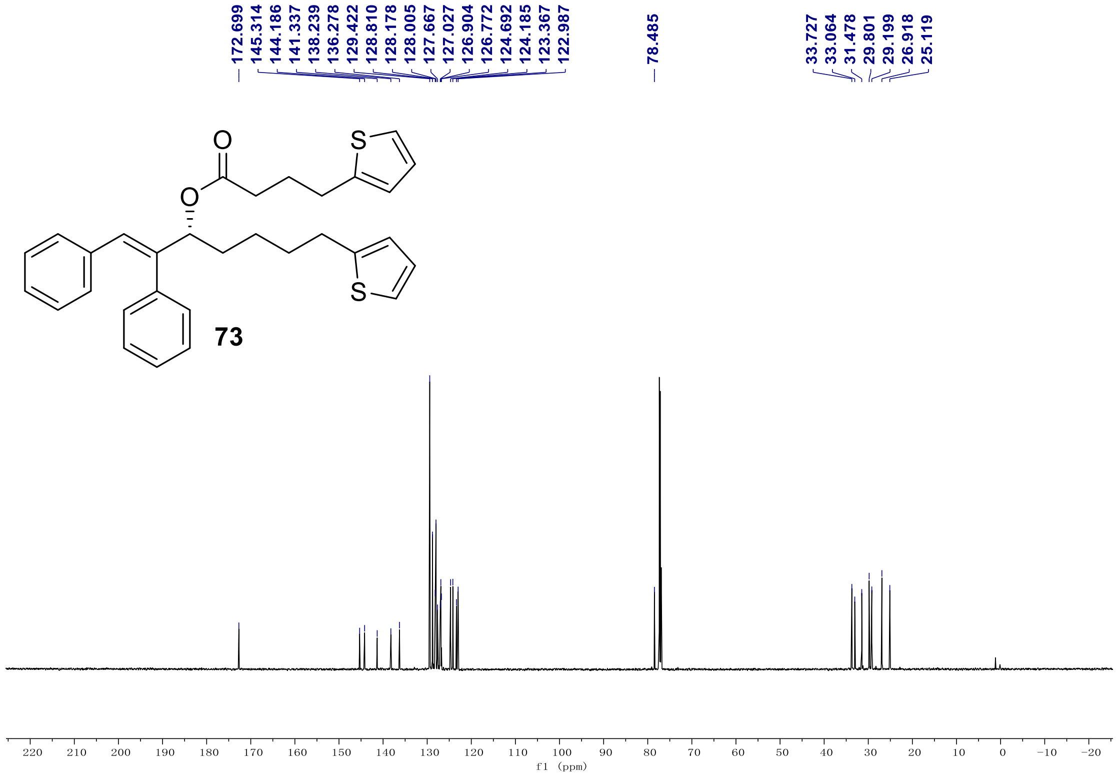

Supplementary Figure 234. ^{13}C NMR spectra of compound **73**

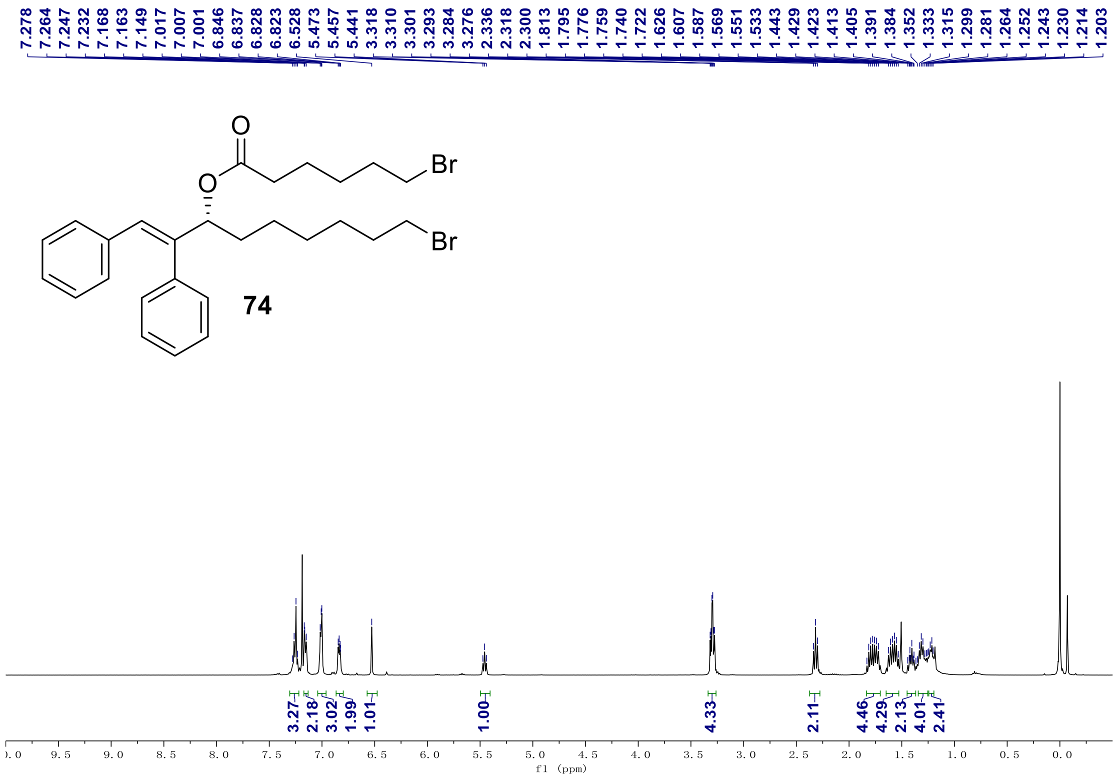

Supplementary Figure 235. ^{1}H NMR spectra of compound **74**

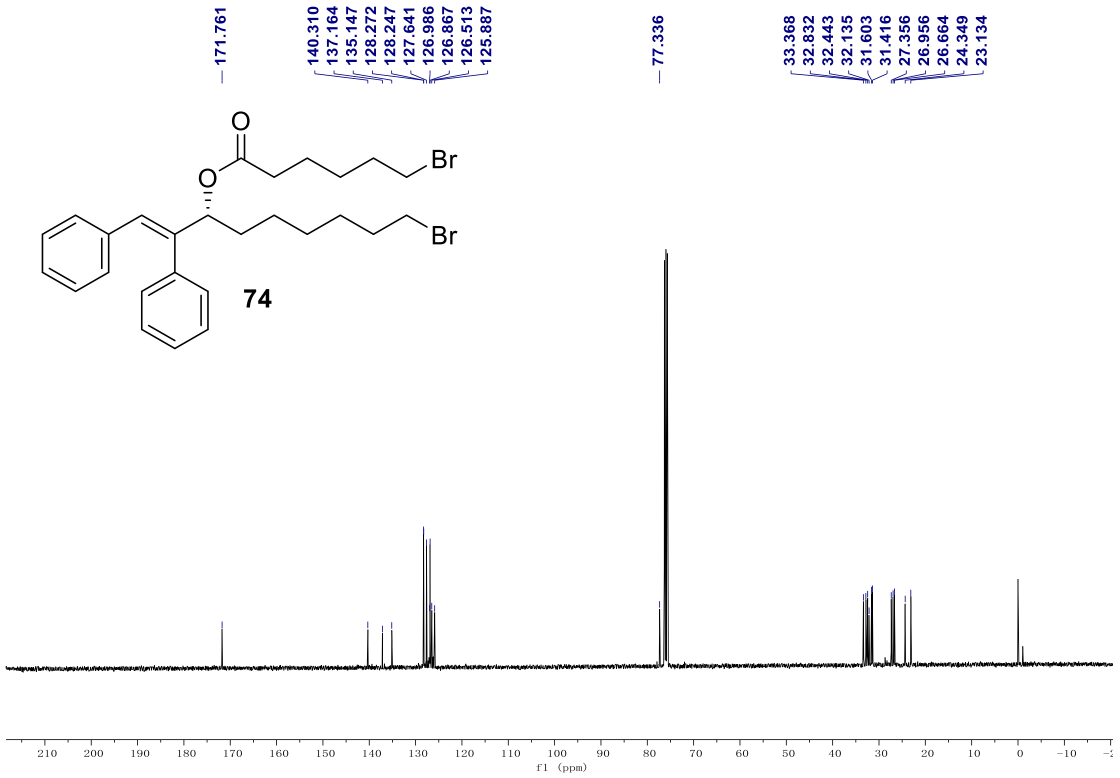

Supplementary Figure 236. ^{13}C NMR spectra of compound **74**

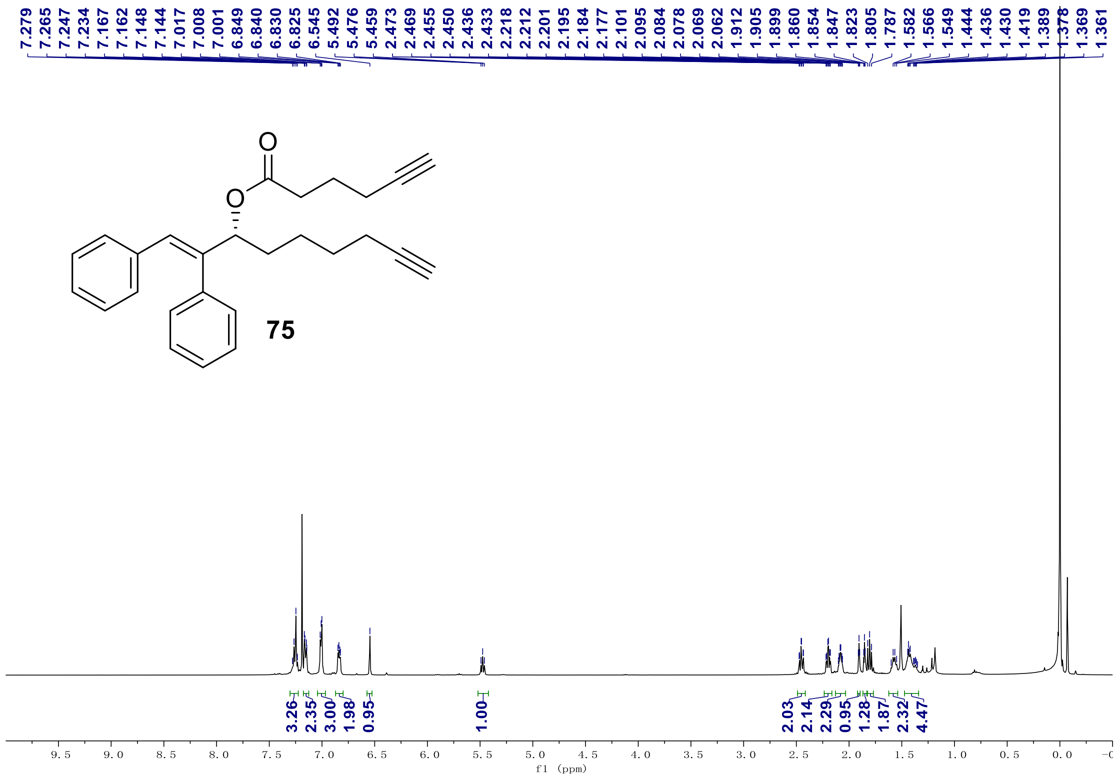

Supplementary Figure 237. ^{1}H NMR spectra of compound **75**

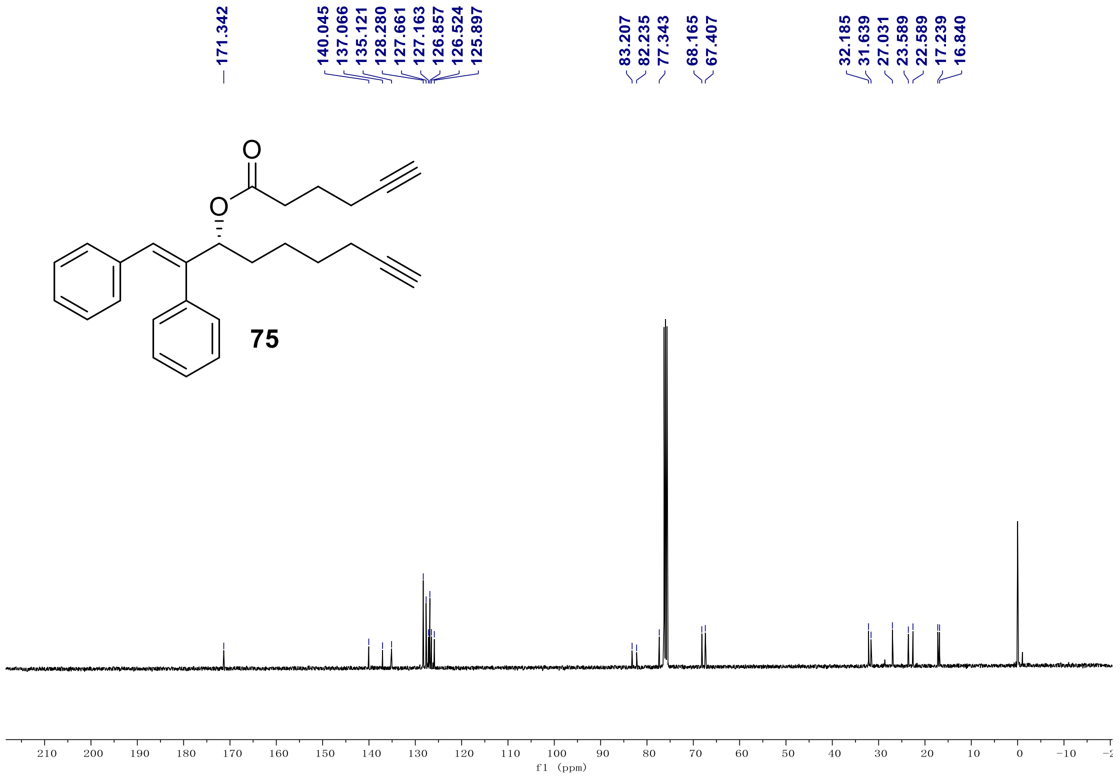

Supplementary Figure 238. ^{13}C NMR spectra of compound **75**

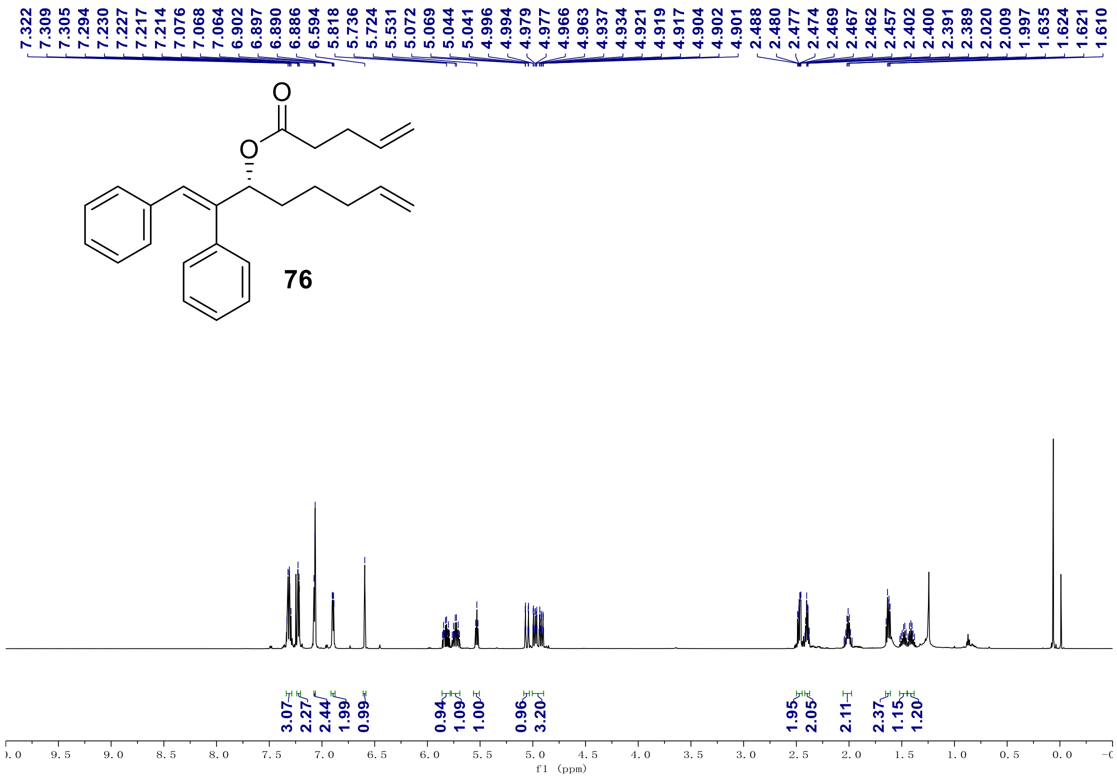

Supplementary Figure 239. ^{1}H NMR spectra of compound **76**

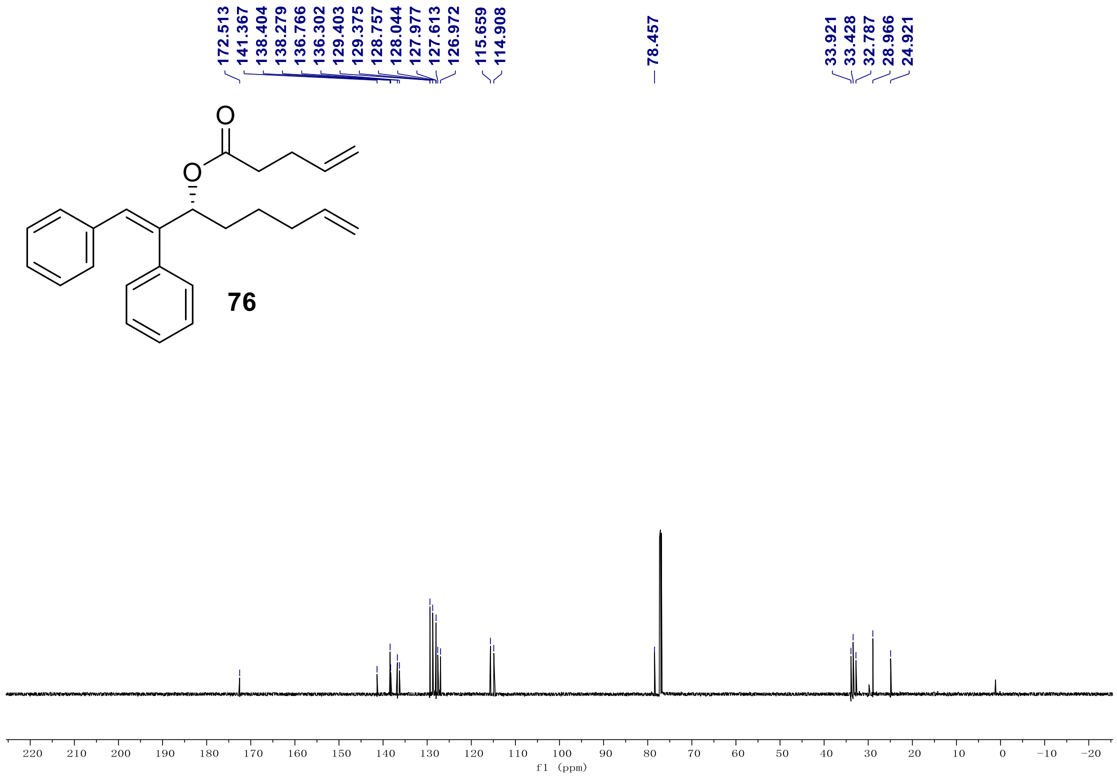

Supplementary Figure 240. ^{13}C NMR spectra of compound **77**

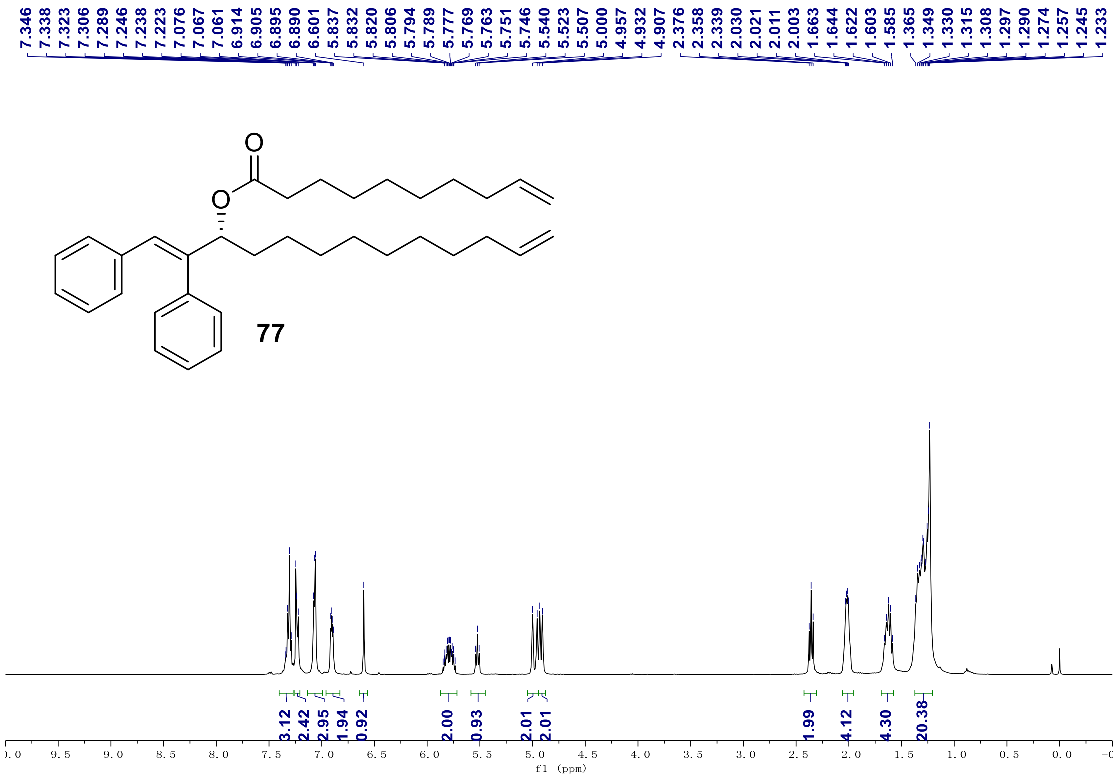

Supplementary Figure 241. ^{1}H NMR spectra of compound **77**

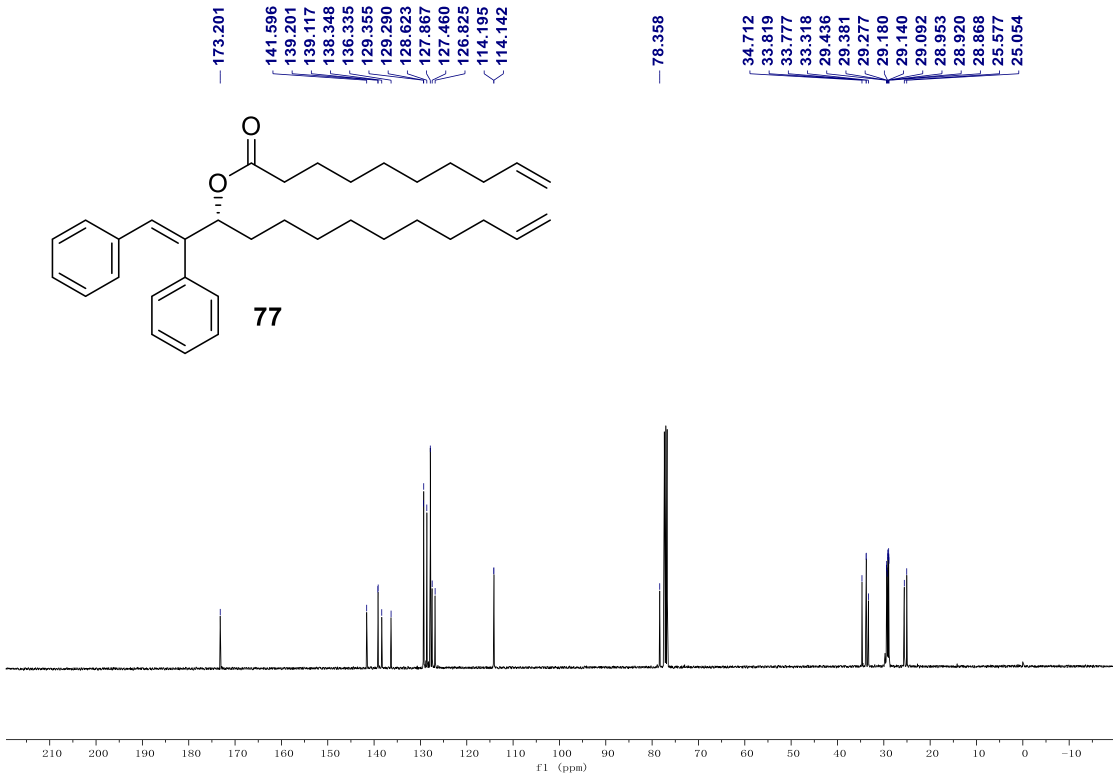

Supplementary Figure 242. ^{13}C NMR spectra of compound **77**

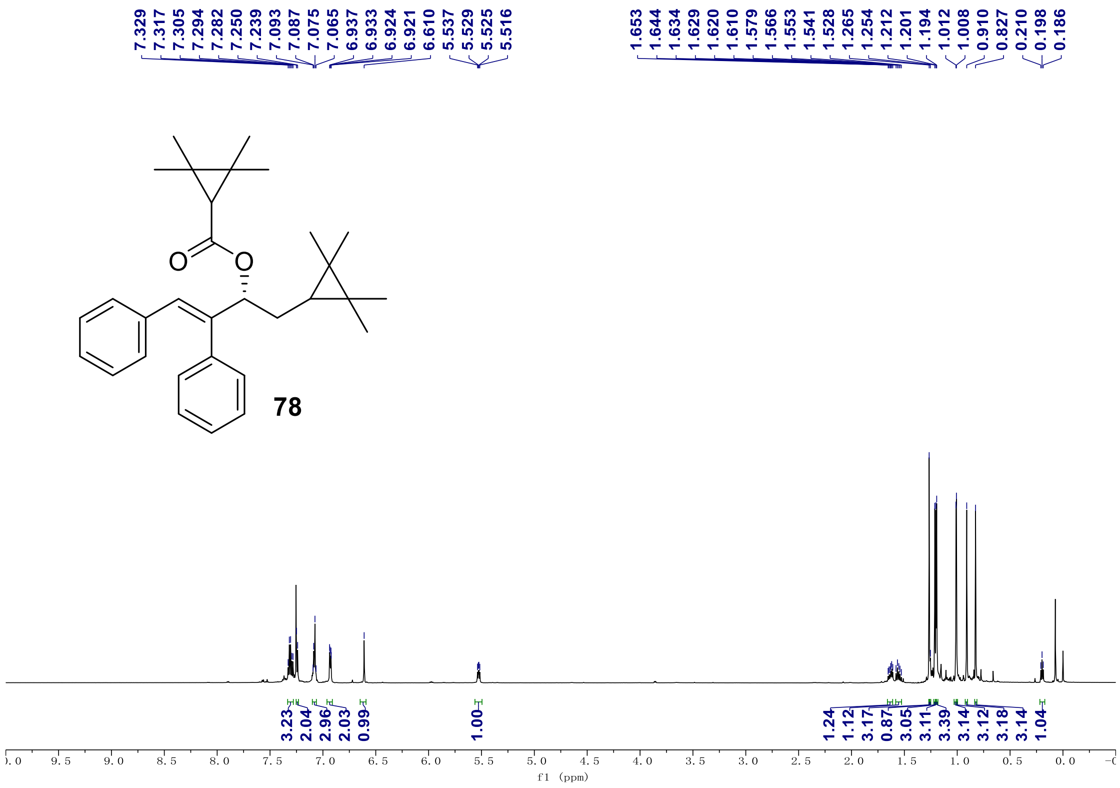

Supplementary Figure 243. ^{1}H NMR spectra of compound **78**

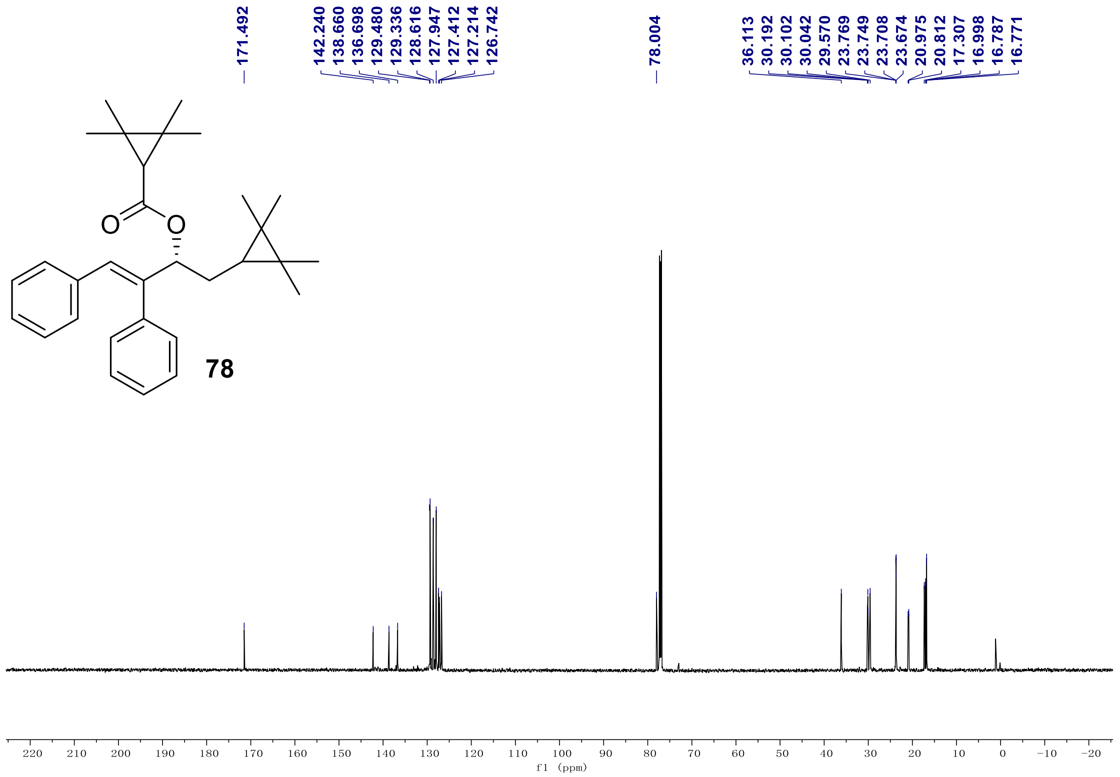

Supplementary Figure 244. ^{13}C NMR spectra of compound **78**

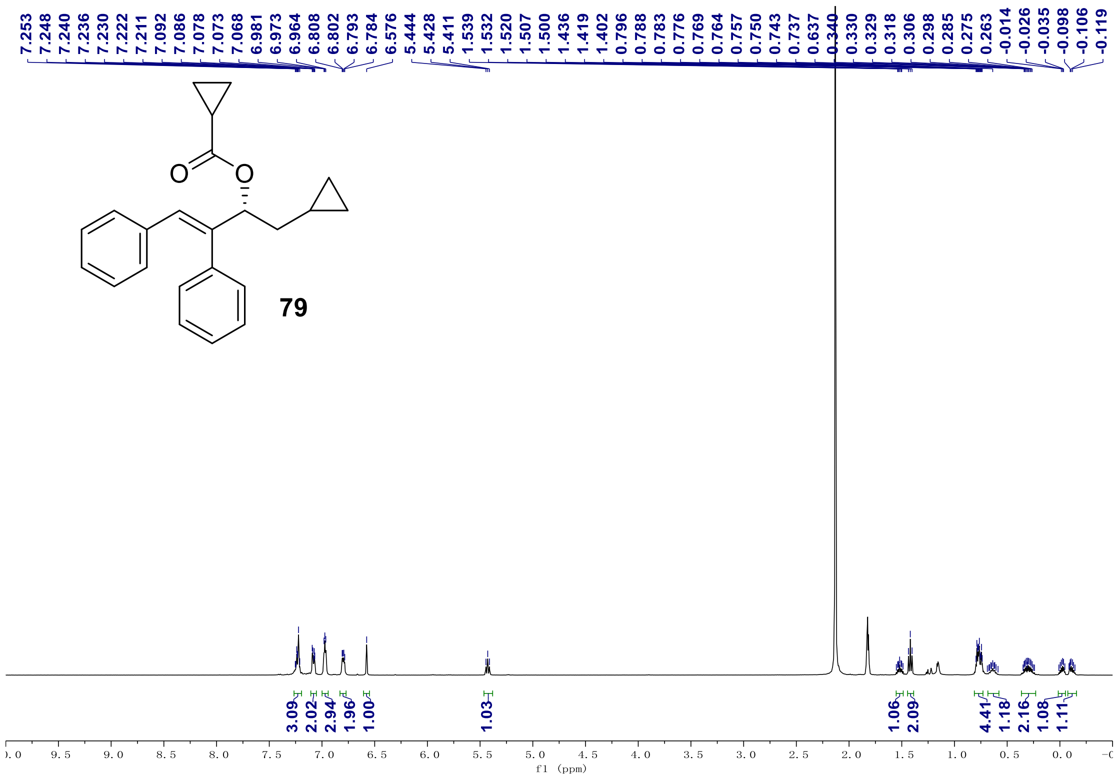

Supplementary Figure 245. ^{1}H NMR spectra of compound 79

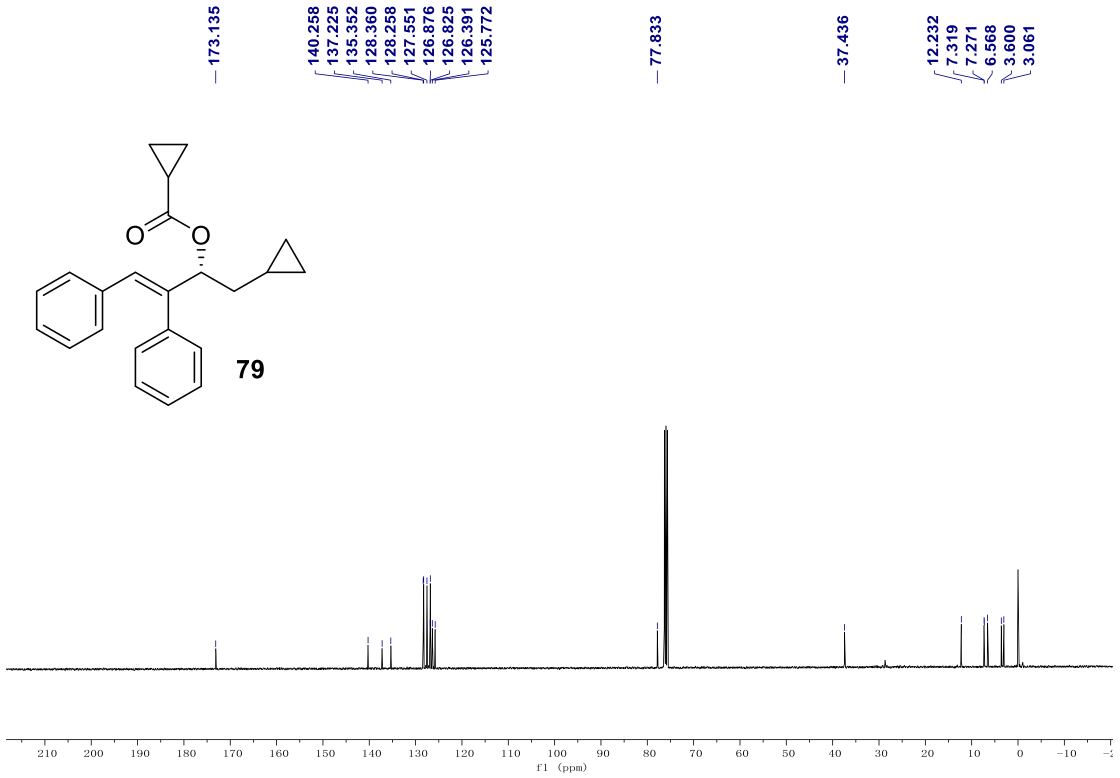

Supplementary Figure 246. ^{13}C NMR spectra of compound 79

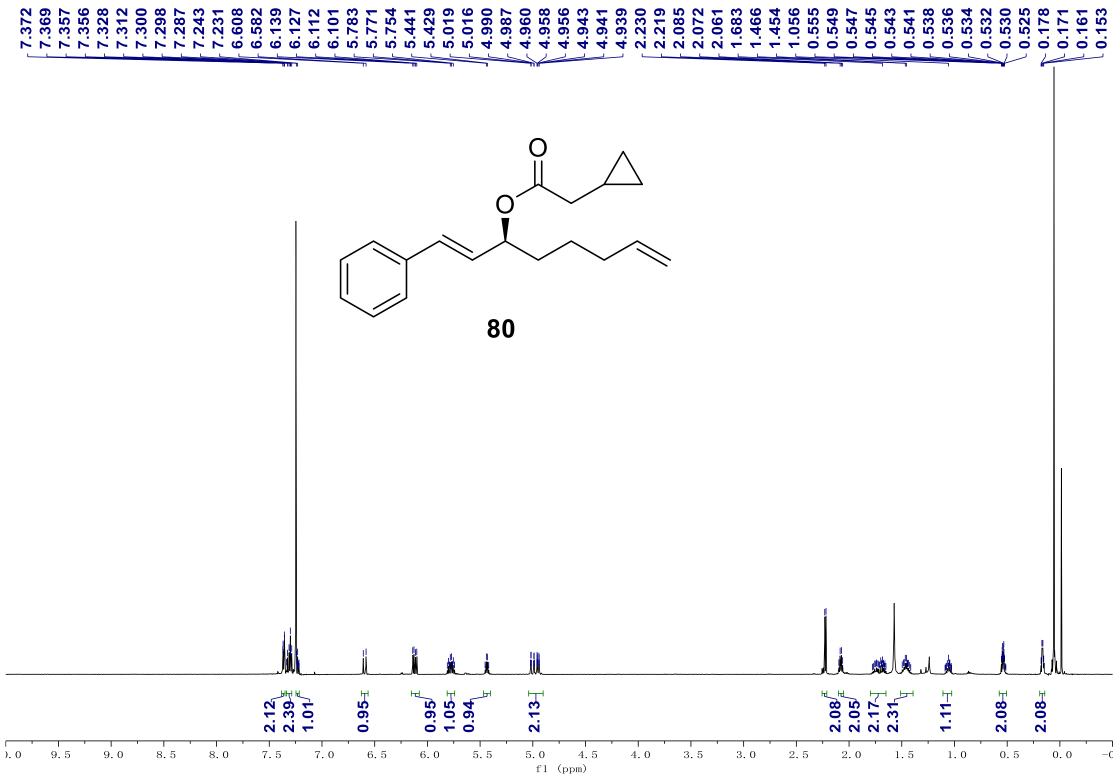

Supplementary Figure 247. ^{1}H NMR spectra of compound **80**

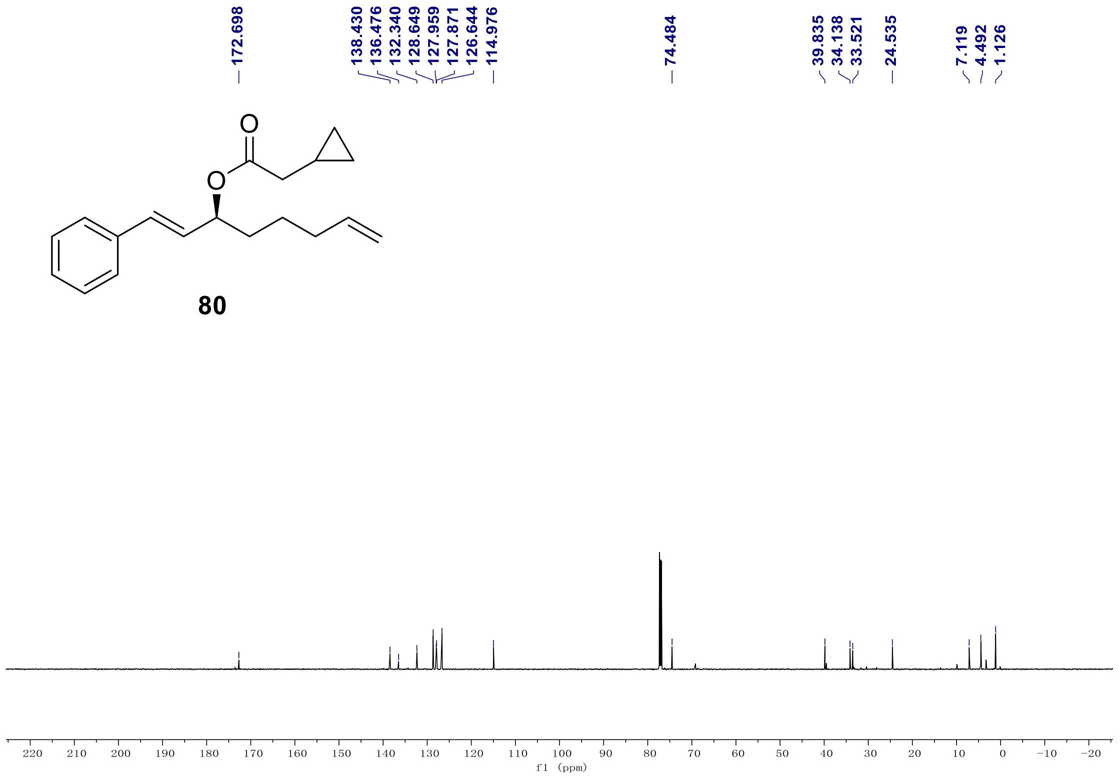

Supplementary Figure 248. ^{13}C NMR spectra of compound **80**

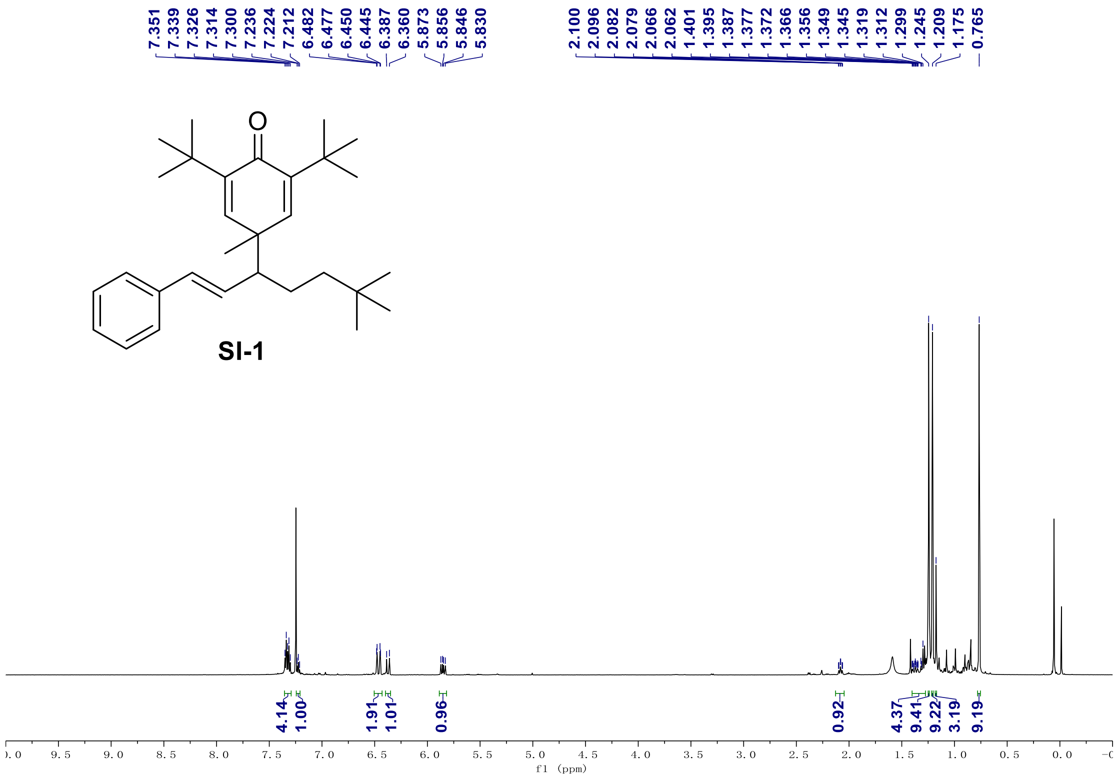

Supplementary Figure 249. ^{1}H NMR spectra of compound **SI-1**

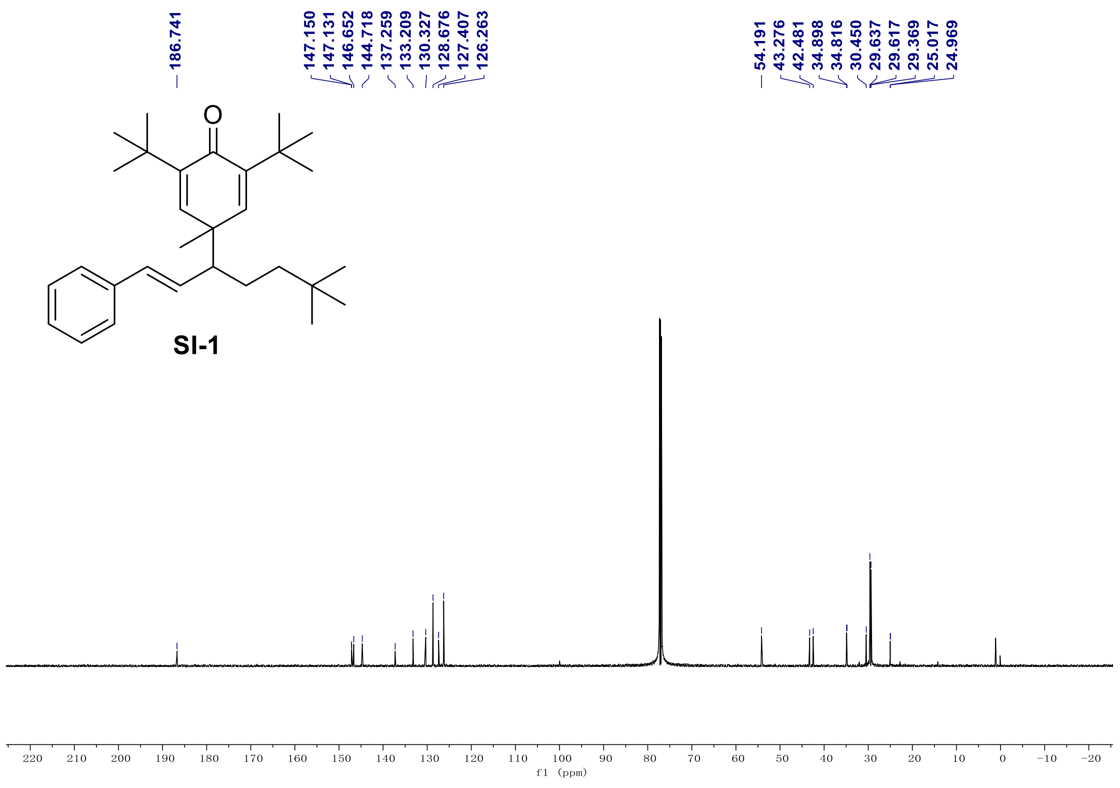

Supplementary Figure 250. ^{13}C NMR spectra of compound **SI-1**

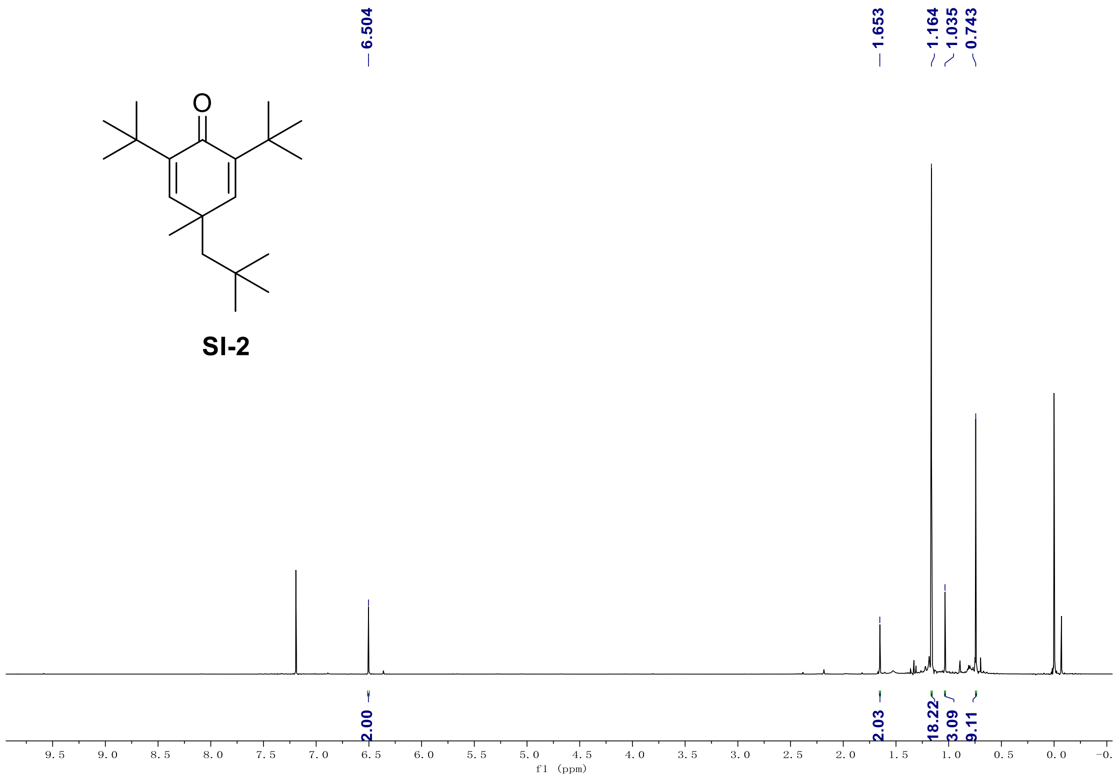

Supplementary Figure 251. ^{1}H NMR spectra of compound **SI-2**

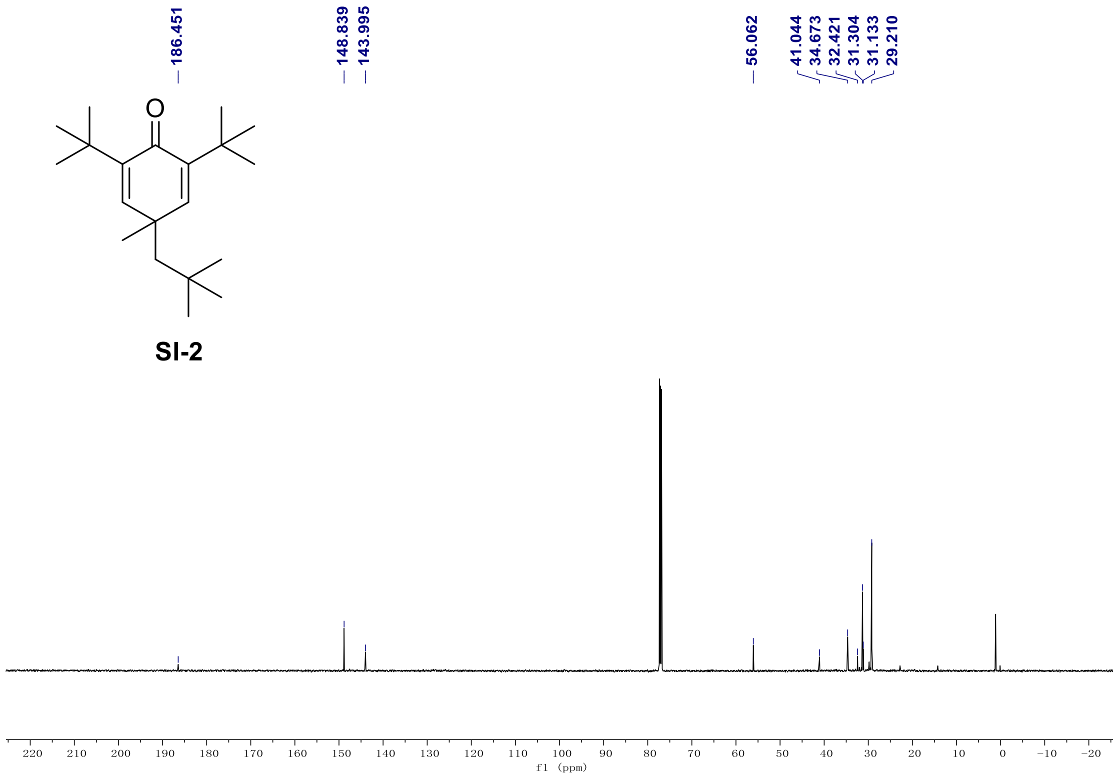

Supplementary Figure 252. ^{13}C NMR spectra of compound **SI-2**

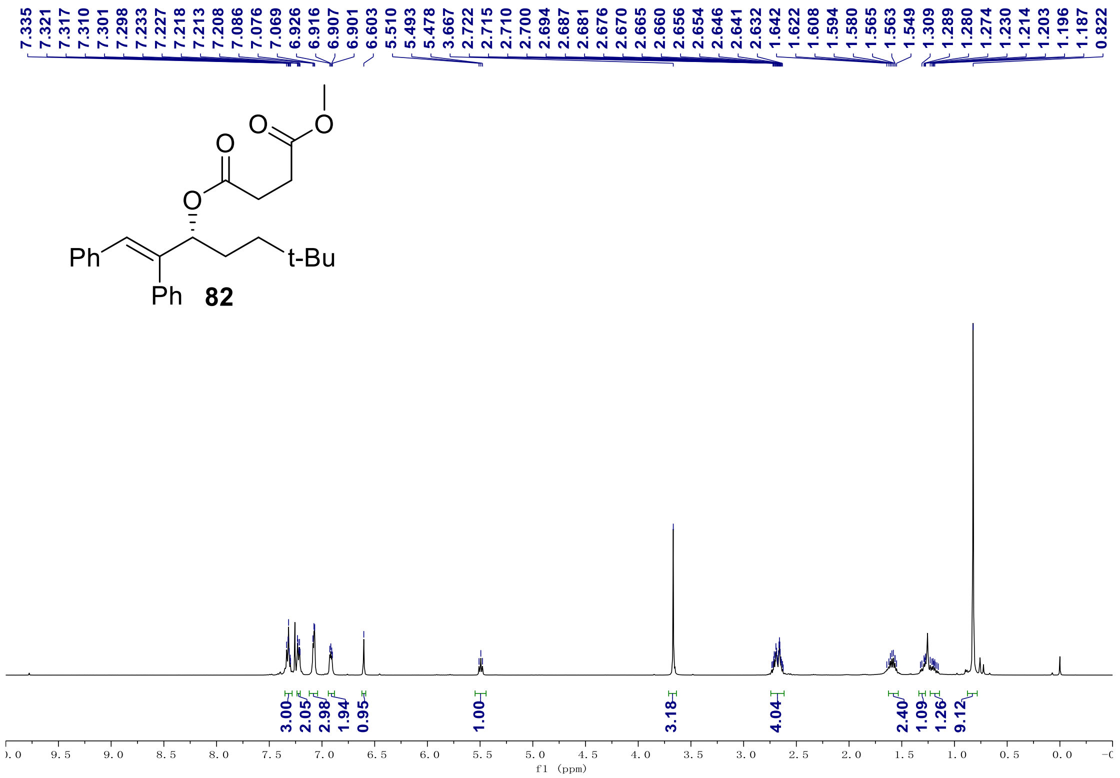

Supplementary Figure 253. ^{1}H NMR spectra of compound **82**

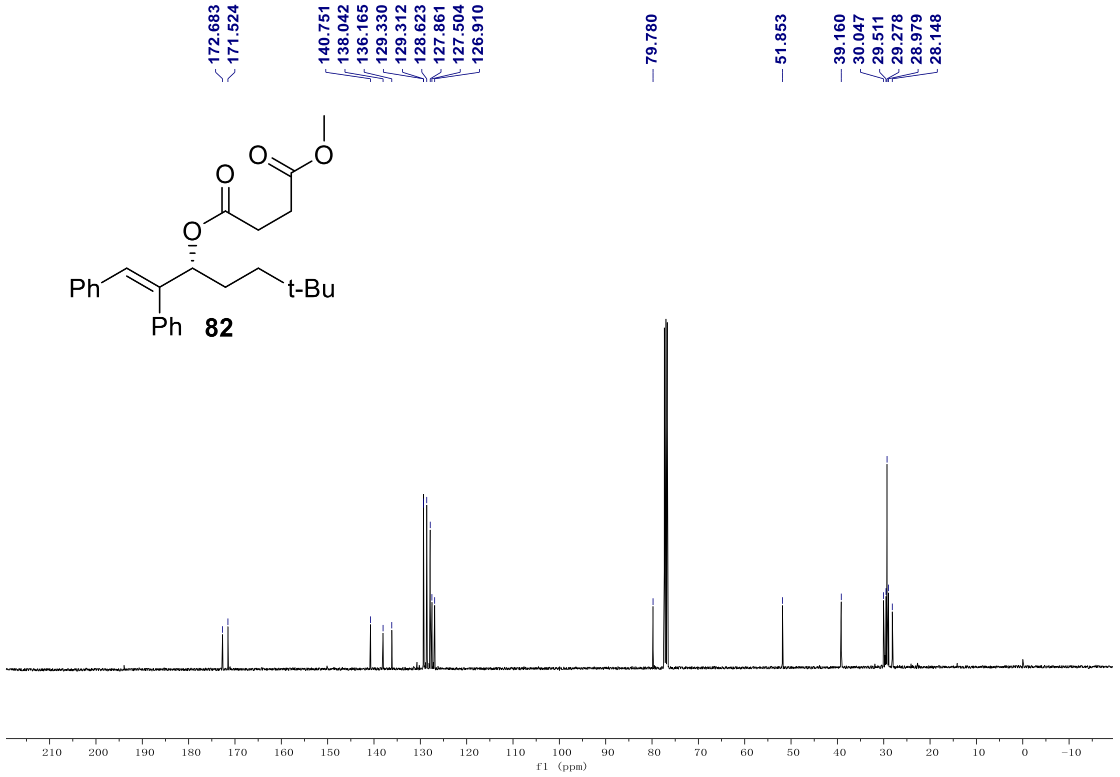

Supplementary Figure 254. ^{13}C NMR spectra of compound **82**

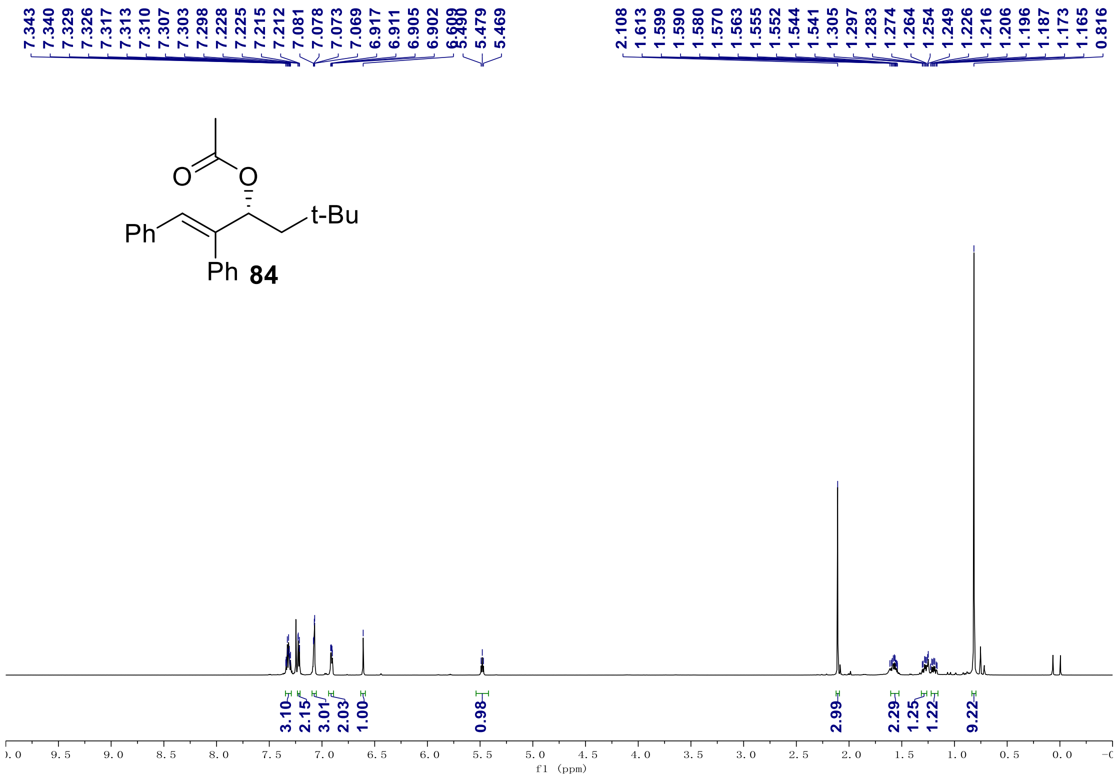

Supplementary Figure 255. ^{1}H NMR spectra of compound **84**

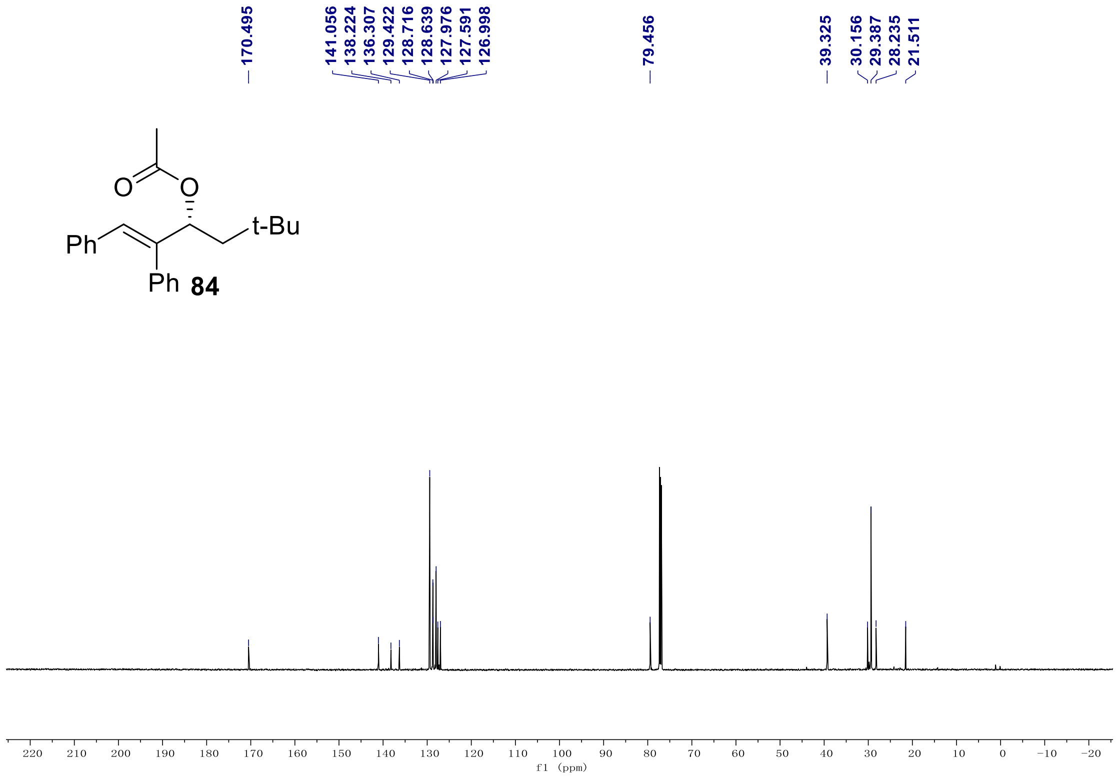

Supplementary Figure 256. ^{13}C NMR spectra of compound **84**

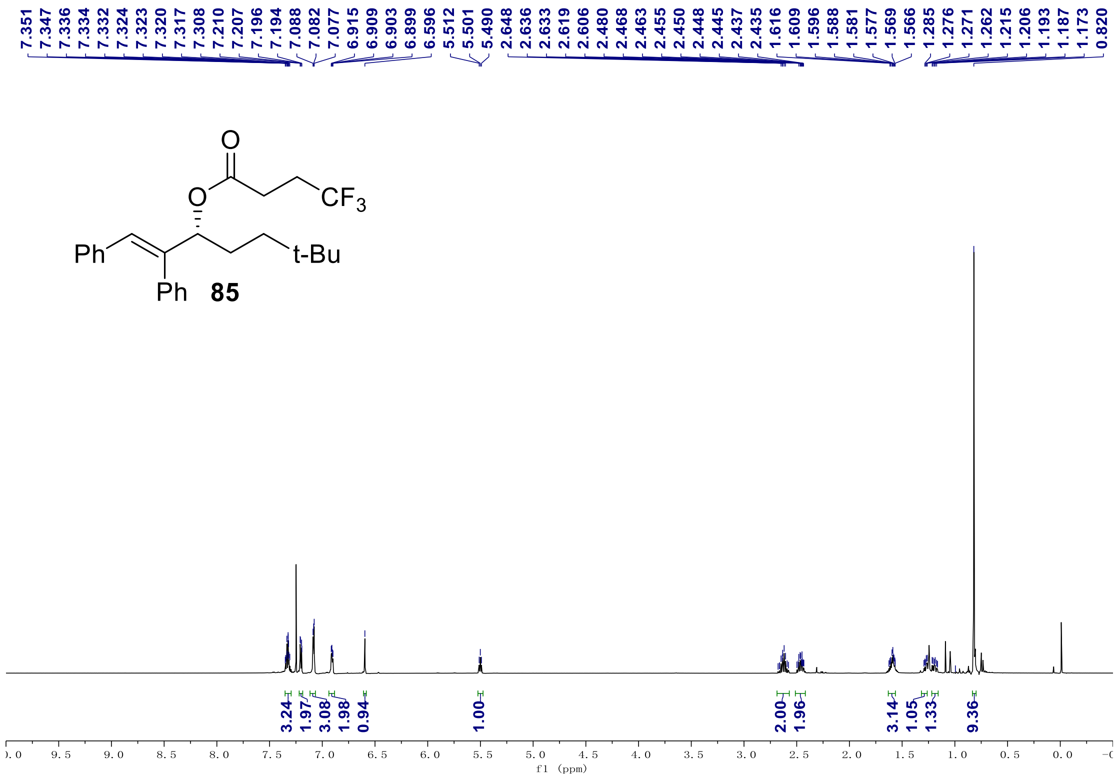

Supplementary Figure 257. ^{1}H NMR spectra of compound **85**

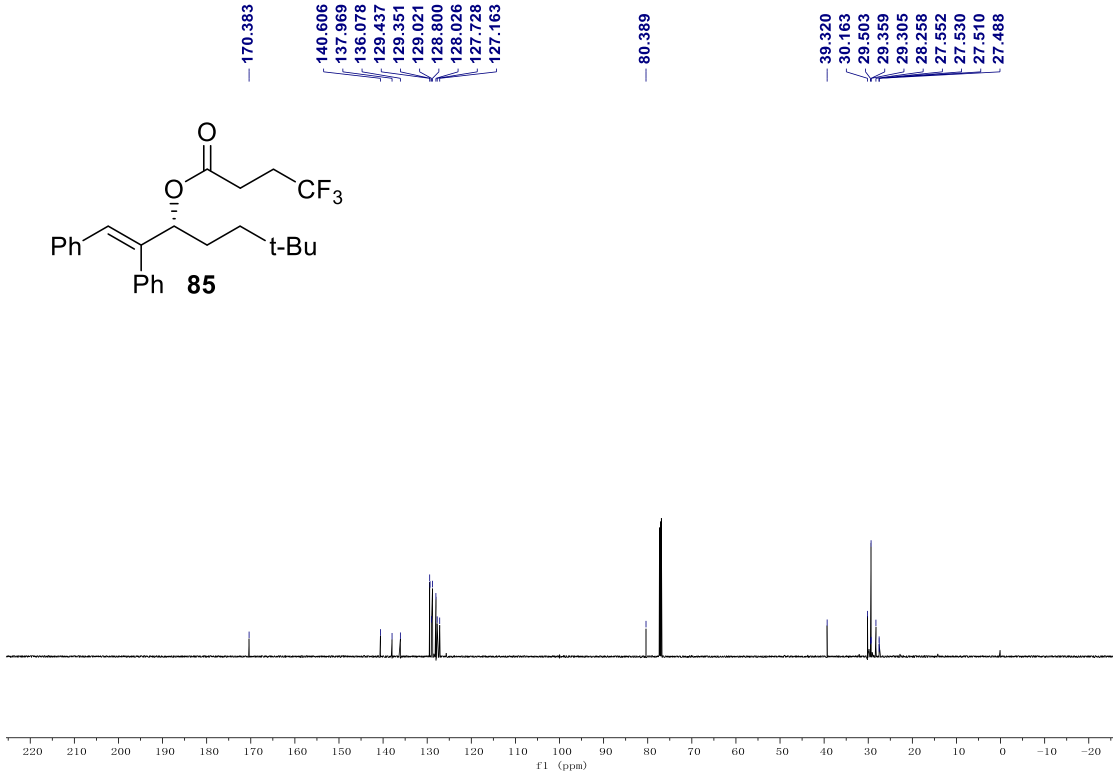

Supplementary Figure 258. ^{13}C NMR spectra of compound **85**

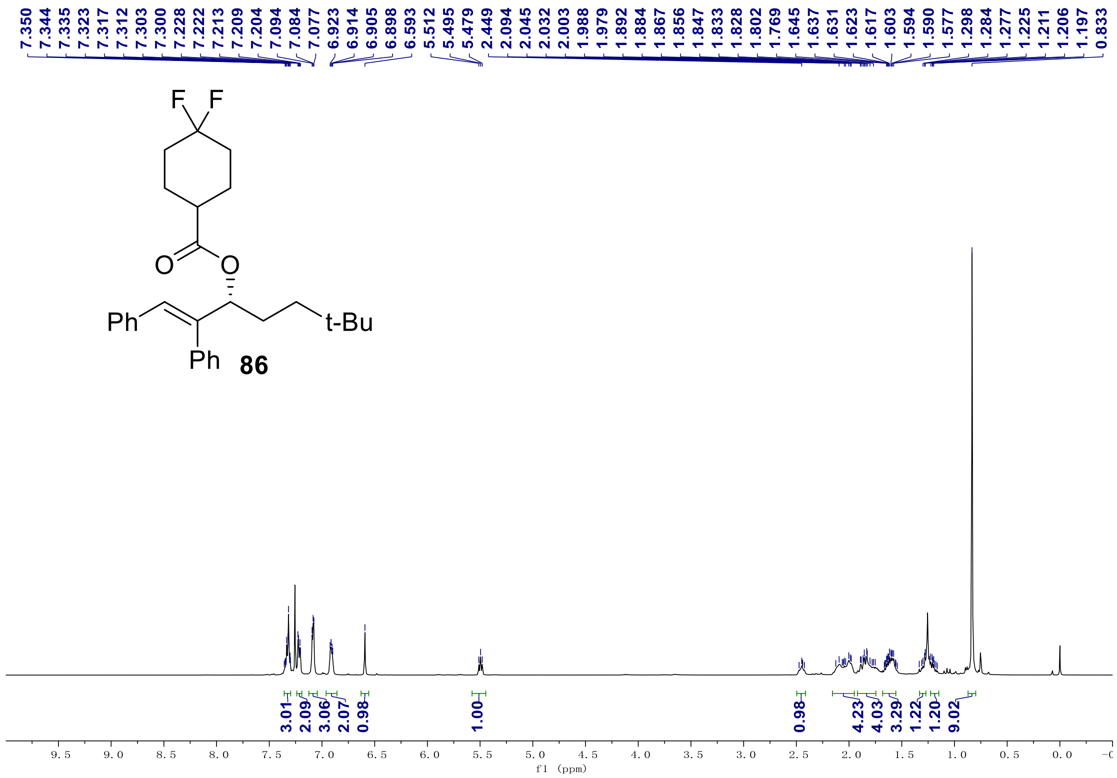

Supplementary Figure 259. ^{1}H NMR spectra of compound **86**

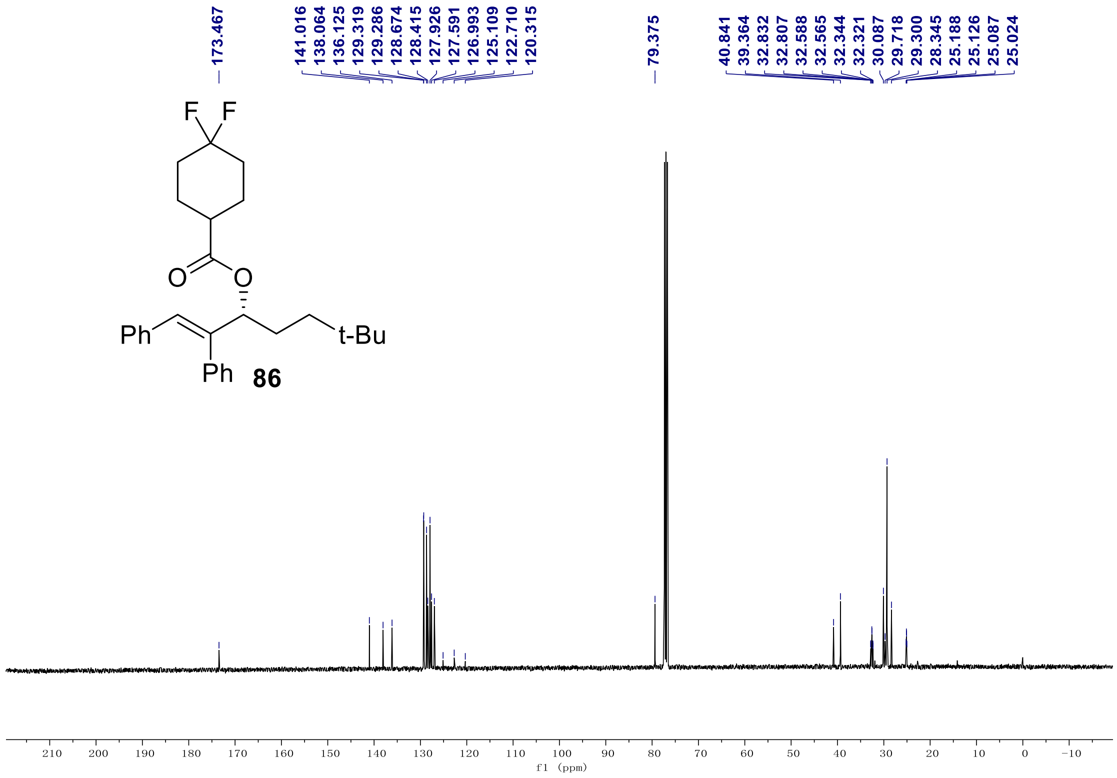

Supplementary Figure 260. ^{13}C NMR spectra of compound **86**

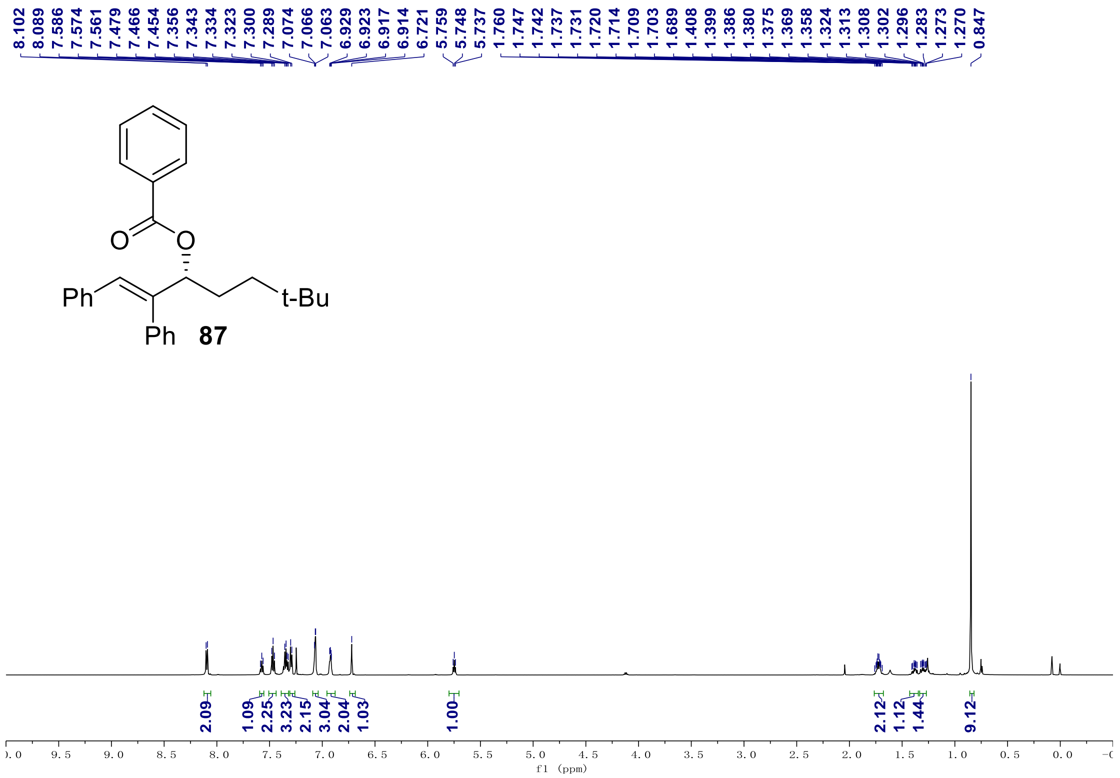

Supplementary Figure 261. ^{1}H NMR spectra of compound **87**

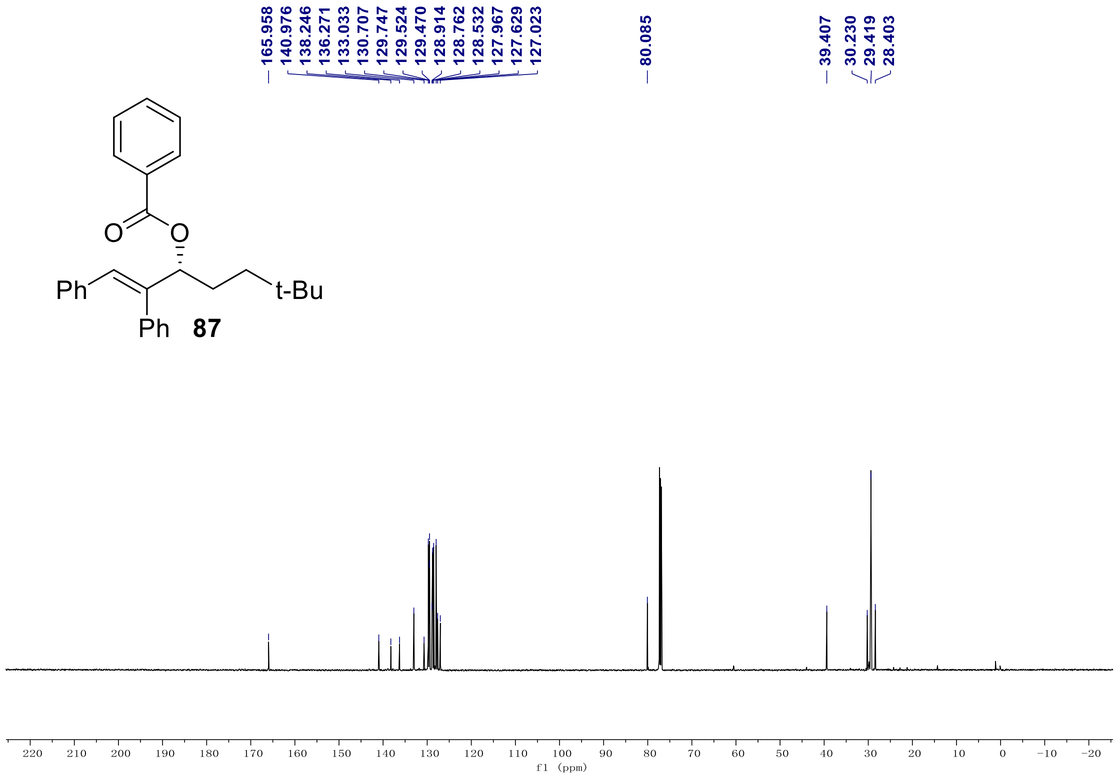

Supplementary Figure 262. ^{13}C NMR spectra of compound **87**

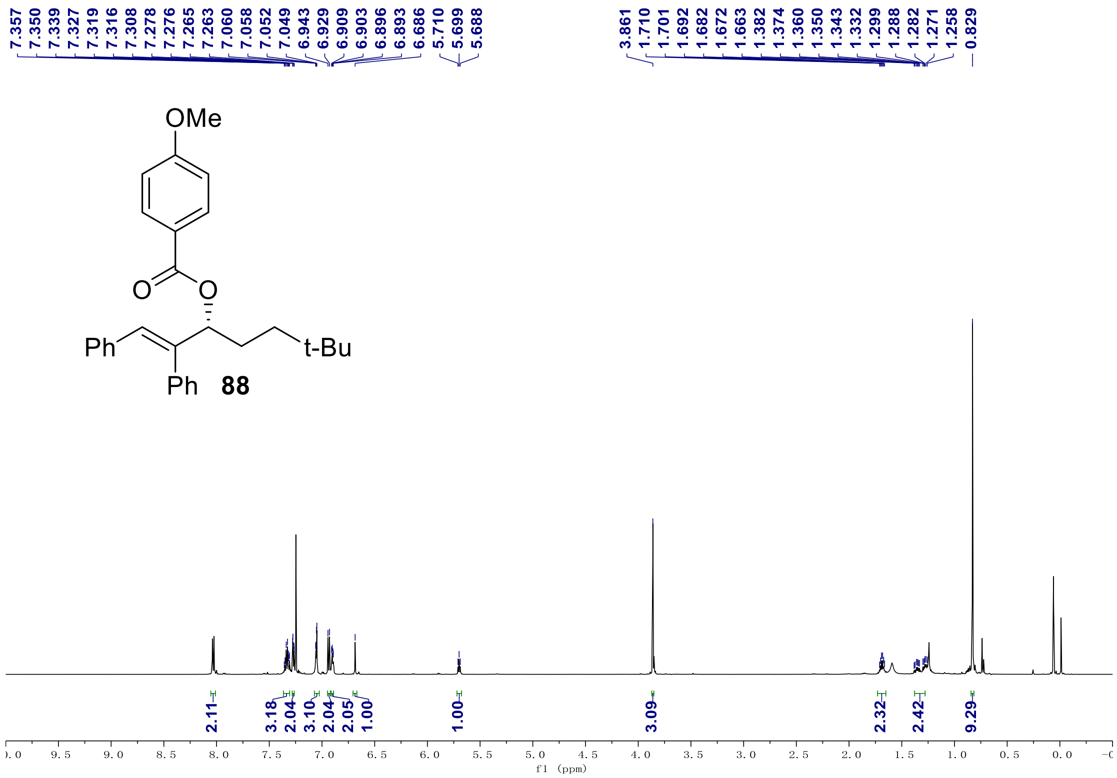

Supplementary Figure 263. ^{1}H NMR spectra of compound **88**

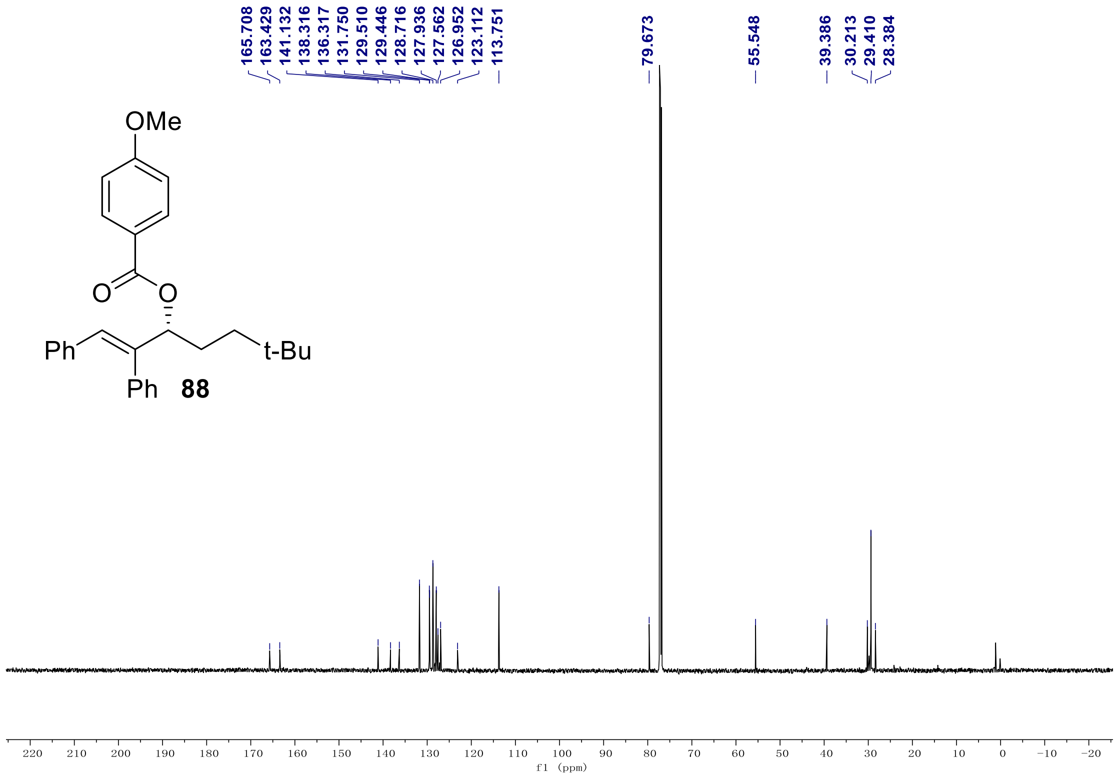

Supplementary Figure 264. ^{13}C NMR spectra of compound **88**

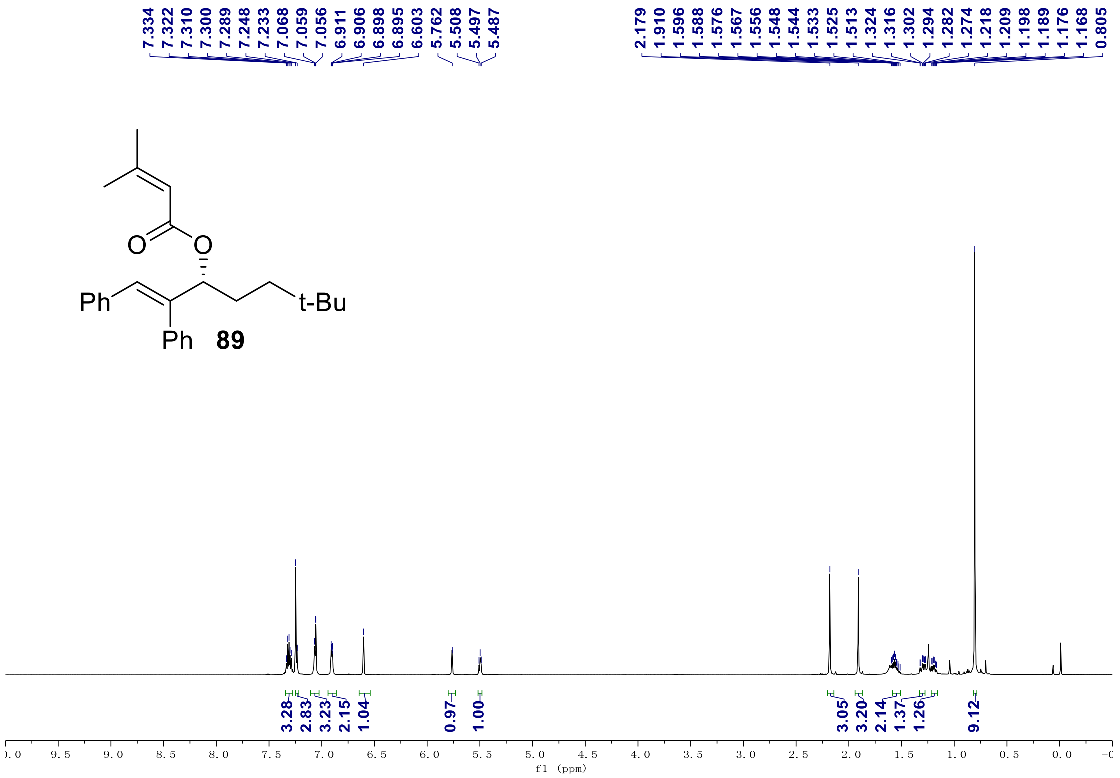

Supplementary Figure 265. ^{1}H NMR spectra of compound **89**

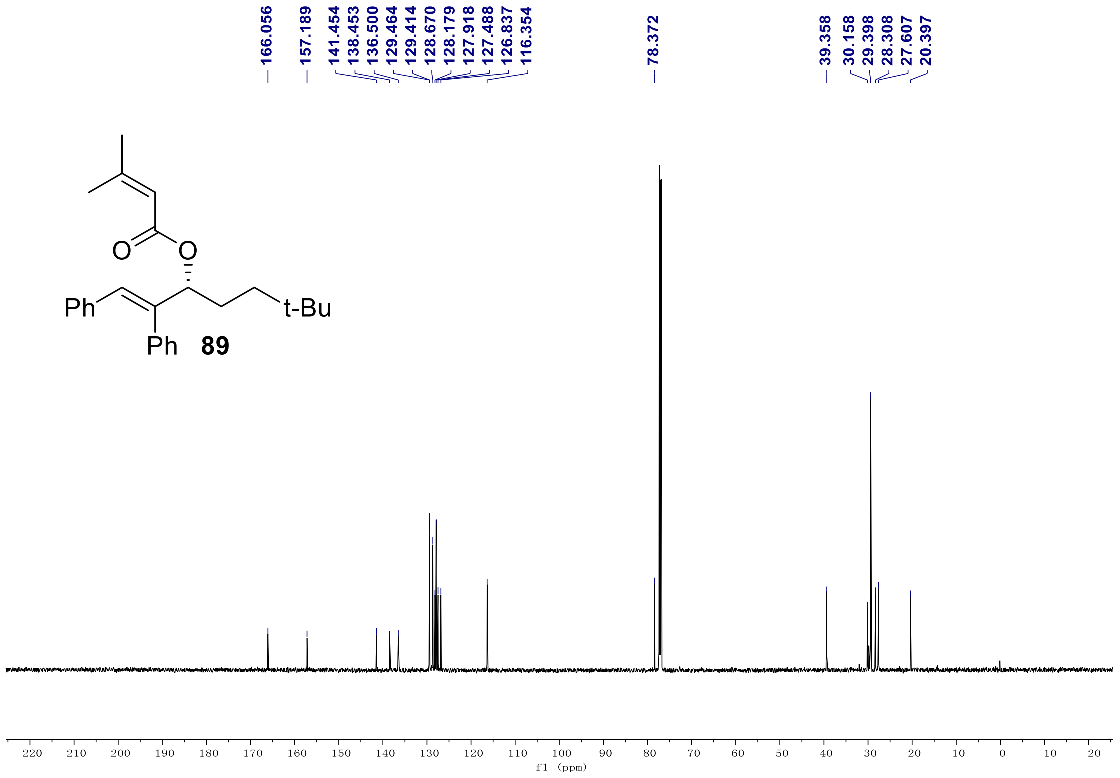

Supplementary Figure 266. ^{13}C NMR spectra of compound **89**

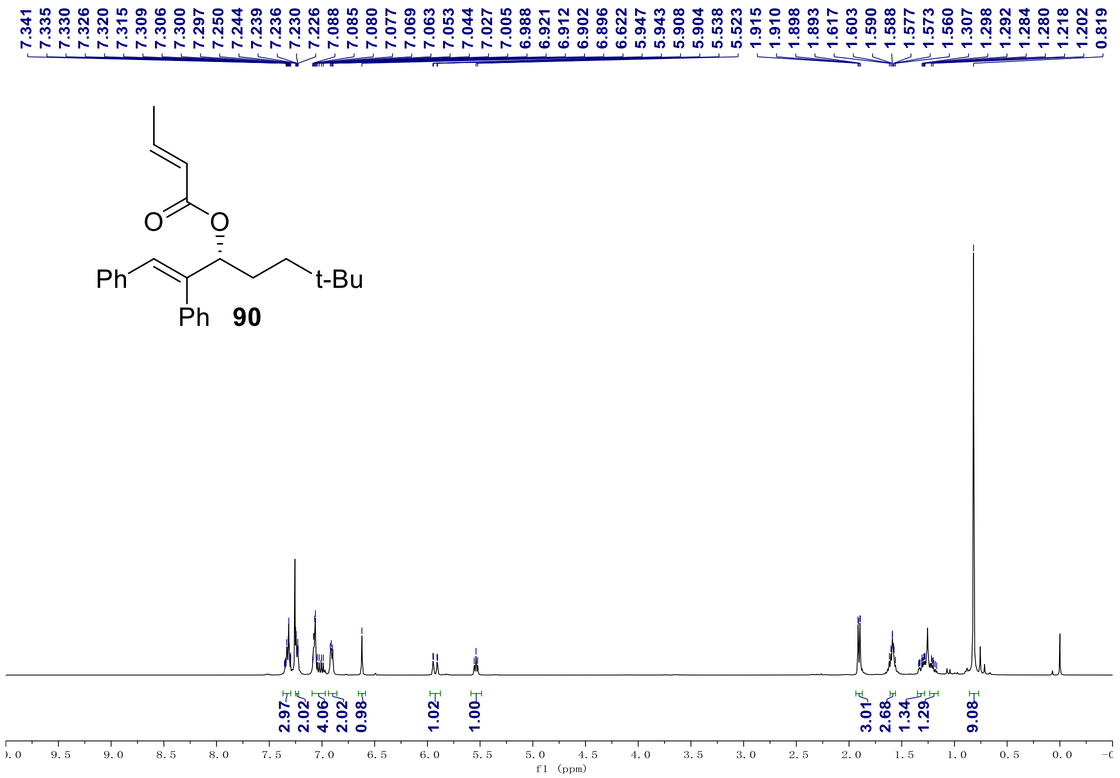

Supplementary Figure 267. ^{1}H NMR spectra of compound **90**

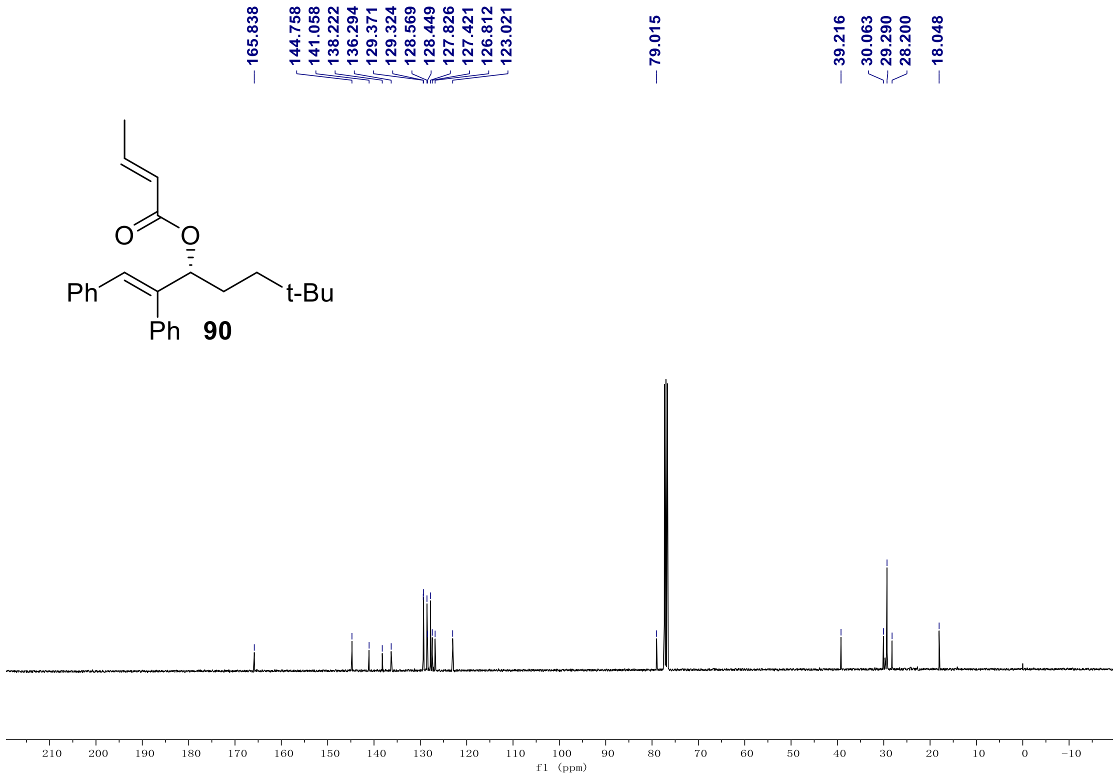

Supplementary Figure 268. ^{13}C NMR spectra of compound **90**

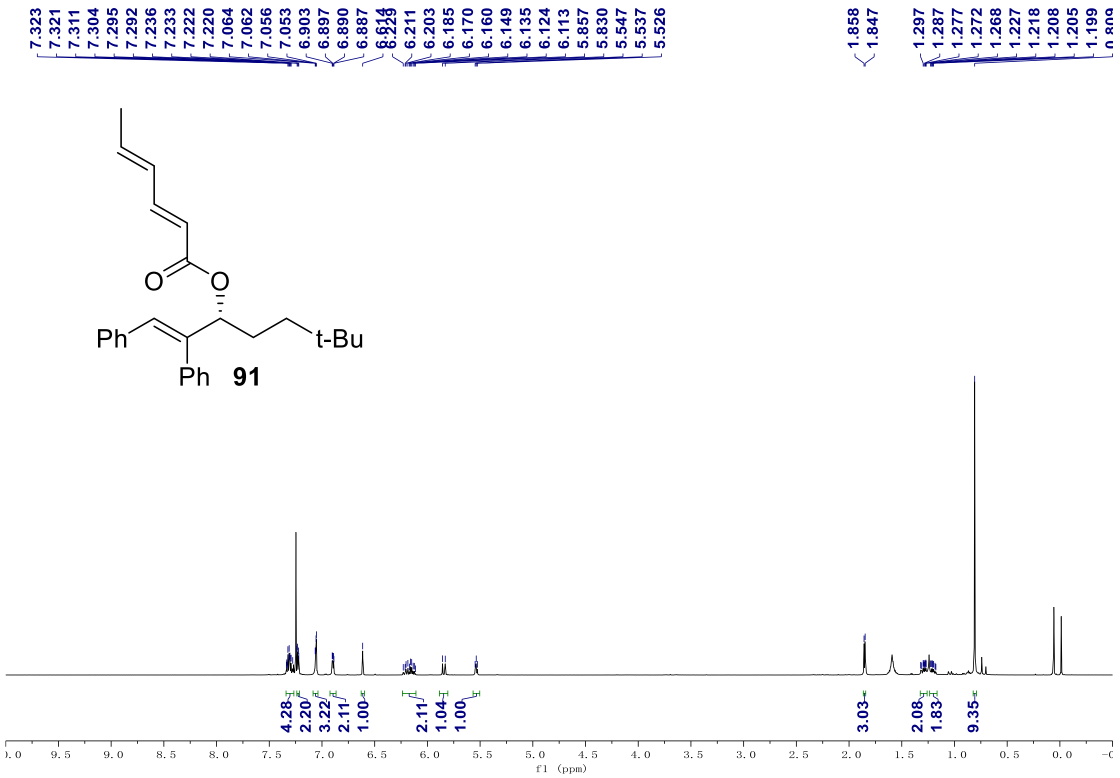

Supplementary Figure 269. ^{1}H NMR spectra of compound **91**

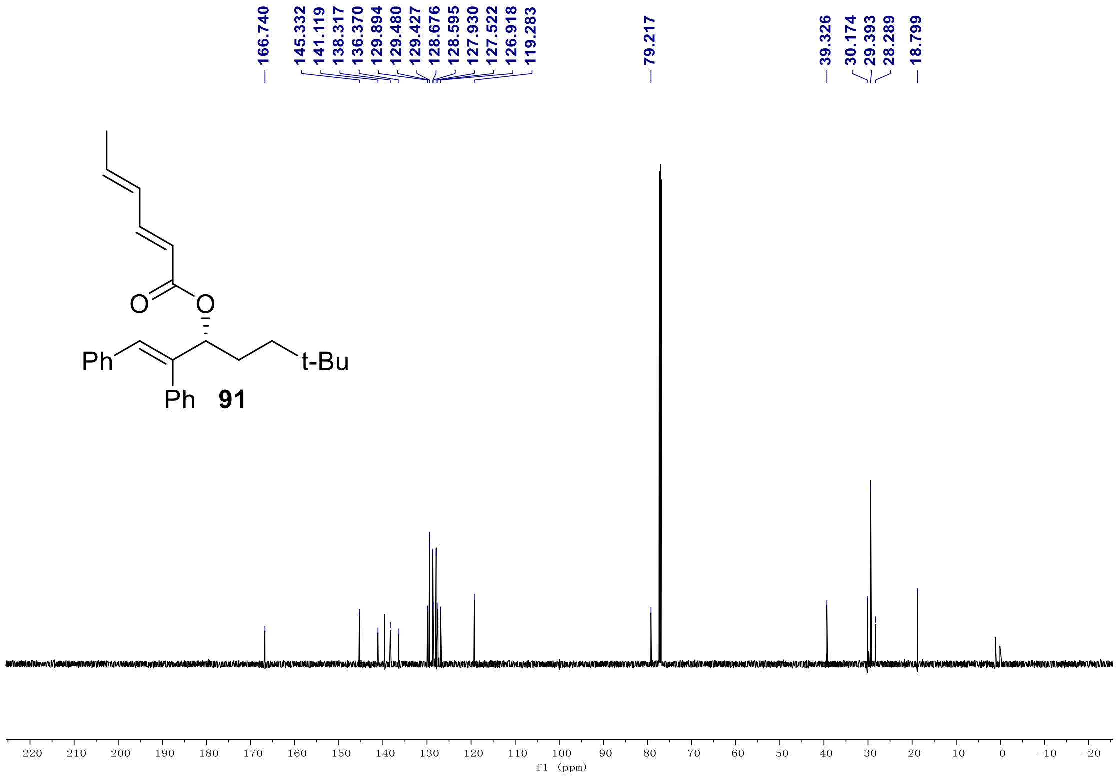

Supplementary Figure 270. ^{13}C NMR spectra of compound **91**

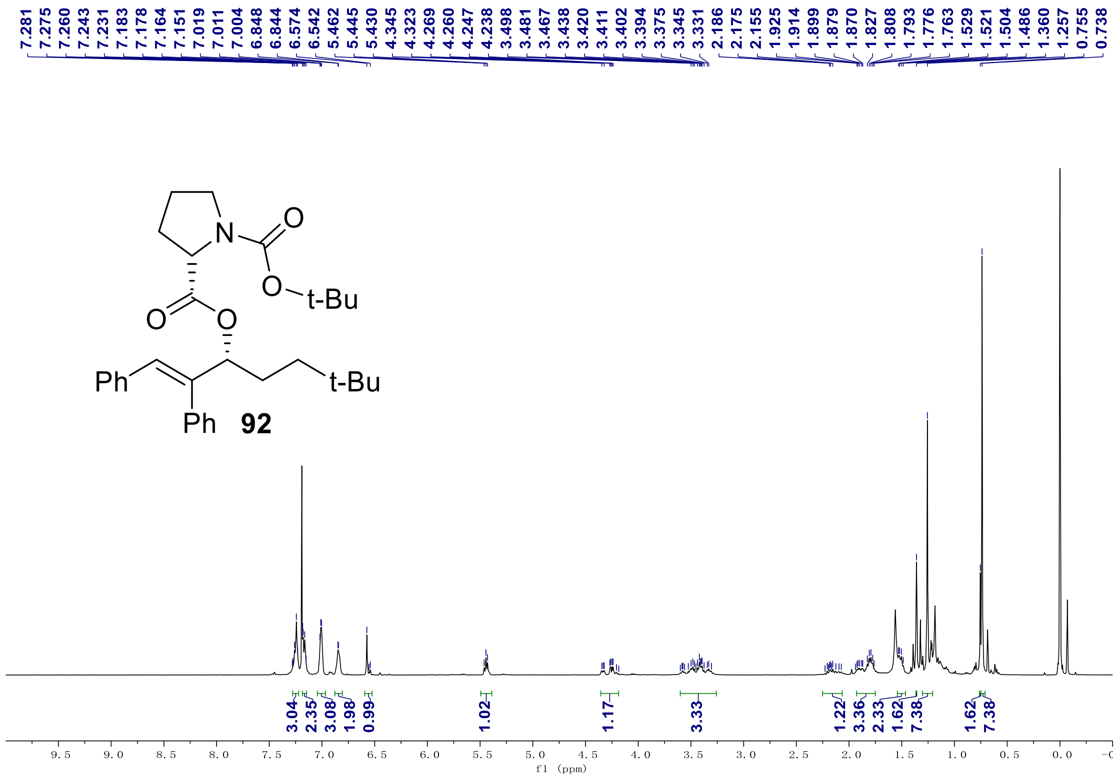

Supplementary Figure 271. ^{1}H NMR spectra of compound **92**

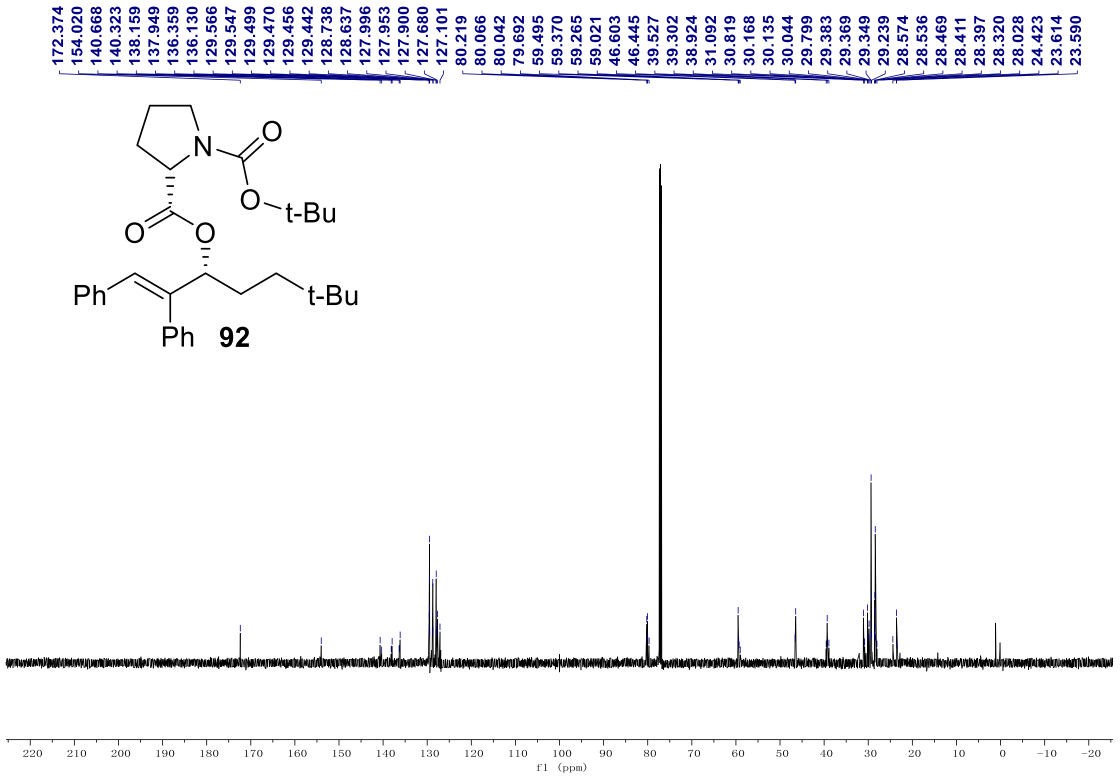

Supplementary Figure 272. ^{13}C NMR spectra of compound **92**

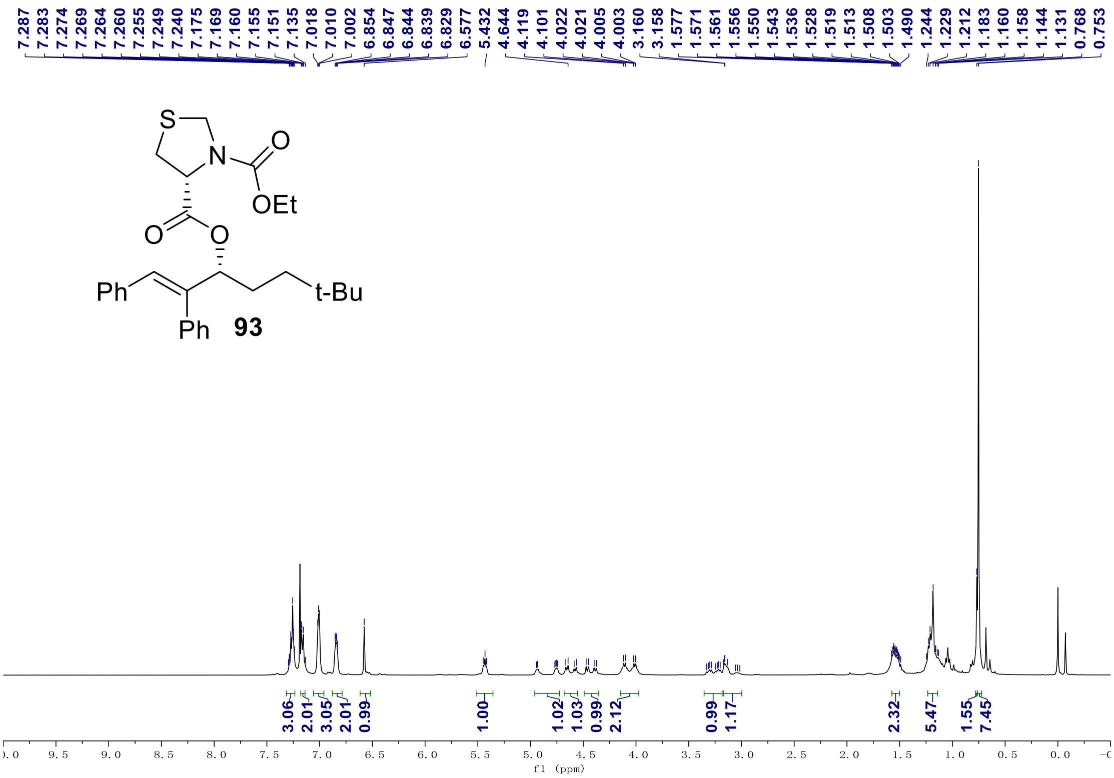

Supplementary Figure 273. ¹H NMR spectra of compound **93**

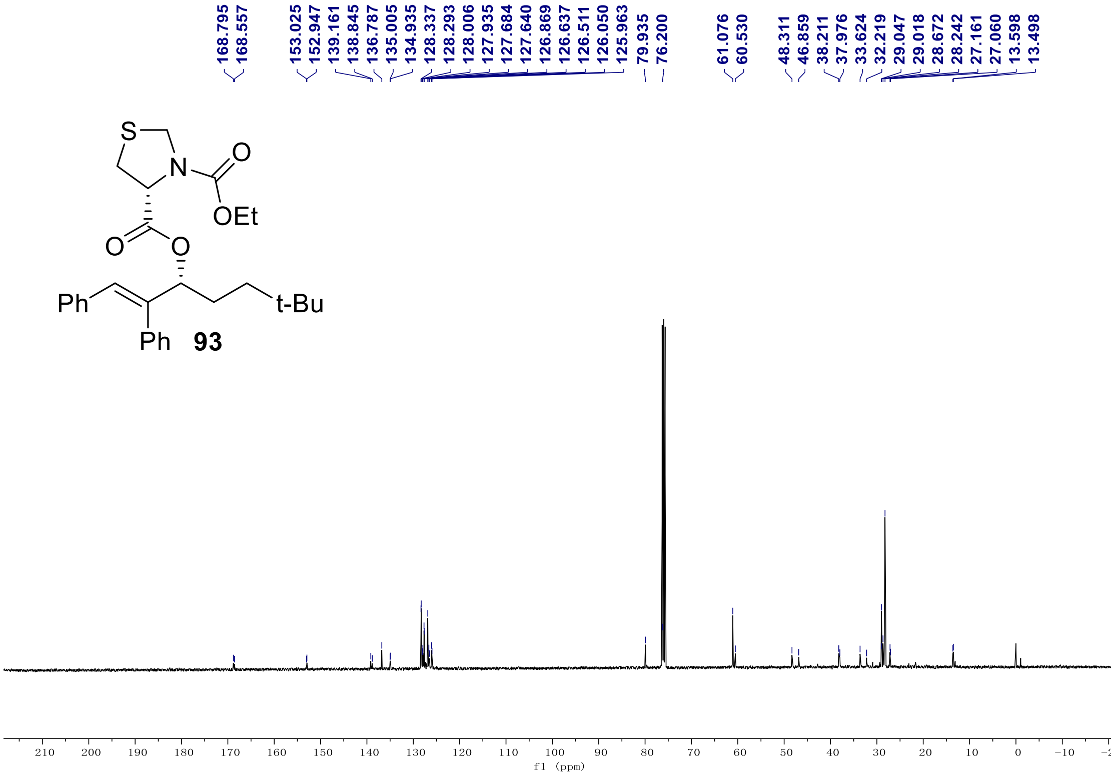

Supplementary Figure 274. ¹³C NMR spectra of compound **93**

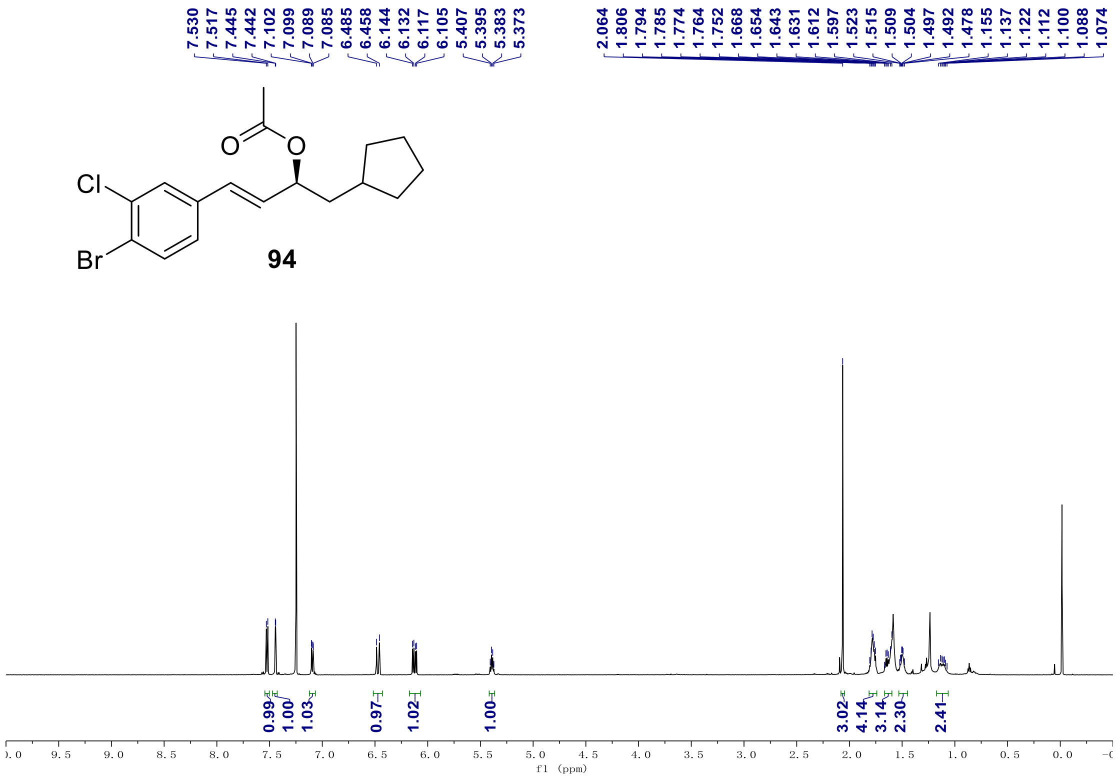

Supplementary Figure 275. ^{1}H NMR spectra of compound **94**

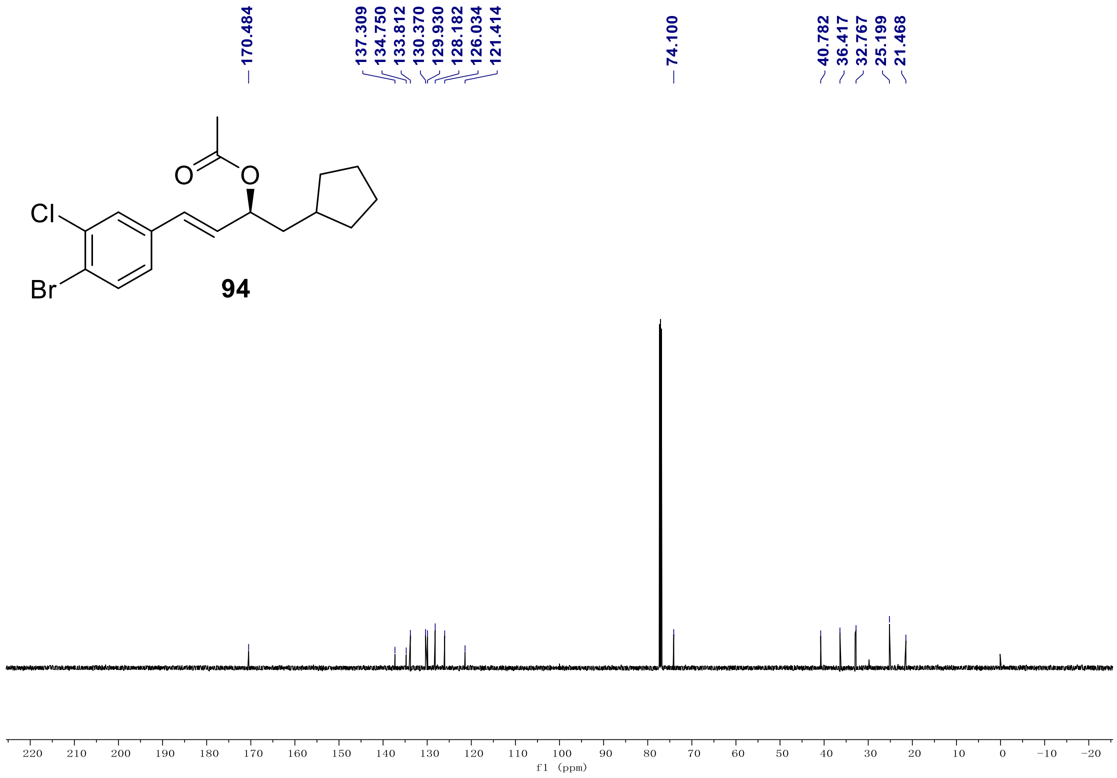

Supplementary Figure 276. ^{13}C NMR spectra of compound **94**

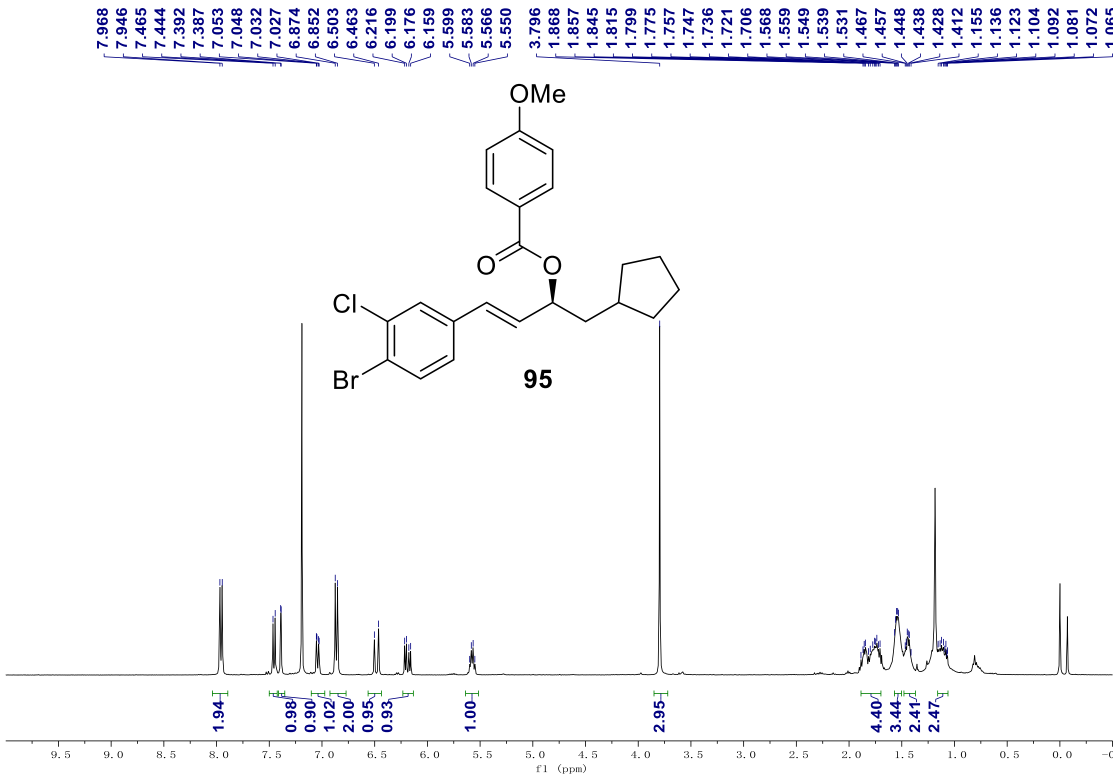

Supplementary Figure 277. ^{1}H NMR spectra of compound **95**

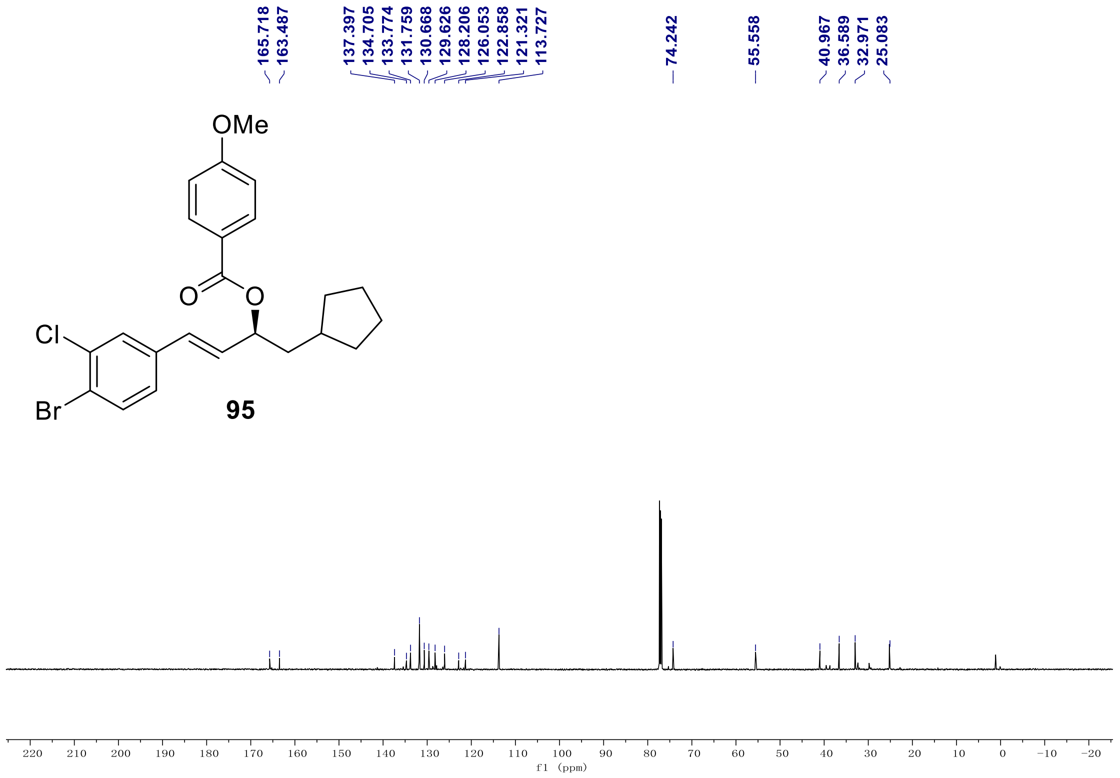

Supplementary Figure 278. ^{13}C NMR spectra of compound **95**

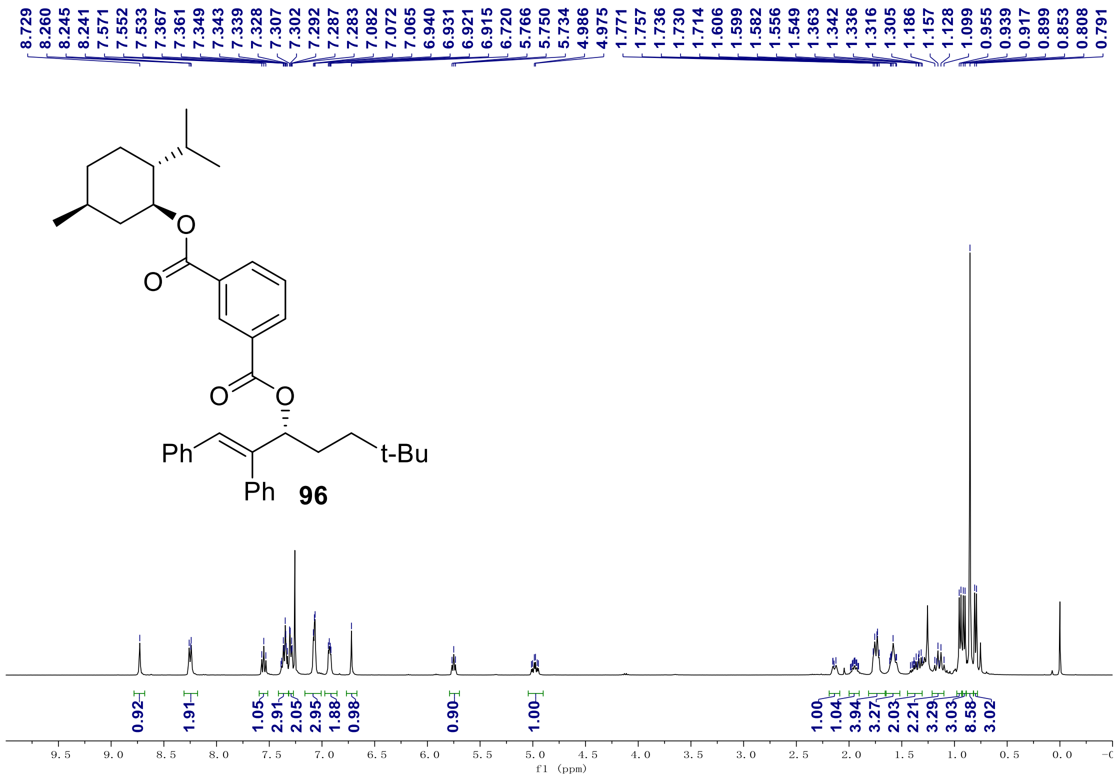

Supplementary Figure 279. ^{1}H NMR spectra of compound **96**

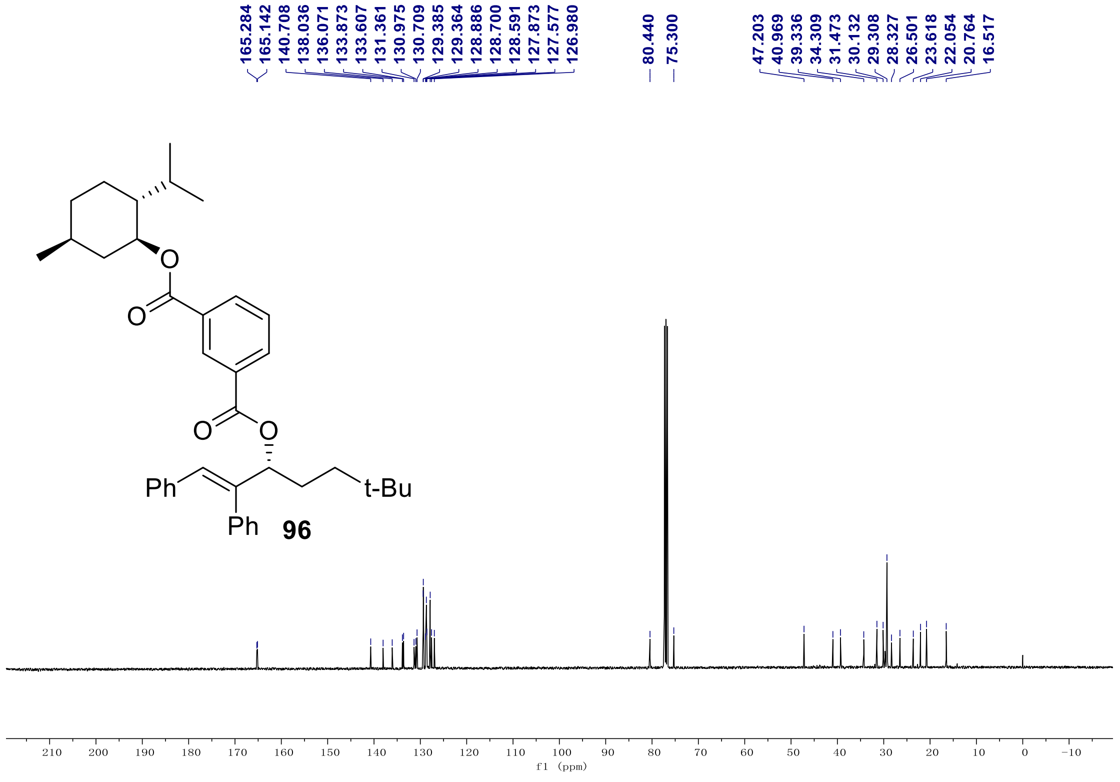

Supplementary Figure 280. ^{13}C NMR spectra of compound **96**

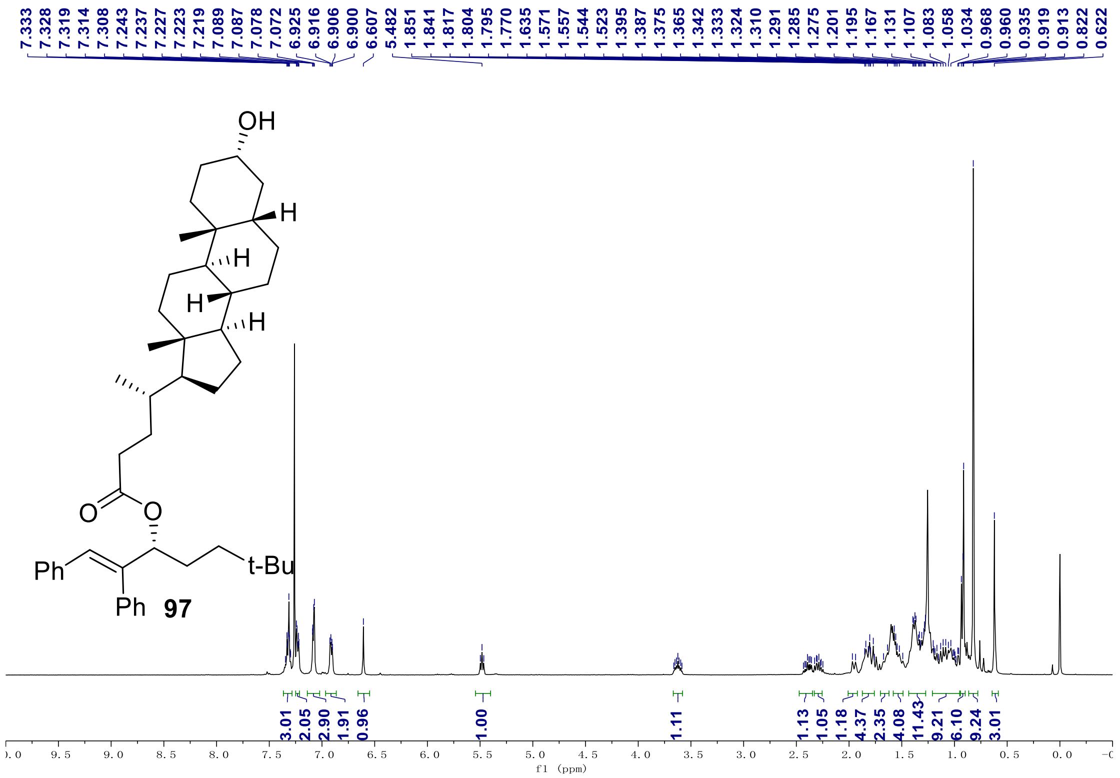

Supplementary Figure 281. ^{1}H NMR spectra of compound **97**

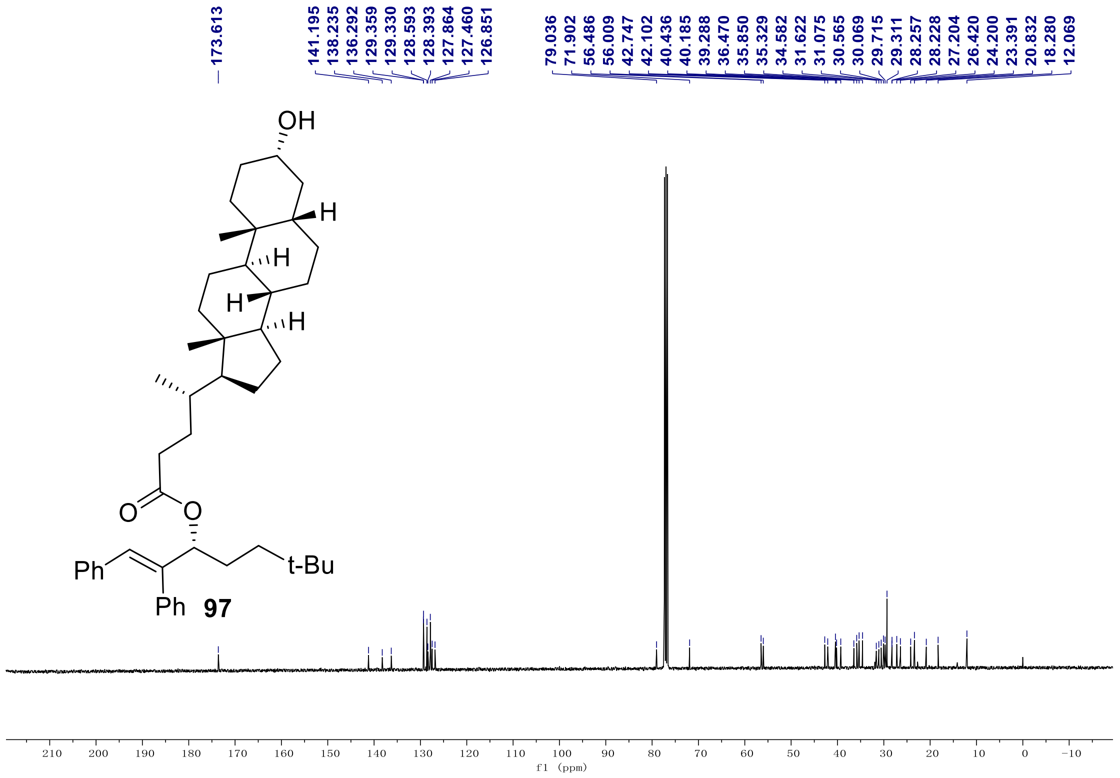

Supplementary Figure 282. ^{13}C NMR spectra of compound **97**

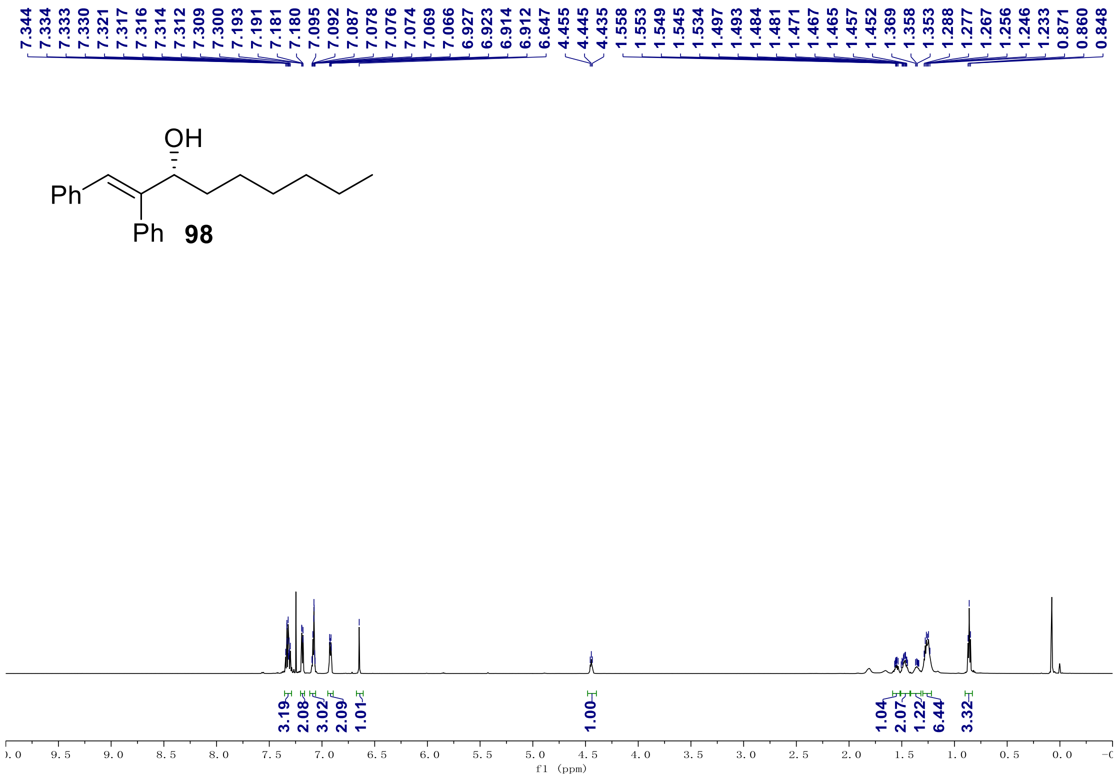

Supplementary Figure 283. ^{1}H NMR spectra of compound **98**

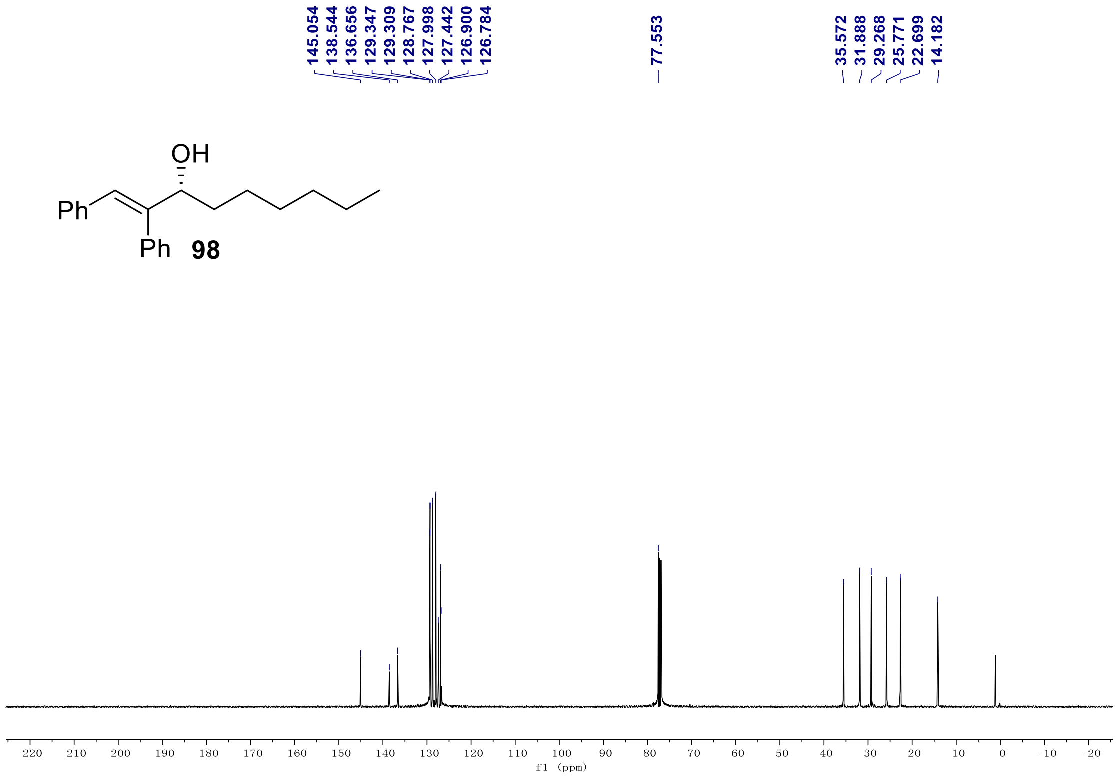

Supplementary Figure 284. ^{13}C NMR spectra of compound **98**

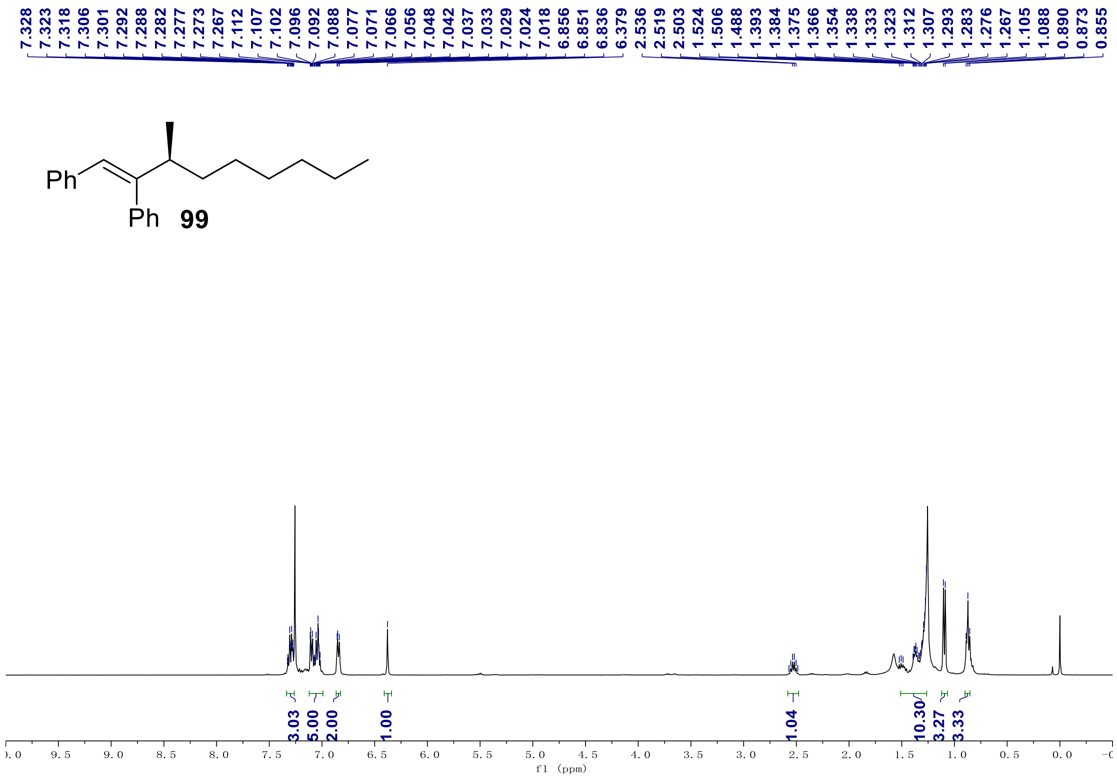

Supplementary Figure 285. ^{1}H NMR spectra of compound **99**

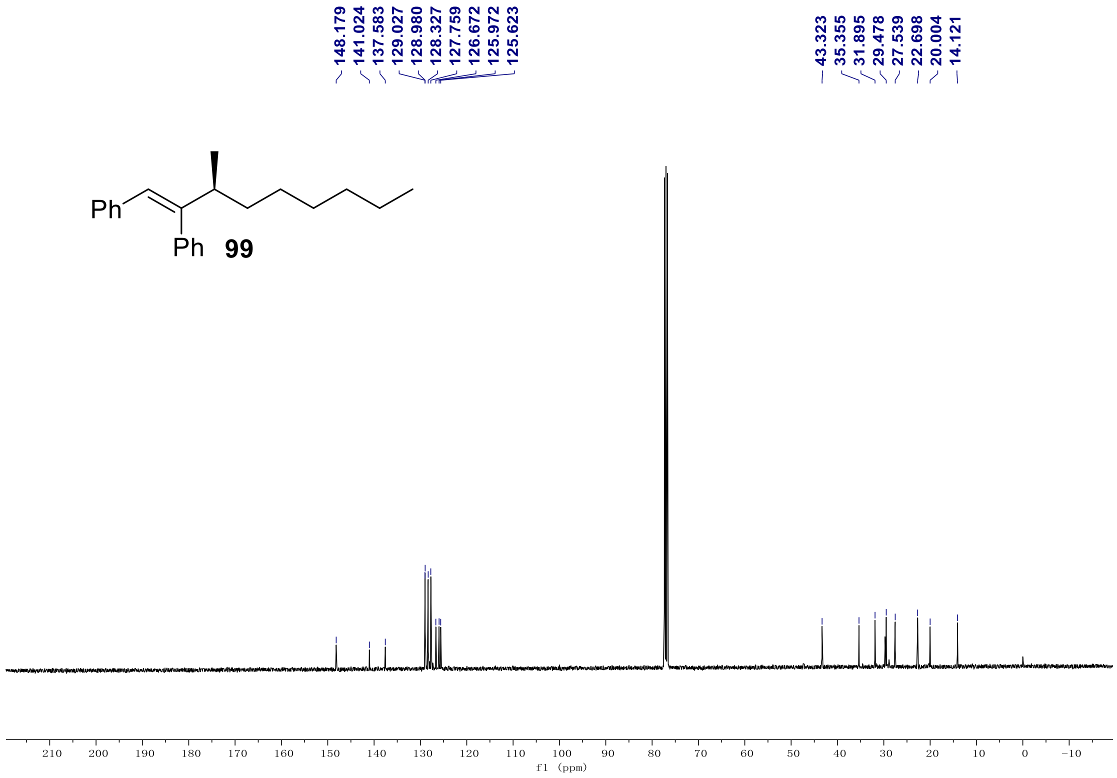

Supplementary Figure 286. ^{13}C NMR spectra of compound **99**

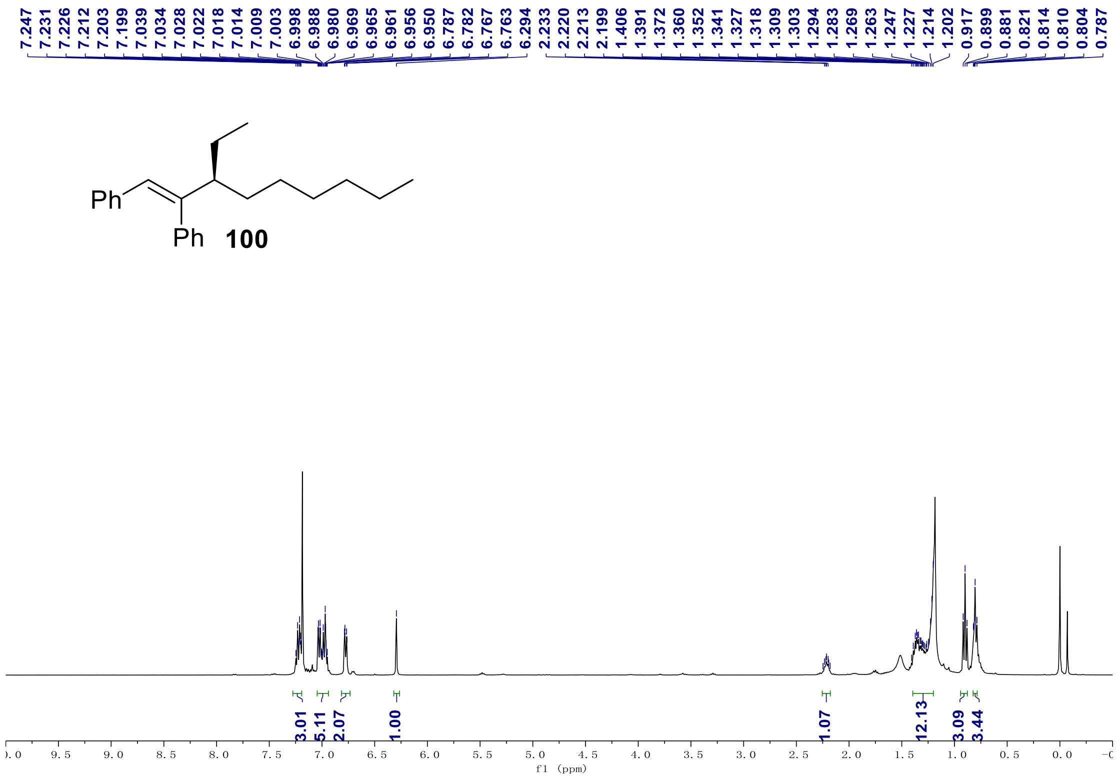

Supplementary Figure 287. ^{1}H NMR spectra of compound **100**

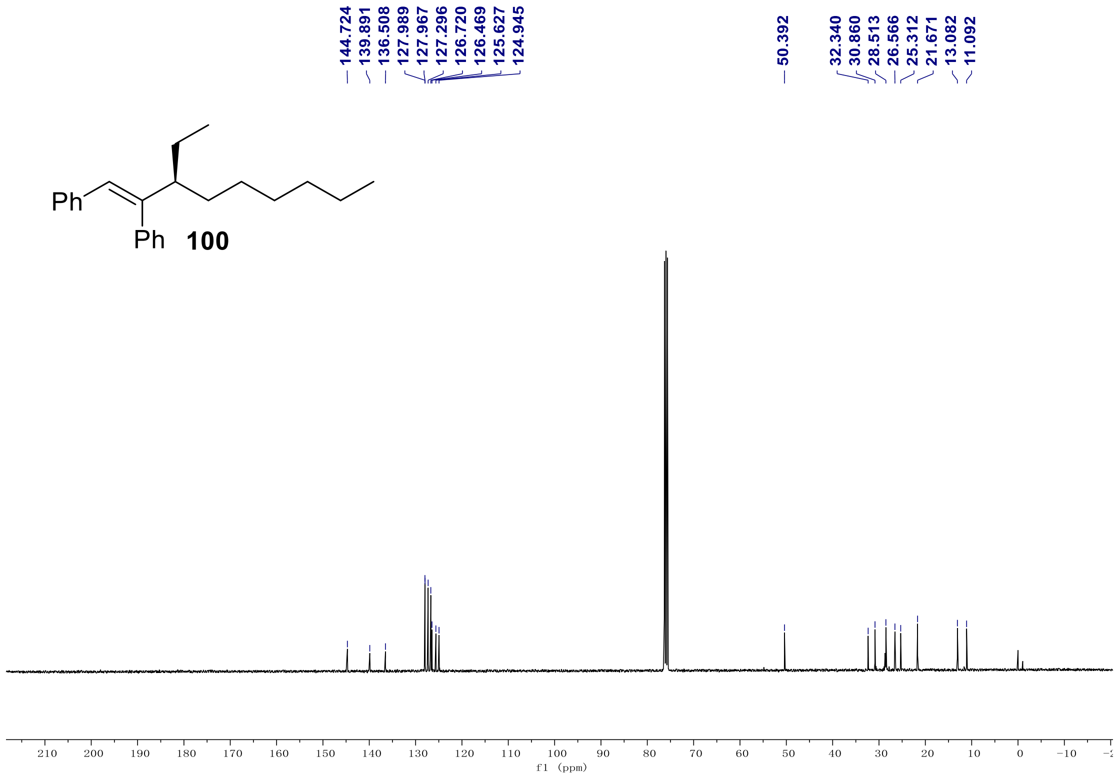

Supplementary Figure 288. ^{13}C NMR spectra of compound **100**

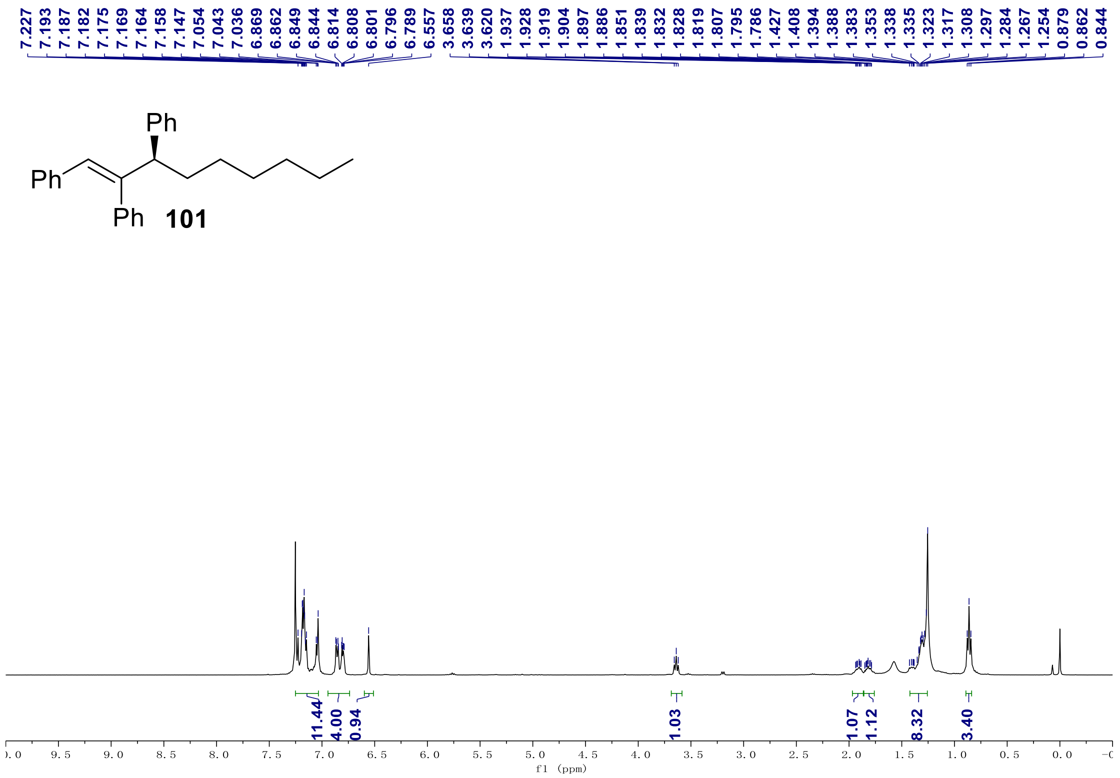

Supplementary Figure 289. ^{1}H NMR spectra of compound **101**

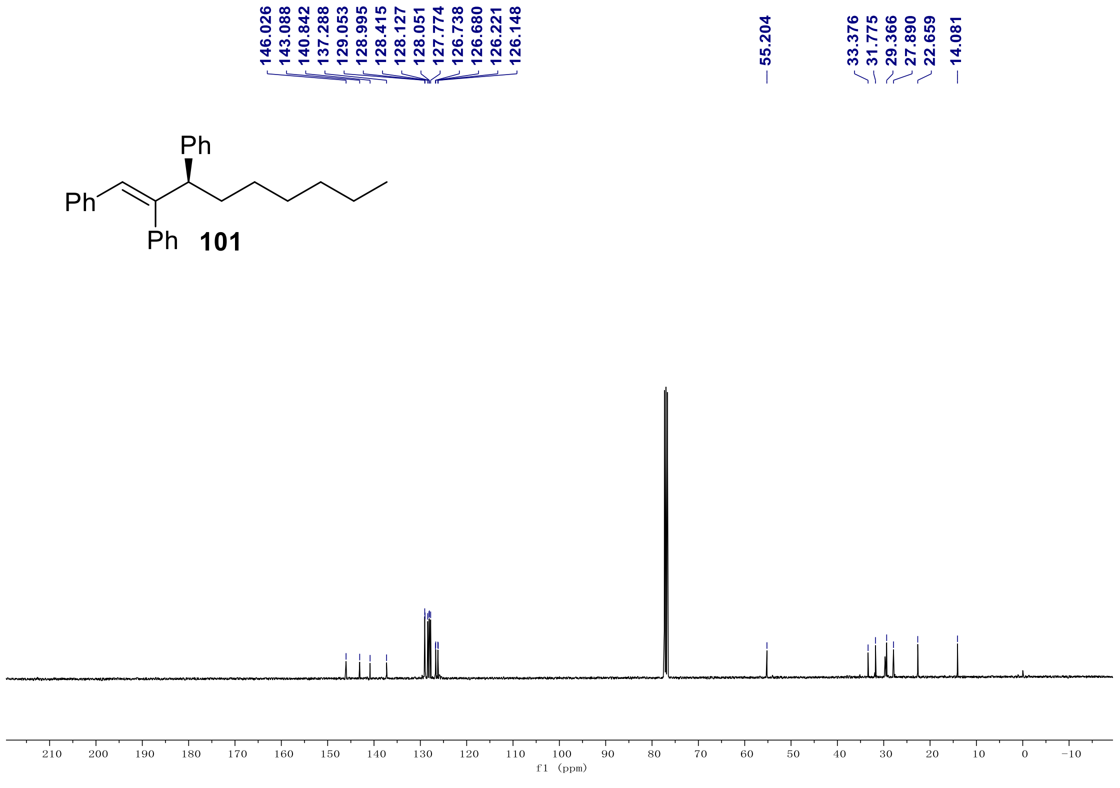

Supplementary Figure 290. ^{13}C NMR spectra of compound **101**

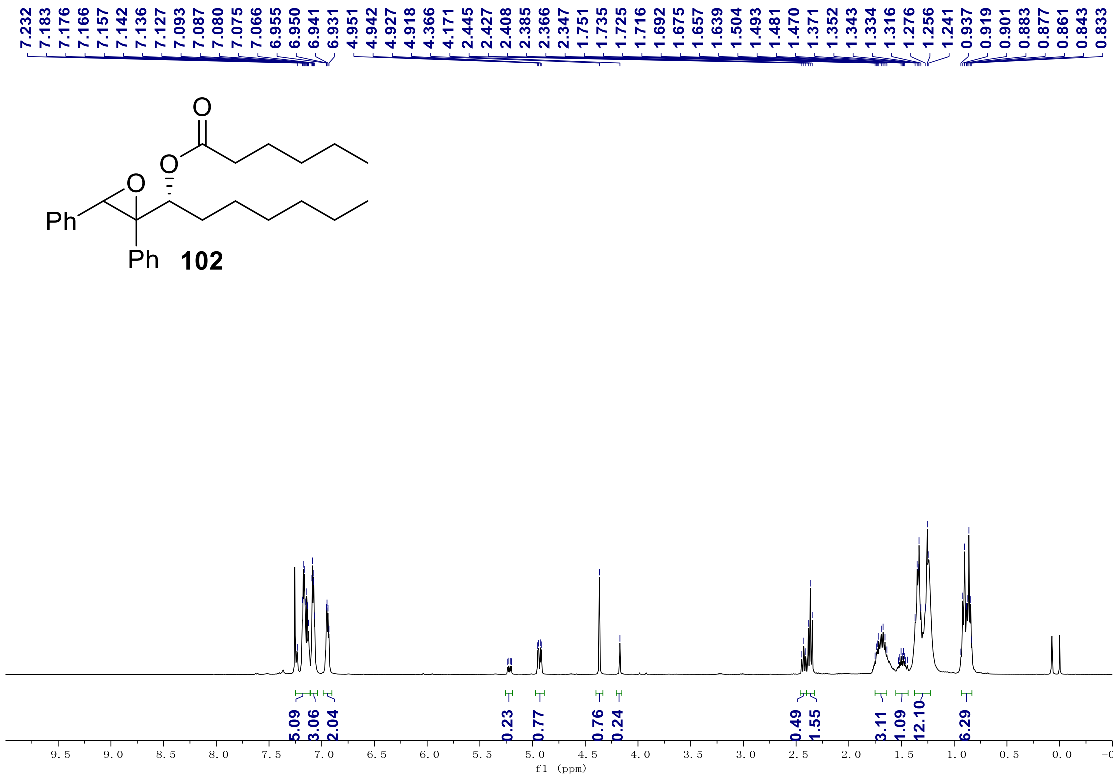

Supplementary Figure 291. ^{1}H NMR spectra of compound **102**

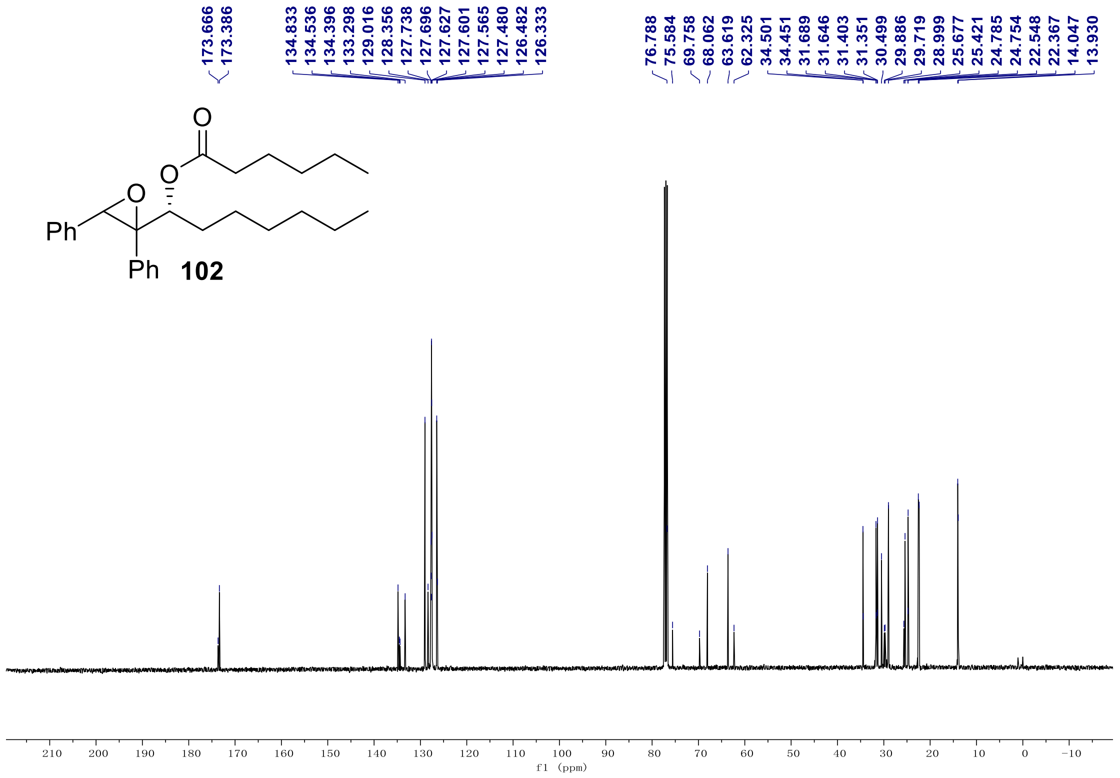

Supplementary Figure 292. ^{13}C NMR spectra of compound **102**

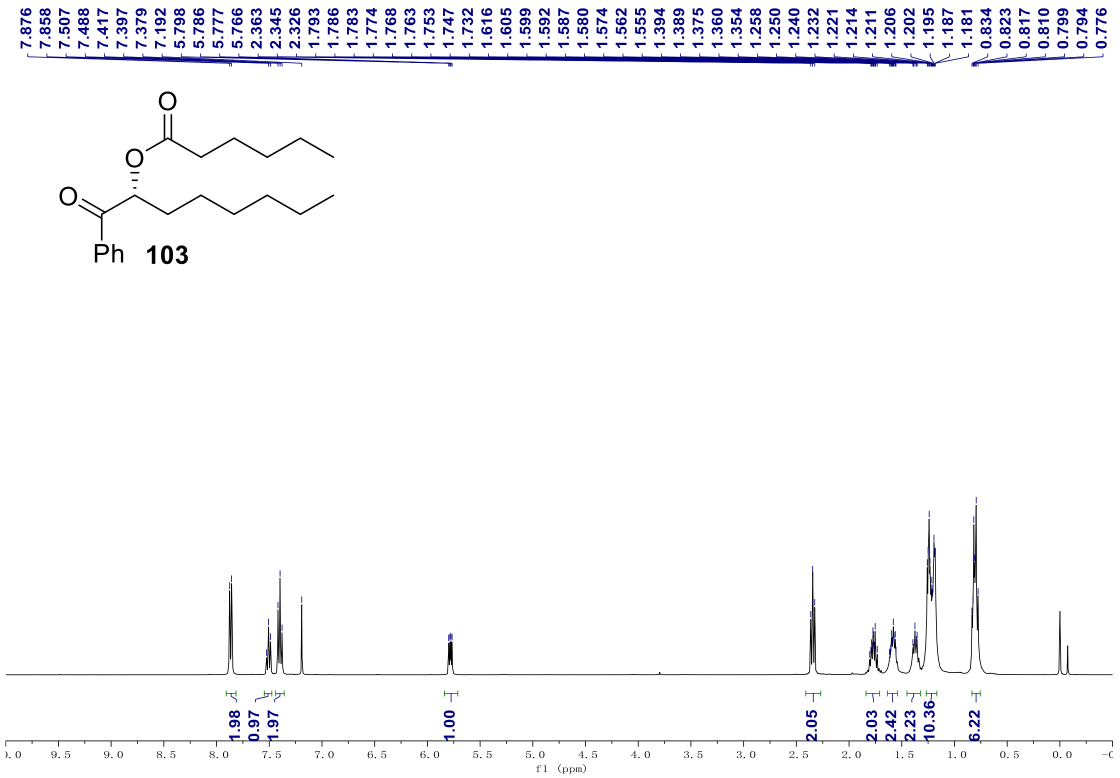

Supplementary Figure 293. ^{1}H NMR spectra of compound **103**

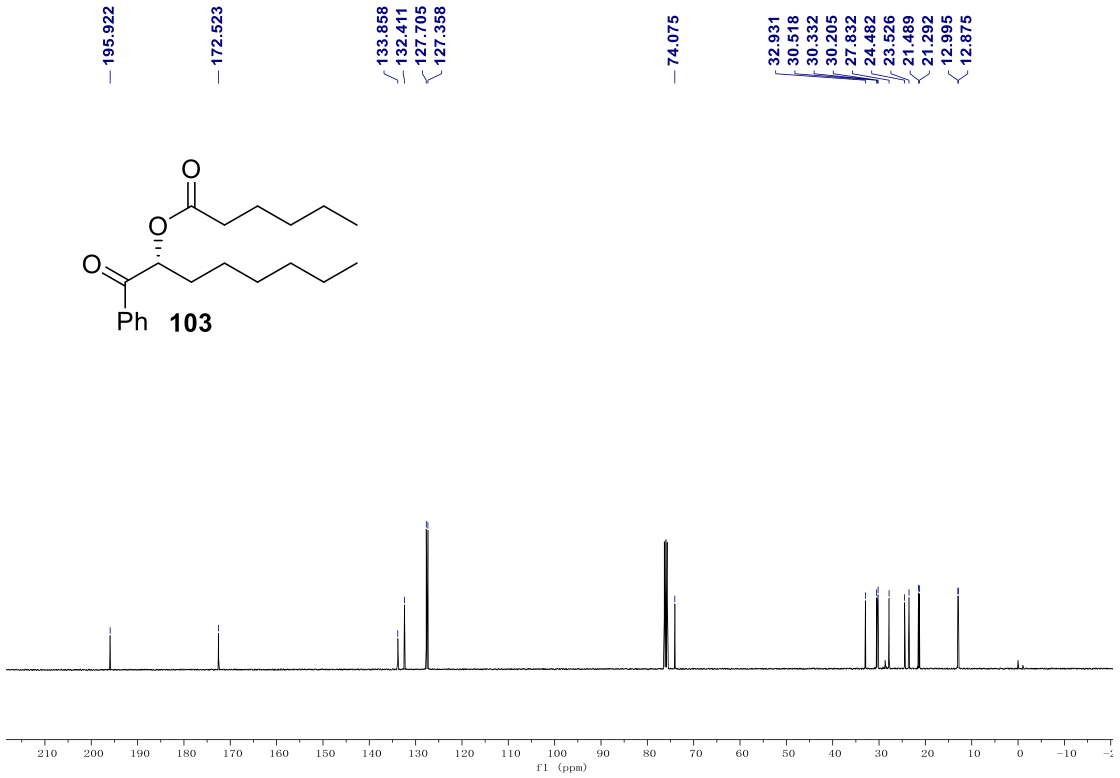

Supplementary Figure 294. ^{13}C NMR spectra of compound **103**

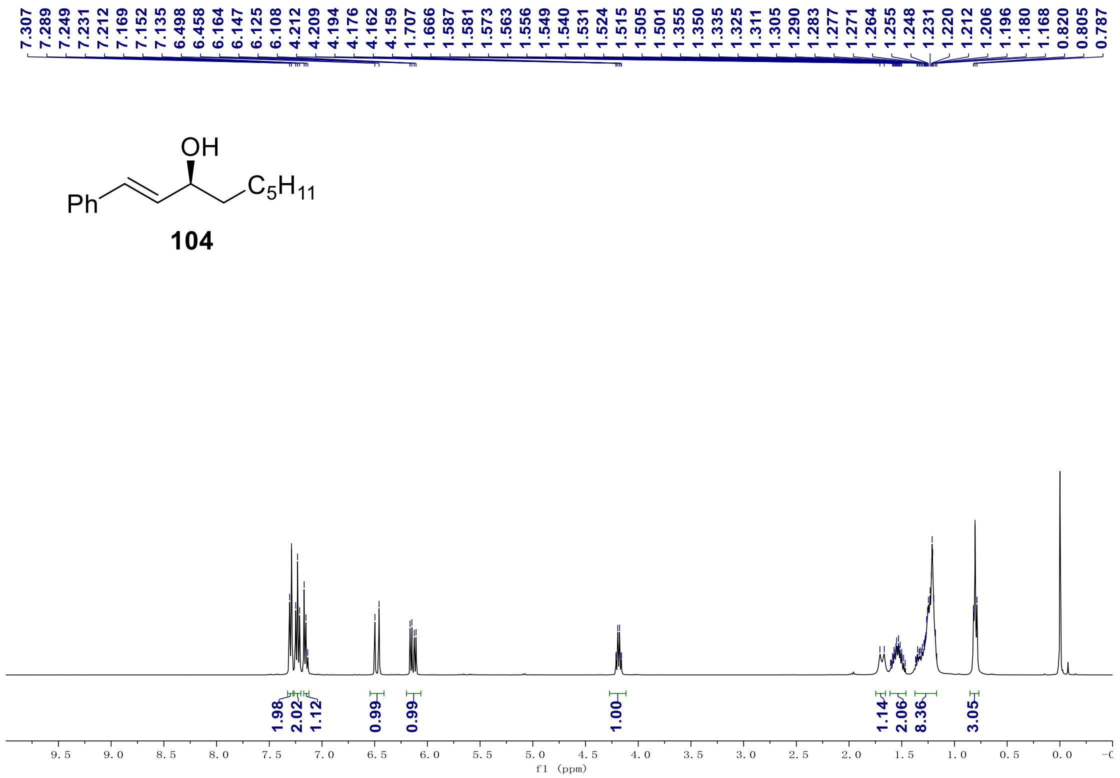

Supplementary Figure 295. ^{1}H NMR spectra of compound **104**

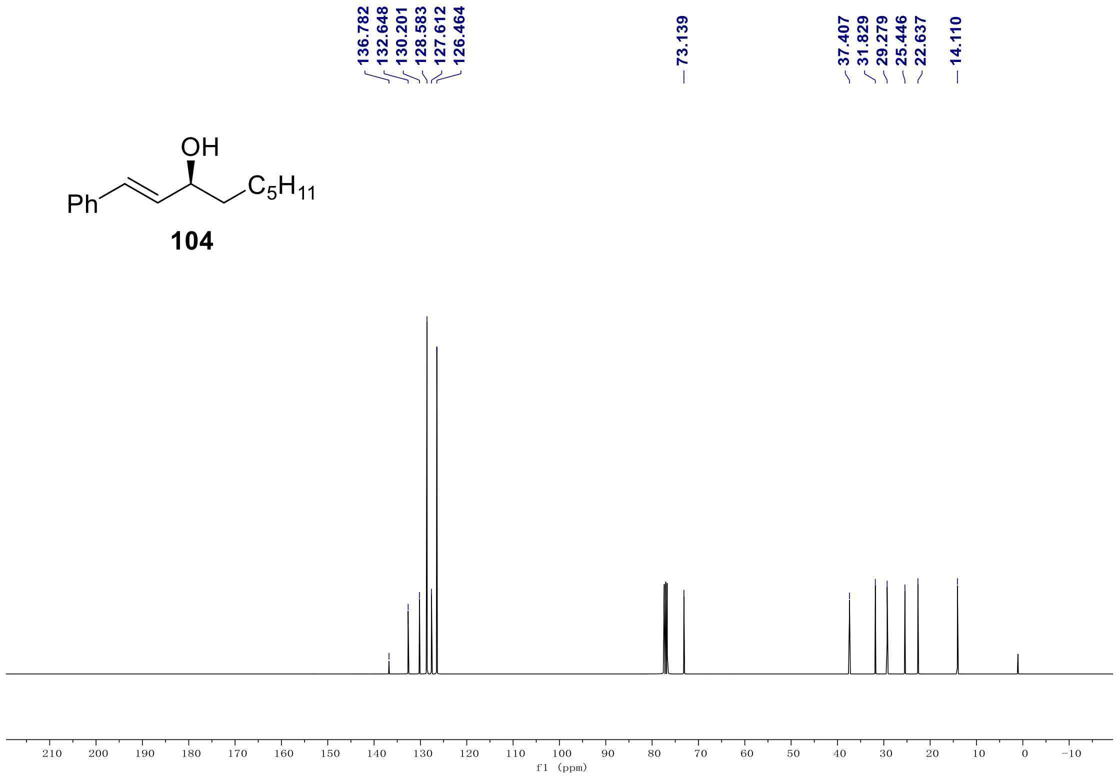

Supplementary Figure 296. ^{13}C NMR spectra of compound **104**

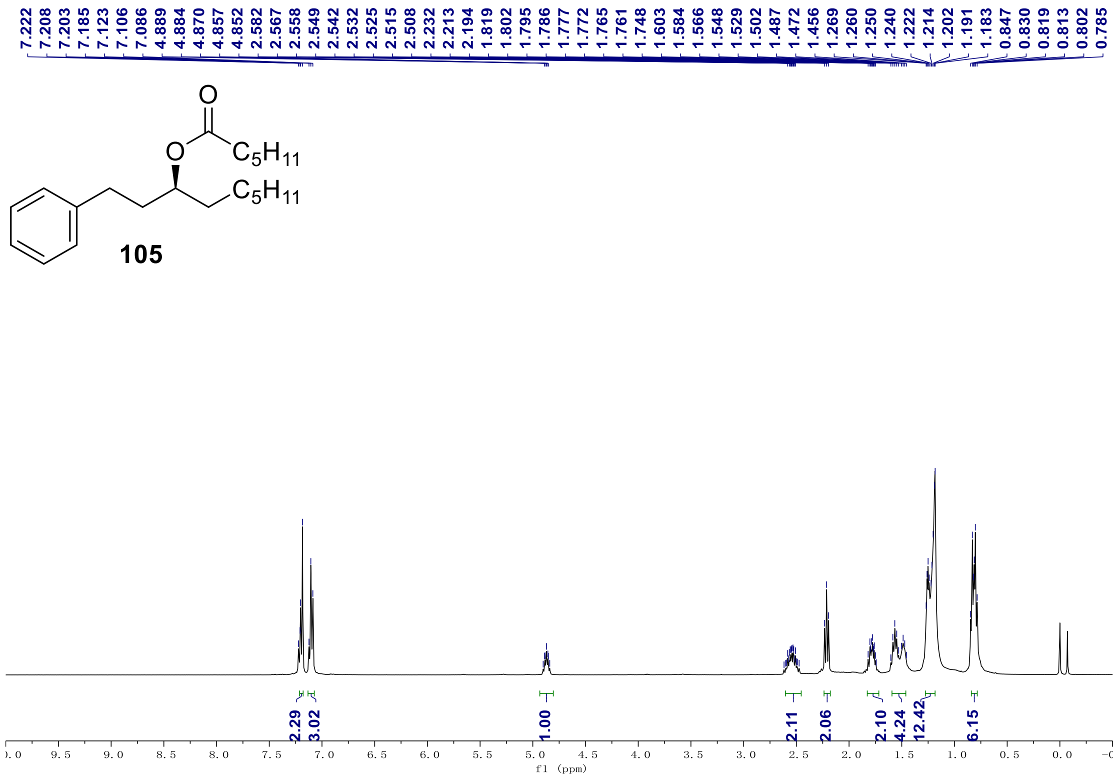

Supplementary Figure 297. ^{1}H NMR spectra of compound **105**

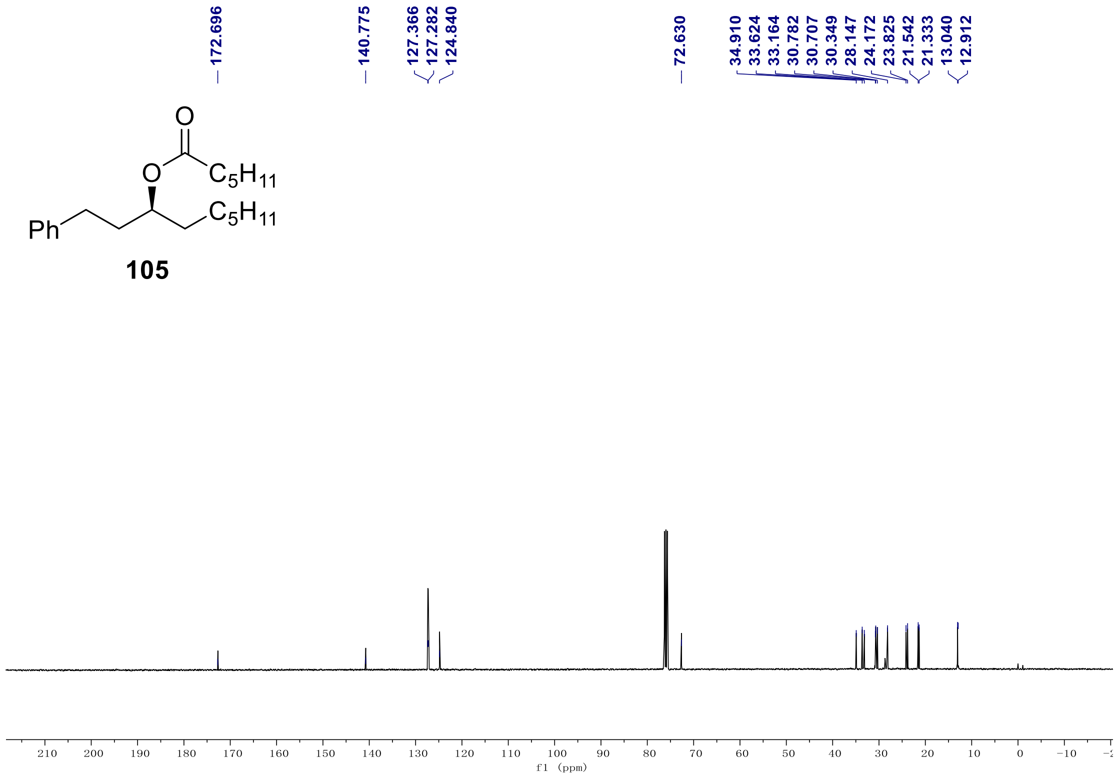

Supplementary Figure 298. ^{13}C NMR spectra of compound **105**

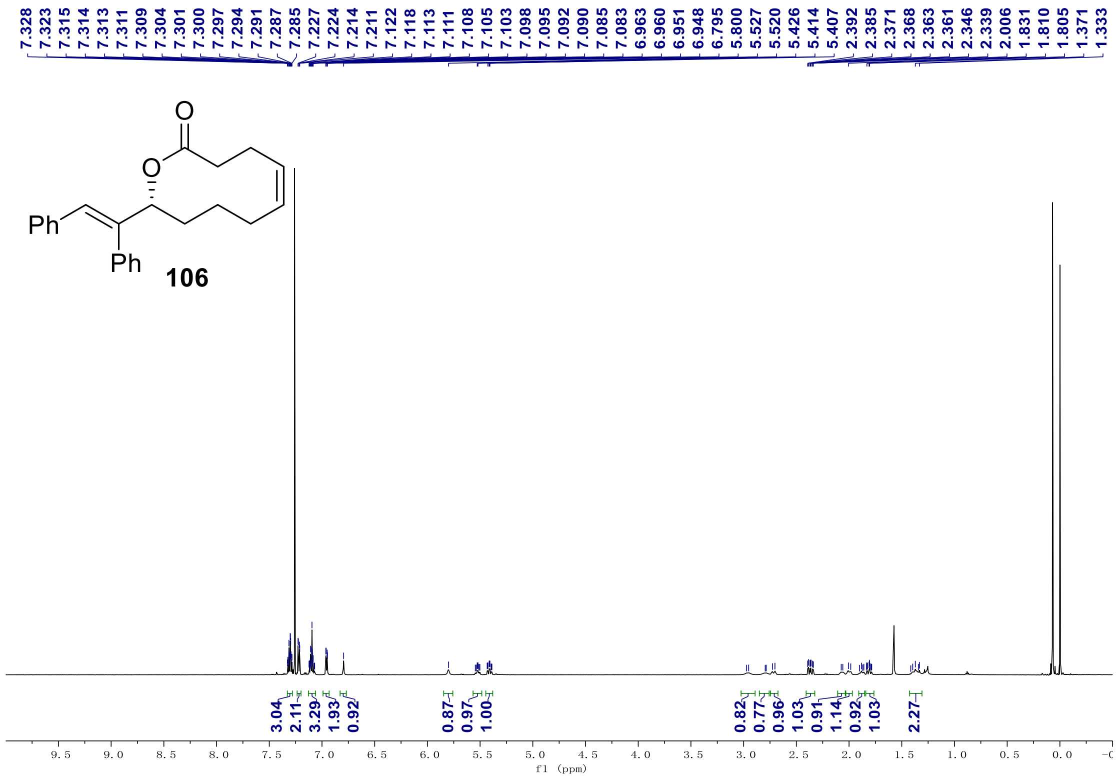

Supplementary Figure 299. ^{1}H NMR spectra of compound **106**

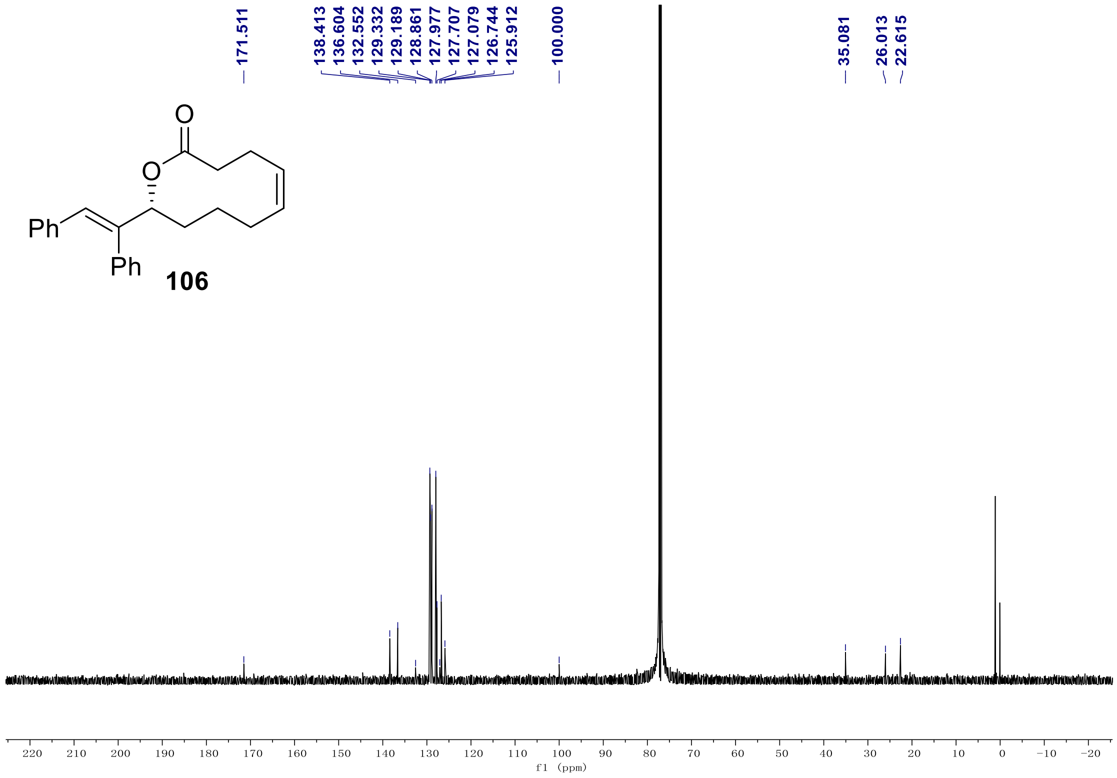

Supplementary Figure 300. ^{13}C NMR spectra of compound **106**

L. HPLC traces

<Chromatogram>

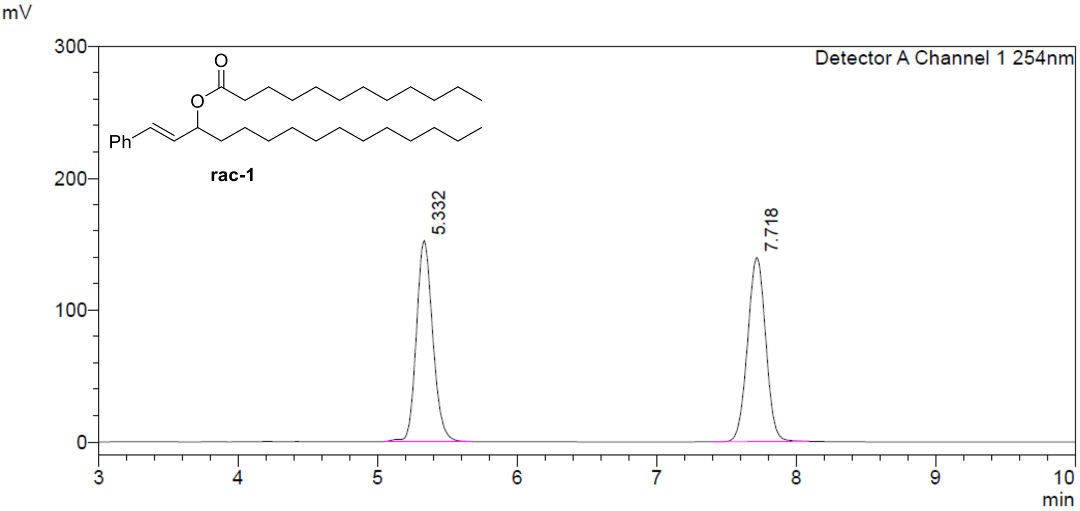

<Peak Table>

Detector A Channel 1 254nm

Peak#	Ret. Time	Height	Height%	Area	Area%
1	5.332	152523	52.197	1250740	49.767
2	7.718	139683	47.803	1262437	50.233
Total		292207	100.000	2513178	100.000

Supplementary Figure 301. Racemic Chromatogram of compound 1

<Chromatogram>

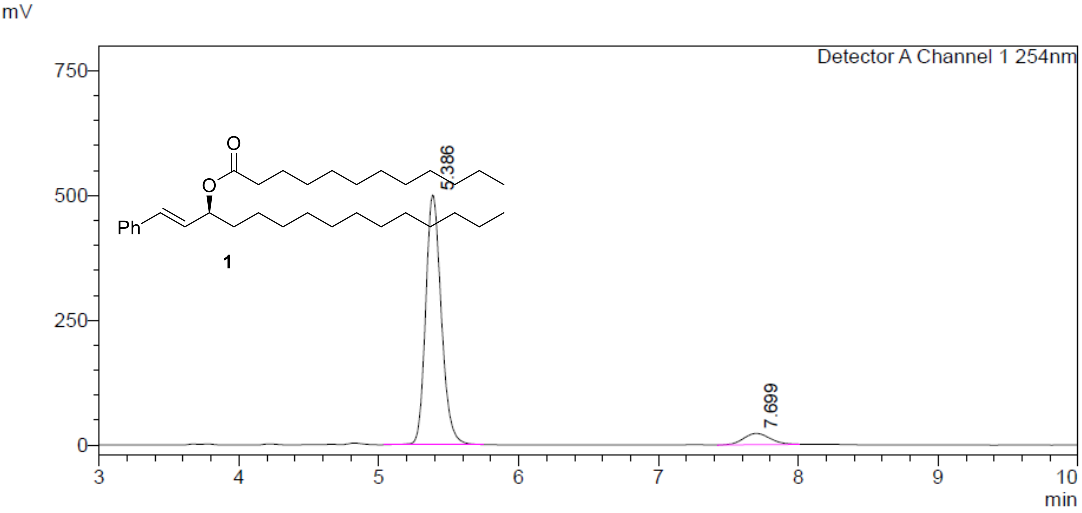

<Peak Table>

Detector A Channel 1 254nm

Peak#	Ret. Time	Height	Height%	Area	Area%
1	5.386	500043	95.698	3889620	92.986
2	7.699	22479	4.302	293406	7.014
Total		522522	100.000	4183026	100.000

Supplementary Figure 302. Scalemic Chromatogram of compound 1

<Chromatogram>

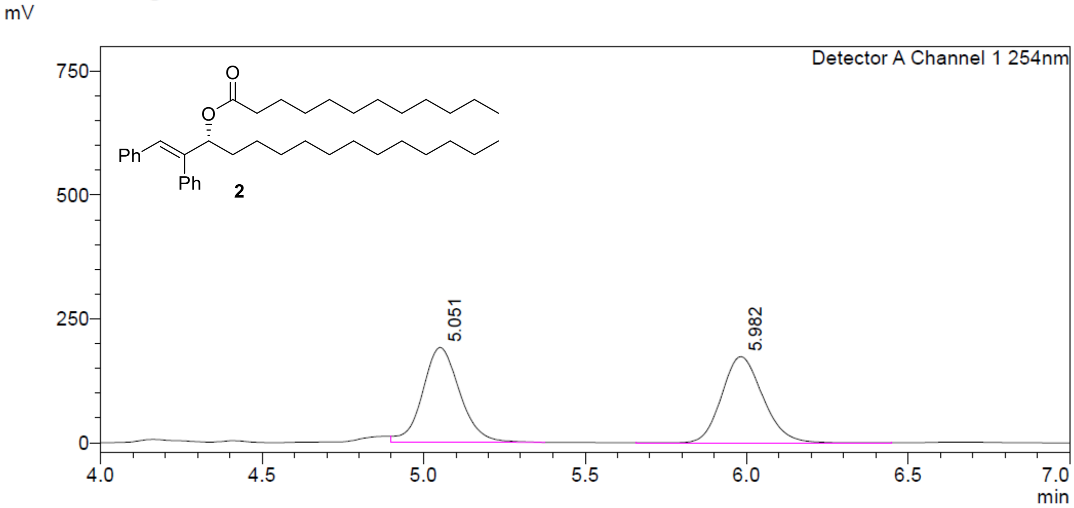

<Peak Table>

Detector A Channel 1 254nm

Peak#	Ret. Time	Height	Height%	Area	Area%
1	5.051	191272	52.333	1527747	49.713
2	5.982	174221	47.667	1545375	50.287
Total		365493	100.000	3073122	100.000

Supplementary Figure 303. Racemic Chromatogram of compound **2**

<Chromatogram>

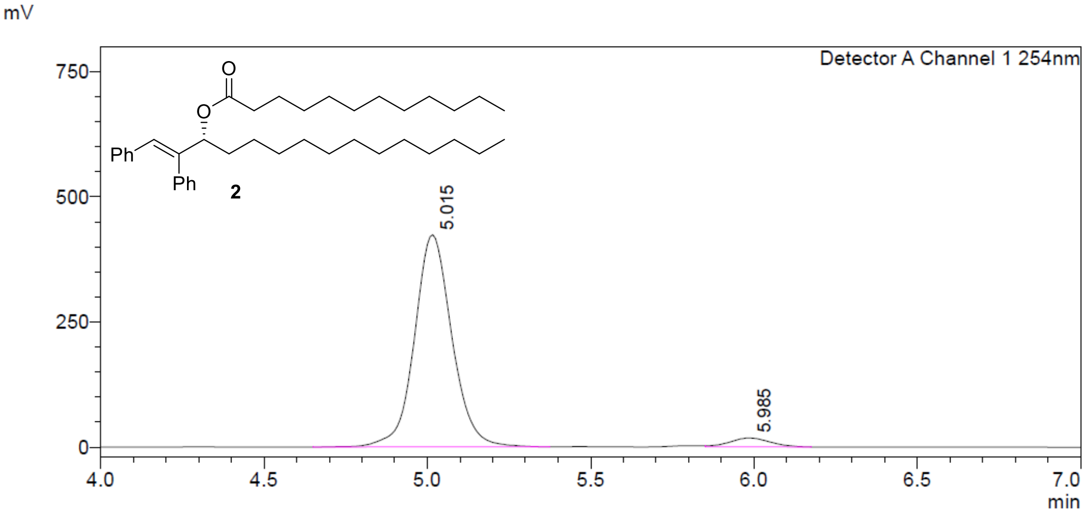

<Peak Table>

Detector A Channel 1 254nm

Peak#	Ret. Time	Height	Height%	Area	Area%
1	5.015	423592	95.973	3377114	95.679
2	5.985	17775	4.027	152499	4.321
Total		441368	100.000	3529612	100.000

Supplementary Figure 304. Scalemic Chromatogram of compound **2**

<Chromatogram>

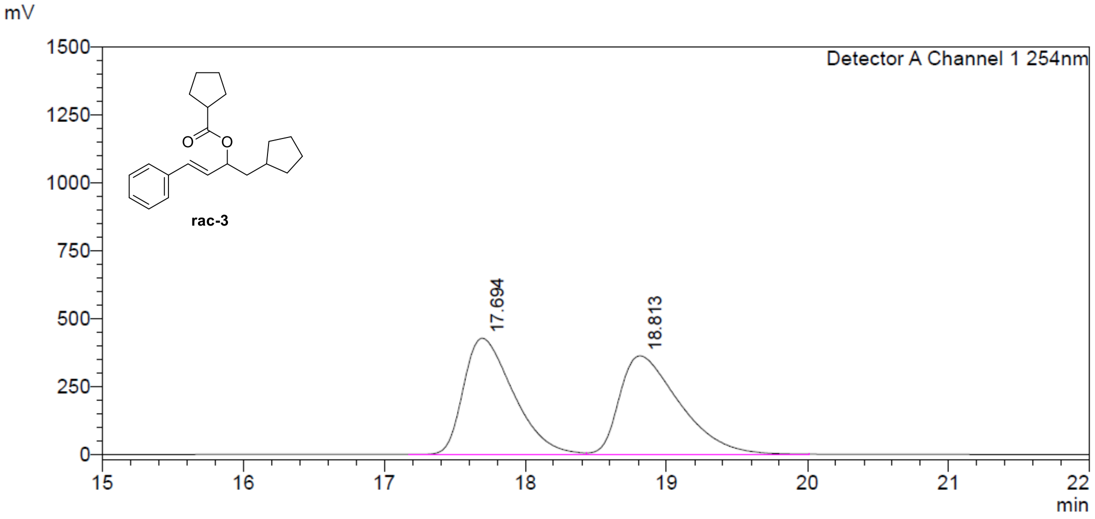

<Peak Table>

Detector A Channel 1 254nm

Peak#	Ret. Time	Height	Height%	Area	Area%
1	17.694	427419	54.118	10505645	49.420
2	18.813	362366	45.882	10752442	50.580
Total		789784	100.000	21258087	100.000

Supplementary Figure 305. Racemic Chromatogram of compound 3

<Chromatogram>

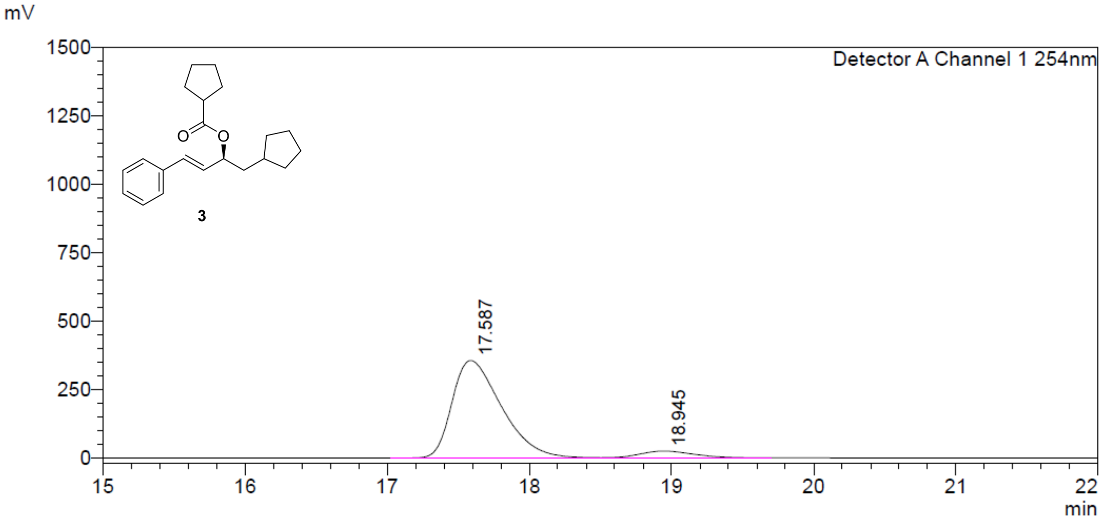

<Peak Table>

Detector A Channel 1 254nm

Peak#	Ret. Time	Height	Height%	Area	Area%
1	17.587	355763	93.443	8585155	92.810
2	18.945	24964	6.557	665122	7.190
Total		380727	100.000	9250277	100.000

Supplementary Figure 306. Scalemic Chromatogram of compound 3

<Chromatogram>

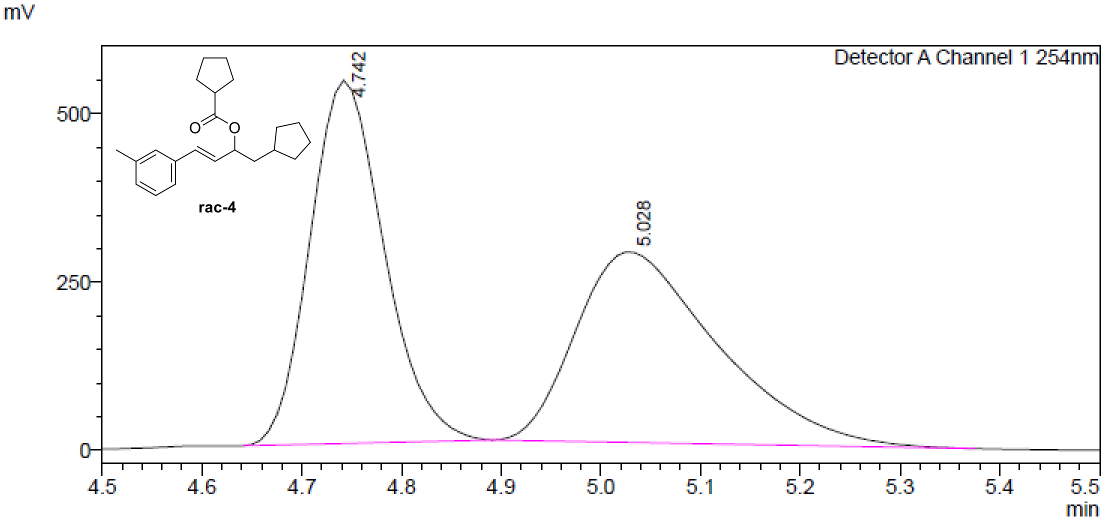

<Peak Table>

Detector A Channel 1 254nm

Peak#	Ret. Time	Height	Height%	Area	Area%
1	4.742	539868	65.587	2794418	49.890
2	5.028	283266	34.413	2806728	50.110
Total		823134	100.000	5601147	100.000

Supplementary Figure 307. Racemic Chromatogram of compound **4**

<Chromatogram>

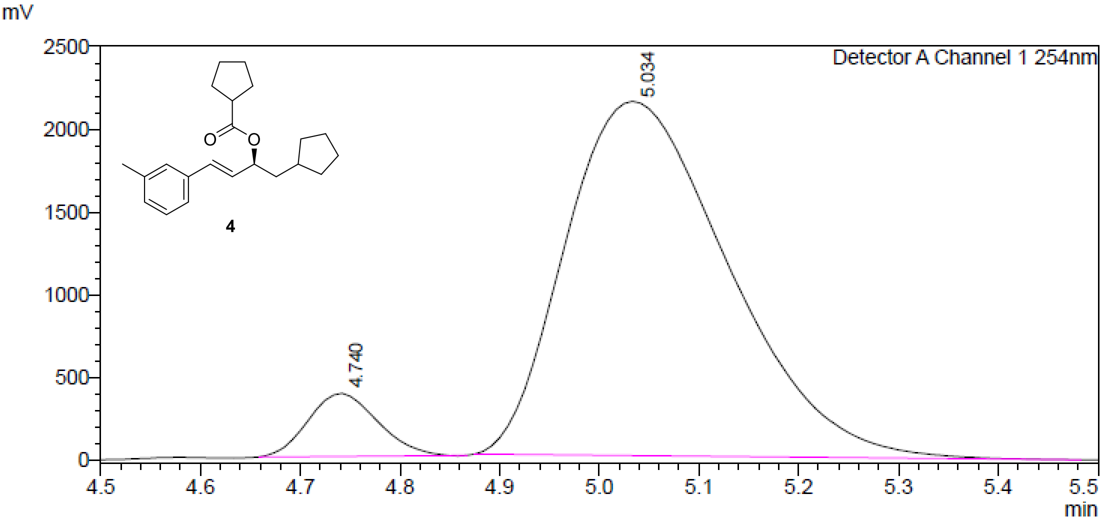

<Peak Table>

Detector A Channel 1 254nm

Peak#	Ret. Time	Height	Height%	Area	Area%
1	4.740	381695	15.148	1852146	7.073
2	5.034	2138134	84.852	24335736	92.927
Total		2519829	100.000	26187882	100.000

Supplementary Figure 308. Scalemic Chromatogram of compound **4**

<Chromatogram>

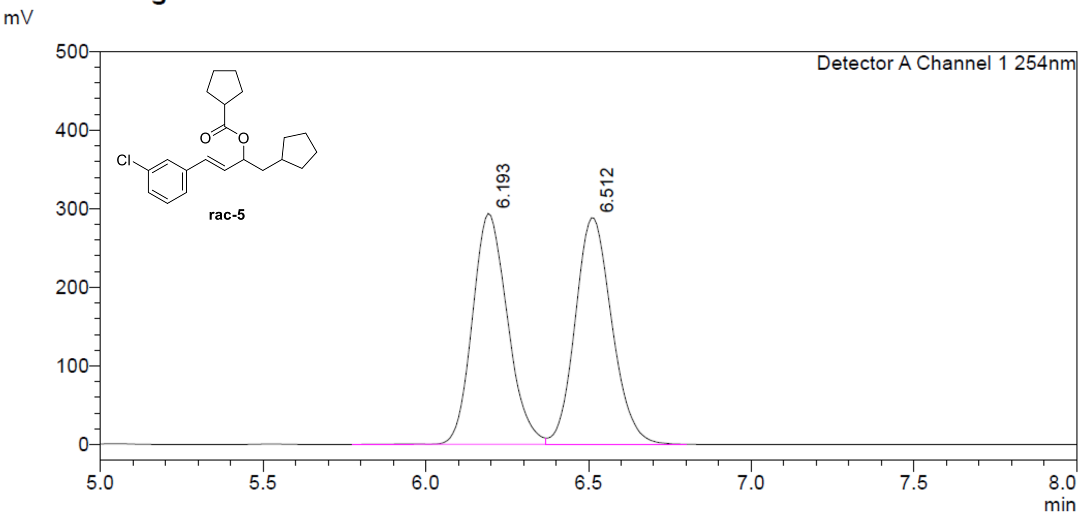

<Peak Table>

Detector A Channel 1 254nm

Peak#	Ret. Time	Height	Height%	Area	Area%
1	6.193	293763	50.466	2203593	49.401
2	6.512	288339	49.534	2257046	50.599
Total		582101	100.000	4460639	100.000

Supplementary Figure 309. Racemic Chromatogram of compound **5**

<Chromatogram>

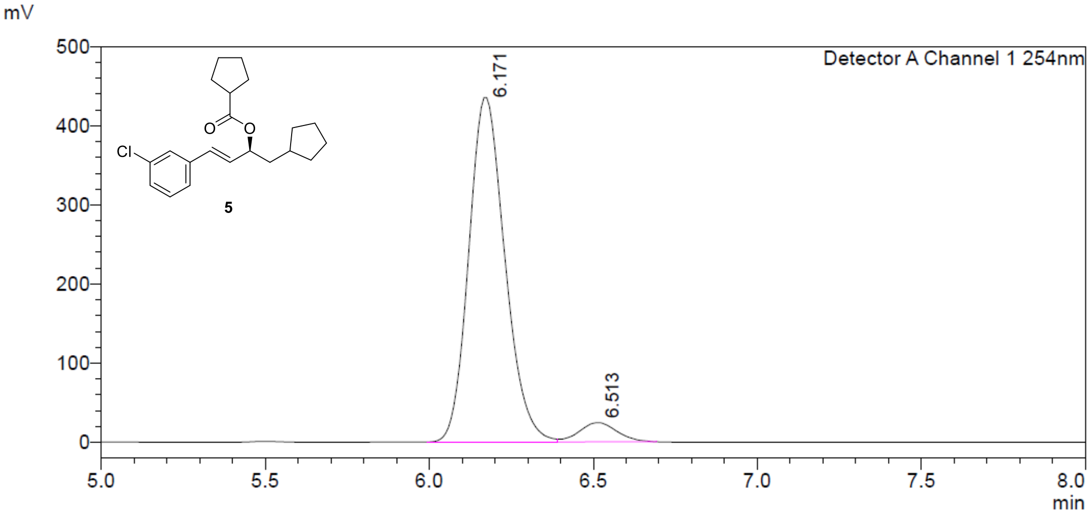

<Peak Table>

Detector A Channel 1 254nm

Peak#	Ret. Time	Height	Height%	Area	Area%
1	6.171	435325	94.712	3340609	94.548
2	6.513	24306	5.288	192645	5.452
Total		459631	100.000	3533253	100.000

Supplementary Figure 310. Scalemic Chromatogram of compound **5**

<Chromatogram>

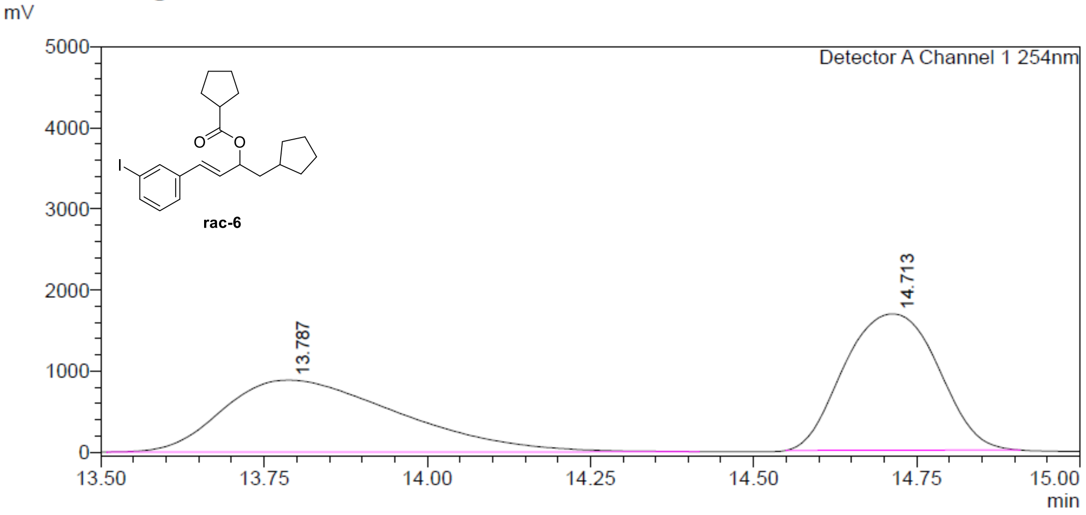

<Peak Table>

Detector A Channel 1 254nm

Peak#	Ret. Time	Height	Height%	Area	Area%
1	13.787	885343	34.523	16610651	49.608
2	14.713	1679175	65.477	16873160	50.392
Total		2564518	100.000	33483811	100.000

Supplementary Figure 311. Racemic Chromatogram of compound **6**

<Chromatogram>

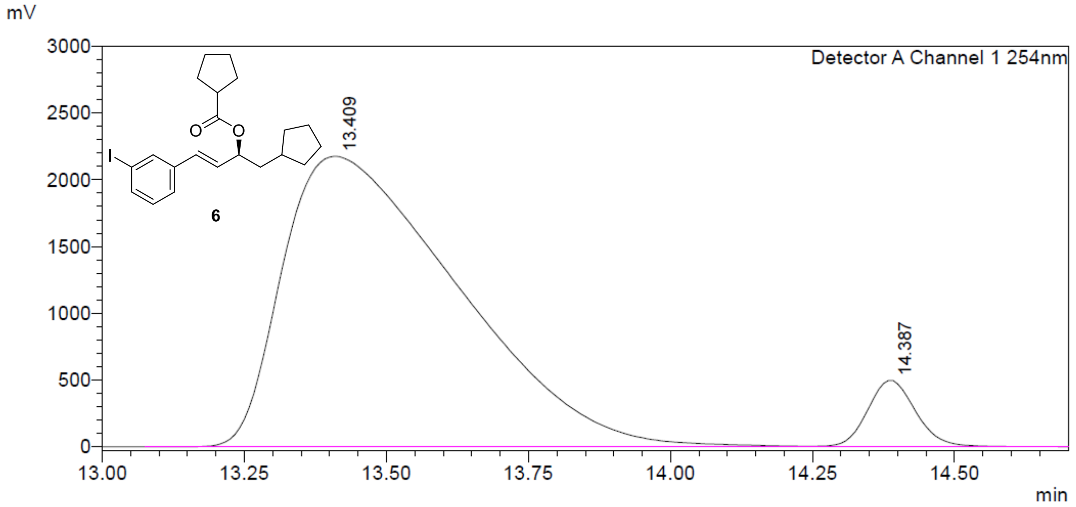

<Peak Table>

Detector A Channel 1 254nm

Peak#	Ret. Time	Height	Height%	Area	Area%
1	13.409	2175680	81.398	46695217	94.041
2	14.387	497221	18.602	2958905	5.959
Total		2672901	100.000	49654122	100.000

Supplementary Figure 312. Scalemic Chromatogram of compound **6**

<Chromatogram>

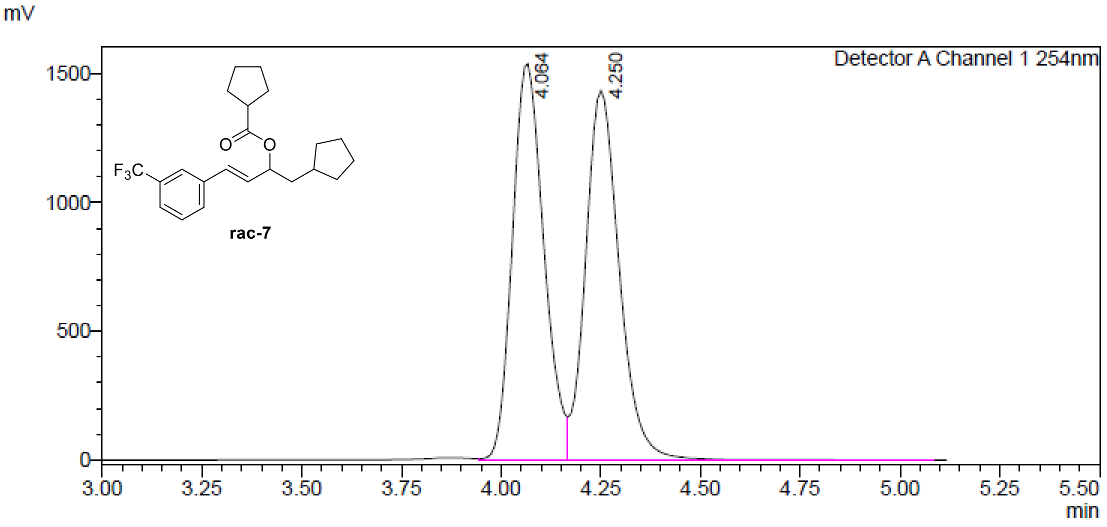

<Peak Table>

Detector A Channel 1 254nm

Peak#	Ret. Time	Height	Height%	Area	Area%
1	4.064	1537725	51.770	8505315	50.292
2	4.250	1432583	48.230	8406487	49.708
Total		2970307	100.000	16911802	100.000

Supplementary Figure 313. Racemic Chromatogram of compound **7**

<Chromatogram>

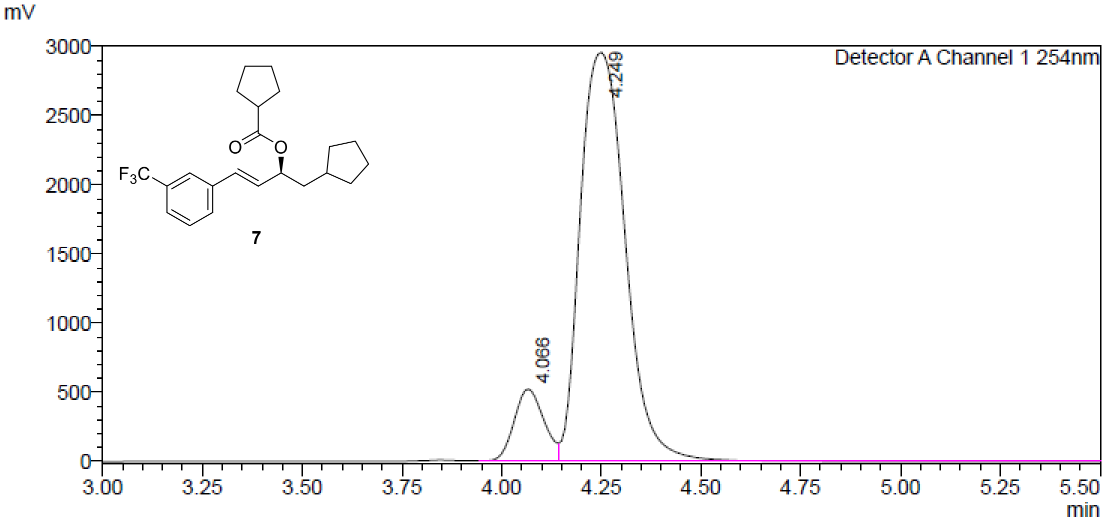

<Peak Table>

Detector A Channel 1 254nm

Peak#	Ret. Time	Height	Height%	Area	Area%
1	4.066	524876	15.071	2770360	10.629
2	4.249	2957736	84.929	23293021	89.371
Total		3482612	100.000	26063381	100.000

Supplementary Figure 314. Scalemic Chromatogram of compound **7**

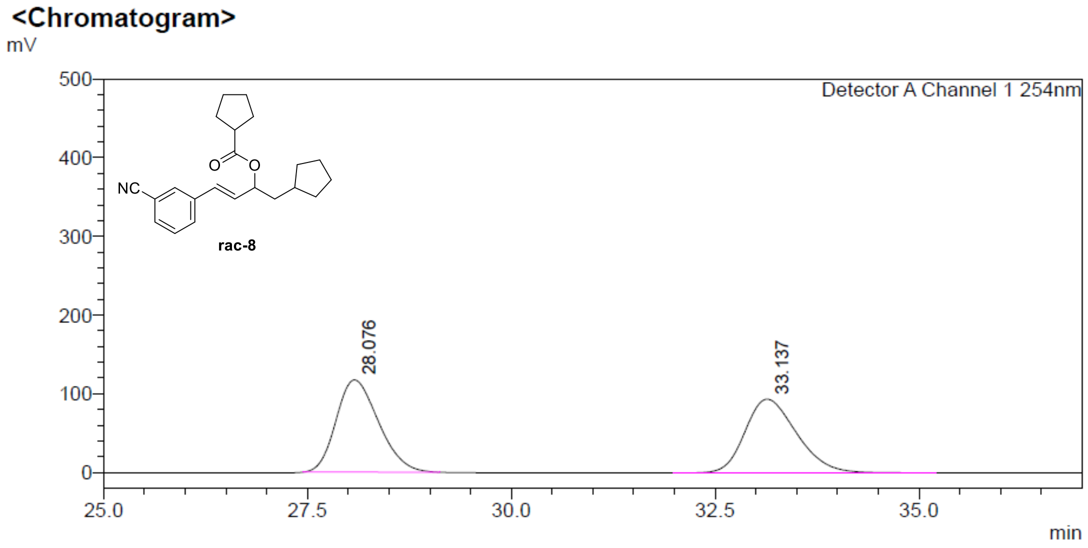

<Peak Table>

Detector A Channel 1 254nm

Peak#	Ret. Time	Height	Height%	Area	Area%
1	28.076	117153	55.636	4240119	50.784
2	33.137	93418	44.364	4109243	49.216
Total		210571	100.000	8349362	100.000

Supplementary Figure 315. Racemic Chromatogram of compound **8**

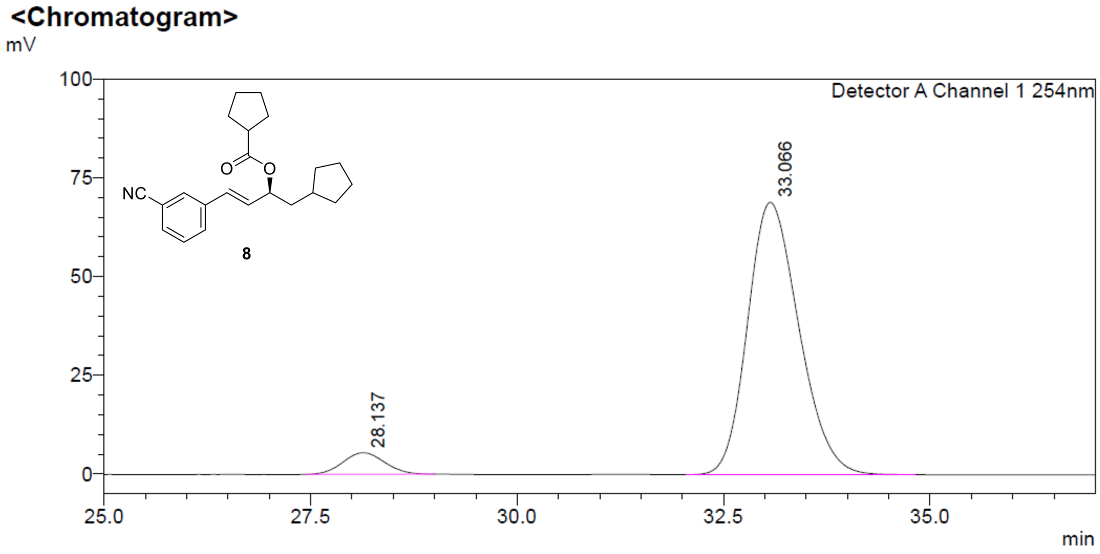

<Peak Table>

Detector A Channel 1 254nm

Peak#	Ret. Time	Height	Height%	Area	Area%
1	28.137	5474	7.351	192977	6.066
2	33.066	68994	92.649	2988377	93.934
Total		74468	100.000	3181354	100.000

Supplementary Figure 316. Scalemic Chromatogram of compound **8**

<Chromatogram>

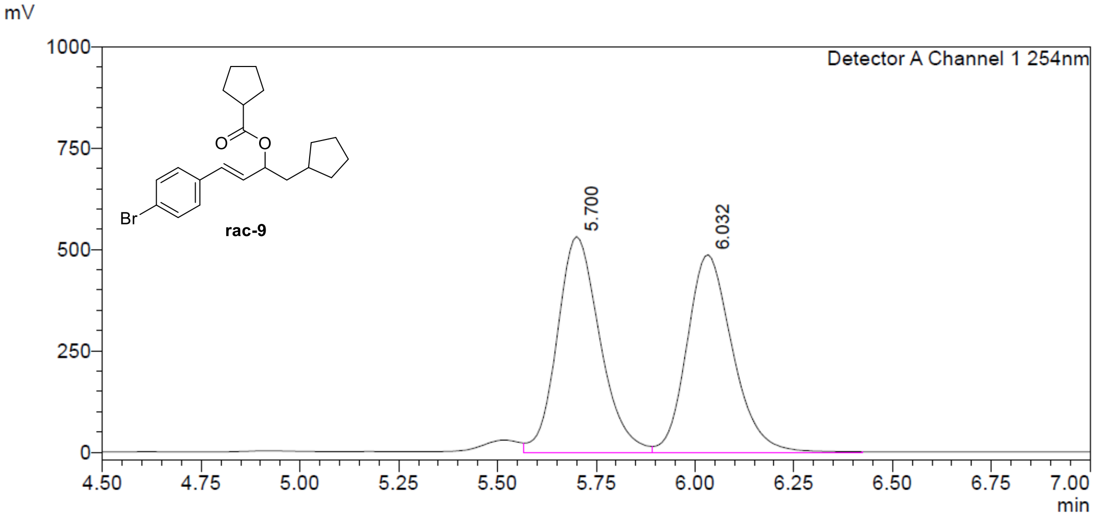

<Peak Table>

Detector A Channel 1 254nm

Peak#	Ret. Time	Height	Height%	Area	Area%
1	5.700	532074	52.214	3986365	50.573
2	6.032	486953	47.786	3896053	49.427
Total		1019028	100.000	7882418	100.000

Supplementary Figure 317. Racemic Chromatogram of compound **9**

<Chromatogram>

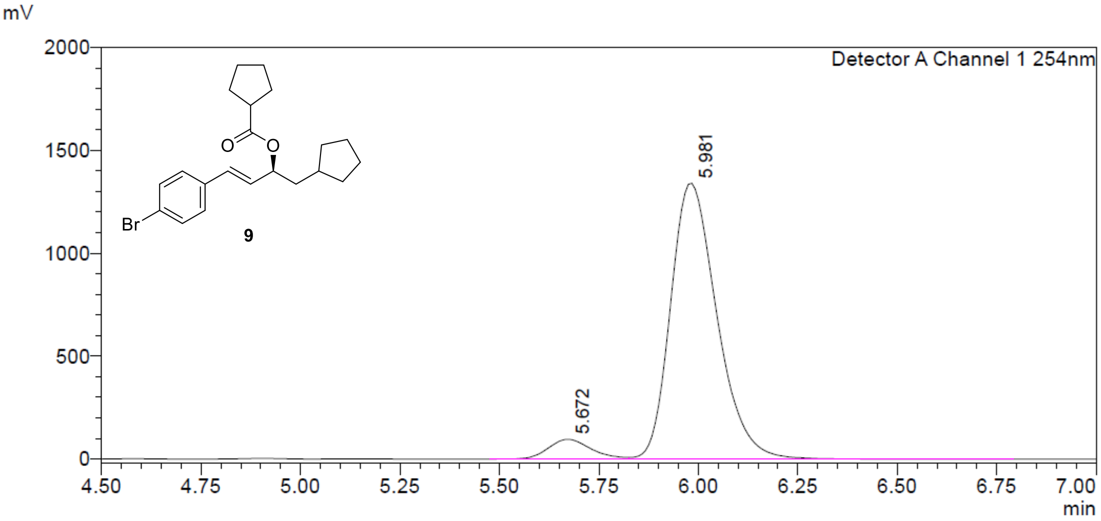

<Peak Table>

Detector A Channel 1 254nm

Peak#	Ret. Time	Height	Height%	Area	Area%
1	5.672	95940	6.683	713220	6.184
2	5.981	1339594	93.317	10820259	93.816
Total		1435534	100.000	11533479	100.000

Supplementary Figure 318. Scalemic Chromatogram of compound **9**

<Chromatogram>

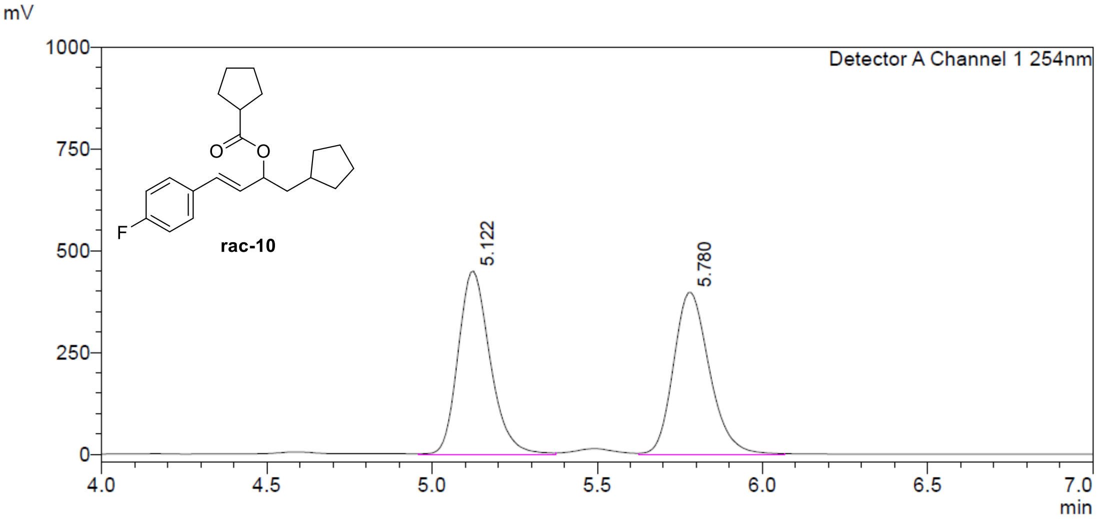

<Peak Table>

Detector A Channel 1 254nm

Peak#	Ret. Time	Height	Height%	Area	Area%
1	5.122	449902	53.075	3030653	50.214
2	5.780	397767	46.925	3004869	49.786
Total		847669	100.000	6035522	100.000

Supplementary Figure 319. Racemic Chromatogram of compound **10**

<Chromatogram>

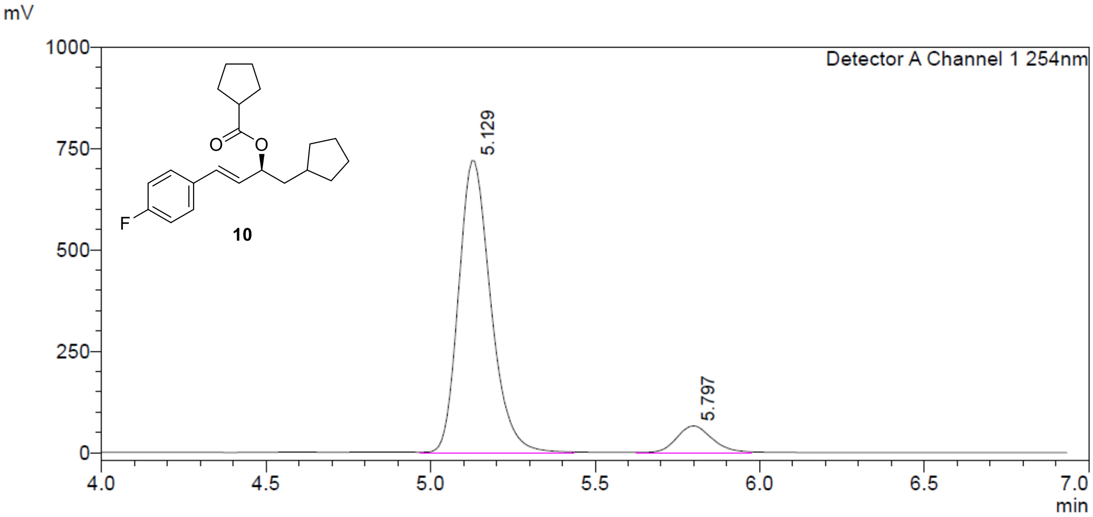

<Peak Table>

Detector A Channel 1 254nm

Peak#	Ret. Time	Height	Height%	Area	Area%
1	5.129	720793	91.638	4891581	90.847
2	5.797	65770	8.362	492849	9.153
Total		786563	100.000	5384430	100.000

Supplementary Figure 320. Scalemic Chromatogram of compound **10**

<Chromatogram>

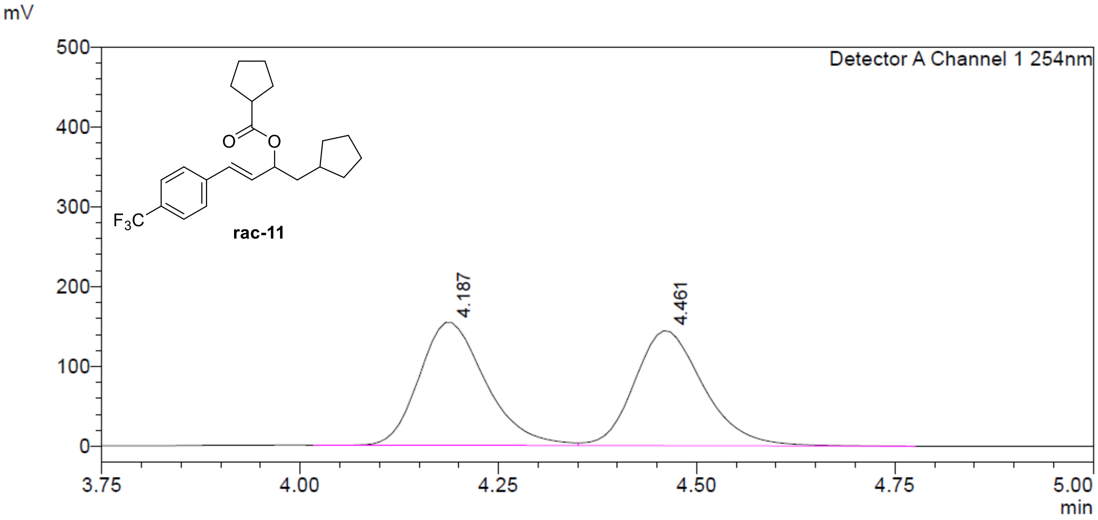

<Peak Table>

Detector A Channel 1 254nm

Peak#	Ret. Time	Height	Height%	Area	Area%
1	4.187	153785	51.657	899832	50.789
2	4.461	143919	48.343	871874	49.211
Total		297704	100.000	1771707	100.000

Supplementary Figure 321. Racemic Chromatogram of compound **11**

<Chromatogram>

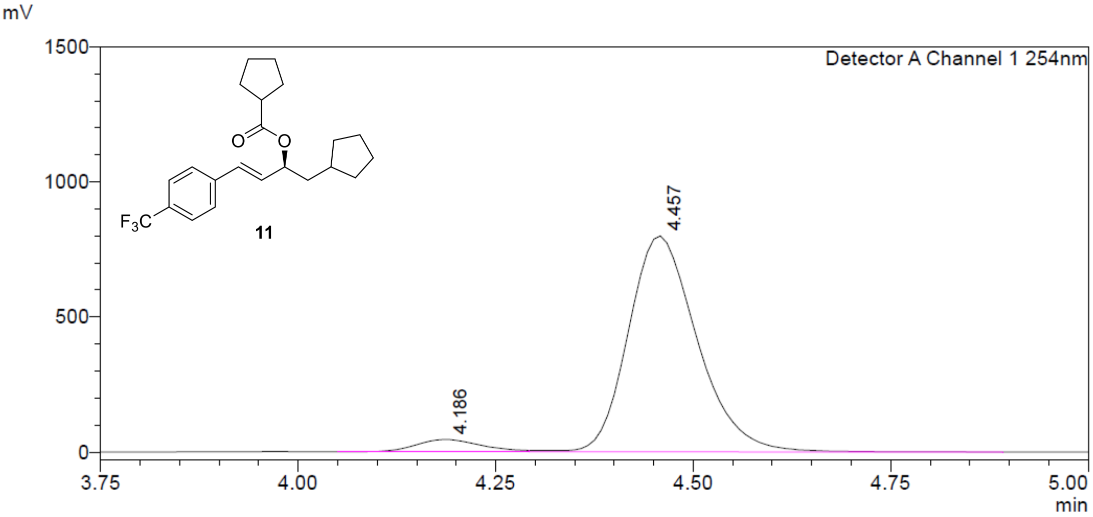

<Peak Table>

Detector A Channel 1 254nm

Peak#	Ret. Time	Height	Height%	Area	Area%
1	4.186	45402	5.372	259553	5.080
2	4.457	799765	94.628	4850040	94.920
Total		845167	100.000	5109593	100.000

Supplementary Figure 322. Scalemic Chromatogram of compound **11**

<Chromatogram>

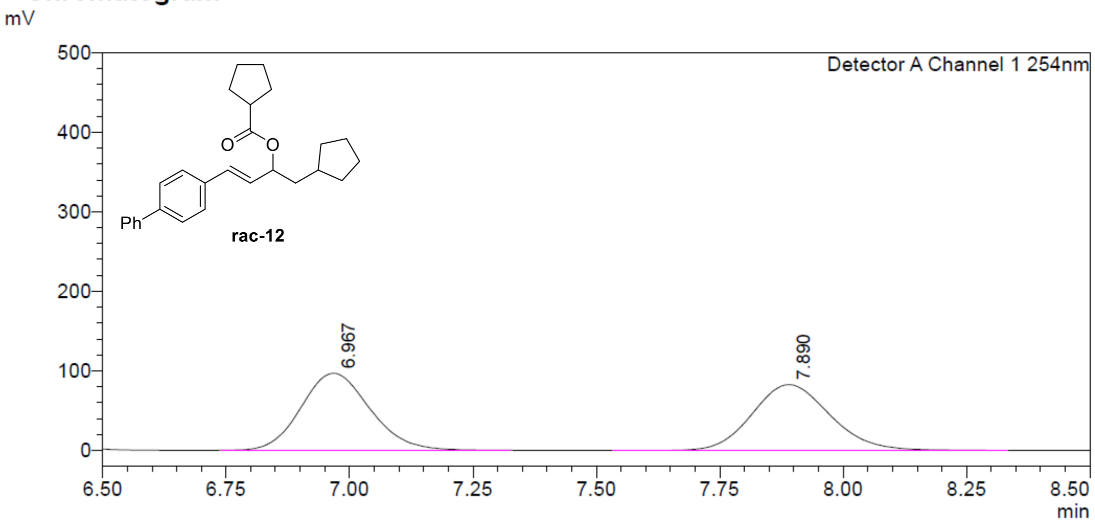

<Peak Table>

Detector A Channel 1 254nm

Peak#	Ret. Time	Height	Height%	Area	Area%
1	6.967	96961	53.997	941112	50.977
2	7.890	82608	46.003	905040	49.023
Total		179569	100.000	1846152	100.000

Supplementary Figure 323. Racemic Chromatogram of compound **12**

<Chromatogram>

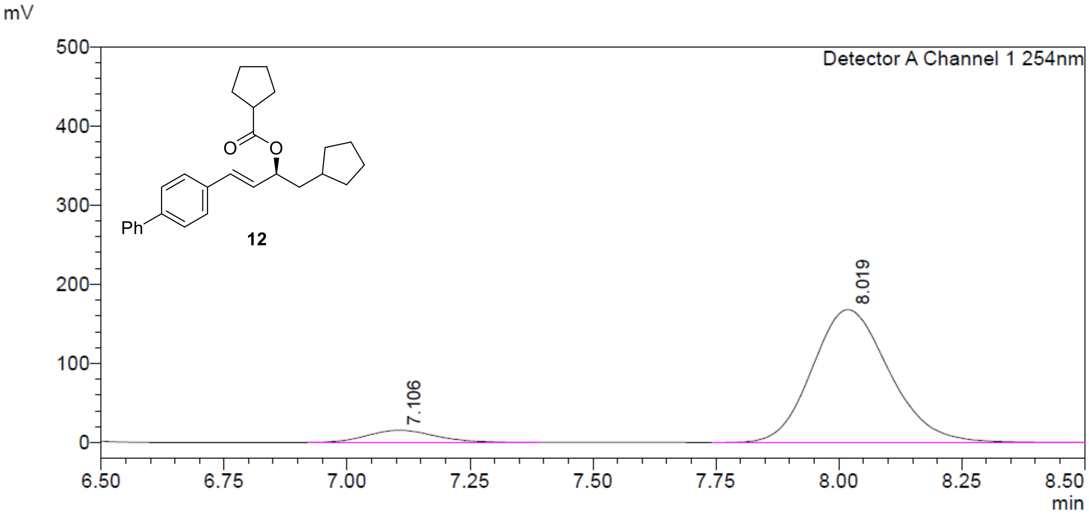

<Peak Table>

Detector A Channel 1 254nm

Peak#	Ret. Time	Height	Height%	Area	Area%
1	7.106	15287	8.333	145879	7.342
2	8.019	168156	91.667	1840944	92.658
Total		183443	100.000	1986823	100.000

Supplementary Figure 324. Scalemic Chromatogram of compound **12**

<Chromatogram>

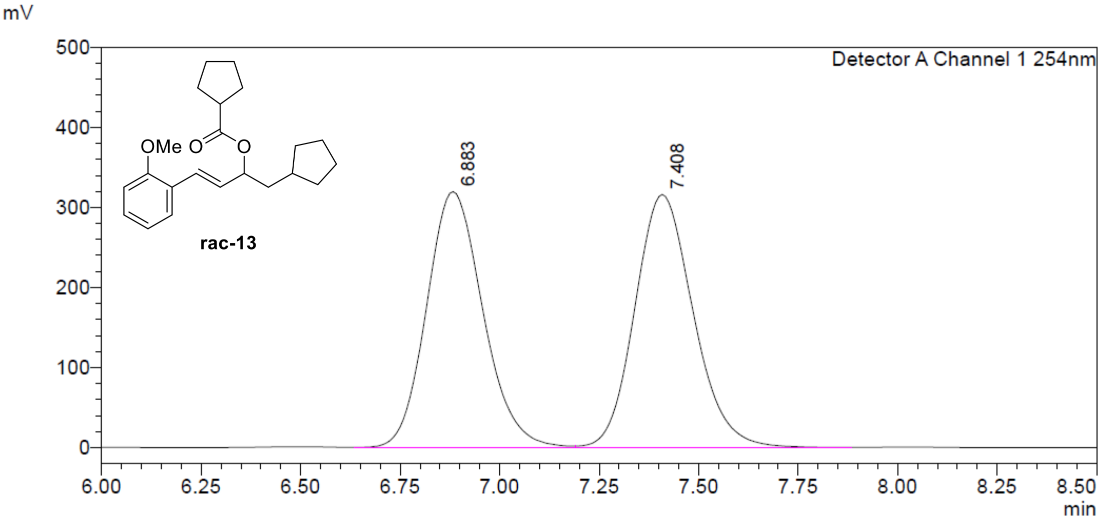

<Peak Table>

Detector A Channel 1 254nm

Peak#	Ret. Time	Height	Height%	Area	Area%
1	6.883	319643	50.277	3146013	49.366
2	7.408	316121	49.723	3226876	50.634
Total		635765	100.000	6372890	100.000

Supplementary Figure 325. Racemic Chromatogram of compound 13

<Chromatogram>

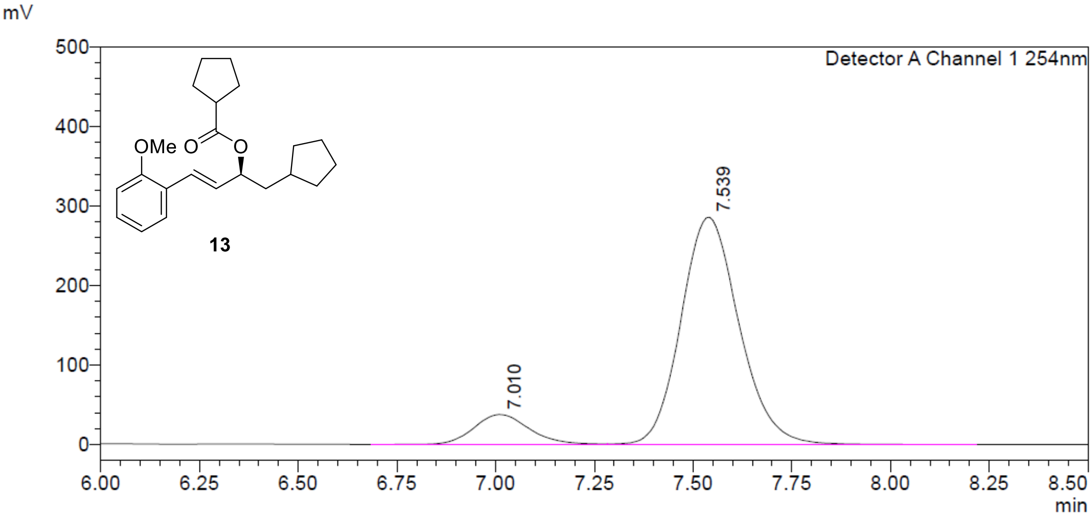

<Peak Table>

Detector A Channel 1 254nm

Peak#	Ret. Time	Height	Height%	Area	Area%
1	7.010	37686	11.650	375074	11.346
2	7.539	285802	88.350	2930683	88.654
Total		323489	100.000	3305757	100.000

Supplementary Figure 326. Scalemic Chromatogram of compound 13

<Chromatogram>

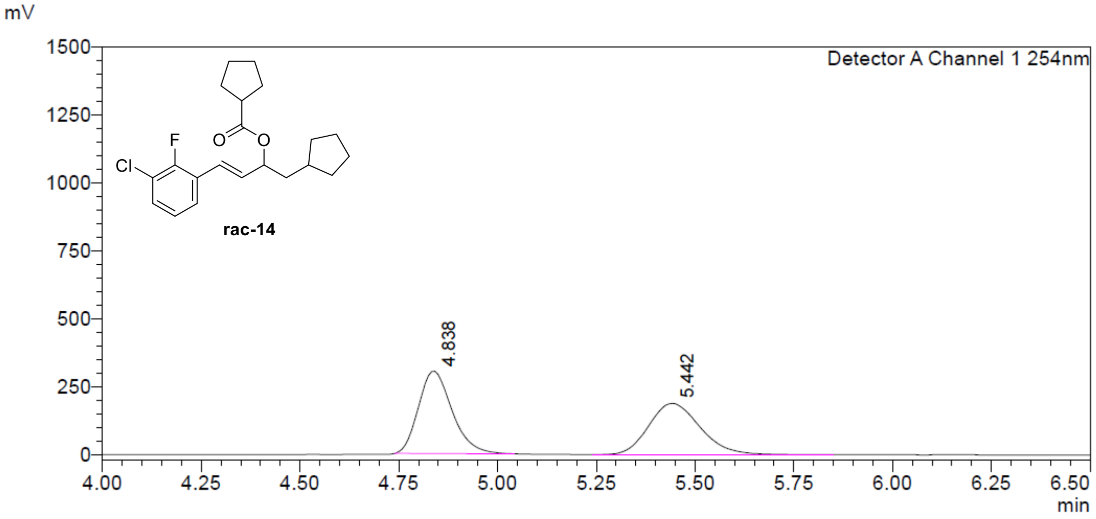

<Peak Table>

Detector A Channel 1 254nm

Peak#	Ret. Time	Height	Height%	Area	Area%
1	4.838	302546	61.496	1793521	50.839
2	5.442	189427	38.504	1734293	49.161
Total		491973	100.000	3527814	100.000

Supplementary Figure 327. Racemic Chromatogram of compound **14**

<Chromatogram>

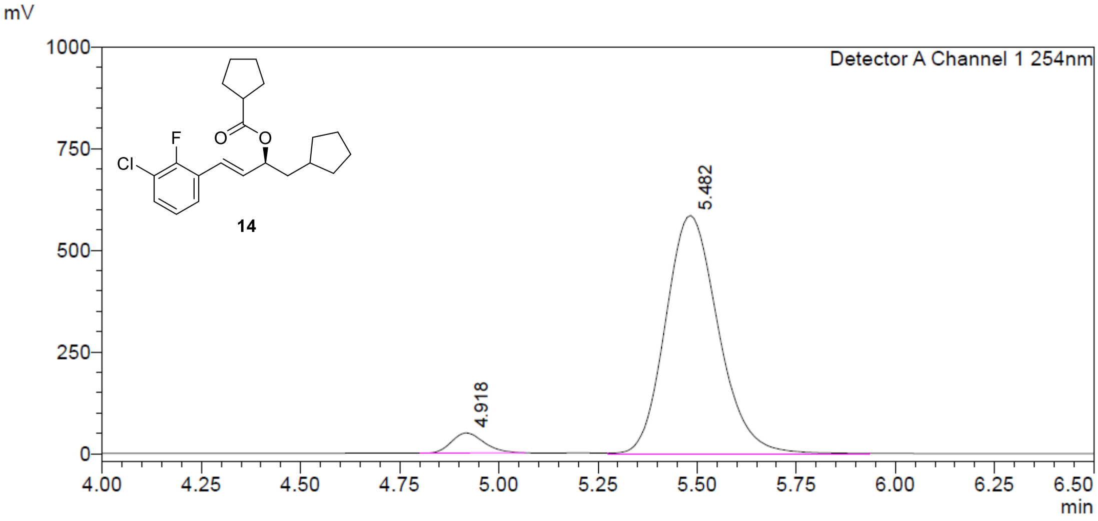

<Peak Table>

Detector A Channel 1 254nm

Peak#	Ret. Time	Height	Height%	Area	Area%
1	4.918	49245	7.757	279190	4.916
2	5.482	585594	92.243	5399505	95.084
Total		634839	100.000	5678696	100.000

Supplementary Figure 328. Scalemic Chromatogram of compound **14**

<Chromatogram>

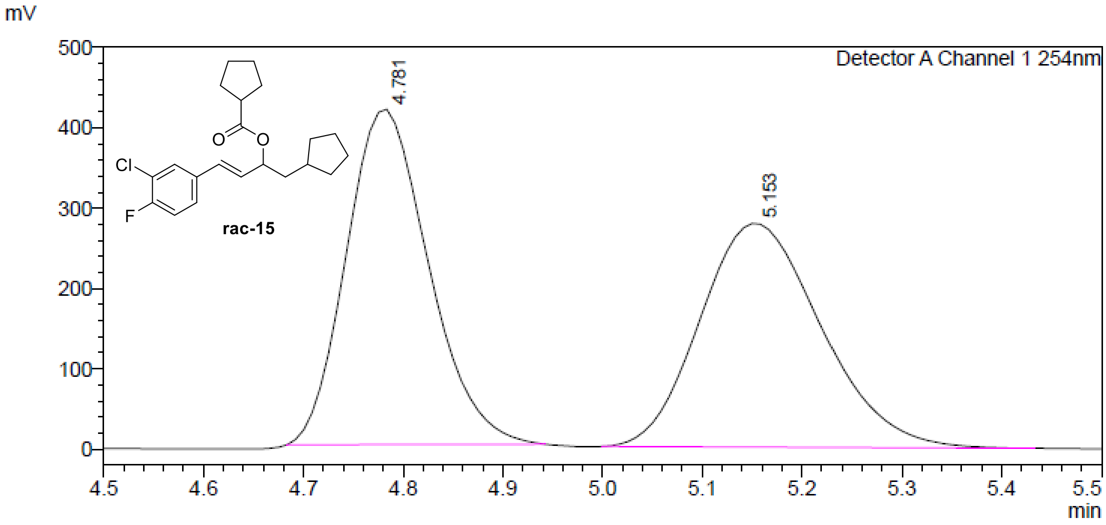

<Peak Table>

Detector A Channel 1 254nm

Peak#	Ret. Time	Height	Height%	Area	Area%
1	4.781	417068	59.932	2395080	50.462
2	5.153	278832	40.068	2351247	49.538
Total		695899	100.000	4746327	100.000

Supplementary Figure 329. Racemic Chromatogram of compound **15**

<Chromatogram>

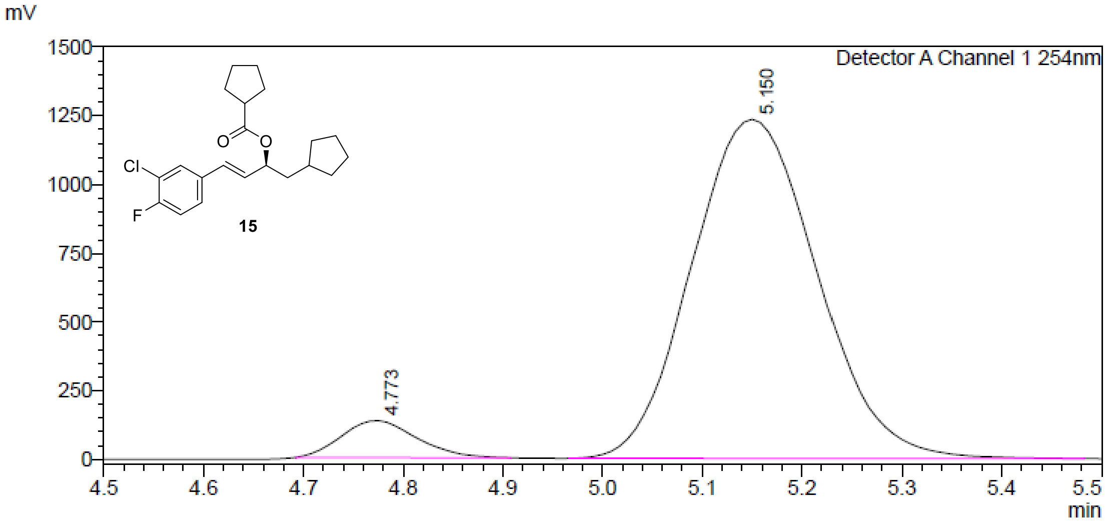

<Peak Table>

Detector A Channel 1 254nm

Peak#	Ret. Time	Height	Height%	Area	Area%
1	4.773	135204	9.873	706264	6.155
2	5.150	1234235	90.127	10768623	93.845
Total		1369439	100.000	11474887	100.000

Supplementary Figure 330. Scalemic Chromatogram of compound **15**

<Chromatogram>

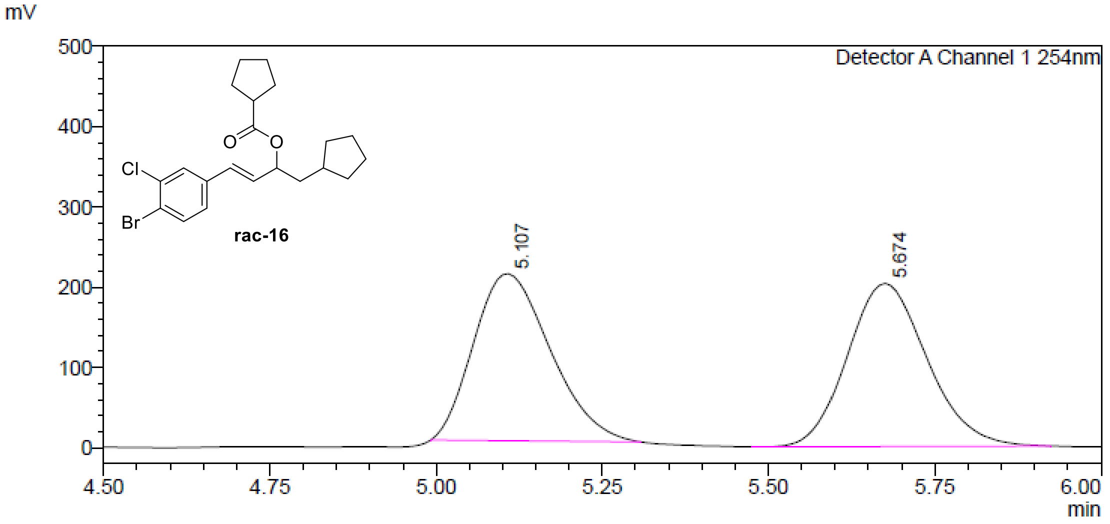

<Peak Table>

Detector A Channel 1 254nm

Peak#	Ret. Time	Height	Height%	Area	Area%
1	5.107	208532	50.625	1677603	49.732
2	5.674	203383	49.375	1695673	50.268
Total		411914	100.000	3373276	100.000

Supplementary Figure 331. Racemic Chromatogram of compound **16**

<Chromatogram>

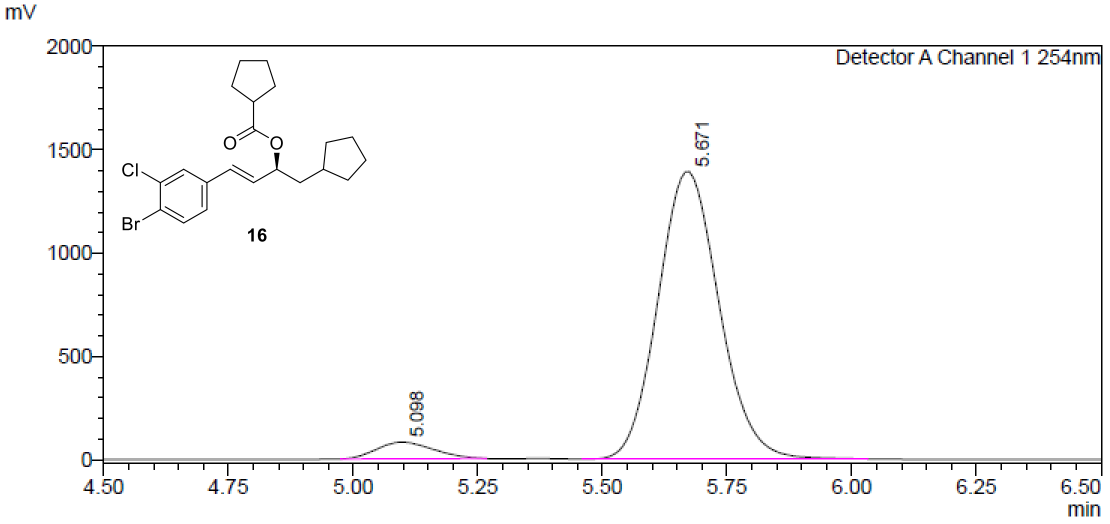

<Peak Table>

Detector A Channel 1 254nm

Peak#	Ret. Time	Height	Height%	Area	Area%
1	5.098	79907	5.430	634355	5.060
2	5.671	1391555	94.570	11902476	94.940
Total		1471462	100.000	12536831	100.000

Supplementary Figure 332. Scalemic Chromatogram of compound **16**

<Chromatogram>

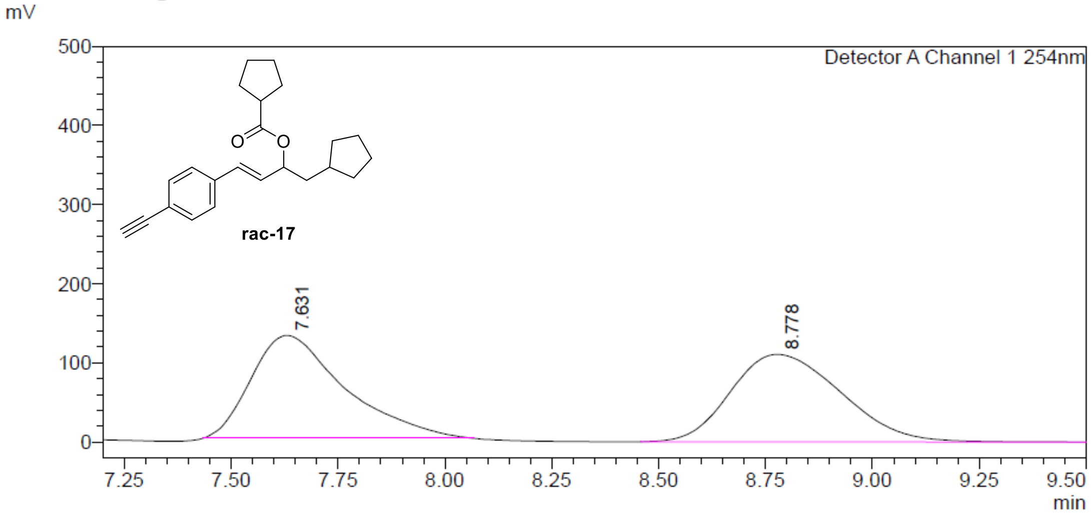

<Peak Table>

Detector A Channel 1 254nm

Peak#	Ret. Time	Height	Height%	Area	Area%
1	7.631	130212	54.119	1980367	50.026
2	8.778	110393	45.881	1978337	49.974
Total		240605	100.000	3958704	100.000

Supplementary Figure 333. Racemic Chromatogram of compound **17**

<Chromatogram>

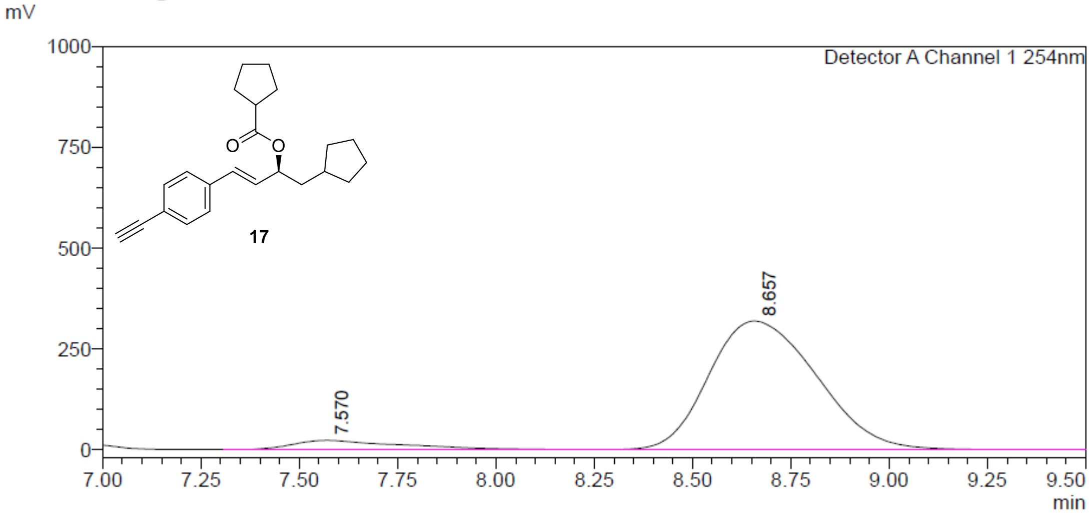

<Peak Table>

Detector A Channel 1 254nm

Peak#	Ret. Time	Height	Height%	Area	Area%
1	7.570	22961	6.716	458015	6.983
2	8.657	318950	93.284	6100651	93.017
Total		341911	100.000	6558666	100.000

Supplementary Figure 334. Scalemic Chromatogram of compound **17**

<Chromatogram>

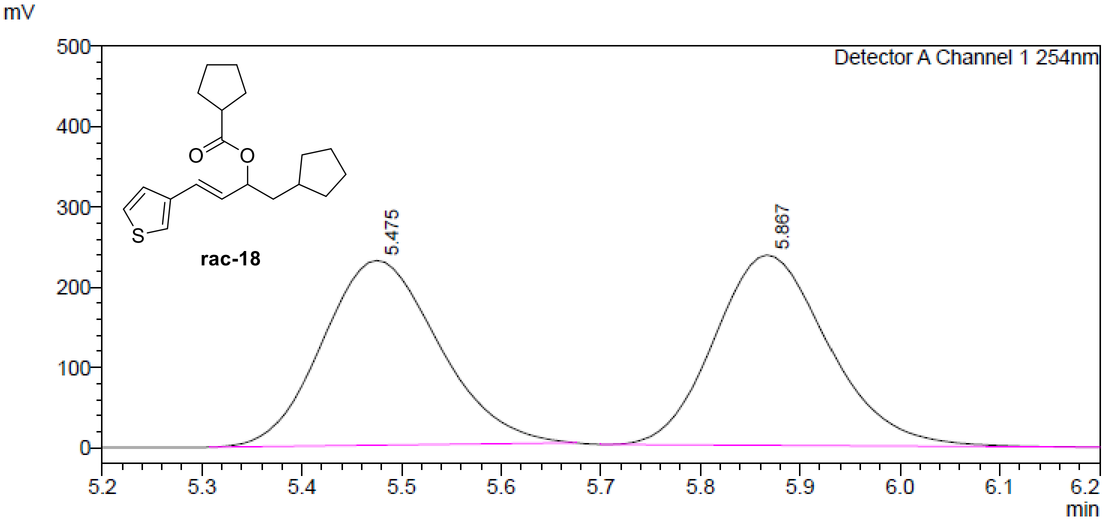

<Peak Table>

Detector A Channel 1 254nm

Peak#	Ret. Time	Height	Height%	Area	Area%
1	5.475	230447	49.237	1875781	49.824
2	5.867	237586	50.763	1889006	50.176
Total		468033	100.000	3764787	100.000

Supplementary Figure 335. Racemic Chromatogram of compound **18**

<Chromatogram>

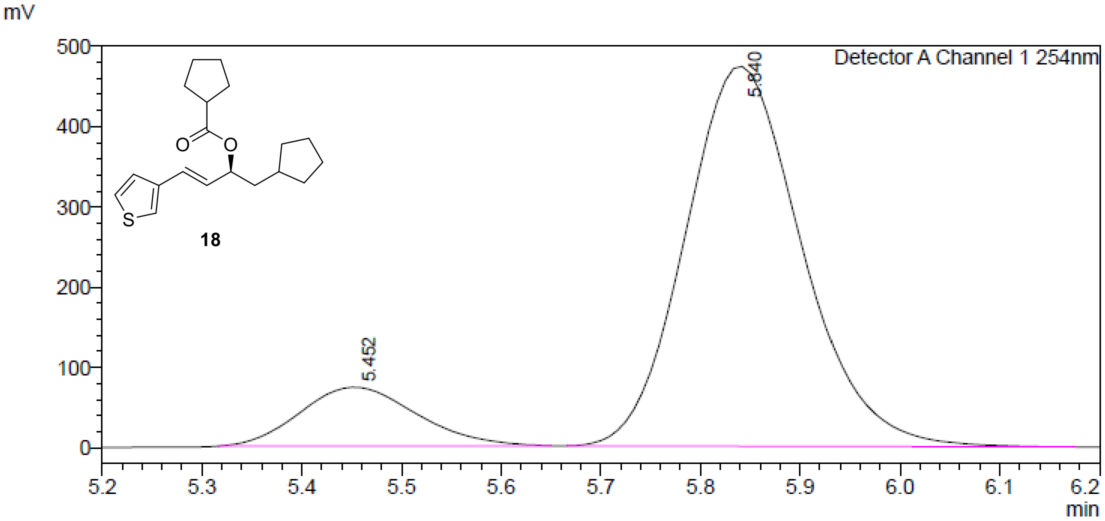

<Peak Table>

Detector A Channel 1 254nm

Peak#	Ret. Time	Height	Height%	Area	Area%
1	5.452	73284	13.410	595140	13.411
2	5.840	473198	86.590	3842537	86.589
Total		546482	100.000	4437677	100.000

Supplementary Figure 336. Scalemic Chromatogram of compound **18**

<Chromatogram>

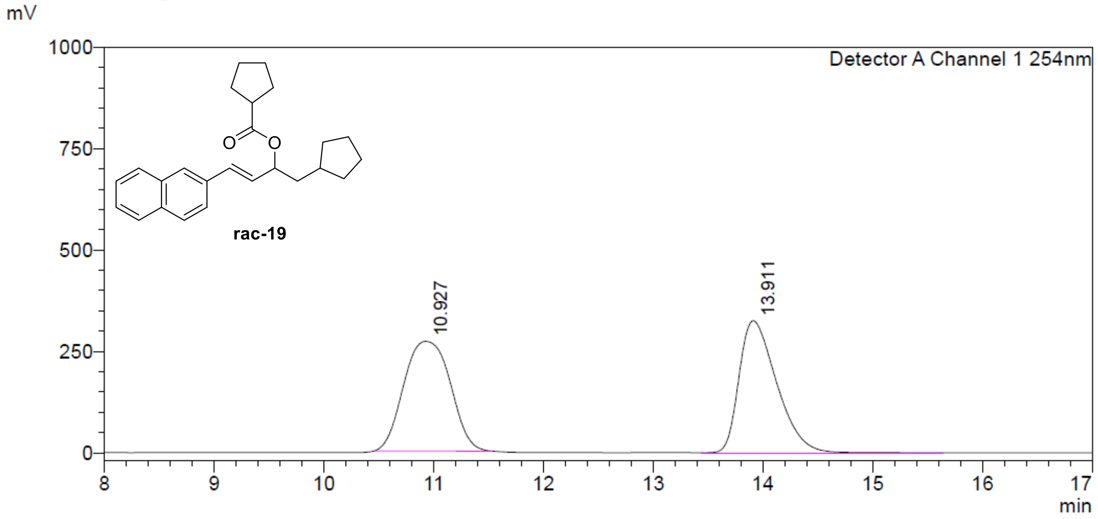

<Peak Table>

Detector A Channel 1 254nm

Peak#	Ret. Time	Height	Height%	Area	Area%
1	10.927	270344	45.385	7982864	50.798
2	13.911	325327	54.615	7731934	49.202
Total		595671	100.000	15714797	100.000

Supplementary Figure 337. Racemic Chromatogram of compound **19**

<Chromatogram>

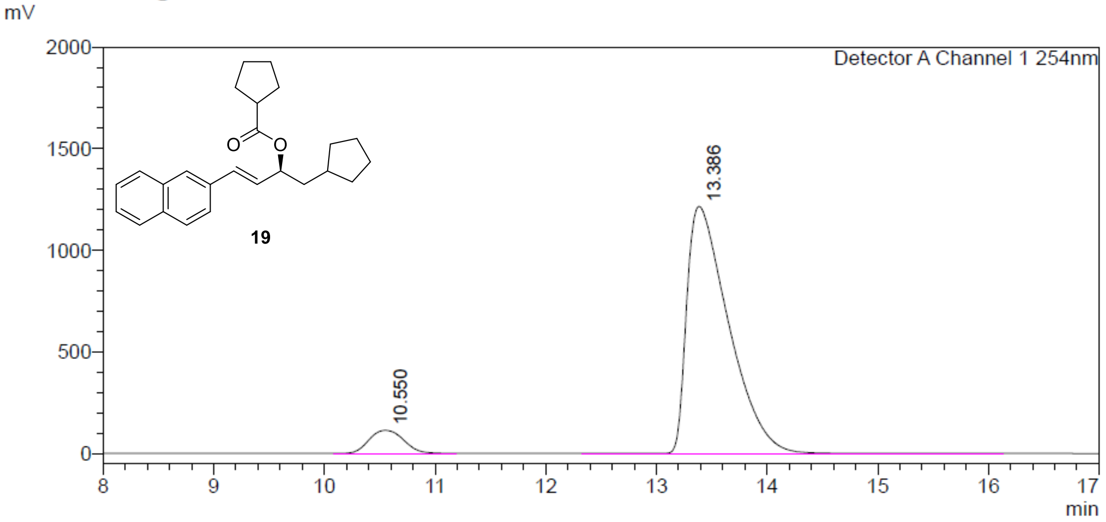

<Peak Table>

Detector A Channel 1 254nm

Peak#	Ret. Time	Height	Height%	Area	Area%
1	10.550	113221	8.517	2459133	7.137
2	13.386	1216208	91.483	31998584	92.863
Total		1329429	100.000	34457717	100.000

Supplementary Figure 338. Scalemic Chromatogram of compound **19**

<Chromatogram>

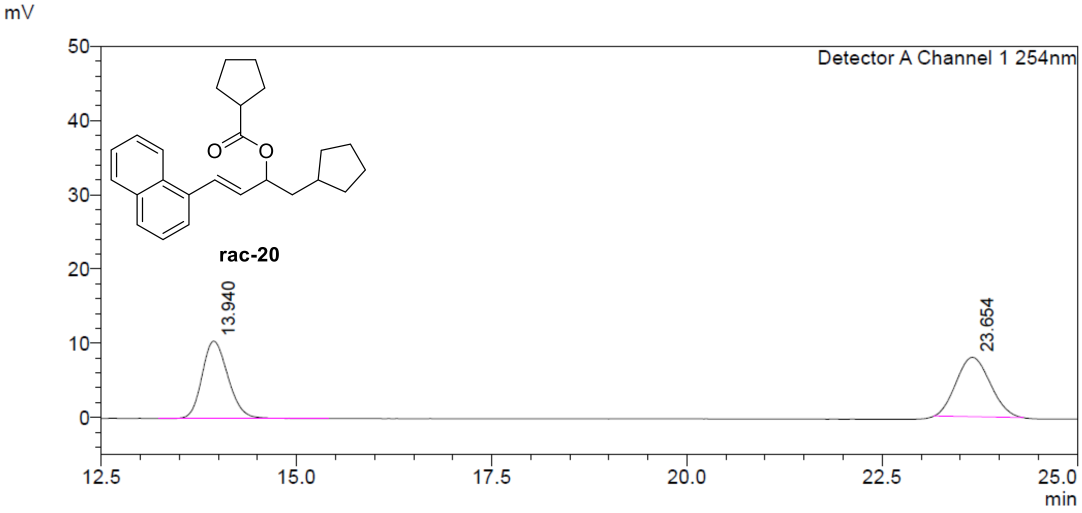

<Peak Table>

Detector A Channel 1 254nm

Peak#	Ret. Time	Height	Height%	Area	Area%
1	13.940	10410	56.574	239708	49.679
2	23.654	7991	43.426	242803	50.321
Total		18400	100.000	482512	100.000

Supplementary Figure 339. Racemic Chromatogram of compound **20**

<Chromatogram>

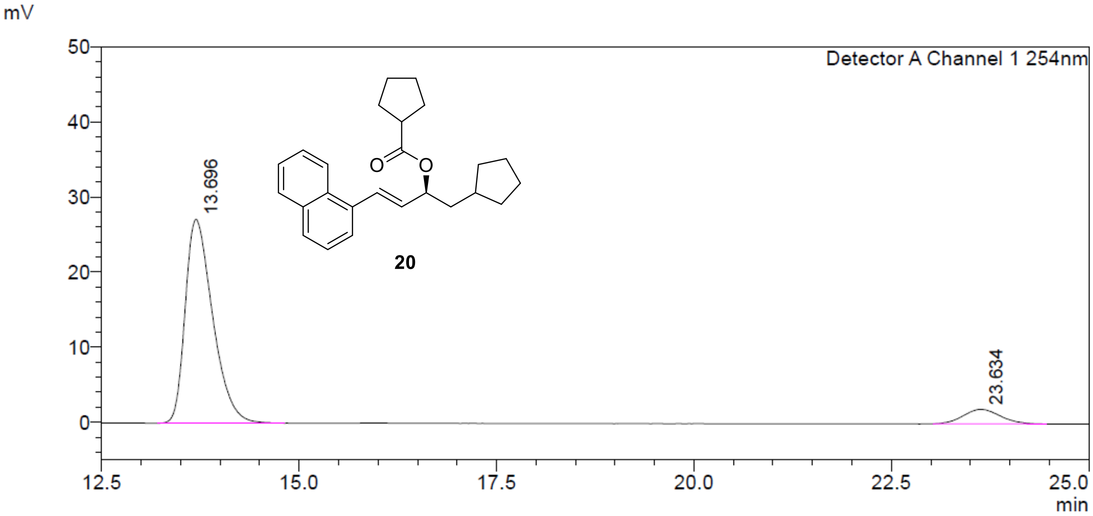

<Peak Table>

Detector A Channel 1 254nm

Peak#	Ret. Time	Height	Height%	Area	Area%
1	13.696	27121	93.381	656909	91.245
2	23.634	1922	6.619	63034	8.755
Total		29043	100.000	719943	100.000

Supplementary Figure 340. Scalemic Chromatogram of compound **20**

<Chromatogram>

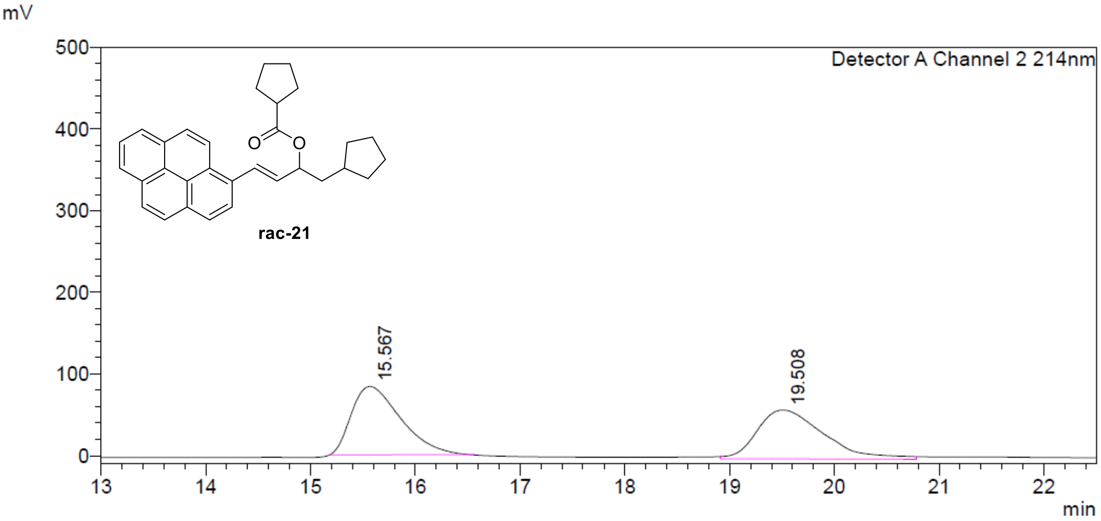

<Peak Table>

Detector A Channel 2 214nm

Peak#	Ret. Time	Height	Height%	Area	Area%
1	15.567	83541	58.257	2728248	50.824
2	19.508	59860	41.743	2639789	49.176
Total		143401	100.000	5368038	100.000

Supplementary Figure 341. Racemic Chromatogram of compound **21**

<Chromatogram>

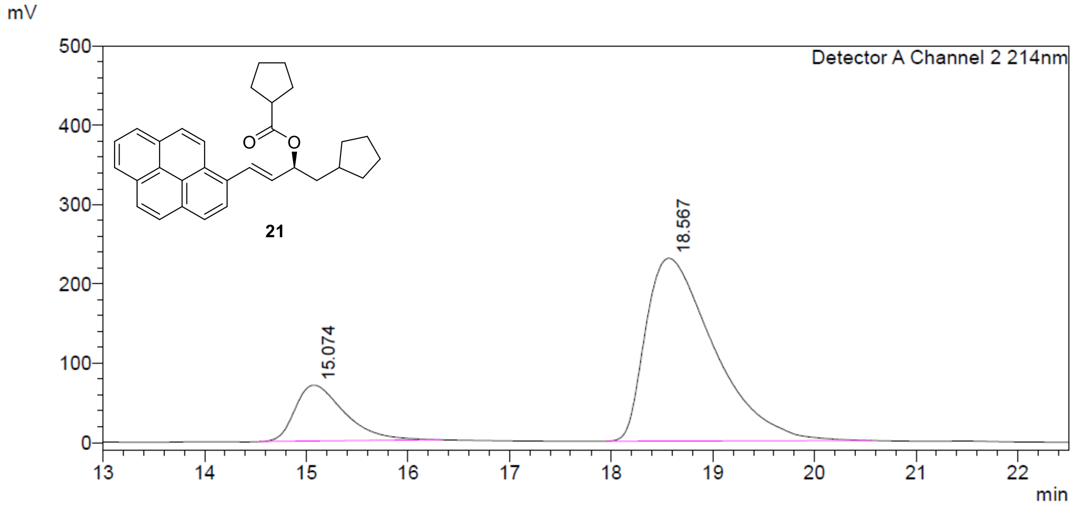

<Peak Table>

Detector A Channel 1 254nm

Peak#	Ret. Time	Height	Height%	Area	Area%
1	15.071	84103	23.492	2826870	17.461
2	18.566	273904	76.508	13362659	82.539
Total		358007	100.000	16189529	100.000

Supplementary Figure 342. Scalemic Chromatogram of compound **21**

<Chromatogram>

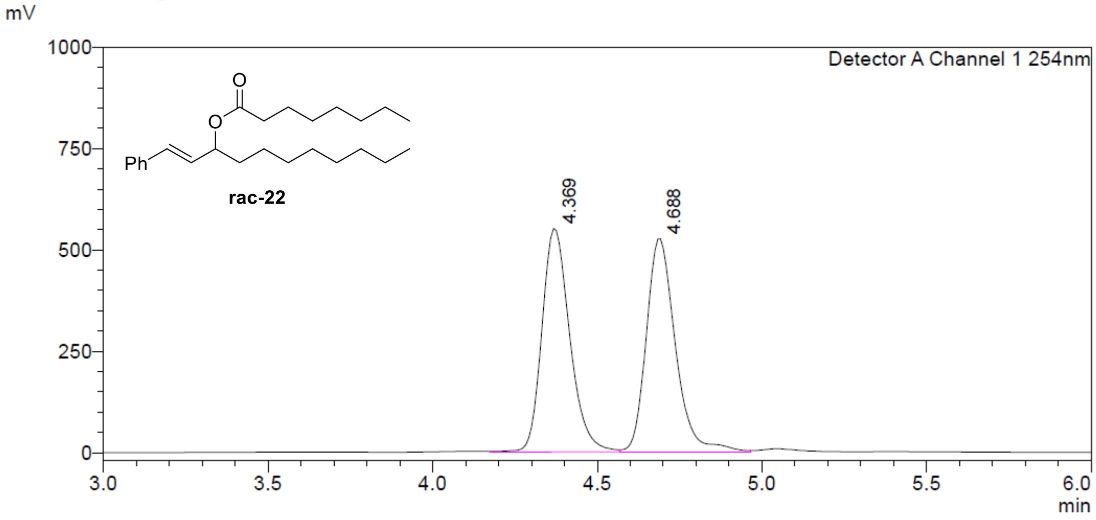

<Peak Table>

Detector A Channel 1 254nm

Peak#	Ret. Time	Height	Height%	Area	Area%
1	4.369	551600	51.223	3244761	50.026
2	4.688	525250	48.777	3241439	49.974
Total		1076850	100.000	6486200	100.000

Supplementary Figure 343. Racemic Chromatogram of compound **22**

<Chromatogram>

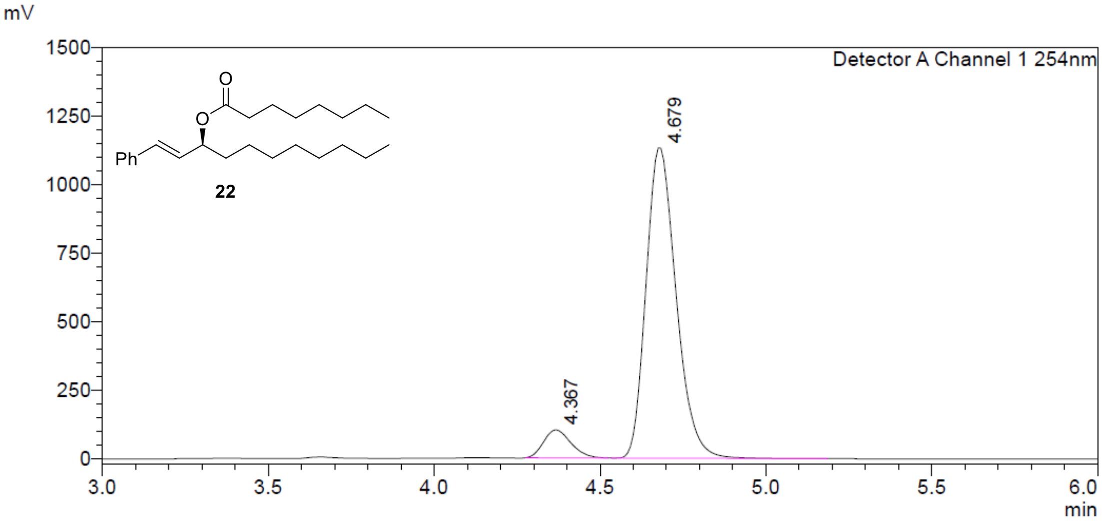

<Peak Table>

Detector A Channel 1 254nm

Peak#	Ret. Time	Height	Height%	Area	Area%
1	4.367	102655	8.315	586218	7.582
2	4.679	1131989	91.685	7145131	92.418
Total		1234644	100.000	7731349	100.000

Supplementary Figure 344. Scalemic Chromatogram of compound **22**

<Chromatogram>

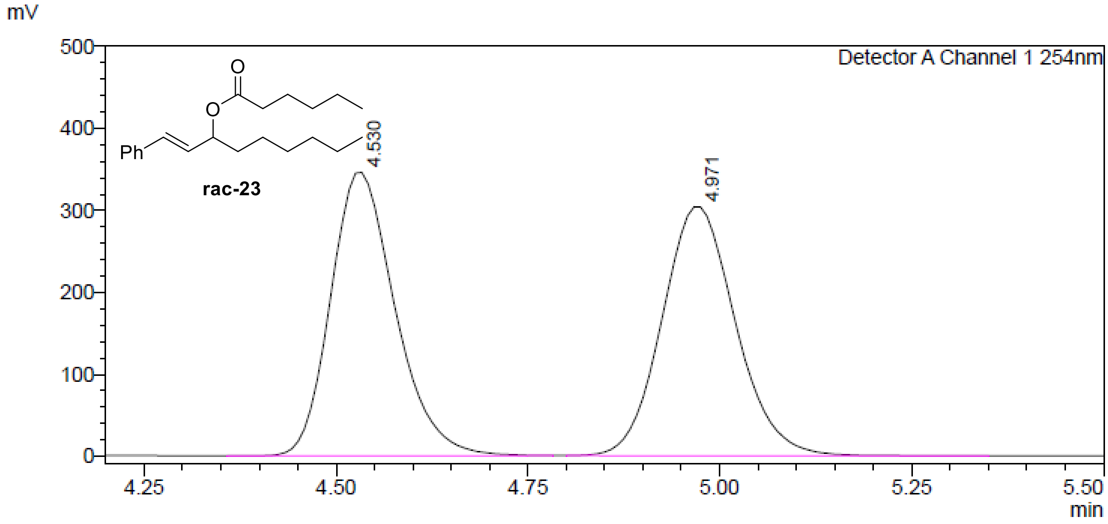

<Peak Table>

Detector A Channel 1 254nm

Peak#	Ret. Time	Height	Height%	Area	Area%
1	4.530	346454	53.250	2027385	50.006
2	4.971	304164	46.750	2026893	49.994
Total		650618	100.000	4054278	100.000

Supplementary Figure 345. Racemic Chromatogram of compound **23**

<Chromatogram>

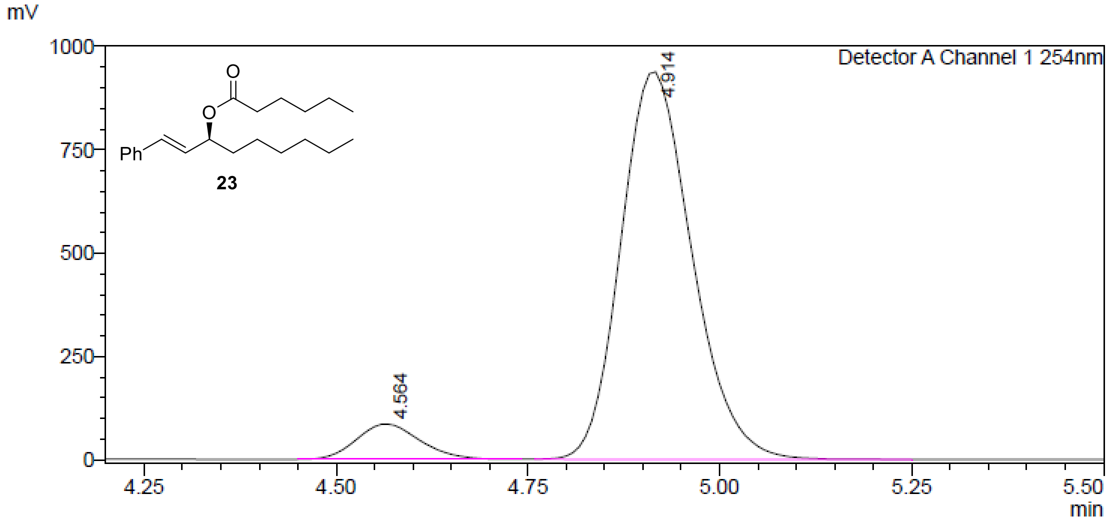

<Peak Table>

Detector A Channel 1 254nm

Peak#	Ret. Time	Height	Height%	Area	Area%
1	4.564	85700	8.377	496148	7.567
2	4.914	937315	91.623	6060907	92.433
Total		1023015	100.000	6557055	100.000

Supplementary Figure 346. Scalemic Chromatogram of compound **23**

<Chromatogram>

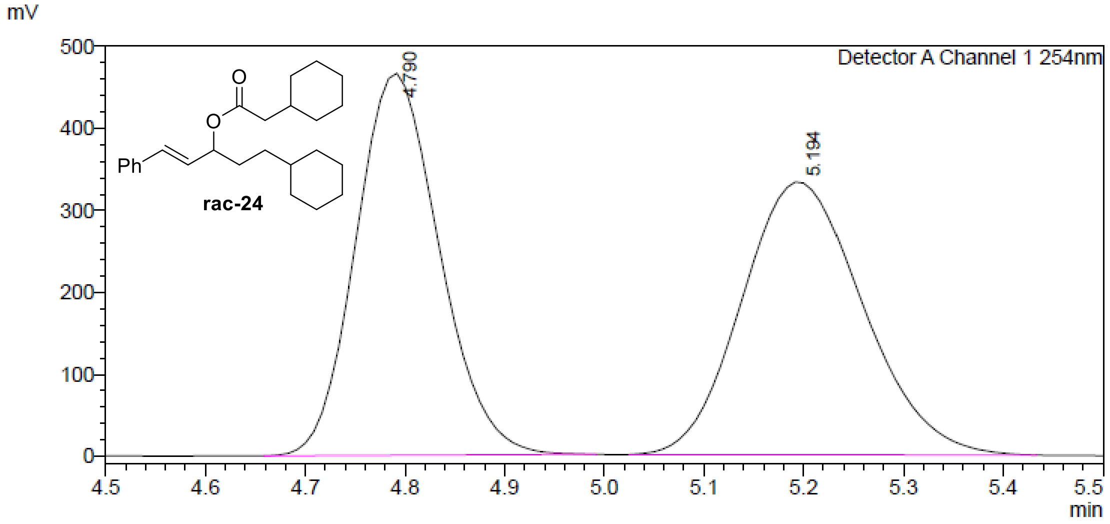

<Peak Table>

Detector A Channel 1 254nm

Peak#	Ret. Time	Height	Height%	Area	Area%
1	4.790	465965	58.314	2721306	49.167
2	5.194	333092	41.686	2813479	50.833
Total		799058	100.000	5534785	100.000

Supplementary Figure 347. Racemic Chromatogram of compound **24**

<Chromatogram>

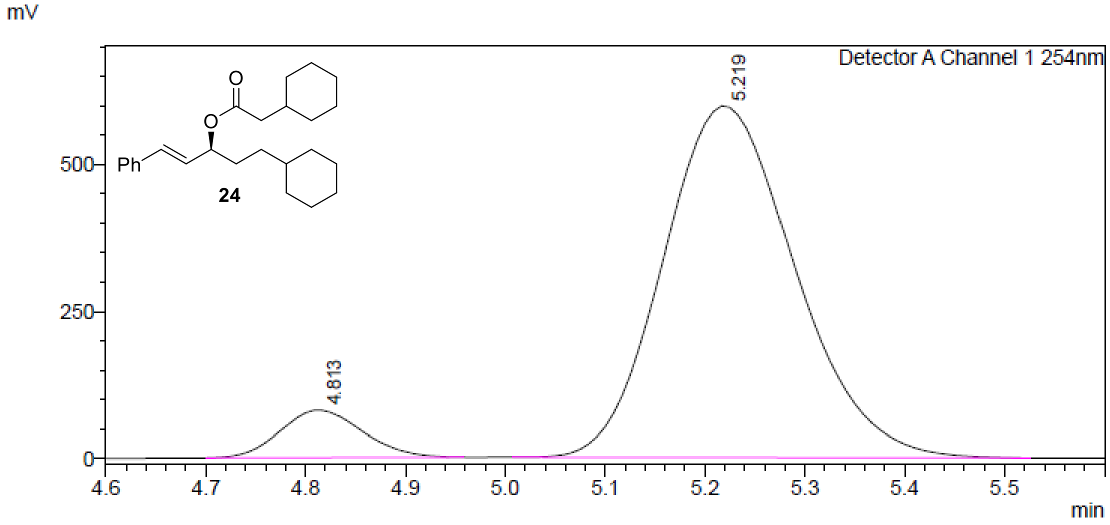

<Peak Table>

Detector A Channel 1 254nm

Peak#	Ret. Time	Height	Height%	Area	Area%
1	4.813	80127	11.830	459587	7.750
2	5.219	597218	88.170	5470321	92.250
Total		677345	100.000	5929908	100.000

Supplementary Figure 348. Scalemic Chromatogram of compound **24**

<Chromatogram>

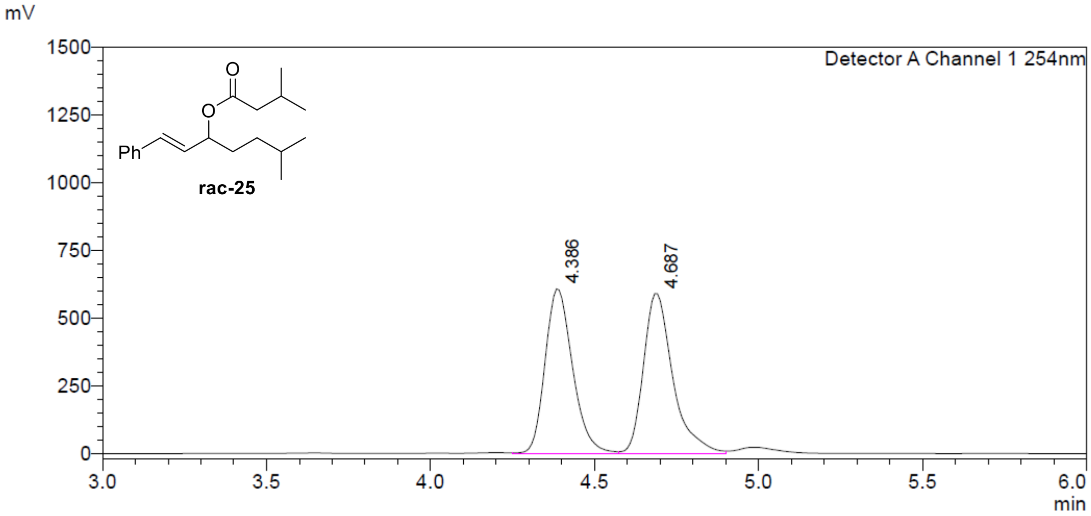

<Peak Table>

Detector A Channel 1 254nm

Peak#	Ret. Time	Height	Height%	Area	Area%
1	4.386	607192	50.698	3546705	49.063
2	4.687	590470	49.302	3682184	50.937
Total		1197662	100.000	7228889	100.000

Supplementary Figure 349. Racemic Chromatogram of compound **25**

<Chromatogram>

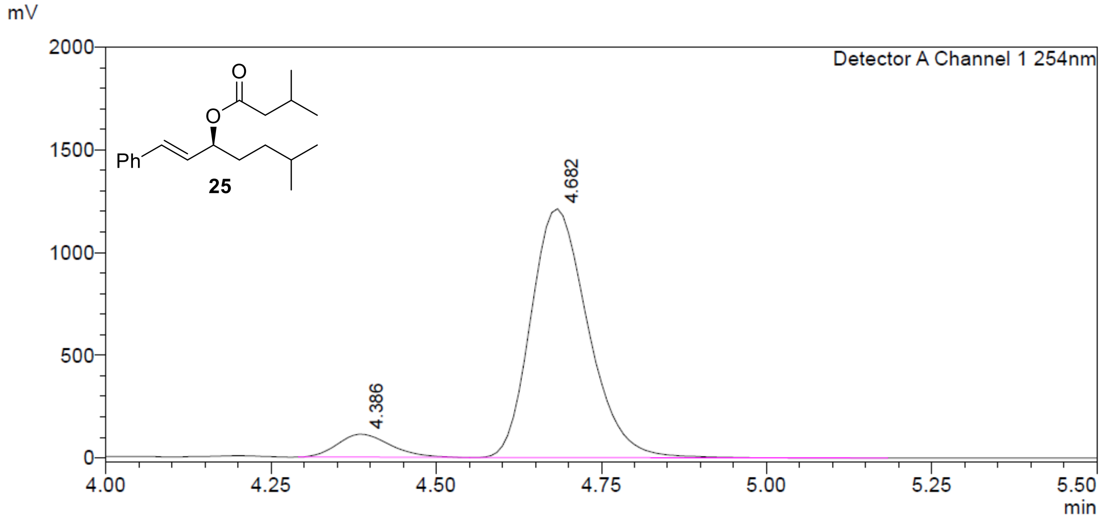

<Peak Table>

Detector A Channel 1 254nm

Peak#	Ret. Time	Height	Height%	Area	Area%
1	4.386	112064	8.470	625660	7.901
2	4.682	1211057	91.530	7293125	92.099
Total		1323120	100.000	7918785	100.000

Supplementary Figure 350. Scalemic Chromatogram of compound **25**

<Chromatogram>

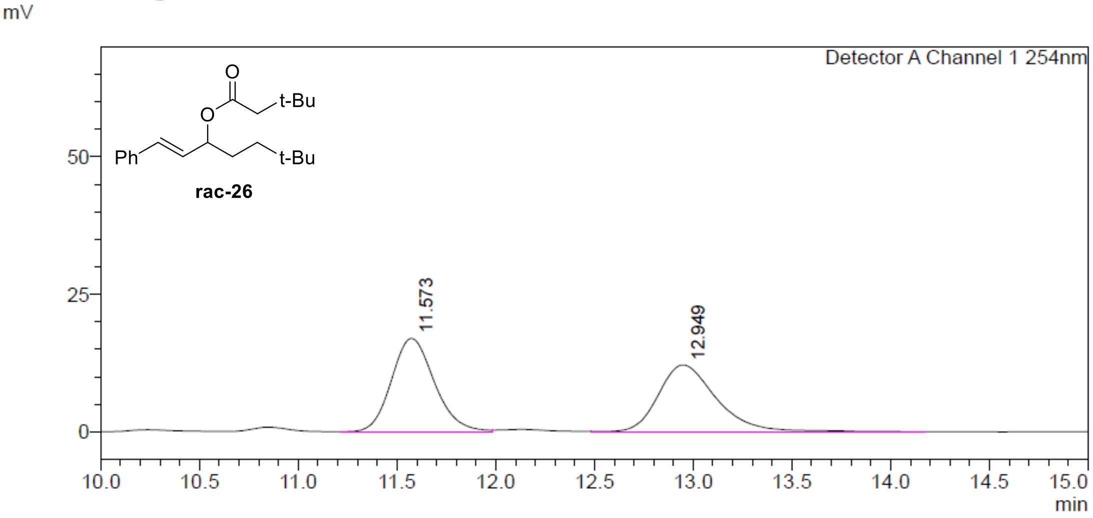

<Peak Table>

Detector A Channel 1 254nm

Peak#	Ret. Time	Height	Height%	Area	Area%
1	11.573	16965	58.265	258240	50.647
2	12.949	12152	41.735	251642	49.353
Total		29117	100.000	509883	100.000

Supplementary Figure 351. Racemic Chromatogram of compound **26**

<Chromatogram>

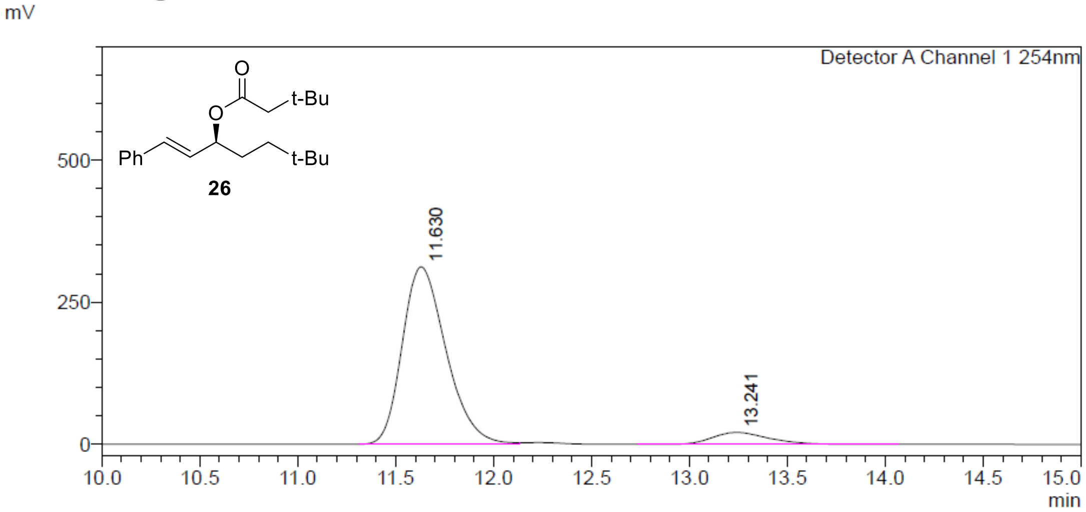

<Peak Table>

Detector A Channel 1 254nm

Peak#	Ret. Time	Height	Height%	Area	Area%
1	11.630	311893	93.720	4871939	92.064
2	13.241	20898	6.280	419943	7.936
Total		332791	100.000	5291882	100.000

Supplementary Figure 352. Scalemic Chromatogram of compound **26**

<Chromatogram>

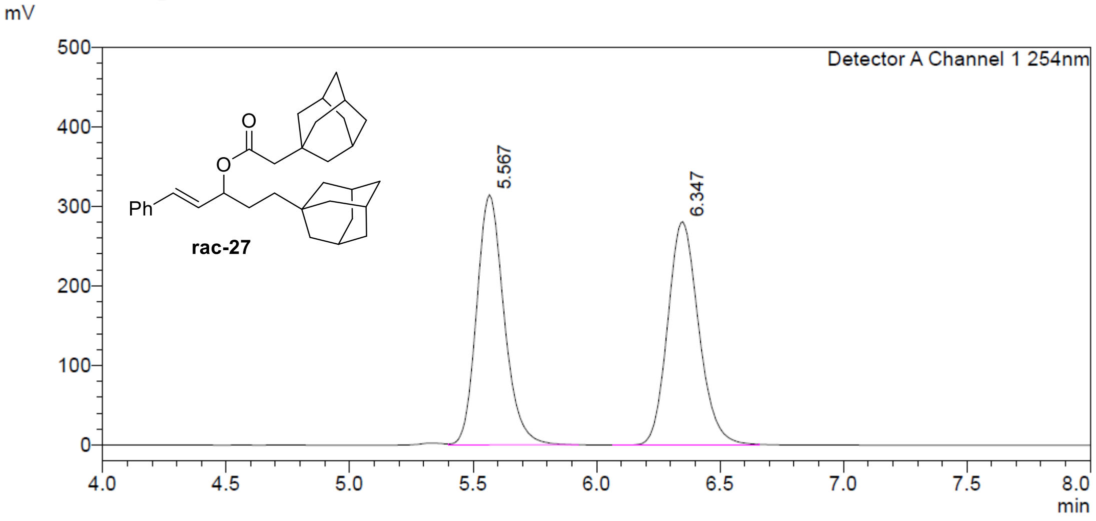

<Peak Table>

Detector A Channel 1 254nm

Peak#	Ret. Time	Height	Height%	Area	Area%
1	5.567	314300	52.841	2430641	49.758
2	6.347	280501	47.159	2454328	50.242
Total		594802	100.000	4884969	100.000

Supplementary Figure 353. Racemic Chromatogram of compound **27**

<Chromatogram>

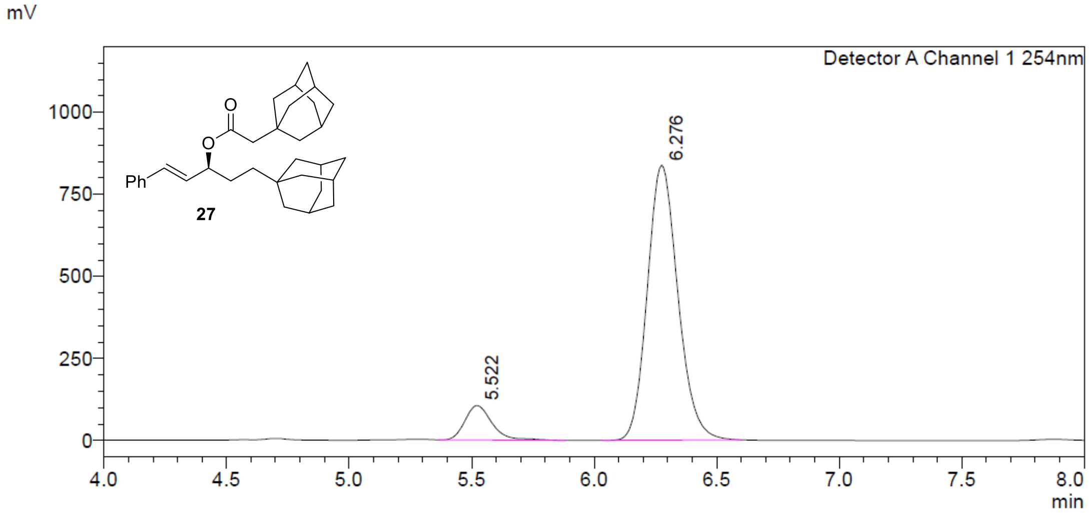

<Peak Table>

Detector A Channel 1 254nm

Peak#	Ret. Time	Height	Height%	Area	Area%
1	5.522	104832	11.127	807657	9.997
2	6.276	837314	88.873	7271262	90.003
Total		942146	100.000	8078919	100.000

Supplementary Figure 354. Scalemic Chromatogram of compound **27**

<Chromatogram>

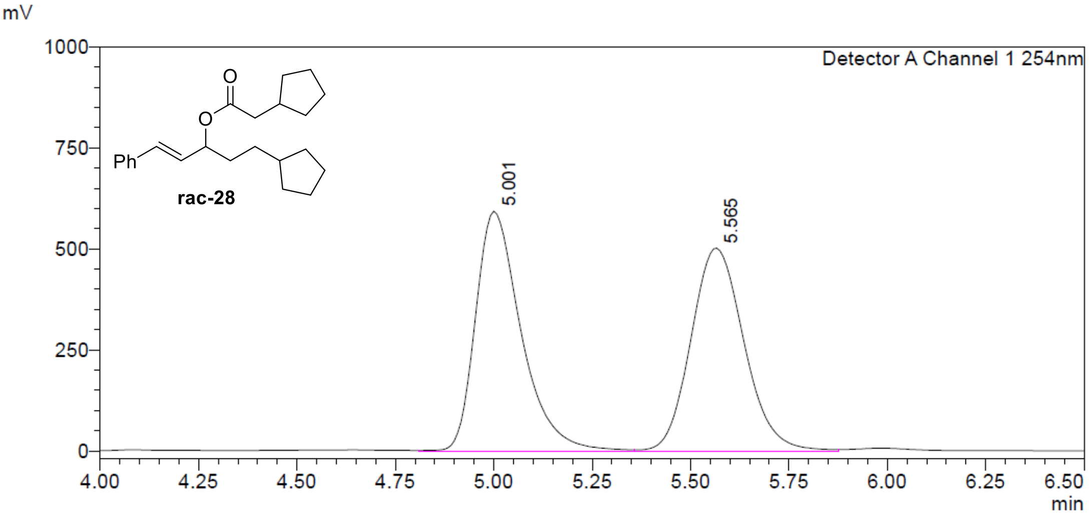

<Peak Table>

Detector A Channel 1 254nm

Peak#	Ret. Time	Height	Height%	Area	Area%
1	5.001	593058	54.166	4762102	50.336
2	5.565	501837	45.834	4698463	49.664
Total		1094894	100.000	9460565	100.000

Supplementary Figure 355. Racemic Chromatogram of compound **28**

<Chromatogram>

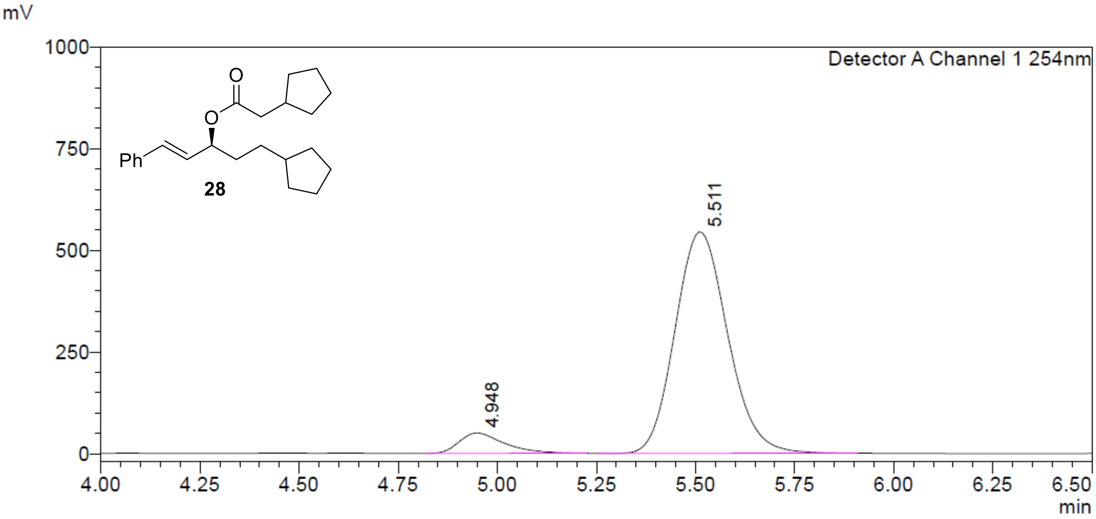

<Peak Table>

Detector A Channel 1 254nm

Peak#	Ret. Time	Height	Height%	Area	Area%
1	4.948	50253	8.448	398559	7.346
2	5.511	544581	91.552	5026720	92.654
Total		594834	100.000	5425278	100.000

Supplementary Figure 356. Scalemic Chromatogram of compound **28**

<Chromatogram>

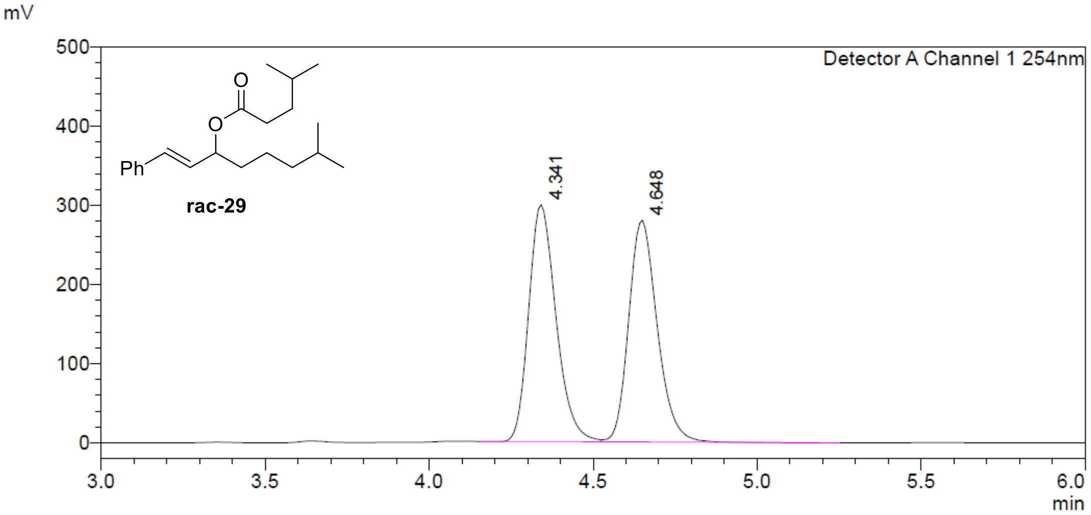

<Peak Table>

Detector A Channel 1 254nm

Peak#	Ret. Time	Height	Height%	Area	Area%
1	4.341	299195	51.652	1730648	50.669
2	4.648	280058	48.348	1684926	49.331
Total		579254	100.000	3415574	100.000

Supplementary Figure 357. Racemic Chromatogram of compound **29**

<Chromatogram>

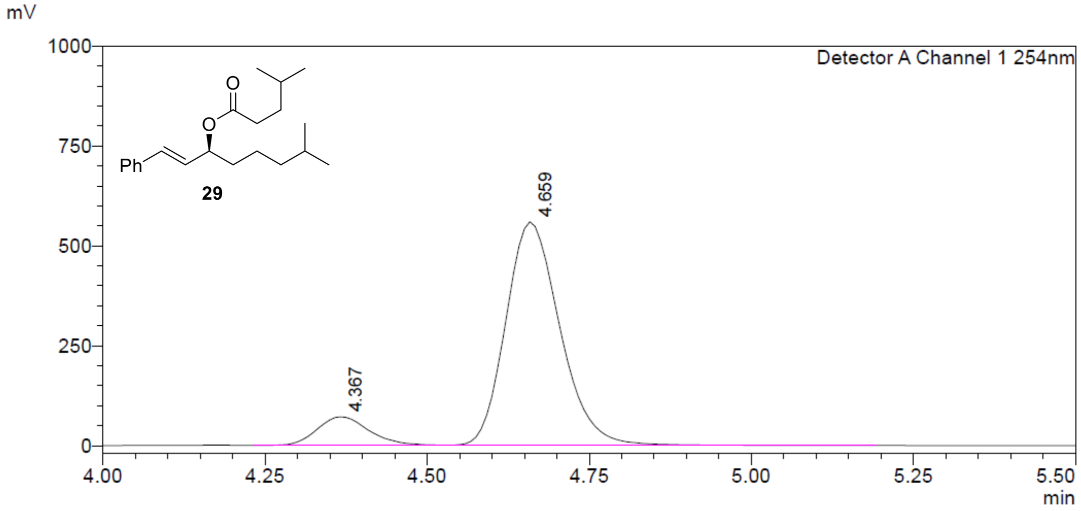

<Peak Table>

Detector A Channel 1 254nm

Peak#	Ret. Time	Height	Height%	Area	Area%
1	4.367	71583	11.339	398228	10.918
2	4.659	559693	88.661	3249184	89.082
Total		631276	100.000	3647412	100.000

Supplementary Figure 358. Scalemic Chromatogram of compound **29**

<Chromatogram>

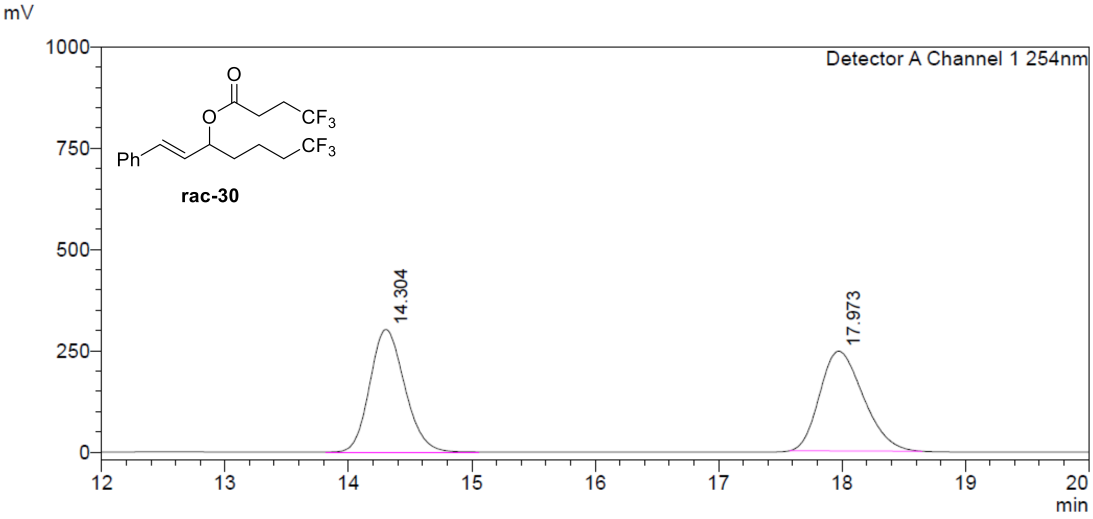

<Peak Table>

Detector A Channel 1 254nm

Peak#	Ret. Time	Height	Height%	Area	Area%
1	14.304	302632	55.147	5940952	49.557
2	17.973	246136	44.853	6047242	50.443
Total		548768	100.000	11988193	100.000

Supplementary Figure 359. Racemic Chromatogram of compound **30**

<Chromatogram>

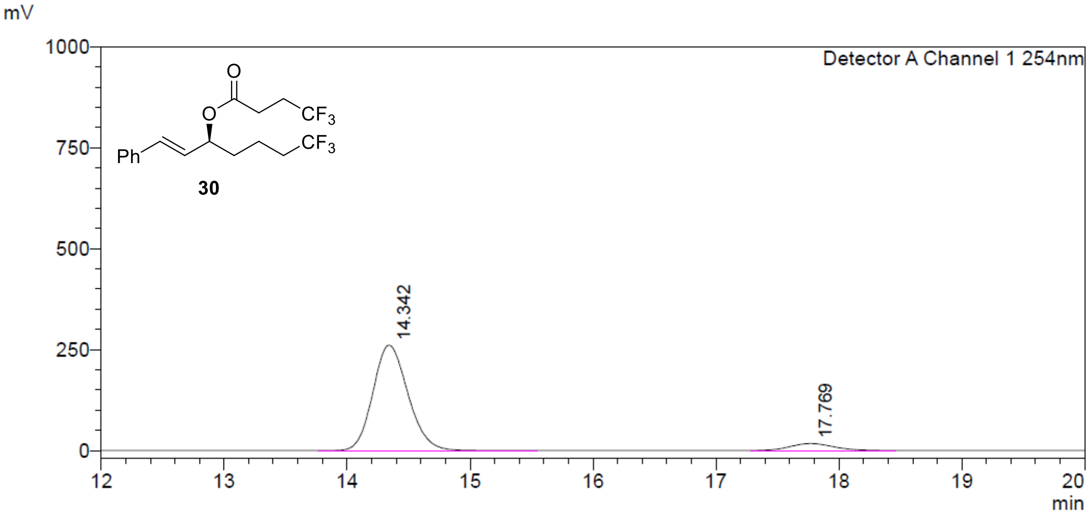

<Peak Table>

Detector A Channel 1 254nm

Peak#	Ret. Time	Height	Height%	Area	Area%
1	14.342	260933	93.853	5168230	92.513
2	17.769	17090	6.147	418250	7.487
Total		278022	100.000	5586480	100.000

Supplementary Figure 360. Scalemic Chromatogram of compound **30**

<Chromatogram>

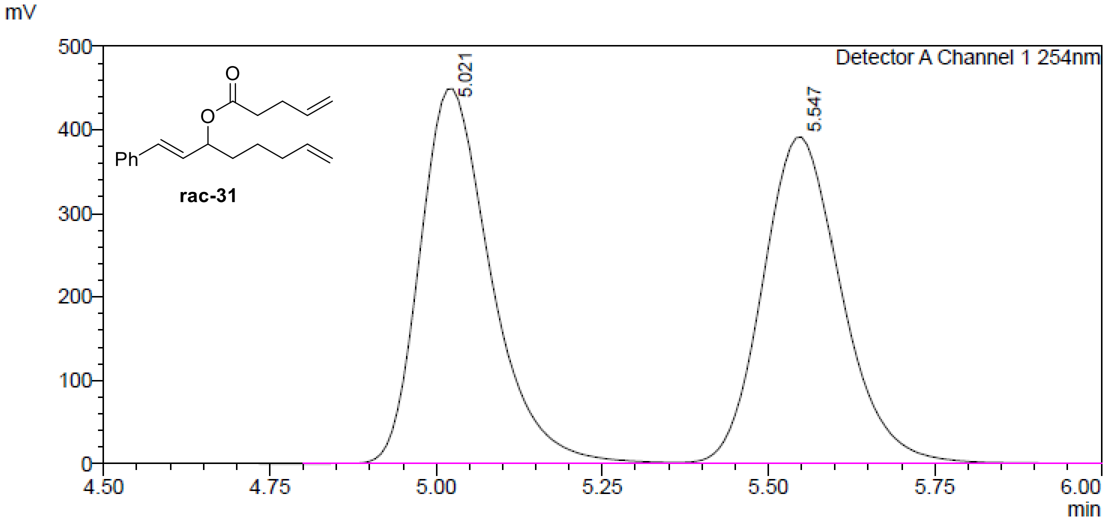

<Peak Table>

Detector A Channel 1 254nm

Peak#	Ret. Time	Height	Height%	Area	Area%
1	5.021	449115	53.439	3357088	50.888
2	5.547	391302	46.561	3239964	49.112
Total		840417	100.000	6597052	100.000

Supplementary Figure 361. Racemic Chromatogram of compound **31**

<Chromatogram>

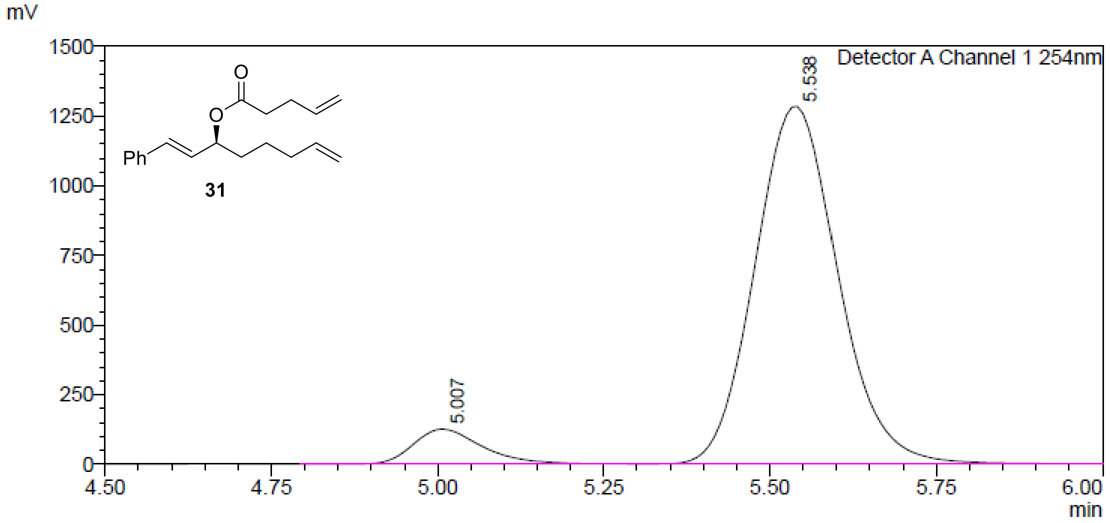

<Peak Table>

Detector A Channel 1 254nm

Peak#	Ret. Time	Height	Height%	Area	Area%
1	5.007	126481	8.970	926583	7.661
2	5.538	1283567	91.030	11168845	92.339
Total		1410047	100.000	12095428	100.000

Supplementary Figure 362. Scalemic Chromatogram of compound **31**

<Chromatogram>

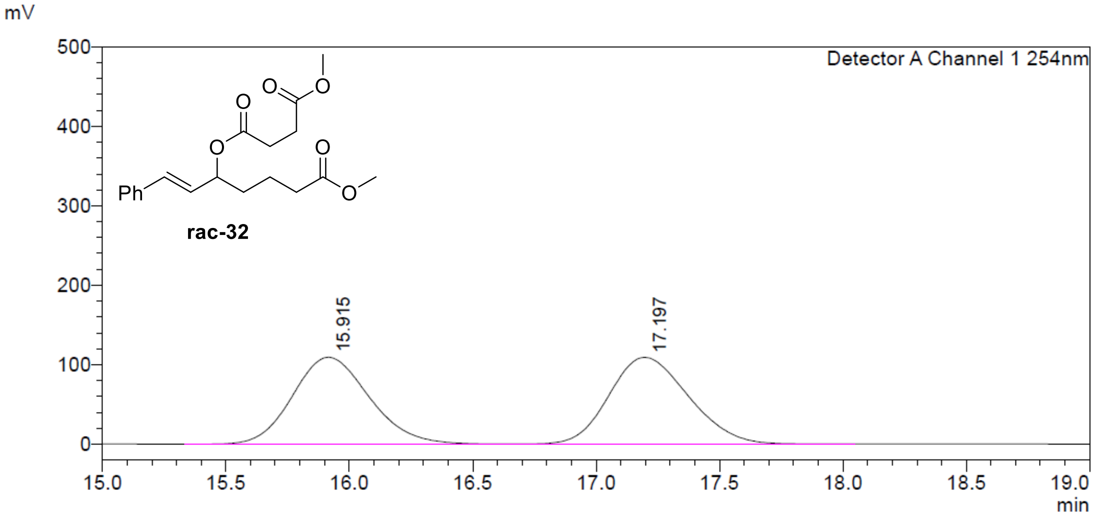

<Peak Table>

Detector A Channel 1 254nm

Peak#	Ret. Time	Height	Height%	Area	Area%
1	15.915	109270	50.033	2371748	49.168
2	17.197	109128	49.967	2451996	50.832
Total		218398	100.000	4823744	100.000

Supplementary Figure 363. Racemic Chromatogram of compound **32**

<Chromatogram>

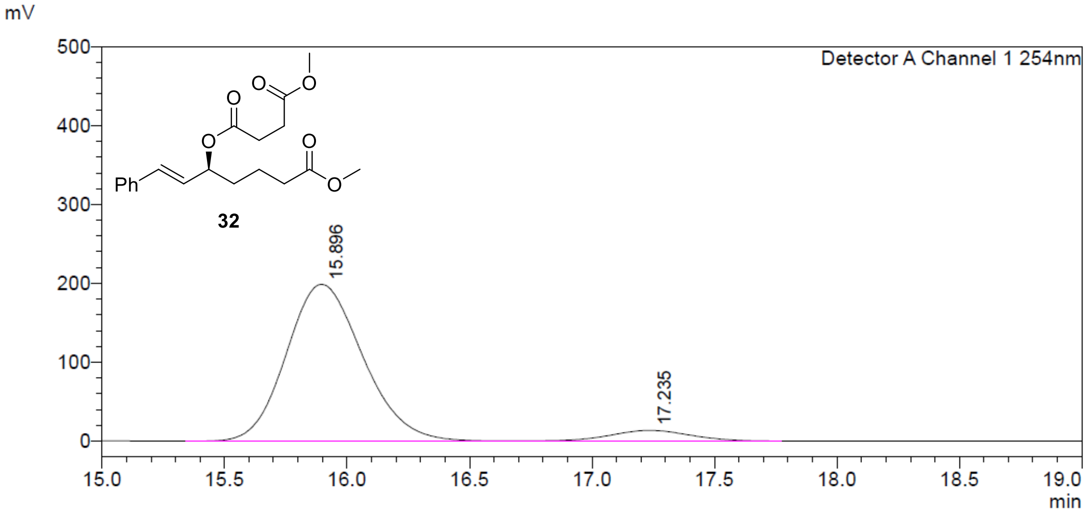

<Peak Table>

Detector A Channel 1 254nm

Peak#	Ret. Time	Height	Height%	Area	Area%
1	15.896	198756	93.607	4329472	93.192
2	17.235	13574	6.393	316276	6.808
Total		212331	100.000	4645748	100.000

Supplementary Figure 364. Scalemic Chromatogram of compound **32**

<Chromatogram>

mV

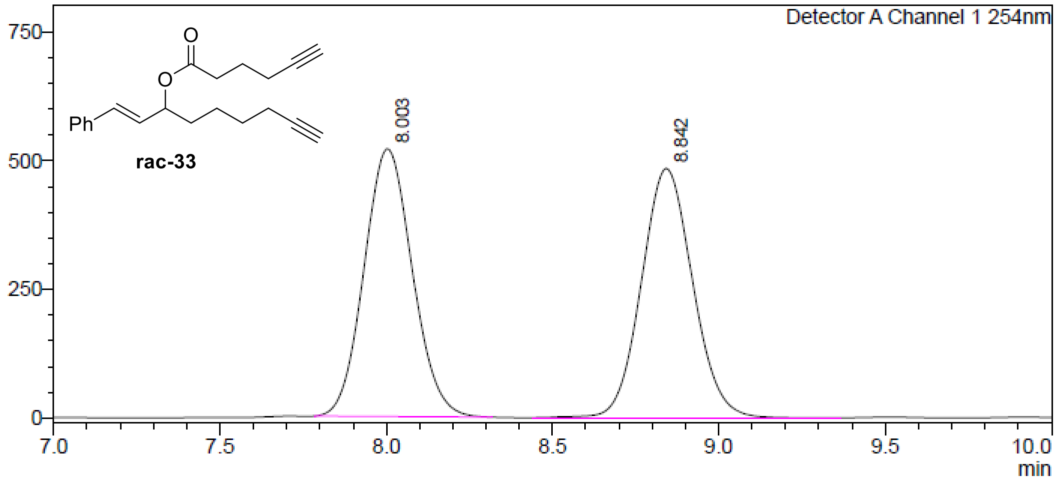

<Peak Table>

Detector A Channel 1 254nm

Peak#	Ret. Time	Height	Height%	Area	Area%
1	8.003	520122	51.778	5261518	50.333
2	8.842	484404	48.222	5191830	49.667
Total		1004526	100.000	10453347	100.000

Supplementary Figure 365. Racemic Chromatogram of compound 33

<Chromatogram>

mV

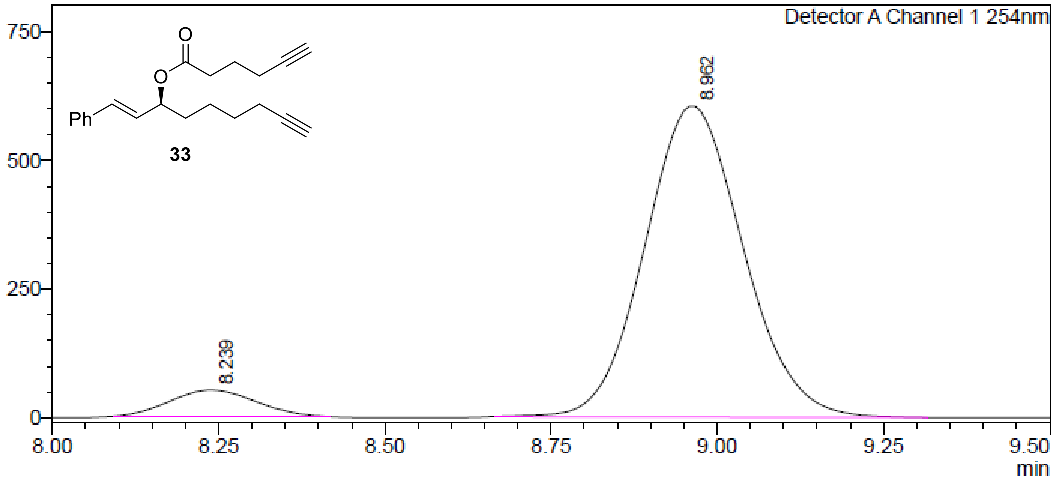

<Peak Table>

Detector A Channel 1 254nm

Peak#	Ret. Time	Height	Height%	Area	Area%
1	8.239	51248	7.836	457195	6.845
2	8.962	602768	92.164	6221840	93.155
Total		654016	100.000	6679035	100.000

Supplementary Figure 366. Scalemic Chromatogram of compound 33

<Chromatogram>

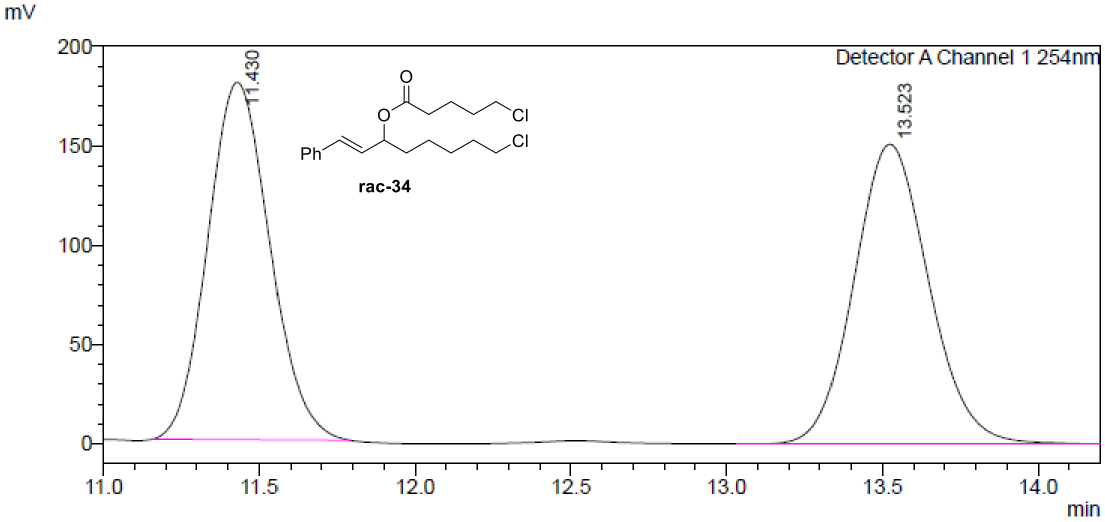

<Peak Table>

Detector A Channel 1 254nm

Peak#	Ret. Time	Height	Height%	Area	Area%
1	11.430	179732	54.385	2494499	49.734
2	13.523	150752	45.615	2521188	50.266
Total		330484	100.000	5015687	100.000

Supplementary Figure 367. Racemic Chromatogram of compound 34

<Chromatogram>

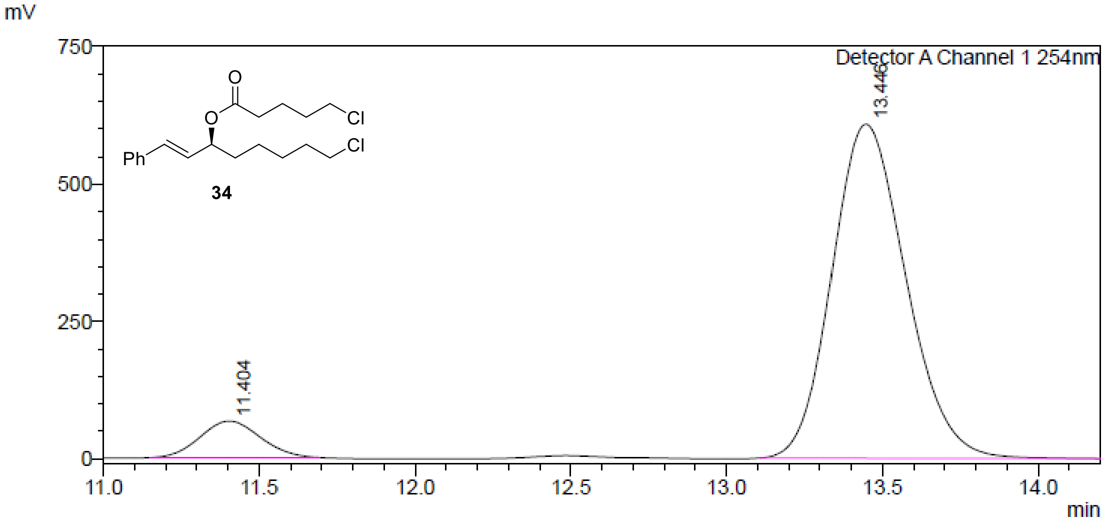

<Peak Table>

Detector A Channel 1 254nm

Peak#	Ret. Time	Height	Height%	Area	Area%
1	11.404	66590	9.882	894814	8.197
2	13.446	607250	90.118	10021584	91.803
Total		673840	100.000	10916399	100.000

Supplementary Figure 368. Scalemic Chromatogram of compound 34

<Chromatogram>

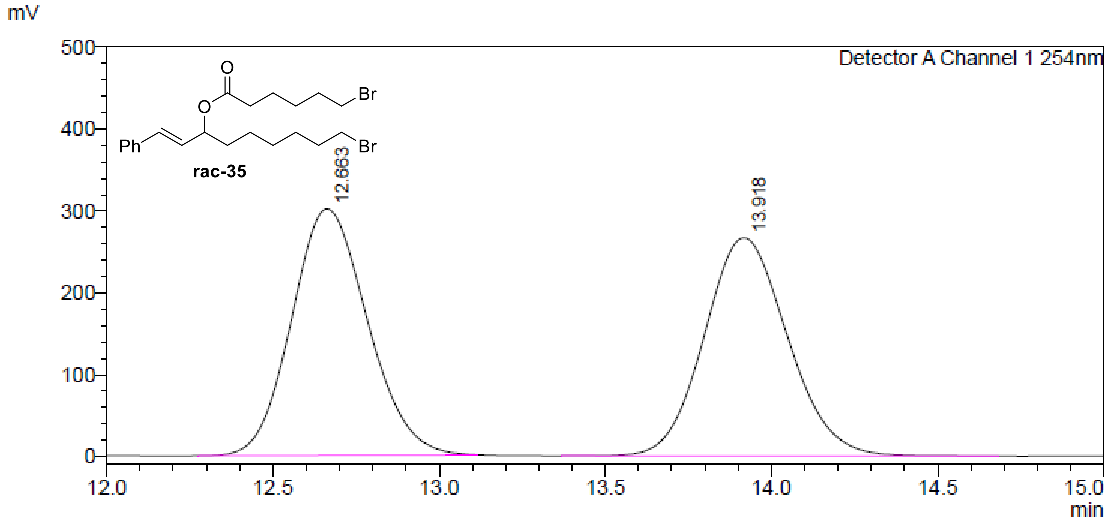

<Peak Table>

Detector A Channel 1 254nm

Peak#	Ret. Time	Height	Height%	Area	Area%
1	12.663	301647	53.027	4798227	50.637
2	13.918	267210	46.973	4677499	49.363
Total		568857	100.000	9475726	100.000

Supplementary Figure 369. Racemic Chromatogram of compound **35**

<Chromatogram>

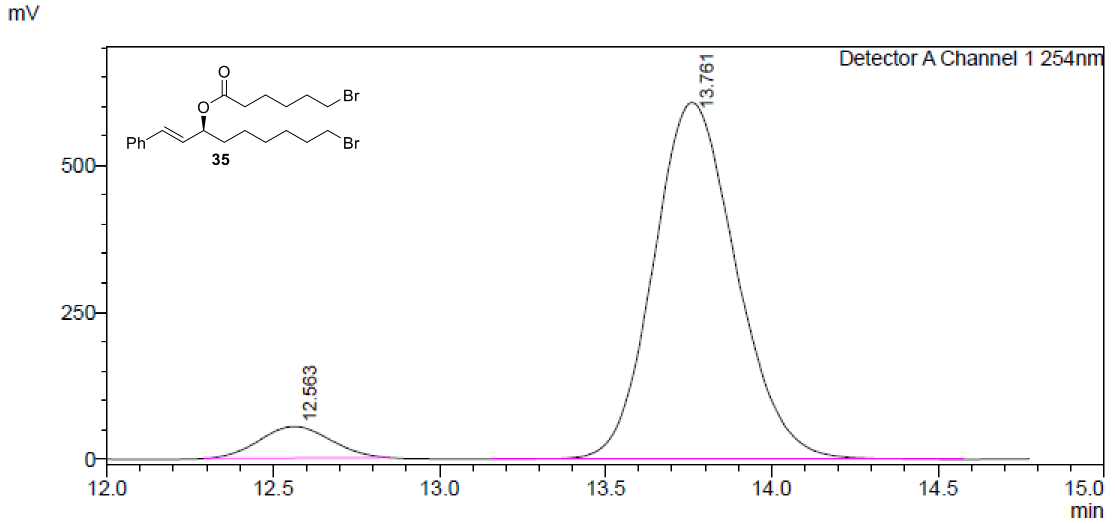

<Peak Table>

Detector A Channel 1 254nm

Peak#	Ret. Time	Height	Height%	Area	Area%
1	12.563	53749	8.142	798358	7.056
2	13.761	606427	91.858	10515644	92.944
Total		660176	100.000	11314001	100.000

Supplementary Figure 370. Scalemic Chromatogram of compound **35**

<Chromatogram>

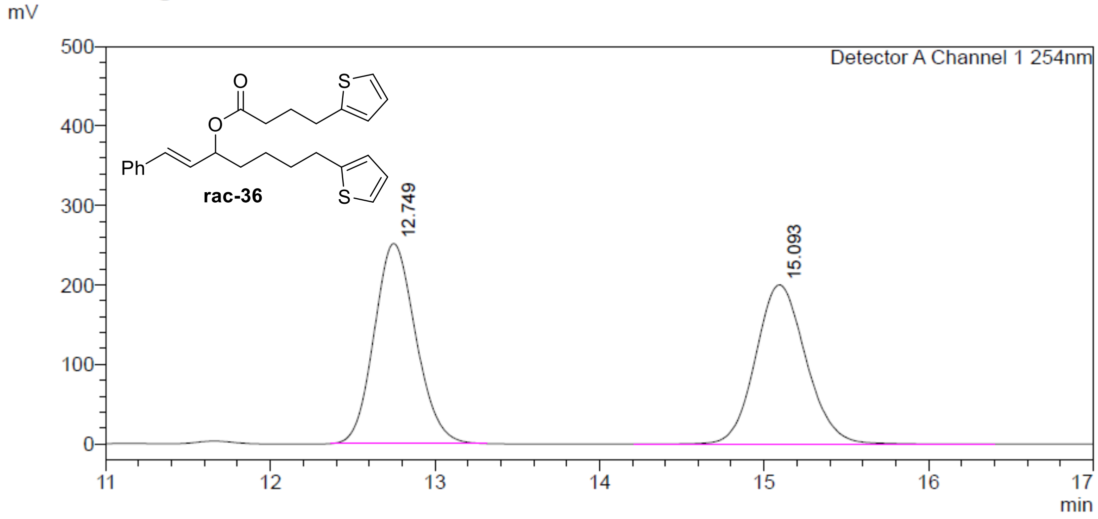

<Peak Table>

Detector A Channel 1 254nm

Peak#	Ret. Time	Height	Height%	Area	Area%
1	12.749	251759	55.707	4430266	50.942
2	15.093	200177	44.293	4266412	49.058
Total		451936	100.000	8696678	100.000

Supplementary Figure 371. Racemic Chromatogram of compound **36**

<Chromatogram>

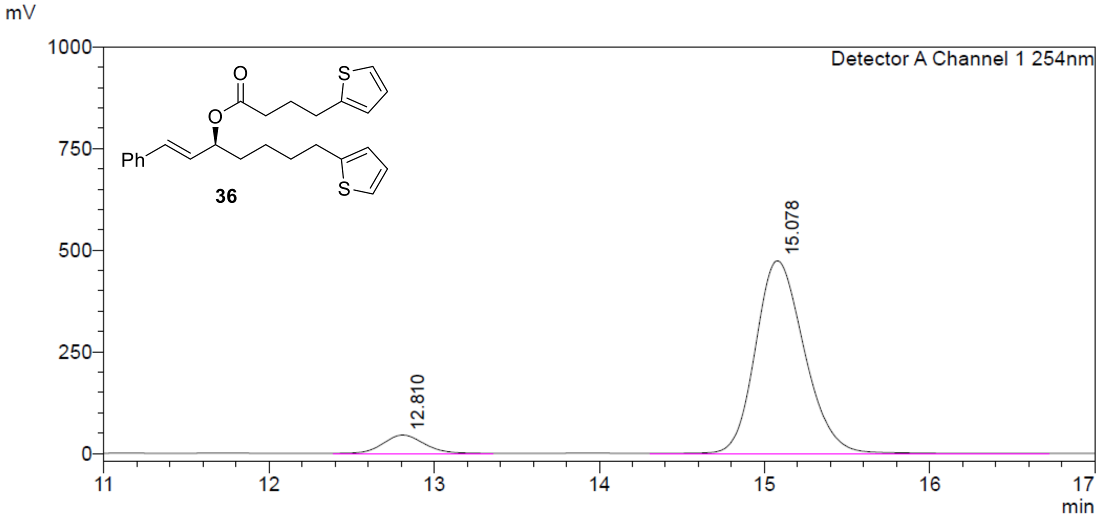

<Peak Table>

Detector A Channel 1 254nm

Peak#	Ret. Time	Height	Height%	Area	Area%
1	12.810	44918	8.658	763552	7.283
2	15.078	473883	91.342	9720080	92.717
Total		518801	100.000	10483632	100.000

Supplementary Figure 372. Scalemic Chromatogram of compound **36**

<Chromatogram>

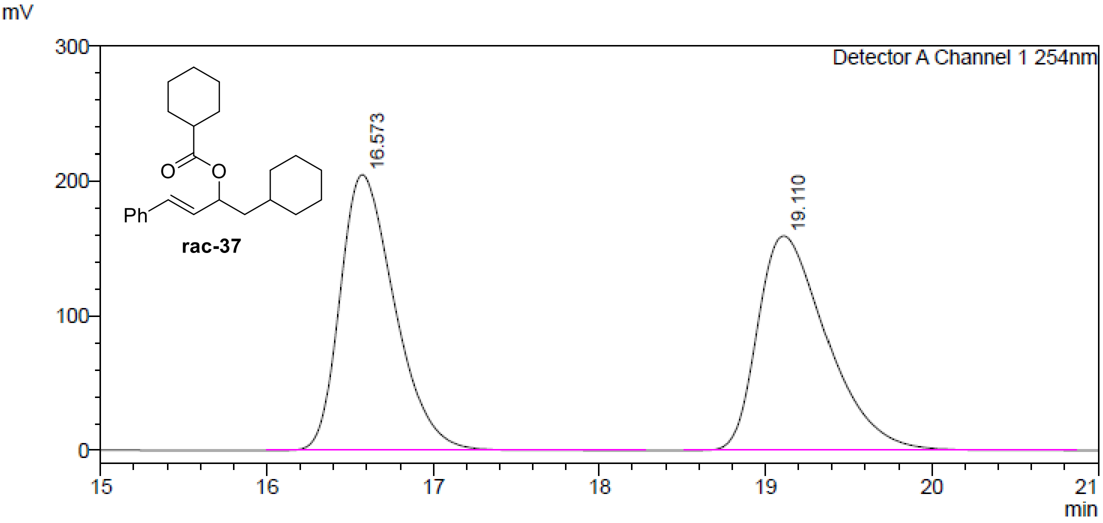

<Peak Table>

Detector A Channel 1 254nm

Peak#	Ret. Time	Height	Height%	Area	Area%
1	16.573	205059	56.253	4696812	50.369
2	19.110	159473	43.747	4628001	49.631
Total		364533	100.000	9324813	100.000

Supplementary Figure 373. Racemic Chromatogram of compound 37

<Chromatogram>

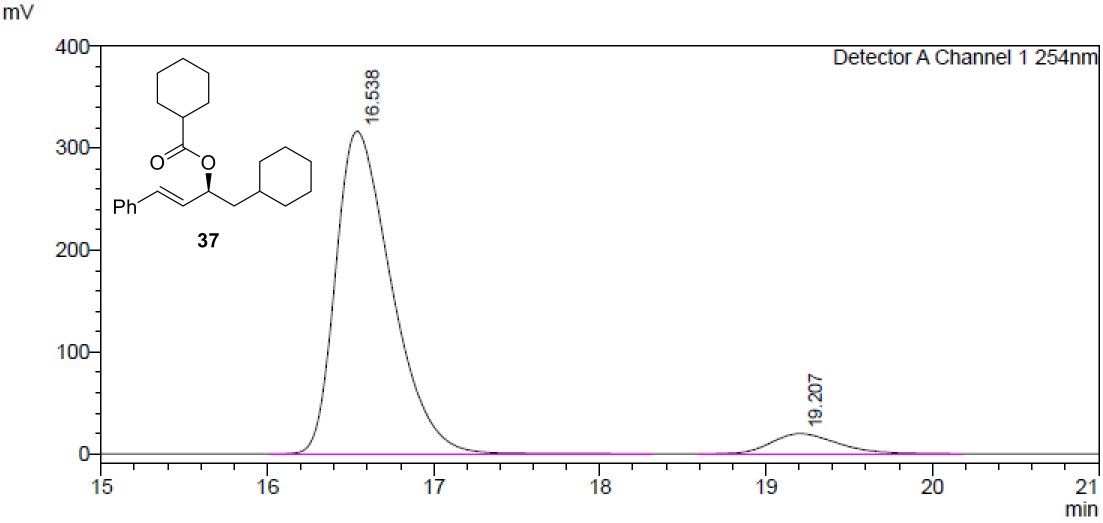

<Peak Table>

Detector A Channel 1 254nm

Peak#	Ret. Time	Height	Height%	Area	Area%
1	16.538	316553	94.138	7393345	93.173
2	19.207	19711	5.862	541745	6.827
Total		336264	100.000	7935090	100.000

Supplementary Figure 374. Scalemic Chromatogram of compound 37

<Chromatogram>

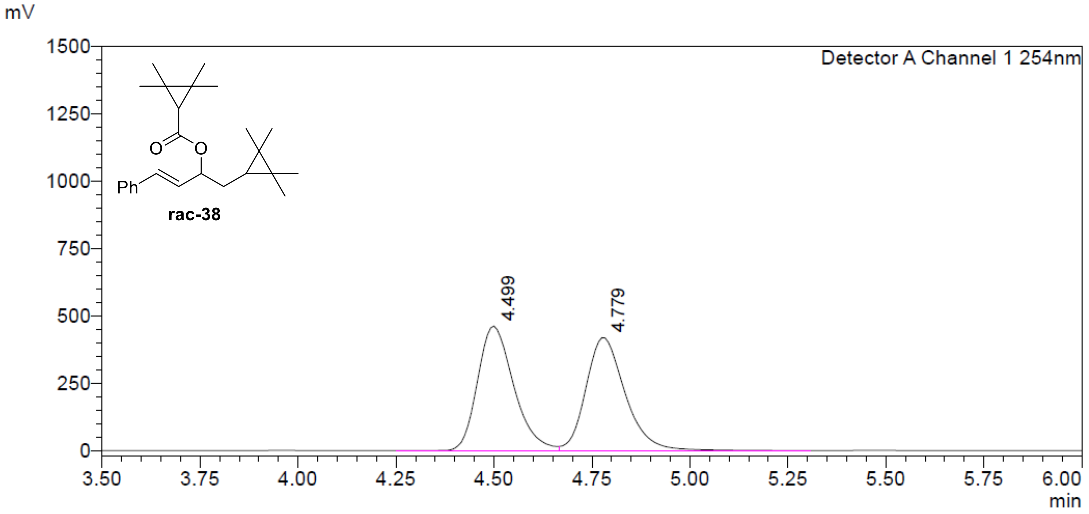

<Peak Table>

Detector A Channel 1 254nm

Peak#	Ret. Time	Height	Height%	Area	Area%
1	4.499	463133	52.457	2988198	50.336
2	4.779	419756	47.543	2948253	49.664
Total		882889	100.000	5936451	100.000

Supplementary Figure 375. Racemic Chromatogram of compound **38**

<Chromatogram>

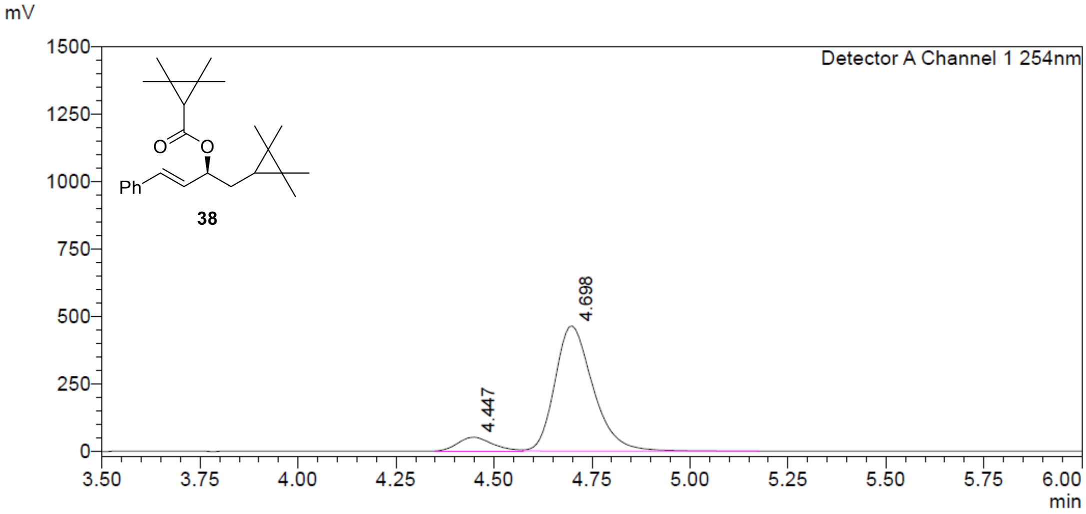

<Peak Table>

Detector A Channel 1 254nm

Peak#	Ret. Time	Height	Height%	Area	Area%
1	4.447	51146	9.934	314762	9.099
2	4.698	463739	90.066	3144423	90.901
Total		514886	100.000	3459185	100.000

Supplementary Figure 376. Scalemic Chromatogram of compound **38**

<Chromatogram>

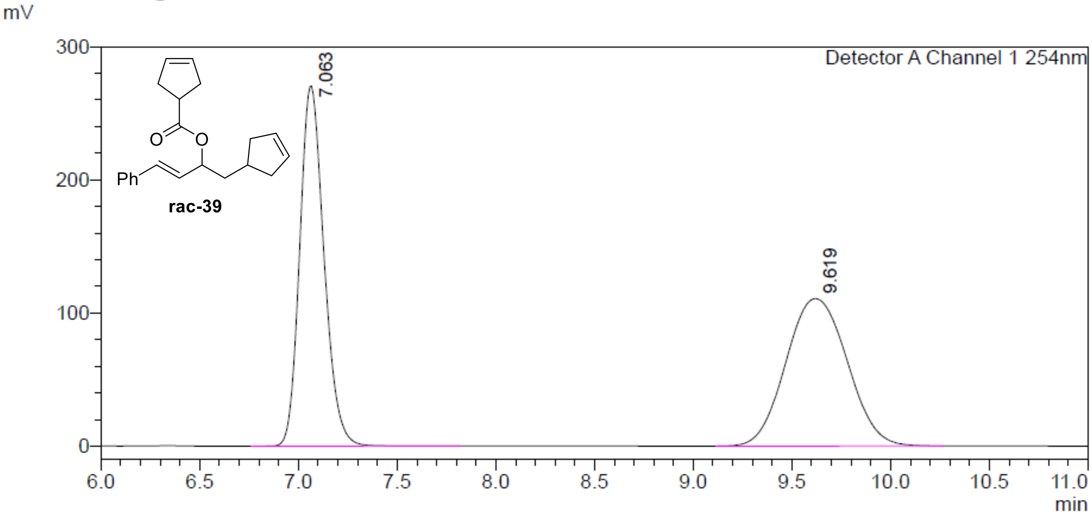

<Peak Table>

Detector A Channel 1 254nm

Peak#	Ret. Time	Height	Height%	Area	Area%
1	7.063	270518	70.948	2314840	49.167
2	9.619	110771	29.052	2393235	50.833
Total		381289	100.000	4708075	100.000

Supplementary Figure 377. Racemic Chromatogram of compound **39**

<Chromatogram>

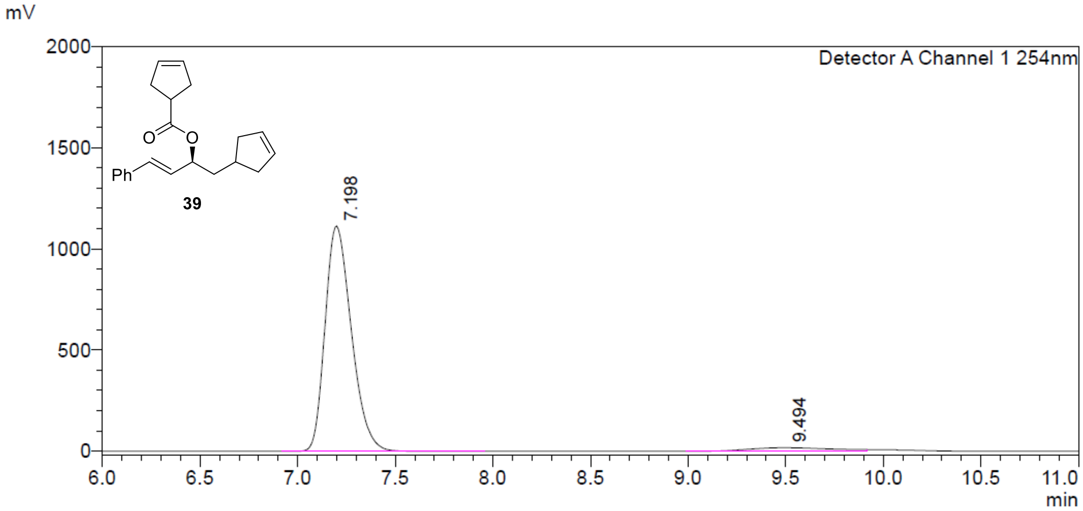

<Peak Table>

Detector A Channel 1 254nm

Peak#	Ret. Time	Height	Height%	Area	Area%
1	7.198	1112559	98.455	10436075	95.152
2	9.494	17461	1.545	531687	4.848
Total		1130021	100.000	10967762	100.000

Supplementary Figure 378. Scalemic Chromatogram of compound **39**

<Chromatogram>

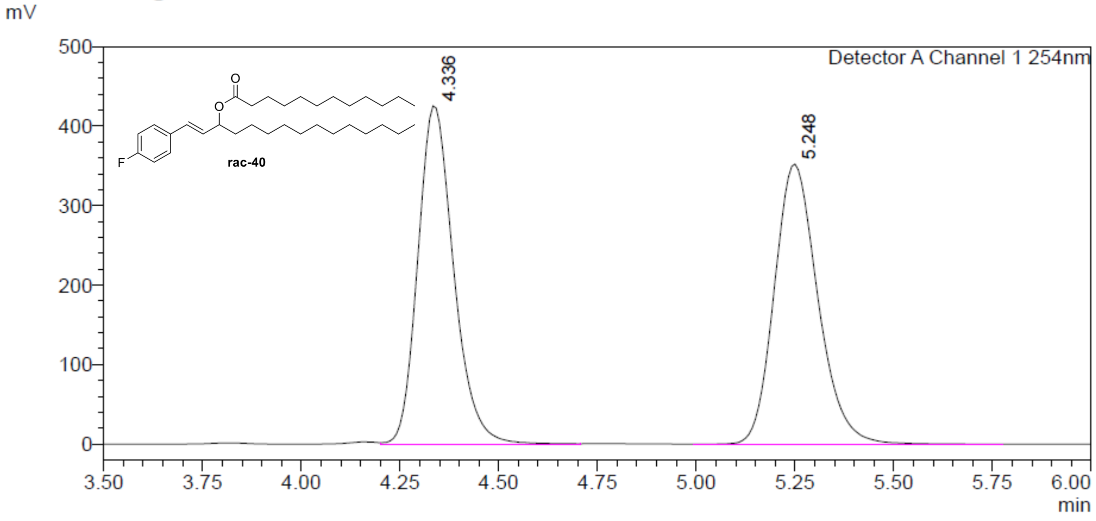

<Peak Table>

Detector A Channel 1 254nm

Peak#	Ret. Time	Height	Height%	Area	Area%
1	4.336	425091	54.676	2688263	49.855
2	5.248	352384	45.324	2703866	50.145
Total		777474	100.000	5392129	100.000

Supplementary Figure 379. Racemic Chromatogram of compound **40**

<Chromatogram>

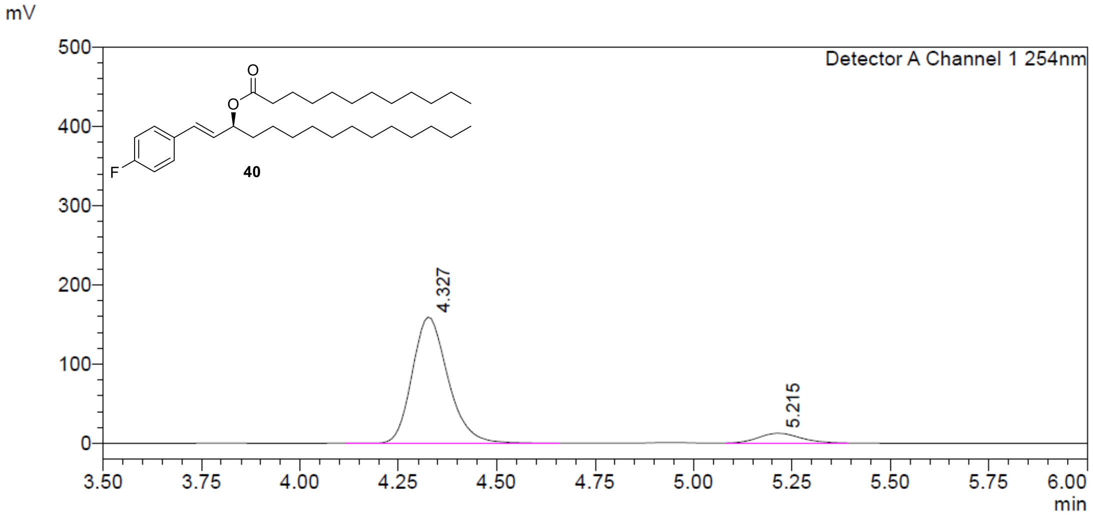

<Peak Table>

Detector A Channel 1 254nm

Peak#	Ret. Time	Height	Height%	Area	Area%
1	4.327	159322	92.831	1013157	91.948
2	5.215	12303	7.169	88719	8.052
Total		171625	100.000	1101876	100.000

Supplementary Figure 380. Scalemic Chromatogram of compound **40**

<Chromatogram>

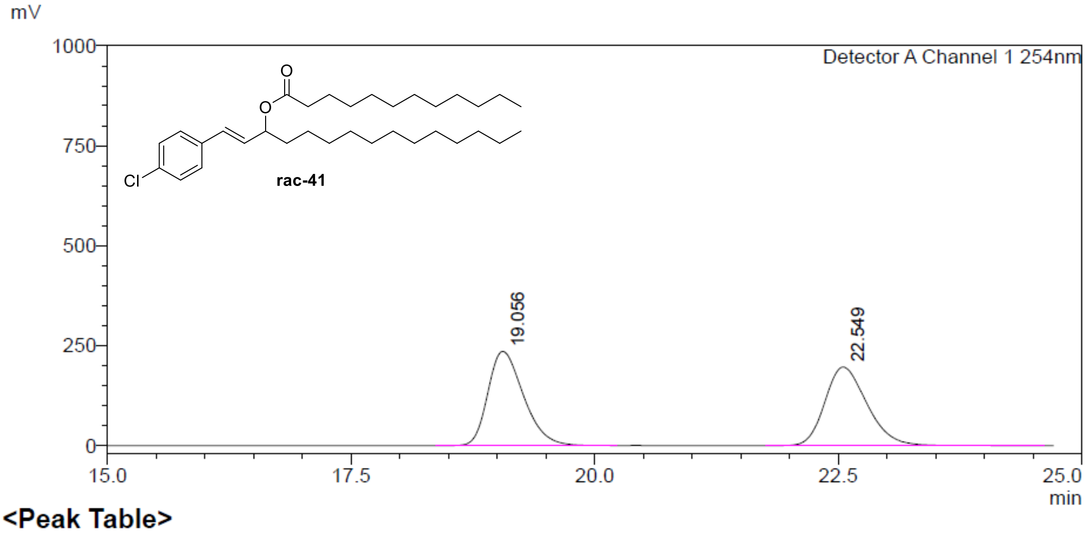

<Peak Table>

Detector A Channel 1 254nm

Peak#	Ret. Time	Height	Height%	Area	Area%
1	19.056	235437	54.544	5973709	50.326
2	22.549	196209	45.456	5896320	49.674
Total		431646	100.000	11870029	100.000

Supplementary Figure 381. Racemic Chromatogram of compound **41**

<Chromatogram>

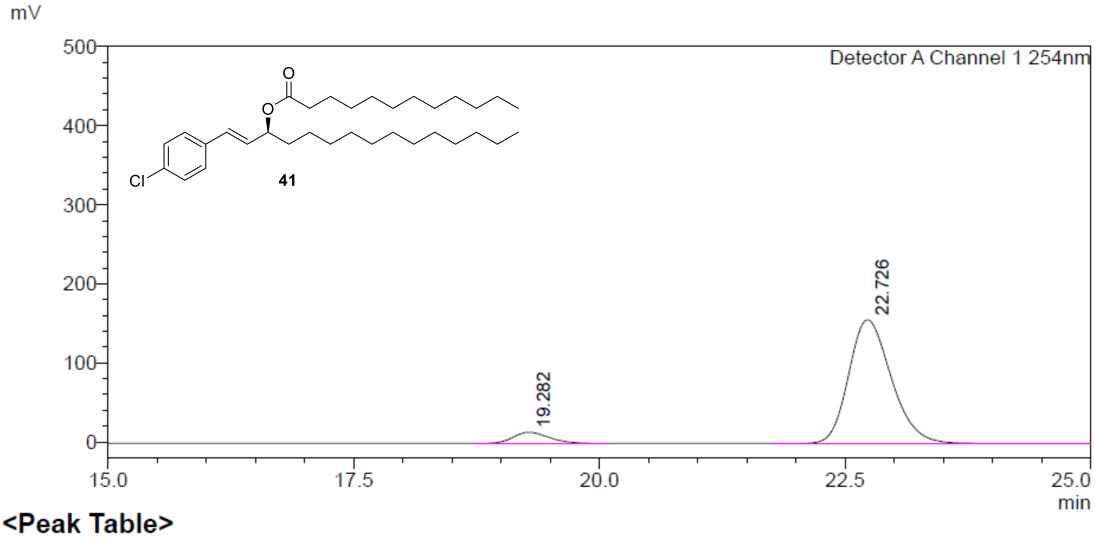

<Peak Table>

Detector A Channel 1 254nm

Peak#	Ret. Time	Height	Height%	Area	Area%
1	19.282	13965	8.207	352276	6.970
2	22.726	156188	91.793	4701645	93.030
Total		170153	100.000	5053921	100.000

Supplementary Figure 382. Scalemic Chromatogram of compound **41**

<Chromatogram>

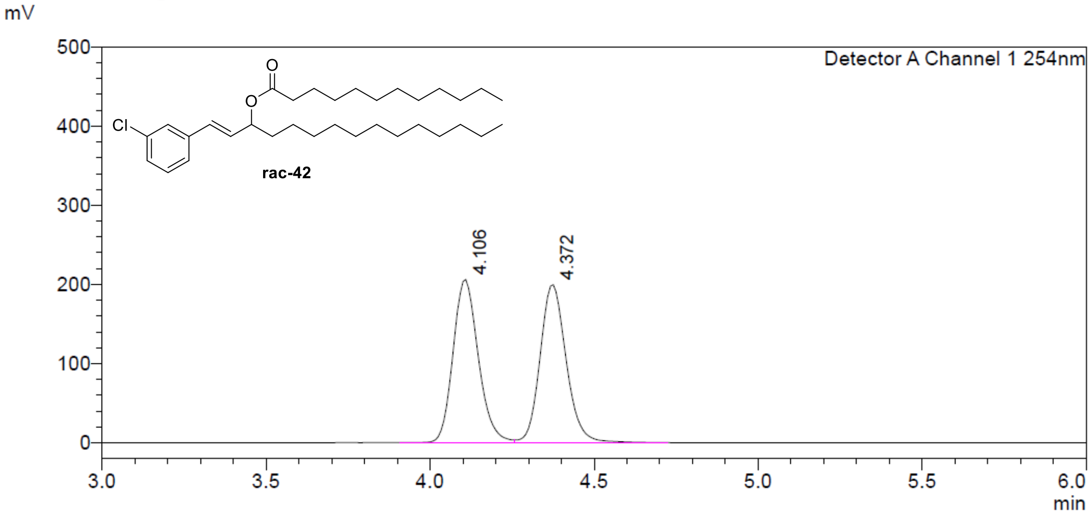

<Peak Table>

Detector A Channel 1 254nm

Peak#	Ret. Time	Height	Height%	Area	Area%
1	4.106	206037	50.794	1105048	49.987
2	4.372	199597	49.206	1105638	50.013
Total		405634	100.000	2210686	100.000

Supplementary Figure 383. Racemic Chromatogram of compound **42**

<Chromatogram>

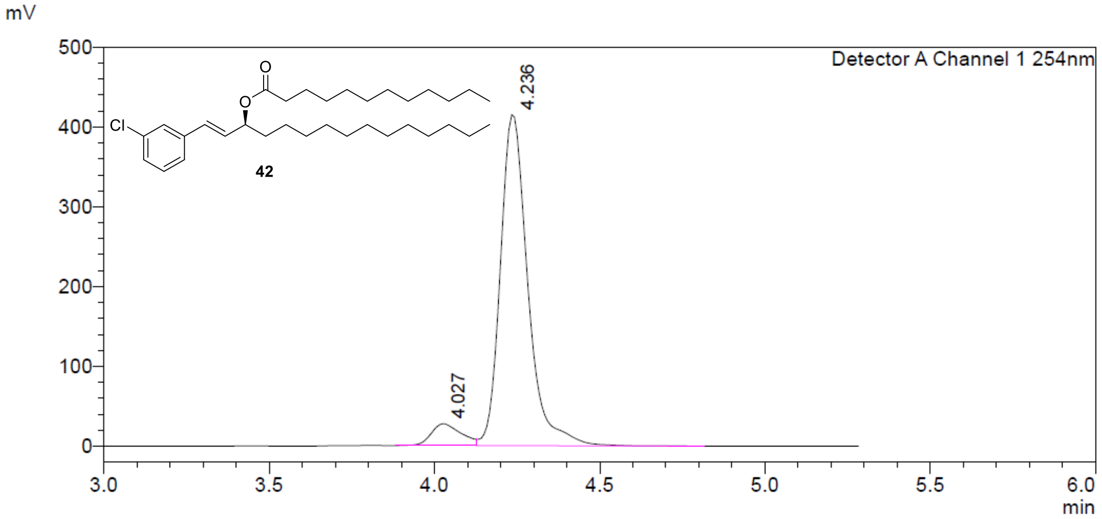

<Peak Table>

Detector A Channel 1 254nm

Peak#	Ret. Time	Height	Height%	Area	Area%
1	4.027	27494	6.214	173950	6.778
2	4.236	414938	93.786	2392582	93.222
Total		442431	100.000	2566532	100.000

Supplementary Figure 384. Scalemic Chromatogram of compound **42**

<Chromatogram>

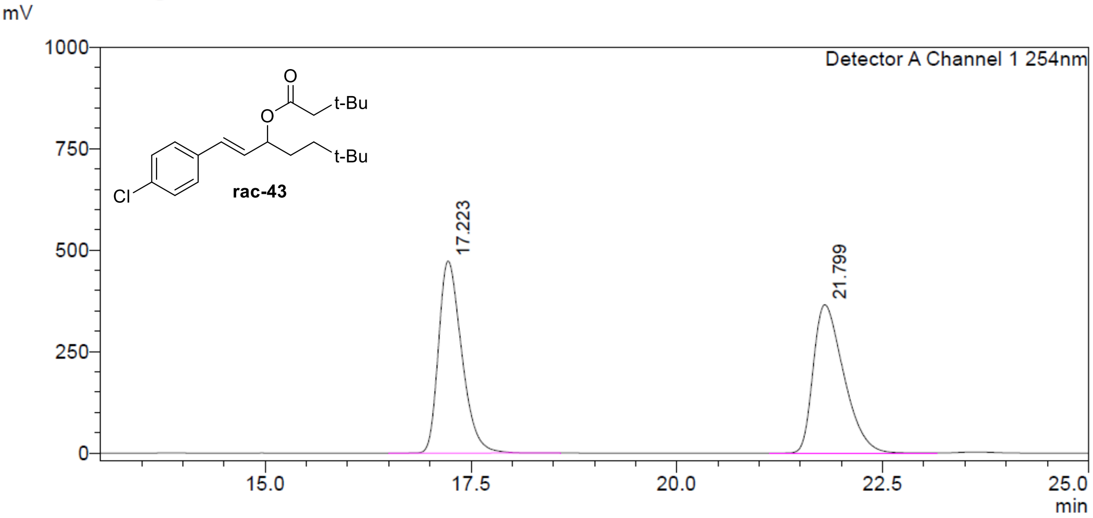

<Peak Table>

Detector A Channel 1 254nm

Peak#	Ret. Time	Height	Height%	Area	Area%
1	17.223	473625	56.411	9302850	49.575
2	21.799	365970	43.589	9462165	50.425
Total		839594	100.000	18765015	100.000

Supplementary Figure 385. Racemic Chromatogram of compound **43**

<Chromatogram>

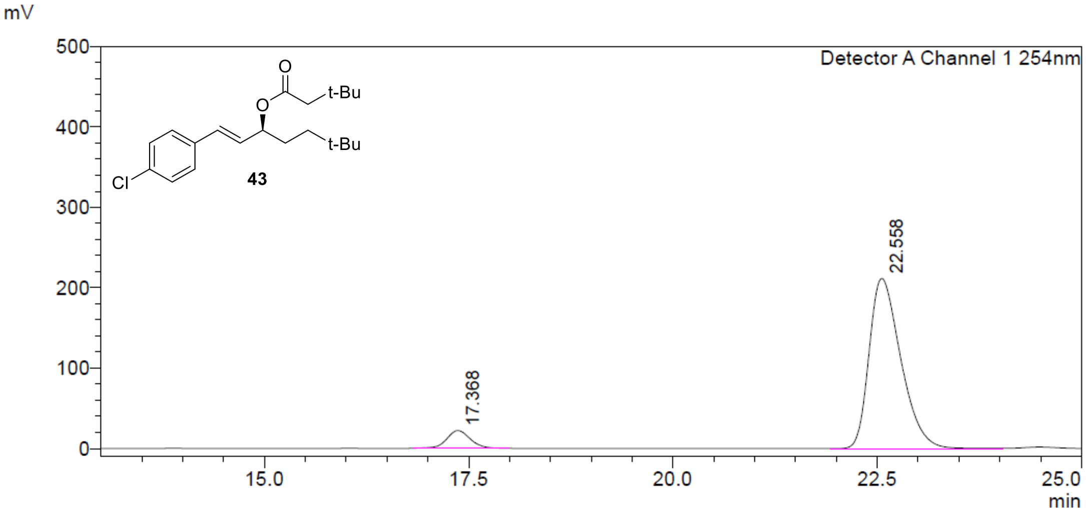

<Peak Table>

Detector A Channel 1 254nm

Peak#	Ret. Time	Height	Height%	Area	Area%
1	17.368	21989	9.412	443712	7.205
2	22.558	211633	90.588	5715048	92.795
Total		233621	100.000	6158760	100.000

Supplementary Figure 386. Scalemic Chromatogram of compound **43**

<Chromatogram>

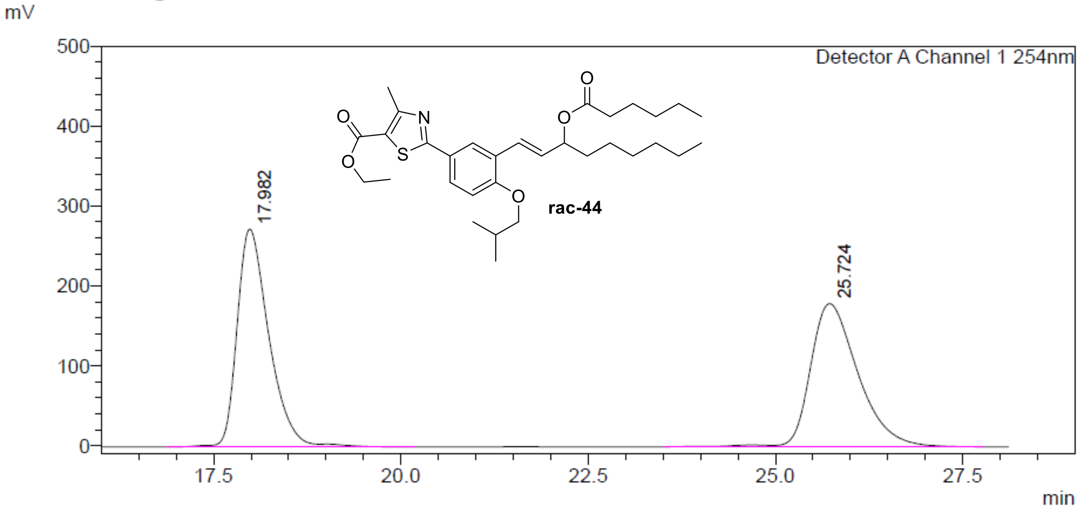

<Peak Table>

Detector A Channel 1 254nm

Peak#	Ret. Time	Height	Height%	Area	Area%
1	17.982	272584	60.340	8079920	50.407
2	25.724	179163	39.660	7949441	49.593
Total		451748	100.000	16029361	100.000

Supplementary Figure 387. Racemic Chromatogram of compound **44**

<Chromatogram>

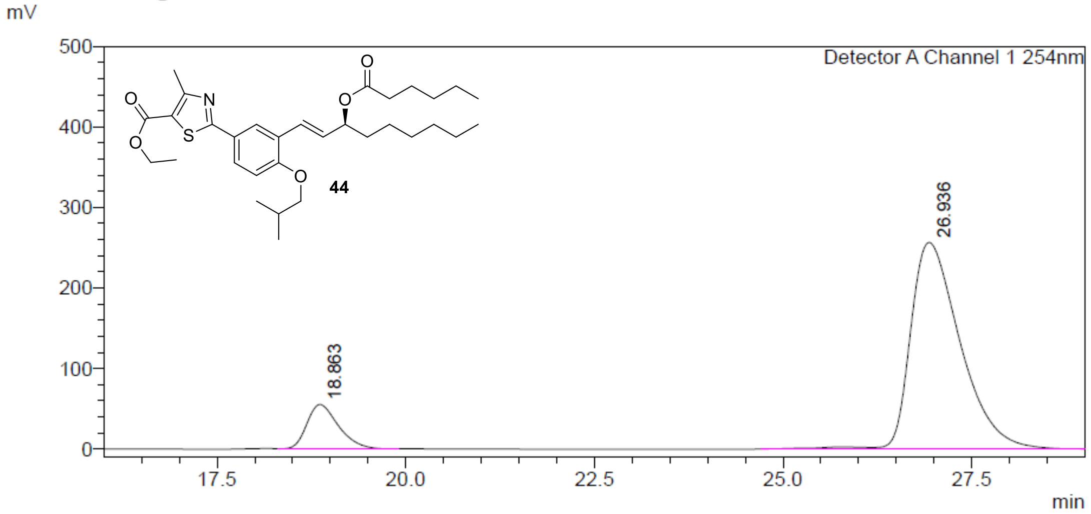

<Peak Table>

Detector A Channel 1 254nm

Peak#	Ret. Time	Height	Height%	Area	Area%
1	18.863	54771	17.582	1575784	11.773
2	26.936	256743	82.418	11808912	88.227
Total		311514	100.000	13384697	100.000

Supplementary Figure 388. Scalemic Chromatogram of compound **44**

<Chromatogram>

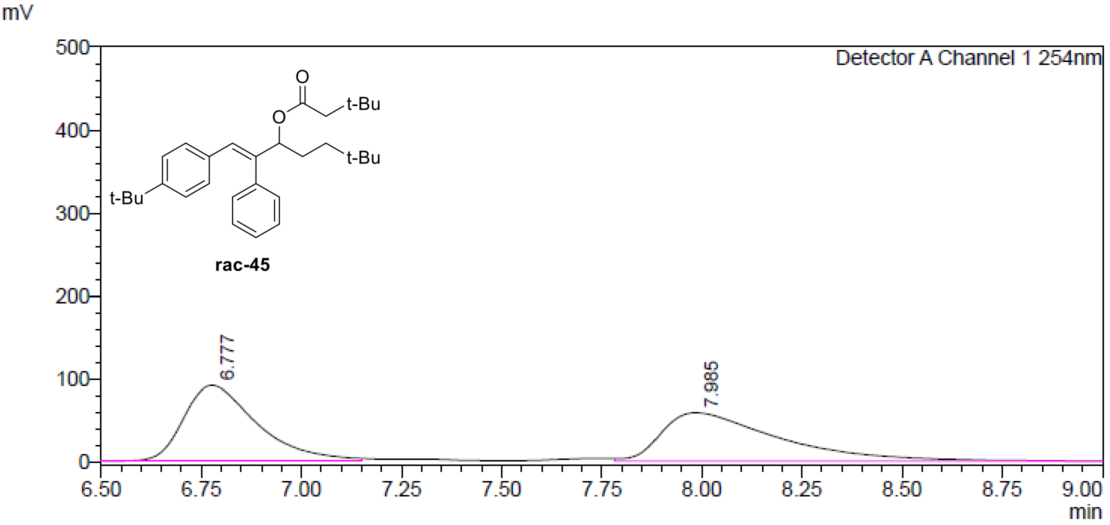

<Peak Table>

Detector A Channel 1 254nm

Peak#	Ret. Time	Height	Height%	Area	Area%
1	6.777	91086	60.924	1158327	49.289
2	7.985	58421	39.076	1191731	50.711
Total		149507	100.000	2350059	100.000

Supplementary Figure 389. Racemic Chromatogram of compound **45**

<Chromatogram>

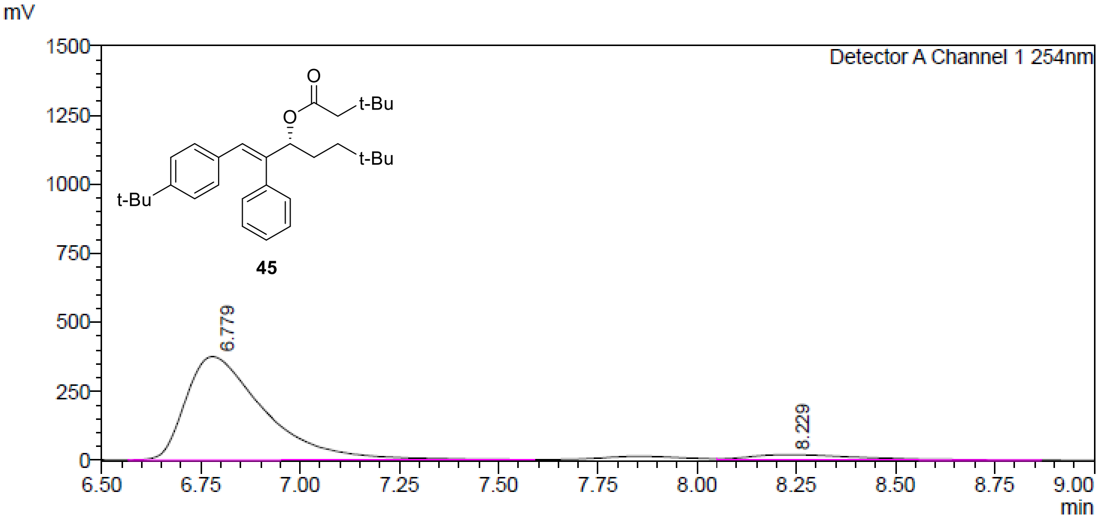

<Peak Table>

Detector A Channel 1 254nm

Peak#	Ret. Time	Height	Height%	Area	Area%
1	6.779	374381	95.217	5123537	93.519
2	8.229	18807	4.783	355095	6.481
Total		393188	100.000	5478632	100.000

Supplementary Figure 390. Scalemic Chromatogram of compound **45**

<Chromatogram>

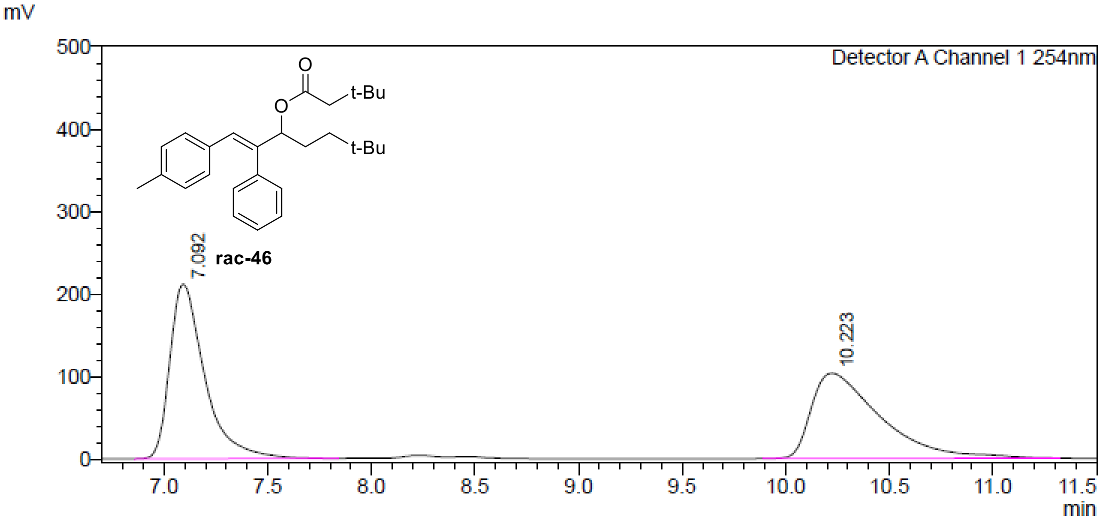

<Peak Table>

Detector A Channel 1 254nm

Peak#	Ret. Time	Height	Height%	Area	Area%
1	7.092	211301	67.156	2474653	50.884
2	10.223	103343	32.844	2388683	49.116
Total		314644	100.000	4863335	100.000

Supplementary Figure 391. Racemic Chromatogram of compound **46**

<Chromatogram>

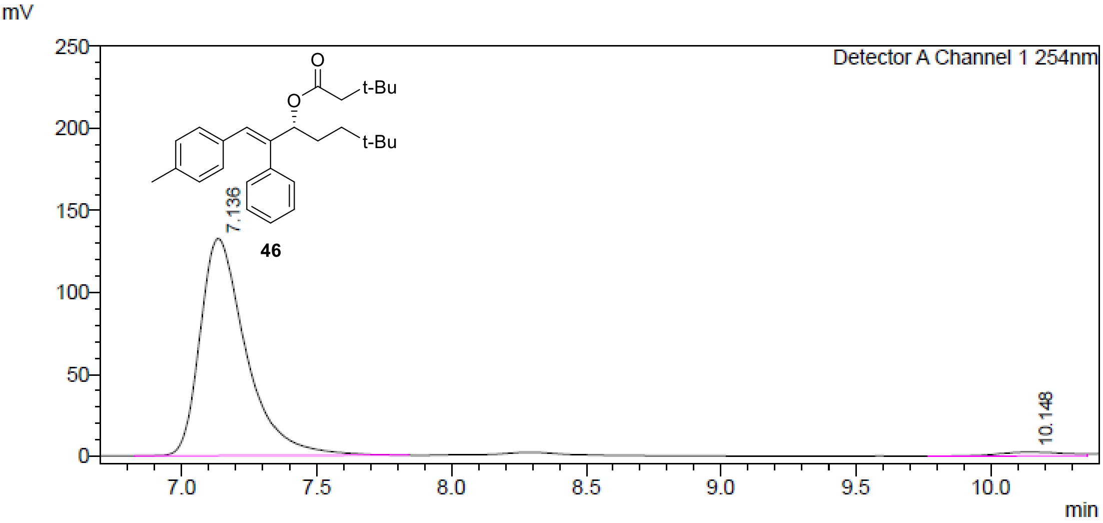

<Peak Table>

Detector A Channel 1 254nm

Peak#	Ret. Time	Height	Height%	Area	Area%
1	7.136	132662	98.180	1577113	97.404
2	10.148	2459	1.820	42038	2.596
Total		135121	100.000	1619151	100.000

Supplementary Figure 392. Scalemic Chromatogram of compound **46**

<Chromatogram>

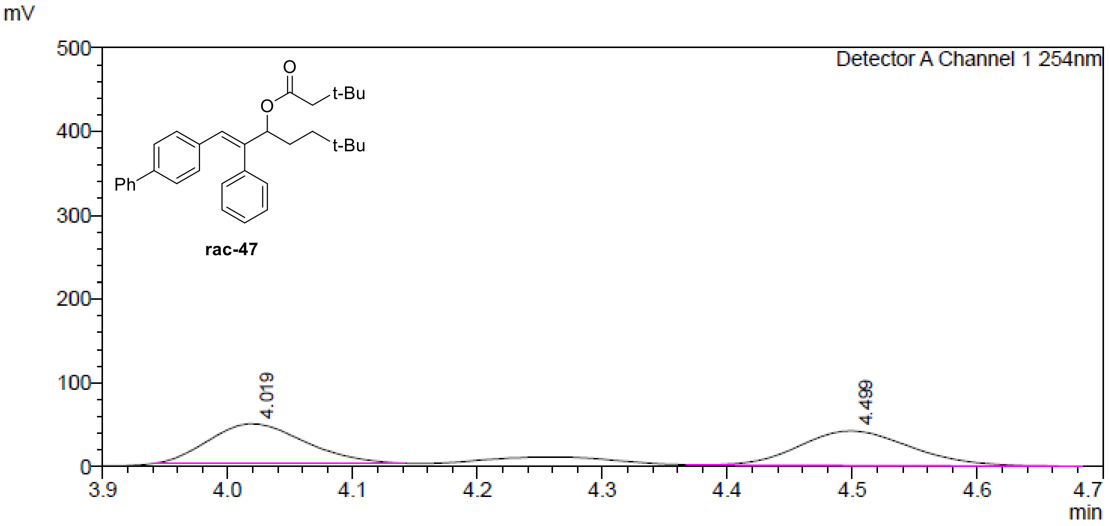

<Peak Table>

Detector A Channel 1 254nm

Peak#	Ret. Time	Height	Height%	Area	Area%
1	4.019	47547	53.519	253295	50.141
2	4.499	41295	46.481	251871	49.859
Total		88841	100.000	505166	100.000

Supplementary Figure 393. Racemic Chromatogram of compound **47**

<Chromatogram>

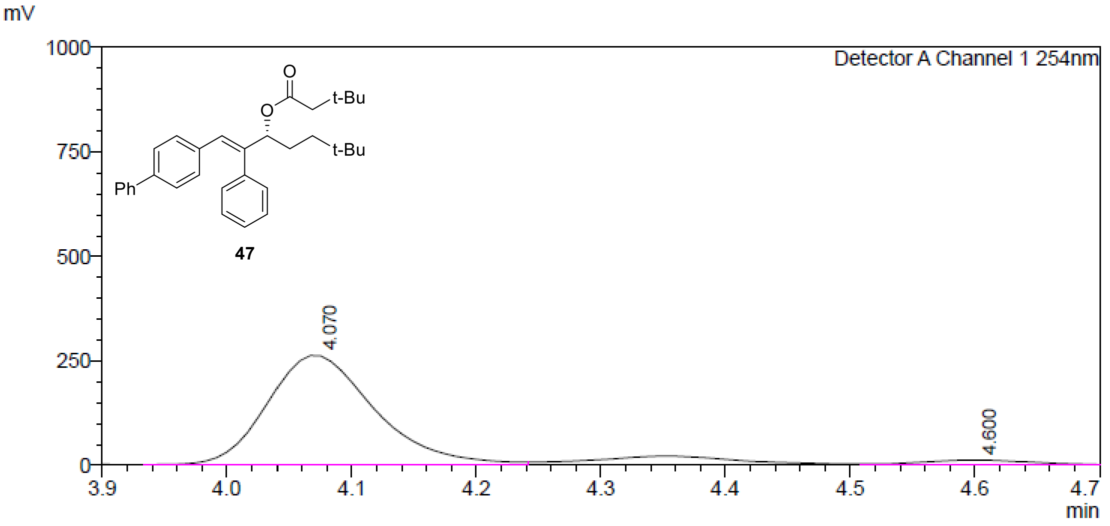

<Peak Table>

Detector A Channel 1 254nm

Peak#	Ret. Time	Height	Height%	Area	Area%
1	4.070	261175	95.899	1560088	95.781
2	4.600	11170	4.101	68715	4.219
Total		272344	100.000	1628804	100.000

Supplementary Figure 394. Scalemic Chromatogram of compound **47**

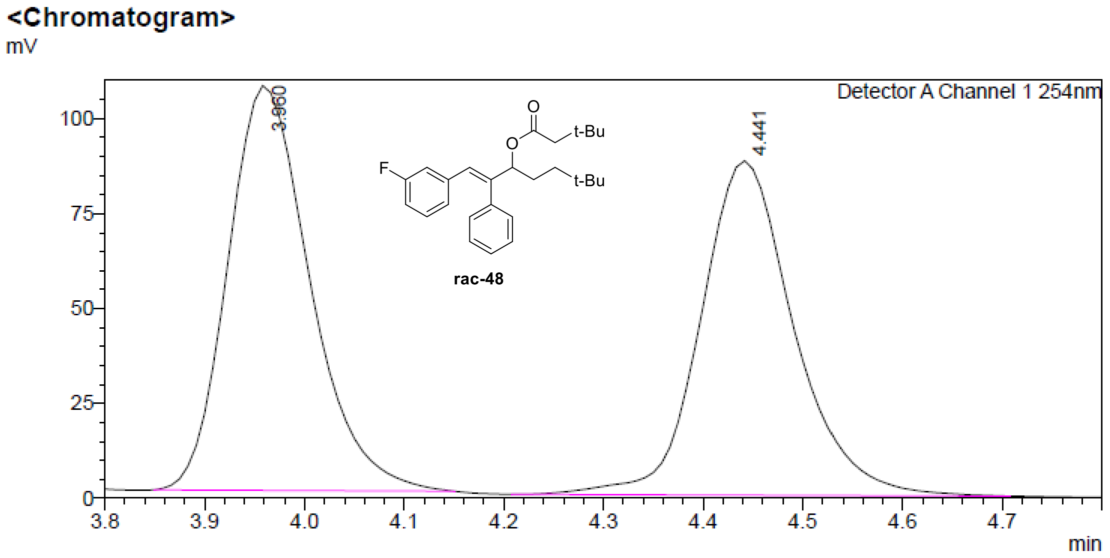

<Peak Table>

Detector A Channel 1 254nm

Peak#	Ret. Time	Height	Height%	Area	Area%
1	3.960	106540	54.750	607327	51.465
2	4.441	88054	45.250	572745	48.535
Total		194594	100.000	1180072	100.000

Supplementary Figure 395. Racemic Chromatogram of compound **48**

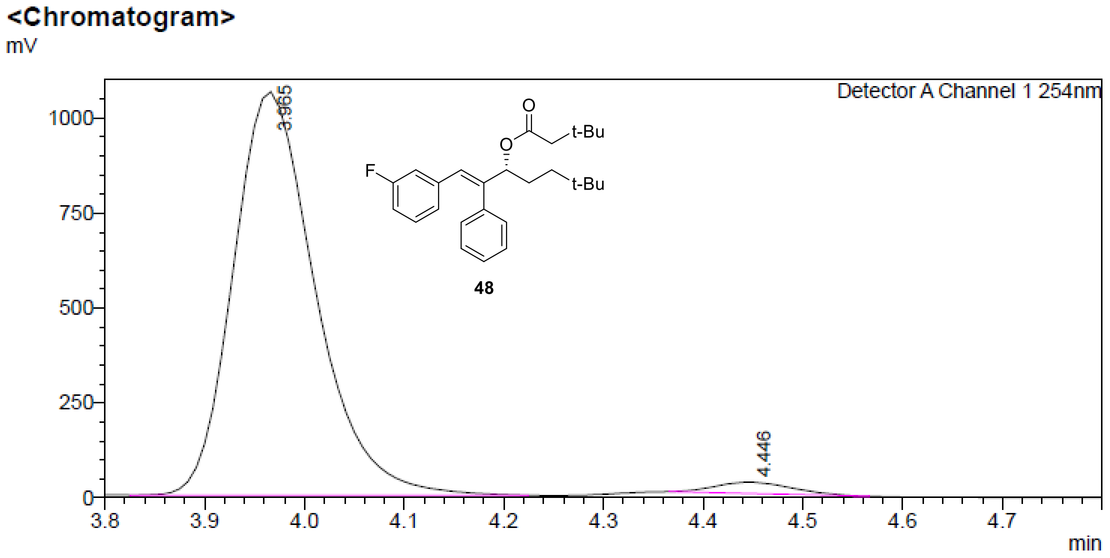

<Peak Table>

Detector A Channel 1 254nm

Peak#	Ret. Time	Height	Height%	Area	Area%
1	3.965	1062022	97.294	6059161	97.483
2	4.446	29540	2.706	156450	2.517
Total		1091562	100.000	6215610	100.000

Supplementary Figure 396. Scalemic Chromatogram of compound **48**

<Chromatogram>

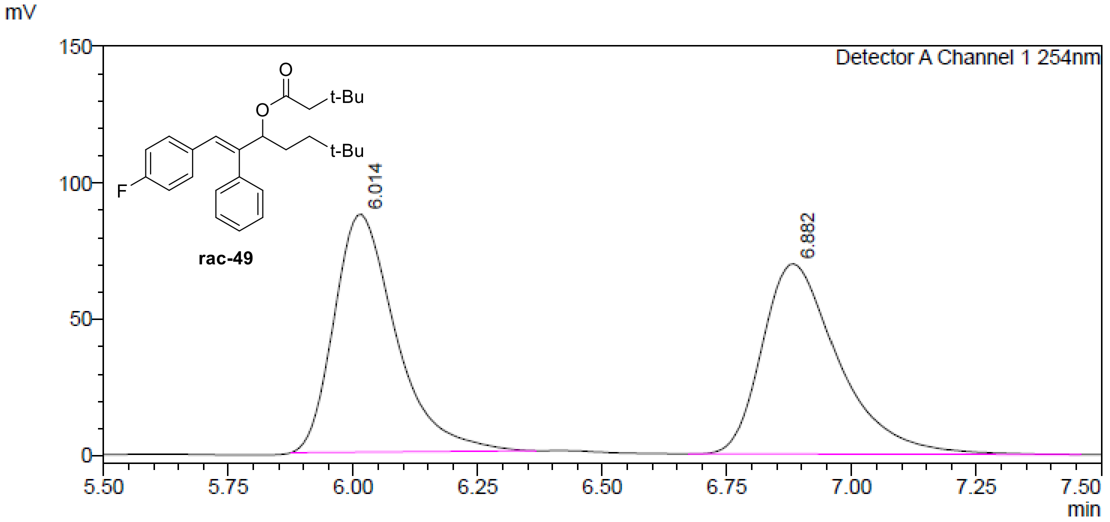

<Peak Table>

Detector A Channel 1 254nm

Peak#	Ret. Time	Height	Height%	Area	Area%
1	6.014	87250	55.554	755973	50.357
2	6.882	69804	44.446	745244	49.643
Total		157053	100.000	1501217	100.000

Supplementary Figure 397. Racemic Chromatogram of compound **49**

<Chromatogram>

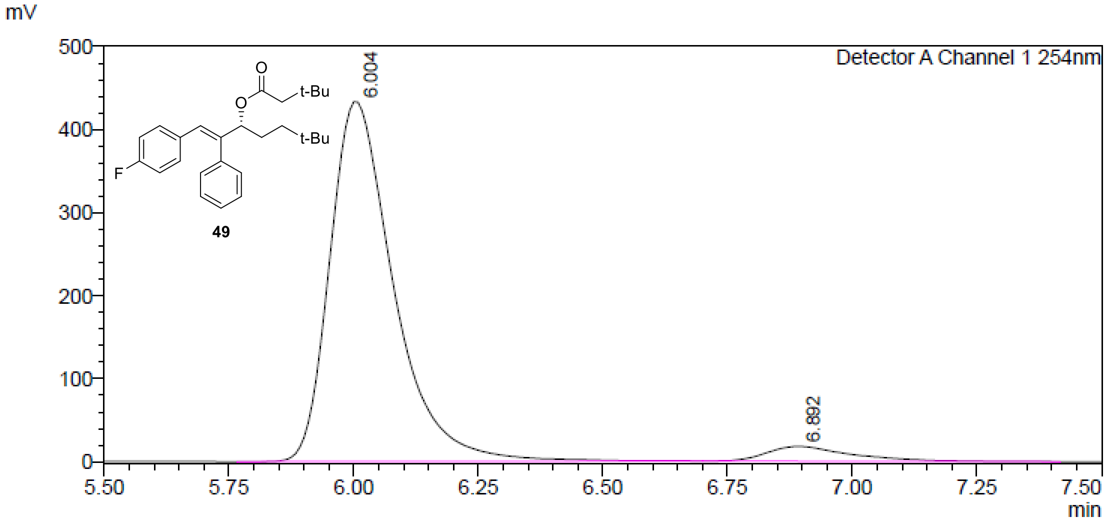

<Peak Table>

Detector A Channel 1 254nm

Peak#	Ret. Time	Height	Height%	Area	Area%
1	6.004	433115	96.055	3859461	94.956
2	6.892	17786	3.945	205008	5.044
Total		450901	100.000	4064470	100.000

Supplementary Figure 398. Scalemic Chromatogram of compound **49**

<Chromatogram>

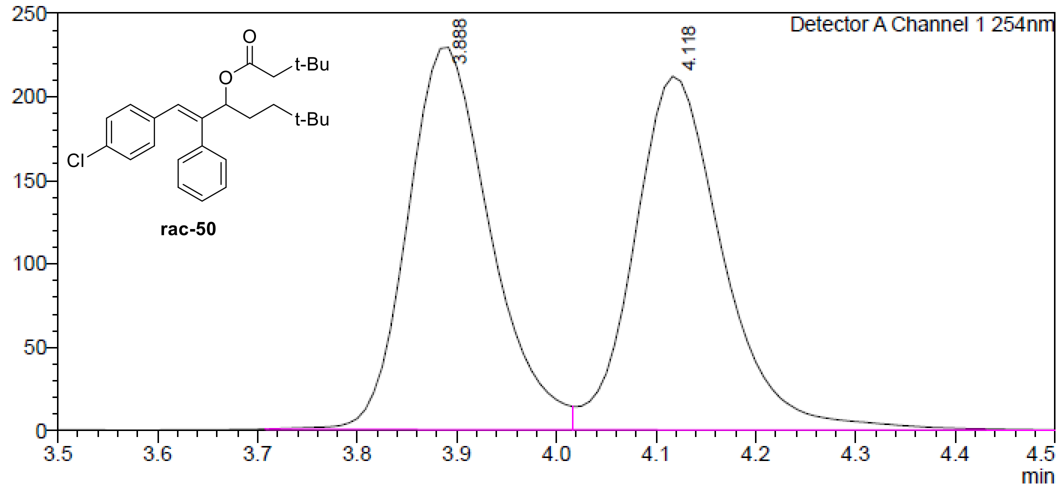

<Peak Table>

Detector A Channel 1 254nm

Peak#	Ret. Time	Height	Height%	Area	Area%
1	3.888	228653	51.932	1323057	50.278
2	4.118	211643	48.068	1308422	49.722
Total		440296	100.000	2631478	100.000

Supplementary Figure 399. Racemic Chromatogram of compound **50**

<Chromatogram>

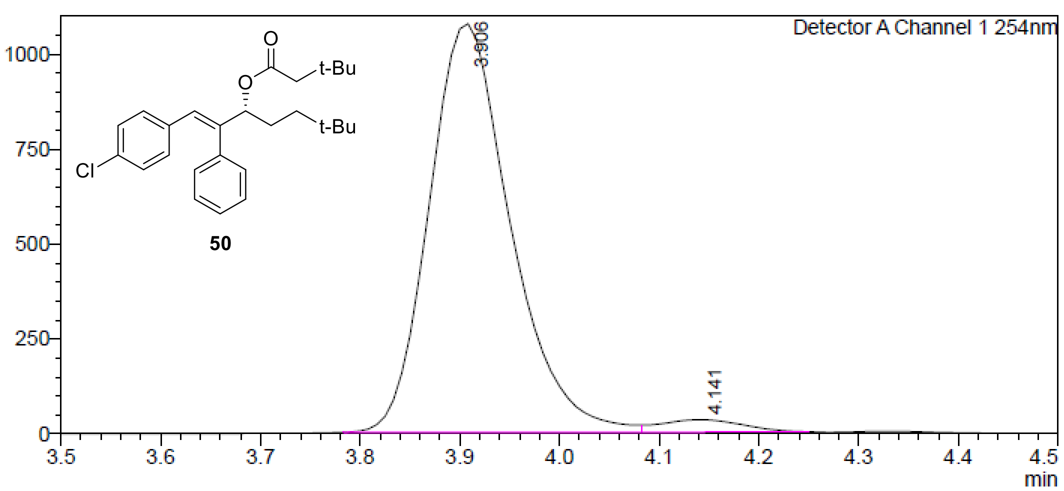

<Peak Table>

Detector A Channel 1 254nm

Peak#	Ret. Time	Height	Height%	Area	Area%
1	3.906	1076733	97.055	6111906	97.119
2	4.141	32669	2.945	181306	2.881
Total		1109402	100.000	6293212	100.000

Supplementary Figure 400. Scalemic Chromatogram of compound **50**

<Chromatogram>

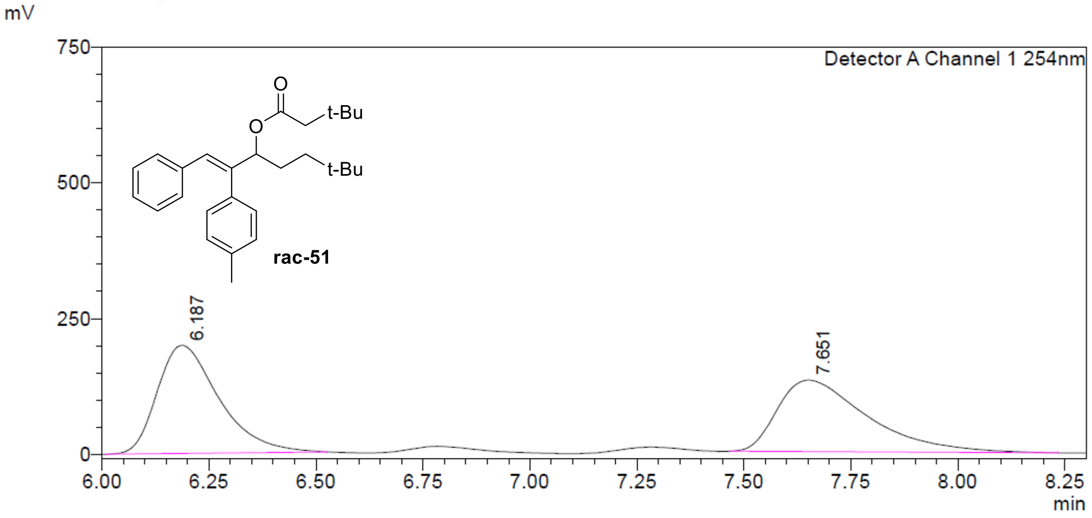

<Peak Table>

Detector A Channel 1 254nm

Peak#	Ret. Time	Height	Height%	Area	Area%
1	6.187	198776	60.124	1996035	50.841
2	7.651	131834	39.876	1929965	49.159
Total		330609	100.000	3926000	100.000

Supplementary Figure 401. Racemic Chromatogram of compound **51**

<Chromatogram>

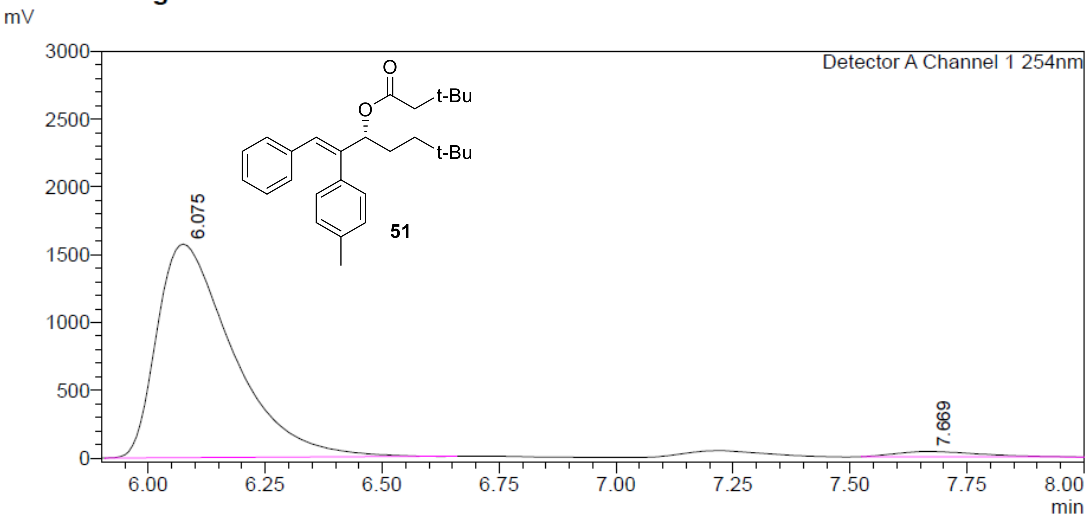

<Peak Table>

Detector A Channel 1 254nm

Peak#	Ret. Time	Height	Height%	Area	Area%
1	6.075	1573948	97.619	17749481	97.313
2	7.669	38385	2.381	490090	2.687
Total		1612332	100.000	18239571	100.000

Supplementary Figure 402. Scalemic Chromatogram of compound **51**

<Chromatogram>

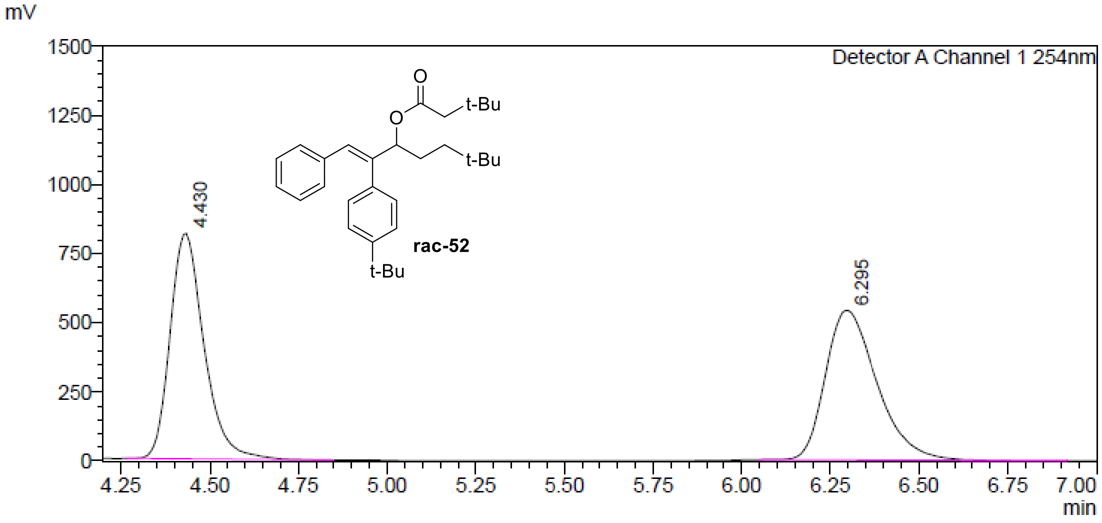

<Peak Table>

Detector A Channel 1 254nm

Peak#	Ret. Time	Height	Height%	Area	Area%
1	4.430	817329	60.173	5427866	49.762
2	6.295	540980	39.827	5479685	50.238
Total		1358309	100.000	10907551	100.000

Supplementary Figure 403. Racemic Chromatogram of compound **52**

<Chromatogram>

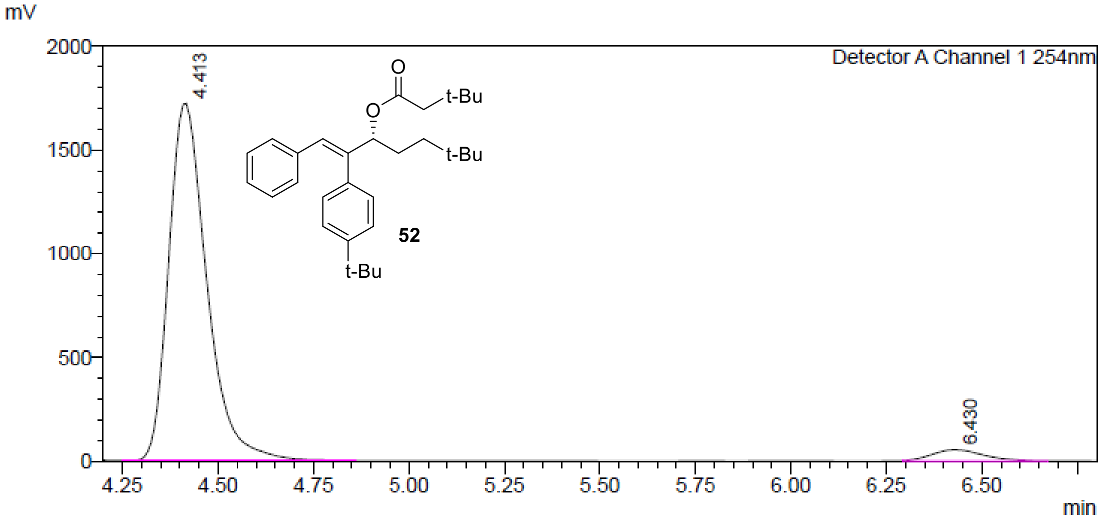

<Peak Table>

Detector A Channel 1 254nm

Peak#	Ret. Time	Height	Height%	Area	Area%
1	4.413	1720829	97.001	11934043	96.082
2	6.430	53207	2.999	486692	3.918
Total		1774036	100.000	12420734	100.000

Supplementary Figure 404. Scalemic Chromatogram of compound **52**

<Chromatogram>

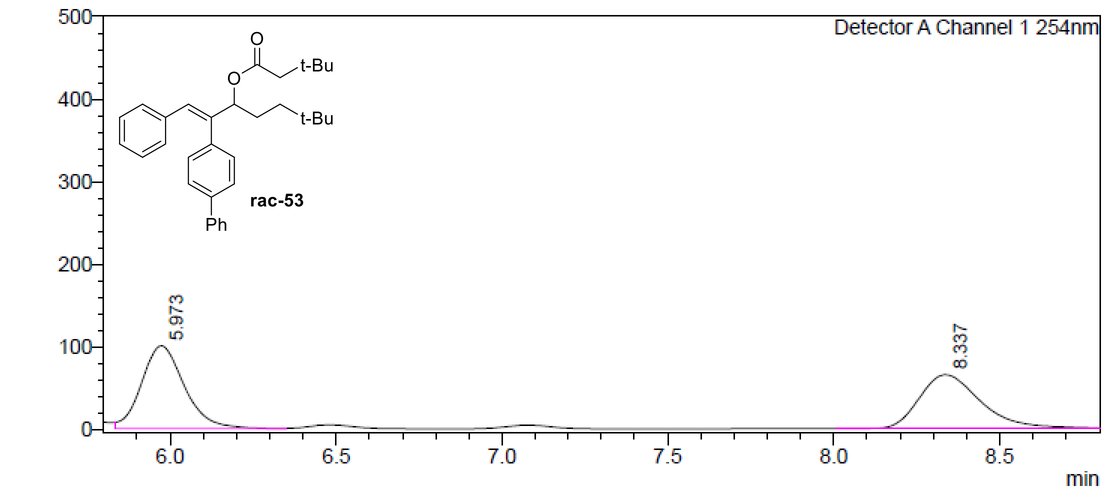

<Peak Table>

Detector A Channel 1 254nm

Peak#	Ret. Time	Height	Height%	Area	Area%
1	5.973	100407	60.543	899634	50.445
2	8.337	65438	39.457	883774	49.555
Total		165844	100.000	1783408	100.000

Supplementary Figure 405. Racemic Chromatogram of compound **53**

<Chromatogram>

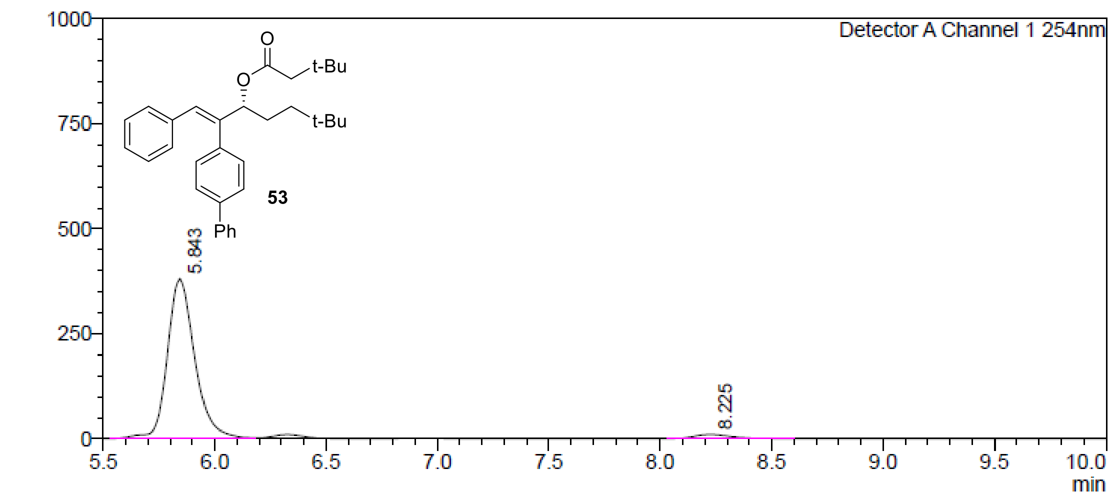

<Peak Table>

Detector A Channel 1 254nm

Peak#	Ret. Time	Height	Height%	Area	Area%
1	5.843	379618	97.475	3268139	96.623
2	8.225	9832	2.525	114222	3.377
Total		389450	100.000	3382362	100.000

Supplementary Figure 406. Scalemic Chromatogram of compound **53**

<Chromatogram>

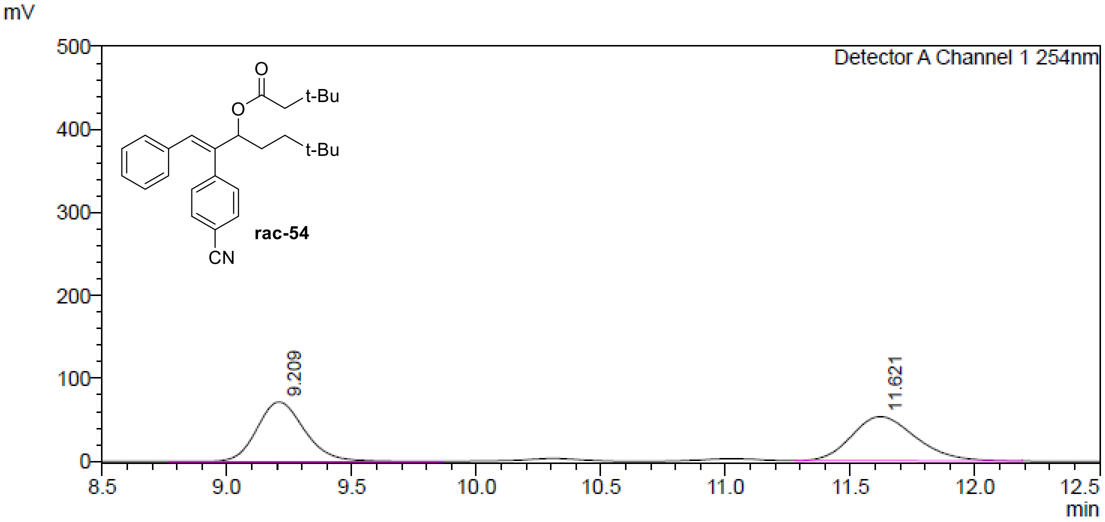

<Peak Table>

Detector A Channel 1 254nm

Peak#	Ret. Time	Height	Height%	Area	Area%
1	9.209	71566	57.289	941945	49.551
2	11.621	53354	42.711	959033	50.449
Total		124920	100.000	1900978	100.000

Supplementary Figure 407. Racemic Chromatogram of compound **54**

<Chromatogram>

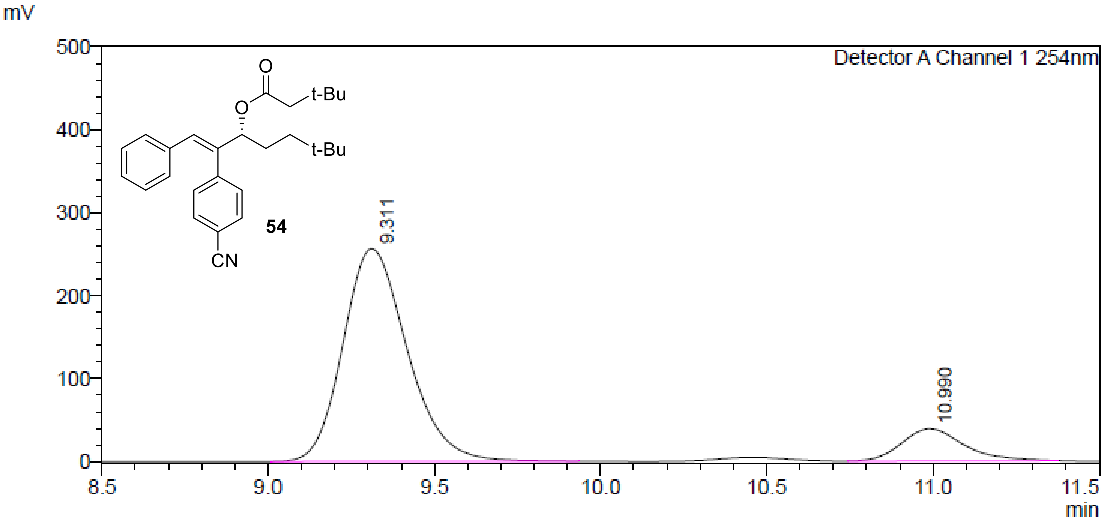

<Peak Table>

Detector A Channel 1 254nm

Peak#	Ret. Time	Height	Height%	Area	Area%
1	9.311	256516	86.861	3446975	87.225
2	10.990	38801	13.139	504824	12.775
Total		295317	100.000	3951800	100.000

Supplementary Figure 408. Scalemic Chromatogram of compound **54**

<Chromatogram>

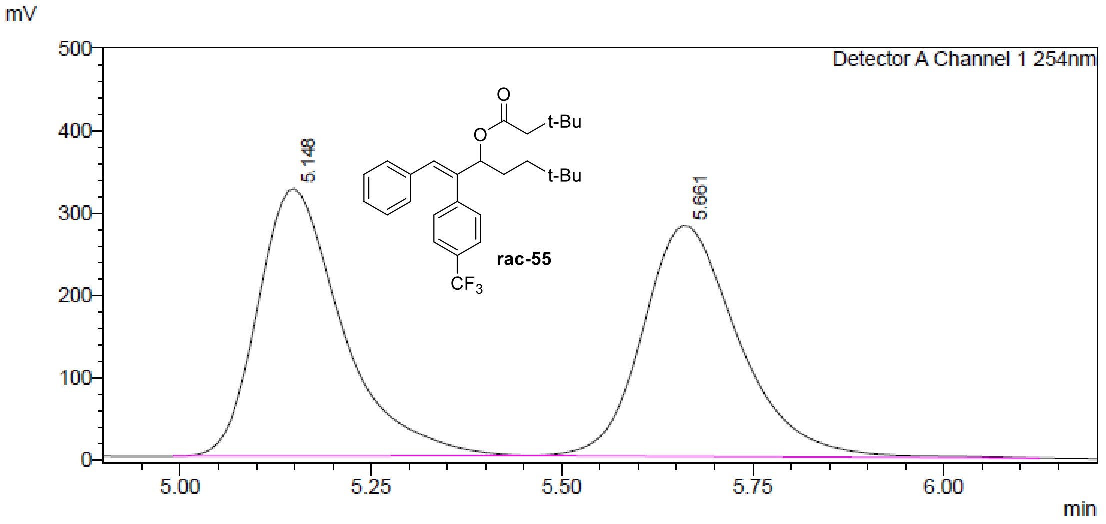

<Peak Table>

Detector A Channel 1 254nm

Peak#	Ret. Time	Height	Height%	Area	Area%
1	5.148	325029	53.668	2502329	50.421
2	5.661	280597	46.332	2460510	49.579
Total		605626	100.000	4962840	100.000

Supplementary Figure 409. Racemic Chromatogram of compound **55**

<Chromatogram>

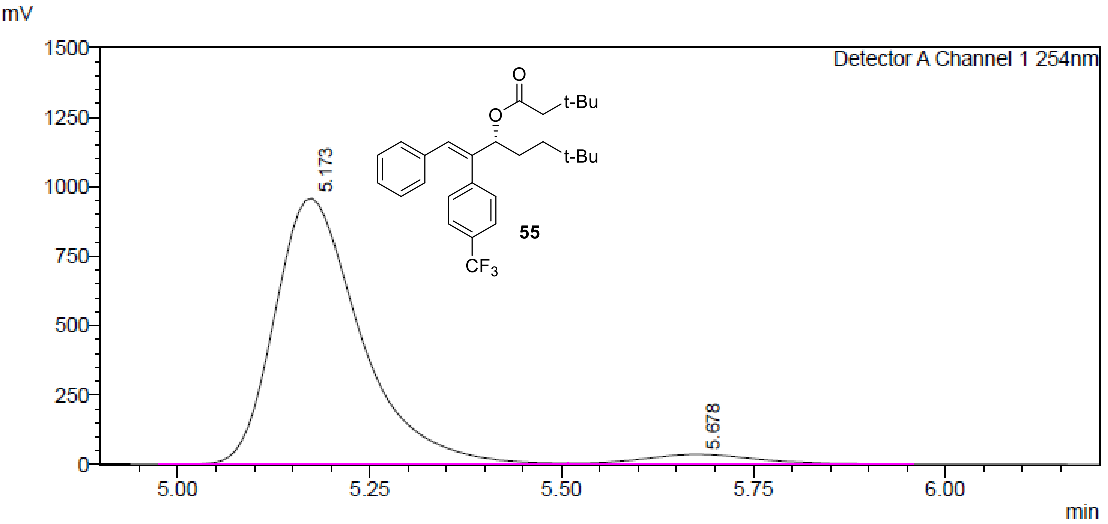

<Peak Table>

Detector A Channel 1 254nm

Peak#	Ret. Time	Height	Height%	Area	Area%
1	5.173	956918	96.390	7442737	95.236
2	5.678	35843	3.610	372308	4.764
Total		992761	100.000	7815045	100.000

Supplementary Figure 410. Scalemic Chromatogram of compound **55**

<Chromatogram>

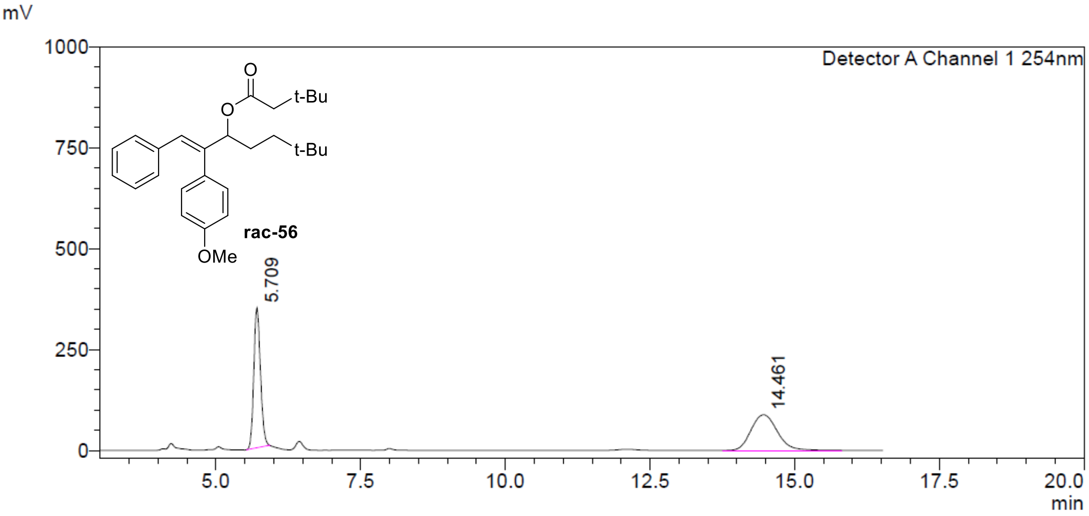

<Peak Table>

Detector A Channel 1 254nm

Peak#	Ret. Time	Height	Height%	Area	Area%
1	5.709	346434	79.727	2771163	50.041
2	14.461	88090	20.273	2766579	49.959
Total		434524	100.000	5537742	100.000

Supplementary Figure 411. Racemic Chromatogram of compound **56**

<Chromatogram>

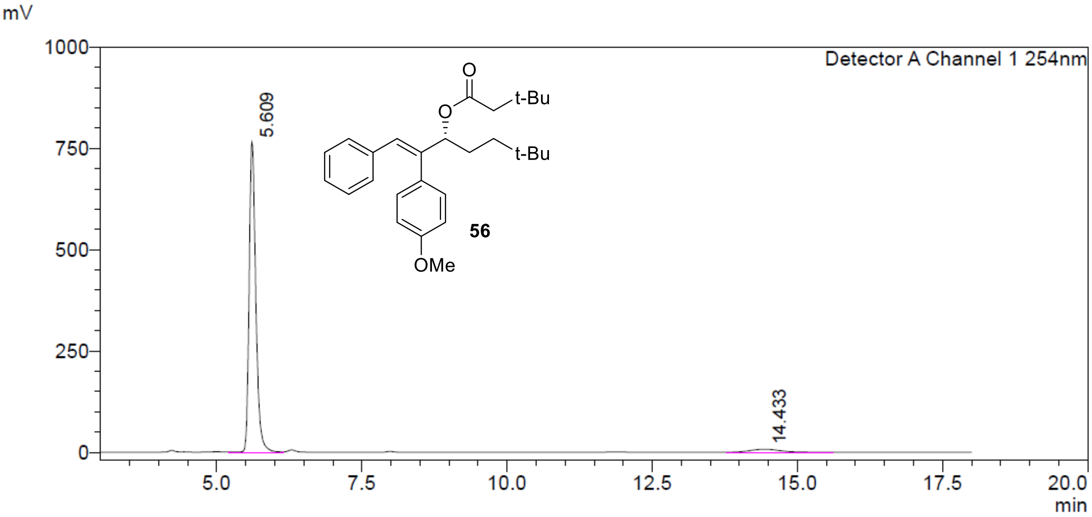

<Peak Table>

Detector A Channel 1 254nm

Peak#	Ret. Time	Height	Height%	Area	Area%
1	5.609	764923	99.027	6120312	95.930
2	14.433	7515	0.973	259655	4.070
Total		772438	100.000	6379967	100.000

Supplementary Figure 412. Scalemic Chromatogram of compound **56**

<Chromatogram>

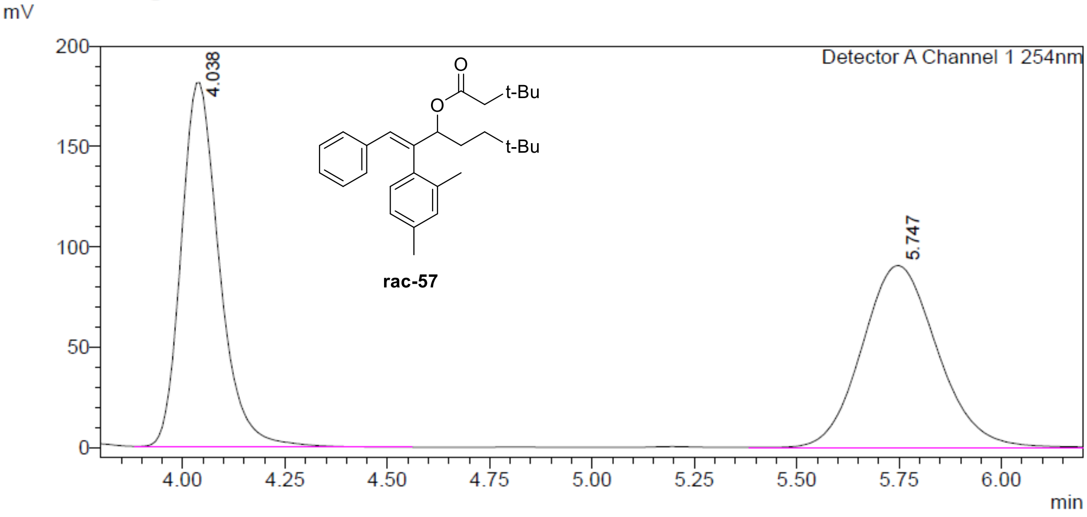

<Peak Table>

Detector A Channel 1 254nm

Peak#	Ret. Time	Height	Height%	Area	Area%
1	4.038	181602	66.700	1180284	50.414
2	5.747	90663	33.300	1160919	49.586
Total		272266	100.000	2341203	100.000

Supplementary Figure 413. Racemic Chromatogram of compound **57**

<Chromatogram>

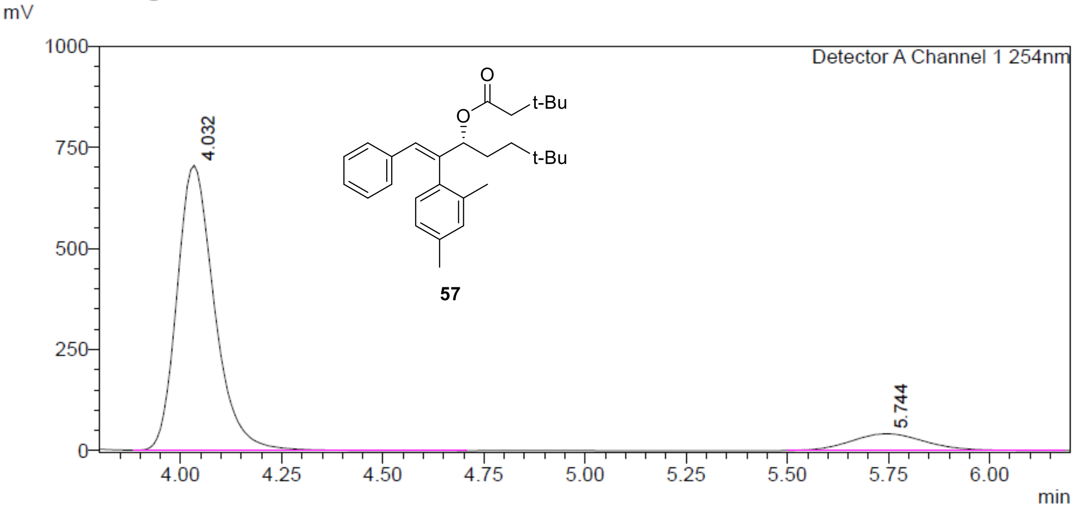

<Peak Table>

Detector A Channel 1 254nm

Peak#	Ret. Time	Height	Height%	Area	Area%
1	4.032	705454	94.471	4543030	89.621
2	5.744	41285	5.529	526135	10.379
Total		746739	100.000	5069165	100.000

Supplementary Figure 414. Scalemic Chromatogram of compound **57**

<Chromatogram>

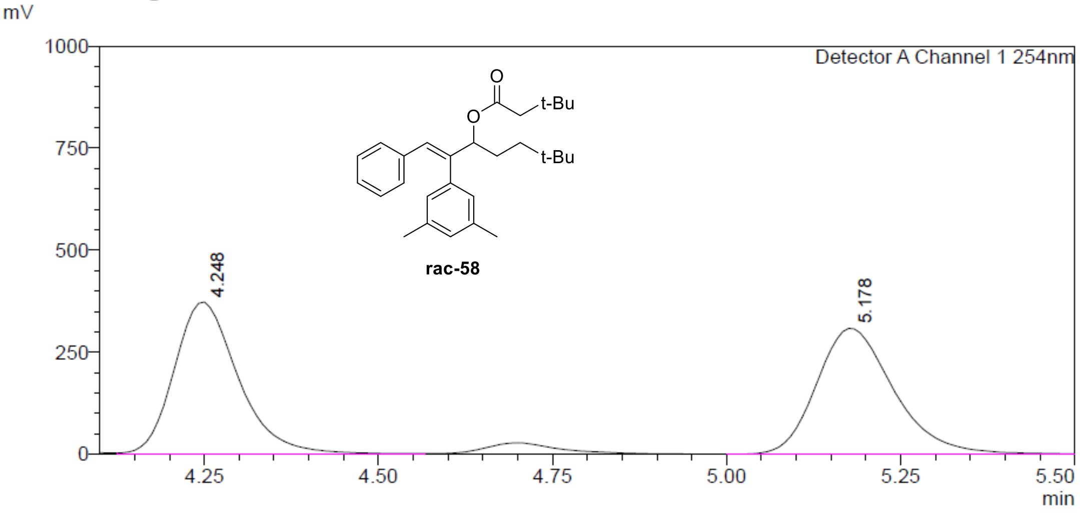

<Peak Table>

Detector A Channel 1 254nm

Peak#	Ret. Time	Height	Height%	Area	Area%
1	4.248	372700	54.749	2392719	50.121
2	5.178	308048	45.251	2381163	49.879
Total		680748	100.000	4773882	100.000

Supplementary Figure 415. Racemic Chromatogram of compound **58**

<Chromatogram>

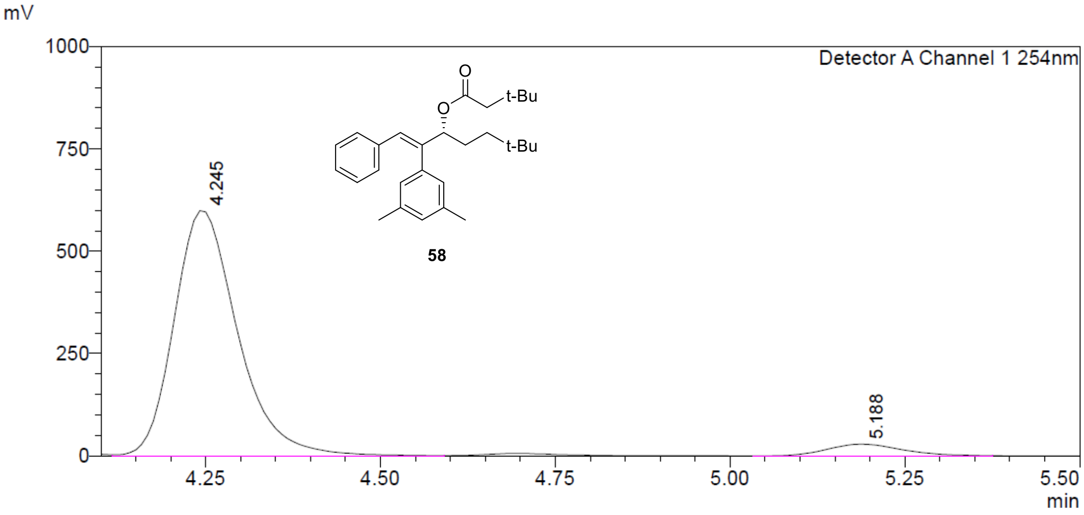

<Peak Table>

Detector A Channel 1 254nm

Peak#	Ret. Time	Height	Height%	Area	Area%
1	4.245	598520	95.449	3782547	94.599
2	5.188	28539	4.551	215965	5.401
Total		627058	100.000	3998512	100.000

Supplementary Figure 416. Scalemic Chromatogram of compound **56**

<Chromatogram>

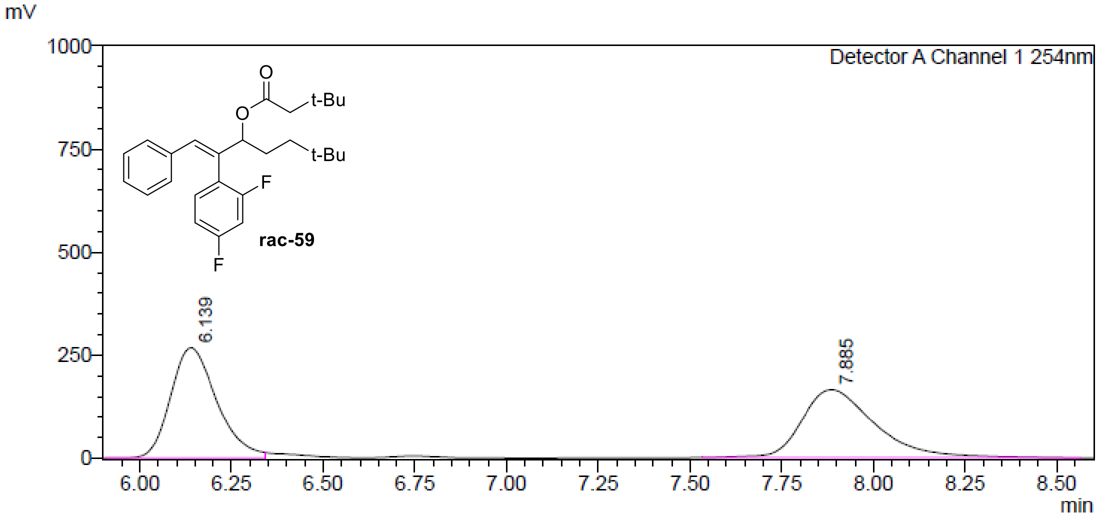

<Peak Table>

Detector A Channel 1 254nm

Peak#	Ret. Time	Height	Height%	Area	Area%
1	6.139	266437	61.760	2328186	50.497
2	7.885	164973	38.240	2282379	49.503
Total		431409	100.000	4610565	100.000

Supplementary Figure 417. Racemic Chromatogram of compound **59**

<Chromatogram>

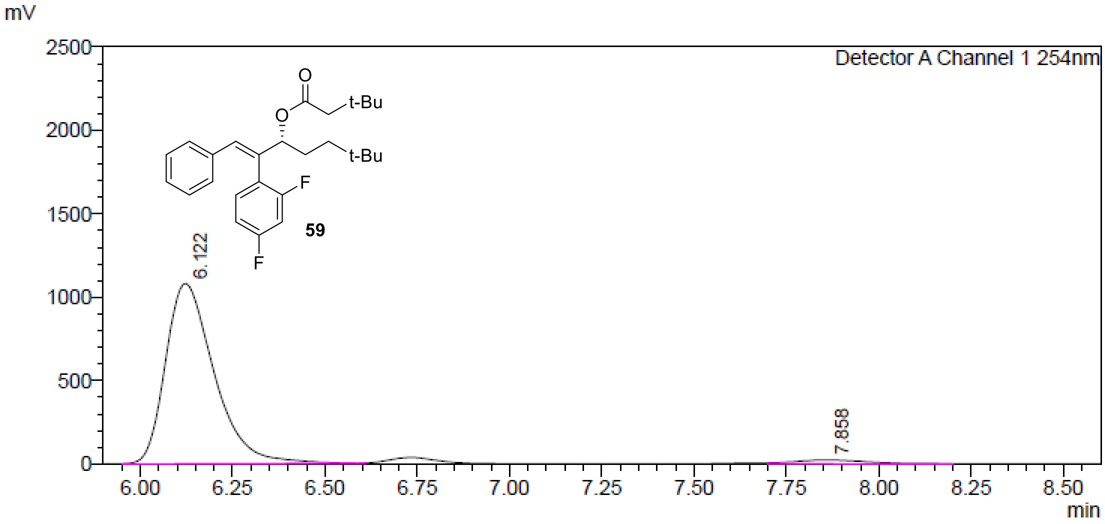

<Peak Table>

Detector A Channel 1 254nm

Peak#	Ret. Time	Height	Height%	Area	Area%
1	6.122	1079794	98.012	9865802	97.194
2	7.858	21897	1.988	284789	2.806
Total		1101691	100.000	10150591	100.000

Supplementary Figure 418. Scalemic Chromatogram of compound **59**

<Chromatogram>

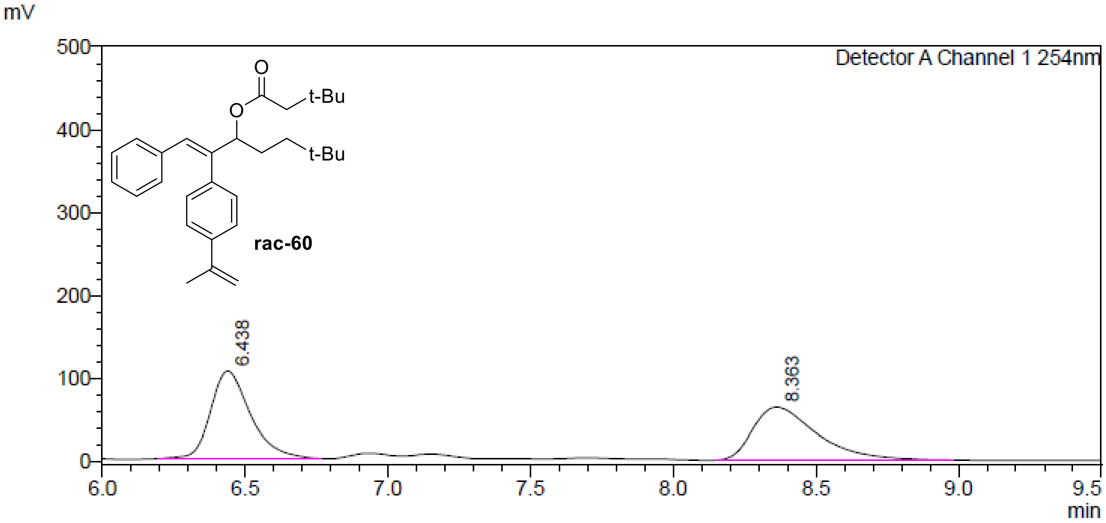

<Peak Table>

Detector A Channel 1 254nm

Peak#	Ret. Time	Height	Height%	Area	Area%
1	6.438	105452	62.422	1031515	50.978
2	8.363	63481	37.578	991950	49.022
Total		168933	100.000	2023464	100.000

Supplementary Figure 419. Racemic Chromatogram of compound **60**

<Chromatogram>

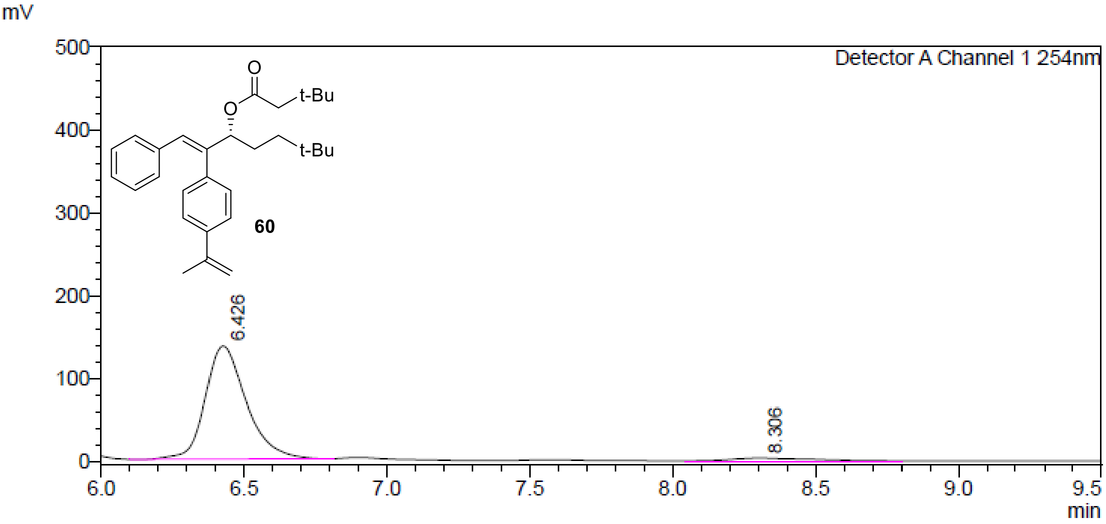

<Peak Table>

Detector A Channel 1 254nm

Peak#	Ret. Time	Height	Height%	Area	Area%
1	6.426	136460	97.479	1359436	95.195
2	8.306	3529	2.521	68621	4.805
Total		139989	100.000	1428057	100.000

Supplementary Figure 420. Scalemic Chromatogram of compound **60**

<Chromatogram>

mV

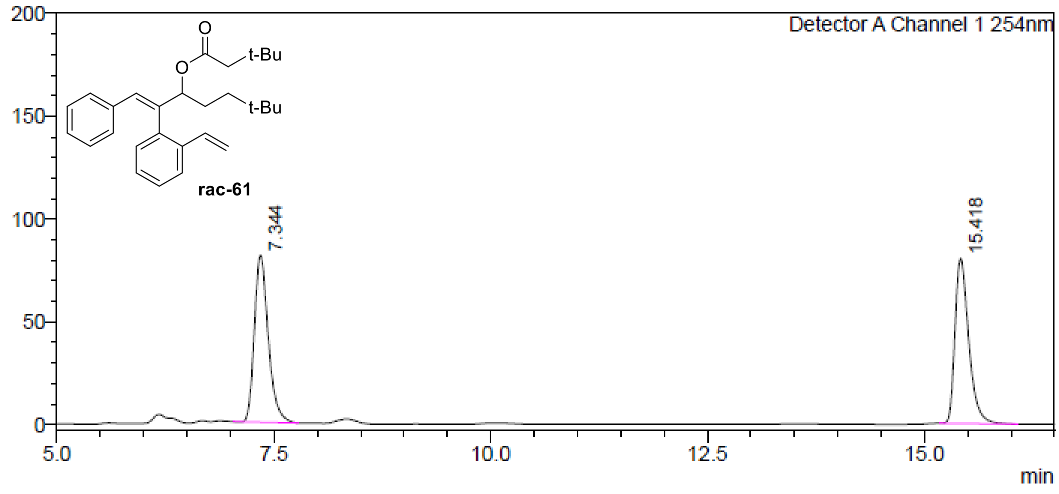

<Peak Table>

Detector A Channel 1 254nm

Peak#	Ret. Time	Height	Height%	Area	Area%
1	7.344	81229	50.264	908231	50.526
2	15.418	80376	49.736	889316	49.474
Total		161605	100.000	1797547	100.000

Supplementary Figure 421. Racemic Chromatogram of compound **61**

<Chromatogram>

mV

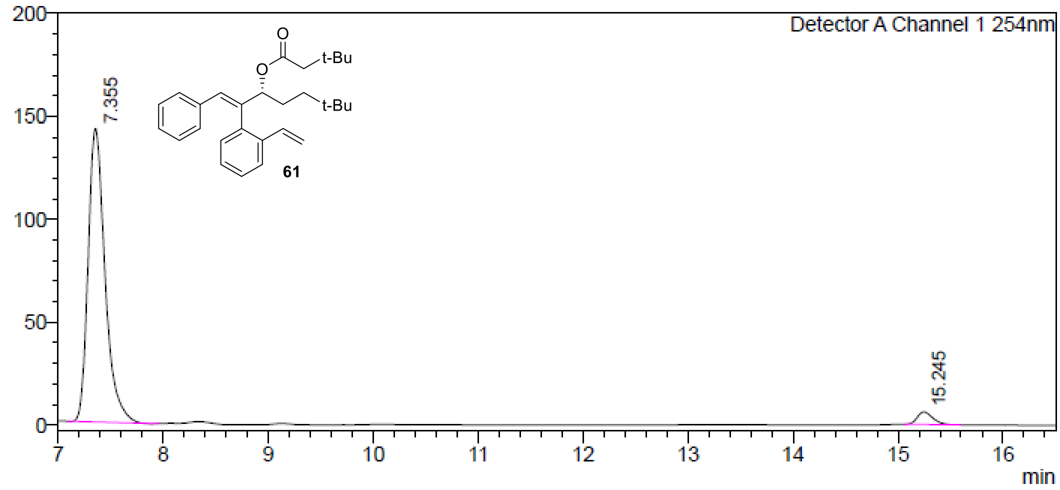

<Peak Table>

Detector A Channel 1 254nm

Peak#	Ret. Time	Height	Height%	Area	Area%
1	7.355	142691	95.928	1602412	96.279
2	15.245	6058	4.072	61935	3.721
Total		148749	100.000	1664346	100.000

Supplementary Figure 422. Scalemic Chromatogram of compound **61**

<Chromatogram>

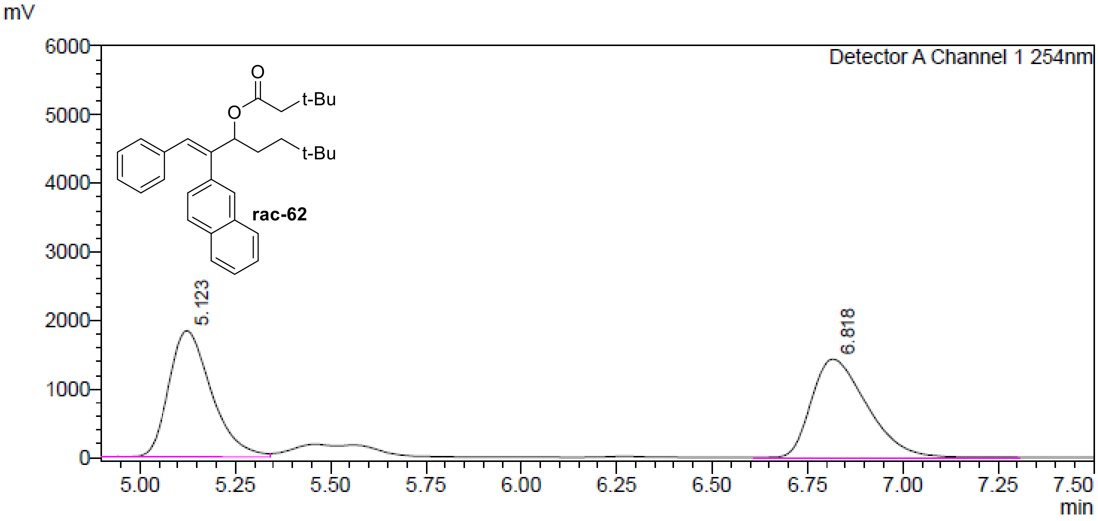

<Peak Table>

Detector A Channel 1 254nm

Peak#	Ret. Time	Height	Height%	Area	Area%
1	5.123	1840038	56.195	14286324	49.651
2	6.818	1434369	43.805	14486968	50.349
Total		3274407	100.000	28773291	100.000

Supplementary Figure 423. Racemic Chromatogram of compound **62**

<Chromatogram>

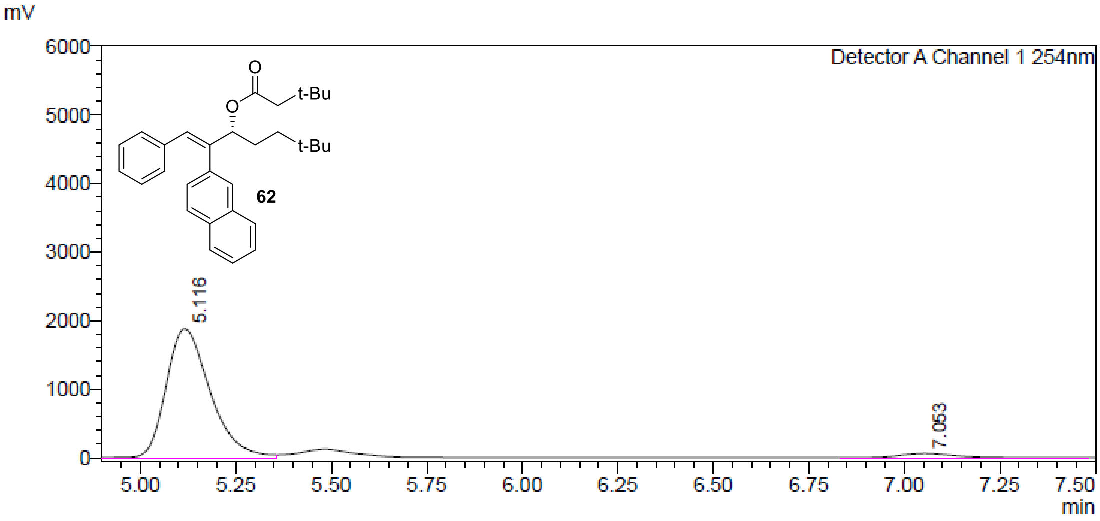

<Peak Table>

Detector A Channel 1 254nm

Peak#	Ret. Time	Height	Height%	Area	Area%
1	5.116	1885222	96.736	15024095	95.864
2	7.053	63603	3.264	648215	4.136
Total		1948825	100.000	15672310	100.000

Supplementary Figure 424. Scalemic Chromatogram of compound **62**

<Chromatogram>

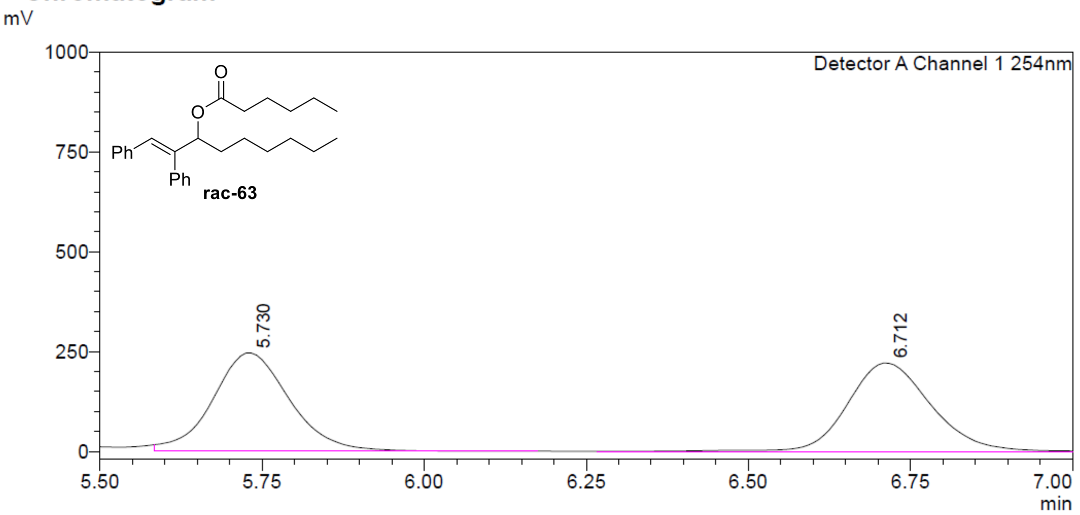

<Peak Table>

Detector A Channel 1 254nm

Peak#	Ret. Time	Height	Height%	Area	Area%
1	5.730	245909	52.658	2026263	50.733
2	6.712	221084	47.342	1967688	49.267
Total		466993	100.000	3993951	100.000

Supplementary Figure 425. Racemic Chromatogram of compound **63**

<Chromatogram>

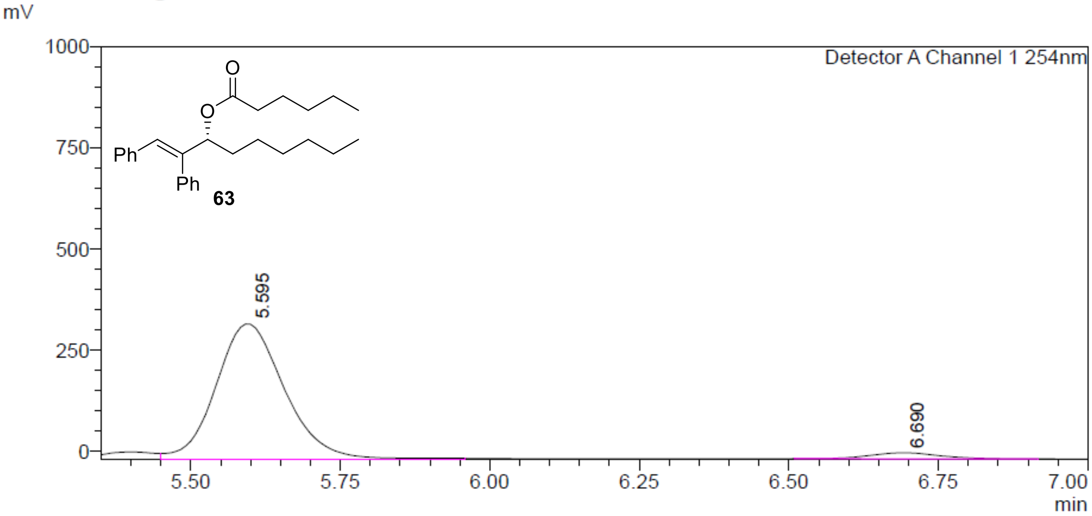

<Peak Table>

Detector A Channel 1 254nm

Peak#	Ret. Time	Height	Height%	Area	Area%
1	5.595	333101	95.741	2630988	95.628
2	6.690	14819	4.259	120282	4.372
Total		347919	100.000	2751270	100.000

Supplementary Figure 426. Scalemic Chromatogram of compound **63**

<Chromatogram>

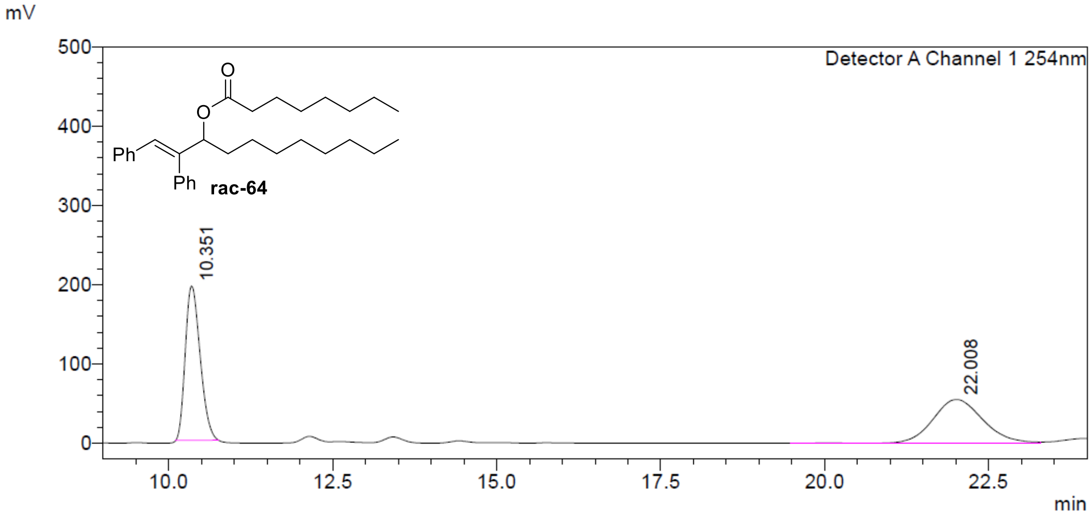

<Peak Table>

Detector A Channel 1 254nm

Peak#	Ret. Time	Height	Height%	Area	Area%
1	10.351	194658	77.917	3041119	50.729
2	22.008	55170	22.083	2953762	49.271
Total		249827	100.000	5994881	100.000

Supplementary Figure 427. Racemic Chromatogram of compound **64**

<Chromatogram>

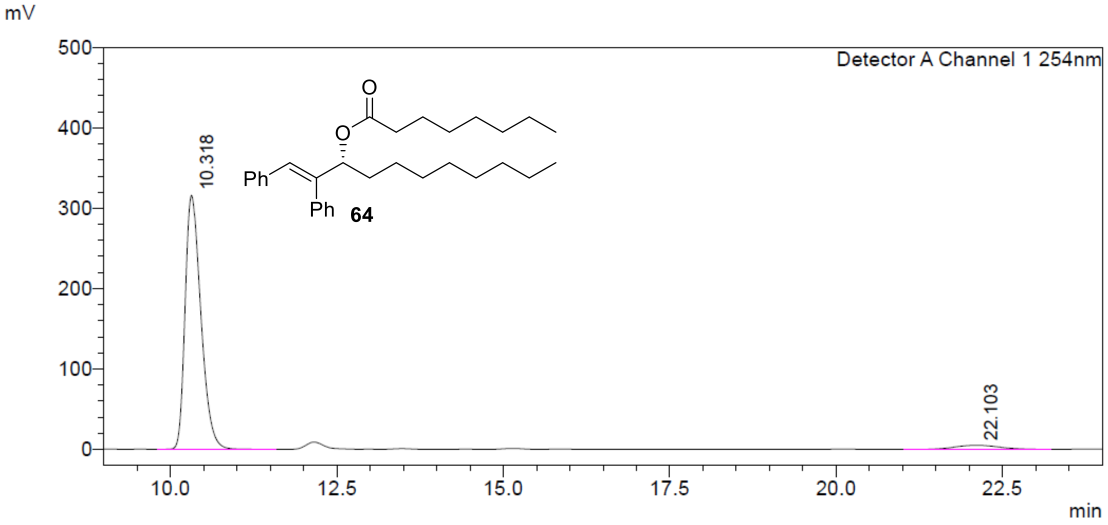

<Peak Table>

Detector A Channel 1 254nm

Peak#	Ret. Time	Height	Height%	Area	Area%
1	10.318	315900	98.431	5183264	95.455
2	22.103	5034	1.569	246798	4.545
Total		320934	100.000	5430063	100.000

Supplementary Figure 428. Scalemic Chromatogram of compound **64**

<Chromatogram>

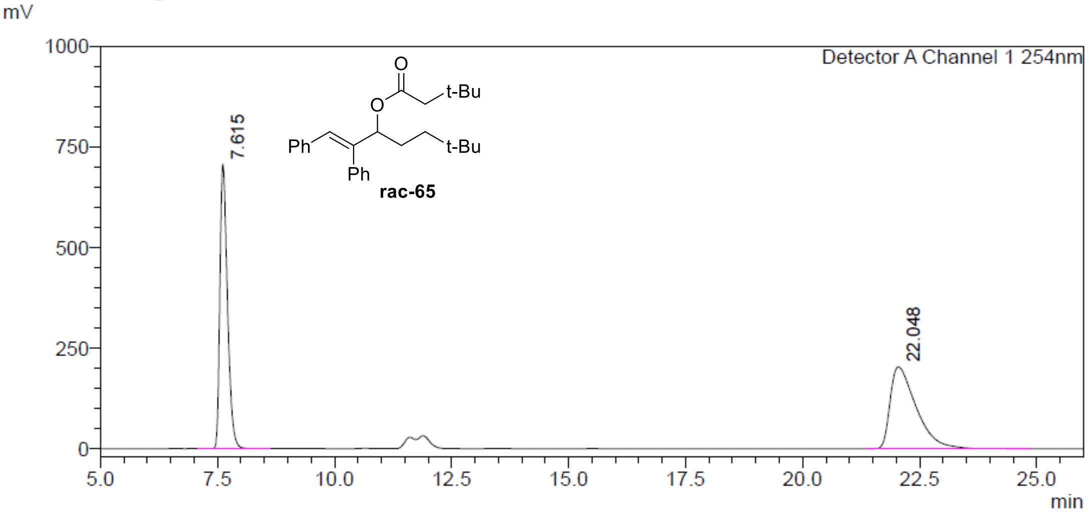

<Peak Table>

Detector A Channel 1 254nm

Peak#	Ret. Time	Height	Height%	Area	Area%
1	7.615	704668	77.587	7948200	50.063
2	22.048	203567	22.413	7928281	49.937
Total		908234	100.000	15876481	100.000

Supplementary Figure 429. Racemic Chromatogram of compound **65**

<Chromatogram>

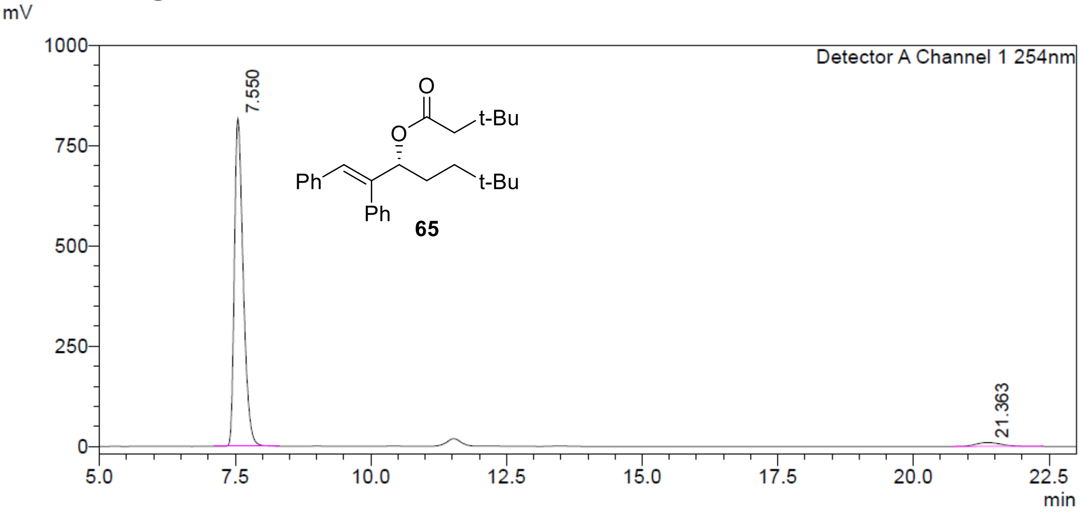

<Peak Table>

Detector A Channel 1 254nm

Peak#	Ret. Time	Height	Height%	Area	Area%
1	7.550	816932	98.858	9281189	96.771
2	21.363	9435	1.142	309720	3.229
Total		826366	100.000	9590909	100.000

Supplementary Figure 430. Scalemic Chromatogram of compound **65**

<Chromatogram>

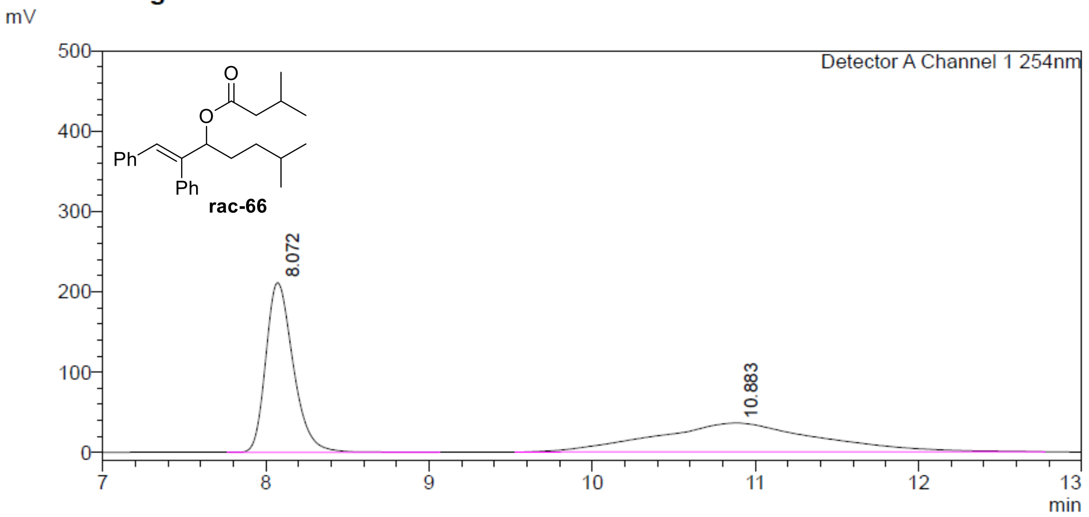

<Peak Table>

Detector A Channel 1 254nm

Peak#	Ret. Time	Height	Height%	Area	Area%
1	8.072	211331	85.498	2474114	49.988
2	10.883	35844	14.502	2475261	50.012
Total		247175	100.000	4949375	100.000

Supplementary Figure 431. Racemic Chromatogram of compound **66**

<Chromatogram>

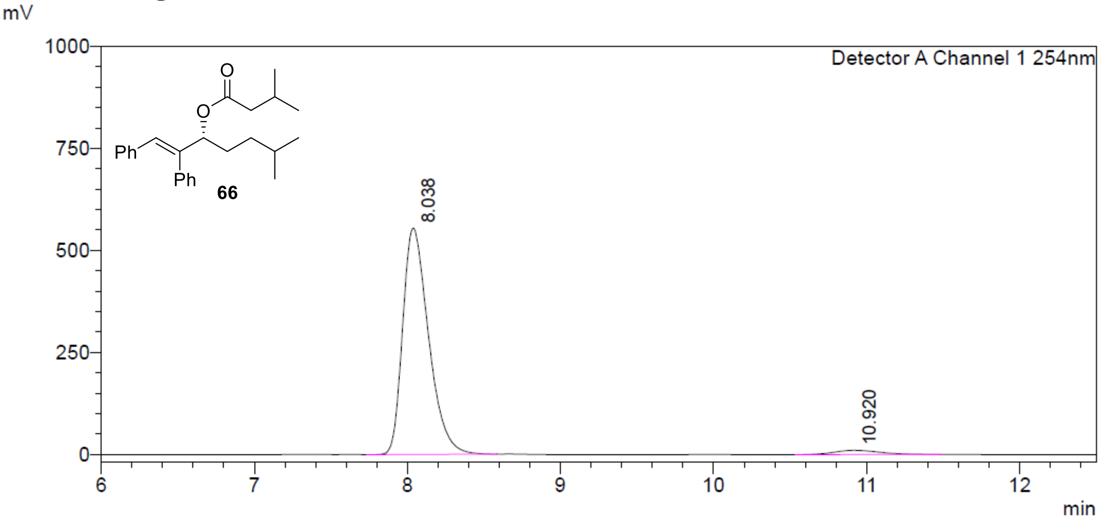

<Peak Table>

Detector A Channel 1 254nm

Peak#	Ret. Time	Height	Height%	Area	Area%
1	8.038	554572	98.204	6514617	96.834
2	10.920	10144	1.796	213009	3.166
Total		564716	100.000	6727626	100.000

Supplementary Figure 432. Scalemic Chromatogram of compound **66**

<Chromatogram>

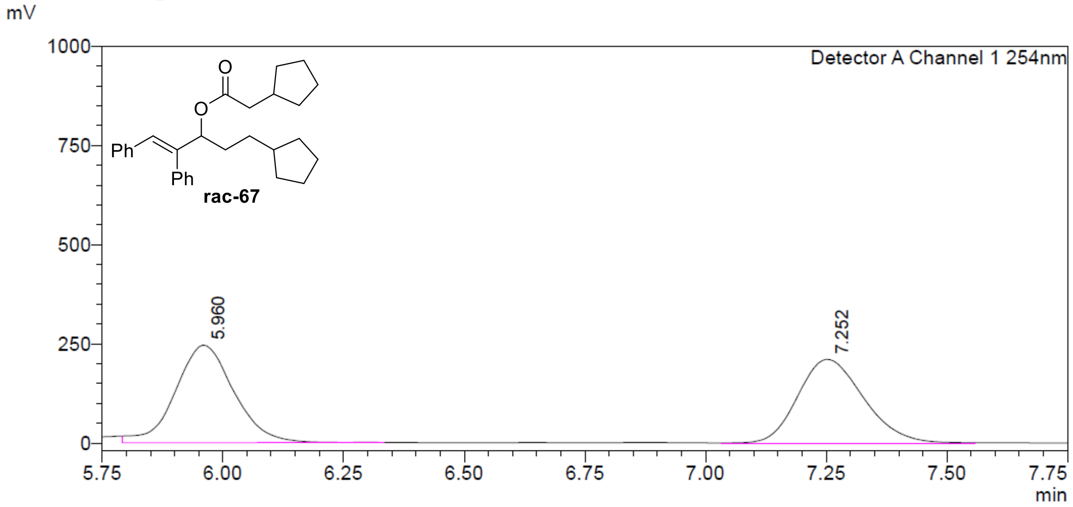

<Peak Table>

Detector A Channel 1 254nm

Peak#	Ret. Time	Height	Height%	Area	Area%
1	5.960	245671	53.792	2062469	50.823
2	7.252	211033	46.208	1995707	49.177
Total		456705	100.000	4058176	100.000

Supplementary Figure 433. Racemic Chromatogram of compound **67**

<Chromatogram>

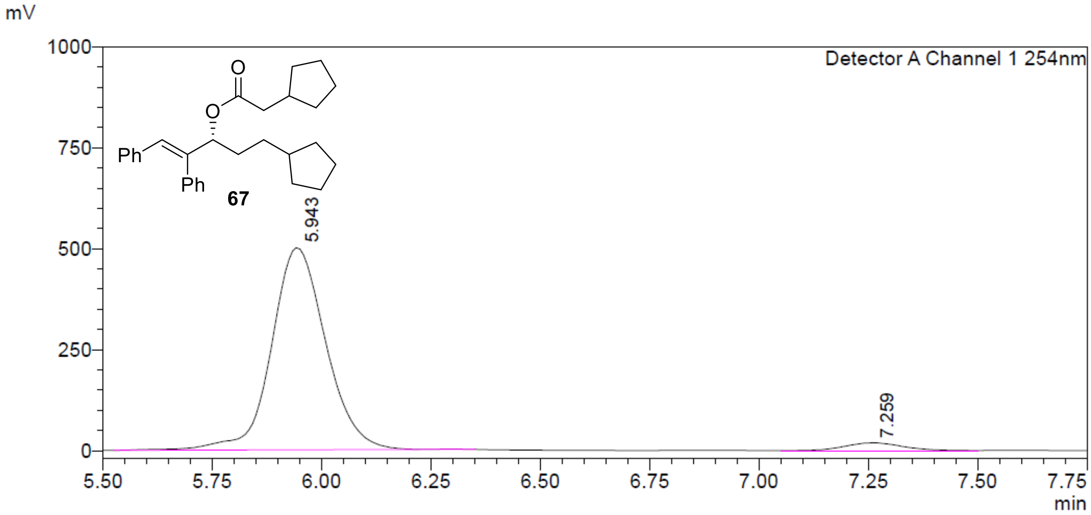

<Peak Table>

Detector A Channel 1 254nm

Peak#	Ret. Time	Height	Height%	Area	Area%
1	5.943	500989	96.307	4349025	96.132
2	7.259	19212	3.693	175008	3.868
Total		520201	100.000	4524033	100.000

Supplementary Figure 434. Scalemic Chromatogram of compound **67**

<Chromatogram>

mV

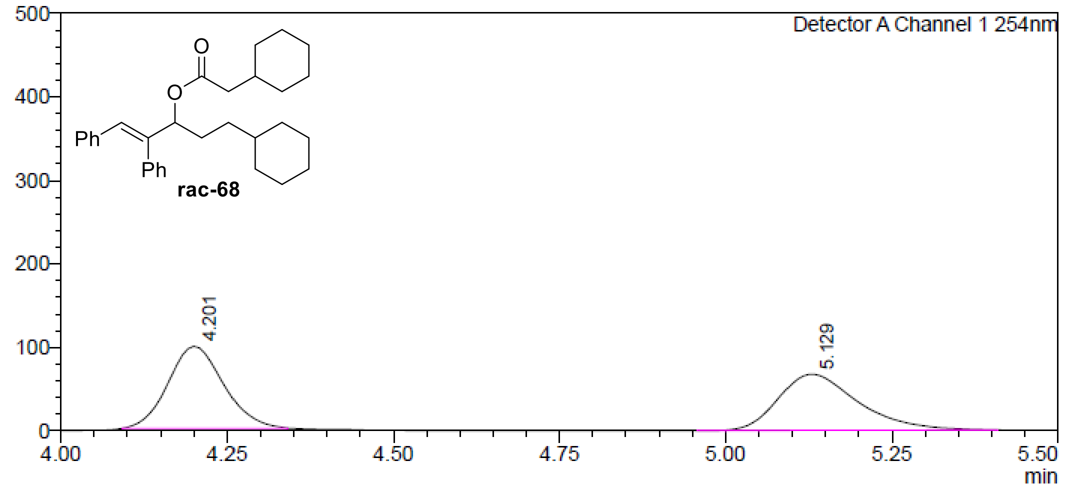

<Peak Table>

Detector A Channel 1 254nm

Peak#	Ret. Time	Height	Height%	Area	Area%
1	4.201	98384	59.373	571277	50.472
2	5.129	67321	40.627	560603	49.528
Total		165705	100.000	1131880	100.000

Supplementary Figure 435. Racemic Chromatogram of compound **68**

<Chromatogram>

mV

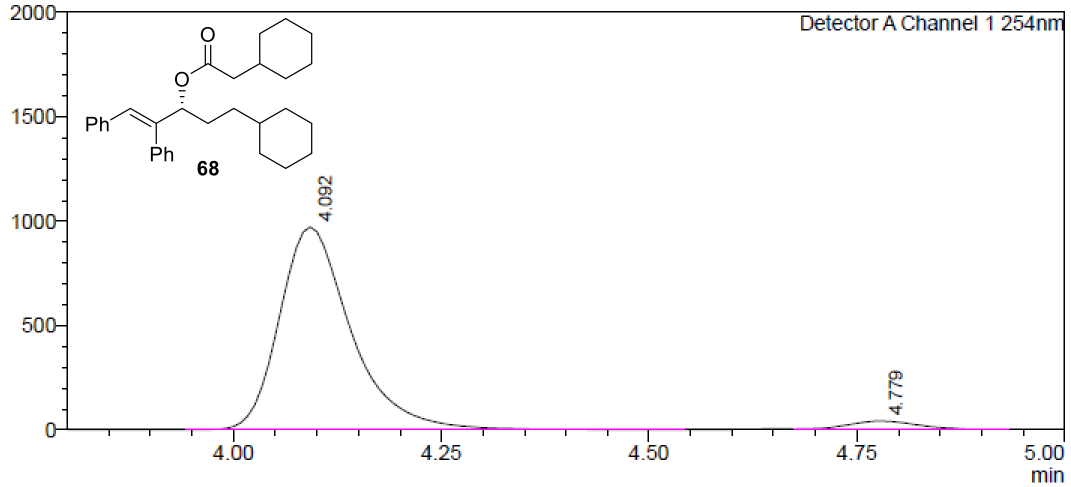

<Peak Table>

Detector A Channel 1 254nm

Peak#	Ret. Time	Height	Height%	Area	Area%
1	4.092	973314	95.837	5853023	95.721
2	4.779	42278	4.163	261645	4.279
Total		1015591	100.000	6114669	100.000

Supplementary Figure 436. Scalemic Chromatogram of compound **68**

<Chromatogram>

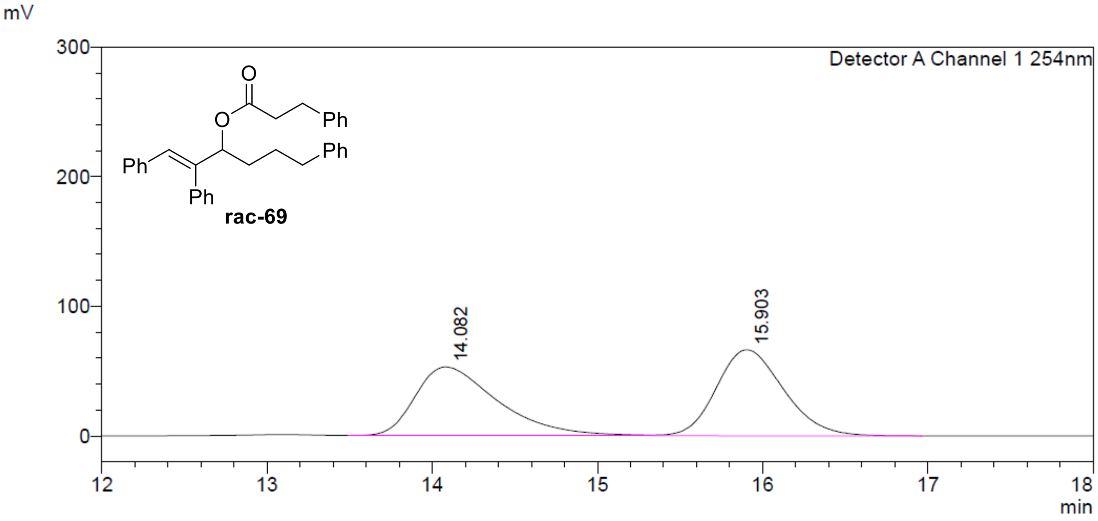

<Peak Table>

Detector A Channel 1 254nm

Peak#	Ret. Time	Height	Height%	Area	Area%
1	14.082	53124	44.430	1855858	49.637
2	15.903	66443	55.570	1883006	50.363
Total		119567	100.000	3738864	100.000

Supplementary Figure 437. Racemic Chromatogram of compound **69**

<Chromatogram>

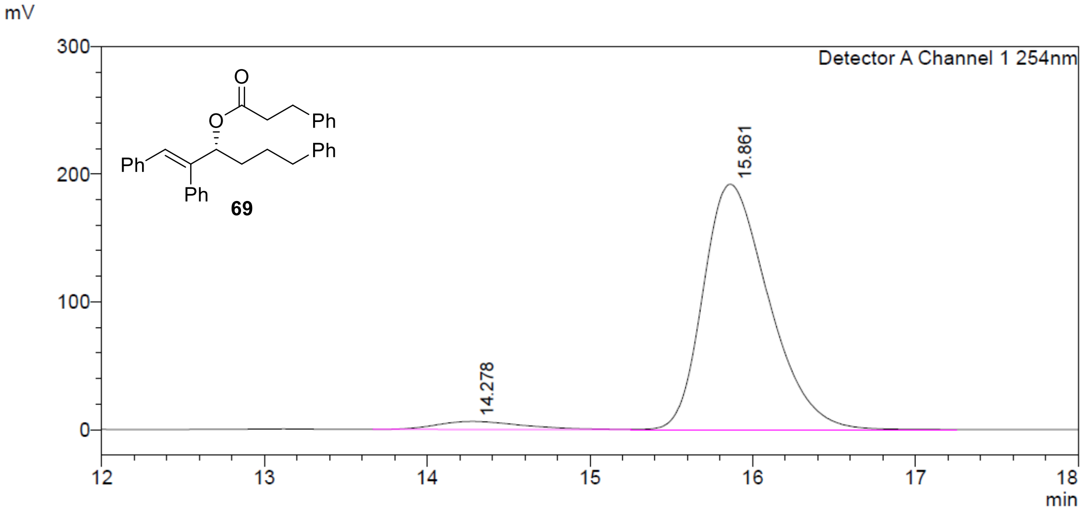

<Peak Table>

Detector A Channel 1 254nm

Peak#	Ret. Time	Height	Height%	Area	Area%
1	14.278	6336	3.194	232478	4.145
2	15.861	192055	96.806	5375569	95.855
Total		198391	100.000	5608047	100.000

Supplementary Figure 438. Scalemic Chromatogram of compound **69**

<Chromatogram>

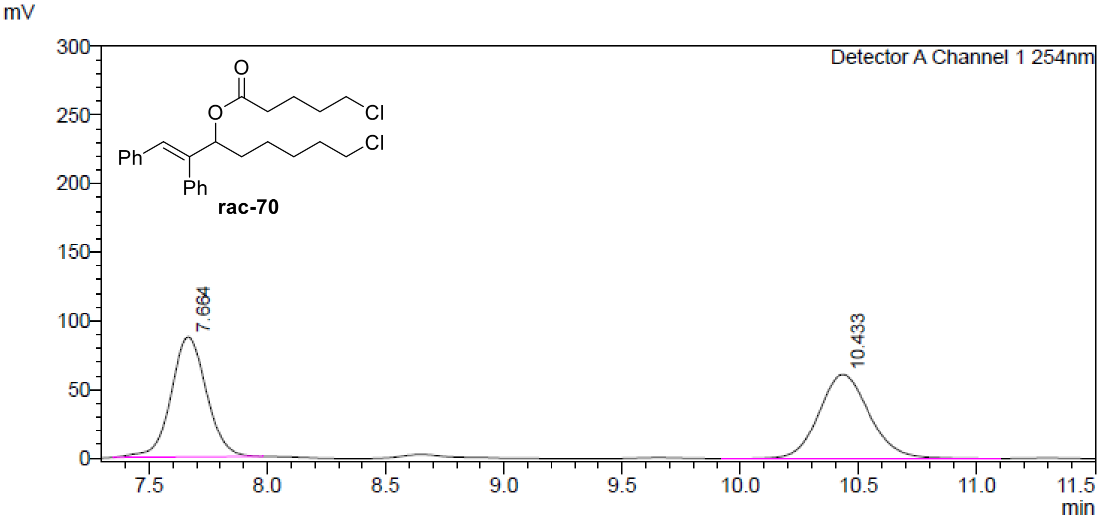

<Peak Table>

Detector A Channel 1 254nm

Peak#	Ret. Time	Height	Height%	Area	Area%
1	7.664	87493	58.740	911572	49.914
2	10.433	61457	41.260	914719	50.086
Total		148950	100.000	1826290	100.000

Supplementary Figure 439. Racemic Chromatogram of compound **70**

<Chromatogram>

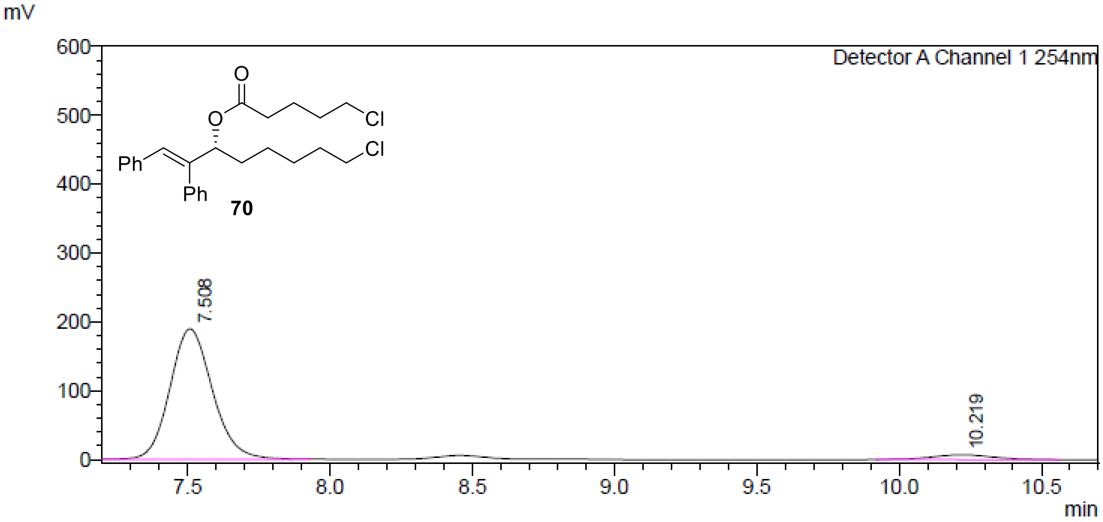

<Peak Table>

Detector A Channel 1 254nm

Peak#	Ret. Time	Height	Height%	Area	Area%
1	7.508	189552	96.489	1972919	95.112
2	10.219	6898	3.511	101389	4.888
Total		196450	100.000	2074308	100.000

Supplementary Figure 440. Scalemic Chromatogram of compound **70**

<Chromatogram>

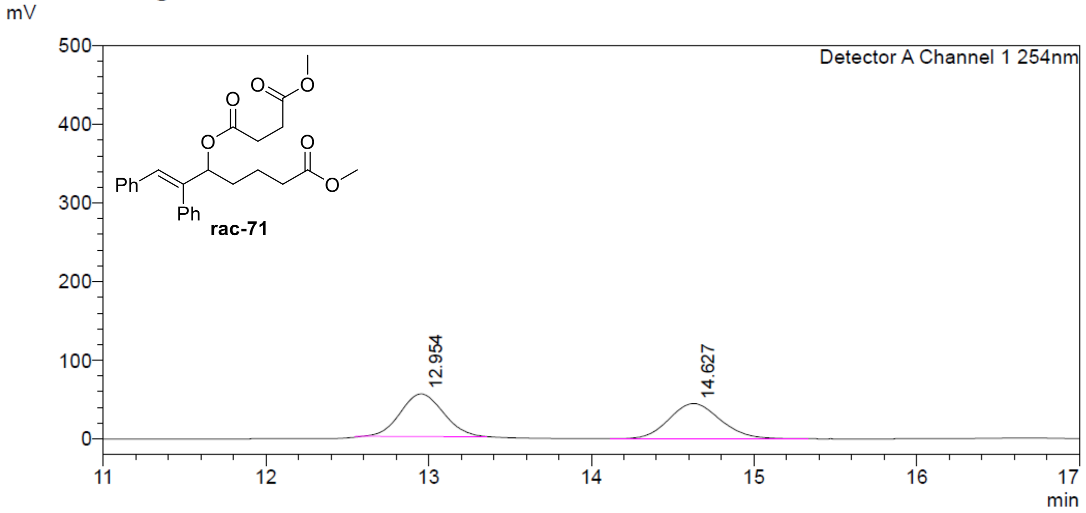

<Peak Table>

Detector A Channel 1 254nm

Peak#	Ret. Time	Height	Height%	Area	Area%
1	12.954	54182	54.735	1001623	50.426
2	14.627	44807	45.265	984693	49.574
Total		98988	100.000	1986316	100.000

Supplementary Figure 441. Racemic Chromatogram of compound **71**

<Chromatogram>

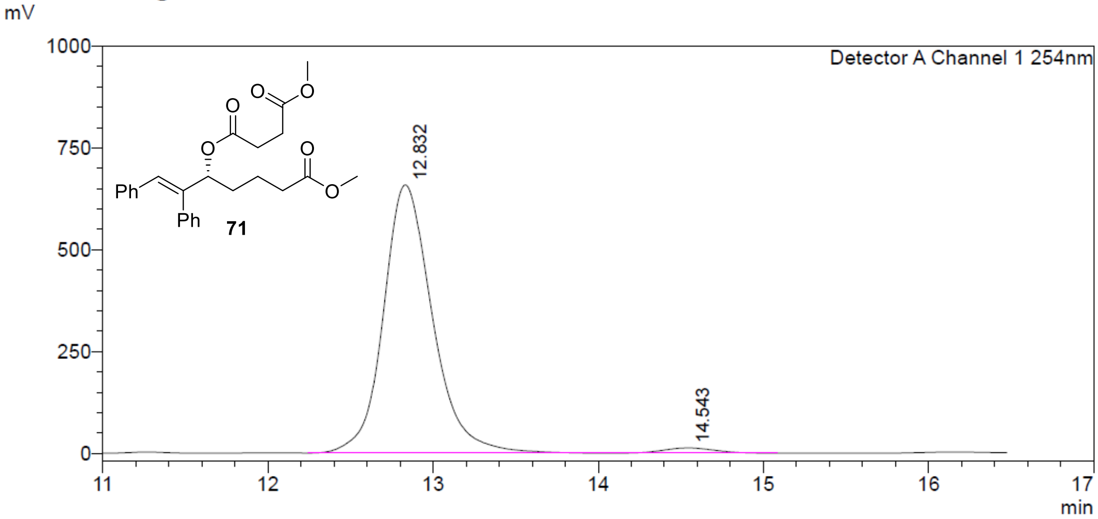

<Peak Table>

Detector A Channel 1 254nm

Peak#	Ret. Time	Height	Height%	Area	Area%
1	12.832	659142	98.097	13641862	97.999
2	14.543	12786	1.903	278565	2.001
Total		671928	100.000	13920427	100.000

Supplementary Figure 442. Scalemic Chromatogram of compound **71**

<Chromatogram>

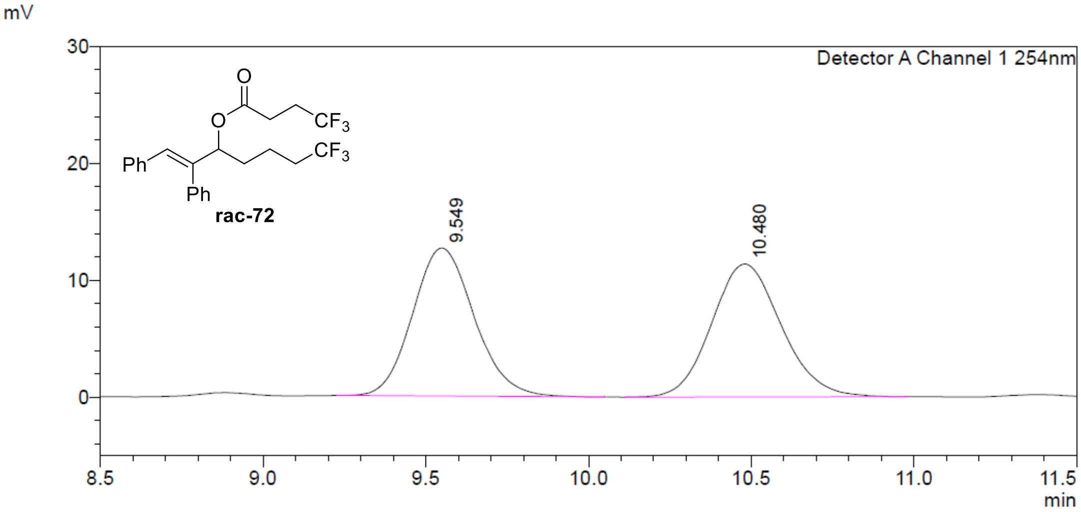

<Peak Table>

Detector A Channel 1 254nm

Peak#	Ret. Time	Height	Height%	Area	Area%
1	9.549	12675	52.715	167138	49.662
2	10.480	11369	47.285	169411	50.338
Total		24045	100.000	336548	100.000

Supplementary Figure 443. Racemic Chromatogram of compound **72**

<Chromatogram>

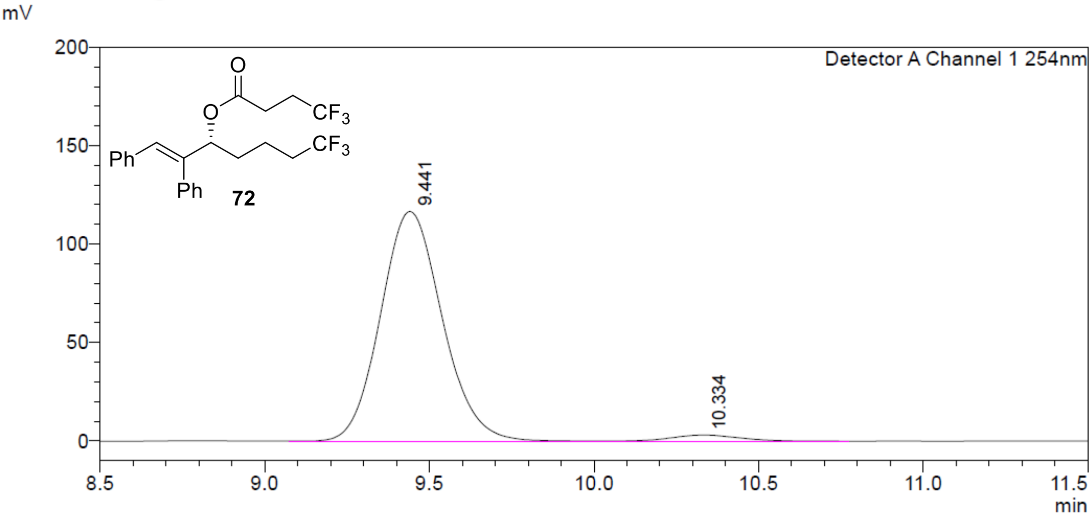

<Peak Table>

Detector A Channel 1 254nm

Peak#	Ret. Time	Height	Height%	Area	Area%
1	9.441	116833	97.395	1526987	97.044
2	10.334	3125	2.605	46518	2.956
Total		119959	100.000	1573506	100.000

Supplementary Figure 444. Scalemic Chromatogram of compound **72**

<Chromatogram>

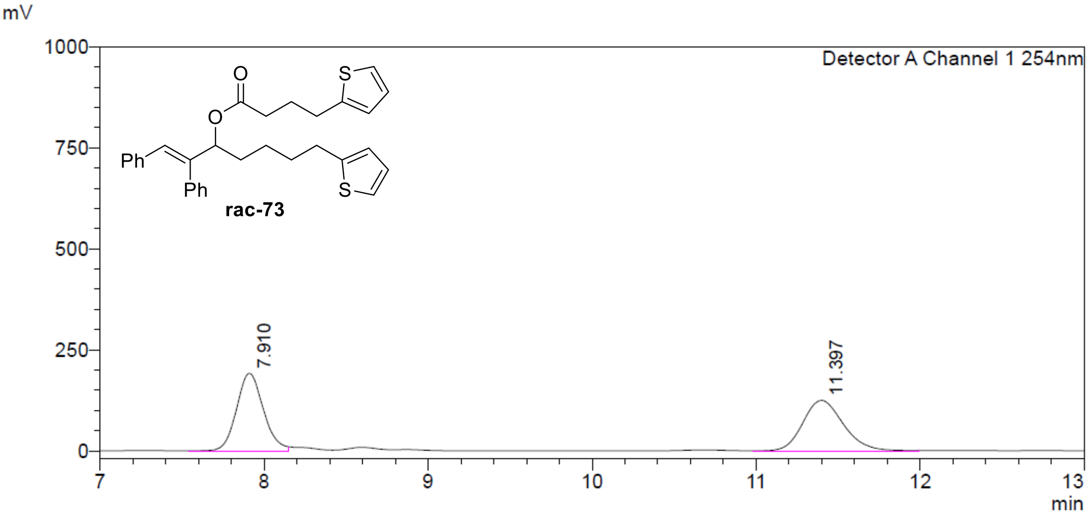

<Peak Table>

Detector A Channel 1 254nm

Peak#	Ret. Time	Height	Height%	Area	Area%
1	7.910	191961	60.587	2186531	50.477
2	11.397	124875	39.413	2145186	49.523
Total		316836	100.000	4331717	100.000

Supplementary Figure 445. Racemic Chromatogram of compound **73**

<Chromatogram>

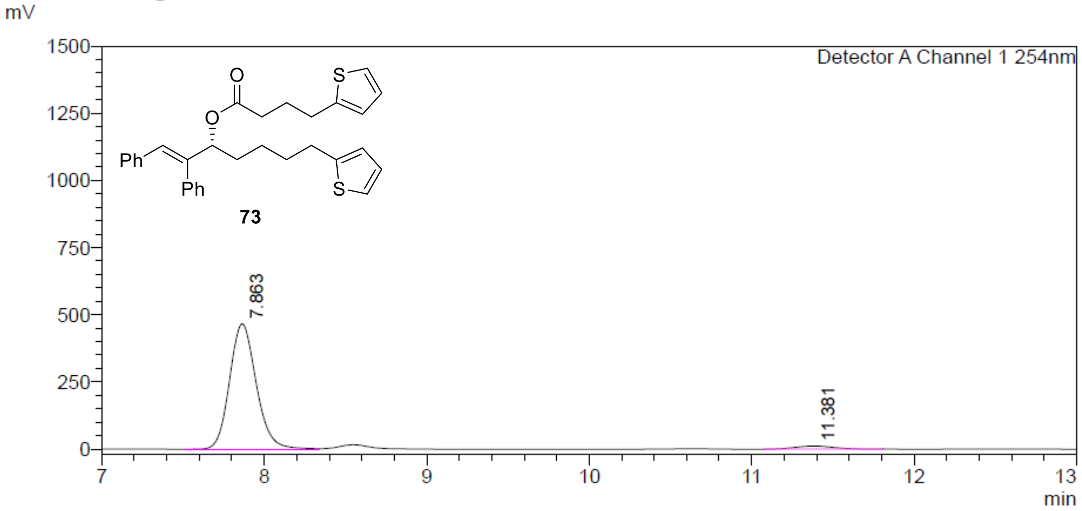

<Peak Table>

Detector A Channel 1 254nm

Peak#	Ret. Time	Height	Height%	Area	Area%
1	7.863	466254	97.710	5319803	96.681
2	11.381	10929	2.290	182623	3.319
Total		477183	100.000	5502426	100.000

Supplementary Figure 446. Scalemic Chromatogram of compound **73**

<Chromatogram>

mV

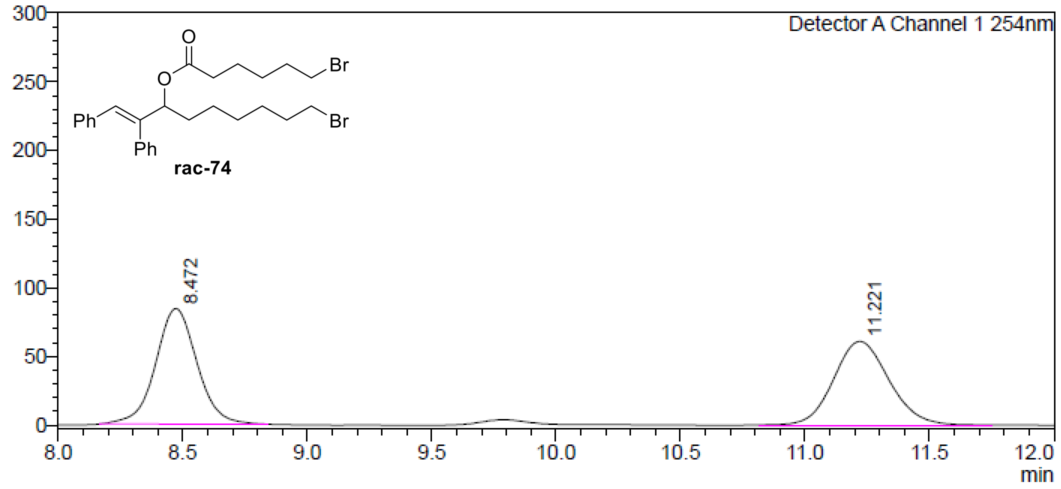

<Peak Table>

Detector A Channel 1 254nm

Peak#	Ret. Time	Height	Height%	Area	Area%
1	8.472	84060	58.033	973393	50.856
2	11.221	60789	41.967	940608	49.144
Total		144848	100.000	1914000	100.000

Supplementary Figure 447. Racemic Chromatogram of compound **74**

<Chromatogram>

mV

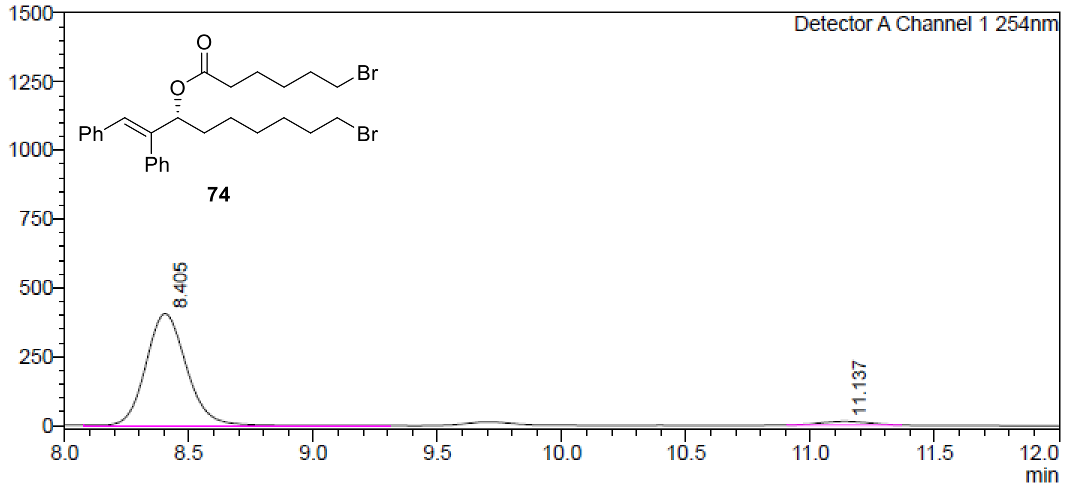

<Peak Table>

Detector A Channel 1 254nm

Peak#	Ret. Time	Height	Height%	Area	Area%
1	8.405	406440	96.825	4612596	96.263
2	11.137	13328	3.175	179071	3.737
Total		419769	100.000	4791668	100.000

Supplementary Figure 448. Scalemic Chromatogram of compound **74**

<Chromatogram>

mV

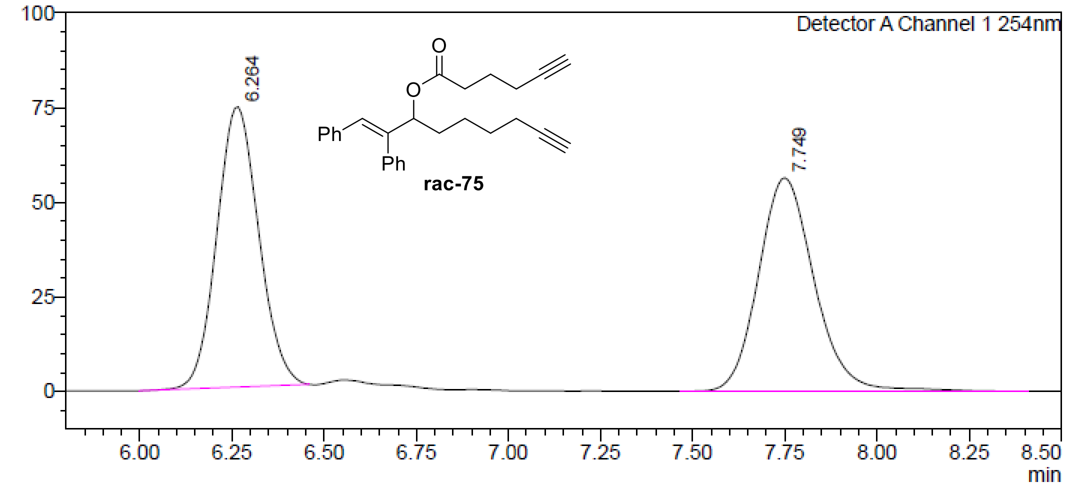

<Peak Table>

Detector A Channel 1 254nm

Peak#	Ret. Time	Height	Height%	Area	Area%
1	6.264	74043	56.743	586804	49.694
2	7.749	56445	43.257	594037	50.306
Total		130488	100.000	1180841	100.000

Supplementary Figure 449. Racemic Chromatogram of compound **75**

<Chromatogram>

mV

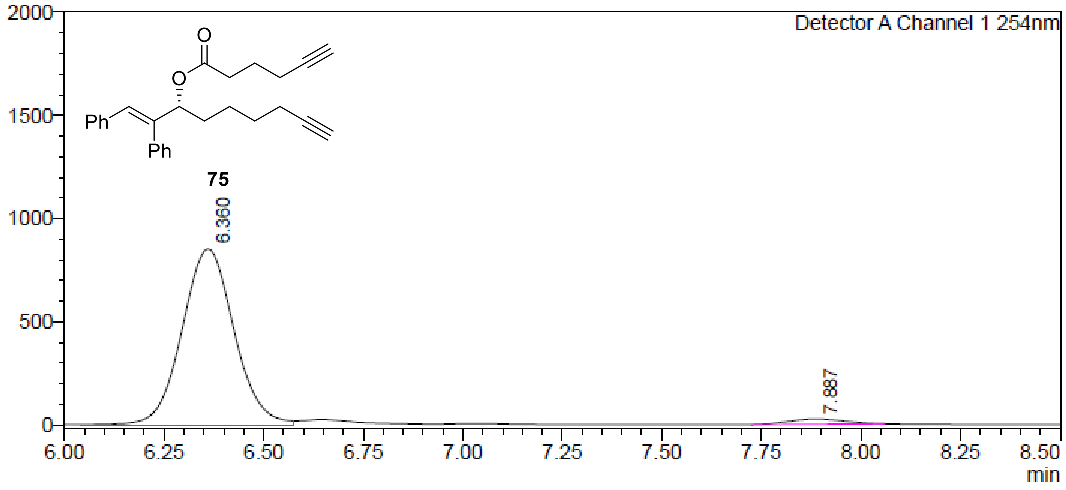

<Peak Table>

Detector A Channel 1 254nm

Peak#	Ret. Time	Height	Height%	Area	Area%
1	6.360	853701	97.136	7769553	96.955
2	7.887	25171	2.864	244023	3.045
Total		878872	100.000	8013576	100.000

Supplementary Figure 450. Scalemic Chromatogram of compound **75**

<Chromatogram>

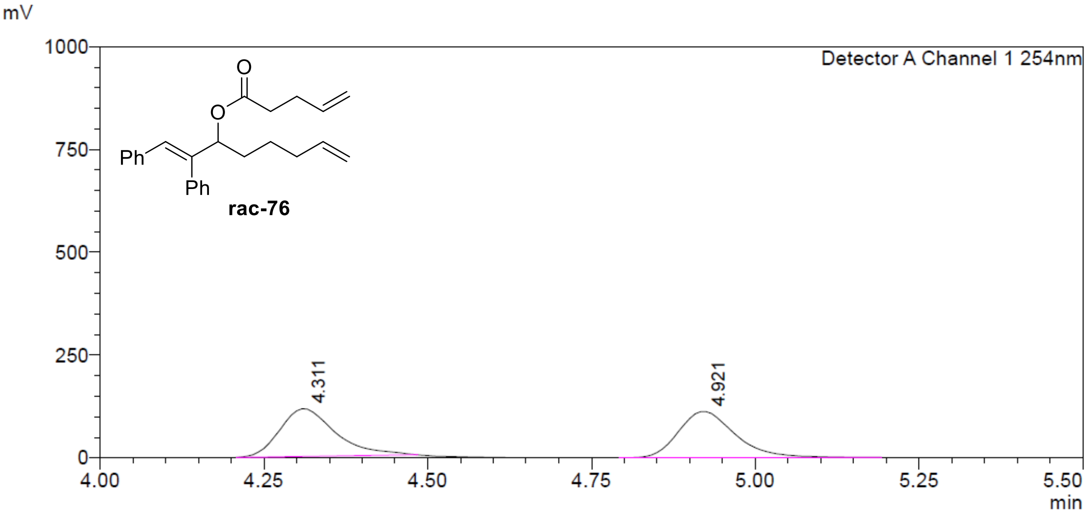

<Peak Table>

Detector A Channel 1 254nm

Peak#	Ret. Time	Height	Height%	Area	Area%
1	4.311	116212	50.876	687796	50.235
2	4.921	112210	49.124	681361	49.765
Total		228422	100.000	1369157	100.000

Supplementary Figure 451. Racemic Chromatogram of compound **76**

<Chromatogram>

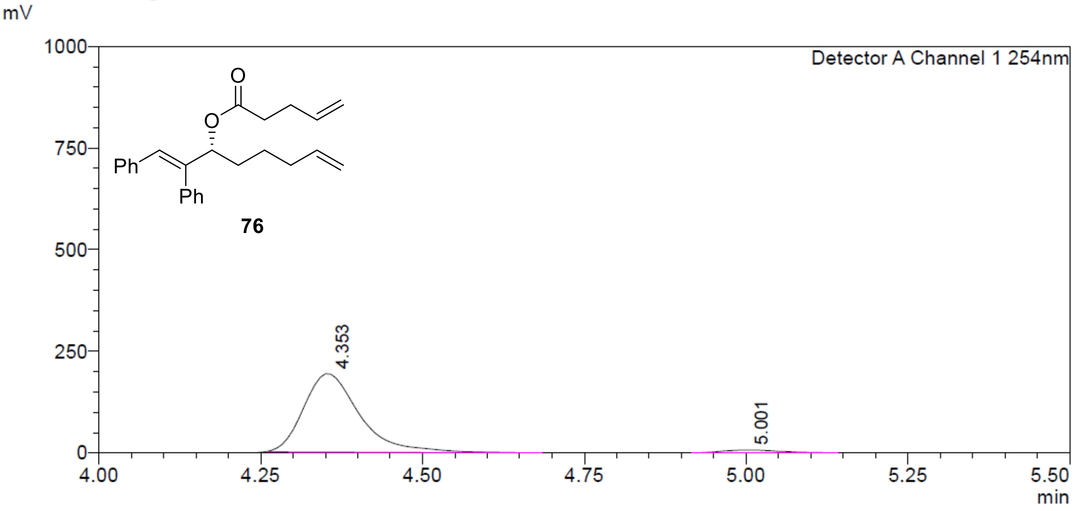

<Peak Table>

Detector A Channel 1 254nm

Peak#	Ret. Time	Height	Height%	Area	Area%
1	4.353	192267	96.499	1154083	96.512
2	5.001	6975	3.501	41709	3.488
Total		199242	100.000	1195792	100.000

Supplementary Figure 452. Scalemic Chromatogram of compound **76**

<Chromatogram>

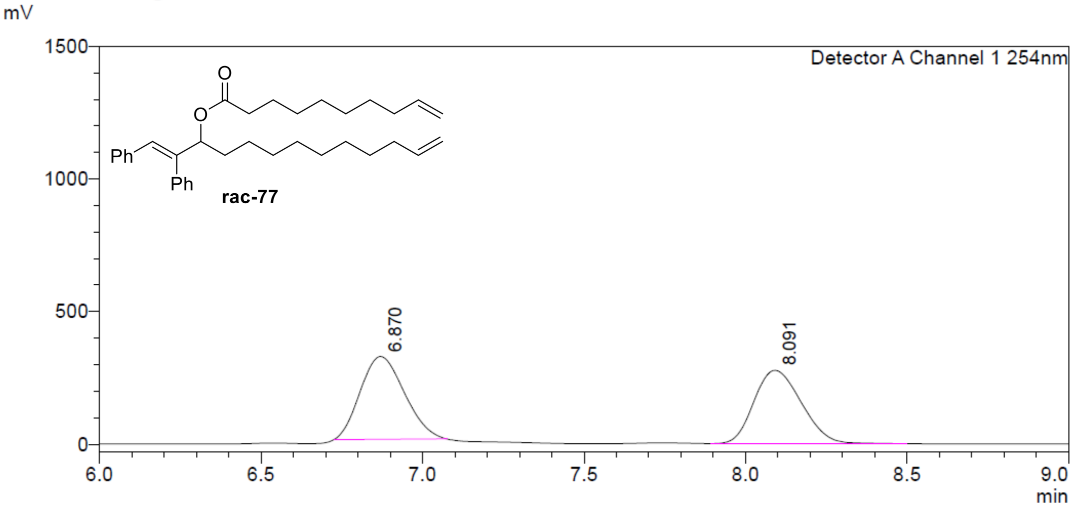

<Peak Table>

Detector A Channel 1 254nm

Peak#	Ret. Time	Height	Height%	Area	Area%
1	6.870	312597	52.929	2974154	50.900
2	8.091	278004	47.071	2868967	49.100
Total		590601	100.000	5843120	100.000

Supplementary Figure 453. Racemic Chromatogram of compound **77**

<Chromatogram>

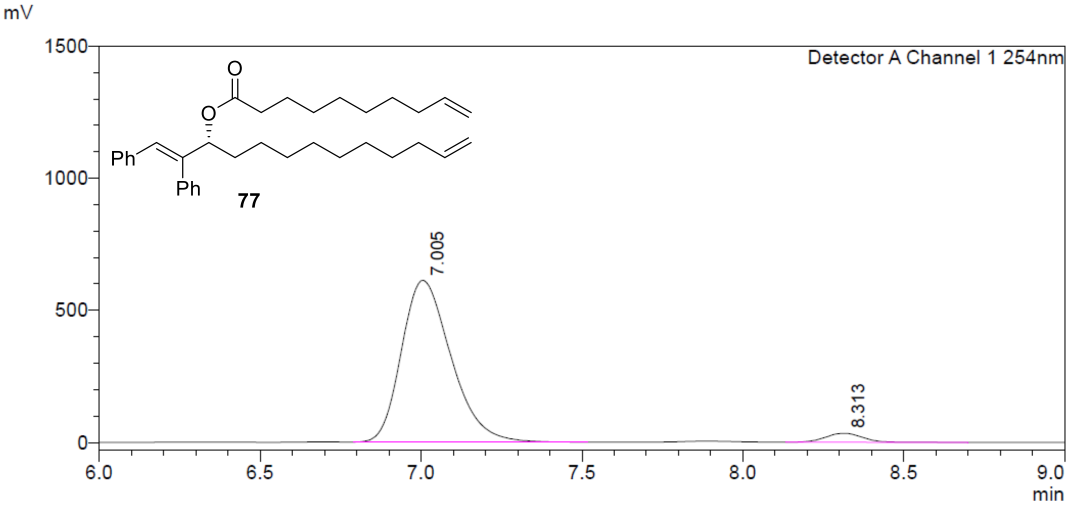

<Peak Table>

Detector A Channel 1 254nm

Peak#	Ret. Time	Height	Height%	Area	Area%
1	7.005	611992	94.741	6570087	96.001
2	8.313	33972	5.259	273715	3.999
Total		645964	100.000	6843802	100.000

Supplementary Figure 454. Scalemic Chromatogram of compound **77**

<Chromatogram>

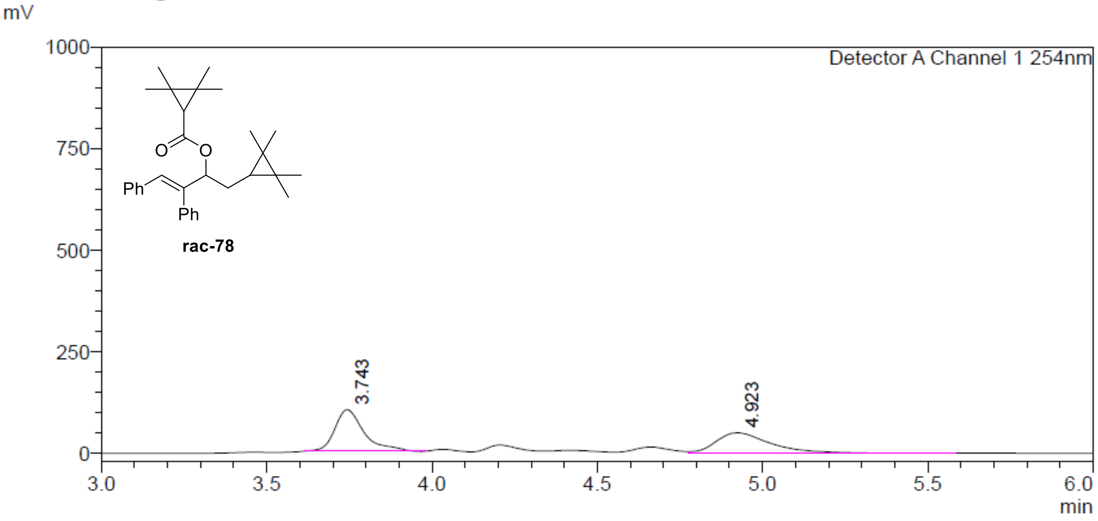

<Peak Table>

Detector A Channel 1 254nm

Peak#	Ret. Time	Height	Height%	Area	Area%
1	3.743	102150	66.844	625996	50.758
2	4.923	50670	33.156	607308	49.242
Total		152820	100.000	1233304	100.000

Supplementary Figure 455. Racemic Chromatogram of compound **78**

<Chromatogram>

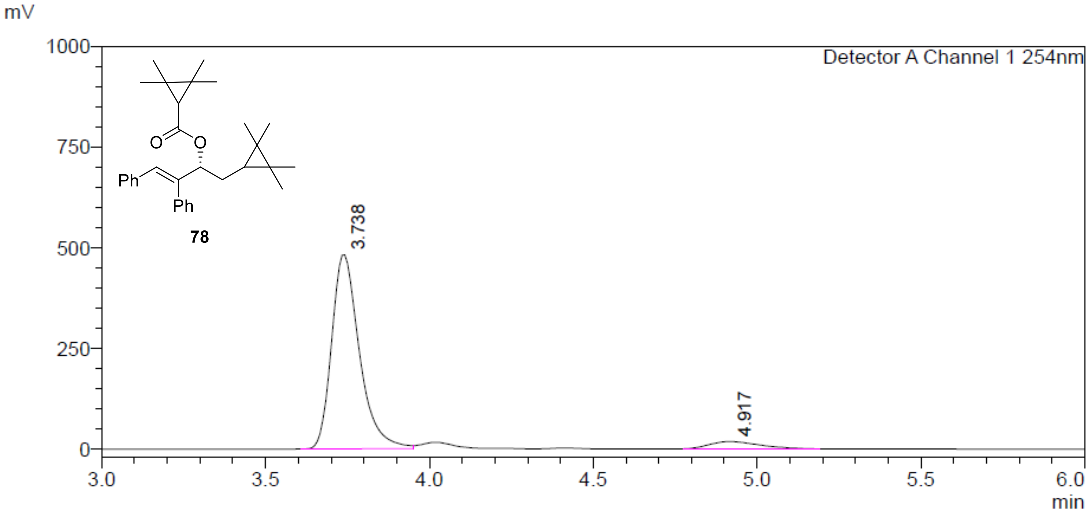

<Peak Table>

Detector A Channel 1 254nm

Peak#	Ret. Time	Height	Height%	Area	Area%
1	3.738	480495	96.398	2858146	93.755
2	4.917	17953	3.602	190366	6.245
Total		498448	100.000	3048512	100.000

Supplementary Figure 456. Scalemic Chromatogram of compound **78**

<Chromatogram>

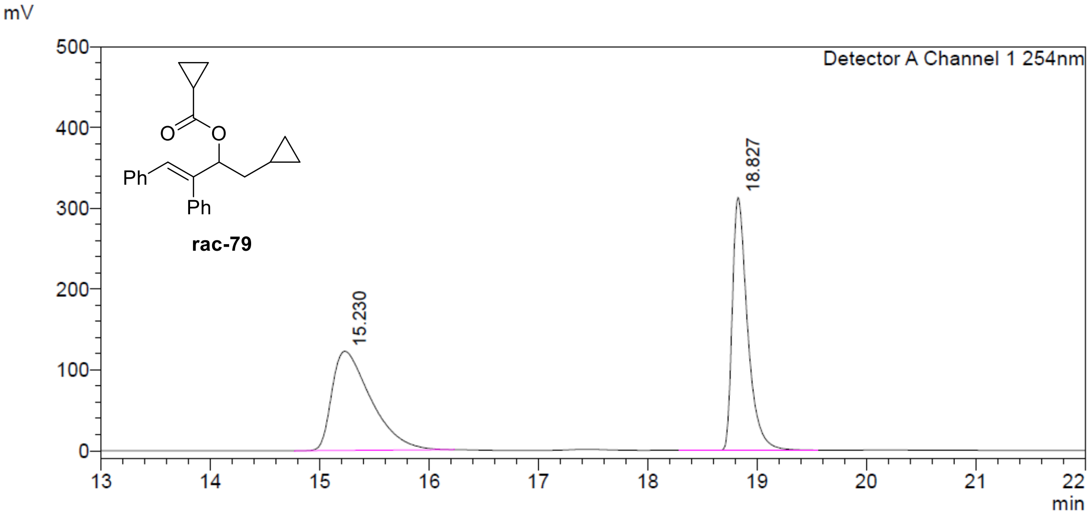

<Peak Table>

Detector A Channel 1 254nm

Peak#	Ret. Time	Height	Height%	Area	Area%
1	15.230	122722	28.184	3045374	50.204
2	18.827	312705	71.816	3020577	49.796
Total		435427	100.000	6065951	100.000

Supplementary Figure 457. Racemic Chromatogram of compound **79**

<Chromatogram>

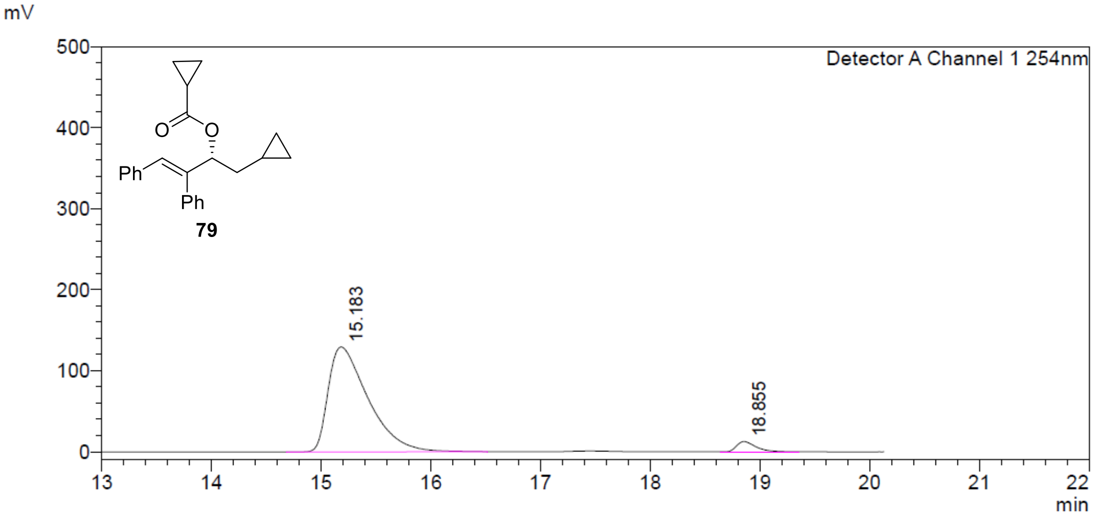

<Peak Table>

Detector A Channel 1 254nm

Peak#	Ret. Time	Height	Height%	Area	Area%
1	15.183	129556	91.173	3274247	95.544
2	18.855	12544	8.827	152688	4.456
Total		142099	100.000	3426935	100.000

Supplementary Figure 458. Scalemic Chromatogram of compound **79**

<Chromatogram>

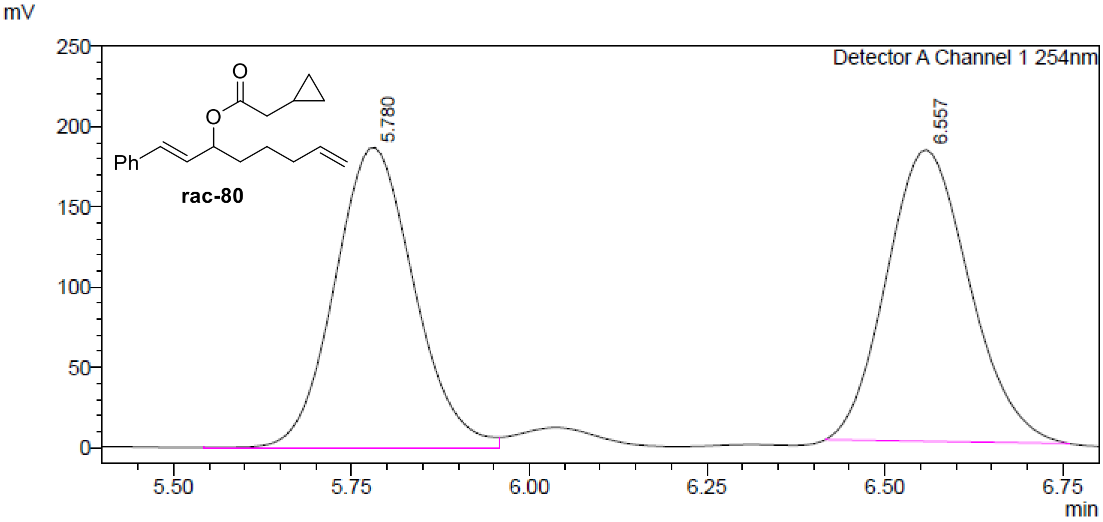

<Peak Table>

Detector A Channel 1 254nm

Peak#	Ret. Time	Height	Height%	Area	Area%
1	5.780	187038	50.717	1453167	49.682
2	6.557	181747	49.283	1471794	50.318
Total		368785	100.000	2924961	100.000

Supplementary Figure 459. Racemic Chromatogram of compound **80**

<Chromatogram>

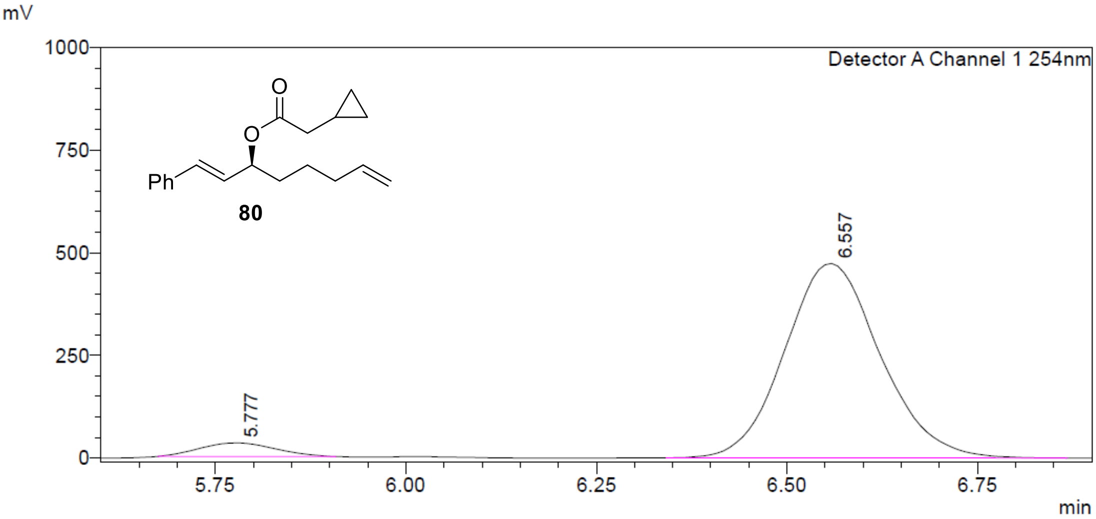

<Peak Table>

Detector A Channel 1 254nm

Peak#	Ret. Time	Height	Height%	Area	Area%
1	5.777	32885	6.501	225628	5.183
2	6.557	472990	93.499	4127560	94.817
Total		505876	100.000	4353189	100.000

Supplementary Figure 460. Scalemic Chromatogram of compound **80**

<Chromatogram>

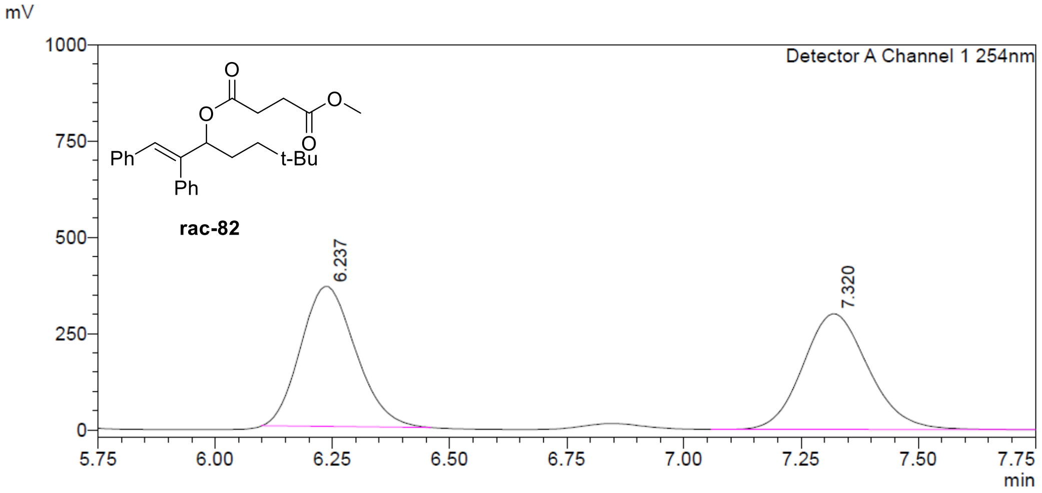

<Peak Table>

Detector A Channel 1 254nm

Peak#	Ret. Time	Height	Height%	Area	Area%
1	6.237	363482	54.760	2970616	50.401
2	7.320	300289	45.240	2923354	49.599
Total		663771	100.000	5893970	100.000

Supplementary Figure 461. Racemic Chromatogram of compound **81**

<Chromatogram>

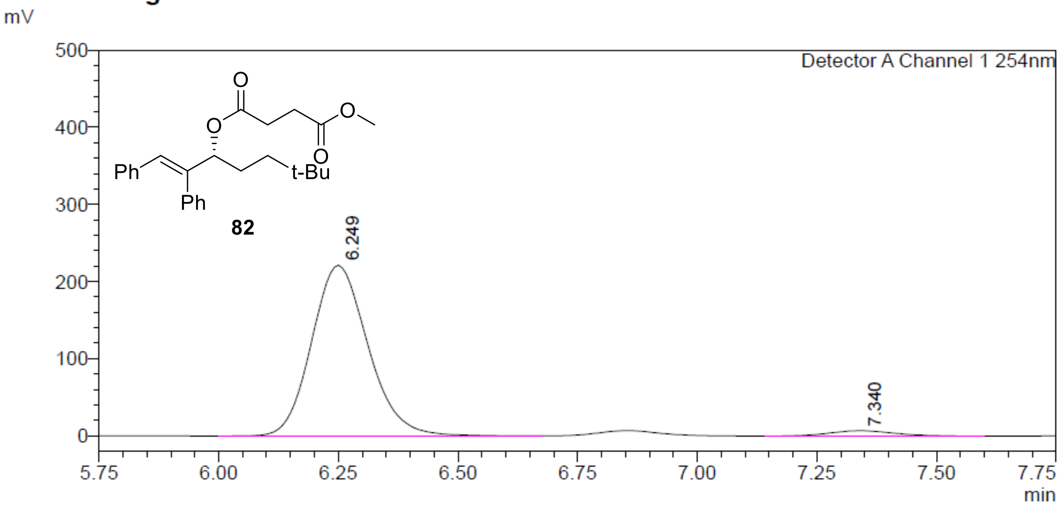

<Peak Table>

Detector A Channel 1 254nm

Peak#	Ret. Time	Height	Height%	Area	Area%
1	6.249	221452	97.013	1821853	96.600
2	7.340	6818	2.987	64124	3.400
Total		228270	100.000	1885978	100.000

Supplementary Figure 462. Scalemic Chromatogram of compound **81**

<Chromatogram>

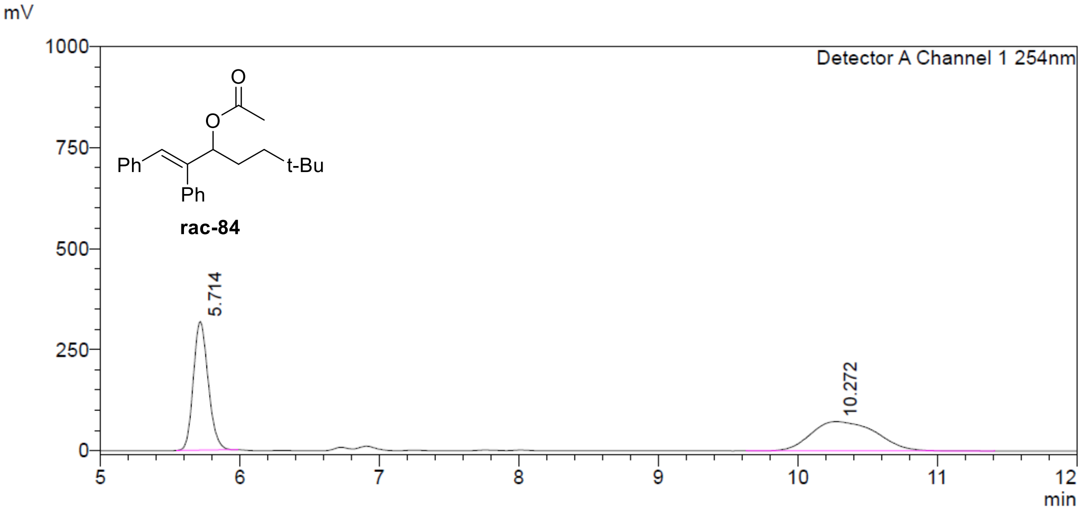

<Peak Table>

Detector A Channel 1 254nm

Peak#	Ret. Time	Height	Height%	Area	Area%
1	5.714	317430	81.457	2351824	50.208
2	10.272	72259	18.543	2332315	49.792
Total		389690	100.000	4684139	100.000

Supplementary Figure 463. Racemic Chromatogram of compound **84**

<Chromatogram>

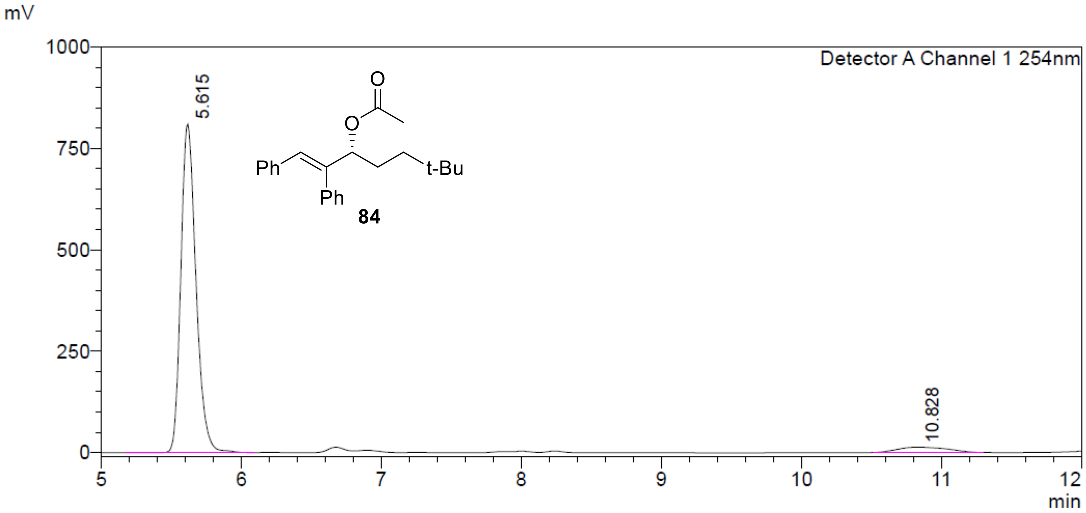

<Peak Table>

Detector A Channel 1 254nm

Peak#	Ret. Time	Height	Height%	Area	Area%
1	5.615	809917	98.421	6083978	95.060
2	10.828	12997	1.579	316189	4.940
Total		822914	100.000	6400167	100.000

Supplementary Figure 464. Scalemic Chromatogram of compound **84**

<Chromatogram>

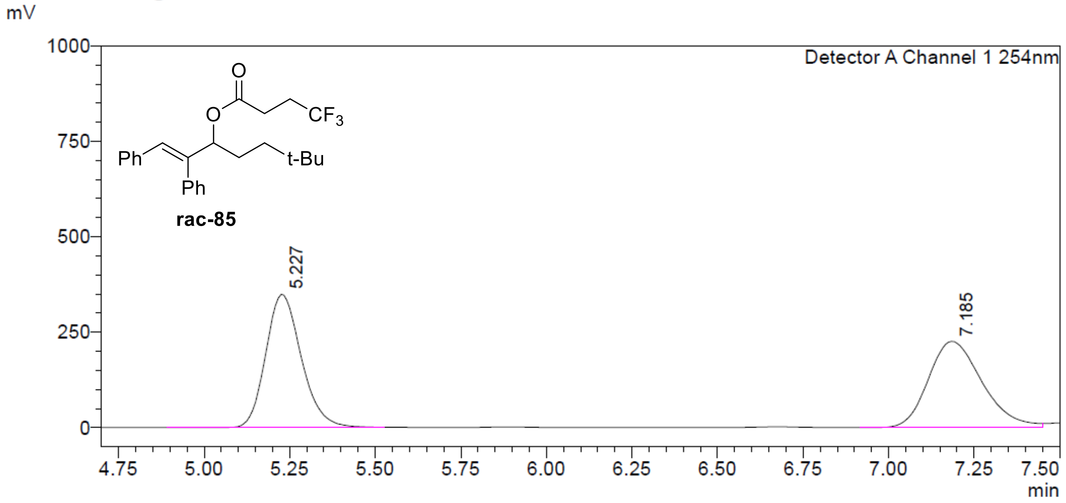

<Peak Table>

Detector A Channel 1 254nm

Peak#	Ret. Time	Height	Height%	Area	Area%
1	5.227	349537	60.709	2540485	50.588
2	7.185	226221	39.291	2481475	49.412
Total		575758	100.000	5021960	100.000

Supplementary Figure 465. Racemic Chromatogram of compound **85**

<Chromatogram>

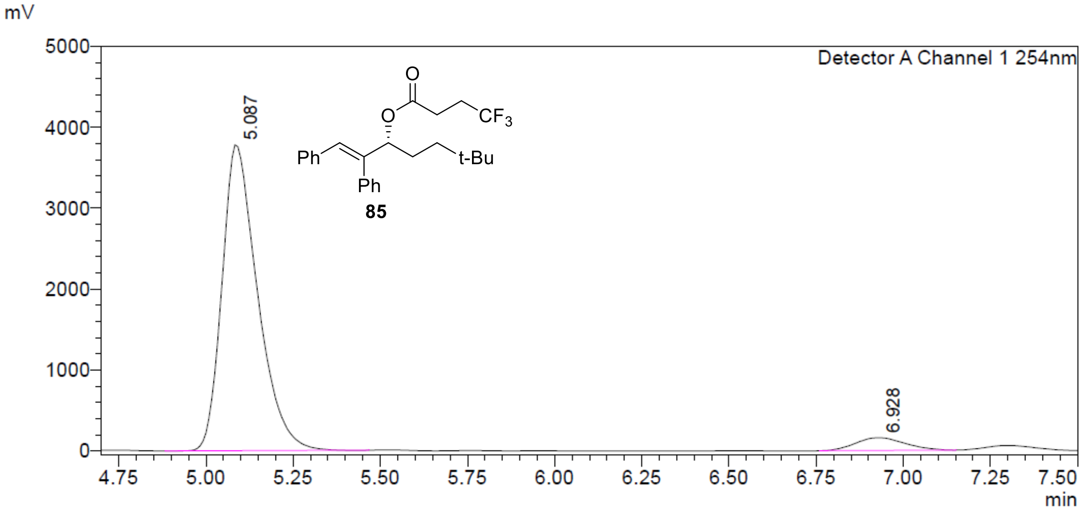

<Peak Table>

Detector A Channel 1 254nm

Peak#	Ret. Time	Height	Height%	Area	Area%
1	5.087	3776015	95.997	26743614	94.520
2	6.928	157459	4.003	1550470	5.480
Total		3933474	100.000	28294084	100.000

Supplementary Figure 466. Scalemic Chromatogram of compound **85**

<Chromatogram>

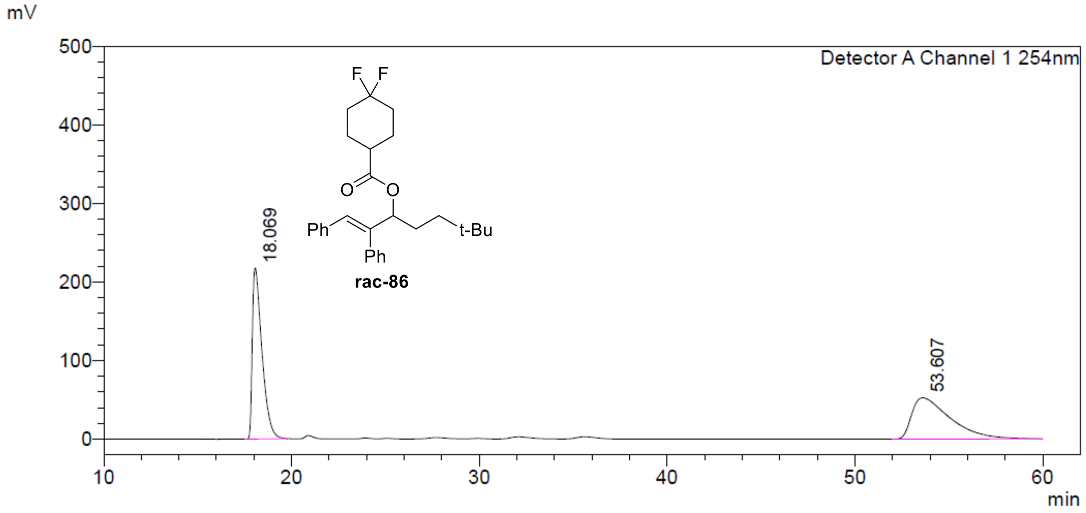

<Peak Table>

Detector A Channel 1 254nm

Peak#	Ret. Time	Height	Height%	Area	Area%
1	18.069	217695	80.480	7776648	50.770
2	53.607	52802	19.520	7540787	49.230
Total		270497	100.000	15317435	100.000

Supplementary Figure 467. Racemic Chromatogram of compound **86**

<Chromatogram>

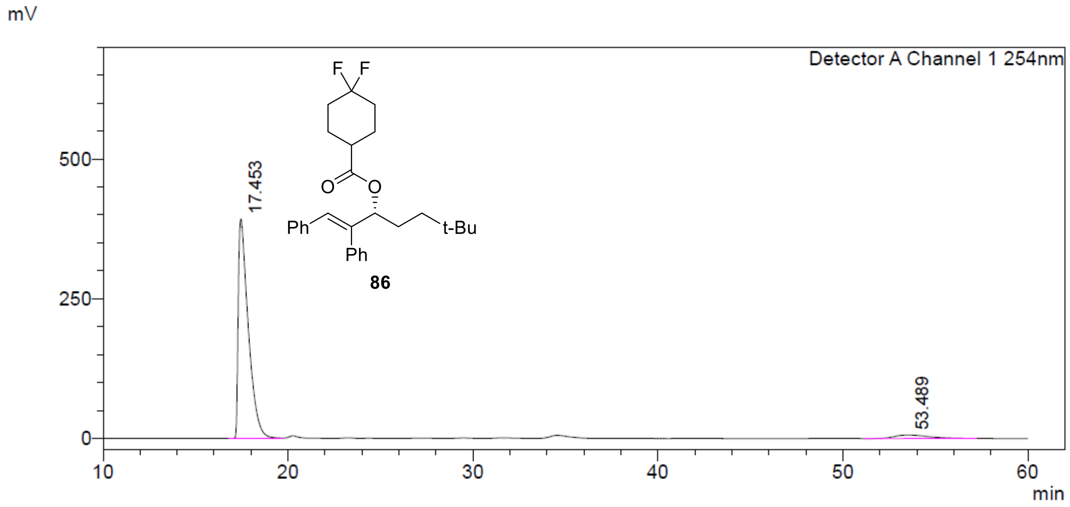

<Peak Table>

Detector A Channel 1 254nm

Peak#	Ret. Time	Height	Height%	Area	Area%
1	17.453	392661	98.527	14831480	95.023
2	53.489	5870	1.473	776846	4.977
Total		398531	100.000	15608326	100.000

Supplementary Figure 468. Scalemic Chromatogram of compound **86**

<Chromatogram>

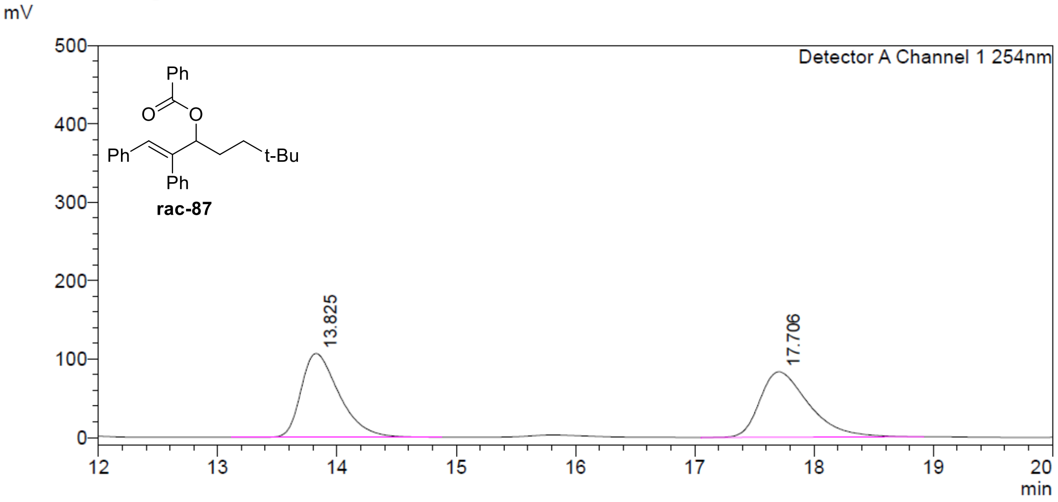

<Peak Table>

Detector A Channel 1 254nm

Peak#	Ret. Time	Height	Height%	Area	Area%
1	13.825	106949	56.198	2374243	50.070
2	17.706	83358	43.802	2367607	49.930
Total		190307	100.000	4741850	100.000

Supplementary Figure 469. Racemic Chromatogram of compound **87**

<Chromatogram>

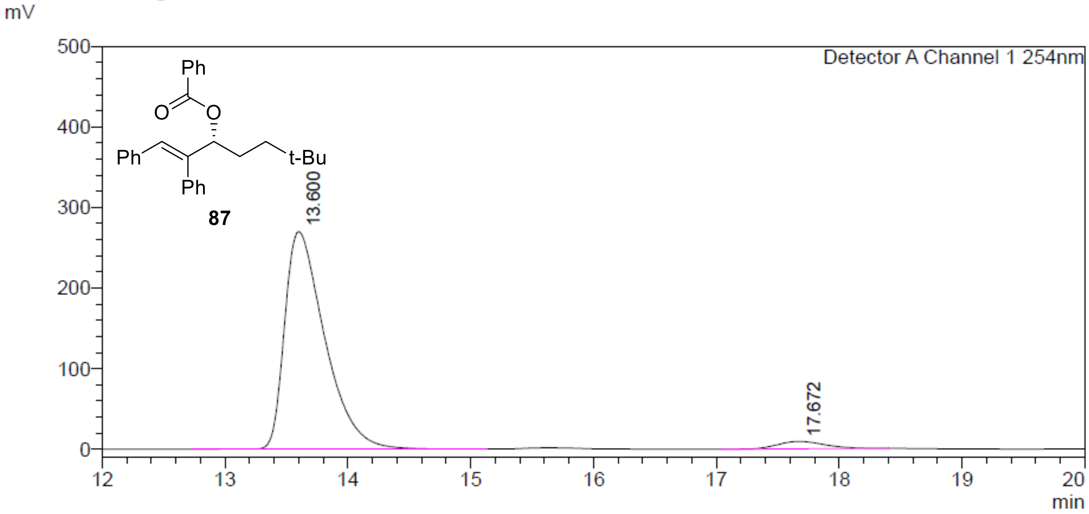

<Peak Table>

Detector A Channel 1 254nm

Peak#	Ret. Time	Height	Height%	Area	Area%
1	13.600	270129	96.675	6149669	96.136
2	17.672	9291	3.325	247167	3.864
Total		279420	100.000	6396836	100.000

Supplementary Figure 470. Scalemic Chromatogram of compound **87**

<Chromatogram>

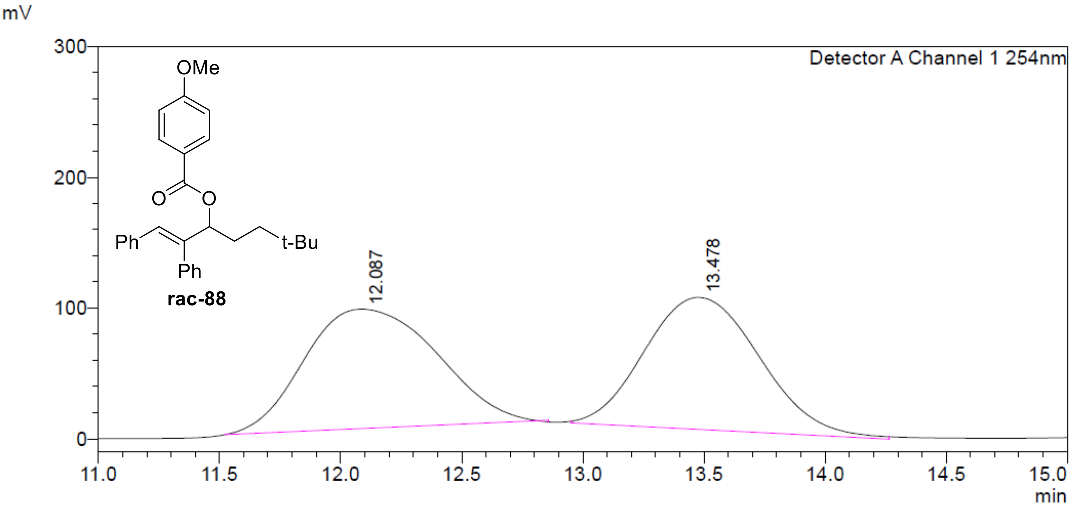

<Peak Table>

Detector A Channel 1 254nm

Peak#	Ret. Time	Height	Height%	Area	Area%
1	12.087	91303	47.509	3465403	50.998
2	13.478	100879	52.491	3329729	49.002
Total		192183	100.000	6795131	100.000

Supplementary Figure 471. Racemic Chromatogram of compound **88**

<Chromatogram>

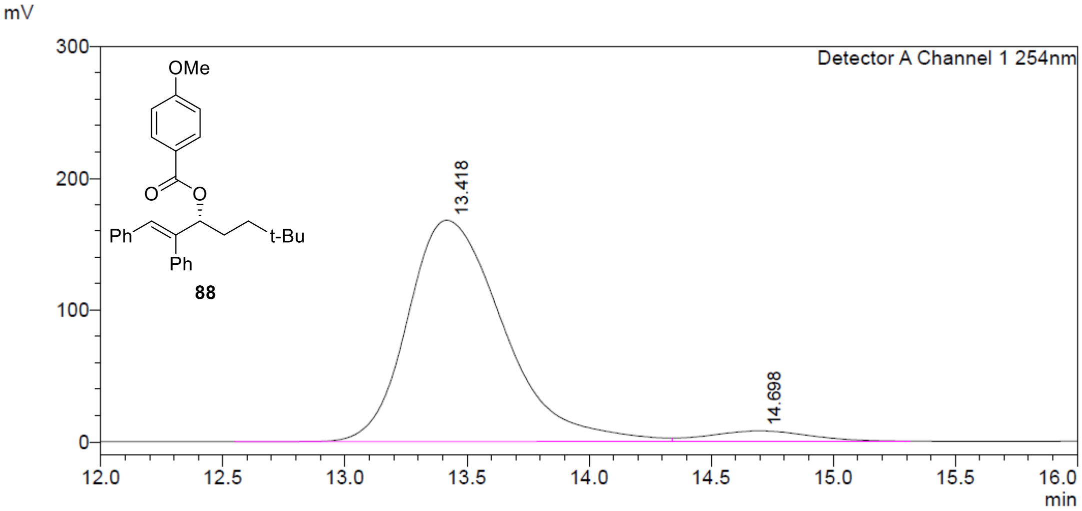

<Peak Table>

Detector A Channel 1 254nm

Peak#	Ret. Time	Height	Height%	Area	Area%
1	13.418	167982	95.420	4598431	95.186
2	14.698	8063	4.580	232571	4.814
Total		176044	100.000	4831002	100.000

Supplementary Figure 472. Scalemic Chromatogram of compound **88**

<Chromatogram>

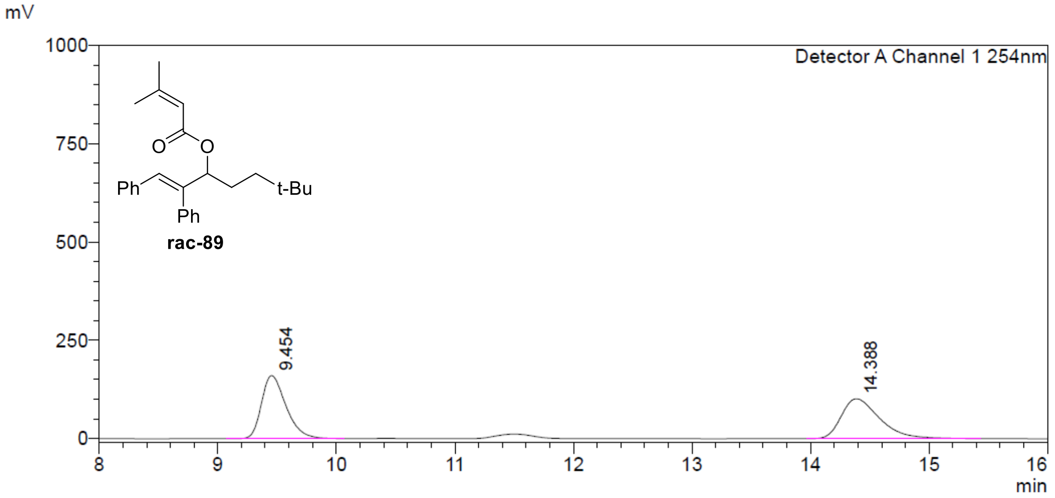

<Peak Table>

Detector A Channel 1 254nm

Peak#	Ret. Time	Height	Height%	Area	Area%
1	9.454	159987	61.279	2294304	50.162
2	14.388	101093	38.721	2279482	49.838
Total		261080	100.000	4573786	100.000

Supplementary Figure 473. Racemic Chromatogram of compound **89**

<Chromatogram>

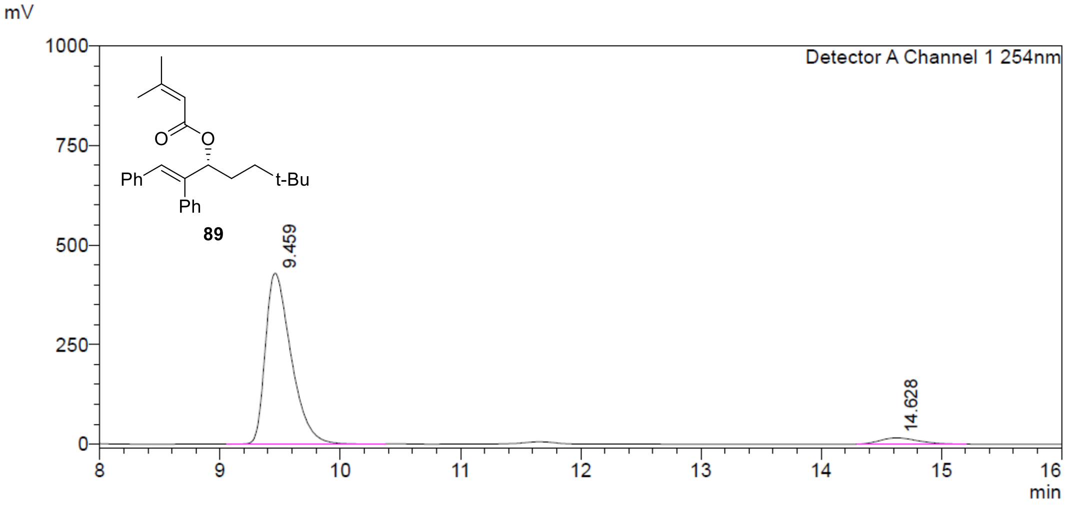

<Peak Table>

Detector A Channel 1 254nm

Peak#	Ret. Time	Height	Height%	Area	Area%
1	9.459	428289	96.478	6331952	94.931
2	14.628	15635	3.522	338095	5.069
Total		443924	100.000	6670047	100.000

Supplementary Figure 474. Scalemic Chromatogram of compound **89**

<Chromatogram>

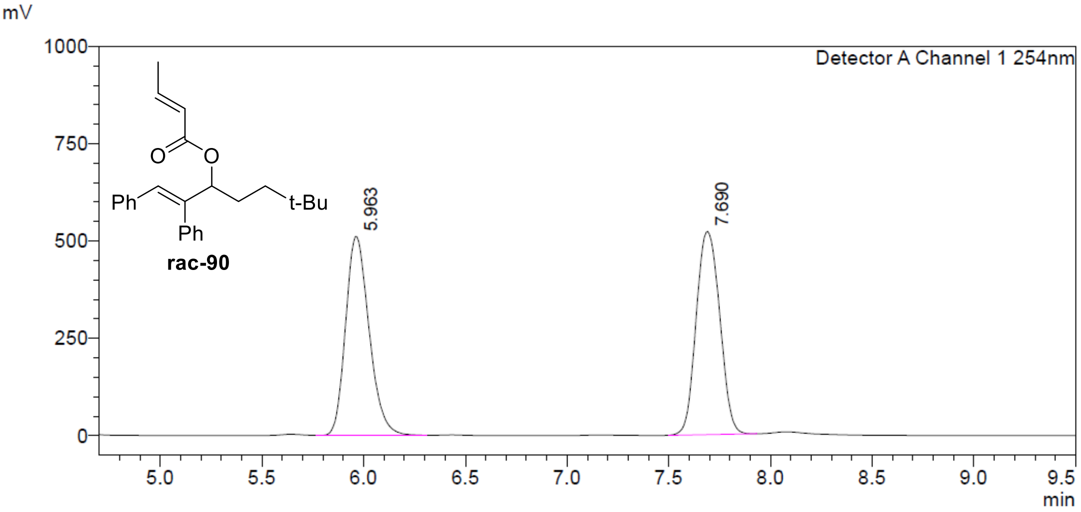

<Peak Table>

Detector A Channel 1 254nm

Peak#	Ret. Time	Height	Height%	Area	Area%
1	5.963	510379	49.447	4118217	49.402
2	7.690	521787	50.553	4217901	50.598
Total		1032166	100.000	8336118	100.000

Supplementary Figure 475. Racemic Chromatogram of compound **90**

<Chromatogram>

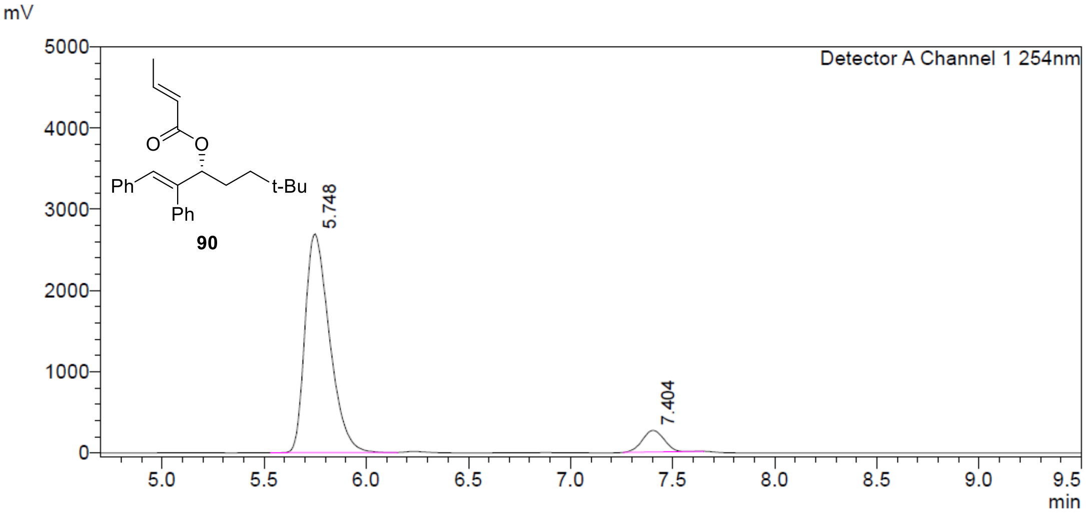

<Peak Table>

Detector A Channel 1 254nm

Peak#	Ret. Time	Height	Height%	Area	Area%
1	5.748	2691514	91.041	22495735	91.967
2	7.404	264860	8.959	1964811	8.033
Total		2956374	100.000	24460546	100.000

Supplementary Figure 476. Scalemic Chromatogram of compound **90**

<Chromatogram>

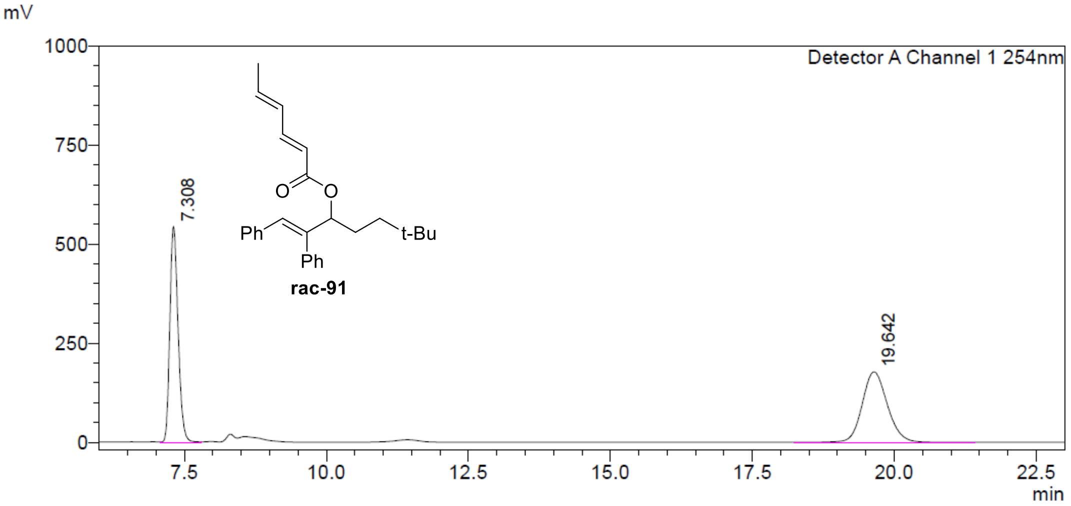

<Peak Table>

Detector A Channel 1 254nm

Peak#	Ret. Time	Height	Height%	Area	Area%
1	7.308	543753	75.349	5478062	49.992
2	19.642	177895	24.651	5479747	50.008
Total		721648	100.000	10957809	100.000

Supplementary Figure 477. Racemic Chromatogram of compound **91**

<Chromatogram>

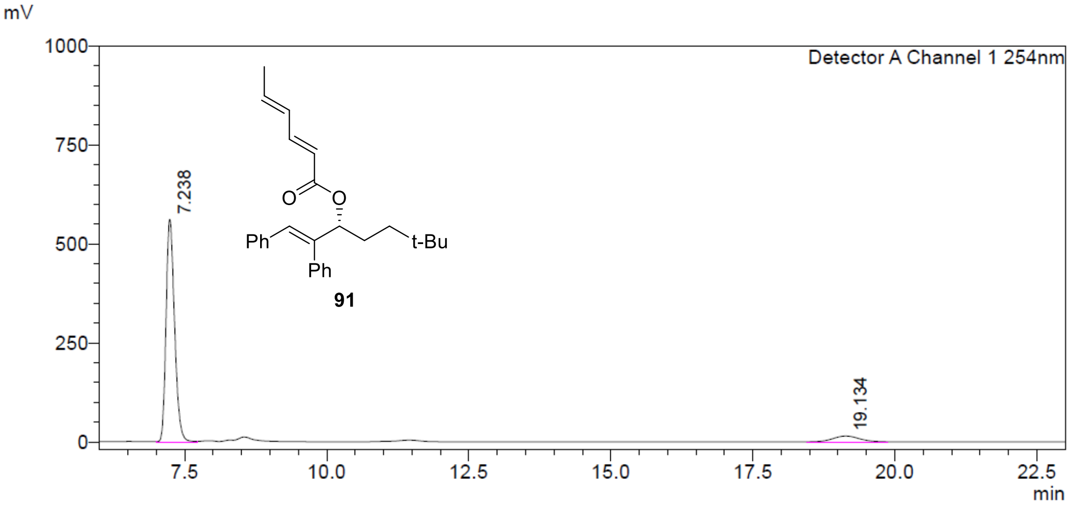

<Peak Table>

Detector A Channel 1 254nm

Peak#	Ret. Time	Height	Height%	Area	Area%
1	7.238	561745	97.515	5852312	92.695
2	19.134	14317	2.485	461236	7.305
Total		576061	100.000	6313548	100.000

Supplementary Figure 478. Scalemic Chromatogram of compound **91**

<Chromatogram>

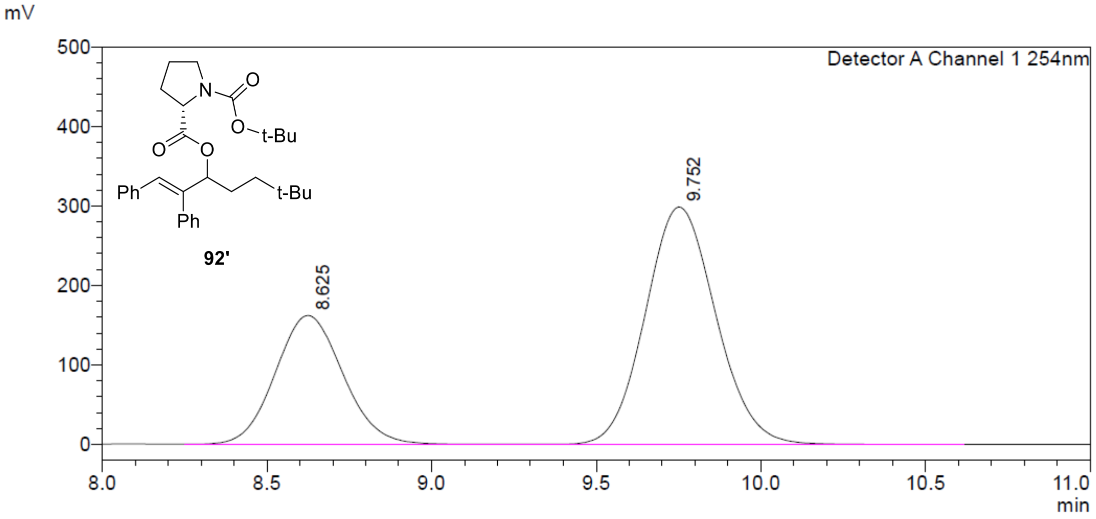

<Peak Table>

Detector A Channel 1 254nm

Peak#	Ret. Time	Height	Height%	Area	Area%
1	8.625	162380	35.215	2337307	34.361
2	9.752	298734	64.785	4464996	65.639
Total		461114	100.000	6802303	100.000

Supplementary Figure 479. Racemic Chromatogram of compound **92**

<Chromatogram>

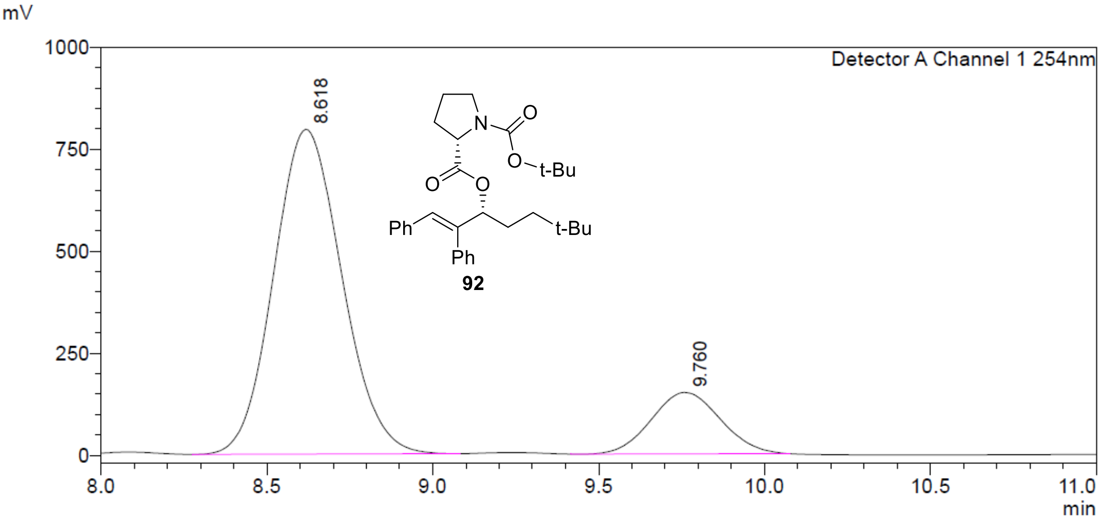

<Peak Table>

Detector A Channel 1 254nm

Peak#	Ret. Time	Height	Height%	Area	Area%
1	8.618	796173	84.088	11383091	83.888
2	9.760	150663	15.912	2186275	16.112
Total		946835	100.000	13569367	100.000

Supplementary Figure 480. Scalemic Chromatogram of compound **92**

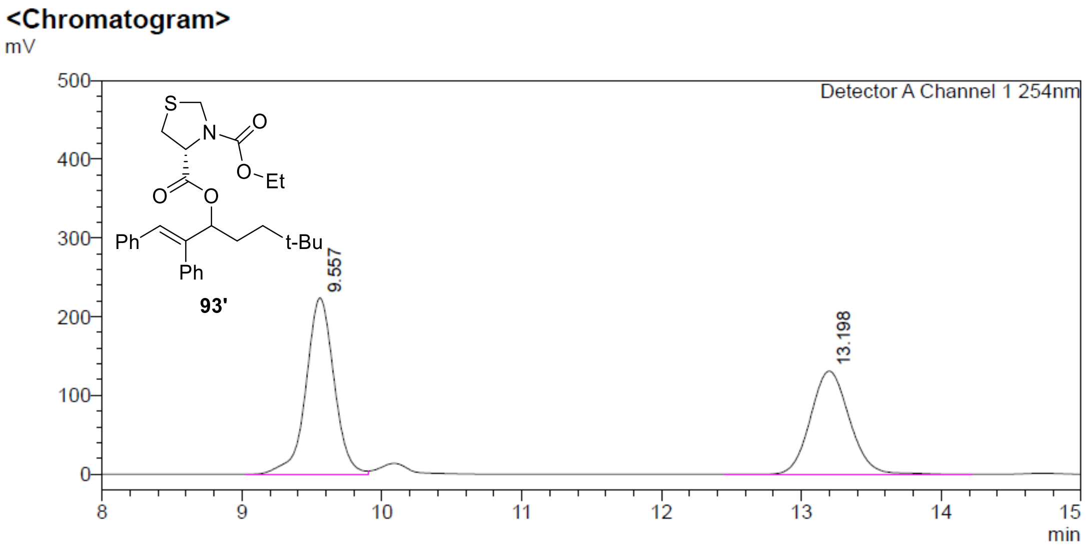

<Peak Table>

Detector A Channel 1 254nm

Peak#	Ret. Time	Height	Height%	Area	Area%
1	9.557	224057	63.089	3131901	55.214
2	13.198	131088	36.911	2540442	44.786
Total		355145	100.000	5672344	100.000

Supplementary Figure 481. Racemic Chromatogram of compound **93**

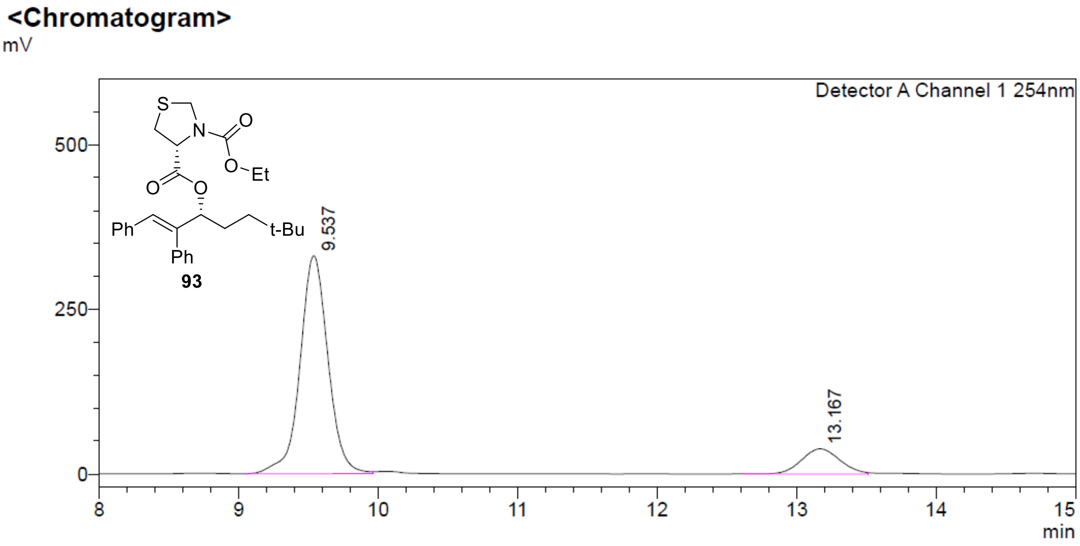

<Peak Table>

Detector A Channel 1 254nm

Peak#	Ret. Time	Height	Height%	Area	Area%
1	9.537	330777	89.651	4616646	86.362
2	13.167	38183	10.349	729044	13.638
Total		368960	100.000	5345691	100.000

Supplementary Figure 482. Scalemic Chromatogram of compound **93**

<Chromatogram>

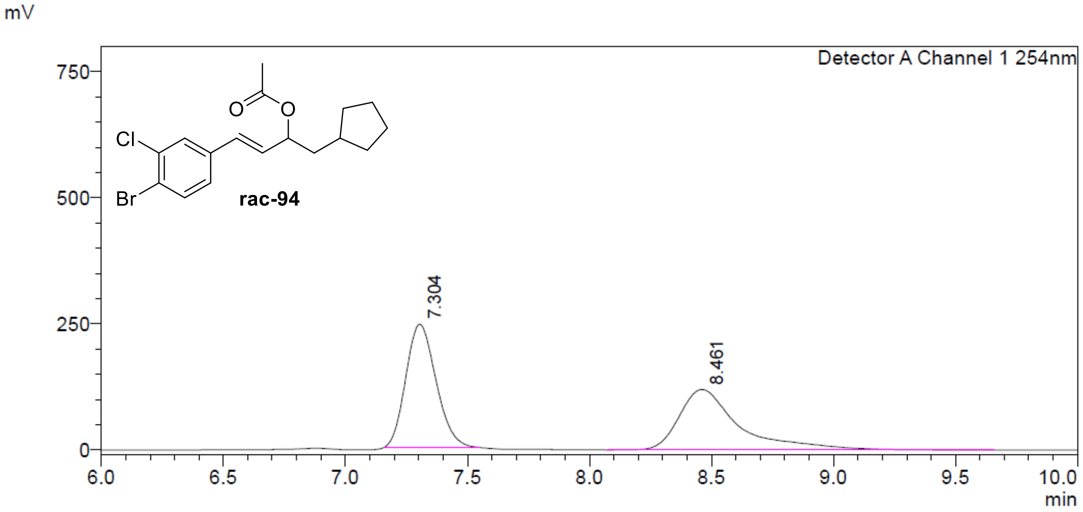

<Peak Table>

Detector A Channel 1 254nm

Peak#	Ret. Time	Height	Height%	Area	Area%
1	7.304	244030	67.098	2111339	50.934
2	8.461	119661	32.902	2033880	49.066
Total		363691	100.000	4145218	100.000

Supplementary Figure 483. Racemic Chromatogram of compound **94**

<Chromatogram>

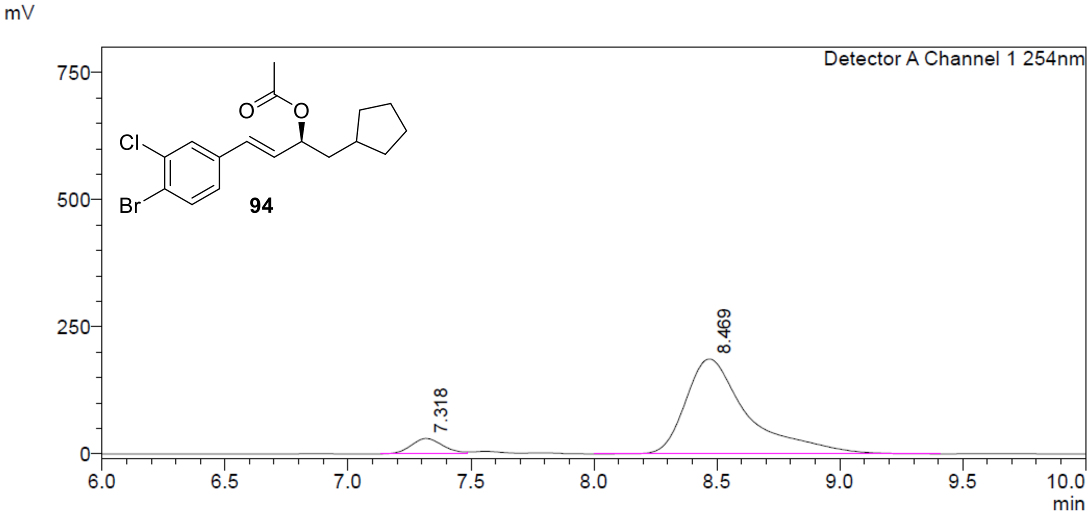

<Peak Table>

Detector A Channel 1 254nm

Peak#	Ret. Time	Height	Height%	Area	Area%
1	7.318	30063	13.877	269769	7.588
2	8.469	186585	86.123	3285550	92.412
Total		216649	100.000	3555319	100.000

Supplementary Figure 484. Scalemic Chromatogram of compound **94**

<Chromatogram>

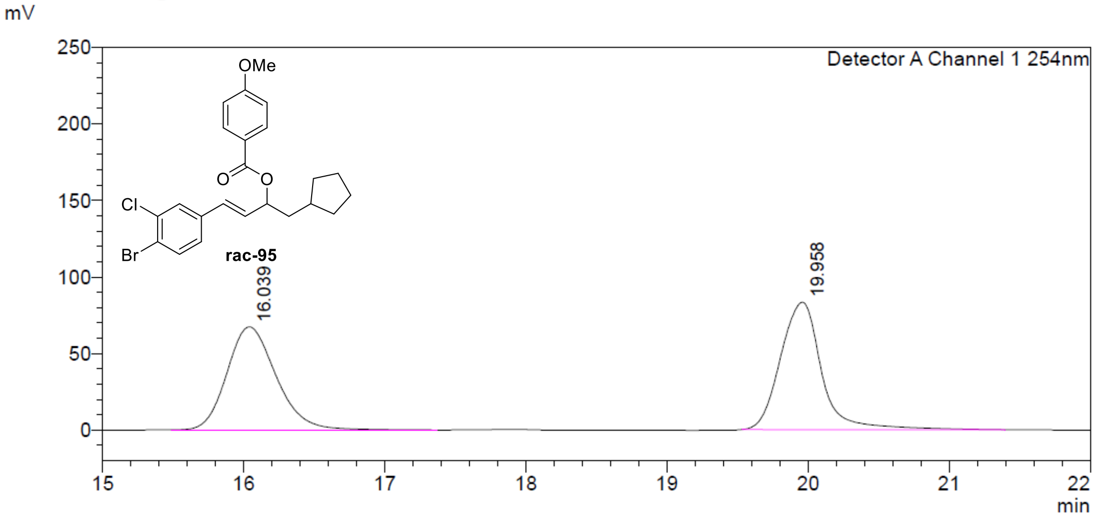

<Peak Table>

Detector A Channel 1 254nm

Peak#	Ret. Time	Height	Height%	Area	Area%
1	16.039	67477	44.768	1652635	49.454
2	19.958	83248	55.232	1689102	50.546
Total		150725	100.000	3341736	100.000

Supplementary Figure 485. Racemic Chromatogram of compound **95**

<Chromatogram>

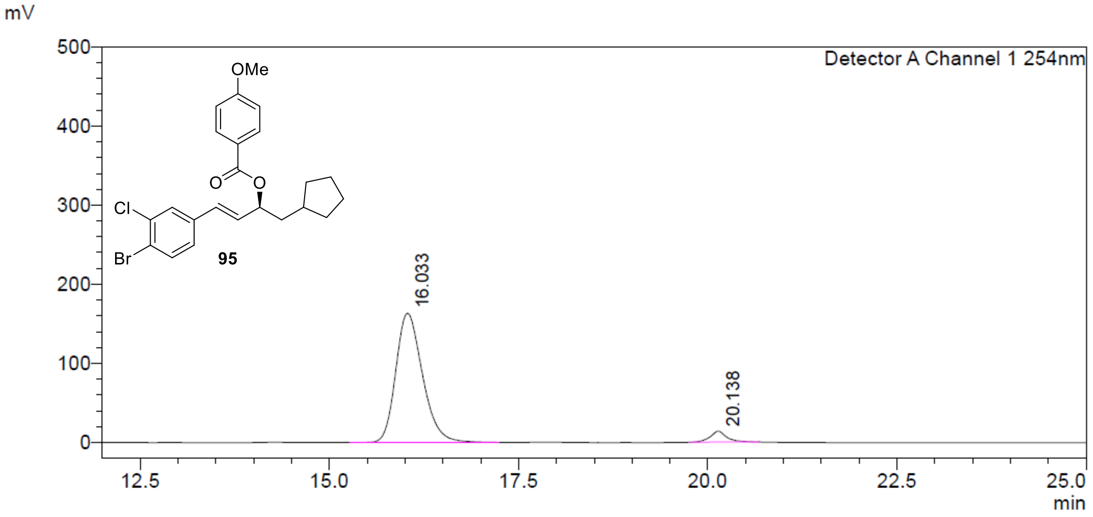

<Peak Table>

Detector A Channel 1 254nm

Peak#	Ret. Time	Height	Height%	Area	Area%
1	16.033	163147	92.254	4001201	94.980
2	20.138	13698	7.746	211482	5.020
Total		176845	100.000	4212683	100.000

Supplementary Figure 486. Scalemic Chromatogram of compound **95**

<Chromatogram>

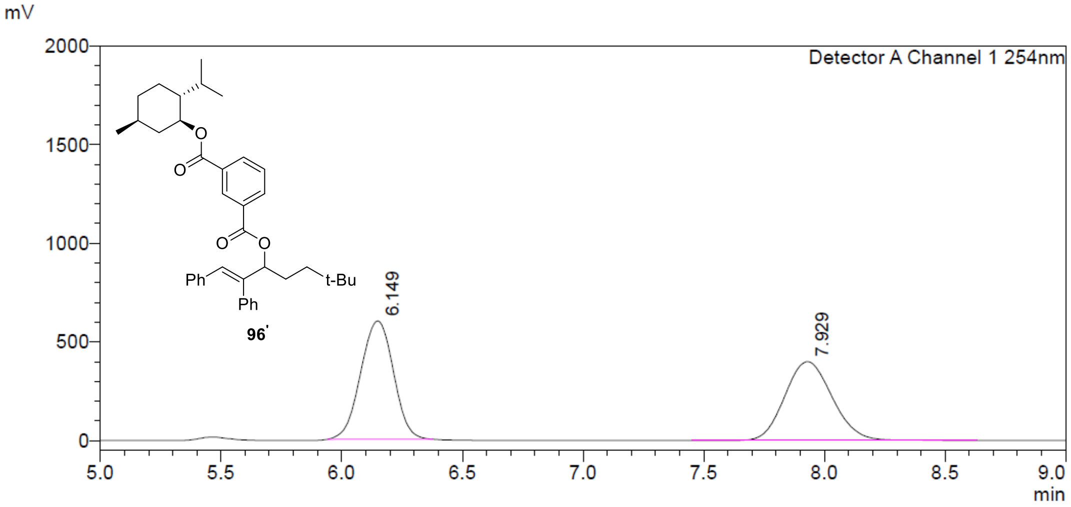

<Peak Table>

Detector A Channel 1 254nm

Peak#	Ret. Time	Height	Height%	Area	Area%
1	6.149	599810	60.040	5666177	50.757
2	7.929	399206	39.960	5497158	49.243
Total		999016	100.000	11163335	100.000

Supplementary Figure 487. Racemic Chromatogram of compound **96**

<Chromatogram>

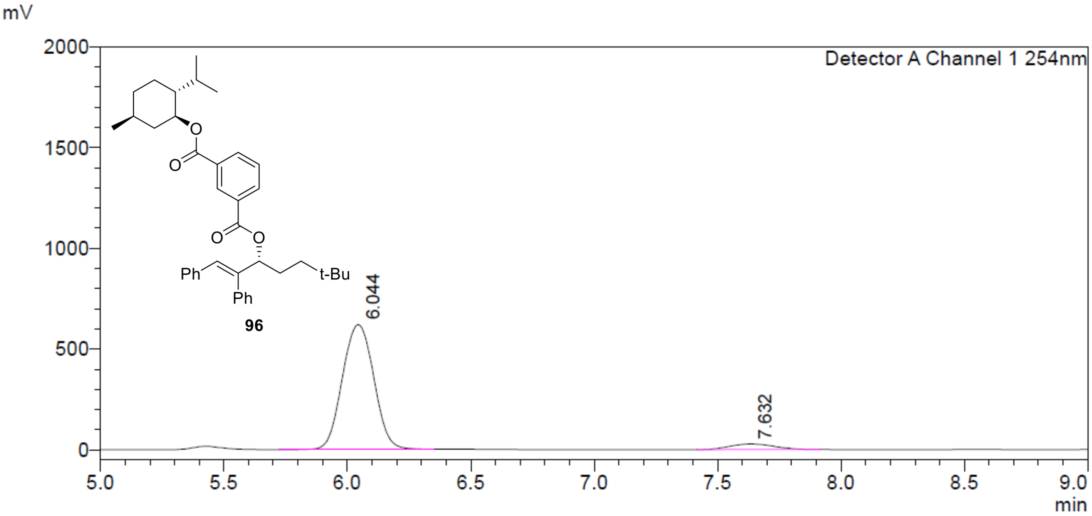

<Peak Table>

Detector A Channel 1 254nm

Peak#	Ret. Time	Height	Height%	Area	Area%
1	6.044	620647	95.724	5532328	94.087
2	7.632	27727	4.276	347658	5.913
Total		648374	100.000	5879986	100.000

Supplementary Figure 488. Scalemic Chromatogram of compound **96**

<Chromatogram>

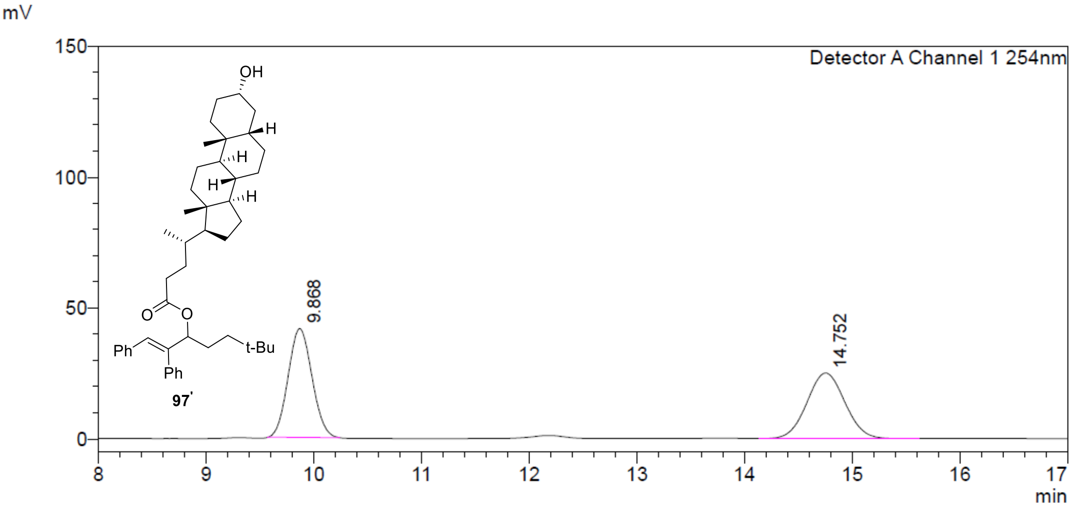

<Peak Table>

Detector A Channel 1 254nm

Peak#	Ret. Time	Height	Height%	Area	Area%
1	9.868	41571	62.347	640768	50.913
2	14.752	25106	37.653	617781	49.087
Total		66676	100.000	1258549	100.000

Supplementary Figure 489. Racemic Chromatogram of compound **97**

<Chromatogram>

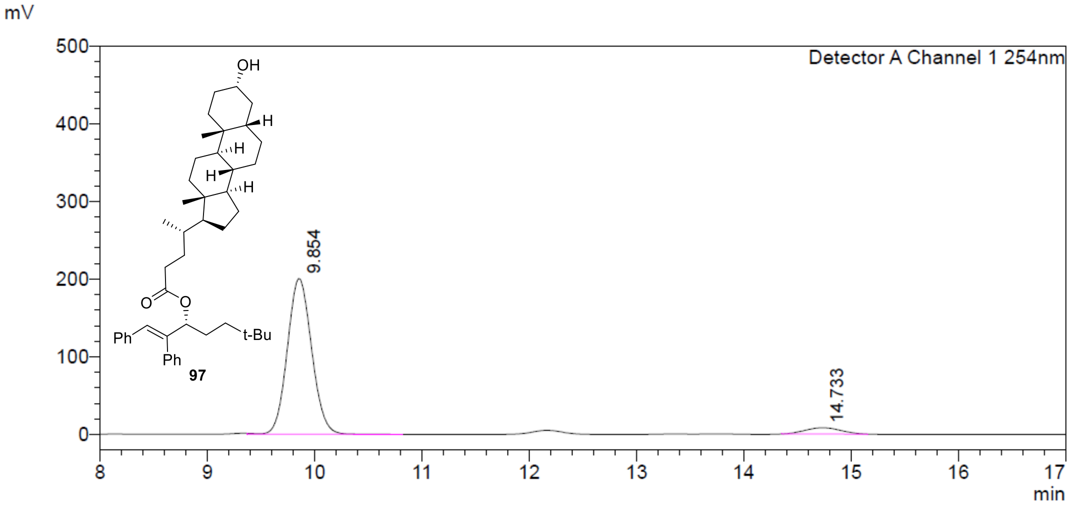

<Peak Table>

Detector A Channel 1 254nm

Peak#	Ret. Time	Height	Height%	Area	Area%
1	9.854	200378	96.128	3166700	94.504
2	14.733	8072	3.872	184173	5.496
Total		208451	100.000	3350873	100.000

Supplementary Figure 490. Scalemic Chromatogram of compound **97**

<Chromatogram>

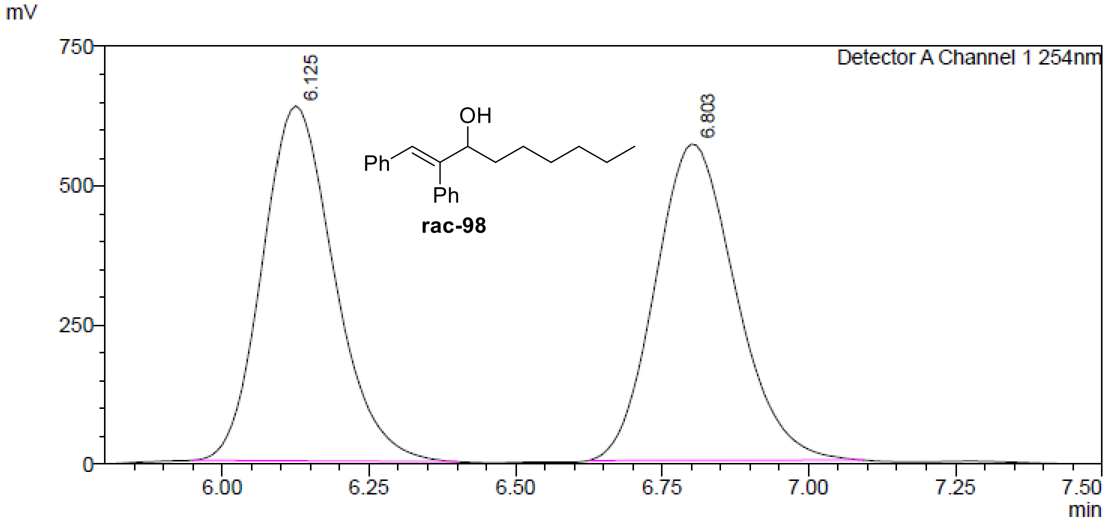

<Peak Table>

Detector A Channel 1 254nm

Peak#	Ret. Time	Height	Height%	Area	Area%
1	6.125	637163	52.895	5416876	49.936
2	6.803	567423	47.105	5430798	50.064
Total		1204586	100.000	10847674	100.000

Supplementary Figure 491. Racemic Chromatogram of compound **98**

<Chromatogram>

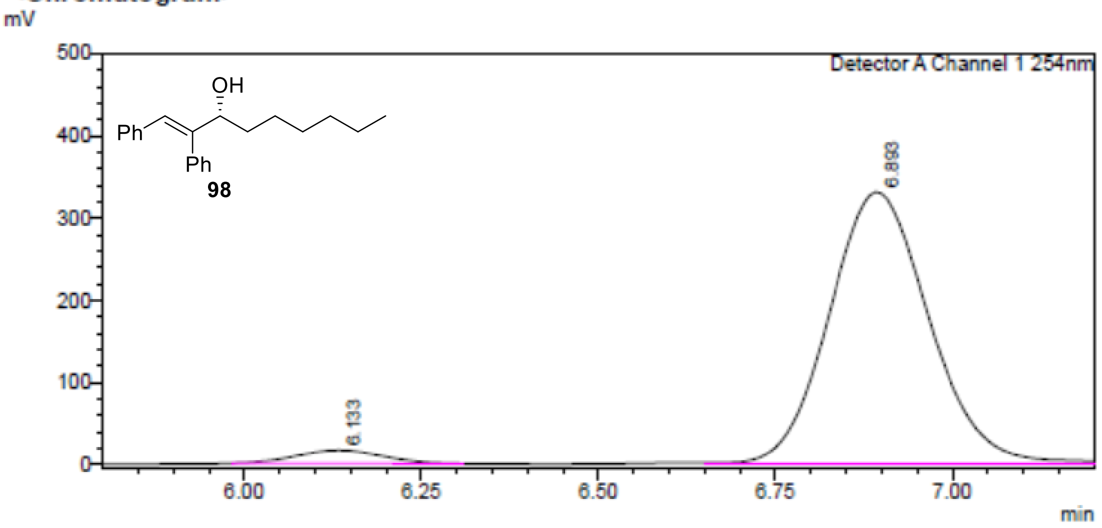

<Peak Table>

Detector A Channel 1 254nm

Peak#	Ret. Time	Height	Height%	Area	Area%
1	6.133	16224	4.691	137084	4.098
2	6.893	329659	95.309	3208425	95.902
Total		345883	100.000	3345509	100.000

Supplementary Figure 492. Scalemic Chromatogram of compound **98**

<Chromatogram>

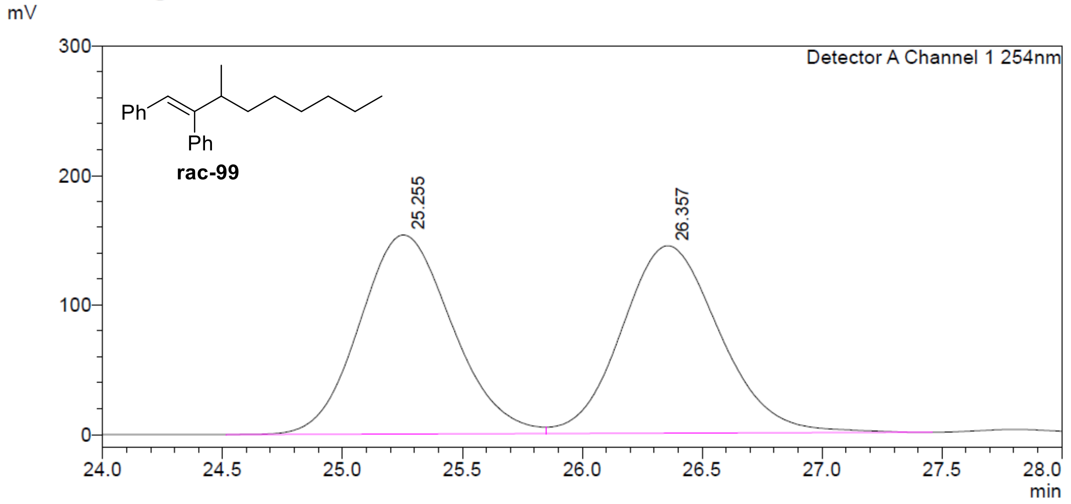

<Peak Table>

Detector A Channel 1 254nm

Peak#	Ret. Time	Height	Height%	Area	Area%
1	25.255	153696	51.514	4137725	50.089
2	26.357	144664	48.486	4123079	49.911
Total		298360	100.000	8260804	100.000

Supplementary Figure 493. Racemic Chromatogram of compound **99**

<Chromatogram>

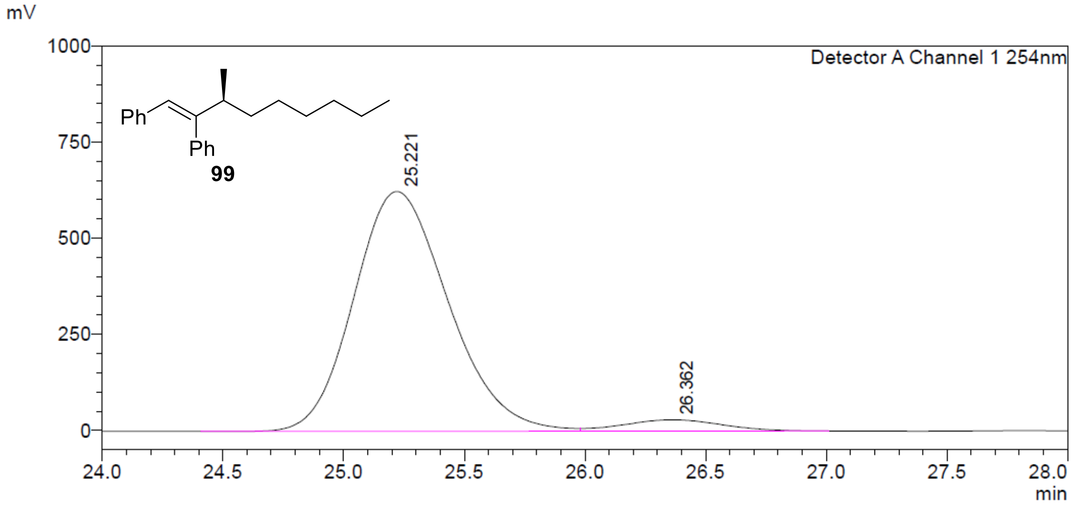

<Peak Table>

Detector A Channel 1 254nm

Peak#	Ret. Time	Height	Height%	Area	Area%
1	25.221	623600	95.430	17039104	95.276
2	26.362	29865	4.570	844887	4.724
Total		653465	100.000	17883991	100.000

Supplementary Figure 494. Scalemic Chromatogram of compound **99**

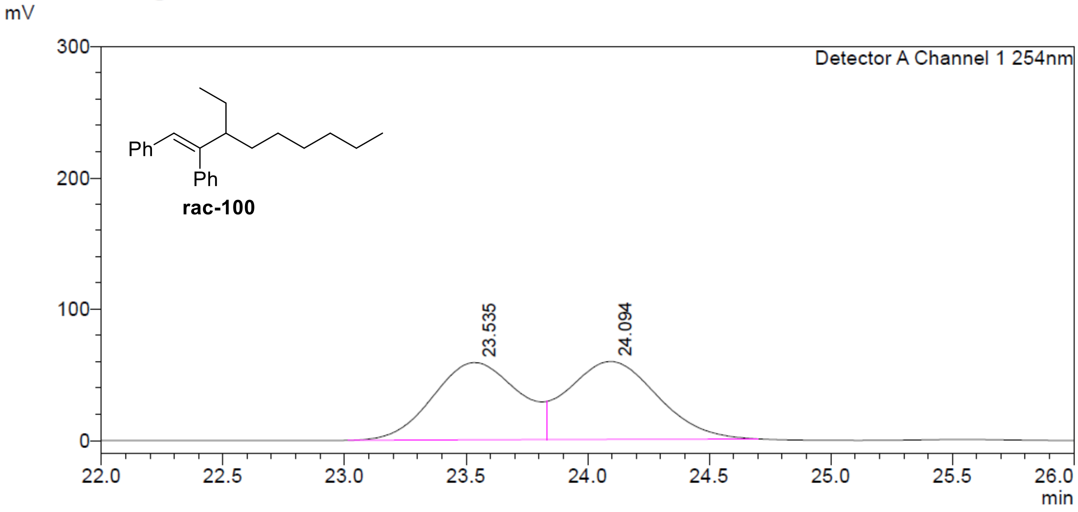

<Peak Table>

Detector A Channel 1 254nm

Peak#	Ret. Time	Height	Height%	Area	Area%
1	23.535	58924	49.795	1467531	49.287
2	24.094	59408	50.205	1510012	50.713
Total		118332	100.000	2977543	100.000

Supplementary Figure 495. Racemic Chromatogram of compound **100**

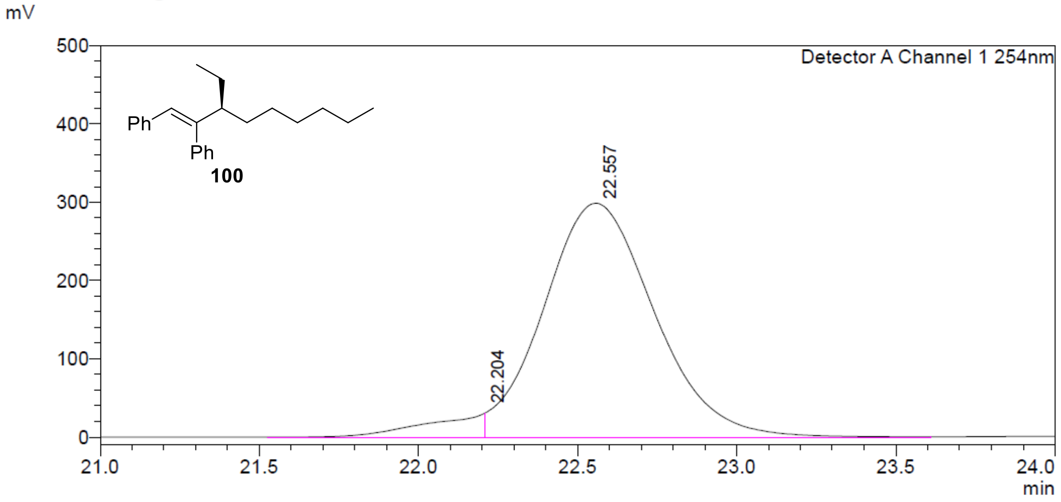

<Peak Table>

Detector A Channel 1 254nm

Peak#	Ret. Time	Height	Height%	Area	Area%
1	22.204	28888	8.817	382384	5.023
2	22.557	298759	91.183	7229579	94.977
Total		327647	100.000	7611963	100.000

Supplementary Figure 496. Scalemic Chromatogram of compound **100**

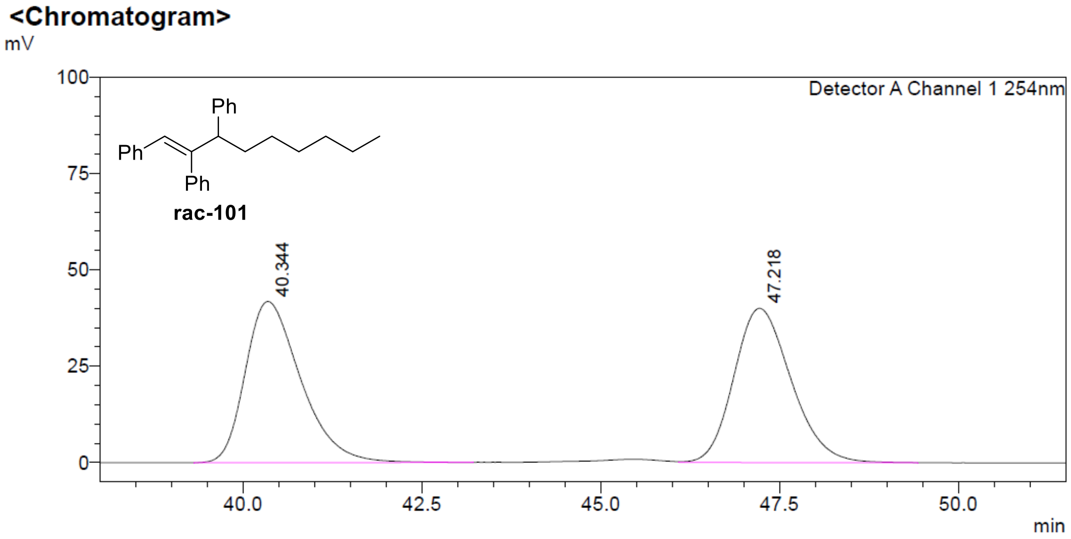

<Peak Table>

Detector A Channel 1 254nm

Peak#	Ret. Time	Height	Height%	Area	Area%
1	40.344	41820	51.099	2254737	50.265
2	47.218	40021	48.901	2230994	49.735
Total		81841	100.000	4485731	100.000

Supplementary Figure 497. Racemic Chromatogram of compound **101**

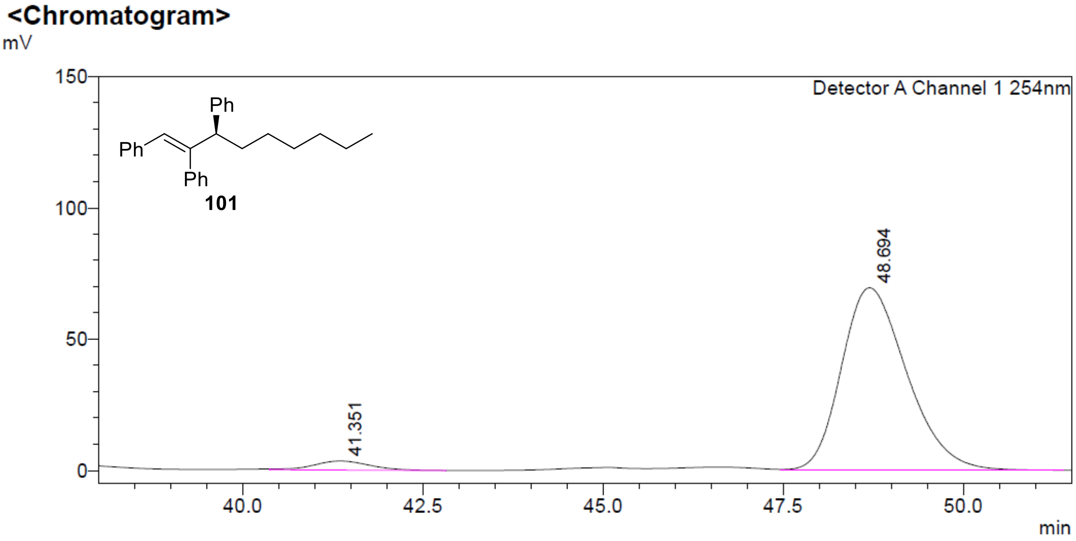

<Peak Table>

Detector A Channel 1 254nm

Peak#	Ret. Time	Height	Height%	Area	Area%
1	41.351	3458	4.740	187165	4.077
2	48.694	69496	95.260	4403203	95.923
Total		72954	100.000	4590369	100.000

Supplementary Figure 498. Scalemic Chromatogram of compound **101**

<Chromatogram>

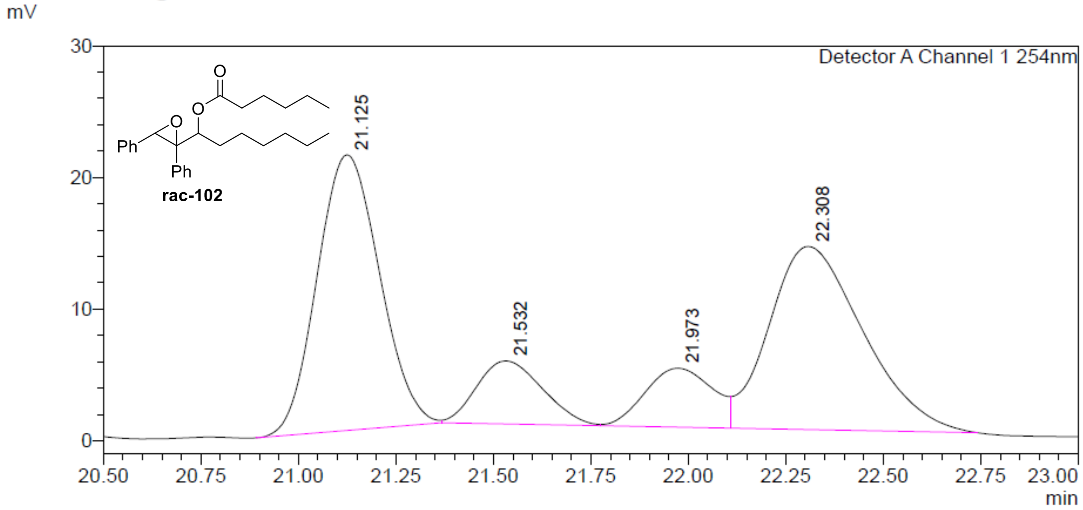

<Peak Table>

Detector A Channel 1 254nm

Peak#	Ret. Time	Height	Height%	Area	Area%
1	21.125	20955	47.471	232918	40.405
2	21.532	4791	10.853	56114	9.734
3	21.973	4476	10.141	54419	9.440
4	22.308	13920	31.536	233002	40.420
Total		44142	100.000	576453	100.000

Supplementary Figure 499. Racemic Chromatogram of compound **102**

<Chromatogram>

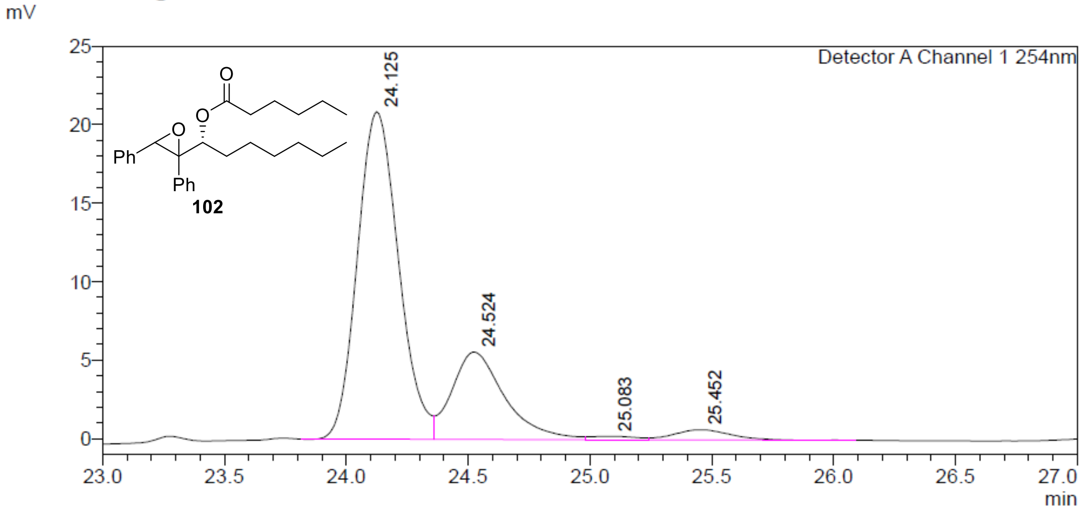

<Peak Table>

Detector A Channel 1 254nm

Peak#	Ret. Time	Height	Height%	Area	Area%
1	24.125	20855	76.406	245378	71.636
2	24.524	5565	20.387	83988	24.520
3	25.083	225	0.823	2821	0.824
4	25.452	651	2.384	10347	3.021
Total		27295	100.000	342534	100.000

Supplementary Figure 500. Scalemic Chromatogram of compound **102**

<Chromatogram>

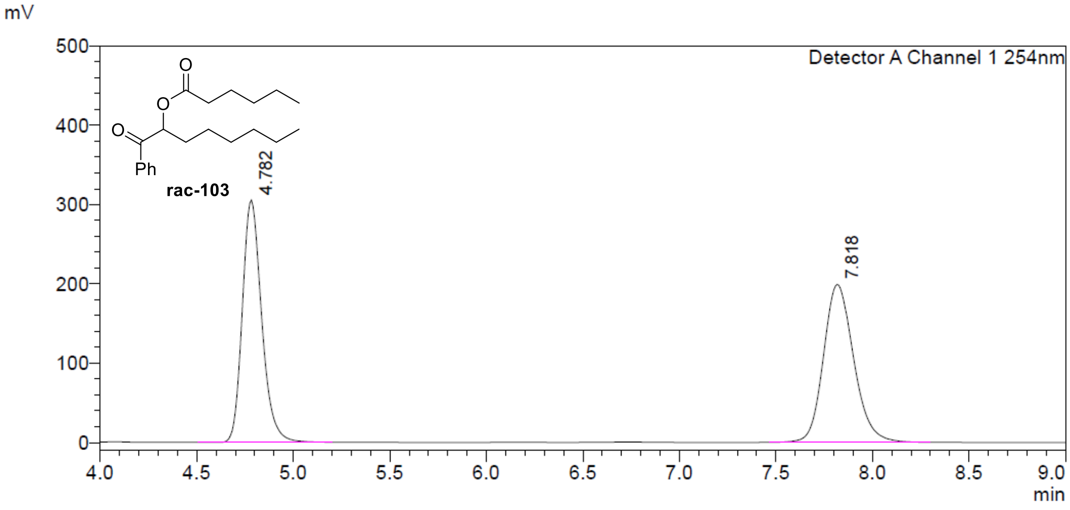

<Peak Table>

Detector A Channel 1 254nm

Peak#	Ret. Time	Height	Height%	Area	Area%
1	4.782	305483	60.541	2137946	49.608
2	7.818	199102	39.459	2171734	50.392
Total		504584	100.000	4309680	100.000

Supplementary Figure 501. Racemic Chromatogram of compound **103**

<Chromatogram>

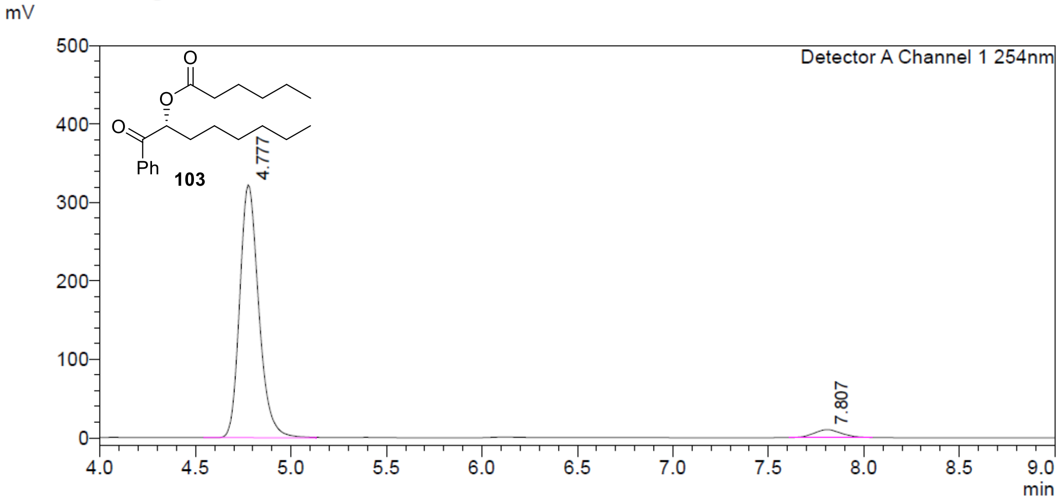

<Peak Table>

Detector A Channel 1 254nm

Peak#	Ret. Time	Height	Height%	Area	Area%
1	4.777	322428	96.990	2247983	95.737
2	7.807	10005	3.010	100094	4.263
Total		332433	100.000	2348077	100.000

Supplementary Figure 502. Scalemic Chromatogram of compound **103**

<Chromatogram>

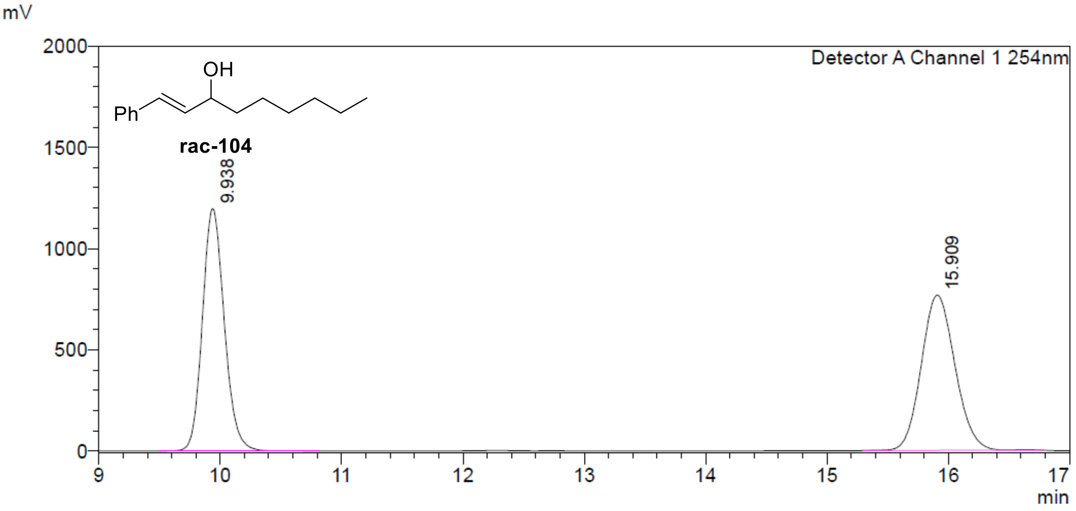

<Peak Table>

Detector A Channel 1 254nm

Peak#	Ret. Time	Height	Height%	Area	Area%
1	9.938	1196196	60.935	14977745	50.198
2	15.909	766871	39.065	14859315	49.802
Total		1963067	100.000	29837060	100.000

Supplementary Figure 503. Racemic Chromatogram of compound **104**

<Chromatogram>

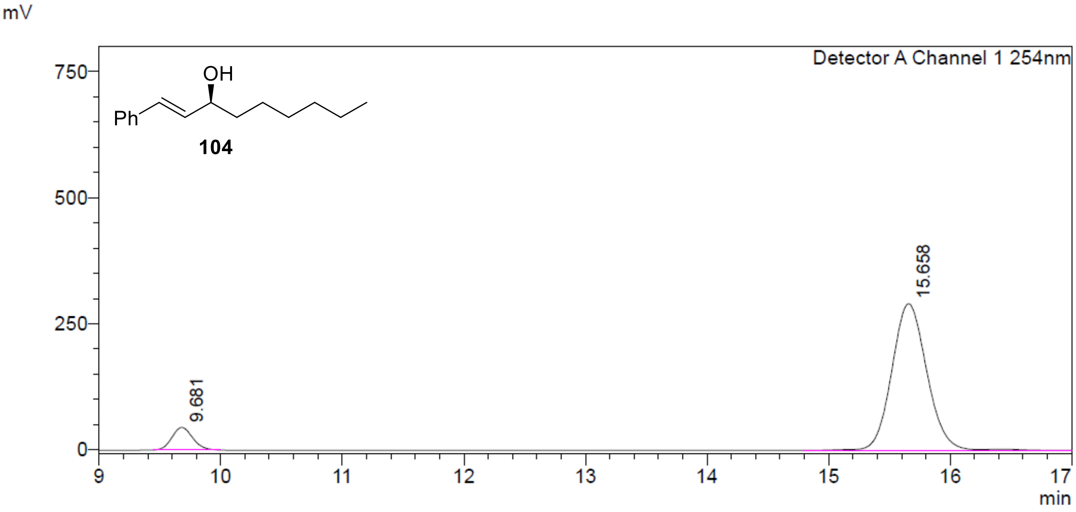

<Peak Table>

Detector A Channel 1 254nm

Peak#	Ret. Time	Height	Height%	Area	Area%
1	9.681	44635	13.313	529847	8.325
2	15.658	290642	86.687	5834623	91.675
Total		335277	100.000	6364470	100.000

Supplementary Figure 504. Scalemic Chromatogram of compound **104**

<Chromatogram>

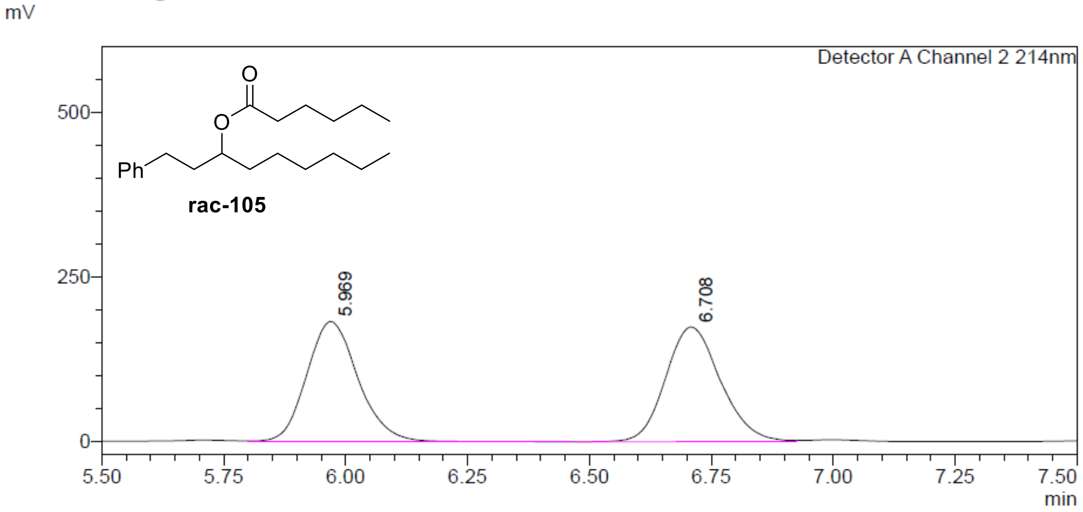

<Peak Table>

Detector A Channel 2 214nm

Peak#	Ret. Time	Height	Height%	Area	Area%
1	5.969	182623	51.168	1343348	49.570
2	6.708	174287	48.832	1366661	50.430
Total		356909	100.000	2710009	100.000

Supplementary Figure 505. Racemic Chromatogram of compound **105**

<Chromatogram>

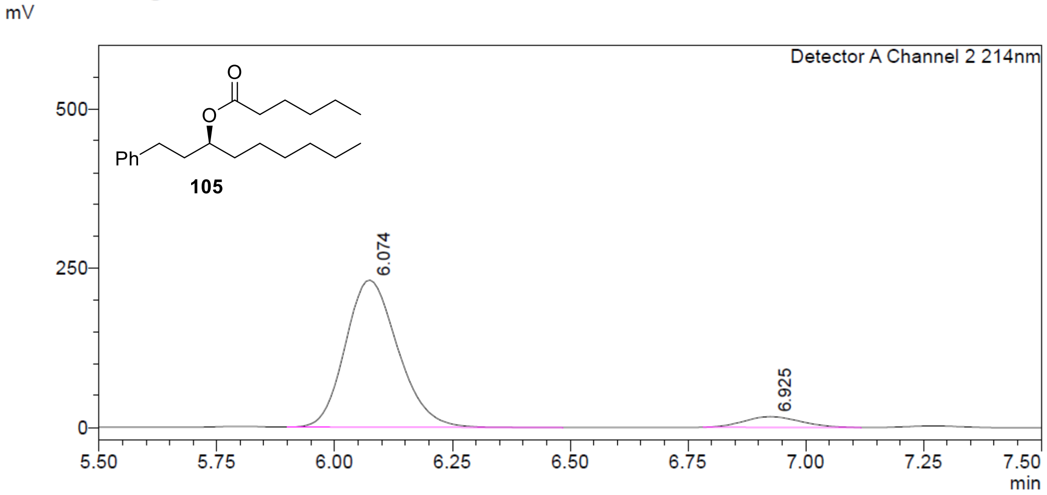

<Peak Table>

Detector A Channel 2 214nm

Peak#	Ret. Time	Height	Height%	Area	Area%
1	6.074	231168	93.206	1793196	92.747
2	6.925	16851	6.794	140236	7.253
Total		248019	100.000	1933432	100.000

Supplementary Figure 506. Scalemic Chromatogram of compound **105**

<Chromatogram>

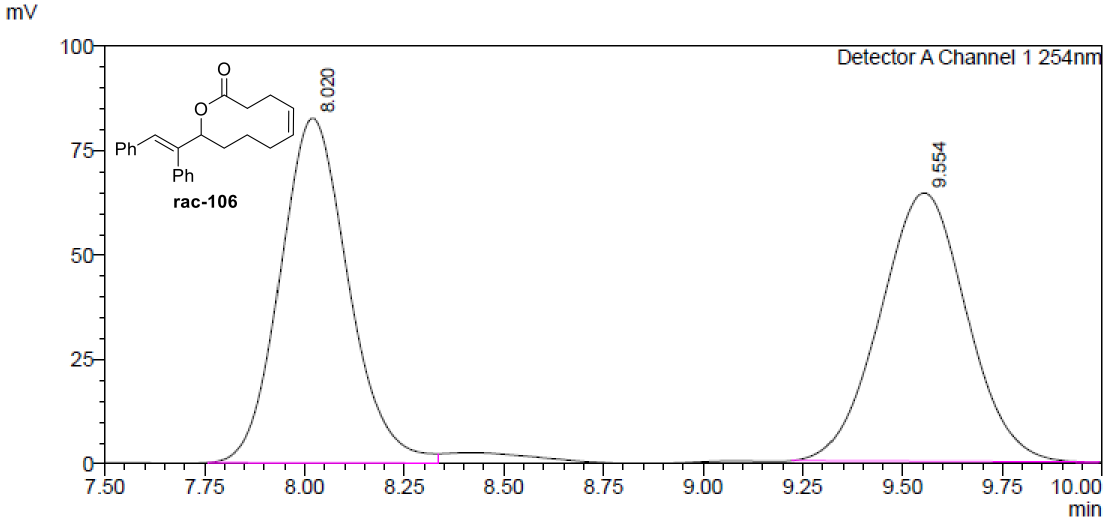

<Peak Table>

Detector A Channel 1 254nm

Peak#	Ret. Time	Height	Height%	Area	Area%
1	8.020	82526	56.193	981181	50.192
2	9.554	64337	43.807	973679	49.808
Total		146863	100.000	1954859	100.000

Supplementary Figure 507. Racemic Chromatogram of compound **106**

<Chromatogram>

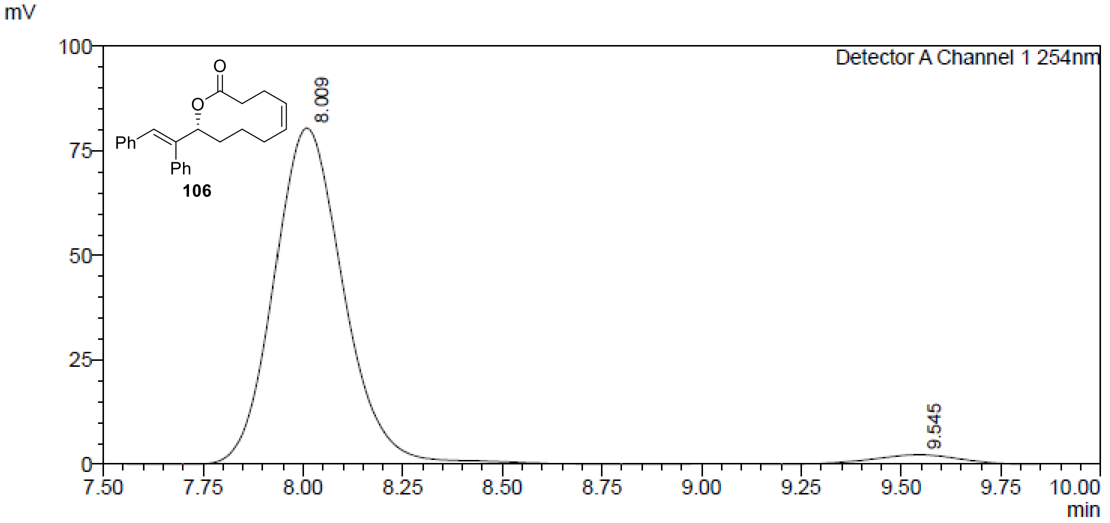

<Peak Table>

Detector A Channel 1 254nm

Peak#	Ret. Time	Height	Height%	Area	Area%
1	8.009	80589	97.152	979087	96.252
2	9.545	2363	2.848	38129	3.748
Total		82951	100.000	1017216	100.000

Supplementary Figure 508. Scalemic Chromatogram of compound **106**

II. Supplementary References

(1) Jian, W., Ge, L., Jiao, Y., Qian, B, Bao. H. Iron-Catalyzed Decarboxylative Alkyl Etherification of Vinylareneswith Aliphatic Acids as the Alkyl Source. *Angew. Chem. Int. Ed.* ***56***, 3650-3654, doi: 10.1002/anie.201612365 (2017).

(2) Mundal, D. A., Lutz, K. E., Thomson, R. J. Stereoselective Synthesis of Dienes from N-Allylhydrazones. *Org. Lett.* **2**, 465-468, doi: 10.1021/ol802585r (2008).

(3) Shao, L., Shi, M. Facile synthesis of multisubstituted buta-1,3-dienes *via* Suzukie Miyaura and Kumada cross-coupling strategy of 2,4-diiodobuta- 1-enes with arylboronic acids and Grignard reagents. *Org . Biomol. Chem*. **3**, 1828-1831, doi: 10.1039/b504071j (2005).

(4) Qiao, C., Chen, A., Gao, B., Liu, Y., Huang, H. Palladium-Catalyzed Cascade Double C-N Bond Activation: A New trategy for Aminomethylation of 1,3-Dienes with Aminals. *Chin. J. Chem.* **36**, 929-933, doi: 10.1002/cjoc.20180025 (2018).

(5) Macreadie, I. G., Avery, T. D., Robinson, T. V., Macreadie, P., Barraclough, M., Taylor, D. K., Tiekink, E. R. T. Design of 1,2-Dioxines with Anti-Candida Activity: Aromatic Substituted 1,2-Dioxines. *Tetrahedron* **64**, 1225-1232, doi:10.1016/j.tet.2007.11.071 (2007).

(6) Preuβ, T., Saak, W., Doye, S. Titanium-Catalyzed Intermolecular Hydroaminoalkylation of Conjugated Dienes. *Chem. Eur. J.* **19**, 3833-3837, doi: 10.1002/chem.201203693 (2013).

(7) Tortajada, A., Ninokata, R., Martin R. Ni-Catalyzed Site-Selective Dicarboxylation of 1,3-Dienes with CO_2. *J. Am. Chem. Soc.* **140**, 2050-2053, doi:10.1021/jacs.7b13220 (2018).

(8) Marcum, J. S., Taylor, T. R., Meek S. J. Enantioselective Synthesis of Functionalized Arenes by Nickel-Catalyzed Siteselective Hydroarylation of 1,3-Dienes with Aryl- Boronates. *Angew. Chem. Int. Ed.* **59**, 14070-14075, doi:10.1002/anie.202004982 (2020).

(9) Della-Felice, F., Assis, F., Sarotti, A. M., Pilli, R. A. Palladium-Catalyzed Formation of Substituted Tetrahydropyrans: Mechanistic Insights and Structural Revision of Natural Products. *Synthesis* **51**, 1545-1560, doi: 10.1055/s-0037-1611708 (2018).

(10) Takashima, Y., Kobayashi, Y. Synthesis of (S)-Imperanene by Using Allylic Substitution. *J. Org. Chem.* **74**, 5920-5926, doi: 10.1021/jo900854p (2008).

(11) Harsh, P., Oarsh, P. De Novo Asymmetric Syntheses of (+)-Goniothalamin, (+)-Goniothalamin Oxide, and 7,8-Bis-*epi*-goniothalamin Using Asymmetric Allylations. *Tetrahedron* **65**, 5051-5055, doi: 10.1016/j.tet.2009.03.097 (2009).

(12) Bogar, K., Vidal, P. H., Leon, A. R. A., Backvall, J. Chemoenzymatic Dynamic Kinetic Resolution of Allylic Alcohols: A Highly Enantioselective Route to Acyloin Acetates. *Org. Lett.* **17**, 3401-3404, doi: 10.1021/ol071395v (2007).

(13) Ariza, X., Garcia, J., Georges, Y., Vicente M. 1-Phenylprop-2-ynyl Acetate: A Useful Building Block for the Stereoselective Construction of Polyhydroxylated Chains. *Org. Lett.* **20**, 4051-4054, doi: 10.1021/ol0616539 (2006).

(14) Inoue, M., Nakada, M. Structure Elucidation and Enantioselective Total Synthesis of the HMG-CoA Reductase Inhibitors FR901512 and FR901516. *Synthesis* **21**, 3694-3707, doi: 10.1055/s-0029-1216980 (2009).
